# An Exposure-Response Perspective on the Clinical Dose of Pretomanid

**DOI:** 10.1128/AAC.01121-20

**Published:** 2020-12-16

**Authors:** Jerry R. Nedelman, David H. Salinger, Vishak Subramoney, Rob Woolson, Karen Wade, Mengchun Li, Daniel Everitt, Carl M. Mendel, Mel Spigelman

**Affiliations:** a TB Alliance, New York, New York, USA; b Certara Inc., Princeton, New Jersey, USA; c Certara Strategic Consulting, Quebec, Canada; d Rho, Raleigh, North Carolina, USA

**Keywords:** antimicrobial agents, pretomanid, tuberculosis

## Abstract

Pretomanid was approved by the U.S. FDA, via the limited population pathway for antibacterial and antifungal drugs, as part of a three-drug regimen with bedaquiline and linezolid for the treatment of extensively drug-resistant and treatment-intolerant or nonresponsive multidrug-resistant tuberculosis. The recommended dose of pretomanid is 200 mg once daily with food. The objective of this work was to retrospectively evaluate this recommended dose by means of exposure-response (E-R) modeling applied to outcomes of both efficacy and safety. Cox proportional-hazards modeling was used, with the steady-state average pretomanid concentration as the exposure metric. The efficacy outcome was time to sputum culture conversion (TSCC) to negative. The safety outcomes were times to the first occurrence of adverse events in classes selected from either pretomanid’s investigator brochure or the new drug application (NDA) submission as recognized safety signals for pretomanid based on preclinical as well as clinical experience. Significant E-R relationships were found for TSCC and two adverse-event classes, vomiting (a single preferred term) and gastrointestinal (GI) symptoms (a collection of related terms). No significant E-R relationships were found for the single preferred terms nausea, alanine aminotransferase (ALT) increased, aspartate aminotransferase (AST) increased, and headache and for the collections hepatic disorders, transaminases increased, skin and subcutaneous tissue disorders, and headache. The results suggest that the recommended dose of pretomanid, 200 mg given in the fed state, is appropriate over the range of pharmacokinetic exposures.

## Abstract (precis)

Pretomanid was approved by the U.S. FDA, via the limited population pathway for antibacterial and antifungal drugs, as part of a three-drug regimen with bedaquiline and linezolid for the treatment of extensively drug-resistant and treatment-intolerant or nonresponsive multidrug-resistant tuberculosis. The recommended dose of pretomanid is 200 mg once daily with food. The objective of this work was to retrospectively evaluate this recommended dose by means of exposure-response (E-R) modeling applied to outcomes of both efficacy and safety.

## INTRODUCTION

Nix-TB (1), an ongoing clinical trial testing a 6- to 9-month, 3-drug oral regimen (bedaquiline, pretomanid, and linezolid [BPaL]) in extensively drug-resistant (XDR) and treatment-intolerant or nonresponsive (TI/NR) multidrug-resistant tuberculosis (MDR-TB), recently reported a treatment success rate of 90%. This study was the pivotal trial for U.S. FDA registration of pretomanid, as part of the three-drug regimen, via the limited population pathway for antibacterial and antifungal drugs (17) for treatment of this TB population.

In the FDA label, the recommended dose for pretomanid is 200 mg once daily (QD) with food, as part of the BPaL regimen. The steps in the development program that led to this recommendation were as follows.

A single-ascending-dose study (2) established the tolerability of pretomanid doses from 50 to 1,500 mg. A 7-day multiple-ascending-dose study (2) established the tolerability of daily doses of 200, 600, and 1,000 mg. The 1,000-mg cohort was stopped after the 5th of 7 planned days because of increased serum creatinine levels, but such elevations were later demonstrated to be attributable to inhibition of the tubular secretion of creatinine and not to negative effects on glomerular filtration (3).

The early bactericidal activity (EBA) of pretomanid was evaluated in two 14-day studies in TB patients. The first one (4) assessed doses from 200 to 1,200 mg QD. All doses exhibited bactericidal activity with no dose response. The next study (5) assessed lower ranges of 50, 100, 150, and 200 mg QD. A test for the equality of the response at 50 mg to the mean of the responses at the higher three doses had *P* values of 0.0605 for EBA based on CFU counts and 0.0511 for EBA based on time to positivity (TTP) in liquid culture, which may be interpreted as weak evidence that 50 mg had a lower EBA. Pretomanid was judged safe and well tolerated, although the incidence of adverse events (AEs) potentially related to pretomanid appeared dose related. Thus, the range of 100 to 200 mg was identified for further evaluation of pretomanid in novel antituberculosis regimens.

In the studies discussed above, pretomanid was administered in the fasting state. Two food-effect studies (6) found a dose-dependent effect of a high-fat, high-calorie meal in healthy volunteers. At 200 mg, the maximum concentration of drug in plasma (*C*_max_) and the area under the concentration-time curve from 0 h to infinity (AUC_0–∞_) increased by 76% and 88%, respectively, for fed versus fasted subjects. A population pharmacokinetic (PopPK) model (7) based on 14 studies in patients, including phase 2 and 3 studies, found an increase in bioavailability of 95% for fed versus fasted subjects at 200 mg.

Next, in the phase 2b study NC-002 (8), pretomanid (Pa) was dosed for 8 weeks combined with 400 mg QD of moxifloxacin (M) and 1,500 mg QD of pyrazinamide (Z). Drug-sensitive TB (DS-TB) patients were randomized to two PaMZ arms, one with 100 mg QD of pretomanid (Pa_100_MZ) and one with 200 mg (Pa_200_MZ), as well as a third arm of the standard of care, HRZE (isoniazid, rifampicin, pyrazinamide, and ethambutol). Dosing was to be at least 2 h after a meal, preferably breakfast; the exposures were generally consistent with expectations for the fed state (7). The 8-week bactericidal activities of the three arms were numerically ordered, Pa_200_MZ > Pa_100_MZ > HRZE. Pa_200_MZ and HRZE were statistically significantly different, but no other paired comparisons were. Both pretomanid regimens were well tolerated and safe, with adverse events generally being similarly distributed.

Based on the totality of the available results discussed above, a 200-mg QD dose of pretomanid was selected as part of the BPaL regimen in the Nix-TB study. The protocol specified administration with food because bedaquiline must be so administered.

Another phase 3 study, STAND (ClinicalTrials.gov identifier NCT02342886), overlapped in time with Nix-TB, so its results were not available to inform dose selection for Nix-TB. STAND randomized drug-sensitive TB patients to 4 arms: HRZE for 6 months, PaMZ with 100 mg of pretomanid for 4 months (4Pa_100_MZ), PaMZ with 200 mg of pretomanid for 4 months (4Pa_200_MZ), and PaMZ with 200 mg of pretomanid for 6 months (6Pa_200_MZ). It was designed as a noninferiority study of PaMZ versus HRZE based on “favorable response,” defined as sputum culture negativity at the end of treatment followed by relapse-free survival for at least 6 months. It was stopped early when evidence from the phase 2b study NC-005 (9) suggested that adding bedaquiline to PaMZ (BPaMZ) substantially improved efficacy. *De facto* underpowered, STAND did not conclude noninferiority. However, among the pretomanid-containing arms, there was a trend favoring 200 mg, with favorable rates (numbers; 95% confidence intervals [CIs]) in the 4Pa_100_MZ, 4Pa_200_MZ, and 6Pa_200_MZ arms of 67% (57; 53% to 79%), 75% (61; 63% to 86%), and 77% (56; 64% to 87%), respectively, although the statistical significance of this trend was not formally tested. Food conditions at dosing times were not specified in the protocol, but observed trough concentrations were consistent with predictions for the fed state based on PopPK modeling (7).

The objective of this work was to retrospectively evaluate the recommended daily dose of pretomanid, 200 mg administered with food, by means of exposure-response (E-R) modeling applied to outcomes of both efficacy and safety.

Of particular interest was the importance of administration with food. In the fasted state, 200 mg yields exposures approximately one-half of those in the fed state, approximately the same as 100 mg administered with food. In the clinical studies considered here, which included some arms with 100 mg of pretomanid, either the protocols specified administration with food or the exposures were consistent with the fed state.

Data were pooled from subjects in four studies and four pretomanid-containing regimens: NC-002 (PaMZ), NC-005 (BPaZ and BPaMZ), STAND (PaMZ), and Nix-TB (BPaL). NC-002 and NC-005 were 8-week phase 2b studies of bactericidal activity, whereas STAND and Nix-TB were 6-month phase 3 studies assessing rates of favorable response, as defined above for STAND. The time to sputum culture conversion (TSCC) determined in liquid culture was reported in all four studies; it was used as the efficacy variable for E-R modeling here.

Although data from all four studies and regimens were used to increase power in model building, interpretation of the models primarily focused on the BPaL regimen in a Nix-TB-like population because this is the currently approved use of pretomanid. Because of primary interest in the BPaL regimen, where only the 200-mg fed dose of pretomanid was used, it was hoped that E-R modeling, including data from regimens where the 100-mg fed dose was used, could reveal the expected behavior for BPaL at the 100-mg fed or 200-mg fasted dose.

Regarding safety, AEs were modeled. E-R modeling of QT prolongation associated with pretomanid has been reported previously (10). Ten sets of AEs were considered. These were based on recognized safety signals for pretomanid from nonclinical as well as clinical experience and were identified either as adverse drug reactions in pretomanid’s investigator brochure or as AEs of special interest in the new drug application (NDA) submission. A set was included if at least 10% of the subjects in the data pool had such treatment-emergent events. These sets (and the percentages of subjects experiencing them) were as follows:

• Single preferred terms
  ∘ Nausea (17.6%)
  ∘ Vomiting (14.7%)
  ∘ Alanine aminotransferase (ALT) increased (14.3%)
  ∘ Aspartate aminotransferase (AST) increased (14.0%)
  ∘ Headache (10.7%)

• Sets of preferred terms (see Section S3 in the supplemental material for details)
  ∘ Gastrointestinal (GI) symptoms (28.4%)
  ∘ Hepatic disorders (25.5%)
  ∘ Transaminases increased (19.2%)
  ∘ Skin and subcutaneous tissue disorders (16.6%)
  ∘ Headache (11.0%)

For both TSCC and AEs, Cox proportional-hazards (PH) models were applied. For AEs, the time to the first occurrence of an event in the set was considered. The exposure variable was the steady-state pretomanid average concentration (*C*_avg_), as determined by a population pharmacokinetic model (7). Other covariates were also considered, regimen, age, gender, body mass index (BMI), HIV status, and TB type (DS, MDR, or XDR), for both efficacy and safety; additionally, the presence/absence of cavities and baseline time to positivity (BTTP) in liquid culture were considered for efficacy.

## RESULTS

### Efficacy: time to sputum culture conversion.

The efficacy analysis data set contained 619 subjects from the four pooled studies, with 454 events, i.e., observed sputum conversions to negative. Data summaries are provided in Section S0 in the supplemental material.

**FIG 1:**
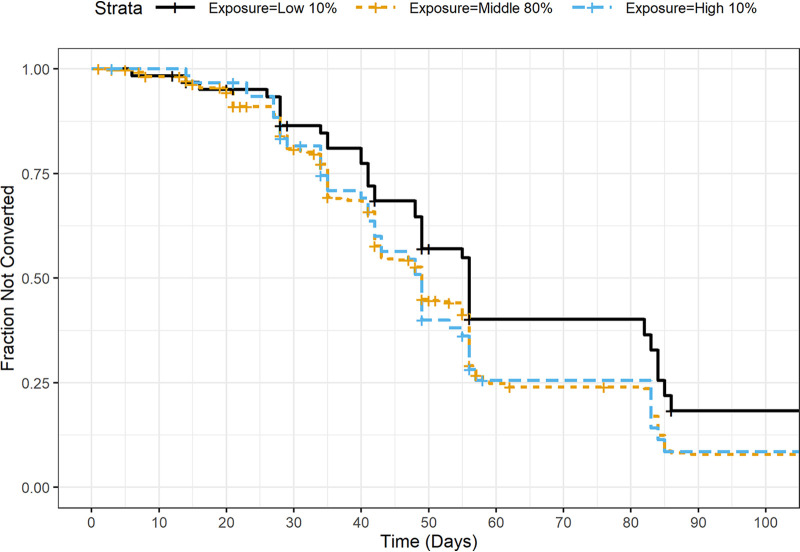
Kaplan-Meier plots of time to sputum culture conversion, by quantiles of *C*_avg_.

Figure 1 shows Kaplan-Meier plots stratified by the quantile of *C*_avg_ (lower 10%, middle 80%, and upper 10%), suggesting that patients with lower exposures may have a longer time to conversion. A PH model with regimen and log-transformed *C*_avg_ as predictors found a significant effect of *C*_avg_ (*P* = 0.005). Based on these results, more extensive modeling, as described in Materials and Methods, was undertaken.

In the final model, exposure was represented by a natural spline with 3 degrees of freedom (df) in log-transformed *C*_avg_. Age and BTTP were retained as covariates along with exposure and regimen, with an interaction between age and exposure. Violation of PH was found for BTTP. Schoenfeld residuals indicated that the impact of BTTP decreased with time; after around 60 days, if a subject’s sputum had not yet converted, BTTP was no longer predictive of conversion. A time-dependent coefficient for BTTP was introduced to account for this. See Section S1 in the supplemental material for details; parameter estimates for the final model can be found on p. 17 in Section S1.

**FIG 2:**
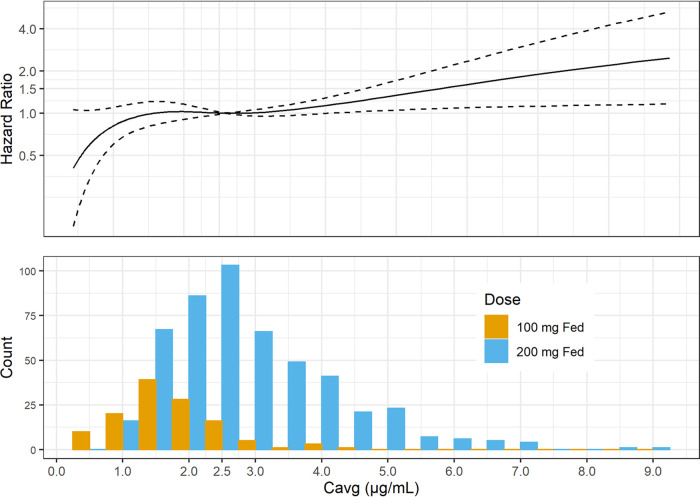
Hazard ratios of TSCC versus *C*_avg_ and distribution of *C*_avg_. Note that in the top panel, the solid curve shows estimated hazard ratios as a function of *C*_avg_ relative to the value at a *C*_avg_ of 2.6 μg/ml, where the ratio, by definition, is 1. Larger values of the hazard ratio imply a faster time to culture conversion. The dashed curves show the pointwise 95% confidence intervals for the hazard ratios.

Figure 2 displays hazard ratios for TSCC versus *C*_avg_, relative to the median *C*_avg_ in Nix-TB, 2.6 μg/ml, for a subject with the median BTTP and age in Nix-TB, 278 h and 35 years, respectively. Because the modeling found no significant interaction between regimen and exposure, the hazard ratios depicted in the figure apply to all regimens; that is, the absolute rates of occurrence of TSCC differ among the regimens, but the ratio of the rate at a given *C*_avg_ to the rate at a *C*_avg_ of 2.6 μg/ml is the same for all regimens, and such ratios are what are plotted in Fig. 2. The figure also shows the distribution of *C*_avg_ values in the analysis data set, by dose. These are *post hoc* estimates from the PopPK model (7), and thus, they represent exposures arising from the distribution of demographics and other baseline conditions of patients in the four pooled studies.

The hazard ratios are relatively constant, or perhaps slightly increasing, over the range of exposures for the 200-mg fed dose. Toward the lower extremes of exposure for the 100-mg fed dose, the hazard ratios trend lower, although the confidence intervals include unity. The distribution of the 100-mg fed dose approximates what would be expected for the 200-mg fasted dose.

**FIG 3:**
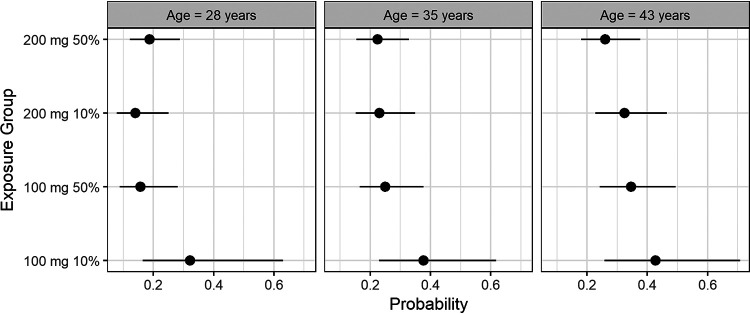
Estimated probabilities of failure to culture convert by 8 weeks and 95% confidence intervals, by age and exposure.

Culture conversion at 8 weeks is often used as a biomarker in phase 2 studies for judging new regimens. The model was used to estimate the probability of failure to convert by week 8 (F8W) for the 25th, 50th, and 75th percentiles of age among the subjects from Nix-TB and for the 10th and 50th percentiles of *C*_avg_ for the 100-mg and 200-mg fed doses. For *C*_avg_, the percentiles for the 200-mg fed dose were determined from the Nix-TB subjects, and the percentiles for 100-mg fed or, equivalently, the 200-mg fasted dose were then set at half the values for the 200-mg fed dose. BTTP was the median value for Nix-TB. Thus, Fig. 3 represents a characterization of a Nix-TB-like population, to the extent possible, even for the 100-mg dose.

Consistent with the shape of the hazard ratio curve, the probabilities of F8W were similar except at the lowest exposures. For the 10th percentiles of the 200-mg fed and 100-mg fed/200-mg fasted doses, the estimated probabilities (95% CIs) at the median age of 35 years were 0.23 (0.15 to 0.35) and 0.38 (0.23 to 0.62), respectively. Other values can be found in Section S1.8.

### Adverse events.

The safety analysis data set contained 644 subjects from the four pooled studies. Numbers of events by set can be found in Section S3.2.

Screening, as described in Materials and Methods, identified only two sets of AEs showing evidence of a relationship with exposure: vomiting and GI symptoms. For both, most events were of grade 1 or 2. Out of 130 and 344 total vomiting and GI symptom events, only 2 and 3 were of grade 3, respectively, and none were of grade 4. See Section S3.2 for details.

**FIG 4:**
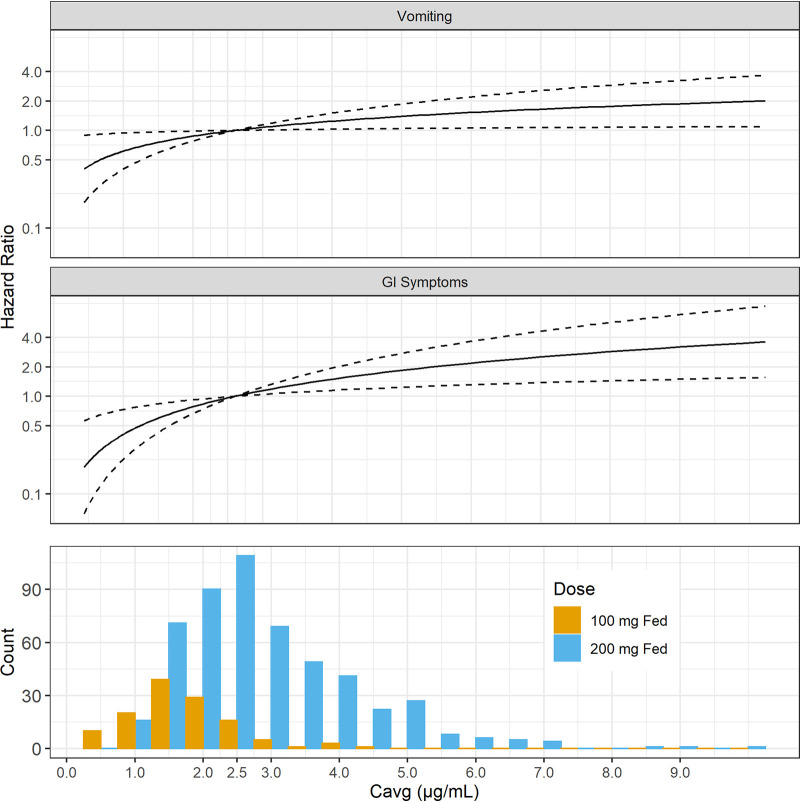
Hazard ratios of exposure-related adverse events versus *C*_avg_ and distribution of *C*_avg_. Note that in the top two panels, the solid curves show estimated hazard ratios as a function of *C*_avg_ relative to the value at a *C*_avg_ of 2.6 μg/ml, where the ratio, by definition, is 1. Larger values of the hazard ratio imply a faster time to adverse event occurrence. The dashed curves show the pointwise 95% confidence intervals for the hazard ratios. Conditions underlying the hazard ratios vary between the two AE types, depending on the optimal model found for each. Both are comparable to the data in Fig. 2 in that they apply to the BPaL regimen, although data for other regimens were used to build the model for vomiting, whereas the model for GI symptoms is based on only the BPaL regimen. See the text for more details.

Figure 4 displays hazard ratios for the two exposure-related AEs as was done in Fig. 2 for TSCC. Specific covariate conditions depend on the final model in each case, as described in the subsections below.

**(i) Vomiting.** For vomiting, there were 96 first events. In the final model, exposure was represented as a linear function of log-transformed *C*_avg_. Gender was retained as a covariate along with exposure and regimen, and it was changed to a stratification variable because it violated PH. There were no interactions.

The hazard ratios in Fig. 4 apply to all subjects on a given regimen and of a given gender.

The risk for vomiting was higher among females. For BPaL, the estimated probabilities (95% CIs) of at least one event during 6 months of exposure at the 50th percentile of a 200-mg pretomanid fed dose were 0.27 (0.17 to 0.36) and 0.40 (0.28 to 0.51) for males and females, respectively. For females, the estimated probability decreased to 0.30 (0.16 to 0.42) at the 50th percentile of the 100-mg fed/200-mg fasted dose, and a similar 25% decrease applied for males. See Section S4 for more details; parameter estimates for the final model can be found on p. 39 in Section S4.

**(ii) Gastrointestinal symptoms.** For gastrointestinal symptoms, there were 185 first events. Initial models indicated that gender was a significant covariate and that both regimen and gender violated PH. Stratifying on both and testing interactions with exposure produced an overparameterized model that failed to converge. The data set was reduced to only the BPaL regimen, which had 53 first events. In the final model, neither gender nor any other covariate was retained, except for exposure, which was represented as a linear function of log-transformed *C*_avg_; parameter estimates for the final model can be found on p. 47 of Section S5.

In Fig. 4, the hazard ratios are thus for the BPaL regimen.

For BPaL, the estimated probabilities (95% CIs) of at least one event of GI symptoms during 6 months of exposure at the 50th percentiles of the 200-mg fed and 100-mg fed/200-mg fasted doses were 0.49 (0.38 to 0.57) and 0.29 (0.14 to 0.42), respectively. See Section S5 for more details.

## DISCUSSION

Evidence for dependence on pretomanid exposure has been found for the efficacy biomarker TSCC and for the adverse-event sets vomiting and GI symptoms.

Associations with other covariates have also been found, and in some cases, these associations affected the E-R relationship: age and baseline TTP for TSCC, and gender for vomiting and GI symptoms. The regimen was also controlled for in the models, although in one case, GI AEs, model complexity required a reduction of the model to only the Nix-TB regimen. Cavitation was not found to be a significant covariate, although others have found it to be associated with poor prognoses (11).

The primary objective here was to evaluate pretomanid’s recommended clinical dose, 200 mg with food, in light of E-R relationships for both efficacy and safety.

Regarding safety, 8 of 10 sets of AEs previously identified based on safety signals for pretomanid, including 4 related to hepatotoxicity, exhibited no significant exposure-response relationship. Thus, for these cases, there is no evidential basis for reducing exposure to improve safety. Two of the 10, both GI related, exhibited significant and monotonically increasing relationships between the pretomanid *C*_avg_ and the probability of experiencing at least one such event. The probability for vomiting was predicted to decrease by 25% with halving exposure, and GI symptoms were predicted to decrease by 40%. However, almost all such events were of grade 1 or 2.

Regarding efficacy, the results here are more difficult to interpret because only a biomarker, TSCC, was used as the efficacy outcome. TSCC exhibited a significant exposure-response relationship, but the relationship was relatively flat over a range that covered most observed exposures, not only at the 200-mg fed but also at the 100-mg fed dose, which approximates the 200-mg fasted dose. F8W may be derived from TSCC. There is some evidence that F8W may be predictive of unfavorable clinical outcomes (12–15). The model developed here for TSCC estimated the probabilities of F8W at the 10th percentiles of the 200-mg fed and 100-mg fed/200-mg fasted doses to be 0.23 and 0.38, respectively. Substituting these values into the model of Wallis et al. (12, 13) for a 6-month regimen produced estimated probabilities of recurrence of 0.077 and 0.103, for a 34% increase at the lower or fasted dose. A Bayesian categorical-data analysis based on the data from Nix-TB similarly predicted probabilities (95% credible intervals) of an unfavorable outcome of 0.119 (0.064 to 0.180) and 0.139 (0.066 to 0.212). See Section S2 in the supplemental material for more details.

In the prematurely terminated STAND study, the 4Pa_100_MZ and 4Pa_200_MZ arms had unfavorable rates (95% CIs) of 0.333 (0.214 to 0.471) and 0.246 (0.145 to 0.373), respectively (ClinicalTrials.gov identifier NCT02342886). The confidence intervals largely overlap, and the regimen and patient population differ from those for BPaL in Nix-TB, but the results suggest the possibility that a dose reduction from 200 mg to 100 mg may have a larger impact on unfavorable outcomes than what is indicated by projections from F8W.

Thus, it seems that halving exposure from that of the 200-mg fed dose to either the 100-mg fed or 200-mg fasted dose may have impacts on both safety and efficacy. The value trade-off is not obvious, but in our experience, most practitioners would be reluctant to sacrifice even a small degree of efficacy unless absolutely necessary. Nevertheless, allowing administration of the 200-mg dose “with or without food” could perhaps be considered under some circumstances. Specifically, as an example, these results might suggest that a temporary period of administration under fasting conditions may help patients suffering from vomiting or GI symptoms without overly compromising efficacy. However, this has not been tested.

Limitations of the modeling work reported here should be noted. (i) It was retrospective and data driven, so the extracted signals are best interpreted as hypotheses in need of confirmation. (ii) *C*_avg_, the exposure metric that was used, may not be optimal for all outcomes. (iii) Note that in Fig. 3, the estimated probability of F8W was slightly higher for the age of 28 years for the 200-mg 50% exposure group than for the 200-mg 10% and 100-mg 50% groups. The spline-based approach to modeling used here offers the advantage of great flexibility but pays the price of not requiring that the hazard rate increase monotonically with *C*_avg_, as might be expected on pharmacological grounds. (iv) Regimen, TB type, and study were highly confounded because of different regimens used for different populations in different studies. (v) Adverse events where no exposure-response was observed are not necessarily unrelated to pretomanid exposure; it is possible that the impact of pretomanid was already maximized over the range of exposures available. (vi) Only the time to the first occurrence of each adverse event type was considered (counts of all and first occurrences of each event type can be found in Section S3).

Overall, these results suggest that the recommended dose of pretomanid, 200 mg given in the fed state, is appropriate over the range of pharmacokinetic exposures experienced by patients in clinical trials of pretomanid.

## MATERIALS AND METHODS

All analyses were performed using R v3.6.2 in addition to CRAN packages (16).

### Data.

Data came from four clinical studies: NC-002 (8) and NC-005 (9), two phase 2b studies of an 8-week duration with bactericidal activity as the primary outcome, and NC-006 (ClinicalTrials.gov identifier NCT02342886) and Nix-TB (1), two phase 3 studies with favorable response as the primary outcome. Only patients on pretomanid-containing treatments were included.

In all four studies, TSCC was based on sputum samples tested in liquid cultures using the mycobacterial growth indicator tube (Bactec MGIT960) system.

Sputum culture and adverse events were assessed according to the following schedules: for NC-002, including the EBA-PK substudy, AEs were assessed on days 1, 2, 3, 4, 5, 6, 7, 8, 9, 10, 11, 12, 13, 14, 15, 22, 29, 36, 43, 50, and 57, and sputum was assessed on days (on which overnight sputum collection began) −1, 1, 2, 3, 5, 7, 9, 11, 14, 21, 28, 35, 42, 49, and 56; for NC-002, excluding the EBA-PK substudy, AEs were assessed on days 1, 4, 11, 15, 22, 29, 36, 43, 50, and 57, and sputum was assessed on days −1, 3, 7, 14, 21, 28, 35, 42, 49, and 56; for NC-005, AEs were assessed on days 1, 4, 8, 14, 15, 22, 29, 36, 43, 50, 56, and 57, and sputum was assessed on days −1, 3, 7, 14, 21, 28, 35, 42, 49, and 56; for NC-006, AEs were assessed on day 1, weekly from weeks 1 to 8, and then at weeks 12, 17, 22, and 26, and sputum was assessed on day −1, weekly from weeks 1 to 8, and then at weeks 12, 17, 22, and 26; and for Nix-TB, AEs were assessed on day 1, weekly from weeks 1 to 16, and then at weeks 20 and 26 (and weeks 30, 34, and 39 for 9-month treatment), and sputum was assessed on day −1 and at weeks, 2, 4, 6, 8, 12, 16, 20, and 26 (and weeks 30, 34, and 39).

### Data analysis.

For each outcome, data analysis proceeded through four or five steps:

1. Initial screen. Evidence was sought for a relationship of the outcome with exposure by four explorations:
  a. Kaplan-Meier plots with strata comprising the lower 10%, the middle 80%, and the upper 10% of *C*_avg_.
  b. A Cox PH model with regimen and *C*_avg_ as covariates.
  c. Box plots of *C*_avg_ by the occurrence versus nonoccurrence of the event. For TSCC, only the first 8 weeks of treatment were considered.
  d. A linear model for log-transformed *C*_avg_ with regimen and occurrence versus nonoccurrence of the event as predictors.

If any of these four explorations indicated that there was a relationship between exposure and outcome, then subsequent steps were undertaken.

1. Covariate selection. Exposure was represented by a 4-df natural spline in log-transformed *C*_avg_ centered at 2.6 μg/ml, the median value in the BPaL regimen. A Cox PH model was fitted with the four spline terms, regimen, and the other covariates age (log transformed and centered at 35 years), BMI (log -transformed and centered at 20 kg/m^2^), gender, HIV status, and TB type (DS, MDR, or XDR). Regimen and the four exposure terms were not considered for elimination. Other covariates with *P* values of >0.05 by Wald tests were removed. A reduced model without the removed covariates was fitted and compared with the full model by a likelihood ratio test to confirm the lack of joint significance of the eliminated terms. PH was then tested on the resulting model. If PH failed for categorical covariates, they were changed to stratification variables. If PH failed for continuous variables, remediation was implemented as guided by Schoenfeld residuals. To test PH, the cox.zph function of the R survival package was used.
2. Covariate interactions. Two-way interactions among covariates retained in step 2 were tested. PH was tested for any models with retained interactions.

For adverse events, step 5 was the next step. For TSCC, step 4 and then step 5 were undertaken.

1. For TSCC, cavitation and then baseline TTP (log transformed and centered at 278 h) were tested by addition to the current best model. These covariates were handled separately because subjects with missing values were removed from the data set.
2. In the best model determined through step 4, the 4-df natural spline for exposure was replaced by splines with 1, 2, and 3 df. The best of these four models was selected based on the Akaike information criterion (AIC). This was the final model. If a 1-df spline was selected, which is equivalent to a linear function, the model was reparameterized with centered, log-transformed *C*_avg_ as the exposure variable rather than the corresponding spline basis function.

### Prediction of clinical outcome from failure to convert at 8 weeks.

Two methods were used for the prediction of clinical outcome from failure to convert at 8 weeks. One was the model of Wallis et al. (13) (see Section S2.1 in the supplemental material).

The other was a Bayesian analysis of the following data from Nix-TB.

Consider the seven non-NA (not applicable) numeric entries in Table 1 to be a sample from a multinomial distribution with probabilities, in order from left to right and top to bottom, *p*_PCF_, *p*_PCU_, *p*_PSF_, *p*_PSU_, *p*_PDU_, *p*_N-F_, and *p*_N-U_, where the subscripts represent category levels: P, positive; N, negative; C, converted; S, still positive; D, died; -, not applicable; F, favorable; U, unfavorable. A joint posterior distribution for these probabilities was determined conditionally on the data for Nix-TB in Table 1 by assuming a uniform Dirichlet prior. From this joint posterior, the conditional posterior distribution was determined for *P*(unfavorable outcome) = *p*_PCU_ + *p*_PSU_ + *p*_PDU_ + *p*_N-U_, given that the value of *P*(F8W) = (*p*_PSF_ + *p*_PSU_)/(*p*_PCF_ + *p*_PCU_ + *p*_PSF_ + *p*_PSU_) is (approximately) 0.23 or 0.38, the values estimated from the TSCC model for the 200- mg fed and 100-mg fed/200-mg fasted doses, respectively. For more details, see Section S2.2 in the supplemental material.

**TABLE 1:** Distribution of patients in the modified intent-to-treat population of Nix-TB at baseline and week 8 and final outcomes^*a*^

Baseline status	Wk 8 status	No. of patients with final outcome	
		Favorable	Unfavorable
Positive	Converted	70	2
	Still positive	12	3
	Died	NA	4

Negative	NA	16	0

a The intent-to-treat (ITT) population comprised all patients excluding late screening failures. The modified intent-to-treat (MITT) population allowed other exclusions (see https://www.nejm.org/doi/suppl/10.1056/NEJMoa1901814/suppl_file/nejmoa1901814_protocol.pdf). However, only two patients in the ITT population were excluded from the MITT population. NA, not applicable.

## Supplementary Material

https://www.nejm.org/doi/suppl/10.1056/NEJMoa1901814/suppl_file/nejmoa1901814_protocol.pdf

Supplemental file 1
