# Supplementary material for: An Exposure-Response Perspective on the Clinical Dose of Pretomanid
Source: Antimicrob Agents Chemother. 2020 Dec 16;65(1):e01121-20. doi: 10.1128/AAC.01121-20 (PMC7927811; doi:10.1128/AAC.01121-20)
Supplement: https://www.nejm.org/doi/suppl/10.1056/NEJMoa1901814/suppl_file/nejmoa1901814_protocol.pdf [file NEJMoa1901814_nejmoa1901814_protocol.pdf]

Protocol

This trial protocol has been provided by the authors to give readers additional information about their work.

Protocol for: Conradie F, Diacon AH, Ngubane N, et al. Treatment of highly drug-resistant pulmonary tuberculosis. N Engl J Med 2020;382:893-902. DOI: 10.1056/NEJMoa1901814

Supplement to the Nix-TB Trial

This supplement contains the following items:

1. Original protocol, final protocol, summary of changes [pages 2-231]
2. The only statistical analysis plan for efficacy [pages 232-246]
3. The original statistical analysis plan for safety, the updated final statistical analysis plan for safety, and a summary of changes (at the beginning of the updated final statistical analysis plan for safety) [pages 247-408]
4. Laboratory manual [pages 409-508]

CONFIDENTIAL

PROTOCOL

Protocol Title: A Phase 3 open-label trial assessing the safety and efficacy of bedaquiline plus PA-824 plus linezolid in Subjects with pulmonary infection of either extensively drug-resistant tuberculosis (XDR-TB) or treatment intolerant / non-responsive multi-drug resistant tuberculosis (MDR-TB).

Protocol Number: NiX-TB-(B-L-Pa)

Protocol Version: 1.0 (Final)

Protocol Date: 21 April 2014

PROTOCOL SIGNATURE PAGE

Protocol Title: A Phase 3 open-label trial assessing the safety and efficacy of bedaquiline plus PA-824 plus linezolid in Subjects with pulmonary infection of either extensively drug-resistant tuberculosis (XDR-TB) or treatment intolerant / non-responsive multi-drug resistant tuberculosis (MDR-TB).

Protocol Number: NiX-TB (B-L-Pa)

Protocol Version: 1.0

Protocol Date: 21APR2014

SPONSOR

I agree to the terms of this study protocol.

[signature]
Signature of Senior Medical Officer

Daniel Everitt MD
Printed Name

21 April 2014
Date

CO-ORDINATING INVESTIGATOR

I agree to the terms of this trial protocol. I will provide medical expertise and ensure consistency across sites for the trial and in accordance to the principles of Good Clinical Practice and local regulations.

Signature

Printed Name

Date

PROTOCOL SIGNATURE PAGE

Protocol Title: A Phase 3 open-label trial assessing the safety and efficacy of bedaquiline plus PA-824 plus linezolid in Subjects with pulmonary infection of either extensively drug-resistant tuberculosis (XDR-TB) or treatment intolerant / non-responsive multi-drug resistant tuberculosis (MDR-TB).

Protocol Number: NiX-TB (B-L-Pa)

Protocol Version: 1.0

Protocol Date: 21APR2014

SPONSOR

I agree to the terms of this study protocol.

Signature of Senior Medical Officer

Printed Name

Date

CO-ORDINATING INVESTIGATOR

I agree to the terms of this trial protocol. I will provide medical expertise and ensure consistency across sites for the trial and in accordance to the principles of Good Clinical Practice and local regulations.

[signature]

Signature

F.M. Conradie.

Printed Name

22 Apr 2014

Date

PRINCIPAL INVESTIGATOR PROTOCOL SIGNATURE PAGE

Protocol Title: A Phase 3 open-label trial assessing the safety and efficacy of bedaquiline plus PA-824 plus linezolid in Subjects with pulmonary infection of either extensively drug-resistant tuberculosis (XDR-TB) or treatment intolerant / non-responsive multi-drug resistant tuberculosis (MDR-TB).

Protocol Number: NiX-TB (B-L-Pa)

Protocol Version: 1.0

Protocol Date: 21APR2014

I hereby confirm that I have read the above protocol and agree to conduct this clinical trial as outlined in the above protocol. I will provide copies of the protocol and access to all the information required to conduct the clinical trial according to the above protocol to the site personnel under my supervision. I will discuss this material with them and ensure they are fully informed on all trial requirements.

______________________________ ______________________________
Signature Printed Name

____
Date

Development Phase:	2
Sponsor:	Global Alliance for TB Drug Development<br>40 Wall Street, 24th Floor<br>New York, NY 10005<br>United States of America
Medical Monitor:	Name: Christo van Niekerk<br>Address: Global Alliance for TB Drug Development<br>Boardwalk Office Park<br>Ground Floor, Block 1, Suite5<br>107 Haymeadow Street<br>Faerie Glen 0045<br>Pretoria, South Africa<br>Telephone: +27 87 700 3901<br>Mobile: +27 82 550 3865<br>Facsimile: +27 12 991 3045<br>Email: christo.vanniekerk@tballiance.org
Co-ordinating Investigator:	Name: Francesca Conradie<br>Address: CHRU Themba Lethu Clinic<br>Helen Joseph Hospital<br>No. 8 Perth Road<br>Westdene<br>Johannesburg<br>South Africa<br>Telephone: +27 11 531 4347<br>Mobile: +27 83 357 7611<br>Facsimile: +27 11 882 3912<br>Email: fconradie@witshealth.co.za

TABLE OF CONTENTS

1. PROTOCOL SYNOPSIS 14
1.1. Synopsis 14
1.2. Trial Flow Chart 18
2. INTRODUCTION 20
2.1. Background 20
2.2. Agents to be Studied 21
2.2.1. Bedaquiline 21
2.2.1.1. Bedaquiline Preclinical Studies 22
2.2.1.2. Bedaquiline Clinical Studies 23
2.2.1.3. Bedaquiline Clinical Safety 24
2.2.2. PA-824 27
2.2.2.1. PA-824 Preclinical Studies 27
2.2.2.2. PA-824 Clinical Studies 30
2.2.2.3. PA-824 Clinical Safety 35
2.2.3. Linezolid 39
2.3. Regimens to be Studied 42
2.3.1. Non-Clinical Studies 42
2.3.2. Clinical Study NC-001 44
2.3.3. Clinical Study NC-003 44
2.4. Overall Benefit/Risk Assessment 46
3. TRIAL RATIONALE AND OBJECTIVES 46
3.1. Trial Rationale 46
3.2. Dose Rationale 47
3.2.1. Bedaquiline 47
3.2.2. PA-824 47
3.2.3. Linezolid .... 47_Toc385314660
3.3. Trial Objectives 47
4. TRIAL DESIGN 47
4.1. Summary 47
4.2. Trial Endpoints 48
4.2.1. Primary Endpoint 48
4.2.2. Secondary Endpoints 49
4.2.2.1. Efficacy 49
4.2.2.2. Safety and Tolerability 49

4.2.2.3. Pharmacokinetics: ... 49
4.2.2.4. Exploratory ... 50
4.2.2.5. General Mycobacteriology ... 50
4.3. Trial Population ... 51
4.3.1. Inclusion Criteria ... 51
4.3.2. Exclusion Criteria ... 52
4.4. Treatment Plan: Schedule of Assessments ... 54
4.4.1. Screening (Up to 9 Days Prior to Treatment) ... 54
4.4.1.1. Screening ... 54
4.4.2. Treatment Period (Day 1 to Week 26 or Week 39) ... 55
4.4.2.1. Day 1 ... 55
4.4.2.2. Week 1 ... 55
4.4.2.3. Week 2 ... 55
4.4.2.4. Weeks 3, 5, 7, 9, 10, 11, 13, 14 and 15 ... 56
4.4.2.5. Week 4 ... 56
4.4.2.6. Week 6 ... 56
4.4.2.7. Week 8 ... 56
4.4.2.8. Week 12 ... 57
4.4.2.9 Week 16 (Week 30 when applicable) ... 57
4.4.2.10 Week 20 (Week 34 when applicable) ... 57
4.4.2.11 Week 26 (Week 39 when applicable) ... 58
4.4.2.12 Early Withdrawal ... 58
4.4.3 Follow-Up Period ... 59
4.4.3.1 1 Month Post-Treatment ... 59
4.4.3.2 2 Months Post-Treatment ... 59
4.4.3.3 3 Months Post-Treatment or Withdrawal ... 59
4.4.3.4 Months 6, 12 and 24 Post-Treatment ... 59
4.4.3.5 Months 9, 15, 18 and 21 Post-Treatment ... 60
4.4.3.6 Unscheduled Visits ... 60
4.5 Treatment Discontinuation and Subject Withdrawal ... 61
4.6 Temporary Dose Interruptions and Modifications ... 61
4.7 Stopping Rules ... 62
4.8 Subject Progress Definitions ... 62
4.8.1 Enrolment ... 62

4.8.2 Completed Trial ... 62
4.8.3 Withdrawn ... 62
4.9 Restrictions ... 63
4.9.1 Prior and Concomitant Medications and Other Treatments ... 63
4.9.1.1 Recommendations for Concomitant use of Anti-Malarials ... 63
4.9.1.2 Antiretroviral Therapy ... 63
4.9.1.3 Other Restrictions ... 64
5 INVESTIGATIONAL MEDICINAL PRODUCT ... 64
5.1 Trial Treatments ... 64
5.2 Method of Assigning Subjects to Study Treatment ... 64
5.3 IMP Administration ... 64
5.4 Subject Compliance ... 65
5.5 Blinding and Procedures for Breaking the Blind ... 65
5.6 IMP Packaging and Labelling ... 65
5.13.1 Packaging ... 65
5.13.2 Labelling ... 65
5.6 Storage ... 66
5.7 Dispensing and Accountability ... 66
5.8 Returns and Destruction ... 66
6 TRIAL VARIABLES AND PROCEDURES ... 66
6.1 Demographic and Background Variables and Procedures ... 66
6.2 Efficacy Variables and Procedures ... 67
6.3 Safety and Tolerability Variables and Procedures ... 68
6.4 Pharmacokinetic Variables and Procedures ... 69
6.5 Mycobacteriology Characterization Variables and Procedures ... 69
7 ADVERSE EVENTS ... 70
7.1 Definitions ... 70
7.1.1 Adverse Event (AE) ... 70
7.1.2 Serious Adverse Event (SAE) ... 70
7.1.3 Unlisted (Unexpected) Adverse Event ... 70
7.1.4 Life Threatening ... 70
7.1.5 Associated with the Use of the Drug ... 71
7.1.6 Attribution/Causality ... 71
7.1.7 Severity ... 71
7.1.8 Other AE Definitions ... 71

7.2 Reporting 72
7.2.1 Adverse Event (AE) 72
7.2.2 Serious Adverse Event (SAE) 72
7.2.3 Follow up of Adverse Events 73
7.2.4 Post-Trial Adverse Events 73
7.2.5 Clinical Laboratory Adverse Events 73
7.2.6 Disease under Study 73
7.2.7 Overdose 74
7.2.8 Drug Interaction 74
7.2.9 Pregnancy 74
7.3 Monitoring and Safety for Specific Toxicities 74
7.3.1 ALT, AST and Alkaline Phosphatase elevations: 75
7.3.2 Amylase elevation 75
7.3.3 Lipase Elevation 75
7.3.4 Musculo-skeletal System and Cardiac Muscle 75
7.3.5 Cardiac Rhythm Disturbances 75
7.3.6 Myelosuppression 76
7.3.7 Peripheral Neuropathy 76
7.3.8 Optic Neuropathy 76
7.3.9 Lactic Acidosis 76
7.3.10 Neurological 76
7.4 Safety Monitoring by the Data Safety Monitoring Committee 77
8 STATISTICAL ANALYSIS 77
8.1 Analysis Population 77
8.2 Sample Size 77
8.3 Interim Analyses 77
8.4 Primary Endpoint Analysis 78
8.5 Secondary Endpoint Analysis 78
8.5.1 Efficacy 78
8.6 Exploratory Endpoint Analysis 78
8.6.1 Efficacy 78
8.6.2 Safety and Tolerability Analysis 79
8.7 Pharmacokinetics: 80
8.8 Pharmacokinetics-Pharmacodynamics (PK-PD): 80
8.9 General Mycobacteriology 80
9 RECORDS MANAGEMENT 80

9.1 Data Collection ... 80
9.2 Source Documents ... 80
9.3 File Management at the Trial Centre ... 80
9.4 Records Retention at the Trial Centre ... 80
10 QUALITY CONTROL AND ASSURANCE ... 81
10.1 Site Procedures ... 81
10.2 Monitoring ... 81
10.3 Auditing ... 81
11 ETHICS AND REGULATORY ... 82
11.8 Basic Principles ... 82
11.2 Independent Ethics Committee/Institutional Review Board (IEC/IRB) Review ... 82
11.3 Regulatory Authorities ... 82
11.4 Informed Consent ... 82
11.5 Confidentiality ... 83
12 PUBLICATION POLICY ... 83
13 PROTOCOL AMENDMENT POLICY ... 83
14 FINANCIAL ASPECTS, INSURANCE AND INDEMNITY ... 83
15 REFERENCES ... 85

APPENDIX 1 THE IUATLD SCALE ... 87
APPENDIX 2 DIVISION OF MICROBIOLOGY AND INFECTIOUS DISEASES (DMID) ADULT TOXICITY TABLE ... 88
APPENDIX 3 VITAL SIGNS ... 97
APPENDIX 4 KARNOFSKY PERFORMANCE STATUS SCALE DEFINITIONS RATING (%) CRITERIA[19] ... 98
APPENDIX 5 EQ-5D-5L QUESTIONNAIRE ... 99
APPENDIX 6 BRIEF PERIPHERAL NEUROPATHY SCREENING ... 101
APPENDIX 7 TUBERCULOSIS SYMPTOM PROFILE (V3) ... 103
APPENDIX 8 LIVER TOXICITY MANAGEMENT GUIDELINES ... 104

TABLES

Table 1: Bedaquiline Adverse Drug Reactions ... 29
Table 2: PA-824 Animal Safety and Toxicology Findings ... 29
Table 3: PA-824 Phase 1 Clinical Studies ... 31
Table 4: PA-824 Phase 2 Studies ... 34
Table 5: NC-002 Elevations in Alanine Aminotransferase ... 38
Table 6: Murine Lung CFU counts during Treatment with Linezolid monotherapy versus Standard Therapy ... 39

Table 7: Murine Lung CFU counts during Treatment: Standard Therapy versus novel mono and combination therapies 43

Table 8: Murine Relapse Data, Standard Therapy versus novel combinations....43

Table 9: Summary Statistics for $EBA_{CFU(0-14)}$ Derived Using Bi-Linear Regression, Study NC-001....44

Table 10: NC-003 Efficacy Results: Daily $BAlogCFU_{(0-14)}$ 45

Table 11: NC-003 Safety Data 45

Table 12: General Mycobacteriology 50

Table 13: Adverse Events Attribution/Causality Ratings....71

Table 14: Adverse Event Severity Ratings 71

FIGURES

Figure 1: Trial Schematic 48

LIST OF ABBREVIATIONS AND DEFINITIONS OF TERMS

ADME	Absorption, Distribution, Metabolism, and Excretion
AE	Adverse Event
AIDS	Acquired Immune Deficiency Syndrome
ALP	Alkaline Phosphatase
ALT	Alanine Aminotransferase
AREDS2	Age Related Eye Disease Study 2
ART	Antiretroviral Therapy
AST	Aspartate Aminotransferase
AUC	Area under the plasma concentration time curve
$AUC_{(0-24)}$	Area under the plasma concentration time curve from zero to end of dosing interval
$AUC_{(0-t)}$	Area under the PK plasma concentration time (t) curve from zero to the last quantifiable PK plasma concentration prior to the subsequent dose, using the linear trapezoidal rule
BA	Bactericidal Activity
B	Bedaquiline (formerly J, TMC-207)
BID	Twice daily dosing
BMI	Body Mass Index
bpm	Beats per minute
BUN	Blood urea nitrogen
C	Clofazimine
°C	Degrees Celsius
CFU	Colony Forming Units
CK	Creatine Phosphokinase
CK-MB	Creatine Phosphokinase of Muscle Brain
C_{max}	Maximum observed plasma concentration
C_{min}	Minimum observed plasma concentration at the end of the dosing interval
CNS	Central Nervous System
CYP3A4	Cytochrome P450 3A4
DBP	Diastolic Blood Pressure
DDI	Drug-Drug Interactions
DMID	Division of Microbiology and Infectious Diseases
DNA	Deoxyribonucleic acid
DOTS	Directly Observed Treatment, Short Course
DS	Drug-Sensitive
DSMC	Data Safety Monitoring Committee
DST	Drug Sensitivity Testing
eCRF	Electronic Case Report Form
EBA	Early Bactericidal Activity
ECG	Electrocardiogram
ELISA	Enzyme-Linked Immunosorbent Assay
EMA	European Medicines Agency
ERPF	Effective Renal Plasma Flow
FDA	United States Food and Drug Administration
FF	Filtration Fraction

FSH	Follicle-Stimulating Hormone
GCP	Good Clinical Practice
GFR	Glomerular Filtration Rate
GGT	Gamma-glutamyltransferase
hERG	Human ether-à-go-go-related gene
HIV	Human Immunodeficiency Virus
hr	Hour
HRZE	isoniazid plus rifampicin plus pyrazinamide plus ethambutol
HRZM	Isoniazid plus rifampicin plus pyrazinamide plus moxifloxacin
IB	Investigator Brochure
IC_{50}	50% inhibitory concentration
ICF	Informed Consent Form
ICH	International Conference on Harmonization
IMP	Investigational Medicinal Product
IUATLD	International Union Against Tuberculosis and Lung Disease
i.v., IV	Intravenous
Kg	Kilogram
LDH	Lactate Dehydrogenase
L	Linezolid
LFT	Liver Function Test
lKr	Delayed rectifier potassium current
LH	Luteinizing Hormone
LSLV	Last Subject Last Visit
m	Meters
M	Moxifloxacin
MAOI	Monoamine Oxidase Inhibitor
MBD	Minimum Bactericidal Dose
M2	Bedaquiline metabolite M2
MDR	Multi Drug-Resistant
MED	Minimum Effective Dose
mg	Milligrams
mg/dl	milligram per decilitre
MGIT	Mycobacterial Growth Indicator Tube
MIC	Minimum inhibitory concentration
ml	Millilitre
mmHg	Millimeter of mercury
M. tb.	*Mycobacterium tuberculosis*
ms	Millisecond
NIH	National Institute of Health
NLME	Non-linear Mixed Effect
NOAEL	No Observed Adverse Effect Level
Pa	PA-824
PD	Pharmacodynamic
PE	Physical Examination

PK	Pharmacokinetic
pncA	A pyrazinamidase encoded in the genes of the *Mycobacterium* species.
PR	Electrocardiographic PR interval
q.d./QD	Once daily dosing
QRS	Electrocardiographic QRS interval
QT	Electrocardiographic QT interval
QTc	Corrected QT interval
QTcB	QT interval corrected by Bazett's method
QTcF	QT interval corrected by Fridericia's method
RR	Electrocardiographic RR interval
SAE	Serious Adverse Event
SAP	Statistical Analysis Plan
SBP	Systolic Blood Pressure
sc	Subcutaneous
SIRE	Streptomycin, Isoniazid, Rifampicin and Ethambutol
SSCC	Serial Sputum Colony Counts
T	Time
$t_{½}$	Apparent terminal elimination phase half-life
TB	Tuberculosis
TEAEs	Treatment-Emergent Adverse Events
TIW	Three times a week
T_{max}	Time at which C_{max} is observed
TMIC	Time over Minimum Inhibitory Concentrations
TTP	Time to Sputum Culture Positivity
UA	Uric Acid
ULN	Upper Limit of Normal
µg/ml	microgram per millilitre
WBC	White Blood Cell
WHO	World Health Organization
XDR	Extensively drug-resistant
Z	Pyrazinamide

1. PROTOCOL SYNOPSIS

1.1. Synopsis

**Name of Sponsor/Company:**	Global Alliance for TB Drug Development
**Name of Finished Products:**	bedaquiline tablets; PA-824 tablets; linezolid tablets.
**Protocol Title:**	A Phase 3 open-label trial assessing the safety and efficacy of bedaquiline plus PA-824 plus linezolid in Subjects with pulmonary infection of either extensively drug-resistant tuberculosis (XDR-TB) or treatment intolerant / non-responsive multi-drug resistant tuberculosis (MDR-TB)
**Treatment Indication:**	Pulmonary XDR-TB and treatment intolerant/non-responsive MDR-TB
**Trial Objective:**	To evaluate the efficacy, safety, tolerability and pharmacokinetics of bedaquiline plus PA-824 plus linezolid after 6 months of treatment (option for 9 months for subjects who remain culture positive at month 4) in Subjects with either pulmonary XDR tuberculosis, treatment intolerant or non-responsive multi-drug resistant tuberculosis (MDR-TB).
**Trial Design:**	An open-label clinical trial.<br><br>Treatment:<br>• bedaquiline 400 mg once daily for 2 weeks then 200mg 3 times per week plus PA-824 200mg once daily plus linezolid 600mg twice daily.<br><br>Treatment Duration:<br>• 6 months<br>• If subjects are still culture positive at month 4, option to extend treatment to 9 months or withdraw.<br><br>Follow-Up:<br>• Subjects who complete treatment will return for follow-up visits 1 and 2 months after end of treatment and every 3 months for 24 months after end of treatment.<br>• Subjects who withdraw after ≤14 days of IMP administration are to return for an Early Withdrawal visit only;<br>Subjects who withdraw after ≥15 days of IMP are to return for the Early Withdrawal, and for the 3, 12 and 24 month follow up visits after their last dose of IMP.<br><br>Data Saftey Monitoring Committee (DSMC) Reviews:<br>Interim Safety/Efficacy data will be reviewed by DSMC as follows:<br>• After the first 10 subjects are in the study for 6 months after assignment of study treatment;<br>• After the next 10 subjects are in the study for 6 months after assignment of study treatment<br>• Every 6 months after the second group of 10 subjects are reviewed;<br>• Ad hoc meetings can be called by Sponsor/DSMC based on rates of SAEs or to review results of futility analysis or if safety concerns arise during the trial.
**Patient Population:**	A total of up to 200 male or female Subjects aged 14 and over with confirmed sputum culture-positive pulmonary XDR-TB or MDR-TB with a documented intolerability or nonresponse to treatment.

**Name of Sponsor/Company:**	Global Alliance for TB Drug Development
**Name of Finished Products:**	bedaquiline tablets; PA-824 tablets; linezolid tablets.
**Test Product, Dose and Mode of Administration:**	The Investigational Medicinal Product (IMP) will be supplied as:<br>• Bedaquiline 100mg tablets<br>• PA-824 200mg tablets<br>• Scored Linezolid 600 mg tablets<br><br>The assigned treatment regimen will be administered orally for 6 months (possibly 9 months) at the following doses and intervals:<br>• Bedaquiline 400 mg once daily for 2 weeks then 200 mg 3 times per week; plus PA-824 200mg once daily; plus linezolid 600mg twice daily.<br><br>A reduction in the dose of linezolid (to either 600 mg qd, 300 mg bid or 300 mg qd) or temporary cessation of linezolid (due to a linezolid-specific toxicity), or of the full regimen per Investigator discretion will be allowed for suspected drug related toxicity. Re-introduction of linezolid (at the same or at a lower dose) or the full regimen could be considered post a cessation not greater than 35 consecutive days.

Criteria for Evaluation:

Primary Endpoint:

Incidence of bacteriologic failure or relapse or clinical failure through follow up until 24 months after the end of treatment.

Abbreviated Definitions (full definitions will be described in the Statistical Analysis Plan (SAP)):

- Bacteriologic failure: During the treatment period, failure to attain culture conversion to negative.
- Bacteriologic relapse: During the follow-up period, failure to maintain culture conversion to negative status in culture, with culture conversion to positive status with a Mycobacterium tuberculosis (*M.tb.*) strain that is genetically identical to the infecting strain at baseline.
- Clinical failure: A change from protocol-specified TB treatment due to treatment failure, retreatment for TB during follow up, or TB-related death.

Note:

- Culture conversion requires at least 2 consecutive culture negative/positive samples at least 21 days apart.
- Subjects who are documented at a visit as unable to produce sputum and who are clinically considered to be responding well to treatment will be considered to be culture negative at that visit.

Secondary Endpoints:

- Time to sputum culture conversion to negative status through the treatment period.
- If liquid culture in the MGIT platform is used, the rate of change in time to sputum culture positivity (TTP) over time in the Mycobacterial Growth Indicator Tube (MGIT) system in sputum, represented by the model-fitted log(TTP) results as calculated by the regression of the observed log(TTP) results over time
- Proportion of subjects with sputum culture conversion to negative status at 4, 6, 8, 12, 16 and 26 or 39 weeks.
- Linezolid dosing (actual) and efficacy will be explored.
- Change from baseline TB symptoms.
- Change from baseline in Patient Reported Health Status.

Safety and Tolerability:

- All cause mortality.
- Incidence of Treatment Emergent Adverse Events (TEAEs) will be presented by severity (DMID Toxicity Grade), drug relatedness and seriousness, leading to early withdrawal and leading to death.
- Quantitative and qualitative clinical laboratory result measurements, including observed and change from baseline.
- Quantitative and qualitative measurement of ECG results, including observed and change from baseline.
- Descriptive statistics of ophthalmology slit lamp examination data (age related eye disease study 2 [AREDS2]

Name of Sponsor/Company:	Global Alliance for TB Drug Development
Name of Finished Products:	bedaquiline tablets; PA-824 tablets; linezolid tablets.

lens opacity classification and grading). Categorical data for lens opacity will be summarized in a frequency table for the right and left eye, respectively, including change from baseline.

- Changes in ophthalmic exam for visual acuity and color vision, including observed and change from baseline.
- Changes noted in peripheral neuropathy signs and symptoms, including observed and change from baseline.

These data will be presented as descriptive analyses, and no inferential tests will be carried out.

Pharmakokinetics (PK):

Pharmacokinetics will consist of two separate schedules:

- All Subjects- Pre-dose sampling at weeks 2, 8 and 16 to measure C_{trough} levels of bedaquiline, bedaquiline metabolite M2, linezolid and PA-824.
- PK Sub-study Subjects- in addition to the C_{trough} samples, there will be intensive PK sampling at week 16 at pre-dose, 0.5, 1, 2, 4, 8 12, 12.5, 13, 14, 16, 20 and 24 hours after dosing in a sub-group of 30 Subjects across selected sites.

For the PK sub-study samples, the following PK parameters will be estimated from the individual (per Subject) PK plasma concentrations: Minimum observed PK plasma concentration (C_{min}), maximum observed PK plasma concentration(C_{max}), time to reach C_{max} obtained without interpolation (T_{max}), area under the PK plasma concentration time (t) curve from zero to the last quantifiable PK plasma concentration prior to the subsequent dose, using the linear trapezoidal rule ($AUC_{(0-t)}$), area under the PK plasma concentration time (t) curve from zero to 24 hours ($AUC_{(0-24)}$). Oral apparent clearance (CL/F) by non-compartment model. These will be derived for each analyte. In addition, for linezolid analyte BID dose, the AUC_{0-12}, C_{max}, C_{min}, CL/F and $t_{1/2}$ will be calculated based on dose interval 0-12 hrs.

Exploratory:

- Evaluate whether any of the secondary endpoints predicts relapse free cure.
- Sub-analysis of populations by HIV status and CD4 count.
- Correlation of Time over mitochondrial protein synthesis inhibition (MPS50) with linezolid toxicity (The MPS50 will be an assumed value from the literature).

Mycobacteriology Characterization:

M.tb. isolates will be processed at a central laboratory for:

- Speciation of the infecting organism by molecular or antigen based test to confirm *M.tb.*
- MIC of bedaquiline, PA-824 and linezolid;
- Drug Susceptibility Testing in liquid culture for rifampicin, isoniazid, streptomycin, ethambutol, and second line TB drugs including fluoroquinolones and injectables;
- Extraction of bacterial (*M.tb*) DNA for molecular strain typing;
- DNA for pncA sequencing.

Statistical Methods:

The primary efficacy endpoint is treatment failure, defined as bacteriologic failure, or relapse, or clinical failure through follow-up until 24 months after the end of treatment. The probability of treatment failure through follow-up until 24 months after the end of treatment, as a function of time after assignment of study treatment, will be analyzed using Kaplan-Meier analysis. The binomial proportion for subjects with bacteriologic failure will be presented. No multiplicity adjustments for alpha will be done as this is an exploratory trial.

Futility Analysis:

Interim analyses for futility will be performed for every 20 patients who reach the primary efficacy endpoint, treatment failure (that is, bacteriologic failure, or relapse, or clinical failure).

The study will be stopped for futility should the simultaneous upper confidence band for the "survival function" (probability, as a function of time after assignment of study treatment, of **not** experiencing treatment failure) fall below 0.4 at any time point after the assignment of study treatment. Equivalently, the study will be stopped for futility if the probability of treatment failure at any time point after assignment to study treatment is statistically significantly higher than 0.6 or 60%.

**Name of Sponsor/Company:**	Global Alliance for TB Drug Development
**Name of Finished Products:**	bedaquiline tablets; PA-824 tablets; linezolid tablets.
Once all patients have been recruited or have completed the treatment period, no further futility analyses will be performed.	
**Trial Duration:**<br>Estimated date of first Subject enrolled: Quarter 4 2014<br>Estimated date of last Subject enrolled: Quarter 1 2018<br>Estimated date of last Subject completed: Quarter 1 2021<br><br>Duration of study:<br>~6 Years (An enrolment period of at least 42 months plus 9 days pre-treatment plus 6-9 month treatment period plus 24 months post treatment follow-up).	

1.2. Trial Flow Chart

Period	Screening[a]	Treatment																			9 Month Treatment ONLY[p]				Post Treatment Follow-up Period[b]									
Time of Visit	Up to 9 days prior to Treatment	Day 1[c]	Week 1	Week 2	Week 3	Week 4	Week 5	Week 6	Week 7	Week 8	Week 9	Week 10	Week 11	Week 12	Week 13	Week 14	Week 15	Week 16	Week 20	Week 26	Week 30	Week 34	Week 39	Early Withdrawal (Treatment)	1 month	2 month	3 month	6 month	9 month	12 month	15 month	18 month	21 month	24 month
Visit Window	N/A	+/- 3 days												+/- 7 days										N/A	+/- 2 weeks									
Informed Consent	X																																	
Demography	X																																	
Medical/Treatment History	X																																	
Inclusion/Exclusion	X	X																																
Karnofsky Assessment	X																																	
HIV Status[d]	X																																	
CD4 Count[e]	X																																	
Chest X-Ray[f]	X																																	
Serum or Urine Pregnancy Test[g]	X	X								X										X[h]			X[h]	X										
TB Symptoms Profile	X									X										X[h]			X[h]	X						X				X
Patient Reported Health Status	X									X										X[h]			X[h]	X						X				X
Slit Lamp Exam[i]	X																			X[h]			X[h]	X			X							
Ophthalmic Exam[j]	X					X				X				X				X	X	X	X	X	X	X			X	X		X				X
Vital Signs	X	X	X	X		X		X		X				X				X	X	X	X	X	X	X			X	X	X	X	X	X	X	X
Single 12-LeadECG	X	X	X			X				X								X		X[h]	X		X[h]	X										
Limited Physical Exam[k]			X	X		X		X		X				X				X	X		X	X					X	X	X	X	X	X	X	X
Full Physical Exam[k]	X	X																		X[h]			X[h]	X										
Laboratory Safety Tests[l]	X	X	X	X		X		X		X				X				X	X	X	X	X	X	X										
Con Meds	X	X	X	X		X		X		X				X				X	X	X	X	X	X	X			X	X	X	X	X	X	X	X
Adverse Events	X	X	X	X		X		X		X				X				X	X	X	X	X	X	X			X	X	X	X	X	X	X	X
Study Medication/Compliance[m]		X	X	X		X		X		X				X				X	X	X	X	X	X	X										
PK Sampling[n]				X						X								X																
Early Morning & Spot Sputum[o]	X	X	X	X		X		X		X				X				X	X	X	X	X	X	X	X	X	X	X	X	X	X	X	X	X
Complete/Full Blood Count					X		X		X		X	X	X		X	X	X																	
Peripheral Neuropathy	X					X				X				X				X	X	X	X	X	X	X			X	X		X				X

Note:

- Unscheduled visits should be planned to assess, confirm, and follow up on clinically relevant AEs or laboratory abnormalities.
- All assessments are to be performed pre-dosing unless otherwise specified.
- On days where the following assessments are done the order should be: ECG before vital signs, blood draws (for Safety or PK).

a. **Screening:** Screening assessments can occur on different days within nine days prior to treatment.
b. **Follow-up Visits for Early Withdrawal Subjects:** Once a Subject has been permanently withdrawn from the trial, they will be required to attend Early Withdrawal, Month 3, Month 12 (if not already performed) and Month 24 follow-up visits. The Month 12 and 24 visits will be to collect Serious Adverse Event (SAE) information (including verification of survival) and patient reported TB outcome information only and may be telephonic, a home or a site visit.
c. **Day 1 (baseline):** All procedures are to be completed prior to dosing.
d. **HIV testing:** if an ELISA and/or Western Blot based HIV test was performed within 1 month prior to trial start, it should not be repeated as long as documentation of testing method and reults can be provided.
e. **CD4 count:** For HIV-positive Subjects.
f. **Chest X-Ray:** Chest X-Ray at Screening or within 1 year prior to Screening. The Investigator is responsible for its review and analyisis for subject inclusion.
g. **Serum or Urine Pregnancy:** Women of child-bearing potential only, whether they are sexually active or not.
h. **Final Treatment Visit:** Serum or Urine Pregnancy Test, TB Symptoms Profile, Full Physical Examination, 12-lead ECG, Patient Reported Health Status and Slit Lamp Eaxamination are only performed at the subjects' applicable End of Treatment Visit dependent on their treatment duration and not on the other indicated End of Treatment Visit. If Week 26 is not the Final Treatment Visit, only a limited physical exam should be done.
i. **Slit-Lamp Exam:** Slit Lamp examination will be performed by an Ophthalmologist with AREDS2 training. See section 4.4.2.12 for details on what follow-up slit lamp exams are necessary for subjets who withdrawal early.
j. **Ophthalmic Exam:** to include Ophthalmologic Medical history at Screening; All exams to include Visual Acuity and Color Vision assessment. Can be done by any trained study staff throughout study. Screening exam must be done by Ophthalmologist in addition to trained study staff that will perform exams throughout the study.
k. **Physical Exam: Full Physical Exams** to include gross neurological exam. All other PEs should be **limited** to weight and a pulmonary, cardiovascular and abdominal exam.
l. **Safety Laboratory Assessments** (refer to section 6.3 for details of laboratory safety assessments)
m. **Study Medication/Compliance:** Study medication administration will be supervised per local site practice to assure compliance to regimen.
n. **PK Sampling:** Pharmacokinetics will consist of two separate schedules:
    - **All Subjects** - Pre-dose C_{trough} sampling at weeks 2, 8 and 16, must be taken within 1 hour before the next scheduled dose.
    - **PK Sub-study Subjects** - in addition to the C_{trough} samples, there will be intensive PK sampling at week 16 at pre-dose, 0.5, 1, 2, 4, 8 12, 12.5, 13, 14, 16, 20 and 24 hours after dosing in a sub-group of 30 Subjects across selected sites. To be collected at the specified time points within the allowed applicable window periods: Pre-dose: 0-5 minutes before dose; 0.5- 1 hours post-dose: +/- 5 minutes; 2-18 hours post dose: +/- 5 minutes; 12 hours post-dose: +/- 5 minutes and prior to next dose for BID treatment arm, 12.5 – 13 hours +/- 5 minutes and 14- 24 hours +/10 minutes and prior to next dose.
o. **Sputum Sampling:**
    1. Screening (Day -9 to -1): A single spot sputum will be collected at the research site under the coaching and observation of the trial staff. The following analysis will be performed on this sample:
        - Direct microscopy for acid-fast bacilli (AFB);
        - Gene Xpert, Hain Assay MTBDRplus or an alternative molecular or antigen-based test to confirm M.tb;
    2. All visits from Day 1 (baseline) up to and including Month 24: Two sputum samples, one early morning brought from home or in the hospital if hospitalized, and one spot at the research site under the coaching and observation of the trial staff (or if hospitalized, in the morning at least 1 hour after the early morning sample) will be collected. If sputum samples obtained at Month 4, End of Treatment (Week 26/39) or end of follow-up Month 24 are contaminated, the Subject should return for an unscheduled visit(s) to give additional samples or to document the Subject is not able to produce sputum. The following analyses will be performed on sputum samples:
        - Culture for presence or absence of *M.tb.*;
        - If MGIT is performed, TTP in liquid medium.

    Mycobacteriology Characterisation Tests, Performed on:
    1. Day 1 (baseline) spot sputum samples (or Screening to Week 4 if the baseline is contaminated or negative);
    2. Confirmed Positive Cultures at or after end of treatment.

    The *M.tb.* isolates will be processed at a central lab for:
    - MIC against bedaquiline, PA-824 and linezolid;
    - Drug Susceptibility Testing in liquid culture for rifampicin, isoniazid, streptomycin, ethambutol and second-line TB drugs such as fluoroquinolones, and injectables;
    - Speciation of the infecting organisms by molecular or antigen based test;
    - Extraction of bacterial (*M.tb.*) DNA for molecular strain typing;
    - DNA extraction for pncA testing (baseline only).

All Day 1 (baseline) *M.tb.* isolates and isolates from positive cultures to be stored at the local microbiology laboratory (or until requested to transfer to a central laboratory for testing), until trial closure for the applicable study tests. The extracted *M.tb.* DNA and isolates will be stored for potential further work to validate new assay tools for a maximum of 5 years after trial closure.

p. **Visit Schedule:** Subjects still culture positive at month 4, will be withdrawn, or will receive a total of 9 months of treatment. *(Week 30, 34 and 39 visits should not be done for Subjects who complete study treatment in 6 months)*

2. INTRODUCTION

2.1. Background

Although some progress has been made in recent years in controlling TB globally, TB has remained a persistent problem in many countries. TB is currently one of the top three fatal infectious diseases, it is the leading cause of death among HIV-infected individuals, and there is more TB in the world today than at any other time in history. The current first-line anti-tuberculosis agents have been in use for over 20 years and although the current regimens and drugs have been very successful in controlled clinical trials resulting in the permanent cure of more than 95% of trial Subjects, treatment takes 6 months to complete. This, plus side effects, result in poor compliance which is particularly likely to occur after the second month of treatment. As a result of poor treatment compliance, drug resistance is becoming more common and fears of an epidemic with virtually untreatable strains of TB – extensively drug resistant TB (XDR-TB) - are growing. Novel drugs for tuberculosis are needed for the growing numbers of patients with untreatable strains.

WHO is tracking the increase of drug resistant strains. They estimate that there are about 650,000 MDR-TB cases in the world at any one time. On average, an estimated 9.6% of MDR-TB cases have XDR-TB, defined as resistance to at least isoniazid and rifampicin, as well as to any fluoroquinolone, and to any of the three second-line injectables (amikacin, capreomycin, and kanamycin)(20). XDR-TB has been reported by 92 countries. Among a subset of 795 XDR-TB patients in 26 countries, treatment success was 20% overall and 44% of patients died; South Africa reports the most XDR-TB cases(22). In 2006 a report from rural South Africa noted that 52 patients died of 53 patients identified with XDR-TB and HIV co-infection; the mean survival was 16 days from the time of diagnosis(21). A recent report from South Africa documented the very grave long term prognosis of patients with XDR-TB(23). Between 2008 and 2012 107 patients with XDR-TB from 3 provinces in South Africa were followed for long term outcomes. At 60 months of follow up 12 patients (11%) had a favourable outcome, 78 (73%) had died, four (4%) had defaulted, and 11 (10%) had failed treatment. With such dire outcomes for patients with XDR-TB, novel drug combinations are needed to improve treatment outcomes. Recently, linezolid was identified as a potentially efficacious drug to use with patients with XDR-TB when added to a failing regimen(24).

Following the declaration of TB as a global emergency by the World Health Organization (WHO) in 1993, there has been a resurgence of efforts to develop improved TB therapies and several promising new agents are presently in or approaching clinical evaluation. On December 28, 2012 the U.S. Food and Drug Administration approved bedaquiline (Sirturo™) as part of combination therapy to treat adults with multi drug-resistant pulmonary tuberculosis (MDR-TB) when other alternatives are not available. On December 20, 2013 the CHMP recommended approval of bedaquiline by the EMA as part of an appropriate combination regimen for pulmonary multi-drug resistant tuberculosis (MDR-TB) when opther treatments are not available. In November of 2013, the CHMP recommended that delamanid be approved by the EMA for treatment of pulmonary MDR-TB in combination with the WHO optimized background regimen. Although both of these regulatory actions are positive steps, more work needs to be done to develop new regimens for both drug-sensitive (DS-TB) and drug resistant TB (MDR-TB/XDR-TB). New combination regimens are desperately needed for two reasons; to shorten treatment to a duration more easily manageable by patients and public health services for DS-TB and to provide more efficacious, safer, better tolerated and affordable treatment for the growing number of patients suffering from MDR-TB and XDR-TB.

The NiX-TB study offers a new opportunity to treat patients with XDR-TB with three drugs for which there is no expected pre-existing resistance. Bedaquiline, PA-824 and Linezolid are active against many drug resistant strains of *M.tb.* and substantial preclinical and clinical data are available to demonstrate their potential for individual and combined microbicidal and sterilizing activity in TB disease. To further development of novel regimens, TB Alliance is currently conducting a series of Phase 2 and potential Phase 3 studies to evaluate building blocks of this regimen. A recently completed 14 day Early Bactericidal Activity

(EBA) study in South Africa (Study NC-003) demonstrated bactericidal activity of the bedaquiline-PA-824-pyrazinamide regimen. This regimen is now being taken into an "SSCC" 8 week trial designed to investigate the combination of bedaquiline, PA-824 and pyrazinamide (B-Pa-Z) in DS-TB and bedaquiline, moxifloxacin, PA-824 and pyrazinamide in MDR-TB. While this study is underway, additional Phase 2 studies are being planned to evaluate this combination with an oxazolidanone (the drug class of linezolid).

The current study (NiX-TB) is an open label trial designed to investigate the combination of bedaquiline, linezolid, and PA-824 in XDR-TB. Since patients with XDR-TB are failing nearly all drugs, there is no useful standard of care (SOC) to use for comparison. Because the long term outcome of patients in South Africa treated with available SOC is up to 75% mortality[(23)], this open label study offers the opportunity to identify a substantially better outcome without the need for a comparison group. This study will provide patients with an oral regimen that contains three novel drugs with the potential for a shorter treatment, better outcomes and with fewer side effects than the drugs currently being used.

The information presented below first details the key preclinical information and human efficacy and safety information for each of the drugs in the regimen and then present preclinical and clinical data to support the combination of these drugs in a regimen to treat patients with XDR-TB.

2.2. Agents to be Studied

2.2.1. Bedaquiline

Bedaquiline (TMC207; Sirturo™ package insert[(3)]) is a new agent being developed for TB treatment. As detailed in the Investigator's Brochure[(4,5)] bedaquiline is a diarylquinoline that offers a novel mechanism of anti-tuberculosis action by specifically inhibiting mycobacterial adenosine triphosphate (ATP) synthase[(6)]. *In vitro*, bedaquiline potently inhibits both drug-sensitive and drug-resistant *M. Tb* isolates[(7,8)], and is also bactericidal against non-replicating *M. tb.*[(9)]. In the murine model of TB, bedaquiline was as active as the triple combination of isoniazid (H), rifampicin (R), and pyrazinamide (Z). Addition of bedaquiline to HRZ results in accelerated clearance of *M. tb.*[(1, 2)]. There appears to be a synergistic interaction with pyrazinamide: 100% of mice were culture negative after 8 weeks of treatment with bedaquiline and pyrazinamide compared to 0% of mice treated with the standard regimen of rifampicin, isoniazid and pyrazinamide[(10)]. Collectively, these findings in the mouse model have led to the suggestion that regimens containing bedaquiline and pyrazinamide could be effective in the treatment of both drug sensitive and drug resistant TB and shorten treatment duration in patients. While the combination of bedaquiline and PA-824 in the murine model of TB appeared somewhat antagonistic relative to bedaquiline alone, it was as active as the triple combination of HRZ[(10)] and in a subsequent study it was more active in the mouse model than HRZ[(11)]. Thus a novel regimen with a bedaquiline plus PA-824 core could be effective in the treatment of MDR-TB/XDR-TB by providing two novel drugs for which there is no known pre-existing resistance.

To date, bedaquiline has been studied as monotherapy in two dose-ranging EBA trials (C202 and TMC207-CL001)[(12,13)], in two combination EBA trials (NC-001[(14)] and NC-003) and in 2 Phase 2b studies (C208 and C209). In the monotherapy studies, bedaquiline was dosed over a range of 100-400 mg/day. Subjects with TB had approximately a 1 log decrease in logCFU over 14 days at all doses studied. The first 14 day EBA combination study (NC-001) demonstrated that bedaquiline in combination with pyrazinamide (B-Z) and bedaquiline in combination with PA-824 (B-Pa) had positive EBA activity. The second 14 day EBA combination study (NC-003), currently undergoing analysis, included a number of bedaquiline-containing arms: bedaquiline, pyrazinamide and clofazimine (B-Z-C), bedaquiline, PA-824 and clofazimine (B-Pa-C); bedaquiline, PA-824 and pyrazinamide (B-Pa-Z) and bedaquiline, PA-824, pyrazinamide and clofazimine (B-Pa-Z-C). Among these, B-Pa-Z had the best activity which was at least as good as the HRZE control.

The 2 completed Phase 2b studies form the pivotal studies reviewed by the FDA for accelerated approval of bedaquiline (Sirturo ™). Together, these clinical studies provide justification for proceeding to the current study and are described briefly below and in greater detail in the IB.

2.2.1.1. Bedaquiline Preclinical Studies

Full details of the preclinical studies are provided in the current bedaquiline Investigator's Brochure[4, 5] and Sirturo TM label[3]. *In vitro* studies have demonstrated that the range of minimum inhibitory concentrations (MICs) for *M. tb.* H37Rv, the international reference strain, and 6 fully drug-susceptible clinical isolates, was 0.030 to 0.120 µg/ml. The activity of bedaquiline appears to be specific for mycobacteria, as the MICs for non-mycobacteria were at least 500-fold higher. The activity of the main metabolite of bedaquiline, M2, was determined against *M. tb.* H37Rv in both solid and liquid media and its MIC was found to be 0.1 µg/ml. This MIC shows that M2 is active against *M. tb.* but 3-6 times less active than the parent compound bedaquiline. Bedaquiline demonstrated similar *in vitro* efficacy against *M. tb.* clinical isolates resistant to the known anti-TB drugs (isoniazid, rifampicin, pyrazinamide, streptomycin, ethambutol, or fluoroquinolones). As expected, from the lack of cross-resistance with currently used anti-tuberculosis agents, bedaquiline retained activity against MDR-TB isolates.

The non-clinical safety evaluation of bedaquiline includes pharmacology, pharmacokinetics, toxicology and metabolism studies that were conducted in accordance with current ICH guidelines. Repeated dose toxicity studies were performed with dosing durations up to 3 months in mice and up to 6 months in rats and in dogs. Recovery was studied in rats and dogs. In repeated dose toxicity studies up to 3 months in mice, up to 6 months in the rat and up to 9 months in dogs, bedaquiline was associated with target organ changes in the mononuclear phagocytic system (indicative of phospholipidosis), stomach, liver, pancreas, and muscle. Toxicity was often associated with an increased presence of neutrophils in some tissues such as the female genital tract and this was preceded by a peripheral neutrophilia. For more detailed information please refer to the bedaquiline IB[4,5].

Respiratory parameters in rats were unaffected by treatment. There were no effects suggestive of neurological impairment or delayed neurotoxicity in rats. In single dose toxicity studies there were no mortalities following oral doses of up to 200 mg/kg in mice and rats. No mutagenicity or clastogenic effects were seen in a series of *in vitro* and *in vivo* genotoxicity tests. Bedaquiline was evaluated for possible developmental toxicity effects in the rat and the rabbit. No teratogenic effects were found. *In vitro*, bedaquiline slightly to moderately inhibited the delayed rectifier potassium current (IKr) in the human ether-à-go-go-related gene (hERG) model. Bedaquiline and M2 had no notable effects on IKr at 0.01 µM (0.006 and 0.005 µg/mL, respectively). However, at higher concentrations (0.03 to 3 µM), both compounds had a slight to strong concentration-dependent effect with a 50% inhibitory concentration (IC_{50}) of 0.37 µM (0.2 µg/mL) for bedaquiline and up to 0.45 µM (0.24 µg/mL) for M2. However, this effect did not manifest as a prolongation of repolarization in subsequent *in vivo* studies. There were no relevant effects on the isolated right atrium of guinea pigs *in vitro*, or in the isolated Langendorff-perfused rabbit heart. *In vivo*, positive chronotropic effects were seen in the anesthetized guinea pig after i.v. administration, but not in the conscious dog. In conscious, telemetered dogs, oral bedaquiline had no relevant effects on cardio-hemodynamic and electrocardiogram (ECG) parameters.

Prior to the use of PA-824 in combination with bedaquiline in clinical study NC-001, a preclinical cardiovascular safety pharmacology study was conducted in unrestrained beagle dogs with both drugs to explore the potential for additive effects on QT prolongation induced by the combination. Results indicate that administration of 100 mg/kg bedaquiline daily, for 7 days, causes a small increase in QTc interval by Day 6 in some animals that is not influenced by the addition of 100 mg/kg PA-824 on Day 7 [15].The effect of PA-824 dosing alone on QT interval appeared to be due to discomfort related to the SC route of administration and not related to the plasma exposure (please see the PA-824 Investigators Brochure for more detail).

2.2.1.2. Bedaquiline Clinical Studies

In the clinical studies conducted to date, a total of approximately 645 Subjects (including 265 healthy volunteers) have been exposed to bedaquiline in the Phase 1 and 2 clinical trials conducted as a part of the development program for the treatment of MDR-TB. An additional 45 Subjects received bedaquiline, either as monotherapy (B) or in combination with other agents (B-Pa or B-Z) in study NC-001, and 45 more in study NC-003 (B-Pa-Z, B-Pa-C, B-Pa-Z-C). Four short-term Phase 2a trials enrolled treatment-naïve Subjects (C202,TMC207-CL001, NC-001 and NC-003). One long-term, open-label, Phase 2 trial, in MDR-TB Subjects (bedaquiline-TiDP13-C209) and one long-term, Phase 2b trial, consisting of 2 different stages in Subjects infected with newly diagnosed sputum smear-positive pulmonary MDR-TB (bedaquiline-TiDP13-C208), have been completed. The principal findings of these trials are summarized below. Full details of the completed clinical studies are provided in the current bedaquiline IB[4,5] and Sirturo ™ label[3].

The Phase 1 trials have provided a basic understanding of bedaquiline's pharmacokinetic characteristics, drug-drug interaction potential, and short-term safety/tolerability profile in healthy Subjects. Bedaquiline was well absorbed with time to reach the maximum plasma concentration at approximately 5 hours post-dose. The maximum plasma concentration and AUC increased proportionally up to the highest doses studied (up to 700 mg in a single dose-ranging study, 800 mg single dose in study bedaquiline-TBC1003 and 400 mg q.d. multiple doses). Accumulation from Days 1 to 14 was approximately 2-fold expressed as increase in AUC, while trough concentrations increased up to 3.5-fold. The pharmacokinetics of bedaquiline were comparable between healthy Subjects and Subjects with pulmonary TB. The apparently close to steady-state concentrations in plasma after 14 days of daily treatment is ascribed to the important amount of the drug that is eliminated from the circulation during the ἀ and β phases of the plasma concentration-time curve. The average terminal elimination half-life of bedaquiline and metabolite M2 noted on extended follow-up after repeat dosing of Subjects with TB infection is about 5.5 months.

Administration of bedaquiline as the tablet formulation with food, increased the relative bioavailability (by 95%) compared to administration without food, and drug-drug interaction trials confirmed the role of cytochrome P450 3A4 (CYP3A4) in the metabolism of bedaquiline to M2. A recently completed study (DMID 10-0043) demonstrated that when given in combination, rifampicin, and to a lesser degree rifabutin, decreased exposure to bedaquiline presumptively due to induction of P450 enzymes. The clinical significance of these findings is unknown, however the current study (NC-005) will not permit the concomitant use of bedaquiline with any rifamycin.

The efficacy of bedaquiline was initially demonstrated in two monotherapy EBA studies C202 and TMC207-CL001. Study C202 was a 7 day study of three daily doses of bedaquiline (25, 100 and 400mg) in treatment-naïve Subjects with MDR-TB. In this study, the 400mg dose group demonstrated positive EBA and was numerically superior to the 25 and 100mg doses. In study TMC207-CL001, doses of 100, 200, 300 and 400mg daily (following a 2-day loading dose) were studied in Subjects with newly-diagnosed pulmonary TB for 14 days. There were no statistically significant differences between the treatment groups, although there was a numerical trend suggesting a positive dose-response relationship. Taken together, these studies establish that bedaquiline monotherapy has EBA in Subjects with TB and that higher doses appear to have greater efficacy.

A 14 day EBA regimen study (TB Alliance Study NC-001-(B-M-Pa-Z)) evaluated bedaquiline administered as monotherapy at 400 mg/d or in combination at that dose with either PA-824 administered at 200 mg/d or weight-adjusted pyrazinamide, to Subjects with pulmonary TB at 2 clinical sites in South Africa. The results indicate that over 14 days, the mean logCFU decreased by 1.3 from baseline in the 15 Subjects given bedaquiline 400 mg/d after a 2 day loading dose. In the cohort of 15 Subjects given bedaquiline 400 mg/d after a loading dose plus weight-adjusted daily doses of pyrazinamide (Z), the mean logCFU decreased by 2.0 logs from baseline, indicating that Z potentiates the early bactericidal effect of bedaquiline. In the cohort

with 15 Subjects given bedaquiline 400 mg/d after a loading dose plus 200 mg/d PA-824, the mean logCFU decreased by 1.9. While it appeared that the addition of PA-824 potentiated the anti-tuberculosis activity of bedaquiline, the mean logCFU decrease of the combination was similar to that of PA-824, administered alone at the 200 mg/d dose in the two prior EBA studies of PA-824 monotherapy.

The second 14 day EBA combination study (NC-003) included a number of bedaquiline-containing arms: bedaquiline, pyrazinamide and clofazimine (B-Z-C), bedaquiline, PA-824 and clofazimine (B-Pa-C), bedaquiline, PA-824, and pyrazinamide (B-Pa-Z) and bedaquiline, PA-824, pyrazinamide and clofazimine (B-Pa-Z-C). Among these, B-Pa-Z had the best activity which was at least as good as the HRZE control (Daily Log CFU – 0.167 vs 0.151, respectively).

The long-term efficacy of bedaquiline in Subjects with MDR-TB has been studied in a placebo-controlled, randomized Phase 2b trial (C208) and an open-label, uncontrolled, Phase 2b trial (C209). In the Phase 2b placebo-controlled trial, C208, the addition of bedaquiline to a 5-drug MDR-TB regimen resulted in significantly faster time to culture conversion compared to placebo. During the 8-week treatment in Stage 1, 47.6% of Subjects in the bedaquiline group became MGIT culture negative compared to 8.7% of Subjects in the placebo group. At Week 24 in Stage 1, after 8 weeks of investigational treatment and 24 weeks of background treatment, 81.0% of Subjects in the bedaquiline group and 65.2% of Subjects in the placebo group showed treatment success, (i.e., completed week 24 and were liquid culture negative at this time point).

For C208 Stage 2, in the interim analysis as well as in the primary efficacy analysis, a statistically significant difference in time to culture conversion between the treatment groups ($p < 0.0001$) in favor of bedaquiline was shown. In both analyses, an identical number of Subjects with culture conversion at week 24 (i.e., 24-week responders [missing = failure]) was observed: 78.8% in the bedaquiline group and 57.6% in the placebo group, which was statistically significantly different ($p = 0.008$) based on a logistic regression model with only treatment as covariate. Microbiological response at Week 24 was durable in C208 Stage 2: the percentage of responders (missing = failure) at week 72 was 71.2% in the bedaquiline group and 56.1% in the placebo group.

In the Phase 2b uncontrolled trial, C209, treatment with bedaquiline in combination with an individualized MDR-TB treatment regimen was effective against pulmonary MDR-TB both in newly diagnosed and in non-newly diagnosed Subjects. Culture conversion rates after 24 weeks of treatment with bedaquiline as part of an individualized anti-tuberculosis regimen were higher in Subjects with lower extent of resistance of the *M. Tb* strain and in Subjects with no lung cavitation compared to Subjects with cavitations (in one or both lungs).

2.2.1.3. Bedaquiline Clinical Safety

In the clinical studies conducted to date, a total of approximately 645 Subjects (including 265 healthy volunteers) have been exposed to bedaquiline in the Phase 1 and 2 clinical trials conducted as a part of the development program for the treatment of MDR-TB. An additional 60 Subjects received bedaquiline in a monotherapy EBA study of 14 days (study TMC207-CL001) 45 Subjects received bedaquiline, either as monotherapy (B) or in combination with other agents (B-Pa or B-Z) in study NC-001 and 45 more in study NC-003 (B-Pa-Z, B-Pa-C, B-Pa-Z-C). In these studies, bedaquiline has been shown to be an effective treatment for Subjects with both DS and MDR-TB. Specifically, the regimen B-Pa-Z was demonstrated to have efficacy at least as good as the HRZE control in study NC-003. Furthermore, bedaquiline is a novel agent with no pre-existing resistance and, based on mouse model data, may result in shortened treatment durations when included in novel regimens to treat both DS- and MDR-TB. Based on the combined experience in these clinical studies, the following known and potential risks have been identified.

Adverse Drug Reactions for bedaquiline

During the Investigational Treatment phase in the controlled trials, the most frequently reported Adverse Drug Reactions in the any bedaquiline group (> 10.0% of Subjects) were nausea, arthralgia, headache, vomiting, and dizziness. Details of Adverse Events, none of which were Serious, that were considered Adverse Drug Reactions are in the table below:

TABLE 1: Bedaquiline Adverse Drug Reactions

	Investigational Treatment Phase			
	Controlled Trials			
	TMC207		Placebo	
SOC<br>ADR (Grouped term), n (%)	24 Weeks<br>N = 79	Any<br>N = 102	24 Weeks<br>N = 81	Any<br>N = 105
At least grade 3 ADR	*5 (6.3)*	*5 (4.9)*	*0*	*0*
**Nervous system disorders**	**1 (1.3)**	**1 (1.0)**	**0**	**0**
Headache	1 (1.3)	1 (1.0)	0	0
**Cardiac disorders**	**0**	**0**	**0**	**0**
ECG QT Prolonged	0	0	0	0
**Gastrointestinal disorders**	**0**	**0**	**0**	**0**
Diarrhea	0	0	0	0
Vomiting	0	0	0	0
**Hepatobiliary disorders**	**2 (2.5)**	**2 (2.0)**	**0**	**0**
Transaminases increased [a]	2 (2.5)	2 (2.0)	0	0
**Musculoskeletal and connective tissue disorders**	**2 (2.5)**	**2 (2.0)**	**0**	**0**
Arthralgia	2 (2.5)	2 (2.0)	0	0

Mortality

Overall, there was an imbalance in the number of deaths in the pooled Stage 1 and Stage 2 C208 trial. In the pooled analysis (Stage 1 and Stage 2), 12 Subjects in the Any bedaquiline group and 5 Subjects in the Any Placebo group experienced a SAE leading to death; causes of death were varied with only death due to TB reported more than once, and none of these Subjects had a treatment-emergent QTcF ≥ 500 ms. The imbalance in deaths is primarily driven by the C208 Stage 2 results in which the imbalance was 10 bedaquiline Subjects (12.7%) compared to 3 placebo Subjects (3.7%). Based on the pooled results (Stage 1 and 2) while being followed in the placebo-controlled trial, 7 Subjects in the Any bedaquiline group and 1 Subject in the Any Placebo group died. Of these deaths, 1 occurred during the Investigational Treatment phase with bedaquiline/placebo, the remaining deaths occurred afterwards. In the Any bedaquiline group, causes of death were myocardial infarction, TB (2 cases), alcohol poisoning, hepatitis and hepatic cirrhosis (1 case), septic shock and peritonitis (1 case), and cerebrovascular accident. In the Any Placebo group, cause of death was hemoptysis. The Investigator considered the SAEs leading to death not related to bedaquiline intake in the Any bedaquiline group and doubtfully related to investigational medication in the Any Placebo group. The analysis of long-term follow-up for survival outcomes in Subjects who prematurely discontinued in trial C208 (Stage 1 and 2), based on data collection every 24 weeks (6 months) after withdrawal (up to LSLV in the rollover arm [16 Oct 2012] in Stage 2), included 9 deaths. One Subject in the bedaquiline group (pulmonary TB) and 2 Subjects in the Placebo group (TB-related illness and pulmonary TB) in Stage 1 died, and 4 Subjects in the bedaquiline group (3 Subjects with TB-related illness, 1 Subject motor vehicle accident) and 2 Subjects in the Placebo group (both TB-related illness) died after they discontinued Stage 2 of the trial. None of these Subjects had a treatment-emergent QTcF ≥ 500 ms. The imbalance in deaths is unexplained. In addition, no discernible pattern between death and sputum culture conversion, relapse, sensitivity to other drugs used to treat TB, human immunodeficiency virus (HIV) status, or severity of disease was observed.

In the uncontrolled Phase 2b trial, C209, the most frequently reported AEs during the investigational phase were hyperuricemia, arthralgia, nausea, vomiting, headache, diarrhea, blood uric acid increased, hypokalemia, pruritus, injection site pain, insomnia, and tinnitus. From start of the trial up to the final analysis, 12 Subjects died during the C209 trial due to SAEs that included TB (5 cases), congestive cardiac failure, renal impairment, lung infection, cardiac arrest (underlying cause pneumonia), hemoptysis, hypertension, and pyopneumothorax/respiratory failure. All of these SAEs leading to death were considered not related to bedaquiline by the Investigator, except for renal impairment that was judged doubtfully related to bedaquiline.

The analysis of long-term follow-up for survival outcomes for Subjects who prematurely discontinued in trial C209, based on data collection every 24 weeks (6 months) after withdrawal, included 4 deaths (all described as TB-related). In total, since the start of the C209 trial, 16 Subjects (6.9%) have died (12 Subjects during the trial and 4 Subjects during the survival follow-up phase after premature discontinuation). None of the fatal SAEs were considered related to bedaquiline by the Investigator and none of these Subjects has a treatment-emergent adverse event.

Cardiovascular safety

During clinical trials with bedaquiline a prolongation of QTc interval was observed. The US Product Label for bedaquiline[3] notes that treatment initiation is not recommended in patients with:

- Heart failure;
- QTcF interval > 450 ms (confirmed by repeat electrocardiogram);
- A personal or family history of congenital QT prolongation;
- Concomitant administration of fluoroquinolone antibiotics that have a greater potential for significant QT prolongation (i.e., gatifloxacin, moxifloxacin and sparfloxacin).
- Hypokalemia

The US Product Label recommends that bedaquiline treatment must be discontinued if the patient develops clinically significant ventricular arrhythmia. An additive or synergistic effect on QT prolongation of bedaquiline when co-administered with other medicinal products that prolong the QT interval cannot be excluded. Caution is recommended when using bedaquiline concomitantly with medicinal products with a known risk of QT prolongation. In the event that co-administration of such medicinal products with bedaquiline is necessary, clinical monitoring including frequent ECG assessment is recommended.

Hepatic safety

The US product label notes increases in transaminases were seen in clinical trials during administration of bedaquiline with the background regimen. Subjects should be monitored during treatment. Other hepatotoxic medicinal products and alcohol should be avoided while taking bedaquiline, especially in those Subjects with diminished hepatic reserve[3].

Additional safety information from a recently completed trial

In a recently completed phase I study not yet included in the bedaquiline Investigators Brochure that evaluated drug-drug interactions between bedaquiline and either rifampin or rifabutin, 7 Subjects experienced SAEs. Of those Subjects, 5 experienced SAEs of lymphocytopenia (all received rifabutin), which did not appear to be related to the AUC or C_{max} of TMC207 or rifabutin. One subject experienced an SAE of elevated Creatine Kinase (rifampicin arm) and one experienced a grade 4 Total Bilirubin SAE (rifampicin arm). Lymphopenia was previously noted to be an infrequent toxicity associated with rifabutin, but not previously seen with bedaquiline. The bedaquiline/rifabutin regimen demonstrated significant reversible lymphopenia, which did not appear to be related to the AUC or C_{max} of TMC-207 or rifabutin.

2.2.2. PA-824

As detailed in the Investigator's Brochure[15], PA-824 is a new chemical entity and a member of a class of compounds known as nitroimidazo-oxazines, which possess significant anti-tuberculosis activity and a unique mechanism of action[16]. PA-824 demonstrated *in vitro* activity against both DS- and MDR-TB[17], and *in vivo* activity in a mouse model of tuberculosis[16,17].

PA-824 has been studied in four 14-day EBA trials to date, including two monotherapy dose-ranging studies and two combination EBA studies. PA-824 monotherapy has demonstrated substantial mycobactericidal activity. The efficacy data from study PA-824-CL-007 indicated that all doses of PA-824 (200, 600, 1000 and 1200 mg) produced an equivalent decrease in sputum CFU counts over the 14-day treatment period. In study PA-824-CL-010, an EBA study with a similar design to study PA-824-CL-007 except for the use of lower doses of PA-824 (50, 100, 150 and 200 mg/day), results indicated that PA-824 treatment resulted in a measurable dose-dependent mycobactericidal activity over the dose range studied, and supported a clinical dose of 200mg per day. In study NC-001-(B-M-Pa-Z) an EBA study with multiple treatment combinations, the three drug combination of PA-824 (200 mg per day), moxifloxacin and pyrazinamide (Pa-M-Z) demonstrated potential as a treatment shortening regimen and was progressed into an 8 week "SSCC" study (NC-002) in which the combination was shown to be statistically better that the HRZE control on some measures of activity. In the second 14-day combination EBA study (NC-003), the bedaquiline, PA-824 and pyrazinamide (B-Pa-Z) regimen showed promising activity and has been selected to move forward in development and is the focus of an 8-week study in patients with DS and MDR-TB (NC-005) that will be initiated in 2014.

2.2.2.1. PA-824 Preclinical Studies

Microbiology

In vitro studies have demonstrated that the minimum inhibitory concentration (MIC) of PA-824 against a variety of drug-sensitive *M. tb.* isolates is similar to the MIC of isoniazid (MIC of PA-824, ≤0.015 to 0.25 µg/mL; MIC of isoniazid, 0.03 to 0.06 µg/mL). PA-824 was efficacious *in vitro* against drug-resistant clinical isolates of *M. tb.,* with MIC values ranging from 0.03 to 0.53 µg/mL. The minimum effective dose (MED) of PA-824 was 12.5 mg/kg/day in a mouse model of TB. The MED is defined as the lowest dose able to prevent the development of macroscopic lung lesions and splenomegaly. The minimum bactericidal dose (MBD) was 100 mg/kg/day in the same mouse model. The MBD is defined as the lowest dose able to reduce the lung TB colony forming unit (CFU) counts by 99%. The magnitude of CFU reduction by PA-824 at this dose is similar to that seen with the highest dose of isoniazid tested in the same study (25 mg/kg/day).

Nonclinical Safety Studies

The non-clinical safety evaluation of PA-824 includes pharmacology, pharmacokinetics, toxicology and metabolism studies that were conducted in accordance with current ICH guidelines.

PA-824 was negative in all genotoxicology studies performed. One of its metabolites (M50) that is found in rat, monkey, and human plasma was positive in a screening Ames assay. M50 is not a major metabolite in humans and the exposure relative to parent drug is higher in the rat (24%) and monkey (18%) than in humans (6%).

PA-824-induced effects in respiratory, CNS, and cardiovascular safety pharmacology studies were generally mild and reversible; effects were most prominent at 450 mg/kg/day. PA-824 is a weak inhibitor (IC_{50}=20uM) of the hERG channel. In a telemetry monkey study, in the dose range 50-450 mg/kg, there was no or minor prolongation of the QT interval, depending on the method of correction. The weight of evidence suggests that there should be no effect on QT in the dose range being explored in the clinical studies.

Repeat-dose toxicology studies with PA-824 have been conducted in male and female rats (14 days to 6 months), and in male and female monkeys (7 days to 3 months). In all studies, dose-dependent reduced

food consumption and reduced weight gain or weight loss were noted. In addition, testicular atrophy was observed in rats while cataracts were observed by indirect ophthalmoscopy in both rats and monkeys. In general, toxicity in both rat and monkey was significantly greater when exposures exceeded approximately 300 µg•hr/mL in the 14-day studies and approximately 200 µg•hr/mL in the 3-month studies.

Reproductive toxicology studies show that PA-824 is not teratogenic in rats or rabbits. In the rat fertility study, dose-dependent reduced fertility rates, due to decreased sperm numbers and decreased motility, were observed at doses of 30 mg/kg and greater. This effect was partially reversible. As in the 3-month rat toxicology study, irreversible testicular lesions were not observed at 30 mg/kg, only at 100 and 300 mg/kg.

To more fully characterize the cataract and male reproductive system findings, a 3-month monkey study in sexually mature males (0, 50, 150, 300 mg/kg/day), and a 6-month rat study (0, 30, 100, 300 mg/kg) in males and females were conducted. Ocular assessments were conducted in a much more careful and systematic manner than in the initial 3-month toxicology studies described above. In each of the later studies, all ophthalmologic examinations were conducted by a single ophthalmologist, using both indirect ophthalmoscopy and slit-lamp biomicroscopy. Animals were screened before dosing to ensure no animal had cataracts at baseline, and then monthly during dosing and recovery. In this monkey study, although similar drug exposures were attained as in the original 3 month monkey study, no cataracts or testes effects (semenology, organ weights, histopathology, or hormones [testosterone, follicle-stimulating hormone, Inhibin B]) were observed at any point during dosing or during a 20-week recovery period. PA-824 does not appear to cause cataracts or testicular toxicity in monkeys. In the 6-month rat study, PA-824 caused irreversible cataracts at 100 mg/kg from Day 118 of the study in males and females. In contrast to the original 3-month rat report, rats in this more carefully conducted study developed cataracts while on drug but not during recovery. The NOAEL was 30 mg/kg for cataracts and 10 mg/kg for testicular toxicity. Rats that developed cataract and testicular toxicity also experienced marked decreases in body weight gain and food consumption. The AUC safety multiples (relative to the exposures obtained at the anticipated clinical dose of 200 mg/day) for cataract are ~1.5x in the rat; in the monkey at the highest dose tolerated, where there were no cataracts in the second well conducted study, the multiple is at least 3.7x.

To summarize, cataracts have been detected in multiple animals from two similar rat studies at mid-to-high doses. In contrast, the finding of cataracts in one monkey study could not be confirmed in a follow-up study. Thus, both cataracts and the testicular effects appear to be species-limited.

An overall summary of the findings from animal safety and toxicology studies is contained in Table 2.

Table 2: PA-824 Key Animal Safety and Toxicology Findings

• Nervous system-related effects.<br><br>Rats given single oral PA-824 doses had decreased body tone, touch responses and decreased grooming behavior at ≥150 mg/kg, which resolved within 24 hours. Rats given repeated daily doses of PA-824 had convulsions, ataxia, hypoactivity, recumbency, hyperactivity and sensitivity to touch, and squinting at ≥100 mg/kg/day, and early deaths occurred at doses ≥500 mg/kg/day. Monkeys given repeated daily doses of PA-824 had hypoactivity, ataxia, tremors, and convulsions at ≥450/300 mg/kg/day. These effects were reversible when dosing stopped and were absent at ≤30 mg/kg/day in rats and ≤150 mg/kg/day in monkeys.
• Testicular toxicity<br><br>Testicular degeneration/atrophy, occurred in rats with repeated doses of PA-824 at ≥30 mg/kg/day but did not occur in monkeys at any dose level. Testicular effects showed evidence of being partially reversible, albeit very slowly, in rats dosed for 7 days, but not in rats dosed for 14 days. As would be expected, there was a dose-related decrease in fertility in male rats at ≥30 mg/kg/day that was associated with decreased sperm numbers and motility. This effect on fertility in male rats was partially reversible.
• Cataracts<br><br>Cataracts developed with prolonged daily dosing in rats at PA-824 doses ≥100 mg/kg/day. In one 13-week study in monkeys, cataracts did develop at 450/300 mg/kg/day, but only by the end of a 13-week recovery period. In a second 13-week study in monkeys that included extensive ophthalmic examinations, cataracts did not develop at the high-dose level of 300 mg/kg/day.
• hERG inhibition and QT prolongation<br><br>PA-824 inhibited hERG current with IC_{50} values of approximately 6.2 µg/mL. Following a single PA-824 dose of 450 mg/kg in monkeys, QTc interval prolongation ranged from 21 to 36 msec using Fridericia's formula (QTcF) to correct for heart rate. Coadministration of PA-824 with moxifloxacin in the monkey or with bedaquiline in the dog did not result in any greater effect on the QT interval than with either agent alone. After repeated daily doses, the QTc interval in the monkey was prolonged at PA-824 doses of ≥150 mg/kg/day.

2.2.2.2. PA-824 Clinical Studies

Phase 1

The safety, tolerability and pharmacokinetics of PA-824 have been studied in 10 Phase 1 studies, which are summarized in Table 3. In these trials, PA-824 has been administered in doses ranging from 50 to 1500 mg, as 50 or 200 mg tablets or as an oral suspension. PK parameters have largely been consistent in each study and can be summarized as follows:

- PA-824 is moderately rapidly absorbed, with median T_{max} values across Subjects and dose levels ranging from 4 to 7 hours.
- The mean half-life for elimination ($t_{½}$) across Subjects and dose levels was approximately 16 - 25 hours.
- Exposure increased approximately linearly but less than dose-proportionally, with increasing doses up to approximately 600 – 1000 mg, while higher doses achieved minimal additional increases in either C_{max} or AUC.

Two studies using radiolabeled PA-824 in an oral-suspension formulation have been conducted to investigate the metabolism and excretion patterns of PA-824 in humans: Study PA-824-CL-004, which used [benzyl-^{14}C] PA-824 and Study PA-824-CL-008, which used [imidazooxazine-^{14}C] PA-824. The mass balance results from the two studies were very similar. In each study, the majority (53-65%) of radioactivity was excreted in the urine; an additional 26-38% was collected in the feces such that approximately 91% of the administered dose was ultimately recovered in the excreta.

Radioprofiling and metabolite identification work have been completed on samples from the two human studies as well as from analogous work in rat and monkey using both radiolabeled PA-824 preparations. The metabolism of PA-824 proceeds via a combination of reductive metabolism (~20 – 25% of the dose) and oxidative metabolism (remainder of the characterized metabolites). The metabolic profile of PA-824 *in vivo* was qualitatively similar in the three species, with quantitative differences being minor. No human unique metabolites were detected and there is no one single metabolic path that can be considered major. Furthermore, there are no major metabolites in human plasma.

Study PA-824-CL-006, a drug-drug interaction study with midazolam to assess the extent of CYP3A inhibition by PA-824, results indicate that dosing of PA-824 400 mg once daily for 14 days did not have a major effect on the exposure of midazolam or its major metabolite 1-hydroxy midazolam. For midazolam, the geometric mean ratio of Day 17 (midazolam+PA-824) vs. Day 1 (midazolam alone) for C_{max} was 0.84 and AUC was 0.85. For the 1-hydroxy midazolam metabolite, the corresponding geometric mean ratio for Cmax was 1.05 and AUC was 1.11. The data suggests that PA-824 does not cause clinically significant drugs interactions with drugs metabolized by CYP3A.

Two additional studies have recently been completed and are currently undergoing analysis: Study DMID 10-0058 (a Thorough QT study comparing PA-824 and PA-824 plus moxifloxacin to moxifloxacin alone) and Study ACTG 5603 (a drug-drug interaction study evaluating the effects of concomitantly administered Efavirenz, Ritonavir-Boosted Lopinavir or rifampicin on the PK parameters of PA-824). The first study found that single doses of PA-824 of 400mg and 1000mg did not have a clinically significant effect on the QTc interval, and when PA-824 at 400mg is co-administered with moxifloxacin (400mg) it did not increase the QTc prolongation substantially over what is seen with moxifloxacin alone. The second study found that when administered with Efavirenz, Ritonavir-Boosted Lopinavir, or Rifampicin, PA-824 (200mg) median PA-824 concentrations (AUC_{0-24h}) were reduced 35% by EFV, 17% by LPV/r, and 66% by rifampin. The clinical significance of these findings requires further investigation.

Study PA-824-CL-009, a food effects study PA-824 (200 mg and 50 mg); results indicate that the food effect observed is dependent on the PA-824 dose administered. When a single dose of PA-824 was administered with a high fat, high calorie meal, C_{max} and AUC of the 50 mg dose increased 1.40-fold and 1.45-fold respectively, whereas for the 200 mg dose, C_{max} increased 1.76-fold and AUC increased 1.88-fold.

Table 3: PA-824 Phase 1 Clinical Studies

Study	Design	PA-824 Dose	Enrolled	Key Findings
**CL-001**	Double-blind, placebo-controlled, single-dose, dose-escalating, PK and safety study	0, 50, 250, 500, 750, 1000, 1250, 1500	53	• Well tolerated; no dose-limiting AEs or abnormal laboratory results; no effects on ECG, vital signs, or PE.
**CL-002**	Double-blind, placebo-controlled 7-day multidose, escalating, PK and safety study	0, 200, 600, 1000	24	• Well tolerated; no effects on ECG, vital signs, or PE.<br>• After 5 days' dosing at 1000 mg/d, progressive moderate creatinine elevation: reversed during 7-day washout period.<br>• No consistent effect on BUN.<br>• A planned 1400-mg cohort not enrolled.
**CL-003**	Open-label, single-dose, food effects	1000	16	• Well tolerated; no dose-limiting AEs or abnormal laboratory results; no effects on ECG, vital signs, or PE.<br>• Treatment-emergent AEs affecting more than one Subject occurred more frequently after dosing in the fed condition than the fasted condition, and more frequently among women than men.<br>• Bioavailability is 3.5-to-4.5-fold higher when PA-824 is administered within 30 minutes of a high-fat, high-calorie meal than when it is administered after an overnight fast.
**CL-004**	Open-label, single-dose, ADME	~860, oral suspension [benzyl-^{14}C]PA-824	6	• Well tolerated; no dose-limiting AEs or abnormal laboratory results; no effects on ECG, vital signs, or PE.<br>• No significant radioactivity captured as [benzyl-^{14}C]CO_2.<br>• ~91% of dose recovered (~65% in urine; ~26% in feces)<br>• Plasma: parent drug and one major metabolite.<br>• Urine: little or no parent drug; multiple major metabolites.<br>• Feces: minimal unchanged parent drug; numerous low-abundance metabolites.

Study	Design	PA-824 Dose	Enrolled	Key Findings
**CL-005**	Double-blind, 8-day multidose, renal effects	0, 800, 1000	47	• Well tolerated; no dose-limiting AEs or abnormal laboratory results; no effects on ECG, vital signs, or PE.<br>• As anticipated, serum/plasma creatinine levels increased significantly (up to ~ 40%) during treatment; reversed during 7-day washout period.<br>• No effect during treatment on GFR, ERPF, FF, BUN or UA.
**CL-006**	Open-label, multidose, DDI	400	14	• Well tolerated; no dose-limiting AEs.<br>• For midazolam, the geometric mean ratio of Day 17 (midazolam+Pa-824) vs. Day 1 (midazolam alone) for C_{max} was 0.84 and $AUC_{(0\text{-infinity})}$ was 0.85.<br>• For the 1-hydroxy midazolam metabolite, the corresponding geometric mean ratio for Cmax was 1.05 and $AUC_{(0\text{-infinity})}$ was 1.11.
**CL-008**	Open-label, single-dose, ADME	~1100, oral suspension [imidazooxazine-^{14}C]PA-824	6	• Well tolerated; no dose-limiting AEs or abnormal laboratory results; no effects on ECG, vital signs, or PE.<br>• No significant radioactivity captured as [imidazooxazine-^{14}C]CO_2.<br>• ~91% of dose recovered (~53% in urine; ~38% in feces) Plasma: parent drug. Urine: little or no parent drug; multiple major metabolites.<br>• Feces: unchanged parent drug and numerous low-abundance metabolites.
**CL-009**	Open-label, single-dose, food effects	50 and 200	32	• Well tolerated; no dose-limiting AEs.<br>• In the presence of high fat, high calorie diet, C_{max} and AUC of the 50-mg dose increased 1.40-fold and 1.45-fold respectively, whereas for the 200-mg dose, C_{max} increased 1.76-fold and AUC increased 1.88-fold.
**A5306**	Antiretroviral DDI	200	48	• Based on preliminary data – the study is currently undergoing analysis.<br>• Co-administration with Efavirenz resulted in a 35% reduction in PA-824 AUC.<br>• Co-administration with Ritonavir-Boosted Lopinavir resulted in a 17% reduction in PA-824 AUC.<br>• Co-administration with rifampicin resulted in a 66% reduction in PA-824 AUC.

Study	Design	PA-824 Dose	Enrolled	Key Findings
**10-0058**	Thorough QT Study	400 and 1000	75	• PA-824, alone and in combination with moxifloxacin, was well tolerated.<br>• PA-824 doses of 400 mg and 1000 mg did not cause QT interval prolongation to a level of clinical concern as the upper limit of the 90% CI associated with any LS mean ΔΔQTcI value did not exceed 4.4 ms for the 400-mg dose or 6.1 ms for the 1000-mg dose, and both were well below 10 ms.<br>• The effect of PA-824 400 mg plus moxifloxacin 400 mg on QTcI was similar to the effect of moxifloxacin administered alone.<br>• No Subject receiving PA-824 or moxifloxacin alone had an observed QTcI value that exceeded 450 ms or experienced a change-from-baseline in QTcI that exceeded 30 ms.<br>• The PK of PA-824 was not affected by the coadministration of moxifloxacin.

- **Phase 2**

Study PA-824-CL-007, a 14 day monotherapy EBA study, indicated that all doses of PA-824 (200, 600, 1000 and 1200mg a day) produced a measurable and equivalent decrease in sputum CFU counts over the 14-day treatment period. Study PA-824-CL-010 was an EBA study with a similar design to study PA-824-CL-007 except for the use of lower doses of PA-824 (50, 100, 150 or 200 mg/day). Results indicate that PA-824 treatment resulted in a measurable dose-dependent mycobactericidal activity, with the 50 mg dose demonstrating less activity than the 100, 150 and 200 mg doses, which were all equivalent.

Study NC-001-(B-M-Pa-Z) was a 14 day EBA study that assessed the two-week EBA of the following drug combinations: PA-824 plus pyrazinamide, PA-824 plus pyrazinamide plus moxifloxacin, along with two other non-PA-824 containing combinations. Results indicate that the three drug combination of PA-824 (200 mg per day), moxifloxacin and pyrazinamide has potential as a treatment shortening regimen. In the study this three drug combination has an EBA 0-14, which is believed indicative of sterilizing activity, numerically better than the current 4-drug intensive phase treatment of HRZE.

The recently completed Phase 2b study, NC-002, was a multi-center open-label partially randomized clinical trial with four treatment groups. Subjects with drug-sensitive TB were randomized to receive moxifloxacin 400 mg plus PA-824 100 mg plus pyrazinamide 1500 mg (M-PA100-Z) or moxifloxacin 400 mg plus PA-824 200 mg plus pyrazinamide 1500 mg (M-PA200-Z) or standard HRZE therapy. HRZE was included as a control arm for the drug-sensitive treatments and for the laboratory methodology. Subjects with MDR-TB received moxifloxacin 400 mg plus PA-824 200 mg plus pyrazinamide 1500 mg (M-PA200-Z MDR). The study population included a total of up to 230 male and female newly diagnosed Subjects with drug-sensitive or multi drug-resistant, smear positive pulmonary tuberculosis aged 18 to 65 years (inclusive). The primary efficacy endpoint was the rate of change in the logarithm of colony forming unit (CFU) (or log[CFU]) count) over 8 weeks of treatment analysed by a Joint Bayesian Non-linear Mixed Effect (NLME) regression.

Preliminary analyses of NC-002 indicate that a total of 207 Subjects were enrolled, with 60 randomized to M-PA100-Z, 62 randomized to M-PA200-Z, and 59 to HRZE. An additional 26 Subjects were treated in the M-PA200-Z MDR-TB arm. Of note, more Subjects in the MDR-TB arm did not complete the full 8 weeks of treatment, primarily because many were withdrawn as late-exclusions (*M. Tb* resistant to pyrazinamide determined in culture after enrollment in the study). Twenty-one MDR-TB Subjects were in the study with active treatment through day 14 and 10 were in the study through the full 8 weeks of treatment (9 with

evaluable results for the primary microbiological endpoint). In contrast, the following number of Subjects in the study with active treatment through 8 weeks in the 3 randomized arms with evaluable results for the primary microbiological endpoint: 55 in the M-PA100-Z arm, 54 in the M-PA200-Z arm and 54 in the HRZE arm. For the primary endpoint, Subjects in the M-PA200-Z arm had a statistically significantly greater decline in the log CFU count over the 8 weeks, than the Subjects in the HRZE arm. Finally, PA-824 has recently been studied in combination with bedaquiline and other agents in a 2 week EBA study (NC-003).

Table 4: PA-824 Phase 2 Studies

Study	Design	PA-824 Doses	Enrolled	Key Findings
**CL-007**	Partially double-blinded (blinded as to PA-824 dose), 14-day multidose, extended early bactericidal activity.	200, 600, 1000, 1200	69	• Overall well tolerated with relatively few AEs and no dose-limiting AEs or laboratory findings. No clinically significant effects on ECG, vitals, or PE noted.<br>• Two SAEs occurred during study, both were considered possibly related to TB disease (hemoptysis).<br>• PA-824 treatment produced a measurable decrease in log CFU, with the magnitude of effect equivalent for all doses.
**CL-010**	Partially double-blinded (blinded as to PA-824 dose), 14-day multidose, extended early bactericidal activity.	50, 100, 150, 200	69	• Well tolerated.<br>• PA-824 treatment produced a measurable decrease in log CFU with some evidence of dose dependence.
**NC-001**	Partially double-blinded (blinded as to combination within Pa or B containing arms), 14-day multidose, extended early bactericidal activity.	200	85	• Well tolerated.<br>• PA-824 plus moxifloxacin plus pyrazinamide combination treatment produced a decrease in log CFU at least comparable to that of the Rifafour e-275® control group.
**NC-002**	Multi-center open-label partially randomized clinical trial in four treatment groups. Subjects with drug-sensitive TB randomized to receive moxifloxacin 400 mg plus PA-824 100 mg plus pyrazinamide 1500 mg or moxifloxacin 400 mg plus PA-824 200 mg plus pyrazinamide 1500 mg or Rifafour e-275®.	100, 200	207	Based on preliminary results:<br>• For the primary endpoint Subjects in the M-PA200-Z arm had a statistically significantly greater decline in the log CFU count over the 8 weeks than the Subjects in the HRZE arm.<br>• Well tolerated overall.<br>• Eleven serious adverse events (SAEs) were reported in 9 Subjects, with one Subject in each of the M-PA100-Z and the Rifafour® groups, and 7 Subjects in the M-PA200-Z group.
**NC-003**	Multi-center, open-label, randomized clinical trial with seven parallel treatment arms. Fifteen Subjects were enrolled in each of the following treatment arms: TMC207 plus PA-824 plus pyrazinamide plus clofazimine, TMC207 plus PA-824 plus pyrazinamide, TMC207 plus PA-824 plus clofazimine, TMC207 plus pyrazinamide plus clofazimine, pyrazinamide alone, clofazimine alone, and Rifafour e-275®.	200	105	• Among the regimens studied, the combination B-PA-Z demonstrated the highest EBA, with results at least comparable to the HRZE control.<br>• The treatments administered in this trial were well tolerated by the trial population.<br>• No deaths were reported in this trial. Serious AEs were reported for 1 Subject (1.0%) in the clofazimine alone arm: gastroenteritis, anemia, and deep vein thrombosis (none of which were considered to be related to the treatment).

2.2.2.3. PA-824 Clinical Safety

Across the 15 clinical studies with PA-824 completed to date, a total of 649 Subjects have been exposed to PA-824, including 289 healthy Subjects across the 10 Phase 1 studies and 360 Subjects with newly diagnosed smear positive pulmonary TB across 5 Phase 2 studies. Among the 289 healthy Subjects, 174 received exposure to a single dose of PA-824 ranging from 50 to 1500 mg and 115 received exposures to repeated daily doses of PA-824 (50 to 1000 mg) for up to 14 days. The 360 Subjects with newly diagnosed smear positive pulmonary TB were exposed to PA-824 either as a single agent at daily doses of 50 to 1200 mg for 14 days or in combination with other anti-TB agents (bedaquiline, moxifloxacin pyrazinamide and/or clofazimine) at a dose of 100 mg or 200 mg for up to 56 days. The overall safety profile determined from the clinical studies completed to date indicates PA-824 is well tolerated in healthy adults and in TB Subjects when administered alone and in combination with moxifloxacin, pyrazinamide, bedaquiline and clofazimine.

In the Phase 1 studies in healthy volunteers the most common side effects or AEs associated with PA-824 exposure include:

- Headache;
- Benign, isolated and reversible elevations of serum creatinine;
- Stomach discomfort (nausea, vomiting, flatulence, and/or diarrhea);
- Skin and subcutaneous tissue disorders.

Key safety considerations of special concern, based on preclinical or clinical findings in the program to date, are noted below and will be under close scrutiny in future trials:

Cataracts

While the detailed examinations in Phase 2 have not raised concern for humans, ophthalmologic examinations, with slit lamp exam and grading of lens opacities, will continue in NiX-TB. These examinations are to follow up on the finding of cataracts in rats exposed to PA-824 in preclinical studies.

Testicular Toxicity

Clinical evaluations of potential testicular toxicity in NC-002 failed to demonstrate any effect, based on evaluations of testosterone, LH and FSH values at baseline and after 2 months of daily dosing of the M-Pa-Z regimen. However to follow up on findings in the male rat of testicular toxicity, all male subjects in the upcoming Phase 3 trial of the M-Pa-Z regimen will have evaluations of male hormones, and a substudy will evaluate changes in semen and sperm parameters in a subset of patients receiving the regimen over 6 months.

Central Nervous System

The PA-824 pre-clinical program identified potential CNS-related toxicities and one Subject treated with PA-824 in clinical study NC-002 experienced a seizure while on treatment. Close surveillance will be in place to identify any seizures or significant central nervous system (CNS) signs or symptoms during the NiX-TB study.

Hepatic Safety

Hepatic enzyme increases have been seen in Subjects treated with PA-824 in combinations with various other medications during the clinical development program. It is difficult to assign specific causality to any one drug within a regimen; nonetheless, the NiX-TB will include specific monitoring of hepatic enzymes.

Detailed Safety Findings in PA-824 Clinical Development

In Phase 1 trials at the clinical dose of 200mg or lower, the incidence of headache was approximately 20-30% and similar to placebo. At doses of 800mg and higher, usually in trials without a placebo or comparator arm, the incidence of headache reached 80%. Headache occurrence was typically higher in studies with longer confinement periods. Throughout the development program, other common TEAEs include elevated serum creatinine, stomach discomfort (including nausea and other gastrointestinal symptoms such as flatulence and/or diarrhea), and skin and subcutaneous tissue disorders (including erythema, pruritus and rash). Skin and subcutaneous tissue disorders, followed by stomach discomfort were the most commonly reported TEAEs in the Phase 2 monotherapy studies (PA-824-CL-007 and PA-824-CL-010). Within Study PA-824-CL-007, a higher incidence of PA-824 TEAEs were observed in the higher PA-824 dose groups (PA-824 200 mg: 7%; PA-824 600 mg: 13%; PA-824 1000 mg: 31%; and PA-824 1200 mg: 33%). The incidence of the TEAEs in the Rifafour® treated group was 25%. Study PA-824-CL-010, among the four PA-824 treatment groups (50 mg, 100 mg, 150 mg, and 200 mg) and the Rifafour® treatment group, a higher incidence of TEAEs was observed in the 50mg PA-824 group (66.7%) when compared with Rifafour® (50.0%) and the other PA-824 treatment groups. For the multidose, placebo-controlled Studies PA-824-CL-002 and PA-824-CL-005, overall AE frequency tended to be greater among PA-824 Subjects than among placebo Subjects, and tended to be higher in higher PA-824 dose groups.

Study PA-824-CL-005 was undertaken to determine the mechanism responsible for the elevation in serum creatinine seen with PA-824 dosing in studies PA-824-CL-001 and PA-824-CL-002. This study explored the effects of PA-824 on kidney function by measuring glomerular filtration rate (GFR), effective renal plasma flow (ERPF), filtration fraction (FF, calculated as GFR/ERPF), and creatinine clearance. Subjects were dosed in blinded fashion with placebo, 800 mg PA-824, or 1000 mg PA-824 for 8 days. Serum creatinine levels rose in both the 800- and 1000-mg/day PA-824 groups, by an average of 0.18 mg/dL (19%) and 0.25 mg/dL (27%) in the two groups respectively by Day 8; the largest individual increase was approximately 40% over baseline. In this study, although serum creatinine levels rose, no meaningful effects were noted during the dosing period on GFR, ERPF, BUN, uric acid or FF. As expected, creatinine clearance was reduced concomitantly with maximally elevated serum creatinine levels relative to baseline. Taken together, these results indicate that PA-824 does not negatively affect renal function. Instead, the drug can be assumed to cause its effects on serum creatinine by inhibiting tubular creatinine secretion; such an effect has been reported with other approved drugs (e.g. cimetidine) and is not considered clinically significant.

Across all studies, the great majority (>~95%) of AEs resolved without sequelae. In Study PA-824-CL-005, one Subject treated with 800 mg PA-824 was discharged with three ongoing AEs (proteinuria [nephrotic range during the study, but non-nephrotic range in follow-up], hypoalbuminemia, and iron deficiency). The proteinuria and hypoalbuminemia were moderate in severity and the iron deficiency was mild. This Subject substantially improved, and the Subject is seen periodically by a nephrologist. A renal biopsy performed 20 months post-study revealed focal segmental glomerulosclerosis likely secondary type, although the Subject remains fundamentally healthy with normal renal function indices and no signs of peripheral edema or hypertension. A complete review of her screening and check-in laboratory values suggests, in the opinion of the Sponsor, that she might have had a pre-existing undiagnosed clinical condition including atypical lipid profile, BUN below the lower limit of the normal range, and ALT and AST above the upper limit of normal range. Furthermore, her eosinophil count was above-normal at Screening at 6.7% and progressively rose during the study to 8.9% by Day 15 and she reported a personal and family history of allergies and rhinorrea. The Investigator considered this individual normal and meeting the protocol entry criteria, and enrolled this Subject.

In most of the completed Phase 2 studies, no Subjects discontinued from the study as a result of AEs. In Study PA-824-CL-002, dosing for all Subjects in the 1000 mg dose group was discontinued on Day 5 in response to rising serum creatinine levels. In Study PA-824-CL-005, one Subject was discontinued for safety reasons in relation to a severe rash that developed approximately 32 hours after the 8th and last dose of

PA-824 (1000 mg). The rash symptoms were treated with diphenhydramine, prednisone, and hydroxyzine at various points during the ensuing approximately 9 days until the symptoms completely resolved. In Study PA-824-CL-007, two Subjects (one in the 200 mg/day PA-824 group and one in the control arm) were discontinued as a result of disease-related hemoptysis. Each of these events was classified as an SAE, both resolved with treatment in hospital and neither was considered possibly related to the study drugs. In Study PA-824-CL-010, one Subject was withdrawn due to the SAE of pneumothorax after 4 days' dosing, which resulted in hospitalization and later resolved. The SAE was deemed related to the Subject's concurrent tuberculosis and unrelated to PA-824.

Post-study follow-up ophthalmic examinations were performed on Subjects and Subjects enrolled in two studies (PA-824-CL-005 [n=30] and PA-824-CL-007 [n=46]) where Subjects received the highest doses of PA-824 for the longest duration among the clinical studies conducted to date. Male and female healthy volunteers were treated at doses up to 1000 mg/day for 8 days in study PA-824-CL-005, and male and female TB Subjects were treated at doses up to 1200 mg/day for 14 days in study PA-824-CL-007. Two ocular events were reported, one cataract was among the 12 Subjects from the 1200 mg PA-824 group in study PA-824-CL-007 and the other cataract was from among the 5 Subjects within the HRZE group.

In NC-001-(B-M-Pa-Z), five Subjects were discontinued prior to completion of their treatment with a PA-824 containing arm. One Subject receiving PA-824 (200mg), moxifloxacin (400 mg), and pyrazinamide (dosed by weight) experienced an SAE considered by the Investigator unrelated to the drug combination. The SAE consisted of convulsion as well as aggressive and violent behaviors. After a CT scan, the Subject was diagnosed with neurocysticercosis. A second Subject receiving PA-824 (200mg), moxifloxacin (400 mg), and pyrazinamide (dosed by weight) was withdrawn on Treatment Day 5 based on a protocol specified criterion of an increase from baseline in QTcF and QTcB greater than 60 msec on repeated ECGs and accompanied by clinically relevant T-wave morphology changes. On Day 5, the Subject had prolonged QTc values (>60 msec) on the pre-dose ECG; however, on repeat ECGs, the QTc values stabilized satisfactorily. Five hours post-dose on Day 5, ECG QTc values were again increased (>60 msec) from baseline and repeat ECGs also revealed T-wave changes. The Subject was, therefore, withdrawn from the study as specified in the protocol. In addition, two Subjects receiving PA-824 (200mg), and pyrazinamide (dosed by weight) were withdrawn due to Grade 3 ALT levels, although the elevation in ALT in one of these Subjects occurred prior to the first dose of study medication. One Subject receiving PA-824 (200 mg) and bedaquiline (400 mg) was withdrawn due to a Grade 3 ALT elevation. Overall in the trial, 53% of the 15 Subjects in the PA-824 + pyrazinamide treatment arm experienced treatment-emergent adverse events, as compared with 53% of the 15 Subjects in the PA-824 + pyrazinamide + moxifloxacin arm and 25% of the 8 Subjects in the HRZE (control) arm. All of these adverse events were rated by the Investigator as mild or moderate. 7% of Subjects in the PA-824 + pyrazinamide treatment arm experienced liver enzyme elevations, as compared with 20% of Subjects in the PA-824 + pyrazinamide + moxifloxacin arm and 0% in the Rifafour® arm, accounting for most of the imbalance between groups. All liver enzyme elevations were < 3x ULN except for two cases.

Also in NC-001-(B-M-Pa-Z), changes in QT interval were assessed pre-dose and at 2hrs and 5 hrs post-dose on each day of the study for the PA-824 + pyrazinamide and PA-824 + pyrazinamide + moxifloxacin treatment arms. On Day 14, the last dosing day, no Subject in either treatment group had a corrected QT interval (QTcF) > 450 msec. One Subject in the PA-824 + pyrazinamide arm had a QTcF increase of between 30 and 60 msec; no Subject had a QTcF increase > 60 msec. No Subjects in the PA-824 + pyrazinamide + moxifloxacin had a QTcF increase > 30 msec.

Safety Findings in 8-Week Study NC-002

Preliminary data analyses of the study NC-002 indicate that a total of 207 Subjects were enrolled, with 60 randomized to M-PA100-Z, 62 randomized to M-PA200-Z, and 59 to HRZE. An additional 26 Subjects were treated in the M-PA200-Z MDR-TB arm. In this study 88% of all Subjects had a treatment emergent adverse event (TEAE), including 87% in the M-PA100-Z group, 92% in the M-PA-Z group, 85% in the HRZE group and

89% in the M-PA-Z MDR-TB group. Adverse events were graded according to the NIH Division of Microbiology and Infectious Diseases Adult Toxicity Table.

Eleven serious adverse events (SAEs) were reported in 9 Subjects, with one Subject in each of the M-PA100-Z and the HRZE groups, and 7 Subjects in the M-PA200-Z group. The Subject in the M-PA100-Z group died of an unknown cause 39 days after a single dose of study drug regimen and the death was not considered related to study drug by the Investigator or the Sponsor. Four other SAEs were considered not related to study drug, including a pneumothorax, a bone fracture, dyspnea requiring hospitalization, and second degree heart block considered on evaluation to be existing prior to entry in to the trial. SAEs considered possibly related or related to the study drug regimen included hyperuricemia likely secondary from pyrazinamide, drug-induced hepatitis and elevated liver enzymes. One Subject had an episode of agranulocytosis that resolved after the study drug regimen was stopped and one Subject had a seizure witnessed by the family and was discontinued from the study.

The protocol required that Subjects with hepatic enzyme ALT or AST elevations greater than 3X the Upper limit of Normal (ULN) must have study drug discontinued. Consequently, 25 Subjects were withdrawn from the study across the study arms for elevations in hepatic enzymes. The distribution of elevations in ALT across the study arms is presented in Table 5. While more Subjects in the M-PA200-Z group had elevations in ALT >3 – 5X ULN, those with elevations >5X ULN or >8X ULN were fairly evenly distributed across the groups of Subjects with drug-sensitive *M. tb*.

Table 5: NC-002 Elevations in Alanine Aminotransferase

ALT	Statistic	M-PA100-Z (N=60)	M-PA200-Z (N=62)	HRZE Control (N=59)	M-PA200-Z MDR (N=26)
> 3X ULN	N (%)	7	10	5	3
> 5X ULN	N (%)	4	5	4	2
> 8X ULN	N (%)	2	4	3	1

Note: Groups are not mutually exclusive: >3X includes >5X and >8X; >5X includes >8X

Ophthalmologic Evaluations

All Subjects received ophthalmologic evaluations using the AREDS2 grading system across a range of 0-4 including visual acuity testing and slit lamp examinations at baseline and 3 months after completion of study drug dosing. All Subjects enrolled with the required zero score grade for all regions of the lens except for 1 Subject who was blind in one eye. Among all Subjects in the trial, 4 Subjects had lens evaluations with a grade of greater than zero. One Subject in the M-PA100-Z group and 3 Subjects in the M-PA200-Z group had grades of 0.5 or 1.0 in a single eye in one of the 3 zones of the lens. It is unlikely these findings represent a drug-induced lens opacity given the low incidence, the unilateral nature of all findings and the differing zone locations of the findings. It is common in persons with no clinical abnormalities to have grades of 0.5 – 1.0+ in the AREDS2 rating on a slit lamp evaluation.

Reproductive Hormone Evaluations

In study NC-002 men were evaluated with plasma samples for the reproductive hormones LH, FSH and Testosterone at baseline and at the end of the dosing period. If the study drug regimen caused testicular toxicity, the most sensitive measure from these hormones would be an increase in levels of FSH. Among Subjects in the M-PA100-Z group the mean baseline FSH was 9.027 U/L which decreased to 8.338 U/L at the

end of therapy. Among Subjects in the M-PA200-Z group the mean baseline FSH was 6.531 U/L at baseline and this decreased to a mean of 6.061 at the end of therapy. Men in the Rifafour® group had a mean baseline of 7.394 U/L which decreased to 6.714 at the end of therapy. This gives relative reassurance that the M-Pa-Z regimen is not likely to cause testicular toxicity in men.

Electrocardiographic Conduction Interval Changes

Subjects in NC-002 had supine resting ECGs taken at baseline, Day 4 and weekly through the 8 week dosing period and 2 weeks after the end of dosing. All ECGs were read by a central cardiology service. No Subjects had a corrected QT interval (QTcF) greater than 500 msec during the study. A small number of Subjects had asymptomatic increases in QTcF from baseline over 60 msec: Two in the M-PA100-Z group, 4 in the M-PA200-Z group, none in the Rifafour® group and 2 in the M-PA200-Z MDR group. An evaluation of the mean change from baseline across all post-baseline ECGs notes increases of 11.1 msec in the Rifafour® group, 11.1 in the M-PA100-Z group, 17.8 msec in the M-PA200-Z group and 6.7 in the M-PA200-Z MDR-TB group. Of note, many Subjects were tachycardic at baseline with their active pulmonary *M. tb.* and had heart rates decrease over the first week of therapy. This fact complicates interpretation of the data based on the QT correction factors that are imperfect when correcting for heart rates that change over time.

2.2.3. Linezolid

Linezolid is a synthetic antibacterial agent of the oxazolidanone class approved in many countries around the world (including South Africa), for drug-resistant, gram-positive bacterial infections, including gram positive organisms such as *Staphylococcus aureus*, coagulase negative *Staphlococcus* and *Enterococcal* infections. The recommended dose for these infections is 600 mg twice daily for up to 28 days of therapy[18, 31, 34]. Antimicrobial effects likely come from inhibition of protein synthesis in the ribosomes of the infecting organism[25]. Resistance of *M.tb*. to linezolid is rare, as this drug has not been widely used to treat tuberculosis. In the recent study using linezolid to treat patients with XDR-TB in Korea, none of 41 patients had resistance to linezolid at baseline[24].

Preclinical *in vitro* data shows linezolid is active against *Mycobacterium tuberculosis* (*M.tb.*), including MDR strains with minimum inhibitory concentrations (MICs) that range from 0.125-1 μg/mL[26]. Recent studies of the bactericidal and sterilizing activity of linezolid in a mouse model of *M.tb.* infection have demonstrated linezolid alone causes marked reductions in lung colony forming units (CFUs) from mice following 1-3 months of therapy (Table 6, below). (Data not yet published, E. Nuermberger personal communication.)

Table 6: Murine Lung CFU counts during Treatment with Linezolid monotherapy versus Standard Therapy

	Mean lung log_{10} CFU count (± S.D.) at:			
**Regimen**	**D0**	**Month 1**	**Month 2**	**Month 3**
Untreated	6.17 ± 0.27	6.50 ± 0.08		
2RHZ/4RH		3.47 ± 0.37	1.59 ± 0.25	0.52 ± 0.36
L		4.97 ± 0.23		

In recent years linezolid has been used to treat patients with MDR and XDR-TB, although there have been no fully controlled trials of linezolid in a regimen for this indication. The World Health Organization management guidelines place linezolid in Group 5 ("Agents with unclear role in treatment of drug resistant-TB") in their groups of drugs to treat MDR-TB.[27] Over the past 10 years small retrospective observational

studies have reported good results when linezolid has been added to failing regimens for patients with MDR-TB[28, 29, 30] . The most compelling recent evidence linezolid may be of benefit to patients with XDR-TB was reported by Lee and colleagues from a study in S. Korea[24]. Forty-one patients who had sputum culture–positive XDR-TB and who had not had a response to any available chemotherapeutic option during the previous 6 months were randomized to start linezolid at 600 mg daily or to delay therapy with linezolid at 600 mg daily for 2 months without changing their failing background regimen. After confirmed sputum-smear conversion, or at 4 months, patients underwent a second randomization to continued linezolid therapy at a dose of 600 mg per day or 300 mg per day for at least an additional 18 months. Thirty four of 39 (87%) of the patients had a negative sputum culture within 6 months after linezolid had been added to their drug regimen. As of the cutoff date prior to publication, of the 38 patients who received linezolid, 17 were still receiving the treatment per protocol, and 13 had completed treatment, including 6 with no relapse during the treatment period, 4 with no relapse at the 6-month follow-up, and 3 with no relapse at the 12-month follow-up (end of study).

Linezolid Clinical Safety

Linezolid is currently marketed globally, including South Africa, for a variety of acute infectious diseases and has been studied for the treatment of XDR-TB in several recent trials, including in South Africa[24,33]. The following list of known and potential risks is based on the warnings and precautions and adverse reactions sections of the current package label[18,31, 34]. Of note, the approved indication for linezolid is for administration up to 28 days.

Warnings and Precautions

- Linezolid should not be used in patients taking any medicinal product which inhibits monoamine oxidases A or B (e.g. phenelzine, isocarboxazid) or within 2 weeks of taking any such product.
- Myelosuppression (including anemia, leukopenia, pancytopenia, and thrombocytopenia) has been reported in patients receiving linezolid. In cases where the outcome is known, when linezolid was discontinued, the affected hematologic parameters have risen toward pretreatment levels. Complete blood counts should be monitored weekly in patients who receive linezolid, particularly in those who receive linezolid for longer than two weeks, those with pre-existing myelosuppression, those receiving concomitant drugs that produce bone marrow suppression or those with a chronic infection who have received previous or concomitant antibiotic therapy. Discontinuation of therapy with linezolid should be considered in patients who develop or have worsening myelosuppression.
- Lactic acidosis has been reported with the use of linezolid. In reported cases, patients experienced repeated episodes of nausea and vomiting. Patients who develop recurrent nausea or vomiting, unexplained acidosis, or low bicarbonate level while receiving linezolid should receive immediate medical evaluation.
- Spontaneous reports of serotonin syndrome associated with the co-administration of linezolid and serotonergic agents, including antidepressants such as selective serotonin reuptake inhibitors (SSRIs), have been reported. Where administration of linezolid and concomitant serotonergic agents is clinically appropriate, patients should be closely observed for signs and symptoms of serotonin syndrome such as cognitive dysfunction, hyperpyrexia, hyperreflexia and incoordination. If signs or symptoms occur physicians should consider discontinuation of either one or both agents. If the concomitant serotonergic agent is withdrawn, discontinuation symptoms can be observed (see package insert of the specified agent(s) for a description of the associated discontinuation symptoms).
- Peripheral and optic neuropathy has been reported in patients treated with linezolid, primarily those patients treated for longer than the maximum recommended duration of 28 days. In cases of optic neuropathy that progressed to loss of vision, patients were treated for extended periods beyond the maximum recommended duration. Visual blurring has been reported in some patients treated with linezolid for less than 28 days. If patients experience symptoms of visual impairment, such as changes in visual acuity, changes in color vision, blurred vision, or visual field defect,

prompt ophthalmic evaluation is recommended. Visual function should be monitored in all patients taking linezolid for extended periods (≥ 3 months) and in all patients reporting new visual symptoms regardless of length of therapy with linezolid. If peripheral or optic neuropathy occurs, the continued use of linezolid in these patients should be weighed against the potential risks. Additional information on the neuropathies reported in recent studies of linezolid administered over prolonged periods to patients with TB infection is presented above in Section 2.2.3.

- Convulsions have been reported in patients when treated with linezolid. In some of these cases, a history of seizures or risk factors for seizures was reported.
- Postmarketing cases of symptomatic hypoglycemia have been reported in patients with diabetes mellitus receiving insulin or oral hypoglycemic agents when treated with linezolid, a reversible, nonselective MAO inhibitor. Some MAO inhibitors have been associated with hypoglycemic episodes in diabetic patients receiving insulin or hypoglycemic agents. While a causal relationship between linezolid and hypoglycemia has not been established, diabetic patients should be cautioned of potential hypoglycemic reactions when treated with linezolid.

Longer term use of linezolid in patients with TB has been limited by the high cost of the drug and concerns about the toxicities of myelosuppression, peripheral neuropathy and optic neuropathy. Published reports of observational trials and case series note use of linezolid at doses ranging from 300 mg daily to 1200 mg daily over many months. The most complete review is a meta-analysis by Cox which noted the proportion of adverse events necessitating treatment discontinuation was significantly different by dose: 29.49% (95%CI 3.24–55.74) for ⩽600 mg daily vs. 60.75% (95%CI 42.69–78.81) for >600 mg daily ($P = 0.05$) [(33)].

The linezolid product label[(18, 31, 34)] notes that "*In clinical trials 2.4 % of patients developed a platelet count less than 75% of the LLN/baseline. Thrombocytopenia appears to be dependent on duration of therapy, (generally greater than 2 weeks of treatment).*" *The label notes also, "In cases where the outcome is known, when linezolid was discontinued, the affected hematologic parameters have risen toward pretreatment levels."*

In the trial of Lee et al in S Korea[(24)], seven of 41 Subjects had myelosuppression, including anemia and neutropenia, primarily within the first 5 months, and only one Subject withdrew due to anemia. Six had clinically significant myelosuppression: 5 in 0-4 months and 1 in 4-8 months, with 0 in 8-12 months.

Peripheral and Optic Neuropathy:

The linezolid product label notes these adverse events have been " *…reported in patients, primarily those patients treated for longer than the maximum recommended duration of 28 days. In cases of optic neuropathy that progressed to loss of vision, patients were treated for extended periods beyond the maximum recommended duration. Visual function should be monitored in all patients taking ZVYOX for extended periods (≥3 months) and in all patients reporting new visual symptoms, regardless of length of therapy*[(32)].

In Lee, NEJM, 2012[(24)], the publication's Supplemental Table 3 notes that 21 patients had clinically significant peripheral neuropathy spread over 12 months: 5 in months 0-4, 10 in months 4-8 and 5 in months 8-12 (time of onset not noted for one). Subjects who developed any peripheral neuropathy had their dosing of linezolid interrupted, generally for several weeks, and then resumed at the lower dose of 300 mg/day (C. Barry, personal communication). None of the Subjects withdrew from the study based on peripheral neuropathies. At baseline, patients received visual acuity testing, contrast sensitivity and color vision tests. Seven cases were observed as having potential effects on vision; only two of 38 patients withdrew from study due to optic neuropathy. For clinically significant optic neuropathy, one had this at 0-4 months, 2 at 4-8 months and 3 at months 8-12. Except for the 2 Subjects who withdrew from the study, the others resumed linezolid at the 300 mg dose after a hiatus of several weeks of treatment and completed the study with resolution of their visual acuity changes (C. Barry, personal communication).

In the Schecter California DOH review[29] peripheral neuropathy developed in 5 of 30 patients (no standardized monitoring), but only one withdrew from linezolid therapy. One patient developed visual loss secondary to optic neuropathy after 10 months of linezolid therapy, but vision returned to normal 3-4 weeks after discontinuation.

In Park, 2006[30], two patients of eight in the case series developed optic neuropathy after 8-9 month and had linezolid discontinued; these patients also had peripheral neuropathy. After linezolid treatment was stopped, the optic neuropathy fully resolved after 2-3 months. A total of 4 patients developed peripheral neuropathy at 4, 5, 8, 11 months; in the patients with optic neuropathy who stopped treatment, the peripheral neuropathic symptoms continued or improved only marginally.

In Singla, 2012[30], two of 29 patients treated with linezolid, 600 mg daily over 12 months, stopped the drug because of peripheral neuritis (one patient) and optic neuritis (one patient). The time course of these adverse events was not noted.

2.3. Regimens to be Studied

The regimen included in this study (B-L-Pa) has been selected based on the performance of the regimen in non-clinical pharmacology studies and on the combination of bedaquiline and PA-824 with other drugs in clinical studies NC-001 and NC-003. In addition, improved treatment outcomes in XDR patients with the addition of linezolid to existing therapy provide support for combining linezolid with other drugs that have no pre-existing resistance.

This regimen has the potential to treat drug resistant strains of tuberculosis. This is an oral regimen, removing the need for injectables as part of drug resistant treatment, and is also projected to be markedly less expensive than current XDR-TB therapy. Treatment duration is anticipated to be shorter than current regimens for drug resistant TB, based on findings in mouse models of infection and the fact that all Subjects will be treated with three active drugs against TB for which there is no expected resistance.

The key data supporting the use of the B-L-Pa regimen are described below.

2.3.1. Non-Clinical Studies

In the murine model of TB, addition of bedaquiline to HRZ results in accelerated clearance of *M.tb* [4,5] when compared to HRZ alone. While the combination of bedaquiline and PA-824 in the murine model of TB appeared somewhat antagonistic relative to bedaquiline alone, it was as active as the triple combination of HRZ[10] and in a subsequent study it was more active in the mouse model than HRZ[11]. Thus a novel regimen with bedaquiline plus PA-824 core could be effective in the treatment of MDR-TB by providing two novel drugs for which there is no known pre-existing resistance.

Recent studies of the bactericidal and sterilizing activity of linezolid in a mouse model of *M.tb.* infection have demonstrated that L alone and in combination with bedaquiline and PA-824 causes marked reductions in lung colony forming units (CFUs) from mice following 1-3 months of therapy (Table 7, below). Additionally, all mice treated daily with bedaquiline, PA-824 and linezolid (B-L-Pa) were cured of the infection after 3 months of therapy as evidenced by no *M.tb.* cultured from lungs when mice were sacrificed 3 months after the completion of therapy (Table 8, below). That is in contrast to the 6 months required to cure all mice when treated with the standard of care isoniazid, rifampicin and pyrazinamide (HRZ; note that typically ethambutol is not used in the mouse model of infection). (Data not yet published, E. Nuermberger personal communication).

Table 7: Murine Lung CFU counts during Treatment: Standard Therapy versus novel mono and combination therapies

	Mean lung log_{10} CFU count (± S.D.) at:			
**Regimen**	**Day 0**	**Month 1**	**Month 2**	**Month 3**
Untreated	6.17 ± 0.27	6.50 ± 0.08		
2RHZ/4RH		3.47 ± 0.37	1.59 ± 0.25	0.52 ± 0.36
B		3.24 ± 0.25		
Pa		5.58 ± 0.12		
L		4.97 ± 0.23		
PaL		5.02 ± 0.38	2.13 ± 0.63	
BL		2.82 ± 0.15	1.91 ± 0.60	
BPa		4.21 ± 0.26	1.62 ± 0.19	0.50 ± 0.51
BLPa		3.22 ± 0.57	0.34 ± 0.41	0 ± 0

Table 8. Murine Relapse Data, Standard Therapy versus novel combinations

	Proportion (%) of mice relapsing after treatment for :	
**Regimen**	**2 months**	**3 months**
2RHZ/4RH	ND	13/15 (87%)
BPa	15/15 (100%)	10/15 (67%)
BPaU	14/20* (70%)	**1/14† (7%)**
BLPa	12/15** (80%)	**0/14† (0%)**

*p = 0.02 vs. BPa by 1-tailed Fisher exact test
**p = 0.11 vs. BPa by 1-tailed Fisher exact test
†p≤ 0.001 vs. BPa by 1-tailed Fisher exact test

In conclusion, linezolid increases the sterilizing activity of the bedaquiline-PA-824 (B-Pa) combination; no *M.tb.* could be cultured from the lungs of mice 3 months after cessation of 3 months of treatment with the combination in contrast to *M.tb.* cultured from 13 of 15 mice treated with the standard 2RHZ/4RH regimen over 3 months.

Prior to the use of PA-824 in combination with bedaquiline in clinical study NC-001, a preclinical cardiovascular safety pharmacology study was conducted in unrestrained beagle dogs with both drugs to explore the potential for additive effects on QT prolongation induced by the combination. Results indicate

that administration of 100 mg/kg bedaquiline daily for 7 days causes a small increase in QTc interval by Day 6 in some animals that is not influenced by the addition of 100 mg/kg PA-824 on Day 7. The effect of PA-824 dosing alone on QT interval appeared to be due to discomfort related to the subcutaneous route of administration and not related to the plasma exposure.

2.3.2. Clinical Study NC-001

Study NC-001 was a partially double-blind, randomized, parallel group study in adult male and female subjects with newly diagnosed, uncomplicated, smear-positive, pulmonary TB. A total of 85 subjects met study eligibility criteria and were randomly assigned to one of the following six treatment groups: bedaquiline alone; bedaquiline + pyrazinamide; bedaquiline + PA-824 200 mg; PA-824 200 mg + pyrazinamide; PA-824 200 mg + pyrazinamide, + moxifloxacin; or Rifafour e-275. All study treatments were given once daily for 14 days. Substantial EBA activity was demonstrated across subjects in all arms of the study and the daily reductions in cultured colony counts per mL of sputum are presented in Table 9 below.

Table 9: Summary Statistics for $EBA_{CFU(0-14)}$ Derived Using Bi-Linear Regression, Study NC-001.

Treatment Group	**N**	**Daily Mean (SD) $EBA_{CFU(0-14)}$**
PA-824 + pyrazinamide + moxifloxacin	13	0.23 (0.128)
PA-824 + pyrazinamide	14	0.15 (0.040)
PA-824 + bedaquiline	15[a]	0.11 (0.050)
Bedaquiline alone	14	0.07 (0.068)
Bedaquiline + pyrazinamide	15	0.13 (0.102)
Rifafour e-275	10	0.14 (0.094)

There were no Serious Adverse Events from the study among subjects treated with PA-824 and bedaquiline. Three Subjects in a bedaquiline-containing treatment arm were withdrawn: one subject on a bedaquiline only arm for a Grade 3 ALT and GGT elevation although the elevation occurred prior to the first dose of study medication: one on a bedaquiline plus pyrazinamide (weight banded) arm for a Grade 3 ALT and AST elevation, and one on a PA-824 and bedaquiline arm for to a Grade 3 ALT elevation.

2.3.3. Clinical Study NC-003

Efficacy

In the 14 day EBA study NC-003 two monotherapy and four different combinations of bedaquiline, PA-824, pyrazinamide and clofazimine were evaluated in DS-TB subjects. Fifteen Subjects were randomized into 7 treatment arms: C, Z, B-Pa-Z-C, B-Pa-Z, B-Pa-C, B-Z-C, and HRZE control. This study demonstrated no EBA for the clofazimine monotherapy arm and modest EBA for the pyrazinamide monotherapy arm. However, all of the experimental regimens demonstrated EBA. In general, adding clofazimine to the various agents resulted in either no increase in EBA, or a decrease when compared to a similar regimen that did not include clofazimine. In this study, the experimental regimen with the best EBA was B-Pa-Z which demonstrated a rate of decrease in both logCFU and logTTP that was at least as good as the HRZE control. The daily logCFU results are presented in Table 10. Similar results were found when TTP was used to calculate the bactericidal activity over 14 days (BA(0-14)).

Table 10: NC-003 Efficacy Results: Daily BAlogCFU$_{(0-14)}$

Arm	logCFU
BPaZC	.124
BPaZ	.180
BPaC	.086
BZC	.098
Z	.036
C	-.025
Rifafour®	.152

Safety

Generally, the regimens in this study were well tolerated. Table 11 provides a list of the overall safety findings. The only SAE experienced in the study was in a Subject in the clofazimine monotherapy arm. Otherwise, the rates of treatment emergent AEs (TEAEs) were similar across the treatment arms. One Subject in the B-Pa-Z arm was withdrawn from the study due an adverse event of increased liver function tests (ALT, AST and GGT).

Table 11: NC-003 Safety Data

	BPaZC	BPaZ	BPaC	BZC	Z	C	HRZE	Total
**N**	15	15	15	15	15	15	15	105
**Subjects with:**								
**TEAEs**	11	9	8	10	10	9	8	65
**TEAEs leading to death**:								
**Serious TEAEs**						1		1
**TEAES leading to early withdrawal**		1						1
**TEAEs leading to discontinuation of study drug**		1						1
**Drug-related TEAES**	8	5	7	3	5	6	5	39
**Serious, drug-related TEAEs**								
**Grade III AEs**		2	1	2		1		6
**Grade IV AEs**		1	1					2
**Grade II/IV AEs**		2	1	2		1		6

QT Prolongation

Because bedaquiline and clofazimine are both known to prolong the QT interval, intensive ECG monitoring was included in the study endpoints. The mean change from baseline in QTcB and QTcF tended to be larger at 5 hours than at 10 hours post-dose in the (B-Pa-Z-C) arm and in the (B-Pa-C) arm. No QTcB or QTcF ≥500 ms were reported. An increase from baseline to Visit 5 and subsequent visits of ≥60 ms in QTcB was

reported for 2 Subjects in the (B-Pa-C) arm and for 1 Subject in the clofazimine alone arm. An increase from baseline to Visit 5 and subsequent visits of ≥60 ms in QTcF was reported for 4 Subjects in the (B-Pa-C) arm and for 1 Subject in the clofazimine alone arm. For both QTcB and QTcF, the (B-Pa-Z-C) arm and the (B-Pa-C) arm showed the largest increase from baseline. Clofazimine will not be used in any treatment arms in the current NiX study.

2.4. Overall Benefit/Risk Assessment

The recent report of the long term outcome of patients with XDR-TB treated in S. Africa highlighted the very poor prognosis for patients with this disease. After 60 months of follow up 73% of 107 patients had died and only 11% had a favourable outcome[23]. These patients have infection with *M.tb.* that is resistant to many/most of the available drugs to treat tuberculosis. Patients with XDR-TB have limited treatment options due to their resistance profile, and the drugs that are typically used in Standard of Care have many side effects, some are administered as injectables and have poor treatment outcomes in XDR-TB. This trial provides an opportunity to treat patients with XDR-TB with three active drugs, for which there is no or minimal pre-existing resistance, in a very closely controlled and monitored clinical trial setting. Subjects will be monitored closely and regular reviews of safety and efficacy will be made by the DSMC. While this is an untested combination regimen in patients with XDR-TB, this regimen has the potential to give relapse-free cure of XDR-TB with a simple regimen in a much shorter period of time than currently required by the available drugs used in the best standard of care. Preclinical studies of this regimen in a murine model of infection demonstrated relapse free cure of *M.tb.* in half the time (3 vs 6 months) required by standard HRZ therapy. Clinical studies of linezolid alone and PA-824 and bedaquiline alone and in combination have demonstrated activity against TB infection.

These three drugs have not been used in combination in humans and thus their combined toxicity profile is not known. There is limited experience with both B and Pa to date and thus their safety profile is emerging. The greatest risks of key concern for subjects in this trial from linezolid are from the adverse events of myelosuppression and peripheral and optic neuropathy. Subjects will be closely monitored with full blood counts, vision examinations, and screening for peripheral neuropathy. The investigator may interrupt dosing of either linezolid or linezolid with PA-824 and bedaquiline if adverse events of concern develop, and a resumption of the drugs, with linezolid at the same or at a lower dose, may be made cautiously. Subjects will be under close surveillance for hepatotoxicity, as that risk for PA-824 and bedaquiline is not yet well characterized. Other adverse events of special concern are seizures or other neurologic events. Seizures have been reported in patients taking linezolid, seizures have been noted in animal toxicology studies of PA-824 at higher doses, and one unexplained seizure was noted in a patient taking M-Pa-Z in Study NC-002.

Overall the benefit-risk balance justifies evaluating the B-L-Pa regimen in this study, with the cautious surveillance in place, to treat patients with XDR-TB who have few options for a successful outcome.

3. TRIAL RATIONALE AND OBJECTIVES

3.1. Trial Rationale

This trial will provide a regimen containing 3 drugs against which there is no expected *M.Tb.* resistance in the community for patients with limited treatment options while simultaneously gathering important efficacy and safety data on a regimen that could potentially treat all strains of *M.tb*. Data from previous trials shows that the combination of B-Pa is well tolerated and has the potential to shorten treatment in Subjects who are suceptable to all drugs. The addition of linezolid will ensure each Subject receives at least 3 drugs active against their TB strain.

3.2. Dose Rationale

3.2.1. Bedaquiline

Bedaquiline will be administered as the dose regimen currently approved by the United States Food and Drug Administration for treatment of patients with MDR-TB: 400mg once daily for Days 1-14 followed by 200mg three times per week for the remainder of treatment.

3.2.2. PA-824

PA-824 has demonstrated good microbicidal activity at the 200mg daily dose as monotherapy in studies PA-824-CL-007 and PA-824-CL-010, in combination with either bedaquiline or pyrazinamide over 14 days in the EBA Study NC-001-(B-M-Pa-Z) and in combination with either bedaquiline and/or pyrazinamide and/or clofazimine over 14 days in the EBA Study NC-003-(B-C-Pa-Z). In the EBA Study PA-824-CL-010 the 100mg dose demonstrated similar microbicidal activity to the 150 and the 200mg daily dose over 14 days. The Phase 2 trial NC-002-(M-Pa-Z) evaluated this regimen at doses of PA-824 of both 100 mg and 200 mg relative to the HRZE control. In this trial the efficacy results were similar between Subjects treated with 100 mg/day and 200 mg/day of PA-824 in the regimen, although for the primary endpoint, reduction in colony forming units of *M.tb.* from sputum, only the 200 mg/day dose group was statistically significantly better than the group randomized to standard HRZE therapy. Safety was also similar between the groups, although the 200 mg/day group had more grade 2 adverse events than either the 100 mg/day group or the HRZE control group. Consequently, in an upcoming Phase 3 trial for this regimen, the PaMZ regimen will be evaluated at both the doses of 100 mg/day and 200 mg/day. However because sterilizing relapse-free cure of TB in patients with XDR-TB may ultimately require a regimen with higher drug exposures, the 200mg dose has been chosen for this study.

3.2.3. Linezolid

The standard dose of linezolid for a multitude of indications is 400mg or 600 mg BID. Doses of linezolid used in reported observational trials and case series range from 300 mg to 1200 mg per day over periods of up to 20 months of treatment. While the development of adverse events is generally higher with higher doses, the adverse events often ameliorate with a reduction of the dose or discontinuation of drug for several weeks and then reintroduction at a lower dose. No controlled trials have clearly identified differences in anti-TB effect across a range of doses. This trial will start all subjects on 600 mg bid of linezolid, the approved dose to treat bacterial infections for up to 28 days. If adverse events develop, the investigator will be able to interrupt dosing or to reduce the dose level to either 600 mg qd, 300 mg bid or 300 mg qd in an effort to allow this patient population wth high mortality on standard care to continue to benefit from the study drug regimen.

3.3. Trial Objectives

To evaluate the efficacy, safety, tolerability and pharmacokinetics of bedaquiline plus PA-824 plus linezolid after 6 months of treatment (with an option to treat for 9 months in Subjects who are still culture positive at month 4) in Subjects with either pulmonary XDR tuberculosis, treatment intolerant or non-responsive multi-drug resistant tuberculosis (MDR-TB).

4. TRIAL DESIGN

4.1. Summary

Up to 200 male and female Subjects aged 14 and over with confirmed sputum positive for *M.tb.* in culture (any sample positive between Screening and Week 4) pulmonary XDR-TB, or with pulmonary MDR-TB with a documented intolerability or non-response to the best treatment available for 6 months or more will be enrolled.

All Subjects will have up to a maximum of 9 days for screening, receive 6 months of treatment, and have follow-up visits performed 1 and 2 month after treatment completetion and every 3 months after study

treatment completion for 24 months. If a Subject is still culture positive by their Month 4 visit, they may have treatment extended to 9 months (with 24 months of Follow Up) or be withdrawn from the study.

Subjects who withdraw after ≤14 days of IMP should attend an Early Withdrawal visit. Subjects who withdraw after ≥15 days of IMP should return for an Early Withdrawal visit and follow-up visits at 3, 12 and 24 months after their last dose of IMP to check for survival, SAEs and resolution of TB symptoms.

Subjects will receive:

- B-L-Pa for the duration of treatment.

Figure 1: Trial Schematic

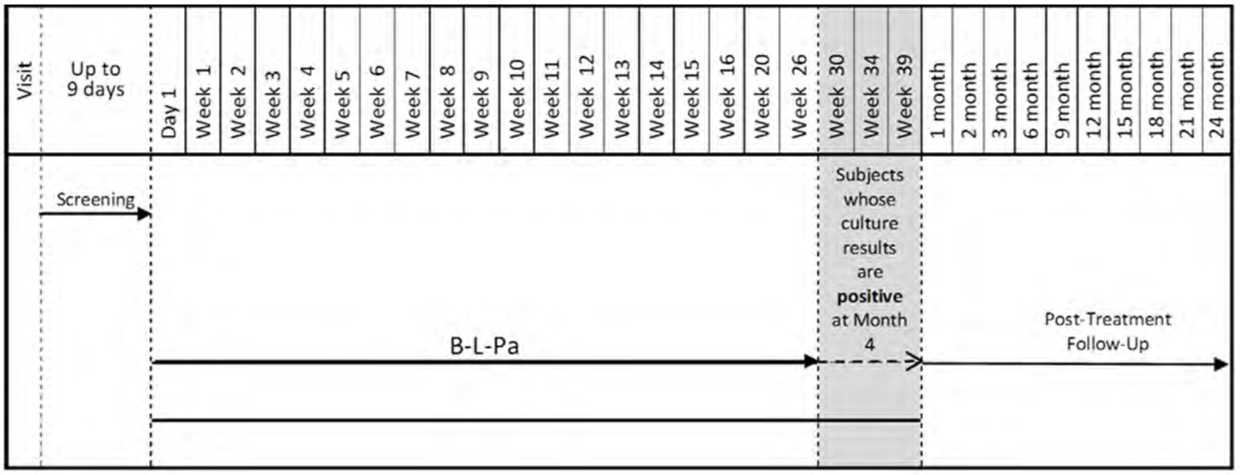

4.2. Trial Endpoints

4.2.1. Primary Endpoint

Incidence of bacteriologic failure or relapse or clinical failure through follow up until 24 months after the end of treatment.

Abbreviated Definitions (full definitions will be described in the Statistical Analysis Plan (SAP)):

- Bacteriologic failure: During the treatment period, failure to attain culture conversion to negative.
- Bacteriologic relapse: During the follow-up period, failure to maintain culture conversion to negative status in culture, with culture conversion to positive status with a of *Mycobacterium tuberculosis* (*M.tb.*) strain that is genetically identical to the infecting strain at baseline.
- Clinical failure: A change from protocol-specified TB treatment due to treatment failure, retreatment for TB during follow up, or TB-related death.

Note:

- Culture conversion requires at least 2 consecutive culture negative/positive samples at least 21 days apart.
- Subjects who are documented at a visit as unable to produce sputum and who are clinically considered to be responding well to treatment will be considered to be culture negative at that visit.

4.2.2. Secondary Endpoints

4.2.2.1. Efficacy:

- Time to sputum culture conversion to negative status through the treatment period.
- If liquid culture in the MGIT platform is used, the rate of change in time to sputum culture positivity (TTP) over time in the Mycobacterial Growth Indicator Tube (MGIT) system in sputum, represented by the model-fitted log(TTP) results as calculated by the regression of the observed log(TTP) results over time.
- Proportion of subjects with sputum culture conversion to negative status at 4, 6, 8, 12, 16 and 26 or 39 weeks.
- Linezolid dosing (actual) and efficacy will be explored.
- Change from baseline TB symptoms.
- Change from baseline in Patient Reported Health Status

4.2.2.2. Safety and Tolerability:

- All cause mortality.
- Incidence of Treatment Emergent Adverse Events (TEAEs) will be presented by severity, (DMID Toxicity Grade), drug relatedness and seriousness, leading to early withdrawal and leading to death.
- Quantitative and qualitative clinical laboratory result measurements, including observed and change from baseline.
- Quantitative and qualitative measurement of ECG results, including observed and change from baseline
- Descriptive statistics of ophthalmology slit lamp examination data (age related eye disease study 2 [AREDS2] lens opacity classification and grading). Categorical data for lens opacity will be summarized in a frequency table for the right and left eye, respectively, including change from baseline.
- Changes in ophthalmic exam for visual acuity and color vision, including observed and change from baseline.
- Changes noted in peripheral neuropathy signs and symptoms, including observed and change from baseline.

These data will be presented as descriptive analyses, and no inferential tests will be carried out.

4.2.2.3. Pharmacokinetics:

Pharmacokinetics will consist of two separate schedules:

- All Subjects- Pre-dose sampling at weeks 2, 8 and 16 to measure C_{trough} levels of bedaquiline, bedaquiline metabolite M2, Linezolid and PA-824.
- PK Sub-study Subjects- in addition to the C_{trough} samples, there will be intensive PK sampling at Week 16 at pre-dose, 0.5, 1, 2, 4, 8 12, 12.5, 13, 14, 16, 20 and 24 hours after dosing in a sub-group of 30 Subjects across selected sites.

For the PK sub-study samples, the following PK parameters will be estimated from the individual (per Subject) PK plasma concentrations: Minimum observed PK plasma concentration (C_{min}), maximum observed PK plasma concentration(C_{max}), time to reach C_{max} obtained without interpolation (T_{max}), area under the PK plasma concentration time (t) curve from zero to the last quantifiable PK plasma concentration prior to the subsequent dose, using the linear trapezoidal rule ($AUC_{(0-t)}$), area under the PK plasma concentration time (t) curve from zero to 24 hours ($AUC_{(0-24)}$). Oral apparent clearance (CL/F) by non-compartment model. These will be derived for each analyte. In addition, for analyte linezolid at BID dose, the AUC_{0-12}, C_{max}, C_{min}, CL/F and $t_{1/2}$ will be calculated based on dose interval 0-12 hrs.

4.2.2.4. Exploratory

- Evaluate whether any of the secondary endpoints predicts relapse free cure.
- Sub-analysis of populations by HIV staus and CD4 count.
- Correlation of Time over mitochondrial protein synthesis inhibition (MPS50) with linezolid toxicity, (the MPS50 value will be an assumed value from literature).

4.2.2.5. General Mycobacteriology

Early Morning and Coached Spot Sputum Samples will be obtained at all scheduled visits, except the Screening Visit when only a Coached Spot Sputum Sample will be collected. Both (if feasible) sputum samples (Coached Spot and Early Morning) collected will be used for identification for the presence or absence of *M.tb.* in culture and if liquid culture in the MGIT platform is used, for TTP in liquid medium.

The following mycobacteriology assays will be carried out according to procedures described in the Laboratory Manual:

Table 12: General Mycobacteriology

Sample	Type	Assessments
**Screening**	Coached Spot Sputum Sample	• Direct microscopy for acid-fast bacilli (AFB);<br>• Gene Xpert, Hain Assay MTBDRplus or an alternative molecular or antigen-based test to confirm *M.tb.*
**Baseline (Day1)**	Early Morning and Coached Spot Sputum Samples	• Culture for presence or absence of *M.tb.*;<br>• If liquid culture in the MGIT platform is used, TTP in liquid medium;<br>The following will be processed at a central lab:<br>• MIC: bedaquiline, linezolid and PA-824;<br>• Drug susceptibility testing in liquid culture for rifampicin, isoniazid, streptomycin, ethambutol and second line TB drugs including fluoroquinolones and injectables;<br>• Speciation of the infecting organisim by molecular / antigen test ;<br>• Extraction of bacterial (*M.tb.*) DNA for molecular strain typing;<br>• DNA extraction for pncA sequencing.
**All Visits Post Baseline**	Early Morning and Coached Spot Sputum Samples	• Culture for presence or absence of *M.tb.;*<br>• If liquid culture in the MGIT platform is used, TTP in liquid medium.
**Any culture positive sample at or following end of treatment**	Early Morning or Coached Spot Sputum	The following will be processed at a central lab:<br>• MIC: bedaquiline, linezolid and PA-824;<br>• Drug susceptibility testing in liquid culture for rifampicin, isoniazid, streptomycin, ethambutol and

Sample	Type	Assessments
	Sample	second line TB drugs including fluoroquinolones and injectables;<br>• Speciation of the infecting organisim by molecular / antigen test;<br>• Extraction of bacterial (*M.tb.)* DNA for molecular strain typing.

- The extracted *M.tb.* DNA and isolates will be stored for potential further work to validate new assay tools for a maximum of 5 years after trial closure.
- Both the Early Morning and the Coached Spot sputum samples should be cultured for the mycobacteriology analyses when feasible. If only one sample is cultured, the other should be kept as a back-up sample for use when the other sample is contaminated.
- MIC isolates and DNA extracted from sputum for pncA analysis can be batched at the end of the study or when a Subject completes/relapses.
- If the Subject was treated with study medication for less than 14 days, the mycobacteriology testing will be performed on the baseline sample isolate only.

4.3. Trial Population

4.3.1. Inclusion Criteria

1. Provide written, informed consent prior to all trial-related procedures (if under 18, include consent of legal guardian).
2. Body weight of ≥30 kg or greater (in light clothing and no shoes).
3. Willingness and ability to attend scheduled follow-up visits and undergo study assessments
4. Provide consent to HIV testing (if an HIV test was performed within 1 month prior to trial start, it should not be repeated as long as documentation can be provided [ELISA and/or Western Blot]).
5. Male or female, aged 14 years or above.
6. Subjects with one of the following pulmonary TB conditions:
    a. XDR-TB documented by culture positive (for *M.tb.*) results (with resistance to isoniazid, rifamycins, a fluoroquinolone and an injectable within 3 months prior to screening);
    b. MDR-TB documented by culture positive results (for *M.tb.*) within 3 months prior to screening with documented non-response to treatment with the best available regimen for 6 months or more prior to enrollment who in the opinion of the Investigator have been adherent to treatment and will be adherent to study regimen;
    c. MDR-TB documented by culture positive (for *M.tb.*) results within 3 months prior to screening who are unable to continue second line drug regimen due to a documented intolerance to:
        i. PAS, ethionamide, aminoglycosides or fluoroquinolones;
        ii. Current treatment not listed above that renders subject eligible for the study in the Investigator's opinion.
7. Chest X-Ray picture (taken within a year prior to screening) consistent with pulmonary TB in the opinion of the Investigator.
8. 8. Be of non-childbearing potential or using effective methods of birth control, as defined below:

    <u>Non-childbearing potential:</u>

    a. Subject - not heterosexually active or practices sexual abstinence; or
    b. Female Subject/sexual partner - bilateral oophorectomy, bilateral tubal ligation and/or hysterectomy or has been postmenopausal with a history of no menses for at least 12 consecutive months; or

c. Male Subject/sexual partner - vasectomised or has had a bilateral orchidectomy minimally three months prior to Screening.

Effective birth control methods:

A double contraceptive method should be used as follows:

a. Double barrier method which can include any 2 of the following: a male condom, diaphragm, cervical cap, or female condom (male and female condoms should not be used together); or
b. Barrier method (one of the above) combined with hormone-based contraceptives or an intra-uterine device for the female Subject/partner;
c. and are willing to continue practicing birth control methods throughout treatment and for 6 months (both male and female Subjects) after the last dose of study medication or discontinuation from study medication in case of premature discontinuation.

Note: Hormone based contraception alone may not be reliable when taking IMP; therefore, hormone based contraceptives alone cannot be used by female Subjects or female partners of male Subjects to prevent pregnancy.

4.3.2. Exclusion Criteria

Medical History

1. Any condition in the Investigator's opinion (i.e., an unstable disease such as uncontrolled diabetes or cardiomyopathy, extra-pulmonary TB requiring extended treatment), where participation in the trial would compromise the well-being of Subject or prevent, limit or confound protocol specified assessments.
2. In the judgment of the Investigator, the patient is not expected to survive for more than 12 weeks.
3. Karnofsky score < 50 within 30 days prior to entry.
4. History of allergy or known hypersensitivity to any of the trial Investigational Medicinal Products or related substances.
5. For HIV infected Subjects having a CD4+ count <50 cells/µL;
   a. Currently treated with or will need to initiate antiretroviral therapy (ART) which is not compatible with the allowed ARTs and is not considered an appropriate candidate for switching to a regimen of ARVs which is allowed as follows:
      i. Nevirapine based regimen consisting of nevirapine in combination with any NRTIs;
      ii. Lopinavir/ritonavir (Aluvia™) based regimen consisting of lopinavir/ritonavir (Aluvia™) in combination with any NRTIs;
      iii. In the case of resistance or intolerance to the above two regimens, a triple nucleosidase reverse trascriptase inhibitors (NRTI) based regimen consisting of zidovudine, lamivudine and abacavir may be used with caution;
   b. Cannot ensure a 2 week interval between commencing IMP and the start of ART, if not already on ARTs.
6. Having participated in other clinical studies with dosing of investigational agents within 8 weeks prior to trial start or currently enrolled in an investigational study that includes treatment with medicinal agents. Subjects who are participating in observational studies or who are in a follow up period of a trial that included drug therapy may be considered for inclusion.
7. Significant cardiac arrhythmia requiring medication.
8. Subjects with the following at Screening:

   a. QTcF interval on ECG >500 msec. Subjects with QTcF > 450 must be discussed with the sponsor medical monitor before enrolment.
   b. History of additional risk factors for Torsade de Pointes, (e.g., heart failure, hypokalemia, family history of Long QT Syndrome);
   c. Clinically significant ventricular arrhythmias;
   d. Subjects with other cardiac abnormalities that may place them at risk of arrhythmias must be discussed with the sponsor medical monitor before enrollment. Such abnormalities include: Evidence of ventricular pre-excitation (e.g., Wolff Parkinson White syndrome); Electrocardiographic evidence of complete or clinically significant incomplete left bundle branch block or right bundle branch block; Evidence of second or third degree heart block; Intraventricular conduction delay with QRS duration more than 120 ms.
9. Females who have a positive pregnancy test at Screening or already known to be pregnant, breast-feeding, or planning to conceive a child during the study or within 6 months of cessation of treatment. Males planning to conceive a child during the study or within 6 months of cessation of treatment.
10. A peripheral neuropathy of Grade 3 or 4, according to DMID (Appendix 2). Or, subjects with a Grade 1 or 2 neuropathy which is likely to progress/worsen over the course of the study, in the opinion of the Investigator.

<u>Specific Treatments</u>

11. Concomitant use of Monoamine Oxidase Inhibitors (MAOIs) or prior use within 2 weeks of treatment assignment.
12. Concommitant use of serotonergic antidepressants or prior use within 3 days of treatment assignment if Investigator foresees potential risks for serotonin syndrome when combined with linezolid.
13. Concomitant use of any drug known to prolong QTc interval (including, but not limited to, amiodarone, bepridil, chloroquine, chlorpromazine, cisapride, cyclobenzaprine, clarithromycin, disopyramide, dofetilide, domperidone, droperidol, erythromycin, fluoroquinolones, halofantrine, haloperidol, ibutilide, levomethadyl, mesoridazine, methadone, pentamidine, pimozide, procainamide, quinidine, sotalol, sparfloxacin, thioridazine).
14. Concomitant use of any drug known to induce myelosuppresion.
15. Subjects may have previously been treated for DS/MDR-TB provided that treatment is/was discontinued at least 3 days prior to treatment assignment.

<u>Based on Laboratory Abnormalities</u>

16. Subjects with the following toxicities at Screening (labs may be repeated) as defined by the enhanced Division of Microbiology and Infectious Disease (DMID) adult toxicity table (November 2007):
    a. serum potassium less than the lower limit of normal for the laboratory;
    b. Hemoglobin level of < 8.0 g/dL;
    c. Platelet count < 80,000/mm^3;
    d. Absolute neutrophil count (ANC) < 1000/ mm^3;
    e. aspartate aminotransferase (AST) ≥5.0 x ULN. Subjects with AST ≥ 3.0 x ULN must be discussed with the sponsor medical monitor before enrolment;
    f. alanine aminotransferase (ALT) ≥5.0 x ULN. Subjects with ALT ≥ 3.0 x ULN must be discussed with the sponsor medical monitor before enrolment;
    g. total bilirubin grade 3 or greater (>2.0 x ULN, or >1.50 x ULN when accompanied by any increase in other liver function test);
    h. Serum creatinine level less than 2 times upper limit of normal.

4.4. Treatment Plan: Schedule of Assessments

The trial consists of three periods, as follows:

- Screening (Up to 9 days Prior to Treatment);
- Treatment Period (Day 1 to Week 26 OR Day 1 to Week 39);
- Follow-Up Period (1 month to 24 months post Treatment End).

Refer to:

- Study Flow Chart (Section 1.2) for the overview of the timing of all procedures and laboratory samples to be done at each visit.
- Trial Procedures (Section 6) for details regarding specific procedures or laboratory tests.

Visit Window:

- Week 1 through Week 11: ± 3 days
- Weeks 12 through End of Treatment (Week 26 or 39): ± 7 days
- Post-Treatment Follow-Up Visits (1-3 months): ± 2 weeks

Note: Subjects on 6 months of treatment should complete treatment within 8 months of treatment assignment (a total halt of up to 60 days if on 6 months)while subjects on 9 months of treatment should complete treatment within 12 months of treatment assignment (a total halt of 90 days if on 9 months of treatment) .

4.4.1. Screening (Up to 9 Days Prior to Treatment)

4.4.1.1. Screening

The screening visit may occur over a number of days up to 9 days prior to treatment assignment, (i.e. all screening procedures do not have to be performed on the same day).

The following information will be collected and procedures performed:

- Written Informed Consent (Main study; HIV testing if applicable);
- Demographic Data;
- Medical and Treatment History;
- Eligibility Assessment;
- Karnofsky Score;
- HIV test and CD4 count:
    - o If an HIV test was performed within 1 month prior to trial start, it need not be repeated as long as documentation can be provided [ELISA and/or Western Blot]).
    - o Subjects may be on current antiretroviral therapy (ART) or commence ART once on the study provided there is at least a 2 week interval between commencing IMP and the start of ART;
- Chest X-Ray;
- Serum or Urine Pregnancy Test, (women of child bearing potential only, whether they are sexually active or not);
- TB Symptoms Profile;
- Patient Reported Health Status;
- Ophthalmology- Slit Lamp Examination;
- Ophthalmic Examination (Opthalmologic Medical History, Visual Acuity, and Color Assessment);
- Vital Signs, including weight (should be done prior to any lab assessments);
- Single 12-lead ECG (the ECG should be done before vital signs and any lab assessments);
- Full Physical Examination including height;

- Laboratory Safety Assessments;
- Coached Spot Sputum Sample collection;
- Concomitant Medication(s)/Other Treatment(s);
- Adverse Events;
- Peripheral Neuropathy Assessment.

4.4.2. Treatment Period (Day 1 to Week 26 or Week 39)

4.4.2.1. Day 1

The following information will be collected and procedures performed pre-dosing:

- Eligibility Assessment;
- Serum or Urine Pregnancy Test, (women of child bearing potential only, whether they are sexually active or not);
- Vital Signs, including weight (should be done before any labs);
- Single 12-lead ECG (the ECG should be done before any vital signs and any labs);
- Full Physical Examination;
- Laboratory Safety Assessments;
- Treatment Assignment;
- Early Morning Sputum Collection;
- Coached Spot Sputum Sample Collection;
- Concomitant Medication(s)/Other Treatment(s);
- Adverse Events;
- Investigational Medicinal Product (IMP) Administration.

4.4.2.2. Week 1

The following information will be collected and procedures performed pre-dosing:

- Vital Signs, including weight (should be done before any labs);
- Single 12-lead ECG (the ECG should be done before any vital signs and any labs);
- Limited Physical Examination;
- Laboratory Safety Asssessments;
- Early Morning Sputum Collection;
- Coached Spot Sputum Sample Collection;
- Concomitant Medication(s)/Other Treatment(s);
- Adverse Events;
- Investigational Medicinal Product (IMP) Administration.

4.4.2.3. Week 2

The following information will be collected and procedures performed pre-dosing:

- Vital Signs, including weight (should be done before any labs);
- Limited Physical Examination;
- Laboratory Safety Asssessments;
- Pre-dose Pharmacokinetic Sampling (All Subjects);
- Early Morning Sputum Collection;
- Coached Spot Sputum Sample Collection;
- Concomitant Medication(s)/Other Treatment(s);
- Adverse Events;
- Investigational Medicinal Product (IMP) Administration.

4.4.2.4. Weeks 3, 5, 7, 9, 10, 11, 13, 14 and 15

- Complete Blood Count/Full Blood Count (performed pre-dosing).

4.4.2.5. Week 4

The following information will be collected and procedures performed pre-dosing:

- Vital Signs, including weight (should be done before any labs);
- Single 12-lead ECG (the ECG should be done before any vital signs or labs);
- Limited Physical Examination;
- Laboratory Safety Asssessments;Early Morning Sputum Collection;
- Coached Spot Sputum Sample Collection;
- Concomitant Medication(s)/Other Treatment(s);
- Ophthalmic Examination (Visual Acuity and Color Assessment);
- Adverse Events;
- Peripheral Neuropathy Assessment;
- Investigational Medicinal Product (IMP) Administration.

4.4.2.6. Week 6

The following information will be collected and procedures performed pre-dosing:

- Vital Signs, including weight (should be done before any labs);
- Limited Physical Examination;
- Laboratory Safety Asssessments;
- Early Morning Sputum Collection;
- Coached Spot Sputum Sample Collection;
- Concomitant Medication(s)/Other Treatment(s);
- Adverse Events;
- Investigational Medicinal Product (IMP) Administration.

4.4.2.7. Week 8

The following information will be collected and procedures performed pre-dosing:

- Serum or Urine Pregnancy Test, (women of child bearing potential only, whether they are sexually active or not);
- TB Symptoms Profile/Patient Reported Health Status;
- Vital Signs, including weight (should be done before any labs);
- Single 12-lead ECG (the ECG should be done before any vital signs or labs);
- Limited Physical Examination;
- Laboratory Safety Asssessments;
- Early Morning Sputum Collection;
- Coached Spot Sputum Sample Collection;
- Concomitant Medication(s)/Other Treatment(s);
- Adverse Events;
- Peripheral Neuropathy Assessment.
- Ophthalmic Examination (Visual Acuity and Color Assessment);
- Pre-dose Pharmacokinetic Sampling (All Subjects);
- Investigational Medicinal Product (IMP) Administration.

4.4.2.8. Week 12

The following information will be collected and procedures performed pre-dosing:

- Vital Signs, including weight (should be done before any labs);
- Limited Physical Examination;
- Laboratory Safety Assessments
- Ophthalmic Examination (Visual Acuity and Color Assessment);
- Early Morning Sputum Collection;
- Coached Spot Sputum Sample Collection;
- Concomitant Medication(s)/Other Treatment(s);
- Adverse Events;
- Peripheral Neuropathy Assessment;
- Investigational Medicinal Product (IMP) Administration.

4.4.2.9 Week 16 (Week 30 when applicable)

The following information will be collected and procedures performed pre-dosing:

- Vital Signs, including weight (should be done before any labs);
- Single 12-lead ECG (the ECG should be done before any vital signs or labs);
- Limited Physical Examination;
- Laboratory Safety Asssessments;
- Ophthalmic Examination (Visual Acuity and Color Assessment)
- Early Morning Sputum Collection;
- Coached Spot Sputum Sample Collection;
- Concomitant Medication(s)/Other Treatment(s);
- Adverse Events;
- Peripheral Neuropathy Assessment;
- Investigational Medicinal Product (IMP) Administration;
- Pre-Dose Pharmacokinetic Sampling (all subjects, this should be done prior to dosing with study medication)
- Intensive PK Sub-Study (30 across all participating sites) at pre-dose, 0.5, 1, 2, 4, 8, 12. 12.5, 13, 14, 16, 20 and 24 hours after dosing.

4.4.2.10 Week 20 (Week 34 when applicable)

The following information will be collected and procedures performed pre-dosing:

- Vital Signs, including weight (should be done before labs);
- Limited Physical Examination;
- Laboratory Safety Asssessments;
- Ophthalmic Examination (Visual Acuity and Color Assessment);
- Early Morning Sputum Collection;
- Coached Spot Sputum Sample Collection;
- Concomitant Medication(s)/Other Treatment(s);
- Adverse Events;
- Peripheral Neuropathy Assessment;
- Investigational Medicinal Product (IMP) Administration.

4.4.2.11 Week 26 (Week 39 when applicable)

The following information will be collected and procedures performed when subject completes *end of treatment visit*:

- Serum or Urine Pregnancy Test, (women of child bearing potential only, whether they are sexually active or not);
- TB Symptoms Profile/Patient Reported Health Status;
- Vital Signs, including weight (should be done before any labs);
- Single 12-lead ECG (the ECG should be done before any vital signs or labs);
- Full Physical Examination;
- Laboratory Safety Asssessments;
- Early Morning Sputum Collection;
- Coached Spot Sputum Sample Collection;
- Ophthalmology Slit Lamp Examination;
- Ophthalmic Examination (Visual Acuity and Color Assessment);
- Concomitant Medication(s)/Other Treatment(s);
- Adverse Events;
- Peripheral Neuropathy Assessment;
- Investigational Medicinal Product (IMP) Administration.

When subject is scheduled to receive 9 months of treatment, the following assessments should be done at the week 26 visit:

- Vital Signs, including weight (should be done before any labs);
- Single 12-lead ECG (the ECG should be done before any vital signs or labs);
- Limited Physical Examination;
- Laboratory Safety Asssessments;
- Early Morning Sputum Collection;
- Coached Spot Sputum Sample Collection;
- Ophthalmic Examination (Visual Acuity and Color Assessment);
- Concomitant Medication(s)/Other Treatment(s);
- Adverse Events;
- Peripheral Neuropathy Assessment;
- Investigational Medicinal Product (IMP) Administration.

4.4.2.12 Early Withdrawal

In case of Early Withdrawal during the treatment period of the study (prior to completing 26 or 39 weeks of treatment as applicable), all efforts shall be made to complete the Early Withdrawal assessments. At the Early Withdrawal visit, the following information will be collected and procedures performed:

- Serum or Urine Pregnancy Test (for women of child bearing potential only, whether they are sexually active or not);
- TB Symptoms Profile/Patient Reported Health Status;
- Vital Signs, including weight (should be done before any labs);
- Single 12-lead ECG (the ECG should be done before any vital signs and labs);
- Full Physical Examination;
- Laboratory Safety Asssessments;
- Early Morning Sputum Collection;
- Coached Spot Sputum Sample Collection;
- Ophthalmology Slit Lamp Examination (if received $\geq$ 12 weeks of study treatment);
- Ophthalmic Examination (Visual Acuity and Color Assessment)

- Concomitant Medication(s)/Other Treatment(s);
- Adverse Events;
- Peripheral Neuropathy Assessment.

Follow-Up required for Early Withdrawals based on Treatment Duration

Treatment Duration at EWD visit	Ophthalmology Examination at EWD	Ophthalmology Examination Visit 3 months after EWD Visit	Month 12	Month 24
≤ 14 days	Not required	Not required	Not Required	Not Required
15 days to ≤ 12 weeks	Not required	Required	Required	Required
> 12 weeks	Required	Required	Required, if not already performed.	Required

Upon Early Withdrawal of IMP, All Subjects will be referred to a unit specializing in treatment of XDR-TB.

4.4.3 Follow-Up Period

4.4.3.1 1 Month Post-Treatment

- Early Morning Sputum Collection;
- Coached Spot Sputum Sample Collection.

4.4.3.2 2 Months Post-Treatment

- Early Morning Sputum Collection;
- Coached Spot Sputum Sample Collection;

4.4.3.3 3 Months Post-Treatment or Withdrawal

The following information will be collected and procedures performed:

- Vital Signs, including weight;
- Ophthalmology Slit Lamp Examination*;
- Ophthalmic Examination (Visual Acuity and Color Assessment);
- Limited Physical Examination;
- Early Morning Sputum Collection;
- Coached Spot Sputum Sample Collection;
- Concomitant Medication(s)/Other Treatment(s);
- Peripheral Neuropathy Assessment;
- Adverse Events.

* - Opthalmology Slit Lamp Exam is required only for subjects who withdraw after 15 days or more of treatment.

4.4.3.4 Months 6, 12 and 24 Post-Treatment

The following information will be collected and procedures performed:

- TB Symptoms Profile/ Patient Reported Health Status (only at Months 12 and 24)*
- Vital Signs, including weight;
- Ophthalmic Examination (Visual Acuity and Color Assessment)

- Limited Physical Examination;
- Early Morning Sputum Collection;
- Coached Spot Sputum Sample Collection;
- Concomitant Medication(s)/Other Treatment(s);
- Peripheral Neuropathy Assessment;
- Adverse Events.*

* - For any Subjects who withdraw early (during the treatment period after more than 14 days treatment or follow-up), the Month 12 and 24 visits will be only to collect Serious Adverse Event (SAE) information (including verification of survival) and patient reported TB outcome information and may be telephonic, a home or a site visit.

4.4.3.5 Months 9, 15, 18 and 21 Post-Treatment

- Vital Signs, including weight;
- Limited Physical Examination;
- Early Morning Sputum Collection;
- Coached Spot Sputum Sample Collection;
- Concomitant Medication(s)/Other Treatment(s);
- Adverse Events.

4.4.3.6 Unscheduled Visits

Any visit which is conducted in addition to those required by the Trial Flow Chart should be considered unscheduled regardless of the reason for the visit. The assessments which are undertaken as part of an unscheduled visit should be as clinically indicated.

If both spot sputum samples obtained at the Month 4, End of Treatment (Week 26/39), End of follow-up Period or Early Withdrawal visits are contaminated, the subject should return for an unscheduled visit(s) to give additional samples or to document the Subject is not able to produce sputum.

In order to be able to define a Subject's primary outcome status it may be necessary in certain situations to contact a Subject and request they visit the site in order to collect additional Spot Sputum samples at Unscheduled Visits, as follows:

- <u>To be assessed on sputum culture results from:</u>
  - End of Treatment Period (Week 26/39);
  - End of Follow-up Period (Month 24);
  - Early Withdrawal (if applicable).

- <u>Confirm whether the Subject has:</u>
  - Two sequential negative sputum culture results; or
  - Two sequential positive sputum culture results; or
  - Has been unable to produce sputum after documentation of two negative sputum cultures with no intervening positive and are clinically asymptomatic.

- If they **do not** fall into one of these categories, keep collecting Spot Sputum samples x 2 (one Early Morning and one Spot at the research site under the coaching and observation of the trial staff) at a minimum of 21 days or more apart until they fall into one of the above categories.

If in any of the above scenarios the Investigator is unsure of the outcome, the Investigator must contact the Sponsor Medical Monitor to discuss and agree on how the patient is to be handled.

4.5 Treatment Discontinuation and Subject Withdrawal

Any Subject for whom the Investigator decides to temporarily discontinue their IMP is to contact the Sponsor Medical Monitor and, if/when applicable, can be restarted on IMP as described in section 4.6

A Subject should immediately discontinue treatment and be prematurely withdrawn from the trial for the following reasons:

- Withdrawal of informed consent;
- Lost to Follow-Up;
- Investigator considers that for safety reasons (including specific toxicities as described in section 7.3), it is in the best interest of the Subject he/she be withdrawn;
- Pregnancy;
- Full regimen or linezolid halted > 35 days consecutively;
- Full regimen or linezolid halted cumulatively greater than 60 days cumulatively for Subjects receiving 6 months of treatment and 90 days cumulatively for Subjects receiving 9 months of treatment;
- At the specific request of the Sponsor or termination of the study by the Sponsor;
- Subject who, in the opinion of the Investigator or Sponsor, fails to comply with the Protocol.

If at any time the investigator is unsure whether or not to withdraw the Subject, the Investigator is to contact the Sponsor Medical Monitor and discuss and agree on how the patient is to be handled.Subjects who withdraw from the trial after having received IMP will not be replaced.

Upon discontinuation of IMP, Subjects will be referred to a unit specializing in the treatment of XDR.

Subjects who withdraw early should have an early withdrawal visit and additional follow-up visits according to timing of withdrawal as outlined in section 4.4.2.9

Early Withdrawal due to TB

Ultimately it is the investigator’s decision whether a Subject requires Early Withdrawal from the trial due to a concern that the Subject has symptomatic worsening TB and/or bacteriological failure/relapse.

Early Withdrawal is usually not indicated by a single positive culture. Should a Subject have a single positive culture result after being negative, the investigator is to evaluate whether the Subject has signs and symptoms suggestive of active inadequately treated TB and whether it is in the Subjects best interest that he/she be withdrawn. Prior to Early Withdrawal of a Subject due to TB, the investigator must discuss the Subject with the sponsor medical monitor, unless the investigator cannot contact the sponsor medical monitor and considers that Early withdrawal must occur immediately due to immediate safety concerns with respect to the Subject.

If the investigator decides to withdraw a Subject due to TB, additional sputum samples may need to be collected in order to ensure the Subject’s outcome status may be determined (section 4.4.3.4).

All Early Withdrawal Subjects who are confirmed sputum positive (two sequential sputum positive cultures) and/or have symptomatic TB will require further TB treatment. These Subjects will be referred to a unit that specializes in treatment of XDR-TB.

4.6 Temporary Dose Interruptions and Modifications

All dose interruption and modifications should be discussed with the Sponsor Medical Monitor prior to implementation.

For Subjects experiencing suspected drug related toxicities due to linezolid, the daily dose of linezolid may be reduced or may be temporarily halted for up to 35 consecutive days. Generally, if temporarily halted, it should be re-instituted at a lower dose. Generally a step down in dose should proceed from 600 mg bid to

600 mg daily or 300 mg bid and then to 300 mg daily. Linezolid dose may be re-started at the same dose at Investigator discretion.

For Subjects experiencing suspected drug related toxicities due to other drugs in the regimen (B-Pa), the full regimen may be halted for up to 35 consecutive days.

Subjects on 6 months of treatment should complete treatment within 8 months of treatment assignment (a total halt of up to 60 days if on 6 months) while subjects on 9 months of treatment should complete treatment within 12 months of treatment assignment (a total halt of 90 days if on 9 months of treatment) .

At no time should the Subject be treated a single agent.

4.7 Stopping Rules

There are no trial specific stopping rules.

The trial or parts of the trial can be stopped by the Sponsor on advice from the Data Safety and Monitoring Committee (DSMC) after their review of applicable trial data. In addition, the Sponsor has the right to stop the trial or a specific Investigational Site at any time, although this should only occur after consultation between involved parties. Should this occur, the local and central Ethics Committee/Institutional review Board (EC/IRB) and Regulatory Authorities will be informed. Should the Trial/Investigational Site be closed prematurely, all trial materials (except documentation that has to remain stored at the Investigational Site) will be returned to the Sponsor or vendor. The Investigator will retain all other documents until notification given by the Sponsor for destruction. Subjects currently on treatment will receive an appropriate regimen and all Subjects will be referred to a unit specializing in the treatment of XDR-TB.

4.8 Subject Progress Definitions

All efforts should be made to contact subjects that do not attend scheduled trial visits. The investigator should attempt to follow up subjects that miss scheduled trial visits unless the subject has withdrawn consent.

If a subject fails to attend a scheduled trial visit, the site will attempt to contact the subject as soon as possible by phone (if applicable) and, if necessary, a home visit will be made, to encourage attendance at the earliest opportunity.

All Subjects will be categorized with two of the following definitions and this should be clearly documented on the eCRF.

4.8.1 Enrolment

- **Screening Failure**

Subjects from whom informed consent is obtained and is documented in writing (that is, subject signs an informed consent form), but are not assigned treatment.

- **Enrolled**

Subjects from whom informed consent is obtained and is documented in writing (that is, subject signs an informed consent form), and who are assigned treatment.

4.8.2 Completed Trial

Subjects who are assigned treatment and complete Treatment and Follow-Up.

4.8.3 Withdrawn

- **During Treatment-** Subjects who are assigned treatment and withdraw/are withdrawn from the trial prior to completion of treatment visits.

During Follow-up- Subjects who are assigned and complete treatment, however withdraw/are withdrawn from the trial prior to completion of their follow-up visits.

4.9 Restrictions

4.9.1 Prior and Concomitant Medications and Other Treatments

Concomitant medications should be kept to a minimum during the trial. However, if concomitant medications are considered to be necessary for the Subject's welfare and are unlikely to interfere with the IMP, they may be given at the discretion of the investigator. For any concomitant medications given as a treatment for a new condition or a worsening of an existing condition occurring after signing of the informed consent form, the condition must be documented on the Adverse Event pages of the electronic Case Report Form (eCRF).

The prescribing information for all concomitant medication should be consulted and reviewed carefully. The determinations listed in the respective contraindicated, warning, and precaution sections must be respected in order to prevent any potentially serious and/or life-threatening drug interactions.

The following concomitant medications are prohhibited during the treatment period to avoid possible drug interactions with the IMP:

- Medicinal products used to treat pulmonary TB: including but not limited to gatifloxacin, amikacin, cycloserine, rifabutin, kanamycin, para-aminosalicylic acid, rifapentine, thioacetazone, capreomycin, quinolones, thioamides, and metronidazole.
- Concomitant use of Monoamine Oxidase Inhibitors (MAOIs). (e.g. phenelzine, isocarboxazid)
- Concomitant use of any drug known to prolong QTc interval (including but not limited to amiodarone, bepridil, chloroquine, chlorpromazine, cisapride, cyclobenzaprine, clarithromycin, disopyramide dofetilide, domperidone, droperidol, erythromycin, halofantrine, haloperidol, ibutilide, levomethadyl, mesoridazine, methadone, pentamidine, pimozide, procainamide, quinidine, sotalol, sparfloxacin, thioridazine).
    - Treatment with fluoroquinolones (as they are known prolong QTc), are strongly discouraged in the trial. They should only be used to treat intercurrent non-TB infections and if the benefit of treatment outweighs the risk of prolonged QTc.
- Concomitant use of any drug known to induce myelosuppression.

Concommitant use of serotonergic antidepressants should be avoided if possible as subjects on these agents and linezolid are at risk for serotonin syndrome.

Caution should be used in treating diabetic patients receiving insulin or oral hypoglycemic agents as cases have been reported of hypoglycemic reactions when patients on these agents have been treated with linezolid.

4.9.1.1 Recommendations for Concomitant use of Anti-Malarials

The following treatments for malaria are recommended for concomitant use with the IMP, should it be necessary:

- Proguanil/atovaquone or
- Artesunate plus sulfadoxine-pyrimethamine

These recommendations are based on the potential for QT prolongation by bedaquiline and many anti-malarials. Due to the extended half-life of bedaquiline commencing anti-malarial treatment containing drugs that could prolong the QT interval, shortly after discontinuing bedaquiline, is not recommended.

4.9.1.2 Antiretroviral Therapy

- Patients taking bedaquiline should avoid efavirenz due to drug-drug interactions with bedaquiline, and thus Subjects taking antiretroviral therapy during the study should only take one of the

following regimens: Nevirapine based regimen consisting of nevirapine in combination with any NRTIs;

- Lopinavir/ritonavir (Aluvia™) based regimen consisting of lopinavir/ritonavir (Aluvia™) in combination with any NRTIs;
- If subject has intolerability or resistance to one of the above regimens, a triple nucleoside reverse transcriptase inhibitor (NRTI) based regimen consisting of zidovudine, lamivudine, and abacavir may be used.

Subjects who are commencing ART may be entered onto the study provided there is at least a 2 week interval between commencing IMP and the start of ART.

4.9.1.3 Other Restrictions

Large quantities of foods or beverages with high tyramine content should be avoided while taking linezolid. Quantities of tyramine consumed should be less than 100mg per meal. Foods high in tyramine content include those that may have undergone protein changes by aging, fermentation, pickling, or smoking to improve flavour, such as aged cheeses (0 to 15 mg tyramine per ounce); fermented or air-dryed meats (0.1 to 8 mg tyramine per ounce); sauerkraut (8 mg tyramine per 8 ounces); soy sauce (5mg tyramine per 1 teaspoon); tap beers (4 mg tyramine per 12 ounces); red wines (0 to 6mg tyramine per 8 ounces). The tyramine content of any protein-rich food may be increased if stored for long periods or improperly refrigerated.

5 INVESTIGATIONAL MEDICINAL PRODUCT

5.1 Trial Treatments

Subjects will receive oral dosing as described below.

- Bedaquiline Days 1-14: 400mg once daily (4 x bedaquiline 100 mg tablets),
- Bedaquiline Weeks 3-26/39*: 200mg three times per week (2 x bedaquiline 100 mg tablets); **plus**
- Linezolid 600mg twice daily day 1 through week 26 or 39* (2 x scored linezolid 600 mg tablets); **plus**
- PA-824 200mg once daily Day 1 through week 26 or 39* (1 x PA-824 200 mg tablet).

Subjects will receive a minimum of 6 months of treatment, (with 24 months of Follow Up). If a Subject is still culture positive by their Month 4 visit, they may have treatment extended to 9 months (with 24 months of Follow Up), or be withdrawn from the study.

5.2 Method of Assigning Subjects to Study Treatment

Eligible Subjects who have given written, informed consent will be enrolled onto the trial during Screening and will be identified by a study generated Subject identification code for anonymity (Subject number).

Once the screening results are available and subject is eligible to participate, the site will request their pharmacist/registered dispenser to assign an IMP treatment number to the Subject. The site pharmacist/registered dispenser will assign the the next available applicable treatment number, in a sequential basis starting from the lowest unused treatment number.

The process of assigning a treatment number will be fully documented.

5.3 IMP Administration

The Subject should be instructed to:

- Take IMP orally twice daily (B-L-Pa in the AM, L in the PM) with food for 26 weeks, preferably at the same time every day, with a glass of water (approximately 240ml);

- Subjects should take IMP with a meal (generally allow the Subjects a window of 30 minutes before to 30 minutes after a meal);
- When Subjects are hospitalized or return for clinic visits, they will be dosed on site.

5.4 Subject Compliance

During site clinic visits or hospitalisation, the IMP will be administered by the Investigator/designated site personnel. During the study, sites will be responsible for ensuring Subjects are taking the IMP correctly and are fully trained on how IMP is to be taken. When possible, Subjects will be checked for IMP compliance by the Investigators or trial personnel/National TB Treatment Program personnel via the hand-and-mouth procedure (both the hand and mouth of the Subject will be checked to ensure that the Subject has swallowed the IMP).

5.5 Blinding and Procedures for Breaking the Blind

This is an open label study. There is no need for blinding or procedures to break the blind.

5.6 IMP Packaging and Labelling

The complete formulations of the bedaquiline and PA-824 are found in the applicable Investigator Brochures [4,5,15]. The complete formulations of linezolid are found in the applicable Package Inserts [18,31,34].

5.13.1 Packaging

IMP will be supplied as:

- Bedaquiline 100mg Tablets;
- Scored Linezolid 600mg Tablets;
- PA-824 200mg Tablets;

Subjects will receive oral dosing as described below.

- Bedaquiline Days 1-14: 400mg once daily (4 x bedaquiline 100 mg tablets),
- Bedaquiline Weeks 3-26/39*: 200mg three times per week (2 x bedaquiline 100 mg tablets); **plus**
- Linezolid 600mg twice daily Day 1 through week 26 or 39* (2 x scored linezolid 600 mg tablets); **plus**
- PA-824 200mg once daily Day 1 through week 26 or 39* (1 x PA-824 200 mg tablet)**.**

Subjects will receive a minimum of 6 months of treatment. If a Subject is still culture positive by their Month 4 visit, they may have treatment extended to 9 months or be withdrawn from the study.

5.13.2 Labelling

The test product will be packaged in blister cards with bulk card supplies available for the B-Pa weeks 1-2, B-Pa weeks 3-End of Treatment and Linezolid. The outer packaging of each bulk pack will be labeled with, at a minimum, the following information:

- Name, address and telephone number of the Sponsor
- Name of medication, dosage, quantity and method of administration
- Reference/Lot Number
- Protocol number, visit numbers and space for completion of name of Investigator and site number
- The statement “For Clinical Trial Use Only”
- Storage conditions
- Expiry date
- The statement “Keep out of reach of children”

The inner packaging on each weekly treatment card will be labeled with, at a minimum, the following information:

- Name, address and telephone number of the Sponsor
- Name of medication, dosage, quantity and method of administration
- Reference/Lot Number
- Protocol number and space for completion of name of Investigator, site and visit number
- Directions for use
- Subject Number and Initials
- The statement "For Clinical Trial Use Only"
- Storage conditions
- Expiry date
- The statement "Keep out of reach of children"

5.6 Storage

All study medication will be kept securely stored by the site pharmacist/registered dispenser in a secured area with limited access to designated site personnel only.

Test product containing treatment arms will be stored in the supplied containers (thereby protected from light and moisture), between 15 to 30 degrees Celsius.

5.7 Dispensing and Accountability

The site pharmacist/ delegated dispenser will be responsible for dispensing the IMP. Accurate accountability records will be kept by the site to assure that the IMP will not be dispensed to any person who is not a Subject under the terms and conditions set forth in this protocol (i.e. delivery to site, inventory at site, use by Subject, destruction, etc.) The Investigator/designee will immediately inform the Sponsor of any quality issues arising with respect to the trial medication. The Sponsor will take whatever action is required should such a situation arise.

The Investigator undertakes to use the trial medication only as indicated in this protocol.

5.8 Returns and Destruction

Upon completion or termination of the trial, all unused and/or partially used IMPs must be returned to Sponsor (or designated vendor) who will arrange for destruction after final accountability has been confirmed. If no supplies remain, this fact will be indicated in the drug accountability section of the final report.

6 TRIAL VARIABLES AND PROCEDURES

6.1 Demographic and Background Variables and Procedures

The following demographic and background variables will be collected at the time points described in the trial flow chart:

- Visit Dates.
- Subject Disposition.
- Written Informed Consent (including HIV when applicable).
- Eligibility criteria.
- Demographic data: Date of birth, race and gender.
- Medical and treatment history.
- Screening Coached Spot Sputum Sample:
    - Direct microscopy for acid-fast bacilli.
    - Gene Xpert, Hain Assay MTBDRplus or an alternative molecular or antigen-based test to confirm *M.tb.*

- Serum or Urine pregnancy test: women of child-bearing potential only, whether they are sexually active or not.
- Serology: HIV and CD4 count.
  - Approval for this to be performed will be obtained from Subjects in the written informed consent process. If an HIV test was performed within 1 month prior to trial start, it should not be repeated as long as documentation can be provided (ELISA and/or Western Blot).
  - Prior to HIV testing and on receipt of the results, Subjects will be counselled on HIV by trained counsellors if they have indicated as such on the HIV consent form. If requested by the Subject, HIV counselling provided to the Subject by the study site should be clearly documented in the Subject's medical records/source. Subjects have the right to decline to know or receive their HIV test results. This decision should be clearly documented in the Subject's medical records/source.
- Karnofsky Score (Appendix 4).
- Chest X-Ray: A Chest X-Ray picture will be obtained from the clinic appointed radiology department or from the Subject if it has been taken within the previous 1 year. The Investigator is responsible for review and analysis for Subject inclusion.
- Method of Birth Control: Male and Female Subjects and their partners.
- IMP Details /Actual Dosing

6.2 Efficacy Variables and Procedures

Two Spot Sputum Samples are collected, (one Early Morning and one spot at the research site under the coaching and observation of the trial staff). The Mycobacteriology sampling methodology and requirements will be described in a separate document, the Laboratory Manual, which will be provided prior to the trial start.

The following analyses will be performed:

- Culture result;
- If liquid culture in the MGIT platform is used, TTP in liquid medium.

Using these observed variables the following derived variables will be assessed for evaluation of the efficacy endpoints:

- Bacteriologic failure/relapse;
- If liquid culture in the MGIT platform is used, the rate of change in log time of sputum culture positivity (TTP) over time in liquid medium;
- Time to Sputum Culture Conversion;
- Number of subjects with Sputum Culture Conversion.

Every effort is to be made to collect sputum samples. However, in general, the inability to produce sputum is treated as being equivalent to having a negative culture (favourable) result. A subject who never achieves culture negative status due to inability to produce sputum, but has completed 12/24 months follow-up and is without clinical or biological evidence of relapse, will be considered to have a favorable outcome.

TB Symptoms Profile:

- The TB Symptoms Profile (Appendix 7) will record subjects' ratings of the severity of common TB symptoms.

Patient Reported Health Status Variables and Procedures:

- The Patient Reported Health Status variables will be collected at the time points described in the trial flow chart.

- Patient Reported Health Status will be collected using the EQ-5D-5L Health Questionnaire (Appendix 5). This descriptive system consists of five health-related quality of life dimensions, each of which will be recorded using five levels of severity.
- Methodology: The Patient Reported Health Status methodology and requirements will be described in a separate document/guideline which will be provided prior to the trial start.

6.3 Safety and Tolerability Variables and Procedures

The following safety and tolerability variables will be collected at the time points described in the trial flow chart and assessed for evaluation of the safety endpoints:

- Laboratory parameters. The Safety Laboratory sampling methodology and requirements will be described in a separate document, the Laboratory Manual, which will be provided prior to the trial start. The following analyses will be performed:
    - Hematology/Complete Blood Count/Full Blod Count (hemoglobin, hematocrit, red blood cell count, white blood cell count with differential, platelet count),
        - At Weeks 3, 5, 7, 9, 10, 11, 13, 14, 15, Complete Blood Count/Full Blood Count including red and white cell counts and indices and platelet count ***only*;** no clinical chemistry or urinalysis at those visits.
        - Coagulation *at Screening only* (activated partial thromboplastin time (APTT), prothrombin time (PT), international normalized ratio (INR));
    - Clinical Chemistry (albumin, serum urea, creatinine, direct, indirect and total bilirubin, uric acid, total protein, alkaline phosphatase, aspartate aminotransferase (AST), alanine aminotransferase (ALT), gamma-glutamyl transferase (GGT), lactic dehydrogenase (LDH), total amylase, lipase, phosphate, sodium, potassium, calcium (corrected for albumin), chloride, magnesium, random/fasting glucose, bicarbonate/CO_2, creatine phosphokinase (CPK and CK-MB).
    - Urinalysis (pH, specific gravity, protein, glucose, micro-albumin, ketones, bilirubin, creatinine, nitrite, sodium, urobilinogen, blood, leukocytes). Microscopy will be completed as follow up to abnormal urinalysis per discretion of Investigator.
- 12-lead Electrocardiogram (ECG):
    - Investigator Assessment: Normal, Abnormal;
    - Methodology:
        - ECGs should be recorded prior to any lab draws and administration of IMP;
        - Subjects should be lying down (recumbent) for at least 5 minutes prior to each 12-lead ECG evaluation;
        - ECGs are to be recorded for 10 seconds;
        - All ECG to be performed in single;
        - For each Subject, the ECGs should, to every extent possible, be collected at approximately the same time of the day and in the same fed/fast state (e.g. 4 hours after lunch).

- Vital signs:
    - Systolic and diastolic blood pressure (mmHg) to be measured supine (after 5 minutes of rest) using an appropriately sized cuff, and using the same type of sphygmomanometer, if possible by the same observer, at each relevant visit.
    - Heart rate (bpm).
    - Axillary body temperature (°C).
- Physical Examination:
    - Height is measured at screening only.
    - Full (complete) and Limited (pulmonary, cardiovascular and abdominal) examinations will be performed and any clinically significant findings will be recorded.
    - Weight (kg) (in light clothing and with no shoes).

  - Using the observed variables weight and height, calculated body mass index (BMI) will be derived.

- Ophthalmology Slit Lamp Examination. To be done by an Ophthalmologist trained on AREDS2 assessment . The ophthalmology slit lamp methodology and requirements will be described in a separate document, the Ophthalmology Guideline, which will be provided prior to the trial start. The following analyses will be performed: AREDS2 opacity typing and grading.
- Ophthalmic Examination. The ophthalmic examinations can peformed by any trained study staff. The screening exams must be done by the trained study staff AND an Ophthalmologist. Methodology and requirements will be detailed in a separate Ophthalmic Examination Manual.
  - Ophthalmology History (Screening only);
  - Visual Acuity Test – Corrected. Near and Distance Vision;
  - Color Vision Assessment.
- Adverse Events.
- Brief Peripheral Neuropathy Screen (Appendix 6) will record ratings.
- Concomitant Medication/Other Treatments.

6.4 Pharmacokinetic Variables and Procedures

Pharmacokinetics will consist of two separate schedules:

- All Subjects- Pre-dose sampling at weeks 2, 8 and 16 to measure C_{trough} levels of B, B metabolite M2, linezolid and Pa.
- PK Sub-Study Subjects- in addition to the C_{trough} samples, there will be intensive PK sampling at week 16 at pre-dose, 0.5, 1, 2, 4, 8 12, 12.5, 13, 14, 16, 20 and 24 hours after dosing in a sub-group of 30 Subjects across selected sites.

Pharmacokinetic Analysis:
For the C_{trough} samples, only descriptive statistics will be prepared (average C_{trough}) derived for each analyte.

For the PK Sub-Study samples, the following PK parameters will be estimated from the individual (per Subject) PK plasma concentrations: minimum observed PK plasma concentration (C_{min}), maximum observed PK plasma concentration(C_{max}), time to reach C_{max} obtained without interpolation (T_{max}), area under the PK plasma concentration time (t) curve from zero to the last quantifiable PK plasma concentration prior to the subsequent dose, using the linear trapezoidal rule ($AUC_{(0-t)}$), area under the PK plasma concentration time (t) curve from zero to 24 hours ($AUC_{(0-24)}$). These will be derived for each analyte. In addition, for analyte linezolid at BID dose, the AUC_{0-12}, C_{max}, C_{min}, CL/F and $t_{1/2}$ will be calculated based on dose interval 0-12 hrs.

6.5 Mycobacteriology Characterization Variables and Procedures

The following Mycobacterial Characterization variables will be collected:

Samples from:

- Day 1 (baseline) Early Morning and coached spot sputum samples (or Screening to Week 4 if the baseline is contaminated or negative);
- Any culture positive sample at or following end of treatment.

The *M.tb.* isolates will be processed at a central lab for:

- MIC against bedaquiline, PA-824 and linezolid;
- Drug Susceptibility Testing in liquid culture for rifampicin, isoniazid, streptomycin, ethambutol and second line TB drugs including fluoroquinolones, and injectables;
- Speciation of the infecting organisms by molecular or antigen based test;
- Extraction of bacterial DNA (*M.tb.*) for molecular strain typing;
- DNA for pncA sequencing.

All Day 1 (baseline) *M.tb.* isolates and isolates from positive cultures to be stored at the local microbiology laboratory (or a central laboratory) until trial closure for the applicable study tests. The extracted *M.tb.* DNA

and isolates will be stored for potential further work to validate new assay tools for a maximum of 5 years after trial closure.

The Mycobacteriology sampling methodology and requirements will be described in a separate document, the Laboratory Manual, which will be provided prior to the trial start.

7 ADVERSE EVENTS

The Investigators are responsible for eliciting adverse events by observing the Subject and recording adverse events observed by him/her or reported by the Subject during the trial.

7.1 Definitions

7.1.1 Adverse Event (AE)

Any untoward medical occurrence in a patient or clinical investigation Subject administered a pharmaceutical product and which does not necessarily have a causal relationship with this treatment. An adverse event can therefore be any unfavorable and unintended sign (including an abnormal laboratory finding), symptom, or disease temporally associated with the use of a medicinal product, whether or not related to the medicinal product.

7.1.2 Serious Adverse Event (SAE)

Any untoward medical occurrence that at any dose:

- results in death;
- is life threatening (any event in which the Subject was at risk of death at the time of the event; it does not refer to an event, which hypothetically might have caused death if it were more severe);
- requires inpatient hospitalization or prolongation of existing hospitalization;
- results in persistent or significant disability/incapacity;
- is a congenital anomaly/birth defect; or
- is a medically important event.

Note: Medical and scientific judgment should be exercised in deciding which is a medically important event that may not be immediately life-threatening or result in death or hospitalization, but may jeopardize the Subject or may require medical or surgical intervention to prevent one of the outcomes listed above. Examples of such events are intensive treatment in an emergency room or at home for allergic bronchospasm, blood dyscrasias or convulsions that do not result in hospitalization, or the development of drug dependency or drug abuse. A “suspected transmission of infectious agent by a medicinal product” is also considered a serious adverse event under the SAE criterion “Other medically important condition”.

7.1.3 Unlisted (Unexpected) Adverse Event

An adverse reaction, the nature or severity of which is not consistent with the applicable product information (e.g., Investigator’s Brochure for an unapproved investigational product or package insert/summary of product characteristics for an approved product).

7.1.4 Life Threatening

Any event in which the Subject was at risk of death at the time of the event; it does not refer to an event, which hypothetically might have caused death if it were more severe.

7.1.5 Associated with the Use of the Drug

An adverse event is considered associated with the use of the drug (Adverse Drug Reaction) if the attribution is possible, probable or very likely.

7.1.6 Attribution/Causality

The definitions for rating attribution/causality will be as described in Table 13.

Table 13: Adverse Events Attribution/Causality Ratings

Relatedness Rating	Definition
Not Related	An adverse event, which is not related to the use of the drug.
Unlikely	An adverse event for which an alternative explanation is more likely, e.g., concomitant drug(s) or concomitant disease(s), and/or the relationship in time suggests that a causal relationship is unlikely.
Possible	An adverse event, which might be due to the use of the drug. An alternative explanation, e.g., concomitant drug(s) or concomitant disease(s), is inconclusive. The relationship in time is reasonable; therefore the causal relationship cannot be excluded.
Probable	An adverse event, which might be due to the use of the drug. The relationship in time is suggestive, e.g., confirmed by dechallenge. An alternative explanation is less likely, e.g., concomitant drug(s) or concomitant disease(s).
Certain	An adverse event, which is listed as a possible adverse reaction and cannot be reasonably explained by an alternative explanation, e.g., concomitant drug(s) or concomitant disease(s).

7.1.7 Severity

Severity rating is to be made per the DMID Adult Toxicity Table (Appendix 2). For abnormalities **NOT found** elsewhere in the Toxicity Tables, the DMID scale described in Table 14 below is to be used to estimate grade of severity:

Table 14: Adverse Event Severity Ratings

Grade	Severity Rating	Definition
**GRADE 1**	Mild	Transient or mild discomfort (< 48 hours); no medical intervention/therapy required.
**GRADE 2**	Moderate	Mild to moderate limitation in activity - some assistance may be needed; no or minimal medical intervention/therapy required.
**GRADE 3**	Severe	Marked limitation in activity, some assistance usually required; medical intervention/therapy required, hospitalizations possible.
**GRADE 4**	Potentially Life-Threatening	Extreme limitation in activity, significant assistance required; significant medical intervention/therapy required, hospitalization or hospice care probable.

7.1.8 Other AE Definitions

The following definitions will be used for Adverse Event Reporting:

Action Taken with IMP

- IMP unchanged
- IMP interrupted
- IMP stopped
- Not applicable (Follow-up period)

Other Action Taken

- None
- Medication given
- Hospitalisation or prolongation of hospitalisation
- Therapeutic or diagnostic procedure

Outcome

- Resolved
- Improved
- Unchanged
- Worse
- Fatal
- Unknown

Occurrence

- Once
- Intermittent
- Continuous

7.2 Reporting

7.2.1 Adverse Event (AE)

Adverse events will be collected by the Investigator from the time a Subject signs the Informed Consent Form through to their Month 24 follow-up visit. The exception is early withdrawal Subjects who will only have SAEs collected from their time of early withdrawal to their Month 24 follow-up visit.

Any AE (serious or non-serious) observed by the Investigator or reported by the Subject will be recorded on the Adverse Event Case Report Form. The Investigator will review each AE and assess its relationship to drug treatment based on all available information at the time of the completion of the case report form. The following information will be recorded for each Adverse Event reported (definitions section 7.1):

- Diagnosis of the AE, if possible. In the case where an overall diagnosis cannot be made, each specific sign and/or symptom will be recorded as individual AEs;
- Date of onset;
- Stop Date (duration) if applicable;
- Severity;
- Action Taken with IMP;
- Other Action Taken;
- Outcome;
- Relationship to IMP;
- Occurrence;
- Seriousness.

7.2.2 Serious Adverse Event (SAE)

Any AE that occurs which is serious must be reported by the Investigator to the study monitor and copied to the Sponsor Medical Monitor within 24 hours of the site first being aware of the SAE, whether or not the serious event is deemed associated with the use of the drug.

In addition, the Investigator will provide a detailed, signed, written, and complete SAE report form that addresses the Investigator's estimates of the attribution/causality of the AE to the study drug and the seriousness of the AE in question to the study monitor and medical monitor within 24 hours of becoming aware of the SAE.

The study monitor will confirm receipt of the SAE Form with the Investigator and review the initial information on the SAE for diagnosis, consistency and completeness of data.

For submission of updated or additional information on a previously reported SAE, the Investigator will provide the study monitor and medical monitor with a newly completed Serious Adverse Event Form, designated as a follow-up report. This will be submitted to the study monitor and medical monitor within 24 hours of the Investigator receiving the information.

The study monitor will query for additional information from the Investigator, if necessary, to complete the profile of the SAE reported.

The Sponsor/Investigator/designee will inform Regulatory Authorities and/or IEC/IRB of all SAEs in accordance with local requirements and ICH guidelines for GCP.

The Sponsor/designee will forward Safety Notification letters to the Investigator for submission to the IEC/IRB.

7.2.3 Follow up of Adverse Events

All AEs will be followed until:

- satisfactory clinical resolution or stabilization; or
- until the end of the follow-up period; and
- until all queries on these AEs have been resolved.

Certain long-term AEs cannot be followed until resolution within the setting of this protocol. In these cases follow-up will be the responsibility of the treating physician. However, this will have to be agreed upon with the Sponsor.

7.2.4 Post-Trial Adverse Events

Any new SAEs reported by the Subject to the Investigator that occur after the last scheduled contact, and are determined by the Investigator to be possible, probable or certainly related to the use of the IMP, will be reported to the Sponsor, IEC/IRB and regulatory authorities on an expedited basis as required in accordance with local requirements and ICH guidelines for GCP.

7.2.5 Clinical Laboratory Adverse Events

Changes in the results of the Clinical Laboratory assessment results which the Investigator feels are clinically significant will be reported as adverse events. It is the Investigators' responsibility to review the results of all laboratory tests as they become available. This review must be documented by the Investigators' dated signature on the laboratory report. For each abnormal laboratory test result, the Investigator needs to ascertain and document if this is a clinically significant change from baseline for that individual Subject. This determination, however, does not necessarily need to be made the first time an abnormal value is observed. The Investigator may repeat the laboratory test or request additional tests to verify the results of the original laboratory tests. If this laboratory value is determined by the Investigator to be a clinically significant change from baseline for that Subject, it is considered to be an adverse event.

7.2.6 Disease under Study

Symptoms of the disease under study (Pulmonary Tuberculosis) experienced by the Subject while on the study will be assessed by the Investigator. If the symptom has:

- worsened while the Subject is in the study; and
- the Investigator assesses it as clinically significant;

it will be recorded as an adverse event.

If there is:

- no change; and
- the Investigator assesses the symptom as due to the Subject's TB; and
- not clinically significant;

it will not be recorded as an AE and this will be noted in the Subject's source documentation.

All TB related symptoms that meet SAE criteria will be recorded and reported as a SAE.

7.2.7 Overdose

Overdose of IMP experienced by the Subject while on the study, will be assessed by the Investigator to determine whether the overdose led to an Adverse Event, including if the taking of the suspect medicine led to suicidal intention and subsequent overdose of the suspect medicine, or other medication. In this case it will be recorded as an adverse event. If it does not lead to an Adverse Event it will not be recorded as an AE and this will be noted in the Subject's source documentation.

7.2.8 Drug Interaction

If the Investigator becomes aware that the Subject has experienced a drug interaction which has resulted in an adverse event, it will be recorded as an adverse event.

7.2.9 Pregnancy

The Investigator will immediately notify the Sponsor of any pregnancy that is discovered during IMP administration or which started during IMP administration. Pregnancy forms will be completed for all pregnancies reported during the clinical trial, as defined below. In addition, the Investigator will report to the Sponsor follow-up information regarding the outcome of the pregnancy, including perinatal and neonatal outcome. Infants will be followed for 6 months.

All women of childbearing potential will be instructed to contact the Investigator immediately if they suspect they might be pregnant (for example, missed or late menses) for the following time-periods:

- During the trial;
- Within 6 months after last dose of IMP.

If pregnancy is suspected while the Subject is receiving IMP, the IMP will be withheld immediately until the result of the pregnancy test is known. If pregnancy is confirmed, the IMP will be permanently discontinued in an appropriate manner and the Subject withdrawn from the trial. Protocol-required procedures for trial discontinuation and follow-up will be performed unless contraindicated by the pregnancy.Should the female partner of a male Subject become pregnant during the study or in the 6 months after the completion of IMP and the Investigator becomes aware that this situation has occurred, consent will be requested from the female partner for collection of information on her pregnancy history and for information on the current pregnancy and birth.

Pregnancy reporting will **follow the same time lines and reporting structures as for a SAE** (see above). SAE reporting will also occur if the pregnancy outcome is a congenital anomaly. This will follow the reporting procedures described above for SAE reporting plus an additional clinical report compiled by the applicable company.

7.3 Monitoring and Safety for Specific Toxicities

AEs still ongoing at the end of treatment in the trial will be followed until satisfactory clinical resolution or stabilization or until the end of the follow-up period and until all queries on these AEs have been resolved. Grade 3 and grade 4 laboratory abnormalities and laboratory abnormalities considered clinically significant should be followed until satisfactory resolution or stabilization.

Note: For Grade 3 or 4 laboratory toxicities, Subjects should have a confirmatory measurement within 48 hours where possible. The recomendations for managing Subjects below assumes the laboratory abnormalities of concern have been confirmed.

Monitoring for specific toxicities is based upon target organs as defined in preclinical toxicity studies (Investigator's Brochures [4,5,15] and Package Inserts [3,18,31,34]).

7.3.1 ALT, AST and Alkaline Phosphatase elevations:

The Investigator should refer to Appendix 8 – Liver Toxicity Management to appropriately manage the Subject for clinically significant elevations of AST, ALT or Alkaline Phosphatase.

7.3.2 Amylase elevation

Grade 3 (> 2.0 to ≤ 5.0 x ULN):

Contact sponsor Medical Monitor to review. Further testing such as pancreatic amylase and trypsin-like immunoreactivity should be considered after consultation with the Sponsor Medical Monitor.

Grade 4 (> 5.1 x ULN):

Contact sponsor Medical monitor to review. Investigator should consider subjects with **confirmed Grade 4** elevations of total amylase for temporary or permanent discontinuation from the full regimen.

7.3.3 Lipase Elevation

Grade 3 (> 2.0 to ≤ 5.0 x ULN) or Grade 4 (> 5.0 x ULN):

Contact Sponsor medical Monitor to review. Investigator should consider subjects with **confirmed Grade 3 or 4** elevations of lipase for temporary or permanent discontinuation from the full regimen.

7.3.4 Musculo-skeletal System and Cardiac Muscle

Myalgia

Grade 2 (muscle tenderness at site other than sites of injection and/or venipuncture or with moderate impairment of activity) or Grade 3 (severe muscle tenderness with marked impairment of activity) or Grade 4 (frank myonecrosis):

Subjects with Grade 2 signs and symptoms should be followed closely. Subjects with Grade 3 or 4 signs and symptoms should be discussed with the Sponsor Medical Monitor and to consider withholding study medication.

Subjects having **Grade 3 (3.1 to 6 x ULN) or Grade 4 (> 6 x ULN) elevation in CK-MB subunit** (with a confirmatory measure 7 days after the initial lab)- The Investigator should consider discontinuing the full regimen and discuss with the Sponsor Medical Monitor.

7.3.5 Cardiac Rhythm Disturbances

Cardiac rhythm disturbances that are **Grade 3 (recurrent, persistent, symptomatic arrhythmia requiring treatment)** or **Grade 4 (unstable dysrhythmia requiring treatment):**

Subjects should be monitored closely. The Investigator should consider discontinuing the full regimen with the Sponsor Medical Monitor.

QTc prolongation

- If QTcF is equal to or greater than 500 msec, the ECG should be repeated and serum electrolytes should be evaluated. If the second ECG also has a QTcF of $\geq$ 500 msec, the full regimen should be withheld and the Sponsor Medical Monitor consulted.

- New left bundle branch block (LBBB) or Mobitz type 2 or complete heart block. Recordings with artifacts that interfere with the interpretation of the ECG should be repeated to confirm the findings.If the finding is from the centralized ECG machine reading the result is to be checked and confirmed by the Investigator. If this is confirmed by the Investigator, dosing is to be withheld until the reading has been confirmed by the central cardiologist and the Subject is to be treated per the Investigator's clinical judgment. If it is confirmed by the central cardiologist, the Subject is to be withdrawn from the full regimen.

7.3.6 Myelosuppression

Investigator should consider withholding linezolid for subjects with:

- Neutropenia with an absolute neutrophil count below 750 (confirmed by repeat);
- Thrombocytopenia below 50,000;
- A drop in haemoglobin to ≤ 6 g/dL;
- Per investigator discretion, a reduction in haemoglobin ≥ 25% of the Subject's baseline value.

Linezolid can be re-started at a later date at Investigator's discretion if there has not been a lapse in treatment of 35 consecutive days or more.

7.3.7 Peripheral Neuropathy

Investigator should consider witholding linezolid or discontinuing the full regimen permanently for subjects who experience a change in a grade or more for peripheral neuropathy (per DMID – Appendix 2).

Linezolid may be re-started at a later date if there has not been a lapse in treatment of 35 consecutive days or more.

7.3.8 Optic Neuropathy

Investigator should consider withholding linezolid and obtain further consultation with the site ophthalmologist for subjects with:

- A drop in visual acuity of two or more lines on the Snellen charts.
- Detection of loss of color vision by Ishihara plates defined as > 4 errors on the 12 plate screening test.

Linezolid may be re-started at a later date at the Investigator's discretion if there has not been a lapse in treatment of 35 consecutive days or more.

7.3.9 Lactic Acidosis

Investigator should consider withholding linezolid for subjects who experience unexplained lactic acidosis characterized with low bicarbonate levels, weakness and nausea, and subjects should receive immediate medical evaluation by the Investigator.

Linezolid may be re-started at a later date at the Investigator's discretion, if there has not been a lapse in treatment of 35 consecutive days or more.

7.3.10 Neurological

Subjects with co-administration of a serotonergic agent, including anti-depressants, should be monitored closely for signs of serotonin syndrome. The Investigator should determine whether permanent discontinuation of the full regimen or the concomitant agent should be discontinued for

those who experience signs or symptoms of serotonin syndrome such as cognitive dysfunction, hyperrexia, hyperreflexia and incoordination.

Linezolid and/or the full regimen should be withheld for subjects experiencing a seizure. The Sponsor Medical Monitor should be contacted to review details and discuss whether linezolid or full regimen should be resumed.

7.4 Safety Monitoring by the Data Safety Monitoring Committee

A DSMC will be appointed for the study. The primary responsibility of the DSMC will be to act in an advisory capacity to the Sponsor to safeguard the interests of trial Subjects by monitoring Subject safety, assess Subject risk versus benefit, and assess data quality and general evaluation of the trial progress. Its activities will be delineated in a DSMC charter that will define the membership, responsibilities and the scope and frequency of data reviews. The DSMC will operate on a conflict-free basis independently of the Sponsor and the study team. It will comprise at least 3 voting members. The DSMC may have an organisational meeting prior to commencement of the trial. The DSMC will have meetings where it will review unblinded data during a closed session. These meetings will be planned to occur after 10 Subjects complete 6 months post treatment assignment and again after the next 10 Subjects reach 6 months after treatment assignment. The DSMC will subsequently meet every 6 months. The Sponsor or the DSMC may convene ad hoc meetings based on rates of SAEs and/or to review results of the futility analysis or if safety concerns arise during the trial. After its assessment, the DSMC will recommend to the Sponsor continuation, modification or termination of the clinical trial.

8 STATISTICAL ANALYSIS

The statistical analysis plan (SAP), which will contain details of the analyses described generally in this section, will be written and signed off prior to Clinical Database Lock.

8.1 Analysis Population

The intention-to-Treat (ITT) analysis population will comprise of all subjects who were assigned study treatment.

The Safety analysis population will contain all subjects included in the ITT analysis population and received at least one administration of study drug.

The analysis populations will be defined in the SAP.

8.2 Sample Size

The objective of this trial is to evaluate the efficacy, safety, tolerability and pharmacokinetics of combinations of bedaquiline, linezolid and PA-824 in Subjects with either pulmonary XDR-TB, treatment intolerant or non-responsive MDR-TB.

Formal sample size calculations have not been performed due to the exploratory nature of the trial (no formal statistical hypothesis is therefore to be tested).

No formal interim analyses will be done for this study.

8.3 Interim Analyses

Interim analyses for futility will be performed for every 20 patients who reach the primary efficacy endpoint, treatment failure (that is, bacteriologic failure, or relapse, or clinical failure).

The study will be stopped for futility should the simultaneous upper confidence band for the “survival function” (probability, as a function of time after the assignment of study treatment, of not experiencing treatment failure) fall below 0.4 at any time point after the assignment of study treatment. Equivalently, the

study will be stopped for futility if the probability of treatment failure at any time point after the assignment of study treament is statistically significantly higher than 0.6 or 60%.

Once all patients have been recruited or have completed the treatment period, no further futility analyses will be performed

8.4 Primary Endpoint Analysis

The primary efficacy endpoint is treatment failure, defined as bacteriologic failure or relapse or clinical failure through follow-up until 24 months after the end of treatment.

The probability of treatment failure through follow-up until 24 months after the end of treatment, as a function of time after assignment of treatment, will be analyzed using Kaplan-Meier analysis.

The binomial proportion for subjects with bacteriologic failure will be presented.

No multiplicity adjustments for alpha will be done as this is an exploratory trial.

8.5 Secondary Endpoint Analysis

8.5.1 Efficacy

The secondary efficacy endpoints and analyses are as follows are:

- Time to sputum culture conversion to negative status through the treatment period.

The time to sputum culture conversion will be analyzed using Kaplan-Meier analysis.

- If liquid culture in the MGIT platform is used, the rate of change in log time of sputum culture positivity (TTP) over time in liquid culture (MGIT) in sputum.

If liquid culture in the MGIT platform is used, the rate of change in log time of sputum culture positivity (TTP) over time will be analyzed using non-linear mixed effects (NLME) regression modelling.

- Proportion of Subjects with sputum culture conversion to negative status at 4, 6, 8, 12 and 16 weeks with no subsequent, confirmed, positive culture(s).

- Proportion of Subjects experiencing a change from baseline of TB symptoms.

The binomial proportion for subjects with sputum culture conversion at each timepoint and subjects experiencing a change from baseline of TB symptoms will be presented.

- Change from baseline in Patient Reported Health Status.

The change from baseline in Patient Reported Health Status will be summarized using descriptive statistics by visit.

The effect of baseline covariates may be explored, including but not limited to the presence or absence of cavities on Chest X-Ray, the presence or absence of HIV infection and CD4 cell count.

8.6 Exploratory Endpoint Analysis

8.6.1 Efficacy

The exploratory efficacy endpoints and analyses are as follows:

- Evaluate whether any of the secondary endpoints predicts relapse free cure.
- Sub-analysis of populations by HIV status and CD4 count.
- Correlation of Time over mitochondrial protein synthesis inhibition (MPS50) with linezolid toxicity, (The MPS50 value will be an assumed value from literature).

Details for the analysis of the aforementioned endpoint will be described in the SAP.

8.6.2 Safety and Tolerability Analysis

- The indicence of all cause mortality will be summarized.
- All adverse events will be coded using the Medical Dictionary for Regulatory Activities (MedDRA) and will be presented by Preferred Term within each MedDRA System Organ Class (SOC).
- Treatment-emergent adverse events (TEAEs) are defined as AEs which started at or after the first administration of IMP and includes those events started prior to the first administration of IMP but which worsened after the first intake. Adverse events starting after the last administration of IMP until the last scheduled visit/assessment/measurement will be regarded as treatment-emergent.
- The incidence of the following events will be summarized for further medical analysis:
    - Incidence of TEAEs;
    - Incidence of TEAEs by Severity;
    - Incidence of TEAEs by DMID toxicity grade;
    - Incidence of Drug-Related TEAEs;
    - Incidence of Serious TEAEs;
    - Incidence of TEAEs Leading to Early Withdrawal;
    - Incidence of TEAEs leading to Death.
- Cardiovascular Safety: QT intervals will be adjusted using Fridericia's correction and Bazett's correction. QT/QTc values and changes from pre-dose (average of Screening and Day 1 values) at each time point will be summarized using descriptive statistics by group and time of collection. These will be presented as descriptive analyses, and no inferential tests will be carried out.
    - Post-baseline QT/QTc intervals will be classified into the following categories:
        - QT/QTc $<$ 450 msec
        - 450 msec $\leq$ QT/QTc $<$ 480 msec
        - 480 msec $\leq$ QT/QTc $<$ 500 msec
        - QT/QTc $\geq$ 500 msec
    - QTc changes from baseline will be classified into the following categories:
        - increase $<$ 30 msec,
        - $\geq$ 30 msec and $<$ 60 msec, and
        - increase $\geq$ 60 msec.
    - Frequency counts will be used to summarize the number of Subjects at each time point according to the above categories.
    - ECG results will be classified as normal or abnormal (investigator assessment) and summarized using frequency counts by dose group and time of collection.
    - Tukey honestly significant difference (HSD) analysis of the mean change from baseline in QTcB and QTcF interval across all post-baseline values.
- Ophthalmology: Descriptive statistics, including changes from baseline, will be summarized and listed by Subject for ophthalmology slit lamp examination (age related eye disease study 2 [AREDS2] lens opacity classification and grading). Categorical data for lens opacity will be summarized in a frequency table for the right and left eye, respectively.
- Visual acuity and color vision: Descriptive statistics, including changes from baseline, will be summarized and listed by Subject for both Visual Acuity and Color Asssessments. Categorical data for changes in visual acuity and color vision from baseline will be summarized in a frequency table for the right and left eye, respectively.
- Descriptive statistics of neuropathy data derived from Brief Peripheral Neuropathy Screen. Categorical data for observed signs and symptoms of neuropathy will be summarized in frequency tables, including changes in signs and symptoms from baseline.

- Other safety variables: Laboratory Parameters, Physical Examination, Vital signs (see Appendix 3), Concomitant medication, ophthalmic examination and peripheral neuropathy. Descriptive summary statistics will be presented. The incidence of liver related laboratory abnormalities will be explored.

8.7 Pharmacokinetics:

For each analyte (per visit), the PK plasma concentrations will be summarized by descriptive statistics, including the mean, SD, coefficient of variation (CV), median, minimum, maximum, geometric mean and geometric CV (%).

In addition, mean and median concentration-versus-time graphs will be provided (with error bars as appropriate).

For the PK sub-study samples, the following PK parameters will be estimated per analyte from the individual (per Subject) PK plasma concentrations: Minimum observed PK plasma concentration (C_{min}), maximum observed PK plasma concentration(C_{max}), time to reach C_{max} obtained without interpolation (T_{max}), area under the PK plasma concentration time (t) curve from zero to the last quantifiable PK plasma concentration prior to the subsequent dose, using the linear trapezoidal rule ($AUC_{(0-t)}$), area under the PK plasma concentration time (t) curve from zero to 24 hours ($AUC_{(0-24)}$).

8.8 Pharmacokinetics-Pharmacodynamics (PK-PD):

Correlations between plasma drug concentrations and efficacy and safety findings will be performed in an exploratory fashion.

8.9 General Mycobacteriology

Descriptive summary statistics of the mycobacterial characteristics will be presented.

9 RECORDS MANAGEMENT

9.1 Data Collection

All CRF/eCRF pages will be completed for each Subject who receives any amount of IMP. For Screening Failure Subjects a Screening failure CRF/eCRF will be completed. For Subjects who are prematurely withdrawn, the visits up to withdrawal plus the withdrawal and applicable follow-up visits need to be completed.

9.2 Source Documents

Source documents are defined as all information in original records and certified copies of original records of clinical findings, observations, or other activities in a clinical trial necessary for the reconstruction and evaluation of the trial. Source documents will include, but are not limited to, progress notes, electronic data, screening logs, and recorded data from automated instruments.

All source documents pertaining to this trial will be maintained by the Investigators. The Investigator has to permit trial-related monitoring, audits, Independent Ethics Committee/Institutional Review Board (IEC/IRB) review and regulatory inspections providing authorized persons direct access to source documents.

9.3 File Management at the Trial Centre

It is the responsibility of the Investigators to ensure that the trial center files are maintained in accordance with International Good Clinical Practice Guidelines and the ethical principles that have their origin in the Declaration of Helsinki.

9.4 Records Retention at the Trial Centre

The Investigator is obliged to retain records and data from the trial for safety reasons and for audit and inspection subsequent to trial completion. The essential documents should be retained for not less than 5 years after the last approval of a marketing application and until there are no pending or contemplated

marketing applications or at least 5 years have elapsed since the formal discontinuation of clinical development of the IMP.

The Sponsor will make financial provisions for the Investigator to deposit the documents at an external site for safekeeping for as long as required by regulations and the Sponsor.

10 QUALITY CONTROL AND ASSURANCE

10.1 Site Procedures

The Investigator undertakes to perform the clinical trial in accordance with this protocol, International GCP, and the ethical principles that have their origin in the Declaration of Helsinki, and applicable regulatory requirements.

The Investigator undertakes to complete the CRFs according to the Sponsor's requirements, in a timely, accurate and legible manner. CRF entries will be verifiable to source documentation other than the CRF.

Site Standard Operating Procedures will be adhered to for all clinical and bioanalytical activities relevant to the quality of the study. Subject compliance will be monitored throughout the study.

The Investigator will sign and date any analysis results (e.g. laboratory, ECG, etc.) to verify that the results have been reviewed.

The Investigator may appoint other Sub-Investigators to assist with the study. However the Investigator maintains responsibility for the study and will supervise the Sub-Investigators. Written IEC/IRB approval will be obtained prior to involvement in the study.

The Investigator will ensure that all site personnel are adequately trained in GCP, the protocol, IB and all study procedures and requirements.

10.2 Monitoring

The Investigator is responsible for the validity of all data collected at the clinical site and must accept the various monitoring procedures employed by the Sponsor. The purpose of monitoring is to verify that the rights and well-being of human Subjects are protected; that trial data are accurate, complete and verifiable with source data; and that the trial is conducted in compliance with the protocol, International GCP, the ethical principles that have their origin in the Declaration of Helsinki and the applicable regulatory requirements.

Monitors assigned by the Sponsor will conduct regular site visits for the purpose of monitoring various aspects of the study. Visits will take place usually within a predetermined interval, but this may vary during the course of the study. The Investigator and site staff will allow the study monitor and authorized representatives of the Sponsor to (1) inspect all CRFs, written informed consent documents and corresponding source documents (e.g. original medical records), Subject records and laboratory raw data, and (2) access clinical supplies, dispensing and storage areas. The Investigator and site staff should also (1) agree to assist with monitoring activities if requested and (2) provide adequate time and space for monitoring visits.

The monitor will query any missing, confusing, spurious, or otherwise ambiguous data with the Investigator. All queries should be resolved in a timely manner. A monitoring log will be maintained recording each visit, the reason for the visit, the monitor's signature and Investigator or designee's confirmation signature.

10.3 Auditing

For the purpose of compliance with International GCP and regulatory agency guidelines, it may be necessary for Sponsor-authorized Quality Assurance personnel and/or authorized personnel from an external regulatory agency to conduct an audit or inspection of the investigational site. The purpose of an audit is to assess the quality of data with regard to accuracy, adequacy and consistency, and to assure that studies are

in accordance with the guidelines. Having the highest quality data from studies is an essential aspect of drug development.

The Investigator and site staff will be given sufficient notice to prepare for such visits, which will usually last between one and two days and may be conducted at any stage during the study. The audit will involve the review of all study-related documentation required by GCP to be maintained by each site; drug storage, dispensing and return; all study-related supplies; and source documents against the CRFs to assure the adequacy and accuracy of the information which has been recorded, including the verification of any AEs which have occurred.

In the event of the site being notified of a Regulatory Inspection, the Sponsor will help with preparation. It is essential that the Sponsor be notified of the inspection as soon as possible.

11 ETHICS AND REGULATORY

11.8 Basic Principles

This research will be carried out in accordance with International GCP, the ethical principles that have their origin in the Declaration of Helsinki and the applicable regulatory requirements.

11.2 Independent Ethics Committee/Institutional Review Board (IEC/IRB) Review

The protocol and required study related documents will be reviewed by the sites respective IEC/IRB. The study will not start until the IEC/IRB has approved the protocol, written informed consent, any written information to be provided to the Subject or any modification thereof, plus any other study related documents required for review. The IEC/IRB shall be constituted and shall operate in accordance with International GCP, the ethical principles that have their origin in the Declaration of Helsinki.

The Investigator will maintain an accurate and complete record of all submissions made to the IRB/IEC. The records should be filed in the Investigator's Study File, and copies will be sent to the Sponsor. The Investigator may delegate IRB/IEC communication responsibilities to another party/vendor (e.g. CRO). This delegation should be clearly documented in writing and filed with the study documents at the site.

11.3 Regulatory Authorities

The Regulatory Authorities will receive the protocol, amendments, reports on SAEs, and the Integrated Clinical Trial Report according to national regulations. As required by local legislation, written approval will be obtained from the Regulatory Authorities prior to commencement of the trial and implementation of e.g. amendments as applicable.

11.4 Informed Consent

Written informed consent will be obtained from all Subjects (or legally acceptable representative) before any trial-related procedures (including any screening or pre-treatment procedures) are performed. Investigators may discuss the availability of the trial and the opportunity for entry with a potential Subject without first obtaining consent. However, informed consent must be obtained and documented prior to initiation of any procedures that are performed solely for the purpose of determining eligibility for research, including withdrawal from current medication(s). When this is done in anticipation of, or in preparation for, the research, it is considered to be part of the research.

The Investigators have both ethical and legal responsibility to ensure that each Subject being considered for inclusion in this trial is given a full explanation of the protocol. This shall be documented on a written informed consent form that shall be approved by the same IEC/IRB responsible for approval of this protocol. Each informed consent form shall include the elements required by the international GCP and must adhere to the ethical principles that have their origin in the Declaration of Helsinki.

Once the appropriate essential information has been provided to the Subject and fully explained by the Investigators (or qualified designees) and it is felt that the Subject understands the implications of

participating, the IEC/IRB approved written informed consent form will be signed and dated by both the Subject and the person obtaining consent (Investigators or designees), and by any other parties required by the IEC/IRB.

The original signed informed consent form will be kept with the trial records and a copy of signed informed consent form will be provided to the Subject. Another copy of the signed informed consent form and a source document identifying the trial and recording the dates of participation will be placed in the Subject's medical record.

The monitor will inspect the original completed consent form(s) for all Subjects.

11.5 Confidentiality

All site staff, the Sponsor, and any Sponsor representatives will preserve the confidentiality of all Subjects taking part in the study, in accordance with International GCP, applicable local legislation/regulations. Subject to the requirement for source data verification by the study personnel by reference to the Subject's notes, confidentiality of all Subject identities will be maintained. Only Subject study number and initials will be used on the CRF and in all study correspondence, as permitted. No material bearing a Subject's name will be kept on file by the Sponsor. The written informed consent will contain a clause granting permission for review of the Subjects' source data.

12 PUBLICATION POLICY

The definition of publication for this purpose is any public presentation of the data emerging from this study.

All unpublished information given to the Investigator by the Sponsor shall not be published or disclosed to a third party, other than to the responsible IEC/IRB, within the understanding of the confidentiality of their nature, without the prior written consent of the Sponsor.

Results of this research will be submitted for publication as soon as feasible upon completion of the study in the form of a joint publication(s) between Sponsor and Investigator(s), including site clinical and laboratory Investigators, as appropriate.

13 PROTOCOL AMENDMENT POLICY

Any change to the protocol will be effected by means of a protocol amendment. Any changes which affect Subject safety or welfare will be submitted to the IEC/IRB and Regulatory Authorities prior to implementation. The Investigator, IEC/IRB, and Sponsor must agree on all amendments. No amendment will be implemented until approved by the relevant Authorities and/or IEC/IRB and signed by all required parties. Exceptions to this are when the Investigator considers that the Subject's safety is compromised.

Protocol amendments detailing minor administrative changes should be submitted by the Investigator to the IEC/IRB and Regulatory Authorities, either for notification purposes or approval as appropriate.

14 FINANCIAL ASPECTS, INSURANCE AND INDEMNITY

The study Sponsor and funder is the Global Alliance for TB Drug Development (TB Alliance). The TB Alliance is a not for profit, product development partnership accelerating the discovery and development of new TB drugs that will shorten treatment, be effective against susceptible and resistant strains, be compatible with antiretroviral therapies for those HIV-TB Subjects currently on such therapies, and improve treatment of latent infection.

The TB Alliance works with public and private partners worldwide. It is committed to ensuring that approved new regimens are affordable, adopted and available to those who need them.

The Subjects will not receive any incentives for their involvement in the study. The Sponsor has made provision to reimburse the Subjects for out-of-pocket expenses such as travelling to and from the study site and other miscellaneous costs as a result of their study participation.

The Sponsor certifies that it has liability insurance coverage for itself and will provide an associated certificate upon request. The insurance does not relieve the Investigators of the obligation to maintain their own liability insurance as required by applicable law. The Sponsor does not assume any obligation for the medical treatment of other injuries and illnesses.

15 REFERENCES

1. Dietze R, Teixeira L, Rocha LMC, et al. "Safety and bactericidal activity of rifalazil in patients with pulmonary tuberculosis" Antimicrob Agents Chemother 2001; 47 (7): 1972-6.
2. Donald PR, Sirgel FA, Venter A, et al. "Early bactericidal activity of antituberculosis agents" Expert Rev Anti-infect Ther 2003; 1 (1): 141-55.
3. Package Insert, Sirturo™, Janssen Therapeutics, February 2012
4. TMC207 Investigator's Brochure, Janssen Research and Development, Edition 7, 18 September 2012 .
5. TMC207 Addendum 1 to Investigator's Brochure Edition 7, 3 July 2013.
6. Koul A, Dendouga N, Vergauwen K, et al. "Diarylquinolines target subunit c of mycobacterial ATP synthase" Nat Chem Biol 2007; 3: 323-4.
7. Andries K, Verhasselt P, Guillemont J, et al. "A diarylquinoline drug active on the ATP synthase of Mycobacterium turberculosis" Science 2005; 307: 223-7.
8. Huitric E, Verhasselt P, Andries K, Hoffner SE. "In vitro antimycobacterial spectrum of a diarylquinoline ATP synthase inhibitor" Antimicrob Agents Chemother 2007; 51: 4202-4.
9. Koul A, Vranckx L, Dendouga N, et al. "Diarylquinolines are bactericidal for dormant mycobacteria as a result of disturbed homeostasis" J Biol Chem 2008; 283: 25273-80.
10. Mdluli K, Nuermberger, E. "Pre-Clinical Drug Combination Studies to Identify Novel Regimens for TB". In: Recent Advances in TB Drug Development symposium, 40th Union World Conference, International Union Against TB and Lung Disease, Cancun, Mexico, 2009.
11. Mdluli K, Nuermberger E, Taylor K, Williams N, Koen A, Khisimuzi E, Rokeya T, Si-Yang L, Peloquin C, Dinesh C. "Sterilizing activity of novel TMC207- and PA-824-containing regimens in a murine model of tuberculosis" Antimicrobial Agents and Chemotherapy 2011; 55: 5485-5492.
12. Rustomjee R, Diacon AH, Allen J, et al. "Early bactericidal activity and pharmacokinetics of the diarylquinoline TMC207 in treatment of pulmonary tuberculosis" Antimicrob Agents Chemother 2008 Aug; 52(8): 2831-5. Epub 2008 May 27.
13. Diacon AH, Dawson R, Von Groote-Bidlingmaier F, Symons G, Venter A, Donald PR, Conradie A, Erondu N, Ginsberg AM, Egizi E, Winter H, Becker P, Mendel CM. "Randomized dose-ranging study of the 14-day early bactericidal activity of bedaquiline (TMC207) in patients with sputum microscopy smear-positive pulmonary tuberculosis" Antimicrob Agents Chemother. 2013 May; 57(5):2199-203. doi: 10.1128/AAC.02243-12.
14. Diacon AH, Dawson R, von Groote-Bidlingmaier F, Symons G, Venter A, Donald PR, van Niekerk C, Everitt D, Winter H, Becker P, Mendel CM, Spigelman MK. "14-day bactericidal activity of PA-824, bedaquiline, pyrazinamide, and moxifloxacin combinations: a randomised trial". Lancet. 2012 Sep 15; 380(9846):986-93. doi: 10.1016/S0140-6736(12)61080-0. Epub 2012 Jul 23
15. Investigator's Brochure, Global Alliance for TB Drug Development, PA-824, Version 16, April 2014.
16. Stover, C. K., P. Warrener, D. R. VanDevanter, et al. 2000. "A small-molecule nitroimidazopyran drug candidate for the treatment of tuberculosis" Nature 405:962–966.
17. Lenaerts AJ, Gruppo V, Marietta KS, Johnson CM, Driscoll DK, Tompkins NM, Rose JD, Reynolds RC, and Orme IM. "Preclinical Testing of the Nitroimidazopyran PA-824 for Activity against Mycobacterium tuberculosis in a Series of In Vitro and In Vivo Models" Antimicrob Agents Chemother. 2005 June; 49(6): 2294–2301.
18. Package Insert, ZYVOX™, Pfizer Pharmaceuticals, January 2014.

19. Oxford Textbook of Palliative Medicine, Oxford University Press. 1993; 109.
20. “Multidrug-resistant tuberculosis (MDR-TB) Fact Sheet October 2013 Update” © WHO October 2013 (GTB number c7)
Available: http://www.who.int/tb/challenges/mdr/mdr_tb_factsheet.pdf
21. Gandhi NR, et al. "Extensively drug-resistant tuberculosis as a cause of death in patients co-infected with tuberculosis and HIV in a rural area of South Africa" Lancet 2006 Nov 4; 368(9547):1575-80.
22. Centers for Disease Control and Prevention (2006) “Emergence of Mycobacterium tuberculosis with extensive resistance to second-line drugs worldwide, 2000–2004” MMWR Morb Mortal Wkly Rep 55: 301–305. Available: http://www.cdc.gov/mmwr/preview/mmwrhtml/mm5511a2.htm
23. Pietersen E, et al. "Long-term outcomes of patients with exntensively drug-resistant tuberculosis in South Africa: a cohort study" Lancet, 2014 Jan 16. pii: S0140-6736(13)62675-6.
24. Lee, et al. "Linezolid for treatment of chronic extensively drug-resistant tuberculosis" N Engl J Med. 2012 Oct 18; 367(16):1508-18.
25. Leach KL, Brickner SJ, Noe MC, Miller PF. "Linezolid, the first oxazolidinone antibacterial agent" Ann NY Acad Sci. 2011 Mar; 1222:49-54. 2011. Lee, et al. "Prevalence of and risk factors for resistance to second-line drugs in people with multi-drug resistant tuberculosis in eight countries: a prospective cohort study” Lancet. 2012 Oct 20; 380(9851):1406-17.
26. Tato M, et al. "In vitro activity of linezolid against Mycobacterium tuberculosis complex, including multidrug-resistant Mycobacterium bovis isolates" Int. J. Antimicrob Agents 2006 Jul;28(1)75-8.
27. WHO Treatment of Tuberculosis Guidelines, 4th Edition. 2010.
28. Schecter GF, et al. “Linezolid Treatment for MDR-TB” Clinical Infectious Diseases 2010; 50:49-55.
29. Park IN, et al. “Efficacy and tolerability of daily-half dose linezolid in patients with intractable multidrug-resistant tuberculosis.” B Antimicrobial Chemotherapy 2006; 58:701-704.;
30. Singla R. "Linezolid: an Effective, Safe and Cheap Drug for Patients Failing Multidrug-resistant Tuberculosis Treatment in India" Eur Respir J. 2012 Apr;39(4):956-62. doi: 10.1183/09031936.00076811. Epub 2011 Sep 29.
31. Approved Package Insert for South Africa, ZYVOXID™ (linezolid), Pfizer Pharmaceuticals, 02 March 2012.
32. Padayatchi N, Mac Kenzie WR, Hirsch-Moverman Y, Feng PJ, Villarino E, Saukkonen J, Heilig CM, Weiner M, El-Sadr WM. “Lessons from a randomised clinical trial for multidrug-resistant tuberculosis” INT J TUBERC LUNG DIS 16(12):1582–1587,2012. http://dx.doi.org/10.5588/ijtld.12.0315
33. Cox H, Ford, N. “Linezolid for the treatment of complicated drug-resistant tuberculosis: a systematic review and meta-analysis” INT J TUBERC LUNG DIS 16(4):447–454, 2012.
34. Package Insert, Lizomac™ 600 (linezolid), MacLeods Pharmaceutical, 2014.

APPENDIX 1 THE IUATLD SCALE

The IUATLD scale proposes five groups for reporting the results of reading smears for acid fast bacilli. They should be recorded as follows:

FINDING	RECORDING
No acid-fast bacilli found in at least 100 fields	negative
1 to 9 acid-fast bacilli per 100 fields	exact figure/100/scanty positive
10 to 99 acid-fast bacilli per 100 fields	+
1 to 10 acid-fast bacilli per field in at least 50 fields	++
More than 10 acid-fast bacilli per field in at least 20 fields	+++

Reference: The Public Health Service National Tuberculosis Reference Laboratory and the National Laboratory Network. Minimum Requirements, Role and Operation in a Low-Income Country. International Union Against Tuberculosis and Lung Disease 1998.

APPENDIX 2 DIVISION OF MICROBIOLOGY AND INFECTIOUS DISEASES (DMID) ADULT TOXICITY TABLE

Source: U.S. National Institute of Allergy and Infectious Diseases, DMID, November 2007 (Draft)

ABBREVIATIONS: Abbreviations utilized in the Table:

ULN = Upper Limit of Normal	LLN = Lower Limit of Normal
R_x = Therapy	Req = Required
Mod = Moderate	IV = Intravenous
ADL = Activities of Daily Living	Dec = Decreased

ESTIMATING SEVERITY GRADE

For abnormalities NOT found elsewhere in the Toxicity Tables use the scale below to estimate grade of severity:

Grade	Severity Rating	Definition
**GRADE 1**	Mild	Transient or mild discomfort (< 48 hours); no medical intervention/therapy required.
**GRADE 2**	Moderate	Mild to moderate limitation in activity - some assistance may be needed; no or minimal medical intervention/therapy required.
**GRADE 3**	Severe	Marked limitation in activity, some assistance usually required; medical intervention/therapy required, hospitalizations possible.
**GRADE 4**	Potentially Life Threatening	Extreme limitation in activity, significant assistance required; significant medical intervention/therapy required, hospitalization or hospice care probable.

SERIOUS OR LIFE-THREATENING AEs

ANY clinical event deemed by the clinician to be serious or life-threatening should be considered a grade 4 event. Clinical events considered to be serious or life-threatening include, but are not limited to: seizures, coma, tetany, diabetic ketoacidosis, disseminated intravascular coagulation, diffuse petechiae, paralysis, acute psychosis, severe depression.

COMMENTS REGARDING THE USE OF THESE TABLES

- Standardized and commonly used toxicity tables (Division of AIDS, NCI's Common Toxicity Criteria (CTC), and World Health Organization (WHO)) have been adapted for use by the Division of Microbiology and Infectious Diseases (DMID) and modified to better meet the needs of patients in DMID trials.
- For parameters not included in the following Toxicity Tables, sites should refer to the "Guide For Estimating Severity Grade" located above.
- Criteria are generally grouped by body system.
- Some protocols may have additional protocol specific grading criteria, which will supersede the use of these tables for specified criteria.

**HEMATOLOGY**				
	**Grade 1**	**Grade 2**	**Grade 3**	**Grade 4**
**Hemoglobin**	9.5 - 10.5 gm/dL	8.0 - 9.4gm/dL	6.5 - 7.9 gm/dL	< 6.5 gm/dL
**Absolute Neutrophil Count**	1000-1500/mm^3	750-999/mm^3	500-749/mm^3	<500/mm^3
**Platelets**	75,000-99,999/mm^3	50,000-74,999/mm^3	20,000-49,999/mm^3	<20,000/mm^3
**WBCs**	11,000-13,000/ mm^3	13,000-15,000 /mm^3	15,000-30,000/mm^3	>30,000 or <1,000 /mm^3
**% Polymorphonuclear Leucocytes + Band Cells**	> 80%	90 – 95%	>95%	----------
**Abnormal Fibrinogen**	Low:<br>100-200 mg/dL<br>High:<br>400-600 mg/dL	Low:<br><100 mg/dL<br>High:<br>>600 mg/dL	Low:<br>< 50 mg/dL<br>----------	Fibrinogen associated with gross bleeding or with disseminated coagulation
**Fibrin Split Product**	20-40 mcg/ml	41-50 mcg/ml	51-60 mcg/ml	> 60 mcg/ml
**Prothrombin Time (PT)**	1.01 - 1.25 x ULN	1.26-1.5 x ULN	1.51 -3.0 x ULN	>3 x ULN
**Activated Partial Thromboplastin (APPT)**	1.01 -1.66 x ULN	1.67 - 2.33 x ULN	2.34 - 3 x ULN	> 3 x ULN
**Methemoglobin**	5.0 - 9.9 %	10.0 - 14.9 %	15.0 - 19.9%	> 20.0 %

**CHEMISTRIES**				
	**Grade 1**	**Grade 2**	**Grade 3**	**Grade 4**
**Hyponatremia**	130-135 mEq/L	123-129 mEq/L	116-122 mEq/L	< 116 mEq/L or abnormal sodium *with* mental status changes or seizures
**Hypernatremia**	146-150 mEq/L	151-157 mEq/L	158-165 mEq/L	> 165 mEq/L or abnormal sodium *with* mental status changes or seizures
**Hypokalemia**	3.0 - 3.4 mEq/L	2.5 - 2.9 mEq/L	2.0 - 2.4 mEq/L or intensive replacement therapy or hospitalization required	< 2.0 mEq/L or abnormal potassium *with* paresis, ileus or life-threatening arrhythmia
**Hyperkalemia**	5.6 - 6.0 mEq/L	6.1 - 6.5 mEq/L	6.6 - 7.0 mEq/l	> 7.0 mEq/L or abnormal potassium *with* life-threatening arrhythmia
**Hypoglycemia**	55-64 mg/dL	40-54 mg/dL	30-39 mg/dL	<30 mg/dL or abnormal glucose *with* mental status changes or coma
**Hyperglycemia (nonfasting and no prior diabetes)**	116 - 160 mg/dL	161- 250 mg/dL	251 - 500 mg/dL	> 500 mg/dL or abnormal glucose *with* ketoacidosis or seizures
**Hypocalcemia (corrected for albumin)**	8.4 - 7.8 mg/dL	7.7 - 7.0 mg/dL	6.9 - 6.1 mg/dL	< 6.1 mg/dL or abnormal calcium *with* life threatening arrhythmia or tetany
**Hypercalcemia (correct for albumin)**	10.6 - 11.5 mg/dL	11.6 - 12.5 mg/dL	12.6 - 13.5 mg/dL	> 13.5 mg/dL or abnormal calcium *with* life threatening arrhythmia
**Hypomagnesemia**	1.4 - 1.2 mEq/L	1.1 - 0.9 mEq/L	0.8 - 0.6 mEq/L	< 0.6 mEq/L or abnormal magnesium *with* life-threatening arrhythmia
**Hypophosphatemia**	2.0 - 2.4 mg/dL	1.5 -1.9 mg/dL or replacement Rx required	1.0 -1.4 mg/dL intensive therapy or hospitalization required	< 1.0 mg/dL or abnormal phosphate *with* life-threatening arrhythmia
**Hyperbilirubinemia (when accompanied by any increase in other liver function test)**	1.1 - <1.25 x ULN	1.25 - <1.5 x ULN	1.5 – 1.75 x ULN	> 1.75 x ULN
**Hyperbilirubinemia (when other liver function are in the normal range)**	1.1 - <1.5 x ULN	1.5 - <2.0 x ULN	2.0 – 3.0 x ULN	> 3.0 x ULN
**BUN**	1.25 - 2.5 x ULN	2.6 - 5 x ULN	5.1 - 10 x ULN	> 10 x ULN
**Hyperuricemia (uric acid)**	7.5 – 10.0 mg/dL	10.1 – 12.0 mg/dL	12.1 – 15.0 mg/dL	>15.0 mg/dL
**Creatinine**	1.1 - 1.5 x ULN	1.6 - 3.0 x ULN	3.1 - 6 x ULN	> 6 x ULN or dialysis required

ENZYMES				
	**Grade 1**	**Grade 2**	**Grade 3**	**Grade 4**
**AST (SGOT)**	1.1 - <2.0 x ULN	2.0 – <3.0 x ULN	3.0 – 8.0 x ULN	> 8 x ULN
**ALT (SGPT)**	1.1 - <2.0 x ULN	2.0 – <3.0 x ULN	3.0 – 8.0 x ULN	> 8 x ULN
**GGT**	1.1 - <2.0 x ULN	2.0 – <3.0 x ULN	3.0 – 8.0 x ULN	> 8 x ULN
**Alkaline Phosphatase**	1.1 - <2.0 x ULN	2.0 – <3.0 x ULN	3.0 – 8.0 x ULN	> 8 x ULN
**Amylase**	1.1 - 1.5 x ULN	1.6 - 2.0 x ULN	2.1 - 5.0 x ULN	> 5.1 x ULN
**Lipase**	1.1 - 1.5 x ULN	1.6 - 2.0 x ULN	2.1 - 5.0 x ULN	> 5.1 x ULN

URINALYSIS				
	**Grade 1**	**Grade 2**	**Grade 3**	**Grade 4**
**Proteinuria**	1+ or 200 mg - 1 gm loss/day	2-3+ or 1- 2 gm loss/day	4+ or 2-3.5 gm loss/day	nephrotic syndrome or > 3.5 gm loss/day
**Hematuria**	microscopic only <10 rbc/hpf	gross, no clots >10 rbc/hpf	gross, with or without clots, OR red blood cell casts	obstructive or required transfusion

CARDIOVASCULAR				
	**Grade 1**	**Grade 2**	**Grade 3**	**Grade 4**
**Cardiac Rhythm**		asymptomatic, transient signs, no Rx required	recurrent/persistent ; symptomatic Rx required	unstable dysrythmia; hospitalization and treatment required
**Hypertension**	transient increase > 20 mm/Hg; no treatment	recurrent, chronic increase > 20mm/Hg. /treatment required	acute treatment required; outpatient treatment or hospitalization possible	end organ damage or hospitalization required
**Hypotension**	transient orthostatic hypotension with heart rate increased by <20 beat/min or decreased by <10 mm Hg systolic BP, No treatment required	symptoms due to orthostatic hypotension or BP decreased by <20 mm Hg systolic; correctable with oral fluid treatment	requires IV fluids; no hospitalization required	mean arterial pressure <60mm/ Hg or end organ damage or shock; requires hospitalization and vasopressor treatment
**Pericarditis**	minimal effusion	mild/moderate asymptomatic effusion, no treatment	symptomatic effusion; pain; EKG changes	tamponade; pericardiocentesis or surgery required
**Hemorrhage, Blood Loss**	microscopic/occult	mild, no transfusion	gross blood loss; 1-2 units transfused	massive blood loss; > 3 units transfused

RESPIRATORY				
	**Grade 1**	**Grade 2**	**Grade 3**	**Grade 4**
**Cough**	Transient - no treatment	persistent cough; treatment responsive	Paroxysmal cough; uncontrolled with treatment	--------------------
**Bronchospasm, Acute**	transient; no treatment; 70% - 80% FEV_1 of peak flow	requires treatment; normalizes with bronchodilator; FEV_1 50% - 70% (of peak flow)	no normalization with bronchodilator;FEV_1 25% - 50% of peak flow; or retractions present	cyanosis: FEV_1 < 25% of peak flow or intubation necessary
**Dyspnea**	dyspnea on exertion	dyspnea with normal activity	dyspnea at rest	dyspnea requiring Oxygen therapy

**GASTROINTESTINAL**				
	**Grade 1**	**Grade 2**	**Grade 3**	**Grade 4**
**Nausea**	mild or transient; maintains reasonable intake	moderate discomfort; intake decreased significantly; some activity limited	no significant intake; requires IV fluids	hospitalization required;
**Vomiting**	1 episode in 24 hours	2-5 episodes in 24 hours	>6 episodes in 24 hours or needing IV fluids	physiologic consequences requiring hospitalization or requiring parenteral nutrition
**Constipation**	requiring stool softener or dietary modification	requiring laxatives	obstipation requiring manual evacuation or enema	obstruction or toxic megacolon
**Diarrhea**	mild or transient; 3-4 loose stools/day or mild diarrhea last < 1 week	moderate or persistent; 5-7 loose stools/day or diarrhea lasting >1 week	>7 loose stools/day or bloody diarrhea; or orthostatic hypotension or electrolyte imbalance or >2L IV fluids required	hypotensive shock or physiologic consequences requiring hospitalization
**Oral Discomfort/Dysphagia**	mild discomfort; no difficulty swallowing	some limits on eating/drinking	eating/talking very limited; unable to swallow solid foods	unable to drink fluids; requires IV fluids

NEUROLOGICAL				
	**Grade 1**	**Grade 2**	**Grade 3**	**Grade 4**
**Neuro-Cerebellar**	slight incoordination dysdiadochokinesis	intention tremor, dysmetria, slurred speech; nystagmus	locomotor ataxia	incapacitated
**Psychiatric**	mild anxiety or depression	moderate anxiety or depression; therapy required; change in normal routine	severe mood changes requiring therapy; or suicidal ideation; or aggressive ideation	acute psychosis requiring hospitalization; or suicidal gesture/attempt or hallucinations
**Muscle Strength**	Subjective weakness no objective symptoms/signs	mild objective signs/symptoms no decrease in function	objective weakness function limited	paralysis
**Paresthesia (burning, tingling, etc.)**	mild discomfort; no treatment required	moderate discomfort; non-narcotic analgesia required	severe discomfort; or narcotic analgesia required with symptomatic improvement	incapacitating; or not responsive to narcotic analgesia
**Neuro-sensory**	mild impairment in sensation (decreased sensation, e.g., vibratory, pinprick, hot/cold in great toes) in focal area or symmetrical distribution; or change in taste, smell, vision and/or hearing	moderate impairment (mod decreased sensation, e.g., vibratory, pinprick, hot/cold to ankles) and/or joint position or mild impairment that is not symmetrical	severe impairment (decreased or loss of sensation to knees or wrists) or loss of sensation of at least mod degree in multiple different body areas (i.e., upper and lower extremities)	sensory loss involves limbs and trunk; paralysis; or seizures

**MUSCULOSKELETAL**				
	**Grade 1**	**Grade 2**	**Grade 3**	**Grade 4**
**Arthralgia (joint pain)**	mild pain not interfering with function	moderate pain, analgesics and/or pain interfering with function but not with activities of daily living	severe pain; pain and/or analgesics interfering with activities of daily living	disabling pain
**Arthritis**	mild pain with inflammation, erythema or joint swelling – but not interfering with function	moderate pain with inflammation, erythema or joint swelling – interfering with function, but not with activities of daily living	severe pain with inflammation, erythema or joint swelling –and interfering with activities of daily living	permanent and/or disabling joint destruction
**Myalgia**	myalgia with no limitation of activity	muscle tenderness (at other than injection site) or with moderate impairment of activity	severe muscle tenderness with marked impairment of activity	frank myonecrosis

**SKIN**				
	**Grade 1**	**Grade 2**	**Grade 3**	**Grade 4**
**Mucocutaneous**	erythema; pruritus	diffuse, maculo papular rash, dry desquamation	vesiculation or moist desquamation or ulceration	exfoliative dermatitis, mucous membrane involvement or erythema, multiforme or suspected Stevens-Johnson or necrosis requiring surgery
**Induration**	< 15mm	15-30 mm	>30mm	
**Erythema**	< 15mm	15-30 mm	>30mm	
**Edema**	< 15mm	15-30 mm	>30mm	
**Rash at Injection Site**	< 15mm	15-30 mm	>30mm	
**Pruritus**	slight itching at injection site	moderate itching at injection extremity	itching over entire body	

SYSTEMIC				
	**Grade 1**	**Grade 2**	**Grade 3**	**Grade 4**
Allergic Reaction	pruritus without rash	localized urticaria	generalized urticaria; angioedema	anaphylaxis
Headache	mild, no treatment required	transient, moderate; treatment required	severe; responds to initial narcotic therapy	intractable; requires repeated narcotic therapy
Fever: oral	37.7 - 38.5 C or 100.0 - 101.5 F	38.6 - 39.5 C or 101.6 - 102.9 F	39.6 - 40.5 C or 103 - 105 F	> 40 C or > 105 F
Fatigue	normal activity reduced < 48 hours	normal activity decreased 25- 50% > 48 hours	normal activity decreased > 50% can't work	unable to care for self

APPENDIX 3 VITAL SIGNS

Vital Signs

The following abnormalities will be defined for vital signs:

**Abnormality Code**	**Vital Signs parameter**		
	**Pulse**	**DBP**	**SBP**
**Abnormalities on actual values**			
**"Abnormally low"**	≤ 50 bpm	≤ 50 mmHg	≤ 90 mm Hg
**"Grade 1 or mild"**	-	> 90 mmHg-<100 mmHg	> 140 mmHg-<160 mmHg
**"Grade 2 or moderate"**	-	≥ 100 mmHg-<110 mmHg	≥ 160 mmHg-<180 mmHg
**"Grade 3 or severe"**	-	≥ 110 mmHg	≥ 180 mmHg
**"Abnormally high"**	≥ 120 bpm	-	-

APPENDIX 4 KARNOFSKY PERFORMANCE STATUS SCALE DEFINITIONS RATING (%) CRITERIA[19]

Description		%
Able to carry on normal activity and to work; no special care needed.	Normal no complaints; no evidence of disease.	100
	Able to carry on normal activity; minor signs or symptoms of disease.	90
	Normal activity with effort; some signs or symptoms of disease.	80
Unable to work; able to live at home and care for most personal needs; varying amount of assistance needed.	Cares for self; unable to carry on normal activity or to do active work.	70
	Requires occasional assistance, but is able to care for most of his personal needs.	60
	Requires considerable assistance and frequent medical care.	50
Unable to care for self; requires equivalent of institutional or hospital care; disease may be progressing rapidly.	Disabled; requires special care and assistance.	40
	Severely disabled; hospital admission is indicated although death not imminent.	30
	Very sick; hospital admission necessary; active supportive treatment necessary.	20
	Moribund; fatal processes progressing rapidly.	10
	Dead	0

Ref: Oxford Textbook of Palliative Medicine, Oxford University Press. 1993; 109.

APPENDIX 5 EQ-5D-5L QUESTIONNAIRE

Under each heading, please tick the ONE box that best describes your health TODAY

MOBILITY

I have no problems in walking about ❑
I have slight problems in walking about ❑
I have moderate problems in walking about ❑
I have severe problems in walking about ❑
I am unable to walk about ❑

SELF-CARE

I have no problems washing or dressing myself ❑
I have slight problems washing or dressing myself ❑
I have moderate problems washing or dressing myself ❑
I have severe problems washing or dressing myself ❑
I am unable to wash or dress myself ❑

USUAL ACTIVITIES *(e.g. work, study, housework, family or leisure activities)*

I have no problems doing my usual activities ❑
I have slight problems doing my usual activities ❑
I have moderate problems doing my usual activities ❑
I have severe problems doing my usual activities ❑
I am unable to do my usual activities ❑

PAIN / DISCOMFORT

I have no pain or discomfort ❑
I have slight pain or discomfort ❑
I have moderate pain or discomfort ❑
I have severe pain or discomfort ❑
I have extreme pain or discomfort ❑

ANXIETY / DEPRESSION

I am not anxious or depressed ❑
I am slightly anxious or depressed ❑
I am moderately anxious or depressed ❑
I am severely anxious or depressed ❑
I am extremely anxious or depressed ❑

SAMPLE

UK (English) v.2 © 2009 EuroQol Group. EQ-5D™ is a trade mark of the EuroQol Group

- We would like to know how good or bad your health is TODAY.
- This scale is numbered from 0 to 100.
- 100 means the best health you can imagine.
  0 means the worst health you can imagine.
- Mark an X on the scale to indicate how your health is TODAY.
- Now, please write the number you marked on the scale in the box below.

YOUR HEALTH TODAY = ☐

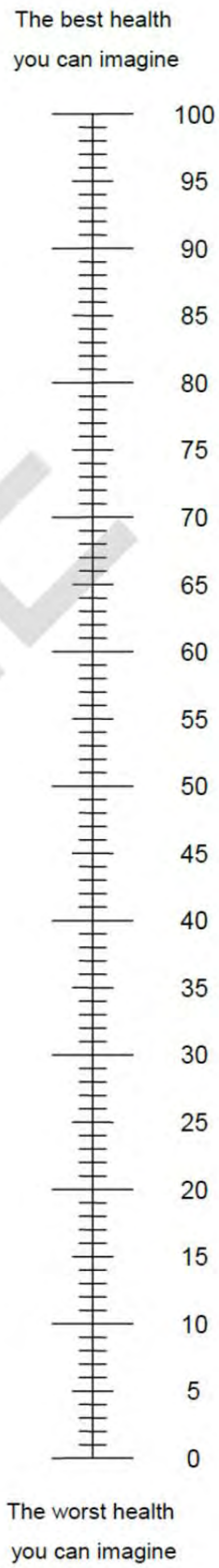

SAMPLE

UK (English) v.2 © 2009 EuroQol Group. EQ-5D™ is a trade mark of the EuroQol Group

APPENDIX 6 BRIEF PERIPHERAL NEUROPATHY SCREENING

BRIEF PERIPHERAL NEUROPATHY SCREEN

Patient Initials				Patient ID									

**1.** Visit (Circle One)								
	All Subjects	**Baseline**	**Week 4**	**Week 8**	**Week 12**	**Week 16**	**Week 20**	**Week 26**
		**3 Month**	**6 Month**	**12 Month**	**24 Month**			
	9 Month Treatment **ONLY**	**Week 30**	**Week 34**	**Week 39**				
	Other	**Early Withdrawal**	**Unscheduled** For new onset or worsening peripheral neuropathy during treatment					

**2.** Date of Assessment	D	D	M	M	M	Y	Y	Y	Y

INTERFERENCE WITH WALKING OR SLEEPING

**3.** In the last two weeks, have pain, aching or burning in your feet interfered with your walking or sleeping? (Check one)	Y	N

3a. *If* ***YES****, ask the patient to rate the level of interference (1 to 10) to his walking or sleeping caused by this pain, ache or burning (circle one).*

**Minimal**			**Modest**				**Severe**		
01	02	03	04	05	06	07	08	09	10

SUBJECT ELICITED SYMPTOMS

- *Using the faces below, ask the patient to rate the severity of the symptoms for the questions 8, 9, 10 on a scale of 1 (mild) to 10 (severe) for both feet. If the severity is different between the left and right foot, record the severity of the most affected foot.*
- *Enter a score for each symptom.*
- *If a symptom has been present in the past, but not since the last visit, enter '00 – Currently Absent'*
- *If a symptom has never been present, enter '11 – Always Been Normal'*

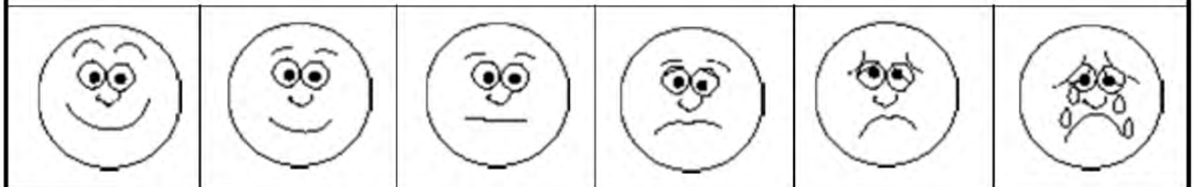

**00**	**02**	**04**	**06**	**08**	**10**
Very Happy, No Symptoms	Just a little bit	A little more	Even more	A whole lot	Worst

**During the last 14 days, have you experienced:**		**Severity**	
	**4.** Pain, aching or burning in feet or legs?		
	**5.** "Pins and needles" in feet or legs?		
	**6.** Numbness (lack of feeling) in feet or legs?		

BRIEF PERIPHERAL NEUROPATHY SCREEN

Patient Initials				Patient ID									

PERCEPTION OF VIBRATION

- *Press the ends of a 128 Hz tuning fork together so the sides touch and let go. Place the vibrating tuning fork on the bony prominence on the patient's wrist to be sure that they can recognize the vibration or "buzzing" quality of the tuning fork.*
- *Again, press the ends of the tuning fork hard enough so that the sides touch and let go. Immediately place the vibrating tuning fork gently but firmly on the top of the distal interphalangeal (DIP) joint of the great toe and begin counting the seconds. Instruct the Subject to tell you when they stop feeling the vibration or "buzzing".*
- *Repeat for the great toe on the other foot*

Vibration Perception Grade Scale:
0 – Vibration felt for >10 seconds (normal)
1 – Vibration felt for 6-10 seconds (mild loss)
*2 – Vibration felt for 5 seconds or less (moderate loss)**
*3 – No feeling of vibration (severe loss)**
*9 – Unable to evaluate or did not assess**

	Right	Left
7. Measured vibration grade of great toe DIP joint		

DEEP TENDON REFLEXES

- *The examiner uses one hand to press upward on the ball of the foot, dorsiflexing the Subject's ankle to 90 degrees. Using the reflex hammer (preferably long handled), the examiner strikes the Achilles tendon.*
- *The tendon reflex is felt by the examiner's hand as plantar flexion of the foot, appearing after a slight delay from the time the Achilles tendon was struck.*
- *Repeat for ankle on other leg*

Ankle reflex grade scale:
0 – Absent
1 – Hypoactive
2 – Normal deep tendon reflexes
3 – hyperactive deep tendon reflexes ***(e.g. with prominent spread of toes)***
4 – clonus
9 – unable to evaluate or did not assess

	Right	Left
8. Measured ankle reflex grade		

COMMENTS

Name of Person Completing Form									Name of Clinician (if required)									
Signature of Person Completing Form									Signature of Clinician (if required)									
Date	D	D	M	M	M	Y	Y	Y	Y	Date	D	D	M	M	M	Y	Y	Y	Y

APPENDIX 7 TUBERCULOSIS SYMPTOM PROFILE (V3)

TUBERCULOSIS SYMPTOM PROFILE (V3)

This questionnaire asks about symptoms that patients with tuberculosis may or may not experience.

Please read each symptom carefully and think about your experience **during the past 7 days** when you make your response. Then tick (☑) one box for each symptom.

If you **did not** experience the symptom **during the past 7 days**, please tick (☑) "None" for that symptom.

If you **did** experience the symptom **during the past 7 days**, please tick (☑) whether the intensity of the symptom you experienced was "Mild", "Moderate" or "Severe".

TB Symptom	Rate your experience of each symptom over the past 7 days.			
Feeling feverish	☐ None	☐ Mild	☐ Moderate	☐ Severe
Feeling chills	☐ None	☐ Mild	☐ Moderate	☐ Severe
Excessive sweating	☐ None	☐ Mild	☐ Moderate	☐ Severe
Shortness of breath	☐ None	☐ Mild	☐ Moderate	☐ Severe
Chest pain	☐ None	☐ Mild	☐ Moderate	☐ Severe
Feeling unwell	☐ None	☐ Mild	☐ Moderate	☐ Severe
Tiredness/weakness	☐ None	☐ Mild	☐ Moderate	☐ Severe
Cough	☐ None	☐ Mild	☐ Moderate	☐ Severe
Coughing up mucus	☐ None	☐ Mild	☐ Moderate	☐ Severe
Coughing up blood	☐ None	☐ Mild	☐ Moderate	☐ Severe

During the past 7 days, how would you rate your appetite?

☐ Good ☐ Fair ☐ Poor

Approved, Issued Date 09-Apr-2012

APPENDIX 8 LIVER TOXICITY MANAGEMENT GUIDELINES

Standard anti-TB chemotherapy is known to cause derangement of liver function tests in a substantial number of patients. In many cases, these will be asymptomatic and self-limiting. In some cases, severe hepatitis and even fulminant liver failure and death can occur.

In pre-marketing clinical trials of new drugs and regimens, it is especially important to identify and carefully manage any trial subjects who are at risk of progressing to serious liver injury. The observation of altered liver function to a degree with a high risk of progressing further to liver failure has been referred to informally as *Hy's Law* (Temple 2001; Reuben 2004); this reflects pure hepatocellular injury sufficient to cause hyperbilirubinemia is an ominous indicator of the potential for a drug to cause serious liver injury. Briefly, Hy's Law cases have the following three components:

1. The drug causes hepatocellular injury, generally shown by a higher incidence of 3-fold or greater elevations above the ULN of ALT or AST than the (non-hepatotoxic) control drug or placebo;
2. Among trial subjects showing such aminotransferase (AT) elevations, often with ATs much greater than 3x ULN, one or more also show elevation of serum total bilirubin (TBL) to >2x ULN, without initial findings of cholestasis (elevated serum ALP) ;
3. No other reason can be found to explain the combination of increased AT and TBL, such as viral hepatitis A, B, or C; pre-existing or acute liver disease; or another drug capable of causing the observed injury.

In a clinical trial of new drugs and combinations, it is especially important for Investigators to closely follow any Subjects who have evidence of potential hepatic inflammation or toxicity. During this trial, liver function will be monitored regularly via clinical assessments and blood tests to assist in determining which follow up laboratory measurements will either document resolution of abnormalities or signal the potential for drug-induced liver injury (DILI). The following procedure describes the management of deranged liver function tests.

Procedure

Blood tests for liver function will be taken routinely at Screening (Days -9 to -1), at the specific visits deisgnated in the protocol and at Early Withdrawal. If at any other visit, the Investigator suspects derangement of liver function (e.g. the Subject describes nausea and vomiting, right upper abdominal pain or is jaundiced), blood should be taken for liver function tests and the Subject comprehensively assessed for evidence of hepatitis, hepatic impairment and any potentially contributing cause(s).

The laboratory source (print-out of any results) should be stored alongside or transcribed into the clinical source document. Each abnormal value should be marked as clinically significant (CS) or non-clinically significant (NCS); the assessment of significance is at the discretion of the Investigator. All abnormal results that are clinically significant must be recorded as Adverse Events in the eCRF and graded clinically per the DMID Adult Toxicity Table (Appendix 2).

Assessments and decision making for elevations in aminotransferase values or bilirubin of various levels of concern are detailed below:

Decision to Consider Stopping Drug Regimen Administration

Consideration of stopping drug administration, at least temporarily, to subjects with liver function abnormalities or signs and symptoms of hepatitis should be discussed with the Sponsor Medical Monitor in the following situations:

- ALT or AST >8x ULN;
- ALT or AST >5x ULN for more than 2 weeks;
- ALT or AST >3x ULN with the appearance of fatigue, nausea, vomiting, right upper quadrant pain or tenderness, fever, rash, and/or eosinophilia (>5%).

If a subject has ALT or AST ≥3x ULN ***and*** *Total Bilirubin >2x ULN, the IMP should be interrupted and the Subject's clinical course discussed with the Sponsor Medical Monitor.*

More detailed assessments and decision making for various levels of elevations in aminotransferase values, alkaline phosphatase or bilirubin are detailed below:

Grade 3 per DMID, ALT, AST, AP ≥ 3x ULN to 8x ULN or if a substantial increase from baseline (such as > 2-fold increase):

- Contact the Subject and recall them as soon as possible. Assess the Subject for other signs and symptoms of more specific hepatic events including hepatic impairment and/or hepatitis. If you are concerned, you should consider arranging for the subject to present to a medical facility (e.g. emergency department) immediately for assessment.
- Assess the clinical significance - if the Subject has jaundice, a coagulation disorder or signs of hepatic encephalopathy, all study medication should be withheld pending assessment/improvement.
- Assess possible contributing factors – This should include (but is not limited to), alcohol, intra-venous and other drug use, travel, unwell contacts, any medications with known hepatotoxic potential, herbal products and dietary supplements, previous or known hepatitis infection and exposure to environmental chemical agents. Although anti-TB chemotherapy is known to cause liver function test derangement, the Subject should always be assessed for other possible cause(s) or contributing factor(s).
- The Subject should also be advised to stop taking any medications/substances, other than the study medications used to treat TB that may be contributing to or causing derangement of liver function tests.
- Make every effort to repeat the testing of ALT, AST, AP and bilirubin within 48-72 hours to confirm the abnormalities and determine if they are increasing or decreasing. Consider any additional laboratory tests that may help characterize the Subject's clinical condition. Subjects should be tested for viral hepatitis (e.g. hepatitis A and B and any other tests available of viral hepatitis). If tests for viral hepatitis are not available or done, it may still be helpful to collect an additional 10ml sample for serum for freezing (5ml yellow/SST tube x2) which may be tested later. The Subject's consent must be obtained for this.

Elevated liver enzymes considered to be of clinical significance but not accompanied by other signs and symptoms, should be reported as an adverse event and recorded as elevated liver enzymes in the eCRF. If the term "hepatitis" is used, the Safety Data Manager will question the site for additional evidence to support the diagnosis, such as clinical signs, serological or biopsy data. While a liver biopsy is not required to make a diagnosis of hepatitis, the term "hepatitis" should be reserved in most instances for cases where there is supportive evidence beyond a liver enzyme abnormality. However, if the investigator confirms the diagnosis of hepatitis solely on the basis of clinical signs and laboratory values, the diagnosis will be accepted. Should other symptoms or signs be present, these should also be recorded as adverse events in the eCRF.

If ALT, AST, AP are Grade 4 per DMID (> 8x ULN):

- Contact the Subject and recall them as soon as possible. Generally, the trial medication should be stopped, but this should be discussed first with the Sponsor Medical Monitor whenever possible. Assess the subject for other signs and symptoms of more specific hepatic events, including hepatic impairment and/or hepatitis. If you are concerned, you should consider arranging for the subject to present to a medical facility (e.g. emergency department) immediately for assessment.
- Assess the clinical significance – Consider hospitalisation if the ALT is more than 10 times the ULN and/or the Subject has jaundice, a coagulation disorder or signs of hepatic encephalopathy. All study medications should be withheld pending assessment/improvement.
- Assess possible contributing factors – This should include (but is not limited to), alcohol, intra-venous and other drug use, travel, unwell contacts, any medications with known hepatotoxic potential, herbal products and dietary supplements, previous or known hepatitis infection and exposure to environmental chemical agents. Although anti-TB chemotherapy is known to cause liver function test derangement, the subject should always be assessed for other possible cause(s) or contributing factor(s).
- Make every effort to repeat the testing of ALT, AST, AP and bilirubin within 48-72 hours to confirm the abnormalities and determine if they are increasing or decreasing. Consider any additional laboratory tests that may help characterize the subject's clinical condition. Subjects should be tested for viral hepatitis (e.g. hepatitis A and B and any other tests available of viral hepatitis). If tests for viral hepatitis are not available or done, it may still be helpful to collect an additional 10ml sample for serum for freezing (5ml yellow/SST tube x2) which may be tested later. The Subject's consent must be obtained for this.

Elevated liver enzymes considered to be of clinical significance but not accompanied by other signs and symptoms, should be reported as an adverse event and recorded as elevated liver enzymes in the eCRF. If the term "hepatitis" is used, the Safety Data Manager will question the site for additional evidence to support the diagnosis, such as clinical signs, serological or biopsy data. While a liver biopsy is not required to make a diagnosis of hepatitis, the term "hepatitis" should be reserved in most instances for cases where there is supportive evidence beyond a liver enzyme abnormality. However, if the investigator confirms the diagnosis of hepatitis solely on the basis of clinical signs and laboratory values, the diagnosis will be accepted. Should other symptoms or signs be present, these should also be recorded as adverse events in the eCRF.

General Principles for following Subjects with potential liver toxicity

The Subject should be contacted regularly depending on the Grade of LFT elevations and the magnitude of increase relative to baseline for the Subject. Initially, this should be daily and subsequently depends on clinical course/individual circumstances. Staff must ensure all Subjects know to seek medical attention urgently if they experience any evidence of worsening liver disease. Symptoms may include (but are not limited to), malaise, fever, nausea, vomiting, loss of appetite, dark urine, yellowing of the eyes or skin (jaundice).

Liver function tests should be repeated regularly, such as every 3 days for the first week, then once a week until they return to near baseline values for the Subject. Manage the Subject symptomatically as required using medications that are not potentially hepatotoxic. Infection control issues must be carefully managed whilst TB medications are being withheld, especially if the Subject is still culture positive for acid fast bacilli.

Restarting Medication

If the Investigator (after consultation with the Sponsor Medical Monitor), stops administration of the study medication, consideration may be given to re-starting the study medication. Once the liver function values have decreased substantially and and symptoms have significantly improved, a decision must be made about further TB management. This will be dependent on clinical context and the decision must be made in discussion with the Sponsor Medical Monitor. In all cases, treatment should be recommenced under close supervision for any evidence of recurrent liver function abnormalities.

If there is a further significant elevation of hepatic enzymes or bilirubin or symptoms of clinical concern after resumption of study medication, the study medication should be withdrawn permanently. Subjects who permanently discontinue study medication should be managed as clinically indicated according to local National TB Programme guidelines. The Sponsor Medical Monitor can provide advice and examples of suitable treatment regimens to use if required.

CONFIDENTIAL

PROTOCOL

Protocol Title: A Phase 3 open-label trial assessing the safety and efficacy of bedaquiline plus pretomanid plus linezolid in Subjects with pulmonary infection of either extensively drug-resistant tuberculosis (XDR-TB) or treatment intolerant / non-responsive multi-drug resistant tuberculosis (MDR-TB).

Protocol Number: Nix-TB-(B-L-Pa)

Working Protocol Version: 5.0 (FINAL)

Working Protocol Date: 16 FEB 2018

COMBINATION OF THE FOLLOWING APPROVED FINAL DOCUMENTS:
Protocol V1.0 dated 21 April 2014
Protocol V2.0 dated 18 March 2015 Protocol V3.0 dated 22 JAN 2016
Protocol V4.0 dated 24 April 2017

PROTOCOL SIGNATURE PAGE

Protocol Title: A Phase 3 open-label trial assessing the safety and efficacy of bedaquiline plus pretomanid plus linezolid in Subjects with pulmonary infection of either extensively drug-resistant tuberculosis (XDR-TB) or treatment intolerant / non-responsive multi-drug resistant tuberculosis (MDR-TB).

Protocol Number: Nix-TB (B-L-Pa)

Protocol Version: 5.0

Protocol Date: 16 FEB 2018

SPONSOR

I agree to the terms of this study protocol.

DocuSigned by:

Signer Name: Dan Everitt
Signing Reason: I approve this document
Signing Time: 4/9/2018 7:40:34 PM EDT
32534894D9294A59B14B10FC37E90452

Signature of Senior Medical Officer	Daniel Everitt, MD<br>Printed Name
April 9, 2018 | 7:40 PM EDT<br>Date	

CO-ORDINATING INVESTIGATOR

I agree to the terms of this trial protocol. I will conduct the trial according to the procedures specified herein and in accordance to the principals of current Good Clinical Practice (cGCP) and local regulations.

DocuSigned by:

Signer Name: Francesca Conradie
Signing Reason: I approve this document
Signing Time: 4/10/2018 4:07:57 AM EDT
8F3C422D6DE04C72AD395BC608A22CC5

Signature	Francesca Conradie, MD<br>Printed Name
April 10, 2018 | 4:08 AM EDT<br>Date	

PRINCIPAL INVESTIGATOR PROTOCOL SIGNATURE PAGE

Protocol Title: A Phase 3 open-label trial assessing the safety and efficacy of bedaquiline plus pretomanid plus linezolid in Subjects with pulmonary infection of either extensively drug-resistant tuberculosis (XDR-TB) or treatment intolerant / non-responsive multi-drug resistant tuberculosis (MDR-TB).

Protocol Number: Nix-TB (B-L-Pa)

Protocol Version: 5.0

Protocol Date: 16 FEB 2018

I hereby confirm that I have read the above protocol and agree to conduct this clinical trial as outlined in the above protocol. I will provide copies of the protocol and access to all the information required to conduct the clinical trial according to the above protocol to the site personnel under my supervision. I will discuss this material with them and ensure they are fully informed on all trial requirements.

Signature

Printed Name

Date

Development Phase	3
Sponsor	Global Alliance for TB Drug Development<br>40 Wall Street, 24th Floor<br>New York, NY 10005<br>United States of America
Medical Monitor	Name: Morounfolu Olugbosi, MD<br>Address: Global Alliance for TB Drug Development<br>Boardwalk Office Park<br>Ground Floor, Block 1, Suite5<br>107 Haymeadow Street<br>Faerie Glen 0043<br>Pretoria, South Africa<br>Telephone: +27 87 700 3900<br>Mobile: +27 79 045 4917<br>Facsimile: +27 12 991 3045<br>Email:Morounfolu.Olugbosi@tballiance.org
Co-ordinating Investigator	Name: Francesca Conradie<br>Address: CHRU Themba Lethu Clinic<br>Helen Joseph Hospital<br>No. 8 Perth Road<br>Westdene<br>Johannesburg<br>South Africa<br>Telephone: +27 11 531 4347<br>Mobile: +27 83 357 7611<br>Facsimile: +27 11 882 3912<br>Email: fconradie@witshealth.co.za

TABLE OF CONTENTS

1. PROTOCOL SYNOPSIS 14
1.1. Synopsis 14
1.2. Trial Flow Chart 18
2. INTRODUCTION 21
2.1. Background 21
2.2. Agents to be Studied 22
2.2.1. Bedaquiline 22
2.2.1.1. Bedaquiline Preclinical Studies 23
2.2.1.2. Bedaquiline Clinical Studies 24
2.2.1.3. Bedaquiline Clinical Safety 25
2.2.2. Pretomanid 28
2.2.2.1. Pretomanid Preclinical Studies 28
2.2.2.2. Pretomanid Clinical Studies 30
2.2.2.3. Pretomanid Clinical Safety 36
2.2.3. Linezolid 40
2.3. Regimens to be Studied 43
2.3.1. Non-Clinical Studies 44
2.3.2. Clinical Study NC-001 45
2.3.3. Clinical Study NC-003 46
2.4. Overall Benefit/Risk Assessment 47
3. TRIAL RATIONALE AND OBJECTIVES 48
3.1. Trial Rationale 48
3.2. Dose Rationale 48
3.2.1. Bedaquiline 48
3.2.2. Pretomanid 48
3.2.3. Linezolid 48
3.3. Trial Objectives 49
4. TRIAL DESIGN 49
4.1. Summary 49
4.2. Trial Endpoints 50
4.2.1. Primary Endpoint 50
4.2.2. Secondary Endpoints 50
4.2.2.1. Efficacy: 50

4.2.2.2. Safety and Tolerability: ... 51
4.2.2.3. Pharmacokinetics: ... 51
4.2.2.4. Exploratory ... 51
4.2.2.5. General Mycobacteriology ... 52
4.3. Trial Population ... 53
4.3.1. Inclusion Criteria ... 53
4.3.2. Exclusion Criteria ... 54
4.4. Treatment Plan: Schedule of Assessments ... 57
4.4.1. Screening (Up to 9 Days Prior to Treatment) ... 57
4.4.1.1. Screening ... 57
4.4.2. Treatment Period (Day 1 to Week 26 or Week 39) ... 58
4.4.2.1. Day 1 ... 58
4.4.2.2. Week 1 ... 58
4.4.2.3. Week 2 ... 58
4.4.2.4. Weeks 3, 5, 7, 9, 10, 11, 13, 14 and 15 ... 59
4.4.2.5. Week 4 ... 59
4.4.2.6. Week 6 ... 59
4.4.2.7. Week 8 ... 59
4.4.2.8. Week 12 ... 60
4.4.2.9 Week 16 (Week 30 when applicable) ... 60
4.4.2.10 Week 20 (Week 34 when applicable) ... 60
4.4.2.11 Week 26 (Week 39 when applicable) ... 61
4.4.2.12 Early Withdrawal ... 61
4.4.3 Follow-Up Period ... 62
4.4.3.1 1 Month Post-Treatment ... 62
4.4.3.2 2 Months Post-Treatment ... 62
4.4.3.3 3 Months Post-Treatment or Withdrawal ... 63
4.4.3.4 Months 6, and 24 Post-Treatment ... 63
4.4.3.5 Months 9, 15, 18 and 21 Post-Treatment ... 63
4.4.3.6 Unscheduled Visits ... 63
4.5 Treatment Discontinuation and Subject Withdrawal ... 64
4.6 Temporary Dose Interruptions and Modifications ... 65
4.7 Stopping Rules ... 66

4.8 Subject Progress Definitions 66
4.8.1 Enrolment 66
4.8.2 Completed Trial 66
4.8.3 Withdrawn 67
4.9 Restrictions 67
4.9.1 Prior and Concomitant Medications and Other Treatments 67
4.9.1.1 Recommendations for Concomitant use of Anti-Malarials 68
4.9.1.2 Antiretroviral Therapy 68
4.9.1.3 Other Restrictions 68
5 INVESTIGATIONAL MEDICINAL PRODUCT 68
5.1 Trial Treatments 68
5.2 Method of Assigning Subjects to Study Treatment 69
5.3 IMP Administration 69
5.4 Subject Compliance 69
5.5 Blinding and Procedures for Breaking the Blind 69
5.6 IMP Packaging and Labelling 69
5.13 .... **Error! Bookmark not defined.**
5.6.1 Packaging 70
5.6.2 Storage 71
5.7 Dispensing and Accountability 71
5.8 Returns and Destruction 71
6 TRIAL VARIABLES AND PROCEDURES 71
6.1 Demographic and Background Variables and Procedures 71
6.2 Efficacy Variables and Procedures 72
6.3 Safety and Tolerability Variables and Procedures 72
6.4 Pharmacokinetic Variables and Procedures 74
6.5 Mycobacteriology Characterization Variables and Procedures 74
7 ADVERSE EVENTS 74
7.1 Definitions 75
7.1.1 Adverse Event (AE) 75
7.1.2 Serious Adverse Event (SAE) 75
7.1.3 Unlisted (Unexpected) Adverse Event 75
7.1.4 Life Threatening 75
7.1.5 Hopsitalization 75
7.1.6 Associated with the Use of the Drug 76

7.1.7 Attribution/Causality ... 76
7.1.8 Severity ... 77
7.1.9 Other AE Definitions ... 77
7.2 Reporting ... 77
7.2.1 Adverse Event (AE) ... 78
7.2.2 Serious Adverse Event (SAE) ... 78
7.2.3 Follow up of Adverse Events ... 78
7.2.4 Post-Trial Adverse Events ... 79
7.2.5 Clinical Laboratory Adverse Events ... 79
7.2.6 Disease under Study ... 79
7.2.7 Overdose ... 79
7.2.8 Drug Interaction ... 79
7.2.9 Pregnancy ... 79
7.3 Monitoring and Safety for Specific Toxicities ... 80
7.3.1 ALT, AST and Alkaline Phosphatase elevations: ... 80
7.3.2 Amylase elevation ... 80
7.3.3 Lipase Elevation ... 81
7.3.4 Musculoskeletal System and Cardiac Muscle ... 81
7.3.5 Cardiac Rhythm Disturbances ... 81
7.3.6 Myelosuppression ... 81
7.3.7 Peripheral Neuropathy ... 82
7.3.8 Optic Neuropathy ... 82
7.3.9. Lactic Acidosis ... 82
7.3.10 Neurological ... 82
7.4 Safety Monitoring by the Data Safety Monitoring Committee ... 83
8 STATISTICAL ANALYSIS ... 83
8.1 Analysis Population ... 83
8.2 Sample Size ... 83
8.3 Interim Analyses ... 83
8.4 Primary Endpoint Analysis ... 83
8.5 Secondary Endpoint Analysis ... 84
8.5.1 Efficacy ... 84
8.6 Exploratory Endpoint Analysis ... 84
8.6.1 Efficacy ... 84
8.6.2 Safety and Tolerability Analysis ... 84

8.7 Pharmacokinetics: 85
8.8 Pharmacokinetics-Pharmacodynamics (PK-PD): 86
8.9 General Mycobacteriology 86
9 RECORDS MANAGEMENT 86
9.1 Data Collection 86
9.2 Source Documents 86
9.3 File Management at the Trial Centre 86
9.4 Records Retention at the Trial Centre 86
10 QUALITY CONTROL AND ASSURANCE 86
10.1 Site Procedures 86
10.2 Monitoring 87
10.3 Auditing 87
11 ETHICS AND REGULATORY 87
11.1 Basic Principles 88
11.2 Independent Ethics Committee/Institutional Review Board (IEC/IRB) Review 88
11.3 Regulatory Authorities 88
11.4 Informed Consent 88
11.5 Confidentiality 88
12 PUBLICATION POLICY 89
13 PROTOCOL AMENDMENT POLICY 89
14 FINANCIAL ASPECTS, INSURANCE AND INDEMNITY 89
15 REFERENCES 90
APPENDIX 1 THE IUATLD SCALE 93
APPENDIX 2 DIVISION OF MICROBIOLOGY AND INFECTIOUS DISEASES (DMID) ADULT TOXICITY TABLE 94
APPENDIX 3 VITAL SIGNS 103
APPENDIX 4 KARNOFSKY PERFORMANCE STATUS SCALE DEFINITIONS RATING (%) CRITERIA[19] 104
APPENDIX 5 EQ-5D-5L Questionnaire 105
APPENDIX 6 Brief Peripheral Neuropathy Screening 107
APPENDIX 7 TUBERCULOSIS SYMPTOM PROFILE (V3) 109
APPENDIX 8 LIVER TOXICITY MANAGEMENT GUIDELINES 110

TABLES

Table 1: Bedaquiline Adverse Drug Reactions 26

Table 2: Pretomanid Key Animal Safety and Toxicology Findings 30

Table 3: Pretomanid Phase 1 Clinical Studies 32

Table 4: Pretomanid Phase 2 Studies 35

Table 5: NC-002 Elevations in Alanine Aminotransferase 39

Table 6: Murine Lung CFU counts during Treatment with Linezolid monotherapy versus Standard Therapy 40

Table 7: Lung CFU counts assessed during Treatment and proportion of mice relapsing after treatment completion...... 44

Table 8: Murine Relapse Data, Impact of L Treatment Duration Lung CFU counts assessed during treatment and proportion of mice relapsing after treatment completion 45

Table 9: Summary Statistics for $EBACFU_{(0-14)}$ Derived Using Bi-Linear Regression, Study NC-001. 46

Table 10: NC-003 Efficacy Results: Daily $BAlogCFU_{(0-14)}$ 46

Table 11: NC-003 Safety Data 47

Table 12: General Mycobacteriology 52

Table 13: Adverse Events Attribution/Causality Ratings 76

Table 14: Adverse Event Severity Ratings 77

FIGURES

Figure 1: Trial Schematic 50

LIST OF ABBREVIATIONS AND DEFINITIONS OF TERMS

ADME	Absorption, Distribution, Metabolism, and Excretion
AE	Adverse Event
AIDS	Acquired Immune Deficiency Syndrome
ALP	Alkaline Phosphatase
ALT	Alanine Aminotransferase
AREDS2	Age Related Eye Disease Study 2
ART	Antiretroviral Therapy
AST	Aspartate Aminotransferase
AUC	Area under the plasma concentration time curve
$AUC_{(0-24)}$	Area under the plasma concentration time curve from zero to end of dosing interval
$AUC_{(0-t)}$	Area under the PK plasma concentration time (t) curve from zero to the last quantifiable PK plasma concentration prior to the subsequent dose, using the linear trapezoidal rule
BA	Bactericidal Activity
B	Bedaquiline (formerly J, TMC-207)
BID	Twice daily dosing
BMI	Body Mass Index
bpm	Beats per minute
BUN	Blood urea nitrogen
C	Clofazimine
°C	Degrees Celsius
CFU	Colony Forming Units
CK	Creatine Phosphokinase
CK-MB	Creatine Phosphokinase of Muscle Brain
C_{max}	Maximum observed plasma concentration
C_{min}	Minimum observed plasma concentration at the end of the dosing interval
CNS	Central Nervous System
CYP3A4	Cytochrome P450 3A4
DBP	Diastolic Blood Pressure
DDI	Drug-Drug Interactions
DMID	Division of Microbiology and Infectious Diseases
DNA	Deoxyribonucleic acid
DOTS	Directly Observed Treatment, Short Course
DS	Drug-Sensitive
DSMC	Data Safety Monitoring Committee
DST	Drug Sensitivity Testing
eCRF	Electronic Case Report Form
EBA	Early Bactericidal Activity
ECG	Electrocardiogram
ELISA	Enzyme-Linked Immunosorbent Assay
EMA	European Medicines Agency
ERPF	Effective Renal Plasma Flow
FDA	United States Food and Drug Administration

FF	Filtration Fraction
FSH	Follicle-Stimulating Hormone
GCP	Good Clinical Practice
GFR	Glomerular Filtration Rate
GGT	Gamma-glutamyltransferase
hERG	Human ether-à-go-go-related gene
HIV	Human Immunodeficiency Virus
hr	Hour
HRZE	isoniazid plus rifampicin plus pyrazinamide plus ethambutol
HRZM	Isoniazid plus rifampicin plus pyrazinamide plus moxifloxacin
IB	Investigator Brochure
IC_{50}	50% inhibitory concentration
ICF	Informed Consent Form
ICH	International Conference on Harmonization
IMP	Investigational Medicinal Product
IUATLD	International Union Against Tuberculosis and Lung Disease
i.v., IV	Intravenous
Kg	Kilogram
LDH	Lactate Dehydrogenase
L	Linezolid
LFT	Liver Function Test
lKr	Delayed rectifier potassium current
LH	Luteinizing Hormone
LSLV	Last Subject Last Visit
m	Meters
M	Moxifloxacin
MAOI	Monoamine Oxidase Inhibitor
MBD	Minimum Bactericidal Dose
M2	Bedaquiline metabolite M2
MDR	Multi Drug-Resistant
MED	Minimum Effective Dose
mg	Milligrams
mg/dl	milligram per decilitre
MGIT	Mycobacterial Growth Indicator Tube
MIC	Minimum inhibitory concentration
ml	Millilitre
mmHg	Millimeter of mercury
M. tb.	*Mycobacterium tuberculosis*
ms	Millisecond
NIH	National Institute of Health
NLME	Non-linear Mixed Effect
NOAEL	No Observed Adverse Effect Level
Pa	Pretomanid (formerly PA-824)

PD	Pharmacodynamic
PE	Physical Examination
PK	Pharmacokinetic
PR	Electrocardiographic PR interval
q.d./QD	Once daily dosing
QRS	Electrocardiographic QRS interval
QT	Electrocardiographic QT interval
QTc	Corrected QT interval
QTcB	QT interval corrected by Bazett's method
QTcF	QT interval corrected by Fridericia's method
RR	Electrocardiographic RR interval
SAE	Serious Adverse Event
SAP	Statistical Analysis Plan
SBP	Systolic Blood Pressure
sc	Subcutaneous
SIRE	Streptomycin, Isoniazid, Rifampicin and Ethambutol
SSCC	Serial Sputum Colony Counts
T	Time
$t_{½}$	Apparent terminal elimination phase half-life
TB	Tuberculosis
TEAEs	Treatment-Emergent Adverse Events
TIW	Three times a week
T_{max}	Time at which C_{max} is observed
TMIC	Time over Minimum Inhibitory Concentrations
TTP	Time to Sputum Culture Positivity
UA	Uric Acid
ULN	Upper Limit of Normal
µg/ml	microgram per millilitre
WBC	White Blood Cell
WHO	World Health Organization
XDR	Extensively drug-resistant
Z	Pyrazinamide

1. PROTOCOL SYNOPSIS

1.1. Synopsis

**Name of Sponsor/Company:**	Global Alliance for TB Drug Development
**Name of Finished Products:**	bedaquiline tablets; pretomanid tablets; linezolid tablets.
**Protocol Title:**	A Phase 3 open-label trial assessing the safety and efficacy of bedaquiline plus pretomanid plus linezolid in Subjects with pulmonary infection of either extensively drug-resistant tuberculosis (XDR-TB) or treatment intolerant / non-responsive multi-drug resistant tuberculosis (MDR-TB)
**Treatment Indication:**	Pulmonary XDR-TB and treatment intolerant/non-responsive MDR-TB
**Trial Objective:**	To evaluate the efficacy, safety, tolerability and pharmacokinetics of bedaquiline plus pretomanid plus linezolid after 6 months of treatment (option for 9 months for subjects who remain culture positive or revert to being culture positive between month 4 and month 6 visits) in Subjects with either pulmonary XDR tuberculosis, treatment intolerant or non-responsive multi-drug resistant tuberculosis (MDR-TB).
**Trial Design:**	An open-label clinical trial.<br><br>Treatment:<br>• bedaquiline 400 mg once daily for 2 weeks then 200mg 3 times per week plus pretomanid 200mg once daily plus linezolid 1200mg once daily. All IMP to be given with a meal.<br><br>Treatment Duration:<br>• 6 months<br>• If subjects are still culture positive at month 4, option to extend treatment to 9 months or withdraw.<br><br>Follow-Up:<br>• Subjects who complete treatment will return for follow-up visits 1, 2 and 3 months after end of treatment then every 3 months up to 24 months after end of treatment.<br>• Subjects who withdraw after ≤14 days of IMP administration are to return for an Early Withdrawal visit only;<br>• Subjects who withdraw after ≥15 days of IMP are to return for the Early Withdrawal, and for the 3, 6, and 24 month follow up visits after their last dose of IMP.<br><br>Data Safety Monitoring Committee (DSMC) Reviews:<br>Interim Safety/Efficacy data will be reviewed by DSMC as follows:<br>• At least every 6 months after the first subject is enrolled;<br>• Ad hoc meetings can be called by Sponsor/DSMC based on rates of SAEs or to review results of futility analysis or if safety concerns arise during the trial.
**Patient Population:**	A total of up to 200 male or female Subjects aged 14 and over with confirmed sputum culture-positive pulmonary XDR-TB or MDR-TB with a documented intolerability or nonresponse to treatment.

**Name of Sponsor/Company:**	Global Alliance for TB Drug Development
**Name of Finished Products:**	bedaquiline tablets; pretomanid tablets; linezolid tablets.
**Test Product, Dose and Mode of Administration:**	The Investigational Medicinal Product (IMP) will be supplied as:<br>• Bedaquiline 100mg tablets<br>• Pretomanid 200mg tablets<br>• Scored Linezolid 600 mg tablets<br><br>The assigned treatment regimen will be administered orally for 6 months (possibly 9 months) at the following doses and intervals:<br>• Bedaquiline 400 mg once daily for 2 weeks then 200 mg 3 times per week; plus pretomanid 200mg once daily; plus linezolid 1200mg once daily.<br><br>A reduction in the dose of linezolid (to either 600 mg qd or 300 mg qd) or temporary cessation of linezolid (due to a linezolid-specific toxicity), or of the full regimen per Investigator discretion will be allowed for suspected drug related toxicity. Re-introduction of the regimen could be considered post a cessation not greater than 35 consecutive days.<br><br>If subjects have toxicity issues with linezolid prohibiting further treatment with that drug, they can remain on the bedaquiline and pretomanid study IMP if they received the initial total of 1200 mg daily dose of linezolid for at least the first 4 consecutive weeks of treatment and they are smear negative, or with trace/scanty results and judged to be clinically improving by the Investigator.

Criteria for Evaluation:

Primary Endpoint:
Incidence of bacteriologic failure or relapse or clinical failure through follow up until 6 months after the end of treatment.

Abbreviated Definitions (full definitions will be described in the Statistical Analysis Plan (SAP)):

- Bacteriologic failure: During the treatment period, failure to attain culture conversion to negative.
- Bacteriologic relapse: During the follow-up period, failure to maintain culture conversion to negative status in culture, with culture conversion to positive status with a Mycobacterium tuberculosis (*M.tb.*) strain that is genetically identical to the infecting strain at baseline.
- Clinical failure: A change from protocol-specified TB treatment due to treatment failure, retreatment for TB during follow up, or TB-related death.

Note:

- Culture conversion requires at least 2 consecutive culture negative/positive samples at least 7 days apart.
- Subjects who are documented at a visit as unable to produce sputum and who are clinically considered to be responding well to treatment will be considered to be culture negative at that visit.

Secondary Endpoints:

- Incidence of bacteriologic failure or relapse or clinical failure through follow up until 24 months after the end of treatment as a confirmatory analysis.
- Time to sputum culture conversion to negative status through the treatment period.
- Proportion of subjects with sputum culture conversion to negative status at 4, 6, 8, 12, 16 and 26 or 39 weeks.
- Linezolid dosing (actual) and efficacy will be explored.
- Change from baseline TB symptoms.
- Change from baseline in Patient Reported Health Status.
- Change from baseline weight.

**Name of Sponsor/Company:**	Global Alliance for TB Drug Development
**Name of Finished Products:**	bedaquiline tablets; pretomanid tablets; linezolid tablets.

Safety and Tolerability:

- All cause mortality.
- Incidence of Treatment Emergent Adverse Events (TEAEs) will be presented by severity (DMID Toxicity Grade), drug relatedness and seriousness, leading to early withdrawal and leading to death.
- Quantitative and qualitative clinical laboratory result measurements, including observed and change from baseline.
- Quantitative and qualitative measurement of ECG results, including observed and change from baseline.
- Descriptive statistics of ophthalmology slit lamp examination data (age related eye disease study 2 [AREDS2] lens opacity classification and grading). Categorical data for lens opacity will be summarized in a frequency table for the right and left eye, respectively, including change from baseline.
- Changes in ophthalmic exam for visual acuity and color vision, including observed and change from baseline.
- Changes noted in peripheral neuropathy signs and symptoms, including observed and change from baseline.
- These data will be presented as descriptive analyses, and no inferential tests will be carried out.

Pharmakokinetics (PK):

Pharmacokinetics will consist of two separate schedules:

- All Subjects- Pre-dose sampling at weeks 2, 8 and 16 to measure C_{trough} levels of bedaquiline, bedaquiline metabolite M2, linezolid and pretomanid.
- PK Sub-study Subjects- in addition to the C_{trough} samples, there will be intensive PK sampling at week 16 at pre-dose, 0.5, 1, 2, 4, 8 12, 12.5, 13, 14, 16, 20 and 24 hours after dosing in a sub-group of 20 evaluable Subjects across selected sites.

For the PK sub-study samples, the following PK parameters will be estimated from the individual (per Subject) PK plasma concentrations: Minimum observed PK plasma concentration (C_{min}), maximum observed PK plasma concentration(C_{max}), time to reach C_{max} obtained without interpolation (T_{max}), area under the PK plasma concentration time (t) curve from zero to the last quantifiable PK plasma concentration prior to the subsequent dose, using the linear trapezoidal rule ($AUC_{(0-t)}$), area under the PK plasma concentration time (t) curve from zero to 24 hours ($AUC_{(0-24)}$). Oral apparent clearance (CL/F) by non-compartment model. These will be derived for each analyte. In addition, for linezolid analyte BID dose, the AUC_{0-12}, C_{max}, C_{min}, CL/F and $t_{1/2}$ will be calculated based on dose interval 0-12 hrs.

Exploratory:

- Evaluate whether any of the secondary endpoints predicts relapse free cure.
- Subgroup analyses of the primary endpoint on the MITT analysis population will be considered
- Correlation of Time over mitochondrial protein synthesis inhibition (MPS50) with linezolid toxicity (The MPS50 will be an assumed value from the literature).

Mycobacteriology Characterization:

M.tb. isolates at baseline and initial relapse (first positive at end of treatment or during follow-up) will be processed at the central lab(s) for:

- MIC of bedaquiline, pretomanid and linezolid;
- Drug Susceptibility Testing in liquid culture for rifampicin, isoniazid, streptomycin, ethambutol, and second line TB drugs including fluoroquinolones and injectables;
- Extraction of bacterial (*M.tb*) DNA for molecular genotyping;

M.tb. isolates at baseline and initial relapse (first positive at end of treatment or during follow-up) or any positive at or after the week 16 visit will also be processed at the study lab (lab where study samples are initially sent from site for culture) for:

- Speciation of the infecting organism by molecular or antigen based test to confirm *M.tb*.

**Name of Sponsor/Company:**	Global Alliance for TB Drug Development
**Name of Finished Products:**	bedaquiline tablets; pretomanid tablets; linezolid tablets.

Statistical Methods:
The primary efficacy endpoint is treatment failure, defined as bacteriologic failure, or relapse, or clinical failure through follow-up until 6 months after the end of treatment. The probability of treatment failure through follow-up until 6 months after the end of treatment, as a function of time after assignment of study treatment, will be analyzed using Kaplan-Meier analysis. The binomial proportion for subjects with bacteriologic failure will be presented. No multiplicity adjustments for alpha will be done as this is an exploratory trial.

Futility Analysis:
Timing of initial interim analysis will be conducted when the first 15 participants reach 6 months after completion of IMP Further interim analyses will be specified in the statistical analysis plan (SAP).

Once all patients have been recruited or have completed the treatment period, no further futility analyses will be performed.

Trial Duration:
Estimated date of first Subject enrolled: Quarter 4 2014
Estimated date of last Subject enrolled: Quarter 3 2017
Estimated date of last Subject completed: Quarter 4 2020

Duration of Study:
~6 Years (An enrolment period of at least 42 months plus 9 days pre-treatment plus 6-9 month treatment period plus 24 months post treatment follow-up.

1.2. Trial Flow Chart

Period	Screening[a]	Treatment																			9 Month Treatment ONLY[p]				Post Treatment Follow-up Period[b]									
**Time of Visit**	Up to 9 days prior to Treatment	Day 1[c]	Week 1	Week 2	Week 3	Week 4	Week 5	Week 6	Week 7	Week 8	Week 9	Week 10	Week 11	Week 12	Week 13	Week 14	Week 15	Week 16	Week 20	Week 26	Week 30	Week 34	Week 39	Early Withdrawal (Treatment)	1 month	2 month	3 month	6 month	9 month	12 month	15 month	18 month	21 month	24 month
**Visit Window[q]**	N/A	+/- 3 days																	+/- 7 days						+/- 2 weeks									
**Informed Consent**	X																																	
**Demography**	X																																	
**Medical/Treatment History**	X																																	
**Inclusion/Exclusion**	X	X																																
**Karnofsky Assessment**	X																																	
**HIV Status[d]**	X																																	
**CD4 Count[e]**	X																																	
**Chest X-Ray[f]**	X																																	
**Serum or Urine Pregnancy Test[g]**	X	X								X										X[h]			X[h]	X										
**TB Symptoms Profile**	X									X										X[h]			X[h]	X						X				X
**Patient Reported Health Status**	X									X										X[h]			X[h]	X						X				X
**Slit Lamp Exam[i]**	X																			X[h]			X[h]	X			X							
**Ophthalmic Exam[j]**	X					X				X				X				X	X	X	X	X	X	X			X	X		X				X
**Vital Signs**	X	X	X	X		X		X		X				X				X	X	X	X	X	X	X			X	X	X	X	X	X	X	X
**Single 12-LeadECG**	X	X	X			X				X								X		X[h]	X		X[h]	X										
**Limited Physical Exam[k]**			X	X		X		X		X				X				X	X		X	X					X	X	X	X	X	X	X	X
**Full Physical Exam[k]**	X	X																		X[h]			X[h]	X										
**Laboratory Safety Tests[l]**	X	X	X	X		X		X		X				X				X	X	X	X	X	X	X										
**Con Meds**	X	X	X	X	X	X	X	X	X	X	X	X	X	X	X	X	X	X	X	X	X	X	X	X	X	X	X	X	X	X	X	X	X	X
**Adverse Events**	X	X	X	X	X	X	X	X	X	X	X	X	X	X	X	X	X	X	X	X	X	X	X	X	X	X	X	X	X	X	X	X	X	X
**Study Medication/Compliance[m]**		X	X	X	X	X	X	X	X	X	X	X	X	X	X	X	X	X	X	X	X	X	X	X										
**PK Sampling[n]**				X						X								X																
**Early Morning & Spot Sputum[o]**	X	X	X	X		X		X		X				X				X	X	X	X	X	X	X	X	X	X	X	X	X	X	X	X	X
**Complete/Full Blood Count**					X		X		X		X	X	X		X	X	X																	
**Peripheral Neuropathy**	X					X				X				X				X	X	X	X	X	X	X			X	X		X				X

Note:

- Unscheduled visits should be planned to assess, confirm, and follow up on clinically relevant AEs or laboratory abnormalities.
- All assessments are to be performed pre-dosing unless otherwise specified.
- On days where the following assessments are done the order should be: ECG before vital signs, blood draws (for Safety or PK).

a. **Screening:** Screening assessments can occur on different days within nine days prior to treatment. If a subject fails screening, a full re-screen (all screening procedures must be repeated) may occur at a later date.

b. **Follow-up Visits for Early Withdrawal Subjects:** Once a Subject has been permanently withdrawn from the trial, they will be required to attend an Early Withdrawal visit. If they receive/take less than 15 doses, additional visits are not required. If they received 15 or more doses, Month 3, Month 6 (if not already performed) and Month 24 follow-up visits are required. The Month 6 follow-up will be a full study visit as outline in section 4.4.3.4. Month 6(if already performed) and Month 24 visit will collect any AEs (Adverse Events), Serious Adverse Event (SAE), Concontimant medication information, including verification of survival and patient reported TB outcome information only and may be telephonic, a home or a site visit.

c. **Day 1 (baseline):** All procedures are to be completed prior to dosing.

d. **HIV testing:** If HIV status is a confirmed known positive, repeated HIV test is not needed provided documentation is available. If HIV status is unknown or suspected negative, HIV test should be requested. If an ELISA and/or Western Blot based HIV test was performed within 1 month prior to trial start, it should not be repeated as long as documentation of testing method and negative results can be provided.

e. **CD4 count:** For all HIV-positive Subjects.

f. **Chest X-Ray:** Chest X-Ray at Screening or within 1 year prior to Screening. The Investigator is responsible for its review and analysis for subject inclusion.

g. **Serum or Urine Pregnancy:** Women of child-bearing potential only, whether they are sexually active or not.

h. **Final Treatment Visit:** Serum or Urine Pregnancy Test, TB Symptoms Profile, Full Physical Examination, 12-lead ECG, Patient Reported Health Status and Slit Lamp Examination are only performed at the subjects' applicable End of Treatment Visit dependent on their treatment duration (week 26, week 39 or when final IMP completed for scenarios where interruptions extend treatment, as applicable). If Week 26 is not the Final Treatment Visit, only a limited physical exam should be done.

i. **Slit-Lamp Exam:** Slit Lamp examination will be performed by an Ophthalmologist with AREDS2 training. See section 4.4.2.12 and 4.4.3 for details on what follow-up slit lamp exams are necessary for subjects who withdrawal early.

j. **Ophthalmic Exam:** to include Ophthalmologic Medical history at Screening; All exams to include Visual Acuity and Color Vision assessment. Can be done by any trained study staff throughout study. Screening exam must be done by Ophthalmologist in addition to trained study staff that will perform exams throughout the study.

k. **Physical Exam: Full Physical Exams** to include gross neurological exam. All other PEs should be **limited** to weight and a pulmonary, cardiovascular and abdominal exam.

l. **Safety Laboratory Assessments** (refer to section 6.3 for details of laboratory safety assessments)

m. **Study Medication/Compliance:** Study medication administration will be supervised per local site practice to assure compliance to regimen.

n. **PK Sampling:** Pharmacokinetics will consist of two separate schedules:
   1. **All Subjects** - Pre-dose C_{trough} sampling at weeks 2, 8 and 16, must be taken within 1 hour before the next scheduled dose.
   2. **PK Sub-study Subjects** - in addition to the C_{trough} samples, there will be intensive PK sampling at week 16 at pre-dose, 0.5, 1, 2, 4, 8 12, 12.5, 13, 14, 16, 20 and 24 hours after dosing in a sub-group of evaluable 20 Subjects across selected sites. To be collected at the specified time points within the allowed applicable window periods: Pre-dose: 0-5 minutes before dose; 0.5- 1 hours post-dose: +/- 5 minutes; 2-8 hours post dose: +/- 5 minutes; 12 hours post-dose: +/- 5 minutes and prior to next dose for BID treatment arm, 12.5 – 13 hours +/- 5 minutes and 14- 24 hours +/- 10 minutes and prior to next dose. All PK sub-study participants must have received IMP at stable doses for at least two weeks prior to the sub-study sampling. If participant is in the midst of an IMP interruption at week 16, the PK draw should be post-poned until the regimen is resumed for 2 weeks.

o. **Sputum Sampling:**
   1. Screening (Day -9 to -1): A single spot sputum will be collected at the research site under the coaching and observation of the trial staff. The following analysis will be performed on this sample:
      - Smear microscopy for acid-fast bacilli (AFB);
      - Culture for presence or absence of *M.tb*.;
      - Gene Xpert, Hain Assay MTBDRplus or an alternative molecular or antigen-based test to confirm M.tb;
   2. All visits from Day 1 (baseline) up to and including Month 24: Two sputum samples, one early morning brought from home or in the hospital if hospitalized, and one spot at the research site under the coaching and observation of the trial staff (or if hospitalized, in the morning at least 1 hour after the early morning sample) will be collected. If early morning is not available, site should make every attempt to collect two spot samples at least 1 hour apart on site. sputum samples obtained at Month 4, End of Treatment (Week 26/39) or end of follow-up Month 24 are contaminated, the Subject should return for an unscheduled visit(s) to give additional samples or to document the Subject is not able to produce sputum. The following analyses will be performed on sputum samples at the study lab (lab that receives samples directly from the site):
      - Culture for presence or absence of *M.tb.*;
      - Speciation (on baseline and first positive at end of treatment or during follow-up or any positive at or after the week 16 visit)
      - If MGIT is performed, TTP in liquid medium.

   If participant has received at least 4 consecutive weeks of linezolid at a total daily dose of 1200 mg, and Investigator would like to consider discontinuing linezolid dosing and continuing bedaquiline and pretomanid dosing:
      - A smear microscopy for acid fast bacilli (AFB) should be requested by the site and performed at the study lab.

   3. First culture positive sample at or following end of treatment: Two sputum samples, one early morning brought from home and one spot at the research site under the coaching and observation of the trial staff (or if hospitalized, in the morning at least 1 hour after the ealry morning

sample) will be collected. If early morning is not available,site should make every attempt to collect two spot samples at least 1 hour apart on site.

- Culture for presence or absence of M.tb.;
- Extraction of bacterial (M.tb.) DNA for molecular genotyping
- Speciation (for initial relapse (first positive at end of treatment or during follow-up) or any positive at or after week 16))

Mycobacteriology Characterisation Tests, Performed on:

1. Day 1 (baseline) spot sputum samples (or Screening up to Week 4 if the baseline is contaminated or negative);
2. Confirmed Positive Cultures at or after end of treatment.

The *M.tb.* isolates will be processed at central lab(s) for:

- MIC against bedaquiline, pretomanid and linezolid;
- Drug Susceptibility Testing in liquid culture for rifampicin, isoniazid, streptomycin, ethambutol, pyrazinamide, and second-line TB drugs such as fluoroquinolones, and injectables;
- Extraction of bacterial (*M.tb.*) DNA for molecular genotyping;
- Speciation of the infecting organism by molecular/antigen tests

All Day 1 (baseline) *M.tb.* isolates and isolates from positive cultures to be stored at the study microbiology laboratory (or until requested to transfer to the central lab(s) for testing), until trial closure for the applicable study tests. The extracted *M.tb.* DNA and isolates will be stored for potential further work to validate new assay tools for a maximum of 5 years after trial closure.

p. **Visit Schedule:** Subjects who are culture positive or revert to being culture positive between month 4 and month 6 visits, will be withdrawn, or will receive a total of 9 months of treatment. *(Week 30, 34 and 39 visits should not be done for Subjects who complete study treatment in 6 months).* If the duration of treatment is extended due to dose interruptions (e.g., takes participant 8 months to complete 6 months of therapy), Unscheduled visits should be added every 4 weeks through last dose of IMP, then post final treatment visit, follow-up visits should be scheduled. Unscheduled Visits to include: Ophthalmology Examination, Vital Signs, Limited Physical Exam, Laboratory Safety Tests, Con Meds, Adverse Events, Study Medication/Compliance, Early Morning and Spot Sputum and the Peripheral Neuropathy Exam.

q. **Visit Windows:** the windows noted on the flowchart for timing of visit also apply to timing within a visit. For example, procedures that are difficult to schedule such as ophthalmology exams, should be scheduled within +/- 3 days of scheduled visit. Sites should make every effort to ensure all other procedures should be done on the same day when possible.

2. INTRODUCTION

2.1. Background

Although some progress has been made in recent years in controlling TB globally, TB has remained a persistent problem in many countries. TB is currently one of the top three fatal infectious diseases, it is the leading cause of death among HIV-infected individuals, and there is more TB in the world today than at any other time in history. The current first-line anti-tuberculosis agents have been in use for over 20 years and although the current regimens and drugs have been very successful in controlled clinical trials resulting in the permanent cure of more than 95% of trial Subjects, treatment takes 6 months to complete. This, plus side effects, result in poor compliance which is particularly likely to occur after the second month of treatment. As a result of poor treatment compliance, drug resistance is becoming more common and fears of an epidemic with virtually untreatable strains of TB – extensively drug resistant TB (XDR-TB) - are growing. Novel drugs for tuberculosis are needed for the growing numbers of patients with untreatable strains.

WHO is tracking the increase of drug resistant strains. They estimate that there are about 650,000 MDR-TB cases in the world at any one time. On average, an estimated 9.6% of MDR-TB cases have XDR-TB, defined as resistance to at least isoniazid and rifampicin, as well as to any fluoroquinolone, and to any of the three second-line injectables (amikacin, capreomycin, and kanamycin)[(20)]. XDR-TB has been reported by 92 countries. Among a subset of 795 XDR-TB patients in 26 countries, treatment success was 20% overall and 44% of patients died; South Africa reports the most XDR-TB cases[(22)]. In 2006 a report from rural South Africa noted that 52 patients died of 53 patients identified with XDR-TB and HIV co-infection; the mean survival was 16 days from the time of diagnosis[(21)]. A recent report from South Africa documented the very grave long term prognosis of patients with XDR-TB[(23)]. Between 2008 and 2012 107 patients with XDR-TB from 3 provinces in South Africa were followed for long term outcomes. At 60 months of follow up 12 patients (11%) had a favourable outcome, 78 (73%) had died, four (4%) had defaulted, and 11 (10%) had failed treatment. With such dire outcomes for patients with XDR-TB, novel drug combinations are needed to improve treatment outcomes. Recently, linezolid was identified as a potentially efficacious drug to use with patients with XDR-TB when added to a failing regimen[(24)].

Following the declaration of TB as a global emergency by the World Health Organization (WHO) in 1993, there has been a resurgence of efforts to develop improved TB therapies and several promising new agents are presently in or approaching clinical evaluation. On December 28, 2012 the U.S. Food and Drug Administration approved bedaquiline (Sirturo™) as part of combination therapy to treat adults with multi drug-resistant pulmonary tuberculosis (MDR-TB) when other alternatives are not available. On December 20, 2013 the CHMP recommended approval of bedaquiline by the EMA as part of an appropriate combination regimen for pulmonary multi-drug resistant tuberculosis (MDR-TB) when other treatments are not available. In November of 2013, the CHMP recommended that delamanid be approved by the EMA for treatment of pulmonary MDR-TB in combination with the WHO optimized background regimen. Although both of these regulatory actions are positive steps, more work needs to be done to develop new regimens for both drug-sensitive (DS-TB) and drug resistant TB (MDR-TB/XDR-TB). New combination regimens are desperately needed for two reasons; to shorten treatment to a duration more easily manageable by patients and public health services for DS-TB and to provide more efficacious, safer, better tolerated and affordable treatment for the growing number of patients suffering from MDR-TB and XDR-TB.

The Nix-TB study offers a new opportunity to treat patients with XDR-TB with three drugs for which there is no expected pre-existing resistance. Bedaquiline, pretomanid and linezolid are active against many drug resistant strains of *M.tb.* and substantial preclinical and clinical data are available to demonstrate their potential for individual and combined microbicidal and sterilizing activity in TB disease. To further development of novel

regimens, TB Alliance is currently conducting a series of Phase 2 studies to evaluate building blocks of this regimen. A recently completed 14 day Early Bactericidal Activity (EBA) study in South Africa (Study NC-003) demonstrated bactericidal activity of the bedaquiline-pretomanid-pyrazinamide regimen. This regimen is now being taken into an “SSCC” 8 week trial designed to investigate the combination of bedaquiline, pretomanid and pyrazinamide (B-Pa-Z) in DS-TB and bedaquiline, moxifloxacin, pretomanid and pyrazinamide in MDR-TB.

The current study (Nix-TB) is an open label trial designed to investigate the combination of bedaquiline, linezolid, and pretomanid in XDR-TB. Since patients with XDR-TB are failing nearly all drugs, there is no useful standard of care (SOC) to use for comparison. Because the long term outcome of patients in South Africa treated with available SOC is up to 75% mortality[23], this open label study offers the opportunity to identify a substantially better outcome without the need for a comparison group. This study will provide patients with an oral regimen that contains three novel drugs with the potential for a shorter treatment, better outcomes and with fewer side effects than the drugs currently being used.

The information presented below first details the key preclinical information and human efficacy and safety information for each of the drugs in the regimen and then present preclinical and clinical data to support the combination of these drugs in a regimen to treat patients with XDR-TB.

2.2. Agents to be Studied

2.2.1. Bedaquiline

Bedaquiline (formerly known as TMC-207; Sirturo™ package insert[3]) is a new agent being developed for TB treatment. As detailed in the Investigator’s Brochure[4,5] bedaquiline is a diarylquinoline that offers a novel mechanism of anti-tuberculosis action by specifically inhibiting mycobacterial adenosine triphosphate (ATP) synthase[6]. *In vitro*, bedaquiline potently inhibits both drug-sensitive and drug-resistant *M. Tb* isolates[7,8], and is also bactericidal against non-replicating *M. tb.*[9] In the murine model of TB, bedaquiline was as active as the triple combination of isoniazid (H), rifampicin (R), and pyrazinamide (Z). Addition of bedaquiline to HRZ results in accelerated clearance of *M. tb.*[1, 2]. There appears to be a synergistic interaction with pyrazinamide: 100% of mice were culture negative after 8 weeks of treatment with bedaquiline and pyrazinamide compared to 0% of mice treated with the standard regimen of rifampicin, isoniazid and pyrazinamide[10]. Collectively, these findings in the mouse model have led to the suggestion that regimens containing bedaquiline and pyrazinamide could be effective in the treatment of both drug sensitive and drug resistant TB and shorten treatment duration in patients. While the combination of bedaquiline and pretomanid in the murine model of TB appeared somewhat antagonistic relative to bedaquiline alone, it was as active as the triple combination of HRZ[10] and in a subsequent study it was more active in the mouse model than HRZ[11]. Thus a novel regimen with a bedaquiline plus pretomanid core could be effective in the treatment of MDR-TB/XDR-TB by providing two novel drugs for which there is no known pre-existing resistance.

To date, bedaquiline has been studied as monotherapy in two dose-ranging EBA trials (C202 and TMC207-CL001)[12,13], in two combination EBA trials (NC-001[14] and NC-003) and in 2 Phase 2b studies (C208 and C209). In the monotherapy studies, bedaquiline was dosed over a range of 100-400 mg/day. Subjects with TB had approximately a 1 log decrease in logCFU over 14 days at all doses studied. The first 14 day EBA combination study (NC-001) demonstrated that bedaquiline in combination with pyrazinamide (B-Z) and bedaquiline in combination with pretomanid (B-Pa) had positive EBA activity. The second 14 day EBA combination study (NC-003), currently undergoing analysis, included a number of bedaquiline-containing arms: bedaquiline, pyrazinamide and clofazimine (B-Z-C), bedaquiline, pretomanid and clofazimine (B-Pa-C); bedaquiline, pretomanid and pyrazinamide (B-Pa-Z) and bedaquiline, pretomanid, pyrazinamide and clofazimine (B-Pa-Z-C). Among these, B-Pa-Z had the best activity which was at least as good as the HRZE control.

The 2 completed Phase 2b studies form the pivotal studies reviewed by the FDA for accelerated approval of bedaquiline (Sirturo ™). Together, these clinical studies provide justification for proceeding to the current study and are described briefly below and in greater detail in the IB.

2.2.1.1. Bedaquiline Preclinical Studies

Full details of the preclinical studies are provided in the current bedaquiline Investigator's Brochure[4, 5] and Sirturo ™ label[3]. *In vitro* studies have demonstrated that the range of minimum inhibitory concentrations (MICs) for *M. tb.* H37Rv, the international reference strain, and 6 fully drug-susceptible clinical isolates, was 0.030 to 0.120 µg/ml. The activity of bedaquiline appears to be specific for mycobacteria, as the MICs for non-mycobacteria were at least 500-fold higher. The activity of the main metabolite of bedaquiline, M2, was determined against *M. tb.* H37Rv in both solid and liquid media and its MIC was found to be 0.1 µg/ml. This MIC shows that M2 is active against *M. tb.* but 3-6 times less active than the parent compound bedaquiline. Bedaquiline demonstrated similar *in vitro* efficacy against *M. tb.* clinical isolates resistant to the known anti-TB drugs (isoniazid, rifampicin, pyrazinamide, streptomycin, ethambutol, or fluoroquinolones). As expected, from the lack of cross-resistance with currently used anti-tuberculosis agents, bedaquiline retained activity against MDR-TB isolates.

The non-clinical safety evaluation of bedaquiline includes pharmacology, pharmacokinetics, toxicology and metabolism studies that were conducted in accordance with current ICH guidelines. Repeated dose toxicity studies were performed with dosing durations up to 3 months in mice and up to 6 months in rats and in dogs. Recovery was studied in rats and dogs. In repeated dose toxicity studies up to 3 months in mice, up to 6 months in the rat and up to 9 months in dogs, bedaquiline was associated with target organ changes in the mononuclear phagocytic system (indicative of phospholipidosis), stomach, liver, pancreas, and muscle. Toxicity was often associated with an increased presence of neutrophils in some tissues such as the female genital tract and this was preceded by a peripheral neutrophilia. For more detailed information please refer to the bedaquiline IB[4,5].

Respiratory parameters in rats were unaffected by treatment. There were no effects suggestive of neurological impairment or delayed neurotoxicity in rats. In single dose toxicity studies there were no mortalities following oral doses of up to 200 mg/kg in mice and rats. No mutagenicity or clastogenic effects were seen in a series of *in vitro* and *in vivo* genotoxicity tests. Bedaquiline was evaluated for possible developmental toxicity effects in the rat and the rabbit. No teratogenic effects were found. *In vitro*, bedaquiline slightly to moderately inhibited the delayed rectifier potassium current (IKr) in the human ether-à-go-go-related gene (hERG) model. Bedaquiline and M2 had no notable effects on IKr at 0.01 µM (0.006 and 0.005 µg/mL, respectively). However, at higher concentrations (0.03 to 3 µM), both compounds had a slight to strong concentration-dependent effect with a 50% inhibitory concentration (IC_{50}) of 0.37 µM (0.2 µg/mL) for bedaquiline and up to 0.45 µM (0.24 µg/mL) for M2. However, this effect did not manifest as a prolongation of repolarization in subsequent *in vivo* studies. There were no relevant effects on the isolated right atrium of guinea pigs *in vitro*, or in the isolated Langendorff-perfused rabbit heart. *In vivo*, positive chronotropic effects were seen in the anesthetized guinea pig after IV administration, but not in the conscious dog. In conscious, telemetered dogs, oral bedaquiline had no relevant effects on cardio-hemodynamic andelectrocardiogram (ECG) parameters.

Prior to the use of pretomanid in combination with bedaquiline in clinical study NC-001, a preclinical cardiovascular safety pharmacology study was conducted in unrestrained beagle dogs with both drugs to explore the potential for additive effects on QT prolongation induced by the combination. Results indicate that administration of 100 mg/kg bedaquiline daily, for 7 days, causes a small increase in QTc interval by Day 6 in some animals that is not influenced by the addition of 100 mg/kg pretomanid on Day 7 [15].The effect of pretomanid dosing alone on QT interval appeared to be due to discomfort related to the SC route of administration and not related to the plasma exposure (please see the pretomanid (formerly PA-824) Investigators Brochure for more detail).

2.2.1.2. Bedaquiline Clinical Studies

In the clinical studies conducted to date, a total of approximately 645 Subjects (including 265 healthy volunteers) have been exposed to bedaquiline in the Phase 1 and 2 clinical trials conducted as a part of the development program for the treatment of MDR-TB. An additional 45 Subjects received bedaquiline, either as monotherapy (B) or in combination with other agents (B-Pa or B-Z) in study NC-001, and 45 more in study NC-003 (B-Pa-Z, B-Pa-C, B-Pa-Z-C). Four short-term Phase 2a trials enrolled treatment-naïve Subjects (C202, TMC207-CL001, NC-001 and NC-003). One long-term, open-label, Phase 2 trial, in MDR-TB Subjects (bedaquiline-TiDP13-C209) and one long-term, Phase 2b trial, consisting of 2 different stages in Subjects infected with newly diagnosed sputum smear-positive pulmonary MDR-TB (bedaquiline-TiDP13-C208), have been completed. The principal findings of these trials are summarized below. Full details of the completed clinical studies are provided in the current bedaquiline IB [4,5] and Sirturo ™ label[3].

The Phase 1 trials have provided a basic understanding of bedaquiline's pharmacokinetic characteristics, drug-drug interaction potential, and short-term safety/tolerability profile in healthy Subjects. Bedaquiline was well absorbed with time to reach the maximum plasma concentration at approximately 5 hours post-dose. The maximum plasma concentration and AUC increased proportionally up to the highest doses studied (up to 700 mg in a single dose-ranging study, 800 mg single dose in study bedaquiline-TBC1003 and 400 mg q.d. multiple doses). Accumulation from Days 1 to 14 was approximately 2-fold expressed as increase in AUC, while trough concentrations increased up to 3.5-fold. The pharmacokinetics of bedaquiline were comparable between healthy Subjects and Subjects with pulmonary TB. The apparently close to steady-state concentrations in plasma after 14 days of daily treatment is ascribed to the important amount of the drug that is eliminated from the circulation during the ά and β phases of the plasma concentration-time curve. The average terminal elimination half-life of bedaquiline and metabolite M2 noted on extended follow-up after repeat dosing of Subjects with TB infection is about 5.5 months.

Administration of bedaquiline as the tablet formulation with food, increased the relative bioavailability (by 95%) compared to administration without food, and drug-drug interaction trials confirmed the role of cytochrome P450 3A4 (CYP3A4) in the metabolism of bedaquiline to M2. A recently completed study (DMID 10-0043) demonstrated that when given in combination, rifampicin, and to a lesser degree rifabutin, decreased exposure to bedaquiline presumptively due to induction of P450 enzymes. The clinical significance of these findings is unknown, however the current study (NC-005) will not permit the concomitant use of bedaquiline with any rifamycin.

The efficacy of bedaquiline was initially demonstrated in two monotherapy EBA studies C202 and TMC207-CL001. Study C202 was a 7 day study of three daily doses of bedaquiline (25, 100 and 400mg) in treatment-naïve Subjects with MDR-TB. In this study, the 400mg dose group demonstrated positive EBA and was numerically superior to the 25 and 100mg doses. In study TMC207-CL001, doses of 100, 200, 300 and 400mg daily (following a 2-day loading dose) were studied in Subjects with newly-diagnosed pulmonary TB for 14 days. There were no statistically significant differences between the treatment groups, although there was a numerical trend suggesting a positive dose-response relationship. Taken together, these studies establish that bedaquiline monotherapy has EBA in Subjects with TB and that higher doses appear to have greater efficacy.

A 14 day EBA regimen study (TB Alliance Study NC-001-(B-M-Pa-Z)) evaluated bedaquiline administered as monotherapy at 400 mg/d or in combination at that dose with either pretomanid administered at 200 mg/d or weight-adjusted pyrazinamide, to Subjects with pulmonary TB at 2 clinical sites in South Africa. The results indicate that over 14 days, the mean logCFU decreased by 1.3 from baseline in the 15 Subjects given bedaquiline 400 mg/d after a 2 day loading dose. In the cohort of 15 Subjects given bedaquiline 400 mg/d after a loading dose plus weight-adjusted daily doses of pyrazinamide (Z), the mean logCFU decreased by 2.0 logs from

baseline, indicating that Z potentiates the early bactericidal effect of bedaquiline. In the cohort with 15 Subjects given bedaquiline 400 mg/d after a loading dose plus 200 mg/d pretomanid, the mean logCFU decreased by 1.9. While it appeared that the addition of pretomanid potentiated the anti-tuberculosis activity of bedaquiline, the mean logCFU decrease of the combination was similar to that of two previous EBA studies using pretomanid monotherapy at 200 mg/day.

The second 14 day EBA combination study (NC-003) included a number of bedaquiline-containing arms: bedaquiline, pyrazinamide and clofazimine (B-Z-C), bedaquiline, pretomanid and clofazimine (B-Pa-C), bedaquiline, pretomanid, and pyrazinamide (B-Pa-Z) and bedaquiline, pretomanid, pyrazinamide and clofazimine (B-Pa-Z-C). Among these, B-Pa-Z had the best activity which was at least as good as the HRZE control (Daily Log CFU – 0.167 vs 0.151, respectively).

The long-term efficacy of bedaquiline in Subjects with MDR-TB has been studied in a placebo-controlled, randomized Phase 2b trial (C208) and an open-label, uncontrolled, Phase 2b trial (C209). In the Phase 2b placebo-controlled trial, C208, the addition of bedaquiline to a 5-drug MDR-TB regimen resulted in significantly faster time to culture conversion compared to placebo. During the 8-week treatment in Stage 1, 47.6% of Subjects in the bedaquiline group became MGIT culture negative compared to 8.7% of Subjects in the placebo group. At Week 24 in Stage 1, after 8 weeks of investigational treatment and 24 weeks of background treatment, 81.0% of Subjects in the bedaquiline group and 65.2% of Subjects in the placebo group showed treatment success, (i.e., completed week 24 and were liquid culture negative at this time point).

For C208 Stage 2, in the interim analysis as well as in the primary efficacy analysis, a statistically significant difference in time to culture conversion between the treatment groups ($p < 0.0001$) in favour of bedaquiline was shown. In both analyses, an identical number of Subjects with culture conversion at week 24 (i.e., 24-week responders [missing = failure]) was observed: 78.8% in the bedaquiline group and 57.6% in the placebo group, which was statistically significantly different ($p = 0.008$) based on a logistic regression model with only treatment as covariate. Microbiological response at Week 24 was durable in C208 Stage 2: the percentage of responders (missing = failure) at week 72 was 71.2% in the bedaquiline group and 56.1% in the placebo group.

In the Phase 2b uncontrolled trial, C209, treatment with bedaquiline in combination with an individualized MDR-TB treatment regimen was effective against pulmonary MDR-TB both in newly diagnosed and in non-newly diagnosed Subjects. Culture conversion rates after 24 weeks of treatment with bedaquiline as part of an individualized anti-tuberculosis regimen were higher in Subjects with lower extent of resistance of the *M. Tb* strain and in Subjects with no lung cavitation compared to Subjects with cavitations (in one or both lungs).

2.2.1.3. Bedaquiline Clinical Safety

In the clinical studies conducted to date, a total of approximately 645 Subjects (including 265 healthy volunteers) have been exposed to bedaquiline in the Phase 1 and 2 clinical trials conducted as a part of the development program for the treatment of MDR-TB. An additional 60 Subjects received bedaquiline in a monotherapy EBA study of 14 days (study TMC207-CL001) 45 Subjects received bedaquiline, either as monotherapy (B) or in combination with other agents (B-Pa or B-Z) in study NC-001 and 45 more in study NC-003 (B-Pa-Z, B-Pa-C, B-Pa-Z-C). In these studies, bedaquiline has been shown to be an effective treatment for Subjects with both DS and MDR-TB. Specifically, the regimen B-Pa-Z was demonstrated to have efficacy at least as good as the HRZE control in study NC-003. Furthermore, bedaquiline is a novel agent with no pre-existing resistance and, based on mouse model data, may result in shortened treatment durations when included in novel regimens to treat both DS- and MDR-TB. Based on the combined experience in these clinical studies, the following known and potential risks have been identified.

Adverse Drug Reactions for bedaquiline

During the Investigational Treatment phase in the controlled trials, the most frequently reported Adverse Drug Reactions in the any bedaquiline group (> 10.0% of Subjects) were nausea, arthralgia, headache, vomiting, and dizziness. Details of Adverse Events, none of which were Serious, that were considered Adverse Drug Reactions are in the table below:

Table 1: Bedaquiline Adverse Drug Reactions

	Investigational Treatment Phase			
	Controlled Trials			
	TMC207		Placebo	
SOC<br>ADR (Grouped term), n (%)	24 Weeks<br>N = 79	Any<br>N = 102	24 Weeks<br>N = 81	Any<br>N = 105
At least grade 3 ADR	*5 (6.3)*	*5 (4.9)*	*0*	*0*
**Nervous system disorders**	**1 (1.3)**	**1 (1.0)**	**0**	**0**
Headache	1 (1.3)	1 (1.0)	0	0
**Cardiac disorders**	**0**	**0**	**0**	**0**
ECG QT Prolonged	0	0	0	0
**Gastrointestinal disorders**	**0**	**0**	**0**	**0**
Diarrhea	0	0	0	0
Vomiting	0	0	0	0
**Hepatobiliary disorders**	**2 (2.5)**	**2 (2.0)**	**0**	**0**
Transaminases increased [a]	2 (2.5)	2 (2.0)	0	0
**Musculoskeletal and connective tissue disorders**	**2 (2.5)**	**2 (2.0)**	**0**	**0**
Arthralgia	2 (2.5)	2 (2.0)	0	0

Mortality

Overall, there was an imbalance in the number of deaths in the pooled Stage 1 and Stage 2 C208 trial. In the pooled analysis (Stage 1 and Stage 2), 12 Subjects in the Any bedaquiline group and 5 Subjects in the Any Placebo group experienced a SAE leading to death; causes of death were varied with only death due to TB reported more than once, and none of these Subjects had a treatment-emergent QTcF ≥ 500 ms. The imbalance in deaths is primarily driven by the C208 Stage 2 results in which the imbalance was 10 bedaquiline Subjects (12.7%) compared to 3 placebo Subjects (3.7%). Based on the pooled results (Stage 1 and 2) while being followed in the placebo-controlled trial, 7 Subjects in the Any bedaquiline group and 1 Subject in the Any Placebo group died. Of these deaths, 1 occurred during the Investigational Treatment phase with bedaquiline/placebo, the remaining deaths occurred afterwards. In the Any bedaquiline group, causes of death were myocardial infarction, TB (2 cases), alcohol poisoning, hepatitis and hepatic cirrhosis (1 case), septic shock and peritonitis (1 case), and cerebrovascular accident. In the Any Placebo group, cause of death was hemoptysis. The Investigator considered the SAEs leading to death not related to bedaquiline intake in the Any bedaquiline group and doubtfully related to investigational medication in the Any Placebo group. The analysis of long-term follow-up for survival outcomes in Subjects who prematurely discontinued in trial C208 (Stage 1 and 2), based on data collection every 24 weeks (6 months) after withdrawal (up to LSLV in the rollover arm [16 Oct 2012] in Stage 2), included 9 deaths. One Subject in the bedaquiline group (pulmonary TB) and 2 Subjects in the Placebo group (TB-related illness and pulmonary TB) in Stage 1 died, and 4 Subjects in the bedaquiline group (3 Subjects with TB-related illness, 1 Subject motor vehicle accident) and 2 Subjects in the Placebo group (both TB-related illness) died after they discontinued Stage 2 of the trial. None of these Subjects had a treatment-emergent QTcF ≥ 500 ms. The imbalance in deaths is unexplained. In addition, no discernible pattern between death and sputum culture conversion, relapse,

sensitivity to other drugs used to treat TB, human immunodeficiency virus (HIV) status, or severity of disease was observed.

In the uncontrolled Phase 2b trial, C209, the most frequently reported AEs during the investigational phase were hyperuricemia, arthralgia, nausea, vomiting, headache, diarrhea, blood uric acid increased, hypokalemia, pruritus, injection site pain, insomnia, and tinnitus. From start of the trial up to the final analysis, 12 Subjects died during the C209 trial due to SAEs that included TB (5 cases), congestive cardiac failure, renal impairment, lung infection, cardiac arrest (underlying cause pneumonia), hemoptysis, hypertension, and pyopneumothorax/respiratory failure. All of these SAEs leading to death were considered not related to bedaquiline by the Investigator, except for renal impairment that was judged doubtfully related to bedaquiline.

The analysis of long-term follow-up for survival outcomes for Subjects who prematurely discontinued in trial C209, based on data collection every 24 weeks (6 months) after withdrawal, included 4 deaths (all described as TB-related). In total, since the start of the C209 trial, 16 Subjects (6.9%) have died (12 Subjects during the trial and 4 Subjects during the survival follow-up phase after premature discontinuation). None of the fatal SAEs were considered related to bedaquiline by the Investigator and none of these Subjects has a treatment-emergent adverse event.

Cardiovascular safety

During clinical trials with bedaquiline a prolongation of QTc interval was observed. The US Product Label for bedaquiline(3) notes that treatment initiation is not recommended in patients with:

- Heart failure;
- QTcF interval > 450 ms (confirmed by repeat electrocardiogram);
- A personal or family history of congenital QT prolongation;
- Concomitant administration of fluoroquinolone antibiotics that have a greater potential for significant QT prolongation (i.e., gatifloxacin, moxifloxacin and sparfloxacin).
- Hypokalemia

The US Product Label recommends that bedaquiline treatment must be discontinued if the patient develops clinically significant ventricular arrhythmia. An additive or synergistic effect on QT prolongation of bedaquiline when co-administered with other medicinal products that prolong the QT interval cannot be excluded. Caution is recommended when using bedaquiline concomitantly with medicinal products with a known risk of QT prolongation. In the event that co-administration of such medicinal products with bedaquiline is necessary, clinical monitoring including frequent ECG assessment is recommended.

Hepatic safety

The US product label notes increases in transaminases were seen in clinical trials during administration of bedaquiline with the background regimen. Subjects should be monitored during treatment. Other hepatotoxic medicinal products and alcohol should be avoided while taking bedaquiline, especially in those Subjects with diminished hepatic reserve(3).

Additional safety information from a recently completed trial

In a recently completed phase I study not yet included in the bedaquiline Investigators Brochure that evaluated drug-drug interactions between bedaquiline and either rifampin or rifabutin, 7 Subjects experienced SAEs. Of those Subjects, 5 experienced SAEs of lymphocytopenia (all received rifabutin), which did not appear to be related to the AUC or C_{max} of TMC207 or rifabutin. One subject experienced an SAE of elevated Creatine Kinase (rifampicin arm) and one experienced a grade 4 Total Bilirubin SAE (rifampicin arm). Lymphopenia was previously noted to be an infrequent toxicity associated with rifabutin, but not previously seen with bedaquiline. The

bedaquiline/rifabutin regimen demonstrated significant reversible lymphopenia, which did not appear to be related to the AUC or C_{max} of TMC-207 or rifabutin.

2.2.2. Pretomanid

As detailed in the Investigator's Brochure[15], pretomanid is a new chemical entity and a member of a class of compounds known as nitroimidazo-oxazines, which possess significant anti-tuberculosis activity and a unique mechanism of action[16]. Pretomanid demonstrated *in vitro* activity against both DS- and MDR-TB[17], and *in vivo* activity in a mouse model of tuberculosis[16,17].

Pretomanid has been studied in four 14-day EBA trials to date, including two monotherapy dose-ranging studies and two combination EBA studies. Pretomanid monotherapy has demonstrated substantial mycobactericidal activity. The efficacy data from study PA-824-CL-007 indicated that all doses of pretomanid (200, 600, 1000 and 1200 mg) produced an equivalent decrease in sputum CFU counts over the 14-day treatment period. In study PA-824-CL-010, an EBA study with a similar design to study PA-824-CL-007 except for the use of lower doses of pretomanid (50, 100, 150 and 200 mg/day), results indicated that pretomanid treatment resulted in a measurable dose-dependent mycobactericidal activity over the dose range studied, and supported a clinical dose of 200mg per day. In study NC-001-(B-M-Pa-Z) an EBA study with multiple treatment combinations, the three drug combination of pretomanid (200 mg per day), moxifloxacin and pyrazinamide (Pa-M-Z) demonstrated potential as a treatment shortening regimen and was progressed into an 8 week "SSCC" study (NC-002) in which the combination was shown to be statistically better that the HRZE control on some measures of activity. In the second 14-day combination EBA study (NC-003), the bedaquiline, pretomanid and pyrazinamide (B-Pa-Z) regimen showed promising activity and has been selected to move forward in development and is the focus of an 8-week study in patients with DS and MDR-TB (NC-005) that will be initiated in 2014.

2.2.2.1. Pretomanid Preclinical Studies

Microbiology

In vitro studies have demonstrated that the minimum inhibitory concentration (MIC) of pretomanid against a variety of drug-sensitive *M. tb.* isolates is similar to the MIC of isoniazid (MIC of pretomanid, ≤0.015 to 0.25 µg/mL; MIC of isoniazid, 0.03 to 0.06 µg/mL). Pretomanid was efficacious *in vitro* against drug-resistant clinical isolates of *M. tb.,* with MIC values ranging from 0.03 to 0.53 µg/mL. The minimum effective dose (MED) of pretomanid was 12.5 mg/kg/day in a mouse model of TB. The MED is defined as the lowest dose able to prevent the development of macroscopic lung lesions and splenomegaly. The minimum bactericidal dose (MBD) was 100 mg/kg/day in the same mouse model. The MBD is defined as the lowest dose able to reduce the lung TB colony forming unit (CFU) counts by 99%. The magnitude of CFU reduction by pretomanid at this dose is similar to that seen with the highest dose of isoniazid tested in the same study (25 mg/kg/day).

Nonclinical Safety Studies

The non-clinical safety evaluation of pretomanid includes pharmacology, pharmacokinetics, toxicology and metabolism studies that were conducted in accordance with current ICH guidelines.

Pretomanid was negative in all genotoxicology studies performed. One of its metabolites (M50) that is found in rat, monkey, and human plasma was positive in a screening Ames assay. M50 is not a major metabolite in humans and the exposure relative to parent drug is higher in the rat (24%) and monkey (18%) than in humans (6%).

Pretomanid-induced effects in respiratory, CNS, and cardiovascular safety pharmacology studies were generally mild and reversible; effects were most prominent at 450 mg/kg/day. Pretomanid is a weak inhibitor (IC_{50}=20µM) of the hERG channel. In a telemetry monkey study, in the dose range 50-450 mg/kg, there was no or minor

prolongation of the QT interval, depending on the method of correction. The weight of evidence suggests that there should be no effect on QT in the dose range being explored in the clinical studies.

Repeat-dose toxicology studies with pretomanid have been conducted in male and female rats (14 days to 6 months), and in male and female monkeys (7 days to 3 months). In all studies, dose-dependent reduced food consumption and reduced weight gain or weight loss were noted. In addition, testicular atrophy was observed in rats while cataracts were observed by indirect ophthalmoscopy in both rats and monkeys. In general, toxicity in both rat and monkey was significantly greater when exposures exceeded approximately 300 µg•hr/mL in the 14-day studies and approximately 200 µg•hr/mL in the 3-month studies.

Reproductive toxicology studies show that pretomanid is not teratogenic in rats or rabbits. In the rat fertility study, dose-dependent reduced fertility rates, due to decreased sperm numbers and decreased motility, were observed at doses of 30 mg/kg and greater. This effect was partially reversible. As in the 3-month rat toxicology study, irreversible testicular lesions were not observed at 30 mg/kg, only at 100 and 300 mg/kg.

To more fully characterize the cataract and male reproductive system findings, a 3-month monkey study in sexually mature males (0, 50, 150, 300 mg/kg/day), and a 6-month rat study (0, 30, 100, 300 mg/kg) in males and females were conducted. Ocular assessments were conducted in a much more careful and systematic manner than in the initial 3-month toxicology studies described above. In each of the later studies, all ophthalmologic examinations were conducted by a single ophthalmologist, using both indirect ophthalmoscopy and slit-lamp biomicroscopy. Animals were screened before dosing to ensure no animal had cataracts at baseline, and then monthly during dosing and recovery. In this monkey study, although similar drug exposures were attained as in the original 3 month monkey study, no cataracts or testes effects (semenology, organ weights, histopathology, or hormones [testosterone, follicle-stimulating hormone, Inhibin B]) were observed at any point during dosing or during a 20-week recovery period. Pretomanid does not appear to cause cataracts or testicular toxicity in monkeys. In the 6-month rat study, pretomanid caused irreversible cataracts at 100 mg/kg from Day 118 of the study in males and females. In contrast to the original 3-month rat report, rats in this more carefully conducted study developed cataracts while on drug but not during recovery. The NOAEL was 30 mg/kg for cataracts and 10 mg/kg for testicular toxicity. Rats that developed cataract and testicular toxicity also experienced marked decreases in body weight gain and food consumption. The AUC safety multiples (relative to the exposures obtained at the anticipated clinical dose of 200 mg/day) for cataract are ~1.5x in the rat; in the monkey at the highest dose tolerated, where there were no cataracts in the second well conducted study, the multiple is at least 3.7x.

To summarize, cataracts have been detected in multiple animals from two similar rat studies at mid-to-high doses. In contrast, the finding of cataracts in one monkey study could not be confirmed in a follow-up study. Thus, both cataracts and the testicular effects appear to be species-limited.

An overall summary of the findings from animal safety and toxicology studies is contained in Table 2.

Table 2: Pretomanid Key Animal Safety and Toxicology Findings

• Nervous system-related effects.<br>Rats given single oral pretomanid doses had decreased body tone, touch responses and decreased grooming behavior at ≥150 mg/kg, which resolved within 24 hours. Rats given repeated daily doses of pretomanid had convulsions, ataxia, hypoactivity, recumbency, hyperactivity and sensitivity to touch, and squinting at ≥100 mg/kg/day, and early deaths occurred at doses ≥500 mg/kg/day. Monkeys given repeated daily doses of pretomanid had hypoactivity, ataxia, tremors, and convulsions at ≥450/300 mg/kg/day. These effects were reversible when dosing stopped and were absent at ≤30 mg/kg/day in rats and ≤150 mg/kg/day in monkeys.
• Testicular toxicity<br>Testicular degeneration/atrophy, occurred in rats with repeated doses of pretomanid at ≥30 mg/kg/day but did not occur in monkeys at any dose level. Testicular effects showed evidence of being partially reversible, albeit very slowly, in rats dosed for 7 days, but not in rats dosed for 14 days. As would be expected, there was a dose-related decrease in fertility in male rats at ≥30 mg/kg/day that was associated with decreased sperm numbers and motility. This effect on fertility in male rats was partially reversible.
• Cataracts<br>Cataracts developed with prolonged daily dosing in rats at pretomanid doses ≥100 mg/kg/day. In one 13-week study in monkeys, cataracts did develop at 450/300 mg/kg/day, but only by the end of a 13-week recovery period. In a second 13-week study in monkeys that included extensive ophthalmic examinations, cataracts did not develop at the high-dose level of 300 mg/kg/day.
• hERG inhibition and QT prolongation<br>Pretomanid inhibited hERG current with IC_{50} values of approximately 6.2 μg/mL. Following a single pretomanid dose of 450 mg/kg in monkeys, QTc interval prolongation ranged from 21 to 36 msec using Fridericia's formula (QTcF) to correct for heart rate. Co-administration of pretomanid with moxifloxacin in the monkey or with bedaquiline in the dog did not result in any greater effect on the QT interval than with either agent alone. After repeated daily doses, the QTc interval in the monkey was prolonged at pretomanid doses of ≥150 mg/kg/day.

2.2.2.2. Pretomanid Clinical Studies

Phase 1

The safety, tolerability and pharmacokinetics of pretomanid have been studied in 10 Phase 1 studies, which are summarized in Table 3. In these trials, pretomanid has been administered in doses ranging from 50 to 1500 mg, as 50 or 200 mg tablets or as an oral suspension. PK parameters have largely been consistent in each study and can be summarized as follows:

- Pretomanid is moderately rapidly absorbed, with median T_{max} values across Subjects and dose levels ranging from 4 to 7 hours.
- The mean half-life for elimination ($t_{½}$) across Subjects and dose levels was approximately 16 - 25 hours.
- Exposure increased approximately linearly but less than dose-proportionally, with increasing doses up to approximately 600 – 1000 mg, while higher doses achieved minimal additional increases in either C_{max} or AUC.

Two studies using radiolabeled pretomanid in an oral-suspension formulation have been conducted to investigate the metabolism and excretion patterns of pretomanid in humans: Study PA-824-CL-004, which used [benzyl-^{14}C] pretomanid and Study PA-824-CL-008, which used [imidazooxazine-^{14}C] pretomanid. The mass balance results from the two studies were very similar. In each study, the majority (53-65%) of radioactivity was excreted in the urine; an additional 26-38% was collected in the feces such that approximately 91% of the administered dose was ultimately recovered in the excreta.

Radioprofiling and metabolite identification work have been completed on samples from the two human studies as well as from analogous work in rat and monkey using both radiolabeled pretomanid preparations. The metabolism of pretomanid proceeds via a combination of reductive metabolism (~20 – 25% of the dose) and oxidative metabolism (remainder of the characterized metabolites). The metabolic profile of pretomanid *in vivo* was qualitatively similar in the three species, with quantitative differences being minor. No human unique metabolites were detected and there is no one single metabolic path that can be considered major. Furthermore, there are no major metabolites in human plasma.

Study PA-824-CL-006, a drug-drug interaction study with midazolam to assess the extent of CYP3A inhibition by pretomanid, results indicate that dosing of pretomanid 400 mg once daily for 14 days did not have a major effect on the exposure of midazolam or its major metabolite 1-hydroxy midazolam. For midazolam, the geometric mean ratio of Day 17 (midazolam+pretomanid) vs. Day 1 (midazolam alone) for C_{max} was 0.84 and AUC was 0.85. For the 1-hydroxy midazolam metabolite, the corresponding geometric mean ratio for Cmax was 1.05 and AUC was 1.11. The data suggests that pretomanid does not cause clinically significant drugs interactions with drugs metabolized by CYP3A.

Two additional studies have recently been completed and are currently undergoing analysis: Study DMID 10-0058 (a Thorough QT study comparing pretomanid and pretomanid plus moxifloxacin to moxifloxacin alone) and Study ACTG 5603 (a drug-drug interaction study evaluating the effects of concomitantly administered Efavirenz, Ritonavir-Boosted Lopinavir or rifampicin on the PK parameters of pretomanid). The first study found that single doses of pretomanid of 400mg and 1000mg did not have a clinically significant effect on the QTc interval, and when pretomanid at 400mg is co-administered with moxifloxacin (400mg) it did not increase the QTc prolongation substantially over what is seen with moxifloxacin alone. The second study found that when administered with Efavirenz, Ritonavir-Boosted Lopinavir, or Rifampicin, pretomanid (200mg) median pretomanid concentrations (AUC_{0-24h}) were reduced 35% by EFV, 17% by LPV/r, and 66% by rifampin. The clinical significance of these findings requires further investigation.

Study PA-824-CL-009, a food effects study pretomanid (200 mg and 50 mg); results indicate that the food effect observed is dependent on the pretomanid dose administered. When a single dose of pretomanid was administered with a high fat, high calorie meal, C_{max} and AUC of the 50 mg dose increased 1.40-fold and 1.45-fold respectively, whereas for the 200 mg dose, C_{max} increased 1.76-fold and AUC increased 1.88-fold.

Table 3: Pretomanid Phase 1 Clinical Studies

Study	Design	Pretomanid Dose	Enrolled	Key Findings
**CL-001**	Double-blind, placebo-controlled, single-dose, dose-escalating, PK and safety study	0, 50, 250, 500, 750, 1000, 1250, 1500	53	• Well tolerated; no dose-limiting AEs or abnormal laboratory results; no effects on ECG, vital signs, or PE.
**CL-002**	Double-blind, placebo-controlled 7-day multidose, escalating, PK and safety study	0, 200, 600, 1000	24	• Well tolerated; no effects on ECG, vital signs, or PE.<br>• After 5 days' dosing at 1000 mg/d, progressive moderate creatinine elevation: reversed during 7-day washout period.<br>• No consistent effect on BUN.<br>• A planned 1400-mg cohort not enrolled.
**CL-003**	Open-label, single-dose, food effects	1000	16	• Well tolerated; no dose-limiting AEs or abnormal laboratory results; no effects on ECG, vital signs, or PE.<br>• Treatment-emergent AEs affecting more than one Subject occurred more frequently after dosing in the fed condition than the fasted condition, and more frequently among women than men.<br>• Bioavailability is 3.5-to-4.5-fold higher when pretomanid is administered within 30 minutes of a high-fat, high-calorie meal than when it is administered after an overnight fast.
**CL-004**	Open-label, single-dose, ADME	~860, oral suspension [benzyl-^{14}C]pretomanid	6	• Well tolerated; no dose-limiting AEs or abnormal laboratory results; no effects on ECG, vital signs, or PE.<br>• No significant radioactivity captured as [benzyl-$^{14}C]CO_2$.<br>• ~91% of dose recovered (~65% in urine; ~26% in feces)<br>• Plasma: parent drug and one major metabolite.<br>• Urine: little or no parent drug; multiple major metabolites.<br>• Feces: minimal unchanged parent drug; numerous low-abundance metabolites.

Study	Design	Pretomanid Dose	Enrolled	Key Findings
**CL-005**	Double-blind, 8-day multidose, renal effects	0, 800, 1000	47	• Well tolerated; no dose-limiting AEs or abnormal laboratory results; no effects on ECG, vital signs, or PE.<br>• As anticipated, serum/plasma creatinine levels increased significantly (up to ~ 40%) during treatment; reversed during 7-day washout period.<br>• No effect during treatment on GFR, ERPF, FF, BUN or UA.
**CL-006**	Open-label, multidose, DDI	400	14	• Well tolerated; no dose-limiting AEs.<br>• For midazolam, the geometric mean ratio of Day 17 (midazolam+Pa-824) vs. Day 1 (midazolam alone) for C_{max} was 0.84 and $AUC_{(0\text{-infinity})}$ was 0.85.<br>• For the 1-hydroxy midazolam metabolite, the corresponding geometric mean ratio for Cmax was 1.05 and $AUC_{(0\text{-infinity})}$ was 1.11.
**CL-008**	Open-label, single-dose, ADME	~1100, oral suspension [imidazooxazine-^{14}C] pretomanid	6	• Well tolerated; no dose-limiting AEs or abnormal laboratory results; no effects on ECG, vital signs, or PE.<br>• No significant radioactivity captured as [imidazooxazine-^{14}C] CO_2.<br>• ~91% of dose recovered (~53% in urine; ~38% in feces) Plasma: parent drug. Urine: little or no parent drug; multiple major metabolites.<br>• Feces: unchanged parent drug and numerous low-abundance metabolites.
**CL-009**	Open-label, single-dose, food effects	50 and 200	32	• Well tolerated; no dose-limiting AEs.<br>• In the presence of high fat, high calorie diet, C_{max} and AUC of the 50-mg dose increased 1.40-fold and 1.45-fold respectively, whereas for the 200-mg dose, C_{max} increased 1.76-fold and AUC increased 1.88-fold.
**A5306**	Antiretroviral DDI	200	48	• Based on preliminary data – the study is currently undergoing analysis.<br>• Co-administration with Efavirenz resulted in a 35% reduction in pretomanid AUC.<br>• Co-administration with Ritonavir-Boosted Lopinavir resulted in a 17% reduction in pretomanid AUC.<br>• Co-administration with rifampicin resulted in a 66% reduction in pretomanid AUC.

Study	Design	Pretomanid Dose	Enrolled	Key Findings
**10-0058**	Thorough QT Study	400 and 1000	75	• Pretomanid, alone and in combination with moxifloxacin, was well tolerated.<br>• Pretomanid doses of 400 mg and 1000 mg did not cause QT interval prolongation to a level of clinical concern as the upper limit of the 90% CI associated with any LS mean ΔΔQTcI value did not exceed 4.4 ms for the 400-mg dose or 6.1 ms for the 1000-mg dose, and both were well below 10 ms.<br>• The effect of pretomanid 400 mg plus moxifloxacin 400 mg on QTcI was similar to the effect of moxifloxacin administered alone.<br>• No Subject receiving pretomanid or moxifloxacin alone had an observed QTcI value that exceeded 450 ms or experienced a change-from-baseline in QTcI that exceeded 30 ms.<br>• The PK of pretomanid was not affected by the co-administration of moxifloxacin.

- **Phase 2**

Study PA-824-CL-007, a 14 day monotherapy EBA study, indicated that all doses of pretomanid (200, 600, 1000 and 1200mg a day) produced a measurable and equivalent decrease in sputum CFU counts over the 14-day treatment period. Study PA-824-CL-010 was an EBA study with a similar design to study PA-824-CL-007 except for the use of lower doses of pretomanid (50, 100, 150 or 200 mg/day). Results indicate that pretomanid treatment resulted in a measurable dose-dependent mycobactericidal activity, with the 50 mg dose demonstrating less activity than the 100, 150 and 200 mg doses, which were all equivalent.

Study NC-001-(B-M-Pa-Z) was a 14 day EBA study that assessed the two-week EBA of the following drug combinations: pretomanid plus pyrazinamide, pretomanid plus pyrazinamide plus moxifloxacin, along with two other non-pretomanid containing combinations. Results indicate that the three drug combination of pretomanid (200 mg per day), moxifloxacin and pyrazinamide has potential as a treatment shortening regimen. In the study this three drug combination has an EBA 0-14, which is believed indicative of sterilizing activity, numerically better than the current 4-drug intensive phase treatment of HRZE.

The recently completed Phase 2b study, NC-002, was a multi-center open-label partially randomized clinical trial with four treatment groups. Subjects with drug-sensitive TB were randomized to receive moxifloxacin 400 mg plus PA-824 100 mg plus pyrazinamide 1500 mg (M-PA100-Z) or moxifloxacin 400 mg plus PA-824 200 mg plus pyrazinamide 1500 mg (M-PA200-Z) or standard HRZE therapy. HRZE was included as a control arm for the drug-sensitive treatments and for the laboratory methodology. Subjects with MDR-TB received moxifloxacin 400 mg plus PA-824 200 mg plus pyrazinamide 1500 mg (M-PA200-Z MDR). The study population included a total of up to 230 male and female newly diagnosed Subjects with drug-sensitive or multi drug-resistant, smear positive pulmonary tuberculosis aged 18 to 65 years (inclusive). The primary efficacy endpoint was the rate of change in the logarithm of colony forming unit (CFU) (or log[CFU]) count) over 8 weeks of treatment analysed by a Joint Bayesian Non-linear Mixed Effect (NLME) regression.
Preliminary analyses of NC-002 indicate that a total of 207 Subjects were enrolled, with 60 randomized to M-PA100-Z, 62 randomized to M-PA200-Z, and 59 to HRZE. An additional 26 Subjects were treated in the M PA200-Z MDR-TB arm. Of note, more Subjects in the MDR-TB arm did not complete the full 8 weeks of treatment, primarily because many were withdrawn as late-exclusions (*M. Tb* resistant to pyrazinamide determined in culture after enrollment in the study). Twenty-one MDR-TB Subjects were in the study with active treatment through day

14 and 10 were in the study through the full 8 weeks of treatment (9 with evaluable results for the primary microbiological endpoint). In contrast, the following number of Subjects in the study with active treatment through 8 weeks in the 3 randomized arms with evaluable results for the primary microbiological endpoint: 55 in the M-PA100-Z arm, 54 in the M-PA200-Z arm and 54 in the HRZE arm. For the primary endpoint, Subjects in the M-PA200-Z arm had a statistically significantly greater decline in the log CFU count over the 8 weeks, than the Subjects in the HRZE arm. Finally, pretomanid has recently been studied in combination with bedaquiline and other agents in a 2 week EBA study (NC-003).

Table 4: Pretomanid Phase 2 Studies

Study	Design	Pretomanid Doses	Enrolled	Key Findings
CL-007	Partially double-blinded (blinded as to pretomanid dose), 14-day multidose, extended early bactericidal activity.	200, 600, 1000, 1200	69	• Overall well tolerated with relatively few AEs and no dose-limiting AEs or laboratory findings. No clinically significant effects on ECG, vitals, or PE noted.<br>• Two SAEs occurred during study, both were considered possibly related to TB disease (hemoptysis).<br>• Pretomanid treatment produced a measurable decrease in log CFU, with the magnitude of effect equivalent for all doses.
CL-010	Partially double-blinded (blinded as to pretomanid dose), 14-day multidose, extended early bactericidal activity.	50, 100, 150, 200	69	• Well tolerated.<br>• Pretomanid treatment produced a measurable decrease in log CFU with some evidence of dose dependence.
NC-001	Partially double-blinded (blinded as to combination within Pa or B containing arms), 14-day multidose, extended early bactericidal activity.	200	85	• Well tolerated.<br>• Pretomanid plus moxifloxacin plus pyrazinamide combination treatment produced a decrease in log CFU at least comparable to that of the Rifafour e-275® control group.
NC-002	Multi-center open-label partially randomized clinical trial in four treatment groups. Subjects with drug-sensitive TB randomized to receive moxifloxacin 400 mg plus pretomanid 100 mg plus pyrazinamide 1500 mg or moxifloxacin 400 mg plus pretomanid 200 mg plus pyrazinamide 1500 mg or Rifafour e-275®.	100, 200	207	Based on preliminary results:<br>• For the primary endpoint Subjects in the M-PA200-Z arm had a statistically significantly greater decline in the log CFU count over the 8 weeks than the Subjects in the HRZE arm.<br>• Well tolerated overall.<br>• Eleven serious adverse events (SAEs) were reported in 9 Subjects, with one Subject in each of the M-PA100-Z and the Rifafour® groups, and 7 Subjects in the M-PA200-Z group.

Study	Design	Pretomanid Doses	Enrolled	Key Findings
NC-003	Multi-center, open-label, randomized clinical trial with seven parallel treatment arms. Fifteen Subjects were enrolled in each of the following treatment arms: TMC207 plus pretomanid plus pyrazinamide plus clofazimine, TMC207 plus pretomanid plus pyrazinamide, TMC207 plus pretomanid plus clofazimine, TMC207 plus pyrazinamide plus clofazimine, pyrazinamide alone, clofazimine alone, and Rifafour e-275®.	200	105	• Among the regimens studied, the combination B-PA-Z demonstrated the highest EBA, with results at least comparable to the HRZE control.<br>• The treatments administered in this trial were well tolerated by the trial population.<br>• No deaths were reported in this trial. Serious AEs were reported for 1 Subject (1.0%) in the clofazimine alone arm: gastroenteritis, anemia, and deep vein thrombosis (none of which were considered to be related to the treatment).

2.2.2.3. Pretomanid Clinical Safety

Across the 15 clinical studies with pretomanid completed to date, a total of 649 Subjects have been exposed to pretomanid, including 289 healthy Subjects across the 10 Phase 1 studies and 360 Subjects with newly diagnosed smear positive pulmonary TB across 5 Phase 2 studies. Among the 289 healthy Subjects, 174 received exposure to a single dose of pretomanid ranging from 50 to 1500 mg and 115 received exposures to repeated daily doses of pretomanid (50 to 1000 mg) for up to 14 days. The 360 Subjects with newly diagnosed smear positive pulmonary TB were exposed to pretomanid either as a single agent at daily doses of 50 to 1200 mg for 14 days or in combination with other anti-TB agents (bedaquiline, moxifloxacin pyrazinamide and/or clofazimine) at a dose of 100 mg or 200 mg for up to 56 days. The overall safety profile determined from the clinical studies completed to date indicates pretomanid is well tolerated in healthy adults and in TB Subjects when administered alone and in combination with moxifloxacin, pyrazinamide, bedaquiline and clofazimine.

In the Phase 1 studies in healthy volunteers the most common side effects or AEs associated with pretomanid exposure include:

- Headache;
- Benign, isolated and reversible elevations of serum creatinine;
- Stomach discomfort (nausea, vomiting, flatulence, and/or diarrhea);
- Skin and subcutaneous tissue disorders.

Key safety considerations of special concern, based on preclinical or clinical findings in the program to date, are noted below and will be under close scrutiny in future trials:

Cataracts

While the detailed examinations in Phase 2 have not raised concern for humans, ophthalmologic examinations, with slit lamp exam and grading of lens opacities, will continue in Nix-TB. These examinations are to follow up on the finding of cataracts in rats exposed to pretomanid in preclinical studies.

Testicular Toxicity

Clinical evaluations of potential testicular toxicity in NC-002 failed to demonstrate any effect, based on evaluations of testosterone, LH and FSH values at baseline and after 2 months of daily dosing of the M-Pa-Z regimen. However to follow up on findings in the male rat of testicular toxicity, all male subjects in the upcoming Phase 3 trial of the M-Pa-Z regimen will have evaluations of male hormones, and a substudy will evaluate changes in semen and sperm parameters in a subset of patients receiving the regimen over 6 months.

Central Nervous System

The pretomanid pre-clinical program identified potential CNS-related toxicities and one Subject treated with pretomanid in clinical study NC-002 experienced a seizure while on treatment. Close surveillance will be in place to identify any seizures or significant central nervous system (CNS) signs or symptoms during the Nix-TB study.

Hepatic Safety

Hepatic enzyme increases have been seen in Subjects treated with pretomanid in combinations with various other medications during the clinical development program. It is difficult to assign specific causality to any one drug within a regimen; nonetheless, the Nix-TB will include specific monitoring of hepatic enzymes.

Detailed Safety Findings in Pretomanid Clinical Development

In Phase 1 trials at the clinical dose of 200mg or lower, the incidence of headache was approximately 20-30% and similar to placebo. At doses of 800mg and higher, usually in trials without a placebo or comparator arm, the incidence of headache reached 80%. Headache occurrence was typically higher in studies with longer confinement periods. Throughout the development program, other common TEAEs include elevated serum creatinine, stomach discomfort (including nausea and other gastrointestinal symptoms such as flatulence and/or diarrhea), and skin and subcutaneous tissue disorders (including erythema, pruritus and rash). Skin and subcutaneous tissue disorders, followed by stomach discomfort were the most commonly reported TEAEs in the Phase 2 monotherapy studies (PA-824-CL-007 and PA-824-CL-010). Within Study PA-824-CL-007, a higher incidence of pretomanid TEAEs were observed in the higher pretomanid dose groups (pretomanid 200 mg: 7%; pretomanid 600 mg: 13%; pretomanid 1000 mg: 31%; and pretomanid 1200 mg: 33%). The incidence of the TEAEs in the Rifafour® treated group was 25%. Study PA-824-CL-010, among the four pretomanid treatment groups (50 mg, 100 mg, 150 mg, and 200 mg) and the Rifafour® treatment group, a higher incidence of TEAEs was observed in the 50mg pretomanid group (66.7%) when compared with Rifafour® (50.0%) and the other pretomanid treatment groups. For the multidose, placebo-controlled Studies PA-824-CL-002 and PA-824-CL-005, overall AE frequency tended to be greater among pretomanid Subjects than among placebo Subjects, and tended to be higher in higher pretomanid dose groups.

Study PA-824-CL-005 was undertaken to determine the mechanism responsible for the elevation in serum creatinine seen with pretomanid dosing in studies PA-824-CL-001 and PA-824-CL-002. This study explored the effects of pretomanid on kidney function by measuring glomerular filtration rate (GFR), effective renal plasma flow (ERPF), filtration fraction (FF, calculated as GFR/ERPF), and creatinine clearance. Subjects were dosed in blinded fashion with placebo, 800 mg pretomanid, or 1000 mg pretomanid for 8 days. Serum creatinine levels rose in both the 800- and 1000-mg/day pretomanid groups, by an average of 0.18 mg/dL (19%) and 0.25 mg/dL (27%) in the two groups respectively by Day 8; the largest individual increase was approximately 40% over baseline. In this study, although serum creatinine levels rose, no meaningful effects were noted during the dosing period on GFR, ERPF, BUN, uric acid or FF. As expected, creatinine clearance was reduced concomitantly with maximally elevated serum creatinine levels relative to baseline. Taken together, these results indicate that pretomanid does not negatively affect renal function. Instead, the drug can be assumed to cause its effects on serum creatinine by inhibiting tubular creatinine secretion; such an effect has been reported with other approved drugs (e.g. cimetidine) and is not considered clinically significant.

Across all studies, the great majority (>~95%) of AEs resolved without sequelae. In Study PA-824-CL-005, one Subject treated with 800 mg pretomanid was discharged with three ongoing AEs (proteinuria [nephrotic range during the study, but non-nephrotic range in follow-up], hypoalbuminemia, and iron deficiency). The proteinuria and hypoalbuminemia were moderate in severity and the iron deficiency was mild. This Subject substantially improved, and the Subject is seen periodically by a nephrologist. A renal biopsy performed 20

months post-study revealed focal segmental glomerulosclerosis likely secondary type, although the Subject remains fundamentally healthy with normal renal function indices and no signs of peripheral edema or hypertension. A complete review of her screening and check-in laboratory values suggests, in the opinion of the Sponsor, that she might have had a pre-existing undiagnosed clinical condition including atypical lipid profile, BUN below the lower limit of the normal range, and ALT and AST above the upper limit of normal range. Furthermore, her eosinophil count was above-normal at Screening at 6.7% and progressively rose during the study to 8.9% by Day 15 and she reported a personal and family history of allergies and rhinorrea. The Investigator considered this individual normal and meeting the protocol entry criteria, and enrolled this Subject.

In most of the completed Phase 2 studies, no Subjects discontinued from the study as a result of AEs. In Study PA-824-CL-002, dosing for all Subjects in the 1000 mg dose group was discontinued on Day 5 in response to rising serum creatinine levels. In Study PA-824-CL-005, one Subject was discontinued for safety reasons in relation to a severe rash that developed approximately 32 hours after the 8th and last dose of pretomanid (1000 mg). The rash symptoms were treated with diphenhydramine, prednisone, and hydroxyzine at various points during the ensuing approximately 9 days until the symptoms completely resolved. In Study PA-824-CL-007, two Subjects (one in the 200 mg/day pretomanid group and one in the control arm) were discontinued as a result of disease-related hemoptysis. Each of these events was classified as an SAE, both resolved with treatment in hospital and neither was considered possibly related to the study drugs. In Study PA-824-CL-010, one Subject was withdrawn due to the SAE of pneumothorax after 4 days' dosing, which resulted in hospitalization and later resolved. The SAE was deemed related to the Subject's concurrent tuberculosis and unrelated to pretomanid.

Post-study follow-up ophthalmic examinations were performed on Subjects and Subjects enrolled in two studies (PA-824-CL-005 [n=30] and PA-824-CL-007 [n=46]) where Subjects received the highest doses of pretomanid for the longest duration among the clinical studies conducted to date. Male and female healthy volunteers were treated at doses up to 1000 mg/day for 8 days in study PA-824-CL-005, and male and female TB Subjects were treated at doses up to 1200 mg/day for 14 days in study PA-824-CL-007. Two ocular events were reported, one cataract was among the 12 Subjects from the 1200 mg pretomanid group in study PA-824-CL-007 and the other cataract was from among the 5 Subjects within the HRZE group.

In NC-001-(B-M-Pa-Z), five Subjects were discontinued prior to completion of their treatment with a pretomanid containing arm. One Subject receiving pretomanid (200mg), moxifloxacin (400 mg), and pyrazinamide (dosed by weight) experienced an SAE considered by the Investigator unrelated to the drug combination. The SAE consisted of convulsion as well as aggressive and violent behaviours. After a CT scan, the Subject was diagnosed with neurocysticercosis. A second Subject receiving pretomanid (200mg), moxifloxacin (400 mg), and pyrazinamide (dosed by weight) was withdrawn on Treatment Day 5 based on a protocol specified criterion of an increase from baseline in QTcF and QTcB greater than 60 msec on repeated ECGs and accompanied by clinically relevant T-wave morphology changes. On Day 5, the Subject had prolonged QTc values (>60 msec) on the pre-dose ECG; however, on repeat ECGs, the QTc values stabilized satisfactorily. Five hours post-dose on Day 5, ECG QTc values were again increased (>60 msec) from baseline and repeat ECGs also revealed T-wave changes. The Subject was, therefore, withdrawn from the study as specified in the protocol. In addition, two Subjects receiving pretomanid (200mg), and pyrazinamide (dosed by weight) were withdrawn due to Grade 3 ALT levels, although the elevation in ALT in one of these Subjects occurred prior to the first dose of study medication. One Subject receiving pretomanid (200 mg) and bedaquiline (400 mg) was withdrawn due to a Grade 3 ALT elevation. Overall in the trial, 53% of the 15 Subjects in the pretomanid + pyrazinamide treatment arm experienced treatment-emergent adverse events, as compared with 53% of the 15 Subjects in the pretomanid + pyrazinamide + moxifloxacin arm and 25% of the 8 Subjects in the HRZE (control) arm. All of these adverse events were rated by the Investigator as mild or moderate. 7% of Subjects in the pretomanid + pyrazinamide treatment arm experienced liver enzyme elevations, as compared with 20% of Subjects in the

pretomanid + pyrazinamide + moxifloxacin arm and 0% in the Rifafour® arm, accounting for most of the imbalance between groups. All liver enzyme elevations were < 3x ULN except for two cases.

Also in NC-001-(B-M-Pa-Z), changes in QT interval were assessed pre-dose and at 2hrs and 5 hrs post-dose on each day of the study for the pretomanid + pyrazinamide and pretomanid + pyrazinamide + moxifloxacin treatment arms. On Day 14, the last dosing day, no Subject in either treatment group had a corrected QT interval (QTcF) > 450 msec. One Subject in the pretomanid + pyrazinamide arm had a QTcF increase of between 30 and 60 msec; no Subject had a QTcF increase > 60 msec. No Subjects in the pretomanid + pyrazinamide + moxifloxacin had a QTcF increase > 30 msec.

Safety Findings in 8-Week Study NC-002

Preliminary data analyses of the study NC-002 indicate that a total of 207 Subjects were enrolled, with 60 randomized to M-PA100-Z, 62 randomized to M-PA200-Z, and 59 to HRZE. An additional 26 Subjects were treated in the M-PA200-Z MDR-TB arm. In this study 88% of all Subjects had a treatment emergent adverse event (TEAE), including 87% in the M-PA100-Z group, 92% in the M-PA-Z group, 85% in the HRZE group and 89% in the M-PA-Z MDR-TB group. Adverse events were graded according to the NIH Division of Microbiology and Infectious Diseases Adult Toxicity Table.

Eleven serious adverse events (SAEs) were reported in 9 Subjects, with one Subject in each of the M-PA100-Z and the HRZE groups, and 7 Subjects in the M-PA200-Z group. The Subject in the M-PA100-Z group died of an unknown cause 39 days after a single dose of study drug regimen and the death was not considered related to study drug by the Investigator or the Sponsor. Four other SAEs were considered not related to study drug, including a pneumothorax, a bone fracture, dyspnea requiring hospitalization, and second degree heart block considered on evaluation to be existing prior to entry in to the trial. SAEs considered possibly related or related to the study drug regimen included hyperuricemia likely secondary from pyrazinamide, drug-induced hepatitis and elevated liver enzymes. One Subject had an episode of agranulocytosis that resolved after the study drug regimen was stopped and one Subject had a seizure witnessed by the family and was discontinued from the study.

The protocol required that Subjects with hepatic enzyme ALT or AST elevations greater than 3X the Upper limit of Normal (ULN) must have study drug discontinued. Consequently, 25 Subjects were withdrawn from the study across the study arms for elevations in hepatic enzymes. The distribution of elevations in ALT across the study arms is presented in Table 5. While more Subjects in the M-PA200-Z group had elevations in ALT >3 – 5X ULN, those with elevations >5X ULN or >8X ULN were fairly evenly distributed across the groups of Subjects with drug-sensitive *M. tb*.

Table 5: NC-002 Elevations in Alanine Aminotransferase

ALT	Statistic	M-PA100-Z (N=60)	M-PA200-Z (N=62)	HRZE Control (N=59)	M-PA200-Z MDR (N=26)
> 3X ULN	N (%)	7	10	5	3
> 5X ULN	N (%)	4	5	4	2
> 8X ULN	N (%)	2	4	3	1

Note: Groups are not mutually exclusive: >3X includes >5X and >8X; >5X includes >8X

Ophthalmologic Evaluations

All Subjects received ophthalmologic evaluations using the AREDS2 grading system across a range of 0-4 including visual acuity testing and slit lamp examinations at baseline and 3 months after completion of study drug dosing. All Subjects enrolled with the required zero score grade for all regions of the lens except for 1 Subject who was blind in one eye. Among all Subjects in the trial, 4 Subjects had lens evaluations with a grade of greater than zero. One Subject in the M-PA100-Z group and 3 Subjects in the M-PA200-Z group had grades of 0.5 or 1.0 in a single eye in one of the 3 zones of the lens. It is unlikely these findings represent a drug-induced lens opacity given the low incidence, the unilateral nature of all findings and the differing zone locations of the findings. It is common in persons with no clinical abnormalities to have grades of 0.5 – 1.0+ in the AREDS2 rating on a slit lamp evaluation.

Reproductive Hormone Evaluations

In study NC-002 men were evaluated with plasma samples for the reproductive hormones LH, FSH and Testosterone at baseline and at the end of the dosing period. If the study drug regimen caused testicular toxicity, the most sensitive measure from these hormones would be an increase in levels of FSH. Among Subjects in the M-PA100-Z group the mean baseline FSH was 9.027 U/L which decreased to 8.338 U/L at the end of therapy. Among Subjects in the M-PA200-Z group the mean baseline FSH was 6.531 U/L at baseline and this decreased to a mean of 6.061 at the end of therapy. Men in the Rifafour® group had a mean baseline of 7.394 U/L which decreased to 6.714 at the end of therapy. This gives relative reassurance that the M-Pa-Z regimen is not likely to cause testicular toxicity in men.

Electrocardiographic Conduction Interval Changes

Subjects in NC-002 had supine resting ECGs taken at baseline, Day 4 and weekly through the 8 week dosing period and 2 weeks after the end of dosing. All ECGs were read by a central cardiology service. No Subjects had a corrected QT interval (QTcF) greater than 500 msec during the study. A small number of Subjects had asymptomatic increases in QTcF from baseline over 60 msec: Two in the M-PA100-Z group, 4 in the M-PA200-Z group, none in the Rifafour® group and 2 in the M-PA200-Z MDR group. An evaluation of the mean change from baseline across all post-baseline ECGs notes increases of 11.1 msec in the Rifafour® group, 11.1 in the M-PA100-Z group, 17.8 msec in the M-PA200-Z group and 6.7 in the M-PA200-Z MDR-TB group. Of note, many Subjects were tachycardic at baseline with their active pulmonary *M. tb.* and had heart rates decrease over the first week of therapy. This fact complicates interpretation of the data based on the QT correction factors that are imperfect when correcting for heart rates that change over time.

2.2.3. Linezolid

Linezolid is a synthetic antibacterial agent of the oxazolidanone class approved in many countries around the world (including South Africa), for drug-resistant, gram-positive bacterial infections, including gram positive organisms such as *Staphylococcus aureus*, coagulase negative *Staphlococcus* and *Enterococcal* infections. The recommended dose for these infections is 600 mg twice daily for up to 28 days of therapy(18, 31, 34). Antimicrobial effects likely come from inhibition of protein synthesis in the ribosomes of the infecting organism(25). Resistance of *M.tb*. to linezolid is rare, as this drug has not been widely used to treat tuberculosis. In a recent study using linezolid to treat patients with XDR-TB in Korea, none of 41 patients had resistance to linezolid at baseline(24).

Preclinical *in vitro* data shows linezolid is active against *Mycobacterium tuberculosis* (*M.tb.*), including MDR strains with minimum inhibitory concentrations (MICs) that range from 0.125-1 μg/mL(26). Recent studies of the bactericidal and sterilizing activity of linezolid in a mouse model of *M.tb.* infection have demonstrated linezolid alone causes marked reductions in lung colony forming units (CFUs) from mice following 1-3 months of therapy(5). (Table 6, below)

Table 6: Murine Lung CFU counts during Treatment with Linezolid monotherapy versus Standard Therapy

	Mean lung log_{10} CFU count (± S.D.) at:			
Regimen	D0	Month 1	Month 2	Month 3
Untreated	6.17 ± 0.27	6.47 ± 0.06		
2RHZ/4RH		3.47 ± 0.37	1.59 ± 0.25	0.50 ± 0.51
L		4.97 ± 0.26		

In recent years linezolid has been used to treat patients with MDR and XDR-TB, although there have been no fully controlled trials of linezolid in a regimen for this indication. The World Health Organization management guidelines place linezolid in Group 5 ("Agents with unclear role in treatment of drug resistant-TB") in their groups of drugs to treat MDR-TB.[27] Over the past 10 years small retrospective observational studies have reported good results when linezolid has been added to failing regimens for patients with MDR-TB[(28, 29, 30)] . The most compelling recent evidence linezolid may be of benefit to patients with XDR-TB was reported by Lee and colleagues from a study in S. Korea[(24)]. Forty-one patients who had sputum culture–positive XDR-TB and who had not had a response to any available chemotherapeutic option during the previous 6 months were randomized to start linezolid at 600 mg daily or to delay therapy with linezolid at 600 mg daily for 2 months without changing their failing background regimen. After confirmed sputum-smear conversion, or at 4 months, patients underwent a second randomization to continued linezolid therapy at a dose of 600 mg per day or 300 mg per day for at least an additional 18 months. Thirty four of 39 (87%) of the patients had a negative sputum culture within 6 months after linezolid had been added to their drug regimen. As of the cutoff date prior to publication, of the 38 patients who received linezolid, 17 were still receiving the treatment per protocol, and 13 had completed treatment, including 6 with no relapse during the treatment period, 4 with no relapse at the 6-month follow-up, and 3 with no relapse at the 12-month follow-up (end of study).

While the standard dose of linezolid for short term use for severe bacterial infections is 600 mg bid, some clinicians and clinical trials using linezolid as Group 5 therapy to treat TB use only 300 mg or 600 mg daily due to concerns about toxicity developing when used over a period of months[(24)] (see below for a review of linezolid toxicity). However, there are no data to indicate what dose of linezolid is required or optimal to effectively treat TB infection. Consequently the TB Alliance has recently conducted and completed an Early Bactericidal Activity trial to evaluate the use of linezolid over 14 days in patients with newly diagnosed DS Pulmonary TB in dosing schedules including 300 mg daily, 300 mg bid, 600 mg daily, 600 mg bid, 1200 mg daily, and HRZE at standard doses daily. Preliminary unpublished in-house results using Bayesian mixed effects modelling have noted that there is a bactericidal effect of linezolid over 14 days that is substantial, but less than for the full HRZE regimen. There is little difference between daily or twice daily dosing of the same total daily dose of drug, and there is a dose-response relationship between total daily dose and daily reductions in either total CFU counts on solid culture or increases in Time to Positivity in liquid culture. Point estimates of the log of the daily increase in Time to Positivity over 14 days ranged from 2.278 for Linezolid 300 mg QD to 4.446 for linezolid 1200 mg qd, with the estimate of 6.860 for HRZE for reference.

Linezolid Clinical Safety

Linezolid is currently marketed globally, including South Africa, for a variety of acute infectious diseases and has been studied for the treatment of XDR-TB in several recent trials, including in South Africa[(24,33)]. The following list of known and potential risks is based on the warnings and precautions and adverse reactions sections of the current package label[(18,31, 34)]. Of note, the approved indication for linezolid is for administration up to 28 days.

Warnings and Precautions

- Linezolid should not be used in patients taking any medicinal product which inhibits monoamine oxidases A or B (e.g. phenelzine, isocarboxazid) or within 2 weeks of taking any such product.

- Myelosuppression (including anemia, leukopenia, pancytopenia, and thrombocytopenia) has been reported in patients receiving linezolid. In cases where the outcome is known, when linezolid was discontinued, the affected hematologic parameters have risen toward pretreatment levels. Complete blood counts should be monitored weekly in patients who receive linezolid, particularly in those who receive linezolid for longer than two weeks, those with pre-existing myelosuppression, those receiving concomitant drugs that produce bone marrow suppression or those with a chronic infection who have received previous or concomitant antibiotic therapy. Discontinuation of therapy with linezolid should be considered in patients who develop or have worsening myelosuppression.
- Lactic acidosis has been reported with the use of linezolid. In reported cases, patients experienced repeated episodes of nausea and vomiting. Patients who develop recurrent nausea or vomiting, unexplained acidosis, or low bicarbonate level while receiving linezolid should receive immediate medical evaluation.
- Spontaneous reports of serotonin syndrome associated with the co-administration of linezolid and serotonergic agents, including antidepressants such as selective serotonin reuptake inhibitors (SSRIs), have been reported. Where administration of linezolid and concomitant serotonergic agents is clinically appropriate, patients should be closely observed for signs and symptoms of serotonin syndrome such as cognitive dysfunction, hyperpyrexia, hyperreflexia and incoordination. If signs or symptoms occur physicians should consider discontinuation of either one or both agents. If the concomitant serotonergic agent is withdrawn, discontinuation symptoms can be observed (see package insert of the specified agent(s) for a description of the associated discontinuation symptoms).
- Peripheral and optic neuropathy has been reported in patients treated with linezolid, primarily those patients treated for longer than the maximum recommended duration of 28 days. In cases of optic neuropathy that progressed to loss of vision, patients were treated for extended periods beyond the maximum recommended duration. Visual blurring has been reported in some patients treated with linezolid for less than 28 days. If patients experience symptoms of visual impairment, such as changes in visual acuity, changes in color vision, blurred vision, or visual field defect, prompt ophthalmic evaluation is recommended. Visual function should be monitored in all patients taking linezolid for extended periods (≥ 3 months) and in all patients reporting new visual symptoms regardless of length of therapy with linezolid. If peripheral or optic neuropathy occurs, the continued use of linezolid in these patients should be weighed against the potential risks. Additional information on the neuropathies reported in recent studies of linezolid administered over prolonged periods to patients with TB infection is presented above in Section 2.2.3.
- Convulsions have been reported in patients when treated with linezolid. In some of these cases, a history of seizures or risk factors for seizures was reported.
- Postmarketing cases of symptomatic hypoglycemia have been reported in patients with diabetes mellitus receiving insulin or oral hypoglycemic agents when treated with linezolid, a reversible, nonselective MAO inhibitor. Some MAO inhibitors have been associated with hypoglycemic episodes in diabetic patients receiving insulin or hypoglycemic agents. While a causal relationship between linezolid and hypoglycemia has not been established, diabetic patients should be cautioned of potential hypoglycemic reactions when treated with linezolid.

Longer term use of linezolid in patients with TB has been limited by the high cost of the drug and concerns about the toxicities of myelosuppression, peripheral neuropathy and optic neuropathy. Published reports of observational trials and case series note use of linezolid at doses ranging from 300 mg daily to 1200 mg daily over many months. The most complete review is a meta-analysis by Cox which noted the proportion of adverse events necessitating treatment discontinuation was significantly different by dose: 29.49% (95%CI 3.24–55.74) for ⩽600 mg daily vs. 60.75% (95%CI 42.69–78.81) for >600 mg daily ($P = 0.05$) [33].

The linezolid product label[18, 31, 34] notes that "*In clinical trials 2.4 % of patients developed a platelet count less than 75% of the LLN/baseline. Thrombocytopenia appears to be dependent on duration of therapy, (generally greater than 2 weeks of treatment).*" *The label notes also, "In cases where the outcome is known, when linezolid was discontinued, the affected hematologic parameters have risen toward pre-treatment levels."*

In the trial of Lee et al in S Korea[24], seven of 41 Subjects had myelosuppression, including anemia and neutropenia, primarily within the first 5 months, and only one Subject withdrew due to anemia. Six had clinically significant myelosuppression: 5 in 0-4 months and 1 in 4-8 months, with 0 in 8-12 months.

Peripheral and Optic Neuropathy:

The linezolid product label notes these adverse events have been " *…reported in patients, primarily those patients treated for longer than the maximum recommended duration of 28 days. In cases of optic neuropathy that progressed to loss of vision, patients were treated for extended periods beyond the maximum recommended duration. Visual function should be monitored in all patients taking ZVYOX for extended periods (≥3 months) and in all patients reporting new visual symptoms, regardless of length of therapy*[32].

In Lee, NEJM, 2012[24], the publication's Supplemental Table 3 notes that 21 patients had clinically significant peripheral neuropathy spread over 12 months: 5 in months 0-4, 10 in months 4-8 and 5 in months 8-12 (time of onset not noted for one). Subjects who developed any peripheral neuropathy had their dosing of linezolid interrupted, generally for several weeks, and then resumed at the lower dose of 300 mg/day (C. Barry, personal communication). None of the Subjects withdrew from the study based on peripheral neuropathies. At baseline, patients received visual acuity testing, contrast sensitivity and color vision tests. Seven cases were observed as having potential effects on vision; only two of 38 patients withdrew from study due to optic neuropathy. For clinically significant optic neuropathy, one had this at 0-4 months, 2 at 4-8 months and 3 at months 8-12. Except for the 2 Subjects who withdrew from the study, the others resumed linezolid at the 300 mg dose after a hiatus of several weeks of treatment and completed the study with resolution of their visual acuity changes (C. Barry, personal communication).

In the Schecter California DOH review[29] peripheral neuropathy developed in 5 of 30 patients (no standardized monitoring), but only one withdrew from linezolid therapy. One patient developed visual loss secondary to optic neuropathy after 10 months of linezolid therapy, but vision returned to normal 3-4 weeks after discontinuation.

In Park, 2006[30], two patients of eight in the case series developed optic neuropathy after 8-9 month and had linezolid discontinued; these patients also had peripheral neuropathy. After linezolid treatment was stopped, the optic neuropathy fully resolved after 2-3 months. A total of 4 patients developed peripheral neuropathy at 4, 5, 8, 11 months; in the patients with optic neuropathy who stopped treatment, the peripheral neuropathic symptoms continued or improved only marginally.

In Singla, 2012[30], two of 29 patients treated with linezolid, 600 mg daily over 12 months, stopped the drug because of peripheral neuritis (one patient) and optic neuritis (one patient). The time course of these adverse events was not noted.

2.3. Regimens to be Studied

The regimen included in this study (B-L-Pa) has been selected based on the performance of the regimen in non-clinical pharmacology studies and on the combination of bedaquiline and pretomanid with other drugs in clinical studies NC-001 and NC-003. In addition, improved treatment outcomes in XDR patients with the addition of linezolid to existing therapy provide support for combining linezolid with other drugs that have no pre-existing resistance.

This regimen has the potential to treat drug resistant strains of tuberculosis. This is an oral regimen, removing the need for injectables as part of drug resistant treatment, and is also projected to be markedly less expensive than current XDR-TB therapy. Treatment duration is anticipated to be shorter than current regimens for drug resistant

TB, based on findings in mouse models of infection and the fact that all Subjects will be treated with three active drugs against TB for which there is no expected resistance.

The key data supporting the use of the B-L-Pa regimen are described below.

2.3.1. Non-Clinical Studies

In the murine model of TB, addition of bedaquiline to HRZ results in accelerated clearance of *M.tb* [4,5] when compared to HRZ alone. While the combination of bedaquiline and pretomanid in the murine model of TB appeared somewhat antagonistic relative to bedaquiline alone, it was as active as the triple combination of HRZ[10] and in a subsequent study it was more active in the mouse model than HRZ[11]. Thus a novel regimen with bedaquiline plus pretomanid core could be effective in the treatment of MDR-TB by providing two novel drugs for which there is no known pre-existing resistance.

Recent studies of the bactericidal and sterilizing activity of linezolid in an animal model where mice were given high dose aerosol *M.tb.* infection have demonstrated that L alone and in combination with bedaquiline and pretomanid causes marked reductions in lung colony forming units (CFUs) from mice following 1-3 months of therapy (Table 7, below). Additionally, all mice treated daily with bedaquiline, pretomanid and linezolid (B-L-Pa) were cured of the infection after 3 months of therapy as evidenced by no *M.tb.* cultured from lungs when mice were sacrificed 3 months after the completion of therapy that lasted 3 months or more (Tables 7 and 8, below). That is in contrast to the 6 months required to cure all mice when treated with the standard of care isoniazid, rifampicin and pyrazinamide (HRZ; note that typically ethambutol is not used in the mouse model of infection). Additional mouse studies were done to determine whether shorter durations of an oxazolidinone such as Linezolid, with continuation of the other drugs, would result in relapse-free cure in the mouse (Table 8 below). Treatment with linezolid for the first 4- 8 weeks of a three month treatment also resulted in relapse-free cure when lungs from the mice were cultured 3 months after the completion of therapy.[5]

Table 7: Lung CFU counts assessed during Treatment and proportion of mice relapsing after treatment completion

	Mean (±SD) $\log_{10}$ CFU count at[a]:					Proportion (%) relapsing after treatment for:		
Drug Regimen	D-13	D0	M1	M2	M3	2 mos	3 mos	4 mos
Untreated	2.69±0.13	6.17±0.27	6.47±0.06					
2RIF+INH+PZA/ RIF+INH			3.47±0.37	1.59±0.25	0.50±0.51		13/15 (87)	1/20 (5)
BDQ			3.24±0.25					
PMD			4.57±0.22					
LZD			4.97±0.26					
SZD			3.85±0.37					
BDQ+PMD			4.21±0.40	1.62±0.19	0.52±0.36	15/15 (100)	10/15 (60)	2/20 (10)
BDQ+LZD			2.82±0.15	1.91±0.66				
BDQ+SZD			2.88±0.07	0.65±0.50				
PMD+LZD			3.23±0.41	1.48±0.12				
PMD+SZD			1.65±0.33	0.23±0.40				
BDQ+PMD+LZD			3.28±0.65	0.34±0.41	0.00±0.00	12/15 (80)	0/14 (0)	0/20 (0)
BDQ+PMD+SZD			0.94±0.14	0.00±0.00		14/20 (70)	1/14 (7)	

[a] Time points are shown in days (e.g., D-13, day-13; D0, day 0) or months (e.g., M1, 1 month) of treatment.

RIF=rifampicin, INH=isoniazid, PZA=pyrazinamide, BDQ=bedaquiline, PMD=pretomanid, LZD=linezolid, SZD=sutezolid (experimental oxazolidanone)

Table 8: Murine Relapse Data, Impact of L Treatment Duration Lung CFU counts assessed during treatment and proportion of mice relapsing after treatment completion

	Mean (±SD) $\log_{10}$ CFU count at[a]:				Proportion (%) relapsing after treatment for:	
Regimen	D-13	D0	M1	M2	2 mos	3 mos
Untreated	3.96±0.08	7.74±0.20				
2RIF+INH+PZA/ 1RIF+INH				1.94±0.27		8/14 (57)
BDQ+PMD			4.48±0.20	2.33±0.30		3/14 (21)
BDQ+PMD+TZD			4.20±0.13	1.67±0.41		
BDQ+PMD+AZD			4.07±0.36	1.43±0.36		
BDQ+PMD+RWJ			3.63±0.18	0.54±0.41		
BDQ+PMD+LZD$_{50}$			3.48±0.36	0.39±0.26		
1BDQ+PMD+LZD$_{100}$/ BDQ+PMD			2.69±0.37	0.93±0.49	9/15 (60)	0/15 (0)
2BDQ+PMD+LZD$_{100}$/ BDQ+PMD				0.66±0.39	6/15 (40)	0/15 (0)
2BDQ+PMD+LZD$_{100}$/ BDQ+PMD+LZD $_{50}$						0/12 (0)
BDQ+PMD+LZD$_{100}$						0/15 (0)
BDQ+PMD+SZD			1.88±0.22	0.00±0.00	1/14 (7)	0/14 (0)

[a] Time points are shown in days (e.g., D-13, day-13; D0, day 0) or months (e.g., M1, 1 month) of treatment.

* 2BDQ+PMD+LZD$_{100}$/BDQ+PMD means 2 months on the full regimen, and then just J and Pa for the 3rd month.
*1BDQ+PMD+LZD$_{100}$/2BDQ+PMD means 1 month on the full regimen, and then just J and Pa for the 2nd and 3rd months.
*LZD$_{100}$ means a 100 mg/kg dose to the mice, which corresponds to the drug exposure attained by humans taking 600 mg twice daily.

In conclusion, linezolid increases the sterilizing activity of the bedaquiline-pretomanid combination; no *M.tb.* could be cultured from the lungs of mice 3 months after cessation of 3 months of treatment with the combination in contrast to *M.tb.* cultured from 13 of 15 mice treated with the standard 2RHZ/4RH regimen over 3 months. In addition, limiting the duration of L to the first month of treatment does not affect L's contribution to the sterilizing activity of the regimen in the preclinical mouse study.

Prior to the use of pretomanid in combination with bedaquiline in clinical study NC-001, a preclinical cardiovascular safety pharmacology study was conducted in unrestrained beagle dogs with both drugs to explore the potential for additive effects on QT prolongation induced by the combination. Results indicate that administration of 100 mg/kg bedaquiline daily for 7 days causes a small increase in QTc interval by Day 6 in some animals that is not influenced by the addition of 100 mg/kg pretomanid on Day 7. The effect of pretomanid dosing alone on QT interval appeared to be due to discomfort related to the subcutaneous route of administration and not related to the plasma exposure.

2.3.2. Clinical Study NC-001

Study NC-001 was a partially double-blind, randomized, parallel group study in adult male and female subjects with newly diagnosed, uncomplicated, smear-positive, pulmonary TB. A total of 85 subjects met study eligibility criteria and were randomly assigned to one of the following six treatment groups: bedaquiline alone; bedaquiline + pyrazinamide; bedaquiline + pretomanid 200 mg; pretomanid 200 mg + pyrazinamide; pretomanid 200 mg + pyrazinamide, + moxifloxacin; or Rifafour e-275. All study treatments were given once daily for 14 days. Substantial EBA activity was demonstrated across subjects in all arms of the study and the daily reductions in cultured colony counts per mL of sputum are presented in Table 9 below.

Table 9: Summary Statistics for EBACFU$_{(0-14)}$ Derived Using Bi-Linear Regression, Study NC-001.

Treatment Group	N	Daily Mean (SD) $EBA_{CFU(0-14)}$
Pretomanid + pyrazinamide + moxifloxacin	13	0.23 (0.128)
Pretomanid + pyrazinamide	14	0.15 (0.040)
Pretomanid + bedaquiline	15[a]	0.11 (0.050)
Bedaquiline alone	14	0.07 (0.068)
Bedaquiline + pyrazinamide	15	0.13 (0.102)
Rifafour e-275	10	0.14 (0.094)

There were no Serious Adverse Events from the study among subjects treated with pretomanid and bedaquiline. Three Subjects in a bedaquiline-containing treatment arm were withdrawn: one subject on a bedaquiline only arm for a Grade 3 ALT and GGT elevation although the elevation occurred prior to the first dose of study medication: one on a bedaquiline plus pyrazinamide (weight banded) arm for a Grade 3 ALT and AST elevation, and one on a pretomanid and bedaquiline arm for to a Grade 3 ALT elevation.

2.3.3. Clinical Study NC-003

Efficacy

In the 14 day EBA study NC-003 two monotherapy and four different combinations of bedaquiline, pretomanid, pyrazinamide and clofazimine were evaluated in DS-TB subjects. Fifteen Subjects were randomized into 7 treatment arms: C, Z, B-Pa-Z-C, B-Pa-Z, B-Pa-C, B-Z-C, and HRZE control. This study demonstrated no EBA for the clofazimine monotherapy arm and modest EBA for the pyrazinamide monotherapy arm. However, all of the experimental regimens demonstrated EBA. In general, adding clofazimine to the various agents resulted in either no increase in EBA, or a decrease when compared to a similar regimen that did not include clofazimine. In this study, the experimental regimen with the best EBA was B-Pa-Z which demonstrated a rate of decrease in both logCFU and logTTP that was at least as good as the HRZE control. The daily logCFU results are presented in Table 10. Similar results were found when TTP was used to calculate the bactericidal activity over 14 days (BA(0-14)).

Table 10: NC-003 Efficacy Results: Daily $BAlogCFU_{(0-14)}$

Arm	logCFU
BPaZC	.124
BPaZ	.180
BPaC	.086
BZC	.098
Z	.036
C	-.025
Rifafour®	.152

Safety

Generally, the regimens in this study were well tolerated. Table 11 provides a list of the overall safety findings. The only SAE experienced in the study was in a Subject in the clofazimine monotherapy arm. Otherwise, the rates of treatment emergent AEs (TEAEs) were similar across the treatment arms. One Subject in the B-Pa-Z arm was withdrawn from the study due an adverse event of increased liver function tests (ALT, AST and GGT).

Table 11: NC-003 Safety Data

	BPaZC	BPaZ	BPaC	BZC	Z	C	HRZE	Total
**N**	15	15	15	15	15	15	15	105
**Subjects with:**								
**TEAEs**	11	9	8	10	10	9	8	65
**TEAEs leading to death**:								
**Serious TEAEs**						1		1
**TEAES leading to early withdrawal**		1						1
**TEAEs leading to discontinuation of study drug**		1						1
**Drug-related TEAES**	8	5	7	3	5	6	5	39
**Serious, drug-related TEAEs**								
**Grade III AEs**		2	1	2		1		6
**Grade IV AEs**		1	1					2
**Grade II/IV AEs**		2	1	2		1		6

QT Prolongation

Because bedaquiline and clofazimine are both known to prolong the QT interval, intensive ECG monitoring was included in the study endpoints. The mean change from baseline in QTcB and QTcF tended to be larger at 5 hours than at 10 hours post-dose in the (B-Pa-Z-C) arm and in the (B-Pa-C) arm. No QTcB or QTcF ≥500 ms were reported. An increase from baseline to Visit 5 and subsequent visits of ≥60 ms in QTcB was reported for 2 Subjects in the (B-Pa-C) arm and for 1 Subject in the clofazimine alone arm. An increase from baseline to Visit 5 and subsequent visits of ≥60 msec in QTcF was reported for 4 Subjects in the (B-Pa-C) arm and for 1 Subject in the clofazimine alone arm. For both QTcB and QTcF, the (B-Pa-Z-C) arm and the (B-Pa-C) arm showed the largest increase from baseline. Clofazimine will not be used in any treatment arms in the current Nix study.

2.4. Overall Benefit/Risk Assessment

The recent report of the long term outcome of patients with XDR-TB treated in S. Africa highlighted the very poor prognosis for patients with this disease. After 60 months of follow up 73% of 107 patients had died and only 11% had a favourable outcome[(23)]. These patients have infection with *M.tb.* that is resistant to many/most of the available drugs to treat tuberculosis. Patients with XDR-TB have limited treatment options due to their resistance profile, and the drugs that are typically used in Standard of Care have many side effects, some are administered as injectables and have poor treatment outcomes in XDR-TB. This trial provides an opportunity to treat patients with XDR-TB with three active drugs, for which there is no or minimal pre-existing resistance, in a very closely controlled and monitored clinical trial setting. Subjects will be monitored closely and regular reviews of safety and efficacy will be made by the DSMC. While this is an untested combination regimen in patients with XDR-TB, this regimen has the potential to give relapse-free cure of XDR-TB with a simple regimen in a much shorter period of time than currently required by the available drugs used in the best standard of care. Preclinical studies of this regimen in a murine model of infection demonstrated relapse free cure of *M.tb.* in half the time (3 vs 6 months) required by standard HRZ therapy. Clinical studies of linezolid alone and pretomanid and bedaquiline alone and in combination have demonstrated activity against TB infection.

These three drugs have not been used in combination in humans and thus their combined toxicity profile is not known. There is limited experience with both B and Pa to date and thus their safety profile is emerging. The greatest risks of key concern for subjects in this trial from linezolid are from the adverse events of

myelosuppression and peripheral and optic neuropathy. Subjects will be closely monitored with full blood counts, vision examinations, and screening for peripheral neuropathy. The investigator may interrupt dosing of either linezolid or linezolid with pretomanid and bedaquiline if adverse events of concern develop, and a resumption of the drugs, with linezolid at the same or at a lower dose, or without linezolid if the subject received at least 4 weeks of the 1200 mg total daily dose, may be made cautiously. Subjects will be under close surveillance for hepatotoxicity, as that risk for pretomanid and bedaquiline is not yet well characterized. Other adverse events of special concern are seizures or other neurologic events. Seizures have been reported in patients taking linezolid, seizures have been noted in animal toxicology studies of pretomanid at higher doses, and one unexplained seizure was noted in a patient taking M-Pa-Z in Study NC-002.

Overall the benefit-risk balance justifies evaluating the B-L-Pa regimen in this study, with the cautious surveillance in place, to treat patients with XDR-TB who have few options for a successful outcome.

3. TRIAL RATIONALE AND OBJECTIVES

3.1. Trial Rationale

This trial will provide a regimen containing 3 drugs against which there is no expected *M.Tb.* resistance in the community for patients with limited treatment options while simultaneously gathering important efficacy and safety data on a regimen that could potentially treat all strains of *M.tb*. Data from previous trials shows that the combination of B-Pa is well tolerated and has the potential to shorten treatment in Subjects who are susceptible to all drugs. The addition of linezolid will ensure each Subject receives at least 3 drugs active against their TB strain.

3.2. Dose Rationale

3.2.1. Bedaquiline

Bedaquiline will be administered as the dose regimen currently approved by the United States Food and Drug Administration for treatment of patients with MDR-TB: 400mg once daily for Days 1-14 followed by 200mg three times per week for the remainder of treatment.

3.2.2. Pretomanid

Pretomanid has demonstrated good microbicidal activity at the 200mg daily dose as monotherapy in studies PA-824-CL-007 and PA-824-CL-010, in combination with either bedaquiline or pyrazinamide over 14 days in the EBA Study NC-001-(B-M-Pa-Z) and in combination with either bedaquiline and/or pyrazinamide and/or clofazimine over 14 days in the EBA Study NC-003-(B-C-Pa-Z). In the EBA Study PA-824-CL-010 the 100mg dose demonstrated similar microbicidal activity to the 150 and the 200mg daily dose over 14 days. The Phase 2 trial NC-002-(M-Pa-Z) evaluated this regimen at doses of pretomanid of both 100 mg and 200 mg relative to the HRZE control. In this trial the efficacy results were similar between Subjects treated with 100 mg/day and 200 mg/day of pretomanid in the regimen, although for the primary endpoint, reduction in colony forming units of *M.tb.* from sputum, only the 200 mg/day dose group was statistically significantly better than the group randomized to standard HRZE therapy. Safety was also similar between the groups, although the 200 mg/day group had more grade 2 adverse events than either the 100 mg/day group or the HRZE control group. Consequently, in an upcoming Phase 3 trial for this regimen, the PaMZ regimen will be evaluated at both the doses of 100 mg/day and 200 mg/day. However because sterilizing relapse-free cure of TB in patients with XDR-TB may ultimately require a regimen with higher drug exposures, the 200mg dose has been chosen for this study.

3.2.3. Linezolid

The standard dose of linezolid for a multitude of indications is 400mg or 600 mg BID. Doses of linezolid used in reported observational trials and case series range from 300 mg to 1200 mg per day over periods of up to 20 months of treatment. While the development of adverse events is generally higher with higher doses, the adverse events often ameliorate with a reduction of the dose or discontinuation of drug for several weeks and then reintroduction at a lower dose. No controlled trials have clearly identified differences in anti-TB effect across a range of doses. This trial initially started all subjects on 600 mg bid of linezolid, the approved dose to treat bacterial infections for up to 28 days. Preliminary unpublished in-house top line results from a recently completed 2 week study sponsored by the TB Alliance have demonstrated that the Bactericidal effects of linezolid over 14 days are greatest at a total daily dose of 1200 mg and lowest at a 300 mg total daily dose (see Section 2.2.3 for more detail). This gives further rationale to begin treating all trial participants at the full daily dose of 1200 mg. Because toxicity is thought to be caused by mitochondrial toxicity and may be lessened by lowering the time the linezolid concentration is greater than the threshold for mitochondrial toxicity, the protocol has been amended to require all participants to begin treatment with linezolid at the 1200 mg qd single daily dose. If adverse events develop, the investigator will be able to interrupt dosing or to reduce the dose level to either 600 mg qd or 300 mg qd in an effort to allow this patient population with high mortality on standard care to continue to benefit from the study drug regimen. If subjects have toxicity issues with linezolid that would prohibit further treatment with that drug, they can remain on the bedaquiline and pretomanid study IMP if they received the initial 1200 mg total daily dose of linezolid for at least the first 4 consecutive weeks of treatment and they are smear negative or with trace/scanty results and judged to be clinically improving by the Investigator.

3.3. Trial Objectives

To evaluate the efficacy, safety, tolerability and pharmacokinetics of bedaquiline plus pretomanid plus linezolid after 6 months of treatment (with an option to treat for 9 months in Subjects who are culture positive or revert to being culture positive between month 4 and month 6 visits) in Subjects with either pulmonary XDR tuberculosis, treatment intolerant or non-responsive multi-drug resistant tuberculosis (MDR-TB).

4. TRIAL DESIGN

4.1. Summary

Up to 200 male and female Subjects aged 14 and over with confirmed sputum positive for *M.tb.* in culture pulmonary XDR-TB, or with pulmonary MDR-TB with a documented intolerability or non-response to the best treatment available for 6 months or more will be enrolled.

All Subjects will have up to a maximum of 9 days for screening, receive 6 months of treatment, and have follow-up visits performed 1 and 2 months after treatment completion and every 3 months after study treatment completion for 24 months. If a Subject is culture positive or revert to being culture positive between Month 4 and Month 6 visits and their clinical condition suggests they may have ongoing TB infection, they may have treatment extended to 9 months (with 24 months of Follow Up) or be withdrawn from the study. If culture results between month 4 and 6 are contaminated, missing or considered an isolated positive without clinical significance, please use available culture results to make this decision and consult with the TB Alliance Medical Monitor. All decisions regarding treatment extension should be discussed with the TB Alliance Medical Monitor before implementation.

Subjects who withdraw after ≤14 days of IMP should attend an Early Withdrawal visit. Subjects who withdraw after ≥15 days of IMP should return for an Early Withdrawal visit and follow-up visits at 3, 6 and 24 months after their last dose of IMP to check for survival, SAEs and resolution of TB symptoms.

Subjects will receive:

- B-L-Pa for the duration of treatment.

Figure 1: Trial Schematic

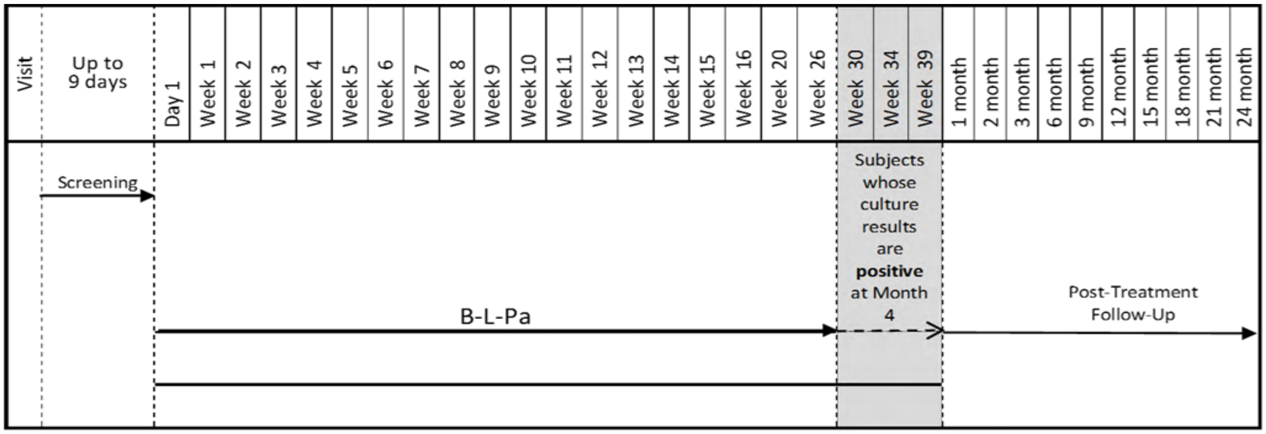

4.2. Trial Endpoints

4.2.1. Primary Endpoint

Incidence of bacteriologic failure or relapse or clinical failure through follow up until 6 months after the end of treatment.

Abbreviated Definitions (full definitions will be described in the Statistical Analysis Plan (SAP)):

- Bacteriologic failure: During the treatment period, failure to attain culture conversion to negative.
- Bacteriologic relapse: During the follow-up period, failure to maintain culture conversion to negative status in culture, with culture conversion to positive status with a *Mycobacterium tuberculosis* (*M.tb*.) strain that is genetically identical to the infecting strain at baseline.
- Clinical failure: A change from protocol-specified TB treatment due to treatment failure, retreatment for TB during follow up, or TB-related death.

Note:

- Culture conversion requires at least 2 consecutive culture negative/positive samples at least 7 days apart.
- Subjects who are documented at a visit as unable to produce sputum and who are clinically considered to be responding well to treatment will be considered to be culture negative at that visit.

4.2.2. Secondary Endpoints

4.2.2.1. Efficacy:

- Incidence of bacteriologic failure or relapse or clinical failure through follow up until 24 months after the end of treatment as a confirmatory analysis
- Time to sputum culture conversion to negative status through the treatment period.
- Proportion of subjects with sputum culture conversion to negative status at 4, 6, 8, 12, 16 and 26 or 39 weeks.
- Linezolid dosing (actual) and efficacy will be explored.
- Change from baseline TB symptoms.

- Change from baseline in Patient Reported Health Status.
- Change from baseline weight.

4.2.2.2. Safety and Tolerability:

- All cause mortality.
- Incidence of Treatment Emergent Adverse Events (TEAEs) will be presented by severity, (DMID Toxicity Grade), drug relatedness and seriousness, leading to early withdrawal and leading to death.
- Quantitative and qualitative clinical laboratory result measurements, including observed and change from baseline.
- Quantitative and qualitative measurement of ECG results, including observed and change from baseline
- Descriptive statistics of ophthalmology slit lamp examination data (age related eye disease study 2 [AREDS2] lens opacity classification and grading). Categorical data for lens opacity will be summarized in a frequency table for the right and left eye, respectively, including change from baseline.
- Changes in ophthalmic exam for visual acuity and color vision, including observed and change from baseline.
- Changes noted in peripheral neuropathy signs and symptoms, including observed and change from baseline.
- These data will be presented as descriptive analyses, and no inferential tests will be carried out.

4.2.2.3. Pharmacokinetics:

Pharmacokinetics will consist of two separate schedules:

- All Subjects- Pre-dose sampling at weeks 2, 8 and 16 to measure C_{trough} levels of bedaquiline, bedaquiline metabolite M2, linezolid and pretomanid.
- PK Sub-study Subjects- in addition to the C_{trough} samples, there will be intensive PK sampling at Week 16 at pre-dose, 0.5, 1, 2, 4, 8 12, 12.5, 13, 14, 16, 20 and 24 hours after dosing in a sub-group of 20 evaluable Subjects across selected sites.

For the PK sub-study samples, the following PK parameters will be estimated from the individual (per Subject) PK plasma concentrations: Minimum observed PK plasma concentration (C_{min}), maximum observed PK plasma concentration(C_{max}), time to reach C_{max} obtained without interpolation (T_{max}), area under the PK plasma concentration time (t) curve from zero to the last quantifiable PK plasma concentration prior to the subsequent dose, using the linear trapezoidal rule ($AUC_{(0-t)}$), area under the PK plasma concentration time (t) curve from zero to 24 hours ($AUC_{(0-24)}$). Oral apparent clearance (CL/F) by non-compartment model. These will be derived for each analyte. In addition, for analyte linezolid at BID dose, the AUC_{0-12}, C_{max}, C_{min}, CL/F and $t_{1/2}$ will be calculated based on dose interval 0-12 hrs.

4.2.2.4. Exploratory

- Evaluate whether any of the secondary endpoints predicts relapse free cure.
- Subgroup analyses of the primary endpoint on the MITT analysis population will be considered
- Correlation of Time over mitochondrial protein synthesis inhibition (MPS50) with linezolid toxicity, (the MPS50 value will be an assumed value from literature).

4.2.2.5. General Mycobacteriology

Early Morning and Coached Spot Sputum Samples will be obtained at all scheduled visits, except the Screening Visit when only a Coached Spot Sputum Sample will be collected. Both (if feasible) sputum samples (Coached Spot and Early Morning; or two spot sputum if Early Morning is unavailable) collected will be used for identification for the presence or absence of *M.tb.* in culture and if liquid culture in the MGIT platform is used, for TTP in liquid medium.

The following mycobacteriology assays will be carried out according to procedures described in the Laboratory Manual:

Table 12: General Mycobacteriology

**Sample**	**Type**	**Assessments**
**Screening**	Coached Spot Sputum Sample	• Culture for presence or absence of M.tb.;<br>• Smear microscopy for acid-fast bacilli (AFB);<br>• Gene Xpert, Hain Assay MTBDRplus or an alternative molecular or antigen-based test to confirm *M.tb.*
**Baseline**<br>**(Day 1)**	Early Morning and Coached Spot Sputum Samples	• Culture for presence or absence of *M.tb*.;<br>• If liquid culture in the MGIT platform is used, TTP in liquid medium;<br>• Speciation of the infecting organism by molecular / antigen test, to be done at study lab (lab where study samples are initially sent from site for culture).<br><br>Only one sample should be used for testing at the Central Lab(s) for mycobacterium characterization tests outlined below. The early morning sputum is the most preferable, but if it is not available or the culture is contaminated, then the coached spot may be sent to the Central Lab(s).<br><br>• MIC: bedaquiline, linezolid and pretomanid;<br>• Drug susceptibility testing in liquid culture for rifampicin, isoniazid, streptomycin, ethambutol, pyrazinamide and second line TB drugs including fluoroquinolones and injectables;<br>• Extraction of bacterial (*M.tb*.) DNA for molecular genotyping;<br>•
**All Visits Post Baseline**	Early Morning and Coached Spot Sputum Samples	• Culture for presence or absence of *M.tb.;*<br>• If liquid culture in the MGIT platform is used, TTP in liquid medium.<br>• For any culture positive at or after month 4, Speciation of the infecting organism by molecular / antigen test to be done at study lab (lab where study samples are initially sent from site for culture).<br>• If participant has received at least 4 consecutive weeks of linezolid at a total daily dose of 1200 mg, and Investigator would like to consider discontinuing linezolid dosing and continuing bedaquiline and pretomanid dosing: A smear microscopy for acid fast bacilli (AFB) should be requested by the site and performed at the study lab.

Sample	Type	Assessments
**First culture positive sample at or following end of treatment. If the first positive turns out to be contaminated, then a subsequent positive, if available, should be sent for the characterization.**	Early Morning or Coached Spot Sputum Sample	The following (Early Morning Sputum OR two Coached Spot Sputum sample) will be processed at the central lab(s):<br>• MIC: bedaquiline, linezolid and pretomanid;<br>• Drug susceptibility testing in liquid culture for rifampicin, isoniazid, streptomycin, ethambutol, pyrazinamide and second line TB drugs including fluoroquinolones and injectables;<br>• Extraction of bacterial (*M.tb.)* DNA for molecular genotyping.<br>• Speciation of the infecting organism by molecular / antigen test, to be done at study lab (lab where study samples are initially sent from site for culture) for initial relapse (first positive at end of treatment or during follow-up) or any positive at or after the week 16 visit.

- The extracted *M.tb.* DNA and isolates will be stored for potential further work to validate new assay tools for a maximum of 5 years after trial closure.
- Both the Early Morning and the Coached Spot sputum samples or two spot sputum if Early Morning is not available should be cultured for the culture/MGIT when feasible. If only one sample is cultured, the other should be kept as a back-up sample for use when the other sample is contaminated.
- Samples for mycocharacterization testing done at central myco lab(s) will be batched and sent for testing routinely. Only one sample should be sent for this testing, preferable the early morning.
- If a subject has a positive culture at or after end of treatment, only the first positive culture will be used for the mycocharacterization at the central myco lab(s). If the first positive culture is contaminated, a later positive culture, if available, may be used for characterization
- If the Subject was treated with study medication for less than 14 days, the mycobacteriology testing will be performed on the baseline sample isolate only.

4.3. Trial Population

4.3.1. Inclusion Criteria

1. Provide written, informed consent prior to all trial-related procedures (if under 18, include consent of legal guardian).
2. Body weight of ≥35 kg (in light clothing and no shoes).
3. Willingness and ability to attend scheduled follow-up visits and undergo study assessments
4. Provide consent to HIV testing (if an HIV test was performed within 1 month prior to trial start, it should not be repeated as long as documentation can be provided [ELISA and/or Western Blot]. If HIV status is a confirmed known positive, repeated HIV test is not needed provided documentation is available.
5. Male or female, aged 14 years or above.
6. Subjects with one of the following pulmonary TB conditions:
    a. XDR-TB with
        i. documented culture positive (for *M.tb.*) results within 3 months prior to screening or *M.tb.* confirmed in sputum based on molecular test within 3 months prior to or at screening;
        ii. documented resistance to isoniazid, rifamycins, a fluoroquinolone and an injectable historically at any time or at screening;
    b. MDR-TB documented by culture positive results (for *M.tb.*) within 3 months prior to or at screening with documented non-response to treatment with the best available regimen for 6

months or more prior to enrolment who in the opinion of the Investigator have been adherent to treatment and will be adherent to study regimen;

c. MDR-TB documented by culture positive (for *M.tb.*) results within 3 months prior to or at screening who are unable to continue second line drug regimen due to a documented intolerance to:
   i. PAS, ethionamide, aminoglycosides or fluoroquinolones;
   ii. Current treatment not listed above that renders subject eligible for the study in the Investigator's opinion.

7. Chest X-Ray picture (taken within a year prior to screening) consistent with pulmonary TB in the opinion of the Investigator.
8. Be of non-childbearing potential or using effective methods of birth control, as defined below:

Non-childbearing potential:

a. Subject - not heterosexually active or practices sexual abstinence; or
b. Female Subject/sexual partner - bilateral oophorectomy, bilateral tubal ligation and/or hysterectomy or has been postmenopausal with a history of no menses for at least 12 consecutive months; or
c. Male Subject/sexual partner - vasectomised or has had a bilateral orchidectomy minimally three months prior to Screening.

Effective birth control methods:

A double contraceptive method should be used as follows:

a. Double barrier method which can include any 2 of the following: a male condom, diaphragm, cervical cap, or female condom (male and female condoms should not be used together); or
b. Barrier method (one of the above) combined with hormone-based contraceptives or an intra-uterine device for the female Subject/partner;
c. And are willing to continue practicing birth control methods throughout treatment and for 6 months (both male and female Subjects) after the last dose of study medication or discontinuation from study medication in case of premature discontinuation.

Note: Hormone based contraception alone may not be reliable when taking IMP; therefore, hormone based contraceptives alone cannot be used by female Subjects or female partners of male Subjects to prevent pregnancy.

4.3.2. Exclusion Criteria

Medical History

1. Any condition in the Investigator's opinion (i.e., an unstable disease such as uncontrolled diabetes or cardiomyopathy, extra-pulmonary TB requiring extended treatment), where participation in the trial would compromise the well-being of Subject or prevent, limit or confound protocol specified assessments.
2. Abuse of alcohol or illegal drugs, that in the opinion of the Investigator would compromise the Subjects' safety or ability to follow through with all protocol-specified visits and evaluations.
3. In the judgment of the Investigator, the patient is not expected to survive for more than 12 weeks.
4. Karnofsky score < 50 within 30 days prior to entry.
5. Body Mass index (BMI) < 17 kg/m^2

6. History of allergy or known hypersensitivity to any of the trial Investigational Medicinal Products or related substances.
7. HIV infected Subjects having a CD4+ count ≤ 50 cells/µL;
   For HIV infected Subjects having a CD4+ count >50 cells/µL;
   a. Currently treated with or will need to initiate antiretroviral therapy (ART) which is not compatible with the allowed ARTs and is not considered an appropriate candidate for switching to a regimen of ARVs which is allowed. Examples of allowed treatment include but are not limited to the following. If there are any questions, discuss with the Sponsor Medical Monitor for confirmation of appropriate ARV regimen.
      i. Nevirapine based regimen consisting of nevirapine in combination with any NRTIs;
      ii. Lopinavir/ritonavir (Aluvia™) based regimen consisting of lopinavir/ritonavir (Aluvia™) in combination with any NRTIs;
      iii. The combination of tenofovir/lamivudine/abacavir should be considered in patients with normal renal function to address myelosuppression cross toxicity of zidovudine and linezolid;
      iv. An alternate regimen that may be considered if the above are not appropriate is a triple nucleosidase reverse transcriptase inhibitors (NRTI) based regimen consisting of zidovudine, lamivudine and abacavir may be used with caution. Regimens including zidovudine should be used with special caution as zivovudine and linezolid may both cause peripheral nerve toxicity;
      v. Raltegravir in combination with nucleoside reverse transcriptase inhibitors (NRTIs).
   b. Cannot ensure a 2 week interval between commencing IMP and the start of ART, if not already on ARTs.
8. Having participated in other clinical studies with dosing of investigational agents within 8 weeks prior to trial start or currently enrolled in an investigational study that includes treatment with medicinal agents. Subjects who are participating in observational studies or who are in a follow up period of a trial that included drug therapy may be considered for inclusion.
9. Significant cardiac arrhythmia requiring medication.
10. Subjects with the following at Screening:
    a. QTcF interval on ECG >500 msec. Subjects with QTcF > 450 must be discussed with the sponsor medical monitor before enrolment.
    b. History of additional risk factors for Torsade de Pointes, (e.g., heart failure, hypokalemia, family history of Long QT Syndrome);
    c. Clinically significant ventricular arrhythmias;
    d. Subjects with other cardiac abnormalities that may place them at risk of arrhythmias must be discussed with the sponsor medical monitor before enrolment. Such abnormalities include: Evidence of ventricular pre-excitation (e.g., Wolff Parkinson White syndrome); Electrocardiographic evidence of complete or clinically significant incomplete left bundle branch block or right bundle branch block; Evidence of second or third degree heart block; Intraventricular conduction delay with QRS duration more than 120 msec.
11. Females who have a positive pregnancy test at Screening or already known to be pregnant, breast-feeding, or planning to conceive a child during the study or within 6 months of cessation of treatment. Males planning to conceive a child during the study or within 6 months of cessation of treatment.
12. A peripheral neuropathy of Grade 3 or 4, according to DMID (Appendix 2). Or, subjects with a Grade 1 or 2 neuropathy which is likely to progress/worsen over the course of the study, in the opinion of the Investigator.

Specific Treatments

13. Concomitant use of Monoamine Oxidase Inhibitors (MAOIs) or prior use within 2 weeks of treatment assignment.
14. Concomitant use of serotonergic antidepressants or prior use within 3 days of treatment assignment if Investigator foresees potential risks for serotonin syndrome when combined with linezolid.
15. Concomitant use of any drug known to prolong QTc interval (including, but not limited to, amiodarone, bepridil, chloroquine, chlorpromazine, cisapride, cyclobenzaprine, clarithromycin, disopyramide, dofetilide, domperidone, droperidol, erythromycin, fluoroquinolones, halofantrine, haloperidol, ibutilide, levomethadyl, mesoridazine, methadone, pentamidine, pimozide, procainamide, quinidine, sotalol, sparfloxacin, thioridazine).
16. Concomitant use of any drug known to induce myelosuppression.
17. Use of any drugs or substances within 30 days prior to dosing known to be strong inhibitors or inducers of cytochrome P450 enzymes(including but not limited to quinidine, tyramine, ketoconazole, fluconazole, testosterone, quinine, gestodene, metyrapone, phenelzine, doxorubicin, troleandomycin, cyclobenzaprine, erythromycin, cocaine, furafylline, cimetidine, dextromethorphan). Exceptions may be made for subjects that have received 3 days or less of one of these drugs or substances, if there has been a wash-out period before administration of IMP equivalent to at least 5 half-lives of that drug or substance.
18. Subjects may have previously been treated for DS/MDR-TB (with specific excepetions for bedaquiline and/or linezolid as noted below) provided that treatment is/was discontinued at least 3 days prior to treatment assignment.
19. Subjects should not receive more than 2 weeks of bedaquiline or linezolid prior to enrolment/first dose of IMP.

Based on Laboratory Abnormalities

20. Subjects with the following toxicities at Screening (labs may be repeated) as defined by the enhanced Division of Microbiology and Infectious Disease (DMID) adult toxicity table (November 2007):
    a. serum potassium less than the lower limit of normal for the laboratory;
    b. Hemoglobin level grade 2 or greater (< 8.0 g/dL);
    c. Platelets grade 2 or greater(<75,000/mm^3);
    d. Absolute neutrophil count (ANC) < 1000/ mm^3;
    e. Aspartate aminotransferase (AST)
        - Grade 3 or greater (> 3.0 x ULN) to be excluded;
        - Greater than ULN must be discussed with and approved by the sponsor Medical Monitor
    f. Alanine aminotransferase
        - Grade 3 or greater (> 3.0 x ULN) to be excluded
        - greater than ULN must be discussed with and approved by the sponsor medical monitor ;
    g. Total bilirubin:
        - Grade 3 or greater (≥2.0 x ULN), or if ≥1.5 up to 2.0 x ULN when accompanied by an increase in other liver function test (ALT, AST, Alk Phos or GGT);
        - 1-1.5 x ULN must be discussed with and approved by the sponsor Medical Monitor
    h. Direct bilirubin:
        - Greater than ULN to be excluded
    i. Serum creatinine level greater than 2 times upper limit of normal

j. Albumin <32 g/L

4.4. Treatment Plan: Schedule of Assessments

The trial consists of three periods, as follows:

- Screening (Up to 9 days Prior to Treatment);
- Treatment Period (Day 1 to Week 26 OR Day 1 to Week 39);
- Follow-Up Period (1 month to 24 months post Treatment End).

Refer to:

- Study Flow Chart (Section 1.2) for the overview of the timing of all procedures and laboratory samples to be done at each visit.
- Trial Procedures (Section 6) for details regarding specific procedures or laboratory tests.

Visit Window:

- Week 1 through Week 16: ± 3 days
- Weeks 20 through End of Treatment (Week 26 or 39): ± 7 days
- Post-Treatment Follow-Up Visits (1-3 months): ± 2 weeks

> **Note:** Subjects on 6 months of treatment should complete a full course of treatment (i.e. 26 weeks of prescribed doses) within 8 months of treatment assignment (a total halt of up to 60 days if on 6 months) while subjects on 9 months of treatment should complete a full course of treatment (i.e. 39 weeks of prescribed doses) within 12 months of treatment assignment (a total halt of 90 days if on 9 months of treatment).

4.4.1. Screening (Up to 9 Days Prior to Treatment)

4.4.1.1. Screening

The screening visit may occur over a number of days up to 9 days prior to treatment assignment, (i.e. all screening procedures do not have to be performed on the same day). If a subject fails screening, a full re-screen (all screening procedures must be repeated) may occur at a later date.

The following information will be collected and procedures performed:

- Written Informed Consent (Main study; HIV testing if applicable);
- Demographic Data;
- Medical and Treatment History;
- Eligibility Assessment;
- Karnofsky Score;
- HIV test and CD4 count:
    - If HIV status is a confirmed known positive, repeated test is not needed provided documentation is available. If HIV status is unknown or suspected negative, HIV test should be requested. If an ELISA and/or Western Blot based HIV test was performed within 1 month prior to trial start, it should not be repeated as long as documentation of testing method and negative results can be provided.
    - Subjects may be on current antiretroviral therapy (ART) or commence ART once on the study provided there is at least a 2 week interval between commencing IMP and the start of ART;
- Chest X-Ray;

- Serum or Urine Pregnancy Test, (women of child bearing potential only, whether they are sexually active or not);
- TB Symptoms Profile;
- Patient Reported Health Status;
- Ophthalmology- Slit Lamp Examination;
- Ophthalmic Examination (Ophthalmologic Medical History, Visual Acuity, and Color Assessment);
- Single 12-lead ECG (the ECG should be done before vital signs and any lab assessments);
- Vital Signs, including weight (should be done prior to any lab assessments);
- Full Physical Examination including height;
- Laboratory Safety Assessments;
- Coached Spot Sputum Sample collection;
- Concomitant Medication(s)/Other Treatment(s);
- Adverse Events;
- Peripheral Neuropathy Assessment

4.4.2. Treatment Period (Day 1 to Week 26 or Week 39)

4.4.2.1. Day 1

The following information will be collected and procedures performed pre-dosing:

- Eligibility Assessment;
- Serum or Urine Pregnancy Test, (women of child bearing potential only, whether they are sexually active or not);
- Vital Signs, including weight (should be done before any labs);
- Single 12-lead ECG (the ECG should be done before any vital signs and any labs);
- Full Physical Examination;
- Laboratory Safety Assessments;
- Treatment Assignment;
- Early Morning Sputum Collection;
- Coached Spot Sputum Sample Collection;
- Concomitant Medication(s)/Other Treatment(s);
- Adverse Events;
- Investigational Medicinal Product (IMP) Administration.

4.4.2.2. Week 1

The following information will be collected and procedures performed pre-dosing:

- Vital Signs, including weight (should be done before any labs);
- Single 12-lead ECG (the ECG should be done before any vital signs and any labs);
- Limited Physical Examination;
- Laboratory Safety Assessments;
- Early Morning Sputum Collection;
- Coached Spot Sputum Sample Collection;
- Concomitant Medication(s)/Other Treatment(s);
- Adverse Events;
- Investigational Medicinal Product (IMP) Administration.

4.4.2.3. Week 2

The following information will be collected and procedures performed pre-dosing:

- Vital Signs, including weight (should be done before any labs);
- Limited Physical Examination;
- Laboratory Safety Assessments;
- Pre-dose Pharmacokinetic Sampling (All Subjects);
- Early Morning Sputum Collection;
- Coached Spot Sputum Sample Collection;
- Concomitant Medication(s)/Other Treatment(s);
- Adverse Events;
- Investigational Medicinal Product (IMP) Administration.

4.4.2.4. Weeks 3, 5, 7, 9, 10, 11, 13, 14 and 15

- Complete Blood Count/Full Blood Count (performed pre-dosing);
- Concomitant Medication(s)/Other Treatment(s);
- Adverse Events.
- Study Medication/Compliance.

4.4.2.5. Week 4

The following information will be collected and procedures performed pre-dosing:

- Vital Signs, including weight (should be done before any labs);
- Single 12-lead ECG (the ECG should be done before any vital signs or labs);
- Limited Physical Examination;
- Laboratory Safety Assessments;
- Early Morning Sputum Collection;
- Coached Spot Sputum Sample Collection;
- Concomitant Medication(s)/Other Treatment(s);
- Ophthalmic Examination (Visual Acuity and Color Assessment);
- Adverse Events;
- Peripheral Neuropathy Assessment;
- Investigational Medicinal Product (IMP) Administration

4.4.2.6. Week 6

The following information will be collected and procedures performed pre-dosing:

- Vital Signs, including weight (should be done before any labs);
- Limited Physical Examination;
- Laboratory Safety Assessments;
- Early Morning Sputum Collection;
- Coached Spot Sputum Sample Collection;
- Concomitant Medication(s)/Other Treatment(s);
- Adverse Events;
- Investigational Medicinal Product (IMP) Administration.

4.4.2.7. Week 8

The following information will be collected and procedures performed pre-dosing:

- Serum or Urine Pregnancy Test, (women of child bearing potential only, whether they are sexually active or not);

- TB Symptoms Profile/Patient Reported Health Status;
- Vital Signs, including weight (should be done before any labs);
- Single 12-lead ECG (the ECG should be done before any vital signs or labs);
- Limited Physical Examination;
- Laboratory Safety Assessments;
- Early Morning Sputum Collection;
- Coached Spot Sputum Sample Collection;
- Concomitant Medication(s)/Other Treatment(s);
- Adverse Events;
- Peripheral Neuropathy Assessment.
- Ophthalmic Examination (Visual Acuity and Color Assessment);
- Pre-dose Pharmacokinetic Sampling (All Subjects);
- Investigational Medicinal Product (IMP) Administration.

4.4.2.8. Week 12

The following information will be collected and procedures performed pre-dosing:

- Vital Signs, including weight (should be done before any labs);
- Limited Physical Examination;
- Laboratory Safety Assessments
- Ophthalmic Examination (Visual Acuity and Color Assessment);
- Early Morning Sputum Collection;
- Coached Spot Sputum Sample Collection;
- Concomitant Medication(s)/Other Treatment(s);
- Adverse Events;
- Peripheral Neuropathy Assessment;
- Investigational Medicinal Product (IMP) Administration.

4.4.2.9 Week 16 (Week 30 when applicable)

The following information will be collected and procedures performed pre-dosing:

- Vital Signs, including weight (should be done before any labs);
- Single 12-lead ECG (the ECG should be done before any vital signs or labs);
- Limited Physical Examination;
- Laboratory Safety Assessments;
- Ophthalmic Examination (Visual Acuity and Color Assessment)
- Early Morning Sputum Collection;
- Coached Spot Sputum Sample Collection;
- Concomitant Medication(s)/Other Treatment(s);
- Adverse Events;
- Peripheral Neuropathy Assessment;
- Investigational Medicinal Product (IMP) Administration;
- Pre-Dose Pharmacokinetic Sampling (all subjects, this should be done prior to dosing with study medication)
- Intensive PK Sub-Study (20 evaluable across all participating sites) at pre-dose, 0.5, 1, 2, 4, 8, 12. 12.5, 13, 14, 16, 20 and 24 hours after dosing.

4.4.2.10 Week 20 (Week 34 when applicable)

The following information will be collected and procedures performed pre-dosing:

- Vital Signs, including weight (should be done before labs);
- Limited Physical Examination;
- Laboratory Safety Assessments;
- Ophthalmic Examination (Visual Acuity and Color Assessment);
- Early Morning Sputum Collection;
- Coached Spot Sputum Sample Collection;
- Concomitant Medication(s)/Other Treatment(s);
- Adverse Events;
- Peripheral Neuropathy Assessment;
- Investigational Medicinal Product (IMP) Administration.

4.4.2.11 Week 26 (Week 39 when applicable)

The following information will be collected and procedures performed when subject completes *end of treatment visit*:

- Serum or Urine Pregnancy Test, (women of child bearing potential only, whether they are sexually active or not);
- TB Symptoms Profile/Patient Reported Health Status;
- Vital Signs, including weight (should be done before any labs);
- Single 12-lead ECG (the ECG should be done before any vital signs or labs);
- Full Physical Examination;
- Laboratory Safety Assessments;
- Early Morning Sputum Collection;
- Coached Spot Sputum Sample Collection;
- Ophthalmology Slit Lamp Examination;
- Ophthalmic Examination (Visual Acuity and Color Assessment);
- Concomitant Medication(s)/Other Treatment(s);
- Adverse Events;
- Peripheral Neuropathy Assessment;
- Investigational Medicinal Product (IMP) Administration.

When subject is scheduled to receive 9 months of treatment, the following assessments should be done at the week 26 visit:

- Vital Signs, including weight (should be done before any labs);
- Single 12-lead ECG (the ECG should be done before any vital signs or labs);
- Limited Physical Examination;
- Laboratory Safety Assessments;
- Early Morning Sputum Collection;
- Coached Spot Sputum Sample Collection;
- Ophthalmic Examination (Visual Acuity and Color Assessment);
- Concomitant Medication(s)/Other Treatment(s);
- Adverse Events;
- Peripheral Neuropathy Assessment;
- Investigational Medicinal Product (IMP) Administration.

4.4.2.12 Early Withdrawal

In case of Early Withdrawal during the treatment period of the study (prior to completing 26 or 39 weeks of treatment as applicable), all efforts shall be made to complete the Early Withdrawal assessments. At the Early Withdrawal visit, the following information will be collected and procedures performed:

- Serum or Urine Pregnancy Test (for women of child bearing potential only, whether they are sexually active or not);
- TB Symptoms Profile/Patient Reported Health Status;
- Vital Signs, including weight (should be done before any labs);
- Single 12-lead ECG (the ECG should be done before any vital signs and labs);
- Full Physical Examination;
- Laboratory Safety Assessments;
- Early Morning Sputum Collection;
- Coached Spot Sputum Sample Collection;
- Ophthalmology Slit Lamp Examination (if received ≥ 12 weeks of study treatment);
- Ophthalmic Examination (Visual Acuity and Color Assessment)
- Concomitant Medication(s)/Other Treatment(s);
- Adverse Events;
- Peripheral Neuropathy Assessment.

Follow-Up required for Early Withdrawals based on Treatment Duration

Treatment Duration at EWD visit	Ophthalmology Examination at EWD	Ophthalmology Examination Visit 3 months after EWD Visit	Month 6	Month 24
≤ 14 days	Not required	Not required	Not Required	Not Required
15 days to ≤ 12 weeks	Not required	Required	Required	Required
> 12 weeks	Required	Required	Required, if not already performed.	Required

Upon Early Withdrawal of IMP, All Subjects will be referred to a unit specializing in treatment of XDR-TB.

4.4.3 Follow-Up Period

4.4.3.1 1 Month Post-Treatment

- Early Morning Sputum Collection;
- Coached Spot Sputum Sample Collection;
- Concomitant Medication(s)/Other Treatment(s);
- Adverse Events.

4.4.3.2 2 Months Post-Treatment

- Early Morning Sputum Collection;
- Coached Spot Sputum Sample Collection;
- Concomitant Medication(s)/Other Treatment(s);
- Adverse Events.

4.4.3.3 3 Months Post-Treatment or Withdrawal

The following information will be collected and procedures performed:

- Vital Signs, including weight;
- Ophthalmology Slit Lamp Examination*;
- Ophthalmic Examination (Visual Acuity and Color Assessment);
- Limited Physical Examination;
- Early Morning Sputum Collection;
- Coached Spot Sputum Sample Collection;
- Concomitant Medication(s)/Other Treatment(s);
- Peripheral Neuropathy Assessment;
- Adverse Events.

* - Ophthalmology Slit Lamp Exam is required only for subjects who complete 15 days or more of treatment.

4.4.3.4 Months 6, and 24 Post-Treatment

The following information will be collected and procedures performed:

- TB Symptoms Profile/ Patient Reported Health Status (only at Months 6 and 24)
- Vital Signs, including weight;
- Ophthalmic Examination (Visual Acuity and Color Assessment)
- Limited Physical Examination;
- Early Morning Sputum Collection;
- Coached Spot Sputum Sample Collection;
- Concomitant Medication(s)/Other Treatment(s);
- Peripheral Neuropathy Assessment;
- Adverse Events.

For any Subjects who withdraw early (during the treatment period after more than 14 days treatment or follow-up), the Month 6 follow-up will be a full study visit if not already prerformed. If Month 6 is already performed, it will serve along with Month 24 visit to collect Adverse Event (AEs), Concomitant Medidcation and Serious Adverse Event (SAE) information,including verification of survival and patient reported TB outcome information only and may be telephonic, a home or a site visit.

4.4.3.5 Months 9, 15, 18 and 21 Post-Treatment

- Vital Signs, including weight;
- Limited Physical Examination;
- Early Morning Sputum Collection;
- Coached Spot Sputum Sample Collection;
- Concomitant Medication(s)/Other Treatment(s);
- Adverse Events.

4.4.3.6 Unscheduled Visits

Any visit which is conducted in addition to those required by the Trial Flow Chart should be considered unscheduled regardless of the reason for the visit. The assessments which are undertaken as part of an unscheduled visit should be as clinically indicated.

If the duration of treatment is extended due to dose interruptions (e.g., takes participant 8 months to complete 6 months of therapy or 12 months to complete 9 months of therapy), Unscheduled visits should be added every 4 weeks.

Visits to include:

- Vital Signs, including weight (should be done before labs);
- Limited Physical Examination;
- Laboratory Safety Assessments;
- Ophthalmology Examination (Visual Acuity and Color Assessment);
- Early Morning Sputum Collection;
- Coached Spot Sputum Sample Collection;
- Concomitant Medication(s)/Other Treatment(s);
- Adverse Events;
- Peripheral Neuropathy Assessment;
- Investigational Medicinal Product (IMP) Administration.

If both spot sputum samples obtained between Month 4 and Month 6, at the End of Treatment (Week 26/39), End of follow-up Period or Early Withdrawal visits are contaminated, the subject should return for an unscheduled visit(s) to give additional samples or to document the Subject is not able to produce sputum.

In order to be able to define a Subject's primary outcome status it may be necessary in certain situations to contact a Subject and request they visit the site in order to collect additional Spot Sputum samples at Unscheduled Visits, as follows:

- <u>To be assessed on sputum culture results from:</u>
  - End of Treatment Period (Week 26/39);
  - End of Follow-up Period (Month 24);
  - Early Withdrawal (if applicable).

- <u>Confirm whether the Subject has:</u>
  - Two sequential negative sputum culture results; or
  - Two sequential positive sputum culture results; or
  - Has been unable to produce sputum after documentation of two negative sputum cultures with no intervening positive and are clinically asymptomatic.

- If they **do not** fall into one of these categories, keep collecting Spot Sputum samples x 2 (one Early Morning and one Spot at the research site under the coaching and observation of the trial staff OR two spot sputum at the research site if Early Morning is not available) at a minimum of 7 days or more apart until they fall into one of the above categories.

If in any of the above scenarios the Investigator is unsure of the outcome, the Investigator must contact the Sponsor Medical Monitor to discuss and agree on how the patient is to be handled.

4.5 Treatment Discontinuation and Subject Withdrawal

Any Subject for whom the Investigator decides to temporarily discontinue their IMP is to contact the Sponsor Medical Monitor and, if/when applicable, can be restarted on IMP as described in section 4.6

A Subject should immediately discontinue treatment and be prematurely withdrawn from the trial (withdrawal of informed consent or lost to follow-up) or treatment phase for the following reasons:

- Withdrawal of informed consent;
- Lost to Follow-Up;
- Investigator considers that for safety reasons (including specific toxicities as described in section 7.3), it is in the best interest of the Subject he/she be withdrawn;
- Pregnancy;

- Regimen halted > 35 days consecutively (providing subject is not smear negative or with trace/scanty results, judged to be clinically improving by the investigator) and did not receive at least 4 weeks of linezolid 1200 mg total daily dose since start of treatment;
- Regimen halted cumulatively greater than 60 days cumulatively for Subjects receiving 6 months of treatment and 90 days cumulatively for Subjects receiving 9 months of treatment;
- If subject did not receive at least 4 consecutive weeks of linezolid 1200 mg total daily dose at treatment start and Linezolid is:
    - **halted cumulatively greater than 60 days for Subjects receiving 6 months of treatment or**
    - **halted 90 days cumulatively for Subjects receiving 9 months of treatment or**
    - **interrupted for greater than 35 days consecutively.**
- At the specific request of the Sponsor or termination of the study by the Sponsor;
- Subject who, in the opinion of the Investigator or Sponsor, fails to comply with the Protocol, including non-compliance to IMP.

If at any time the investigator is unsure whether or not to withdraw the Subject, the Investigator is to contact the Sponsor Medical Monitor and discuss and agree on how the patient is to be handled. Subjects who withdraw from the trial after having received IMP will not be replaced.

Upon discontinuation of IMP, Subjects will be referred to a unit specializing in the treatment of XDR.

Subjects who withdraw early should have an early withdrawal visit and additional follow-up visits according to timing of withdrawal as outlined in section 4.4.3.4

Early Withdrawal due to TB

Ultimately it is the investigator's decision whether a Subject requires Early Withdrawal from the trial due to a concern that the Subject has symptomatic worsening TB and/or bacteriological failure/relapse.

Early Withdrawal is usually not indicated by a single positive culture. Should a Subject have a single positive culture result after being negative, the investigator is to evaluate whether the Subject has signs and symptoms suggestive of active inadequately treated TB and whether it is in the Subjects best interest that he/she be withdrawn. Prior to Early Withdrawal of a Subject due to TB, the investigator must discuss the Subject with the sponsor medical monitor, unless the investigator cannot contact the sponsor medical monitor and considers that Early withdrawal must occur immediately due to immediate safety concerns with respect to the Subject.

If the investigator decides to withdraw a Subject due to TB, additional sputum samples may need to be collected in order to ensure the Subject's outcome status may be determined (section 4.4.3.4).

All Early Withdrawal Subjects who are confirmed sputum positive (two sequential sputum positive cultures) and/or have symptomatic TB will require further TB treatment. These Subjects will be referred to a unit that specializes in treatment of XDR-TB.

4.6 Temporary Dose Interruptions and Modifications

All dose interruption and modifications should be discussed with the Sponsor Medical Monitor prior to implementation.

For Subjects experiencing suspected drug related toxicities due to linezolid, the daily dose of linezolid may be reduced or may be temporarily halted for up to 35 consecutive days. Generally, if temporarily halted, it should be re-instituted at a lower dose. Generally a step down in dose could proceed from 1200 mg QD to 600 mg and then to 300 mg daily. Linezolid dose may be re-started at the same dose at Investigator discretion. If subjects have toxicity issues with linezolid that would prohibit further treatment with that drug, they can remain on the bedaquiline and pretomanid study IMP if they received the initial 1200 mg QD dose of linezolid for at least the

first 4 weeks of treatment and they are smear negative or with trace results and judged to be clinically improving by the Investigator.

For Subjects experiencing suspected drug related toxicities due to other drugs in the regimen (B-Pa), the full regimen may be halted for up to 35 consecutive days.

Subjects on 6 months of treatment should complete a full course of treatment (i.e. 26 weeks of prescribed doses) within 8 months of treatment assignment (a total halt of up to 60 days if on 6 months) while subjects on 9 months of treatment should complete a full course of (i.e. 39 weeks of prescribed doses) treatment within 12 months of treatment assignment (a total halt of 90 days if on 9 months of treatment). For Subjects who completed the first 4 consecutive weeks of treatment on the 1200 mg linezolid total dose and later in treatment only halted linezolid, treatment can be considered complete at 6 months, even if there were multiple interruptions and rechallenges of just linezolid while the participant remained on pretomanid and bedaquiline.

When total of missed dosing days and/or pauses is greater than 7 days, the same number of missed dosing days should be dispensed/treatment extended to make up for the total missed doses.

At no time should the Subject be treated with a single agent.

4.7 Stopping Rules

There are no trial specific stopping rules.

The trial or parts of the trial can be stopped by the Sponsor on advice from the Data Safety and Monitoring Committee (DSMC) after their review of applicable trial data. In addition, the Sponsor has the right to stop the trial or a specific Investigational Site at any time, although this should only occur after consultation between involved parties. Should this occur, the local and central Ethics Committee/Institutional review Board (EC/IRB) and Regulatory Authorities will be informed. Should the Trial/Investigational Site be closed prematurely, all trial materials (except documentation that has to remain stored at the Investigational Site) will be returned to the Sponsor or vendor. The Investigator will retain all other documents until notification given by the Sponsor for destruction. Subjects currently on treatment will receive an appropriate regimen and all Subjects will be referred to a unit specializing in the treatment of XDR-TB.

4.8 Subject Progress Definitions

All efforts should be made to contact subjects that do not attend scheduled trial visits. The investigator should attempt to follow up subjects that miss scheduled trial visits unless the subject has withdrawn consent.

If a subject fails to attend a scheduled trial visit, the site will attempt to contact the subject as soon as possible by phone (if applicable) and, if necessary, a home visit will be made, to encourage attendance at the earliest opportunity.

All Subjects will be categorized with two of the following definitions and this should be clearly documented on the eCRF.

4.8.1 Enrolment

Screening Failure

Subjects from whom informed consent is obtained and is documented in writing (that is, subject signs an informed consent form), but are not assigned treatment.

Enrolled

Subjects from whom informed consent is obtained and is documented in writing (that is, subject signs an informed consent form), and who are assigned treatment.

4.8.2 Completed Trial

Subjects who are assigned treatment and complete Treatment and Follow-Up.

4.8.3 Withdrawn

During Treatment- Subjects who are assigned treatment and withdraw/are withdrawn from the trial prior to completion of treatment visits.

During Follow-up- Subjects who are assigned and complete treatment, however withdraw/are withdrawn from the trial prior to completion of their follow-up visits.

4.9 Restrictions

4.9.1 Prior and Concomitant Medications and Other Treatments

Concomitant medications should be kept to a minimum during the trial. However, if concomitant medications are considered to be necessary for the Subject's welfare and are unlikely to interfere with the IMP, they may be given at the discretion of the investigator. For any concomitant medications given as a treatment for a new condition or a worsening of an existing condition occurring after signing of the informed consent form, the condition must be documented on the Adverse Event pages of the electronic Case Report Form (eCRF).

The prescribing information for all concomitant medication should be consulted and reviewed carefully. The determinations listed in the respective contraindicated, warning, and precaution sections must be respected in order to prevent any potentially serious and/or life-threatening drug interactions.

The following concomitant medications are prohibited during the treatment period to avoid possible drug interactions with the IMP:

- Medicinal products used to treat pulmonary TB: including but not limited to gatifloxacin, amikacin, cycloserine, rifabutin, kanamycin, para-aminosalicylic acid, rifapentine, thioacetazone, capreomycin, quinolones, thioamides, and metronidazole.
- Concomitant use of Monoamine Oxidase Inhibitors (MAOIs). (e.g. phenelzine, isocarboxazid)
- Concomitant use of any drug known to prolong QTc interval (including but not limited to amiodarone, bepridil, chloroquine, chlorpromazine, cisapride, cyclobenzaprine, clarithromycin, disopyramide dofetilide, domperidone, droperidol, erythromycin, halofantrine, haloperidol, ibutilide, levomethadyl, mesoridazine, methadone, pentamidine, pimozide, procainamide, quinidine, sotalol, sparfloxacin, thioridazine).
  - Treatment with fluoroquinolones (as they are known prolong QTc), are strongly discouraged in the trial. They should only be used to treat intercurrent non-TB infections and if the benefit of treatment outweighs the risk of prolonged QTc.
- Concomitant use of any drug known to induce myelosuppression.
- The systemic use of CYP3A4 inhibitors (e.g., azole antifungals: ketoconazole, voriconazole, itraconazole, fluconazole; ketolids such as telithromycin; and macrolide antibiotics other than azithromycin) for more than 3 consecutive days;
- The systemic use of CYP3A4 inducers (e.g., phenytoin, carbamazepine, phenobarbital, St. John's wort, rifamycins and systemic dexamethasone.

Concomitant use of serotonergic antidepressants should be avoided if possible as subjects on these agents and linezolid are at risk for serotonin syndrome.

Caution should be used in treating diabetic patients receiving insulin or oral hypoglycemic agents as cases have been reported of hypoglycemic reactions when patients on these agents have been treated with linezolid.

Any drug known to be hepatotoxic should be avoided as much as possible during screening and throughout the treatment period (including but not limited to acetaminophen/paracetamol, acetazolamide, allopurinol, amiodarone, amitriptyline, amoxicillin, amprenavir, atorvastatin, augmentin/co-amoxiclav, azathioprine,

baclofen, bumetanide, captopril, carbamazepine, celecoxib, chlorpromazine, chlorpromazine, clindamycin, clopidogrel, contraceptive pill, co-trimoxazole, darunavir, delavirdine, diclofenac, doxycycline, enalapril, fluconazole, fluoxetine, fosamprenavir, furosemide, gliclazide, glimeperide, glipizide, ibuprofen, irbesartan, ketoconazole, lisinopril, loperamide, losartan, methotrexate, metolazone, mirtazepine, nitrofurantoin, omeprazole, other non-steroidal anti-inflammatory drugs, paroxetine, phenobarbital, phenothiazines, phenytoin, pravastatin, probenecid, prochlorperazine, risperidone, rosuvastatin, sertraline, simeprevir, simvastatin, sodium valproate, sotalol, sulfasalazine, sumatriptan, tamsulosin, terbinafine, tetracycline, theophyllin/uniphyllin, tipranavir, tolazamide, tolbutamide, topiramate, trazodone, tricyclic antidepressants, trimethoprim, verapamil).

4.9.1.1 Recommendations for Concomitant use of Anti-Malarials

The following treatments for malaria are recommended for concomitant use with the IMP, should it be necessary:

- Proguanil/atovaquone or
- Artesunate plus sulfadoxine-pyrimethamine

These recommendations are based on the potential for QT prolongation by bedaquiline and many anti-malarials. Due to the extended half-life of bedaquiline commencing anti-malarial treatment containing drugs that could prolong the QT interval, shortly after discontinuing bedaquiline, is not recommended.

4.9.1.2 Antiretroviral Therapy

Patients taking bedaquiline should avoid efavirenz due to drug-drug interactions with bedaquiline, and thus Examples of allowed treatment include but are not limited to the following. If there are any questions, discuss with the Sponsor Medical Monitor for confirmation of appropriate ARV regimen:

- Nevirapine based regimen consisting of nevirapine in combination with any NRTIs;
- Lopinavir/ritonavir (Aluvia™) based regimen consisting of lopinavir/ritonavir (Aluvia™) in combination with any NRTIs;
- The combination of tenofovir/lamivudine/abacavir should be considered in patients with normal renal function to address myelosuppression cross toxicity of zidovudine and linezolid;
- An alternate regimen that may be considered if the above are not appropriate is a triple nucleoside reverse transcriptase inhibitor (NRTI) based regimen consisting of zidovudine, lamivudine, and abacavir may be used with caution. Regimens including zidovudine and linezolid may both cause peripheral nerve toxicity;
- Raltegravir in combination with nucleoside reverse transcriptase inhibitors (NRTIs).

Subjects who are commencing ART may be entered onto the study provided there is at least a 2 week interval between commencing IMP and the start of ART.

4.9.1.3 Other Restrictions

Large quantities of foods or beverages with high tyramine content should be avoided while taking linezolid. Quantities of tyramine consumed should be less than 100mg per meal. Foods high in tyramine content include those that may have undergone protein changes by aging, fermentation, pickling, or smoking to improve flavour, such as aged cheeses (0 to 15 mg tyramine per ounce); fermented or air-dried meats (0.1 to 8 mg tyramine per ounce); sauerkraut (8 mg tyramine per 8 ounces); soy sauce (5mg tyramine per 1 teaspoon). The tyramine content of any protein-rich food may be increased if stored for long periods or improperly refrigerated.

Alcohol should be avoided while on IMP, especially in patients with impaired hepatic function.

5 INVESTIGATIONAL MEDICINAL PRODUCT

5.1 Trial Treatments

Subjects will receive oral dosing as described below.

Bedaquiline Days 1-14: 400mg once daily (4 x bedaquiline 100 mg tablets),

Bedaquiline Weeks 3-26/39*: 200mg three times per week (2 x bedaquiline 100 mg tablets); **plus**

Linezolid 1200mg once daily day 1 through week 26 or 39* (2 x scored linezolid 600 mg tablets); **plus**

Pretomanid 200mg once daily Day 1 through week 26 or 39* (1 x pretomanid 200 mg tablet)**.**

Subjects will receive a minimum of 6 months of treatment, (with 24 months of Follow Up). If a Subject is culture positive or revert to being culture positive between their Month 4 visits and their clinical condition suggests they may have ongoing TB infection, they may have treatment extended to 9 months (with 24 months of Follow Up), or be withdrawn from the study. If culture result between month 4 and 6 are contaminated, missing or considered an isolated positive without clinical significance, please use available culture results to make this decision and consult with the TB Alliance Medical Monitor. All decisions regarding treatment extension should be discussed with the TB Alliance Medical Monitor before implementation

5.2 Method of Assigning Subjects to Study Treatment

Eligible Subjects who have given written, informed consent will be enrolled onto the trial during Screening and will be identified by a study generated Subject identification code for anonymity (Subject number).

Once the screening results are available and subject is eligible to participate, the site will request their pharmacist/registered dispenser to assign an IMP treatment number to the Subject. The site pharmacist/registered dispenser will assign the next available applicable treatment number, in a sequential basis starting from the lowest unused treatment number.

The process of assigning a treatment number will be fully documented.

5.3 IMP Administration

The Subject should be instructed to:

- Take IMP orally once daily with food for 26 weeks, preferably at the same time every day, with a glass of water (approximately 240ml);
- Subjects should take IMP with a meal (generally allow the Subjects a window of 30 minutes before to 30 minutes after a meal);
- When Subjects are hospitalized or return for clinic visits, they will be dosed on site.

5.4 Subject Compliance

During site clinic visits or hospitalisation, the IMP will be administered by the Investigator/designated site personnel. During the study, sites will be responsible for ensuring Subjects are taking the IMP correctly and are fully trained on how IMP is to be taken. When possible, Subjects will be checked for IMP compliance by the Investigators or trial personnel/National TB Treatment Program personnel via the hand-and-mouth procedure (both the hand and mouth of the Subject will be checked to ensure that the Subject has swallowed the IMP).

5.5 Blinding and Procedures for Breaking the Blind

This is an open label study. There is no need for blinding or procedures to break the blind.

5.6 IMP Packaging and Labelling

The complete formulations of the bedaquiline and pretomanid are found in the applicable Investigator Brochures (4,5,15). The complete formulations of linezolid are found in the applicable Package Inserts (18,31,34).

5.6.1 Packaging

IMP will be supplied as:

bedaquiline 100mg Tablets;
Scored linezolid 600mg Tablets;
pretomanid 200mg Tablets;

Subjects will receive oral dosing as described below.

Bedaquiline Days 1-14: 400mg once daily (4 x bedaquiline 100 mg tablets),

Bedaquiline Weeks 3-26/39*: 200mg three times per week (2 x bedaquiline 100 mg tablets); **plus**

Linezolid 1200mg once daily Day 1 through week 26 or 39* (2 x scored linezolid 600 mg tablets); **plus**

pretomanid 200mg once daily Day 1 through week 26 or 39* (1 x pretomanid 200 mg tablet).

Subjects will receive a minimum of 6 months of treatment. If a Subject is culture positive or revert to being culture positive between Month 4 and Month 6 visits and their clinical condition suggests they may have ongoing TB infection, they may have treatment extended to 9 months or be withdrawn from the study.

Labelling

The test product will be packaged in blister cards with bulk card supplies available for the B-Pa weeks 1-2, B-Pa weeks 3-End of Treatment and Linezolid. The outer packaging of each bulk pack will be labelled with, at a minimum, the following information:

Name and address of the Sponsor
Telephone number of the investigational site
Name of medication, dosage, quantity and method of administration
Reference/Lot Number
Protocol number, visit numbers and space for completion of name of Investigator and site number
The statement "For Clinical Trial Use Only"
Storage conditions
Expiry date
The statement "Keep out of reach of children"

The inner packaging on each weekly treatment card will be labelled with, at a minimum, the following information:

Name and address of the Sponsor
Telephone number of the investigational site
Name of medication, dosage, quantity and method of administration
Reference/Lot Number
Protocol number and space for completion of name of Investigator, site and visit number
Directions for use
Subject Number and Initials
The statement "For Clinical Trial Use Only"
Storage conditions
Expiry date
The statement "Keep out of reach of children"

5.6.2 Storage

All study medication will be kept securely stored by the site pharmacist/registered dispenser in a secured area with limited access to designated site personnel only.

Test product containing treatment arms will be stored in the supplied containers (thereby protected from light and moisture), between 15 to 25 degrees Celsius.

5.7 Dispensing and Accountability

The site pharmacist/ delegated dispenser will be responsible for dispensing the IMP. Accurate accountability records will be kept by the site to assure that the IMP will not be dispensed to any person who is not a Subject under the terms and conditions set forth in this protocol (i.e. delivery to site, inventory at site, use by Subject, destruction, etc.) The Investigator/designee will immediately inform the Sponsor of any quality issues arising with respect to the trial medication. The Sponsor will take whatever action is required should such a situation arise.

The Investigator undertakes to use the trial medication only as indicated in this protocol.

5.8 Returns and Destruction

Upon completion or termination of the trial, all unused and/or partially used IMPs must either be returned to Sponsor (or designated vendor) who will arrange for destruction or destroyed at site as agreed by sponsor after final accountability has been confirmed. If no supplies remain, this fact will be indicated in the drug accountability section of the final report.

6 TRIAL VARIABLES AND PROCEDURES

6.1 Demographic and Background Variables and Procedures

The following demographic and background variables will be collected at the time points described in the trial flow chart:

- Visit Dates.
- Subject Disposition.
- Written Informed Consent (including HIV when applicable).
- Eligibility criteria.
- Demographic data: Date of birth, race and gender.
- Medical and treatment history.
- Screening Coached Spot Sputum Sample:
  - Smear microscopy for acid-fast bacilli.
  - Gene Xpert, Hain Assay MTBDRplus or an alternative molecular or antigen-based test to confirm *M.tb.*
- Serum or Urine pregnancy test: women of child-bearing potential only, whether they are sexually active or not.
- Serology: HIV and CD4 count.
  - Approval for this to be performed will be obtained from Subjects in the written informed consent process. If an HIV test was performed within 1 month prior to trial start, it should not be repeated as long as documentation can be provided (ELISA and/or Western Blot).
  - Prior to HIV testing and on receipt of the results, Subjects will be counselled on HIV by trained counsellors if they have indicated as such on the HIV consent form. If requested by the Subject, HIV counselling provided to the Subject by the study site should be clearly documented in the Subject's medical records/source. Subjects have the right to decline to know or receive their HIV test results. This decision should be clearly documented in the Subject's medical records/source.
- Karnofsky Score (Appendix 4).

- Chest X-Ray: A Chest X-Ray picture will be obtained from the clinic appointed radiology department or from the Subject if it has been taken within the previous 1 year. The Investigator is responsible for review and analysis for Subject inclusion.
- Method of Birth Control: Male and Female Subjects and their partners.
- IMP Details/Actual Dosing

6.2 Efficacy Variables and Procedures

Two sputum Samples are collected, (one Early Morning brought from home or done in the hospital ward and one spot at the research site under the coaching and observation of the trial staff OR two spot sputum at the research site if Early Morning is not available). The Mycobacteriology sampling methodology and requirements will be described in a separate document, the Laboratory Manual, which will be provided prior to the trial start.

The following analyses will be performed:

- Culture result;
- If liquid culture in the MGIT platform is used, TTP in liquid medium.

Using these observed variables the following derived variables will be assessed for evaluation of the efficacy endpoints:

- Bacteriologic failure/relapse;
- Time to Sputum Culture Conversion;
- Number of subjects with Sputum Culture Conversion.

Every effort is to be made to collect sputum samples. However, in general, the inability to produce sputum is treated as being equivalent to having a negative culture (favourable) result. A subject who never achieves culture negative status due to inability to produce sputum, but has completed 6/24 months follow-up and is without clinical or biological evidence of relapse, will be considered to have a favorable outcome.

TB Symptoms Profile:

- The TB Symptoms Profile (Appendix 7) will record subjects' ratings of the severity of common TB symptoms.

Patient Reported Health Status Variables and Procedures:

- The Patient Reported Health Status variables will be collected at the time points described in the trial flow chart.
- Patient Reported Health Status will be collected using the EQ-5D-5L Health Questionnaire (Appendix 5). This descriptive system consists of five health-related quality of life dimensions, each of which will be recorded using five levels of severity.
- Methodology: The Patient Reported Health Status methodology and requirements will be described in a separate document/guideline which will be provided prior to the trial start.

6.3 Safety and Tolerability Variables and Procedures

The following safety and tolerability variables will be collected at the time points described in the trial flow chart and assessed for evaluation of the safety endpoints:

- Laboratory parameters. The Safety Laboratory sampling methodology and requirements will be described in a separate document, the Laboratory Manual, which will be provided prior to the trial start. The following analyses will be performed:
    - Hematology/Complete Blood Count/Full Blood Count (hemoglobin, hematocrit, red blood cell count, white blood cell count with differential, platelet count),

    - At Weeks 3, 5, 7, 9, 10, 11, 13, 14, 15, Complete Blood Count/Full Blood Count including red and white cell counts and indices and platelet count ***only*;** no clinical chemistry or urinalysis at those visits.
  - Clinical Chemistry (albumin, serum urea, creatinine, direct, indirect and total bilirubin, uric acid, total protein, alkaline phosphatase, aspartate aminotransferase (AST), alanine aminotransferase (ALT), gamma-glutamyl transferase (GGT), lactic dehydrogenase (LDH), total amylase, lipase, phosphate, sodium, potassium, calcium (corrected for albumin), chloride, magnesium, random/fasting glucose, bicarbonate/CO_2, creatine phosphokinase (CPK and CK-MB).
  - Urinalysis (pH, specific gravity, protein, glucose, micro-albumin, ketones, bilirubin, creatinine, nitrite, sodium, urobilinogen, blood, leukocytes). Microscopy will be completed as follow up to abnormal urinalysis per discretion of Investigator.
- 12-lead Electrocardiogram (ECG):
  - Investigator Assessment: Normal, Abnormal;
  - Methodology:
    - ECGs should be recorded prior to any lab draws and administration of IMP;
    - Subjects should be lying down (recumbent) for at least 5 minutes prior to each 12-lead ECG evaluation;
    - ECGs are to be recorded for 10 seconds;
    - All ECG to be performed in single;
    - For each Subject, the ECGs should, to every extent possible, be collected at approximately the same time of the day and in the same fed/fast state (e.g. 4 hours after lunch).

- Vital signs:
  - Vital Signs, including weight (should be done before any labs)
  - Systolic and diastolic blood pressure (mmHg) to be measured supine (after 5 minutes of rest) using an appropriately sized cuff, and using the same type of sphygmomanometer, if possible by the same observer, at each relevant visit.
  - Heart rate (bpm).
  - Respiratory rate (breaths per minute)
  - Axillary body temperature (°C).
- Physical Examination:
  - Height is measured at screening only.
  - Full (complete) and Limited (pulmonary, cardiovascular and abdominal) examinations will be performed and any clinically significant findings will be recorded.
  - Weight (kg) (in light clothing and with no shoes).
  - Using the observed variables weight and height, calculated body mass index (BMI) will be derived.

- Ophthalmology Slit Lamp Examination. To be done by an Ophthalmologist trained on AREDS2 assessment. The ophthalmology slit lamp methodology and requirements will be described in a separate document, the Ophthalmology Guideline, which will be provided prior to the trial start. The following analyses will be performed: AREDS2 opacity typing and grading.
- Ophthalmic Examination. The ophthalmic examinations can performed by any trained study staff. The screening exams must be done by the trained study staff AND an Ophthalmologist. Methodology and requirements will be detailed in a separate Ophthalmic Examination Manual.
  - Ophthalmology History (Screening only);
  - Visual Acuity Test – Corrected. Near and Distance Vision;
  - Color Vision Assessment.
- Adverse Events.
- Brief Peripheral Neuropathy Screen (Appendix 6) will record ratings.

- Concomitant Medication/Other Treatments.

6.4 Pharmacokinetic Variables and Procedures

Pharmacokinetics will consist of two separate schedules:

- All Subjects- Pre-dose sampling at weeks 2, 8 and 16 to measure C_{trough} levels of B, B metabolite M2, linezolid and Pa.
- PK Sub-Study Subjects- in addition to the C_{trough} samples, there will be intensive PK sampling at week 16 at pre-dose, 0.5, 1, 2, 4, 8 12, 12.5, 13, 14, 16, 20 and 24 hours after dosing in a sub-group of 20 evaluable Subjects across selected sites.

Pharmacokinetic Analysis:
For the C_{trough} samples, only descriptive statistics will be prepared (average C_{trough}) derived for each analyte.

For the PK Sub-Study samples, the following PK parameters will be estimated from the individual (per Subject) PK plasma concentrations: minimum observed PK plasma concentration (C_{min}), maximum observed PK plasma concentration(C_{max}), time to reach C_{max} obtained without interpolation (T_{max}), area under the PK plasma concentration time (t) curve from zero to the last quantifiable PK plasma concentration prior to the subsequent dose, using the linear trapezoidal rule ($AUC_{(0-t)}$), area under the PK plasma concentration time (t) curve from zero to 24 hours ($AUC_{(0-24)}$). These will be derived for each analyte. In addition, for analyte linezolid at BID dose, the AUC_{0-12}, C_{max}, C_{min}, CL/F and $t_{1/2}$ will be calculated based on dose interval 0-12 hrs.

6.5 Mycobacteriology Characterization Variables and Procedures

The following Mycobacterial Characterization variables will be collected:

Samples from:

- Day 1 (baseline) Early Morning and coached spot sputum samples (or Screening to Week 4 if the baseline is contaminated or negative);
- Any culture positive sample at or following end of treatment.

The *M.tb.* isolates will be processed at the central lab(s) for:

- MIC against bedaquiline, pretomanid and linezolid;
- Drug Susceptibility Testing in liquid culture for rifampicin, isoniazid, streptomycin, ethambutol, pyrazinamide and second line TB drugs including fluoroquinolones, and injectables;
- Extraction of bacterial DNA (*M.tb.*) for molecular genotyping;

The *M.tb.* isolates will be processed at study lab for:

- Speciation of the infecting organisms by molecular or antigen based test at baseline (Day 1 or screening to Week 4 if baseline is contaminated or negative) and initial relapse (first positive at end of treatment or during follow-up) or any positive at or after the week 16 visit

All Day 1 (baseline) *M.tb.* isolates and isolates from positive cultures to be stored at the study microbiology laboratory (or the central lab(s)) until trial closure for the applicable study tests. The extracted *M.tb.* DNA and isolates will be stored for potential further work to validate new assay tools for a maximum of 5 years after trial closure.

The Mycobacteriology sampling methodology and requirements will be described in a separate document, the Laboratory Manual, which will be provided prior to the trial start.

7 ADVERSE EVENTS

The Investigators are responsible for eliciting adverse events by observing the Subject and recording adverse events observed by him/her or reported by the Subject during the trial.

7.1 Definitions

7.1.1 Adverse Event (AE)

Any untoward medical occurrence in a patient or clinical investigation Subject administered a pharmaceutical product and which does not necessarily have a causal relationship with this treatment. An adverse event can therefore be any unfavorable and unintended sign (including an abnormal laboratory finding), symptom, or disease temporally associated with the use of a medicinal product, whether or not related to the medicinal product.

7.1.2 Serious Adverse Event (SAE)

Any untoward medical occurrence that at any dose:

- results in death;
- is life threatening (any event in which the Subject was at risk of death at the time of the event; it does not refer to an event, which hypothetically might have caused death if it were more severe);
- requires inpatient hospitalization or prolongation of existing hospitalization;
- results in persistent or significant disability/incapacity;
- is a congenital anomaly/birth defect; or
- is a medically important event.

Note: Medical and scientific judgment should be exercised in deciding which is a medically important event that may not be immediately life-threatening or result in death or hospitalization, but may jeopardize the Subject or may require medical or surgical intervention to prevent one of the outcomes listed above. Examples of such events are intensive treatment in an emergency room or at home for allergic bronchospasm, blood dyscrasias or convulsions that do not result in hospitalization, or the development of drug dependency or drug abuse. A “suspected transmission of infectious agent by a medicinal product” is also considered a serious adverse event under the SAE criterion “Other medically important condition”.

7.1.3 Unlisted (Unexpected) Adverse Event

An adverse reaction, the nature or severity of which is not consistent with the applicable product information (e.g., Investigator’s Brochure for an unapproved investigational product or package insert/summary of product characteristics for an approved product).

7.1.4 Life Threatening

Any event in which the Subject was at risk of death at the time of the event; it does not refer to an event, which hypothetically might have caused death if it were more severe.

7.1.5 Hopsitalization

Any adverse event leading to hospitalisation or prolongation of hospitalisation will be considered as serious, unless at least one of the following exceptions is met:

The admission results in a hospital stay of less than 12 hours;

or

The admission is pre-planned (i.e. elective or scheduled surgery arranged prior to the start of the study);

or

The admission is not associated with an adverse event (e.g. social hospitalisation for purposes of respite care);

or

Hospitalization is standard of care in the treatment of the subjects TB. However if the hospitalisation is prolonged due to the subjects TB symptoms worsening this will be considered serious.

However it should be noted that invasive treatment during any hospitalisation may fulfil the criteria of 'medically important', dependant on clinical judgement.

7.1.6 Associated with the Use of the Drug

An adverse event is considered associated with the use of the drug (Adverse Drug Reaction) if the attribution is possible, probable or very likely.

7.1.7 Attribution/Causality

The definitions for rating attribution/causality will be as described in Table 13.

Table 13: Adverse Events Attribution/Causality Ratings

Relatedness Rating	Definition
Not Related	An adverse event, which is not related to the use of the drug.
Unlikely	An adverse event for which an alternative explanation is more likely, e.g., concomitant drug(s) or concomitant disease(s), and/or the relationship in time suggests that a causal relationship is unlikely.
Possible	An adverse event, which might be due to the use of the drug. An alternative explanation, e.g., concomitant drug(s) or concomitant disease(s), is inconclusive. The relationship in time is reasonable; therefore the causal relationship cannot be excluded.
Probable	An adverse event, which might be due to the use of the drug. The relationship in time is suggestive, e.g., confirmed by dechallenge. An alternative explanation is less likely, e.g., concomitant drug(s) or concomitant disease(s).
Certain	An adverse event, which is listed as a possible adverse reaction and cannot be reasonably explained by an alternative explanation, e.g., concomitant drug(s) or concomitant disease(s).

7.1.8 Severity

Severity rating is to be made per the DMID Adult Toxicity Table (Appendix 2). For abnormalities **NOT found** elsewhere in the Toxicity Tables, the DMID scale described in Table 14 below is to be used to estimate grade of severity:

Table 14: Adverse Event Severity Ratings

Grade	Severity Rating	Definition
**GRADE 1**	Mild	Transient or mild discomfort (< 48 hours); no medical intervention/therapy required.
**GRADE 2**	Moderate	Mild to moderate limitation in activity - some assistance may be needed; no or minimal medical intervention/therapy required.
**GRADE 3**	Severe	Marked limitation in activity, some assistance usually required; medical intervention/therapy required, hospitalizations possible.
**GRADE 4**	Potentially Life-Threatening	Extreme limitation in activity, significant assistance required; significant medical intervention/therapy required, hospitalization or hospice care probable.

7.1.9 Other AE Definitions

The following definitions will be used for Adverse Event Reporting:

Action Taken with IMP

- IMP unchanged
- IMP interrupted
- IMP dose reduced
- IMP stopped
- Not applicable (Follow-up period)

Other Action Taken

- None
- Medication given
- Hospitalisation or prolongation of hospitalisation
- Therapeutic or diagnostic procedure

Outcome

- Resolved
- Improved
- Unchanged
- Worse
- Fatal
- Unknown

Occurrence

- Once
- Intermittent
- Continuous

7.2 Reporting

7.2.1 Adverse Event (AE)

Adverse events will be collected by the Investigator from the time a Subject signs the Informed Consent Form through to their Month 24 follow-up visit. The exception is early withdrawal Subjects who will only have SAEs collected from their time of early withdrawal to their Month 24 follow-up visit.

Any AE (serious or non-serious) observed by the Investigator or reported by the Subject will be recorded on the Adverse Event Case Report Form. The Investigator will review each AE and assess its relationship to drug treatment based on all available information at the time of the completion of the case report form. The following information will be recorded for each Adverse Event reported (definitions section 7.1):

- Diagnosis of the AE, if possible. In the case where an overall diagnosis cannot be made, each specific sign and/or symptom will be recorded as individual AEs;
- Date of onset;
- Stop Date (duration) if applicable;
- Severity;
- Action Taken with IMP;
- Other Action Taken;
- Outcome;
- Relationship to IMP;
- Occurrence;
- Seriousness.

7.2.2 Serious Adverse Event (SAE)

Any AE that occurs which is serious must be reported by the Investigator to the study monitor and copied to the Sponsor Medical Monitor within 24 hours of the site first being aware of the SAE, whether or not the serious event is deemed associated with the use of the drug.

In addition, the Investigator will provide a detailed, signed, written, and complete SAE report form that addresses the Investigator's estimates of the attribution/causality of the AE to the study drug and the seriousness of the AE in question to the study monitor and medical monitor within 24 hours of becoming aware of the SAE.

The study monitor will confirm receipt of the SAE Form with the Investigator and review the initial information on the SAE for diagnosis, consistency and completeness of data.

For submission of significant updated or additional information on a previously reported SAE, the Investigator will provide the study monitor and medical monitor with a newly completed Serious Adverse Event Form. This will be submitted to the study monitor and medical monitor within 24 hours of the Investigator receiving the information.

The study monitor will query for additional information from the Investigator, if necessary, to complete the profile of the SAE reported.

The Sponsor/Investigator/designee will inform Regulatory Authorities and/or IEC/IRB of all SAEs in accordance with local requirements and ICH guidelines for GCP.

The Sponsor/designee will forward Safety Notification letters to the Investigator for submission to the IEC/IRB

7.2.3 Follow up of Adverse Events

All AEs will be followed until:

- satisfactory clinical resolution or stabilization; or
- until the end of the follow-up period; and

- until all queries on these AEs have been resolved.

Certain long-term AEs cannot be followed until resolution within the setting of this protocol. In these cases follow-up will be the responsibility of the treating physician. However, this will have to be agreed upon with the Sponsor.

7.2.4 Post-Trial Adverse Events

Any new SAEs reported by the Subject to the Investigator that occur after the last scheduled contact, and are determined by the Investigator to be possible, probable or certainly related to the use of the IMP, will be reported to the Sponsor, IEC/IRB and regulatory authorities on an expedited basis as required in accordance with local requirements and ICH guidelines for GCP.

7.2.5 Clinical Laboratory Adverse Events

Changes in the results of the Clinical Laboratory assessment results which the Investigator feels are clinically significant will be reported as adverse events. It is the Investigators' responsibility to review the results of all laboratory tests as they become available. This review must be documented by the Investigators' dated signature on the laboratory report. For each abnormal laboratory test result, the Investigator needs to ascertain and document if this is a clinically significant change from baseline for that individual Subject. This determination, however, does not necessarily need to be made the first time an abnormal value is observed. The Investigator may repeat the laboratory test or request additional tests to verify the results of the original laboratory tests. If this laboratory value is determined by the Investigator to be a clinically significant change from baseline for that Subject, it is considered to be an adverse event.

7.2.6 Disease under Study

Symptoms of the disease under study (Pulmonary Tuberculosis) experienced by the Subject while on the study will be assessed by the Investigator. If the symptom has:

- worsened while the Subject is in the study; and
- the Investigator assesses it as clinically significant;

it will be recorded as an adverse event.

If there is:

- no change; and
- the Investigator assesses the symptom as due to the Subject's TB; and
- not clinically significant;

it will not be recorded as an AE and this will be noted in the Subject's source documentation.

All TB related symptoms that meet SAE criteria will be recorded and reported as a SAE.

7.2.7 Overdose

Overdose of IMP experienced by the Subject while on the study, will be assessed by the Investigator to determine whether the overdose led to an Adverse Event, including if the taking of the suspect medicine led to suicidal intention and subsequent overdose of the suspect medicine, or other medication. In this case it will be recorded as an adverse event. If it does not lead to an Adverse Event it will not be recorded as an AE and this will be noted in the Subject's source documentation.

7.2.8 Drug Interaction

If the Investigator becomes aware that the Subject has experienced a drug interaction which has resulted in an adverse event, it will be recorded as an adverse event.

7.2.9 Pregnancy

The Investigator will immediately notify the Sponsor of any pregnancy that is discovered during IMP administration or which started during IMP administration. Pregnancy forms will be completed for all pregnancies reported during the clinical trial, as defined below. In addition, the Investigator will report to the Sponsor follow-up information regarding the outcome of the pregnancy, including perinatal and neonatal outcome. Infants will be followed for 6 months.

All women of childbearing potential will be instructed to contact the Investigator immediately if they suspect they might be pregnant (for example, missed or late menses) for the following time-periods:

- During the trial;
- Within 6 months after last dose of IMP.

If pregnancy is suspected while the Subject is receiving IMP, the IMP will be withheld immediately until the result of the pregnancy test is known. If pregnancy is confirmed, the IMP will be permanently discontinued in an appropriate manner and the Subject withdrawn from the trial. Protocol-required procedures for trial discontinuation and follow-up will be performed unless contraindicated by the pregnancy. Should the female partner of a male Subject become pregnant during the study or in the 6 months after the completion of IMP and the Investigator becomes aware that this situation has occurred, consent will be requested from the female partner for collection of information on her pregnancy history and for information on the current pregnancy and birth.

Pregnancy reporting will **follow the same time lines and reporting structures as for a SAE** (see above). SAE reporting will also occur if the pregnancy outcome is a congenital anomaly. This will follow the reporting procedures described above for SAE reporting plus an additional clinical report compiled by the applicable company.

7.3 Monitoring and Safety for Specific Toxicities

AEs still ongoing at the end of treatment in the trial will be followed until satisfactory clinical resolution or stabilization or until the end of the follow-up period and until all queries on these AEs have been resolved. Grade 3 and grade 4 laboratory abnormalities and laboratory abnormalities considered clinically significant should be followed until satisfactory resolution or stabilization.

Note: For Grade 3 or 4 laboratory toxicities, Subjects should have a confirmatory measurement within 48 hours where possible. The recommendations for managing Subjects below assumes the laboratory abnormalities of concern have been confirmed.

Monitoring for specific toxicities is based upon target organs as defined in preclinical toxicity studies (Investigator's Brochures [4,5,15] and Package Inserts [3,18,31,34]).

7.3.1 ALT, AST and Alkaline Phosphatase elevations:

The Investigator should refer to Appendix 8 – Liver Toxicity Management to appropriately manage the Subject for clinically significant elevations of AST, ALT or Alkaline Phosphatase.

7.3.2 Amylase elevation

Grade 3 (> 2.0 to ≤ 5.0 x ULN):

Contact sponsor Medical Monitor to review. Further testing such as pancreatic amylase should be considered after consultation with the Sponsor Medical Monitor.

Grade 4 (> 5.1 x ULN):

Contact sponsor Medical monitor to review. Investigator should consider subjects with **confirmed Grade 4** elevations of total amylase for temporary or permanent discontinuation from the full regimen.

7.3.3 Lipase Elevation

Grade 3 (> 2.0 to ≤ 5.0 x ULN) or Grade 4 (> 5.0 x ULN):

Contact Sponsor medical Monitor to review. Investigator should consider subjects with **confirmed Grade 3 or 4** elevations of lipase for temporary or permanent discontinuation from the full regimen.

7.3.4 Musculoskeletal System and Cardiac Muscle

Myalgia

Grade 2 (muscle tenderness at site other than sites of injection and/or venipuncture or with moderate impairment of activity) or Grade 3 (severe muscle tenderness with marked impairment of activity) or Grade 4 (frank myonecrosis):

Subjects with Grade 2 signs and symptoms should be followed closely. Subjects with Grade 3 or 4 signs and symptoms should be discussed with the Sponsor Medical Monitor and to consider withholding study medication.

Subjects having **Grade 3 (3.1 to 6 x ULN) or Grade 4 (> 6 x ULN) elevation in CK-MB subunit** (with a confirmatory measure 7 days after the initial lab), the Investigator should consider discontinuing the full regimen and discuss with the Sponsor Medical Monitor.

7.3.5 Cardiac Rhythm Disturbances

Cardiac rhythm disturbances that are **Grade 3 (recurrent, persistent, symptomatic arrhythmia requiring treatment)** or **Grade 4 (unstable dysrhythmia requiring treatment):**

Subjects should be monitored closely. The Investigator should consider discontinuing the full regimen with the Sponsor Medical Monitor.

QTc prolongation

- If QTcF is equal to or greater than 500 msec, the ECG should be repeated and serum electrolytes should be evaluated. If the second ECG also has a QTcF of ≥ 500 msec, the full regimen should be withheld and the Sponsor Medical Monitor consulted.
- New left bundle branch block (LBBB) or Mobitz type 2 or complete heart block. Recordings with artifacts that interfere with the interpretation of the ECG should be repeated to confirm the findings. If the finding is from the centralized ECG machine reading the result is to be checked and confirmed by the Investigator. If this is confirmed by the Investigator, dosing is to be withheld until the reading has been confirmed by the central cardiologist and the Subject is to be treated per the Investigator's clinical judgment. If it is confirmed by the central cardiologist, the Subject is to be withdrawn from the full regimen

7.3.6 Myelosuppression

Investigator should consider withholding linezolid for subjects with:

- Neutropenia with an absolute neutrophil count below 750 (confirmed by repeat);
- Thrombocytopenia below 50,000;
- A drop in haemoglobin to ≤ 6 g/dL;

- Per investigator discretion, a reduction in haemoglobin ≥ 25% of the Subject's baseline value.

For participants who completed the first 4 weeks on linezolid 1200 mg per day, linezolid can be re-started at a later date at Investigator's discretion. If participant did not complete first 4 consecutive weeks of linezolid at 1200 mg per day, a lapse in treatment of 35 consecutive days or more should result in withdrawal from the study.

7.3.7 Peripheral Neuropathy

Investigator should consider withholding linezolid or discontinuing the full regimen permanently for subjects who if in the investigators opinion there is a significant worsening in peripheral neuropathy.

For participants who completed the first 4 weeks on linezolid 1200 mg per day, linezolid may be re-started at a later date at investigator's discretion. If participant did not complete first 4 consecutive weeks of linezolid at 1200 mg per day, a lapse in treatment of 35 consecutive days or more should result in withdrawal from the study.

7.3.8 Optic Neuropathy

Investigator should consider withholding linezolid and obtain further consultation with the site ophthalmologist for subjects with:

- A drop in visual acuity of two or more lines on the Snellen charts.
- Detection of loss of color vision by Ishihara plates defined as > 4 errors on the 12 plate screening test.

For participants who completed the first 4 weeks on linezolid 1200 mg per day, linezolid may be re-started at a later date at investigator's discretion. If participant did not complete first 4 consecutive weeks of linezolid at 1200 mg per day, a lapse in treatment of 35 consecutive days or more should result in withdrawal from the study.

7.3.9. Lactic Acidosis

Investigator should consider withholding linezolid for subjects who experience unexplained lactic acidosis characterized with low bicarbonate levels, weakness and nausea, and subjects should receive immediate medical evaluation by the Investigator.

For participants who completed the first 4 weeks on linezolid 1200 mg per day, linezolid may be re-started at a later date at investigator's discretion. If participant did not complete first 4 weeks of linezolid at 1200 mg per day, a lapse in treatment of 35 consecutive days or more should result in withdrawal from the study.

7.3.10 Neurological

Subjects with co-administration of a serotonergic agent, including anti-depressants, should be monitored closely for signs of serotonin syndrome. The Investigator should determine whether permanent discontinuation of the full regimen or the concomitant agent should be discontinued for those who experience signs or symptoms of serotonin syndrome such as cognitive dysfunction, hyperrexia, hyperreflexia and incoordination.

Linezolid and/or the full regimen should be withheld for subjects experiencing a seizure. The Sponsor Medical Monitor should be contacted to review details and discuss whether linezolid or full regimen should be resumed.

7.4 Safety Monitoring by the Data Safety Monitoring Committee

A DSMC will be appointed for the study. The primary responsibility of the DSMC will be to act in an advisory capacity to the Sponsor to safeguard the interests of trial Subjects by monitoring Subject safety, assess Subject risk versus benefit, and assess data quality and general evaluation of the trial progress. Its activities will be delineated in a DSMC charter that will define the membership, responsibilities and the scope and frequency of data reviews. The DSMC will operate on a conflict-free basis independently of the Sponsor and the study team. It will comprise at least 3 voting members. The DSMC may have an organisational meeting prior to commencement of the trial. The DSMC will have meetings where it will review unblinded data during a closed session. These meetings will be planned to occur every 6 months at a minimum. The Sponsor or the DSMC may convene ad hoc meetings based on rates of SAEs and/or to review results of the futility analysis or if safety concerns arise during the trial. After its assessment, the DSMC will recommend to the Sponsor continuation, modification or termination of the clinical trial.

8 STATISTICAL ANALYSIS

The statistical analysis plan (SAP), which will contain details of the analyses described generally in this section, will be written and signed off prior to Clinical Database Lock.

8.1 Analysis Population

The intention-to-Treat (ITT) analysis population will comprise of all subjects who were assigned study treatment.

The Safety analysis population will contain all subjects included in the ITT analysis population and received at least one administration of study drug.

The analysis populations will be defined in the SAP.

8.2 Sample Size

The objective of this trial is to evaluate the efficacy, safety, tolerability and pharmacokinetics of combinations of bedaquiline, linezolid and pretomanid in Subjects with either pulmonary XDR-TB, treatment intolerant or non-responsive MDR-TB.

Formal sample size calculations have not been performed due to the exploratory nature of the trial (no formal statistical hypothesis is therefore to be tested).

No formal interim analyses will be done for this study.

8.3 Interim Analyses

Timing of initial interim analysis will be conducted when the first 15 participants reach 6 months after completion of IMP. Further interim analyses will be specified in the statistical analysis plan (SAP).

Once all patients have been recruited or have completed the treatment period, no further futility analyses will be performed.

8.4 Primary Endpoint Analysis

The primary efficacy endpoint is treatment failure, defined as bacteriologic failure or relapse or clinical failure through follow-up until 6 months after the end of treatment.

The probability of treatment failure through follow-up until 6 months after the end of treatment, as a function of time after assignment of treatment, will be analyzed using Kaplan-Meier analysis.

The binomial proportion for subjects with bacteriologic failure will be presented.

No multiplicity adjustments for alpha will be done as this is an exploratory trial.

8.5 Secondary Endpoint Analysis

8.5.1 Efficacy

The secondary efficacy endpoints and analyses are as follows are:

- Incidence of bacteriologic failure or relapse or clinical failure through follow-up until 24 months after the end of treatment as a confirmatory analysis.
- Time to sputum culture conversion to negative status through the treatment period.

The time to sputum culture conversion will be analyzed using Kaplan-Meier analysis.

- Proportion of Subjects with sputum culture conversion to negative status at 4, 6, 8, 12 and 16 weeks with no subsequent, confirmed, positive culture(s).

- Proportion of Subjects experiencing a change from baseline of TB symptoms.

The binomial proportion for subjects with sputum culture conversion at each timepoint and subjects experiencing a change from baseline of TB symptoms will be presented.

- Change from baseline in Patient Reported Health Status.
- Change from baseline weight.

The change from baseline in Patient Reported Health Status will be summarized using descriptive statistics by visit.

The effect of baseline covariates may be explored, including but not limited to the presence or absence of cavities on Chest X-Ray, the presence or absence of HIV infection and CD4 cell count.

8.6 Exploratory Endpoint Analysis

8.6.1 Efficacy

The exploratory efficacy endpoints and analyses are as follows:

- Evaluate whether any of the secondary endpoints predicts relapse free cure.
-
- Subgroup analyses of the primary endpoint on the MITT analysis population will be considered
- Correlation of Time over mitochondrial protein synthesis inhibition (MPS50) with linezolid toxicity, (The MPS50 value will be an assumed value from literature).

Details for the analysis of the aforementioned endpoint will be described in the SAP.

8.6.2 Safety and Tolerability Analysis

- The incidence of all cause mortality will be summarized.
- All adverse events will be coded using the Medical Dictionary for Regulatory Activities (MedDRA) and will be presented by Preferred Term within each MedDRA System Organ Class (SOC).
- Treatment-emergent adverse events (TEAEs) are defined as AEs which started at or after the first administration of IMP and includes those events started prior to the first administration of IMP but which worsened after the first intake. Adverse events starting after the last administration of IMP until the last scheduled visit/assessment/measurement will be regarded as treatment-emergent.

- The incidence of the following events will be summarized for further medical analysis:
    - Incidence of TEAEs;
    - Incidence of TEAEs by Severity;
    - Incidence of TEAEs by DMID toxicity grade;
    - Incidence of Drug-Related TEAEs;
    - Incidence of Serious TEAEs;
    - Incidence of TEAEs Leading to Early Withdrawal;
    - Incidence of TEAEs leading to Death.
- Cardiovascular Safety: QT intervals will be adjusted using Fridericia's correction and Bazett's correction. QT/QTc values and changes from pre-dose (average of Screening and Day 1 values) at each time point will be summarized using descriptive statistics by group and time of collection. These will be presented as descriptive analyses, and no inferential tests will be carried out.
    - Post-baseline QT/QTc intervals will be classified into the following categories:
        - QT/QTc < 450 msec
        - 450 msec ≤ QT/QTc < 480 msec
        - 480 msec ≤ QT/QTc < 500 msec
        - QT/QTc ≥ 500 msec
    - QTc changes from baseline will be classified into the following categories:
        - increase < 30 msec,
        - ≥ 30 msec and < 60 msec, and
        - increase ≥ 60 msec.
    - Frequency counts will be used to summarize the number of Subjects at each time point according to the above categories.
    - ECG results will be classified as normal or abnormal (investigator assessment) and summarized using frequency counts by dose group and time of collection.
- Ophthalmology: Descriptive statistics, including changes from baseline, will be summarized and listed by Subject for ophthalmology slit lamp examination (age related eye disease study 2 [AREDS2] lens opacity classification and grading). Categorical data for lens opacity will be summarized in a frequency table for the right and left eye, respectively.
- Visual acuity and color vision: Descriptive statistics, including changes from baseline, will be summarized and listed by Subject for both Visual Acuity and Color Assessments. Categorical data for changes in visual acuity and color vision from baseline will be summarized in a frequency table for the right and left eye, respectively.
- Descriptive statistics of neuropathy data derived from Brief Peripheral Neuropathy Screen. Categorical data for observed signs and symptoms of neuropathy will be summarized in frequency tables, including changes in signs and symptoms from baseline.
- Other safety variables: Laboratory Parameters, Physical Examination, Vital signs (see Appendix 3), Concomitant medication, ophthalmic examination and peripheral neuropathy. Descriptive summary statistics will be presented. The incidence of liver related laboratory abnormalities will be explored.

8.7 Pharmacokinetics:

For each analyte (per visit), the PK plasma concentrations will be summarized by descriptive statistics, including the mean, SD, coefficient of variation (CV), median, minimum, maximum, geometric mean and geometric CV (%).

In addition, mean and median concentration-versus-time graphs will be provided (with error bars as appropriate).

For the PK sub-study samples, the following PK parameters will be estimated per analyte from the individual (per Subject) PK plasma concentrations: Minimum observed PK plasma concentration (C_{min}), maximum observed PK plasma concentration(C_{max}), time to reach C_{max} obtained without interpolation (T_{max}), area under the PK plasma concentration time (t) curve from zero to the last quantifiable PK plasma concentration prior to the subsequent dose, using the linear trapezoidal rule ($AUC_{(0-t)}$), area under the PK plasma concentration time (t) curve from zero to 24 hours ($AUC_{(0-24)}$).

8.8 Pharmacokinetics-Pharmacodynamics (PK-PD):

Further detail on correlations between plasma drug concentrations and efficacy and safety findings will be outlined in the SAP.

8.9 General Mycobacteriology

Descriptive summary statistics of the mycobacterial characteristics will be presented.

9 RECORDS MANAGEMENT

9.1 Data Collection

All CRF/eCRF pages will be completed for each Subject who receives any amount of IMP. For Screening Failure Subjects a Screening failure CRF/eCRF will be completed. For Subjects who are prematurely withdrawn, the visits up to withdrawal plus the withdrawal and applicable follow-up visits need to be completed.

9.2 Source Documents

Source documents are defined as all information in original records and certified copies of original records of clinical findings, observations, or other activities in a clinical trial necessary for the reconstruction and evaluation of the trial. Source documents will include, but are not limited to, progress notes, electronic data, screening logs, and recorded data from automated instruments.

All source documents pertaining to this trial will be maintained by the Investigators. The Investigator has to permit trial-related monitoring, audits, Independent Ethics Committee/Institutional Review Board (IEC/IRB) review and regulatory inspections providing authorized persons direct access to source documents.

9.3 File Management at the Trial Centre

It is the responsibility of the Investigators to ensure that the trial center files are maintained in accordance with International Good Clinical Practice Guidelines and the ethical principles that have their origin in the Declaration of Helsinki.

9.4 Records Retention at the Trial Centre

The Investigator is obliged to retain records and data from the trial for safety reasons and for audit and inspection subsequent to trial completion. The essential documents should be retained for not less than 5 years after the last approval of a marketing application and until there are no pending or contemplated marketing applications or at least 5 years have elapsed since the formal discontinuation of clinical development of the IMP.

The Sponsor will make financial provisions for the Investigator to deposit the documents at an external site for safekeeping for as long as required by regulations and the Sponsor.

10 QUALITY CONTROL AND ASSURANCE

10.1 Site Procedures

The Investigator undertakes to perform the clinical trial in accordance with this protocol, International GCP, and the ethical principles that have their origin in the Declaration of Helsinki, and applicable regulatory requirements.

The Investigator undertakes to complete the CRFs according to the Sponsor's requirements, in a timely, accurate and legible manner. CRF entries will be verifiable to source documentation other than the CRF.

Site Standard Operating Procedures will be adhered to for all clinical and bioanalytical activities relevant to the quality of the study. Subject compliance will be monitored throughout the study.

The Investigator will sign and date any analysis results (e.g. laboratory, ECG, etc.) to verify that the results have been reviewed.

The Investigator may appoint other Sub-Investigators to assist with the study. However the Investigator maintains responsibility for the study and will supervise the Sub-Investigators. Written IEC/IRB approval will be obtained prior to involvement in the study.

The Investigator will ensure that all site personnel are adequately trained in GCP, the protocol, IB and all study procedures and requirements.

10.2 Monitoring

The Investigator is responsible for the validity of all data collected at the clinical site and must accept the various monitoring procedures employed by the Sponsor. The purpose of monitoring is to verify that the rights and well-being of human Subjects are protected; that trial data are accurate, complete and verifiable with source data; and that the trial is conducted in compliance with the protocol, International GCP, the ethical principles that have their origin in the Declaration of Helsinki and the applicable regulatory requirements.

Monitors assigned by the Sponsor will conduct regular site visits for the purpose of monitoring various aspects of the study. Visits will take place usually within a predetermined interval, but this may vary during the course of the study. The Investigator and site staff will allow the study monitor and authorized representatives of the Sponsor to (1) inspect all CRFs, written informed consent documents and corresponding source documents (e.g. original medical records), Subject records and laboratory raw data, and (2) access clinical supplies, dispensing and storage areas. The Investigator and site staff should also (1) agree to assist with monitoring activities if requested and (2) provide adequate time and space for monitoring visits.

The monitor will query any missing, confusing, spurious, or otherwise ambiguous data with the Investigator. All queries should be resolved in a timely manner. A monitoring log will be maintained recording each visit, the reason for the visit, the monitor's signature and Investigator or designee's confirmation signature.

10.3 Auditing

For the purpose of compliance with International GCP and regulatory agency guidelines, it may be necessary for Sponsor-authorized Quality Assurance personnel and/or authorized personnel from an external regulatory agency to conduct an audit or inspection of the investigational site. The purpose of an audit is to assess the quality of data with regard to accuracy, adequacy and consistency, and to assure that studies are in accordance with the guidelines. Having the highest quality data from studies is an essential aspect of drug development.

The Investigator and site staff will be given sufficient notice to prepare for such visits, which will usually last between one and two days and may be conducted at any stage during the study. The audit will involve the review of all study-related documentation required by GCP to be maintained by each site; drug storage, dispensing and return; all study-related supplies; and source documents against the CRFs to assure the adequacy and accuracy of the information which has been recorded, including the verification of any AEs which have occurred.

In the event of the site being notified of a Regulatory Inspection, the Sponsor will help with preparation. It is essential that the Sponsor be notified of the inspection as soon as possible.

11 ETHICS AND REGULATORY

11.1 Basic Principles

This research will be carried out in accordance with International GCP, the ethical principles that have their origin in the Declaration of Helsinki and the applicable regulatory requirements.

11.2 Independent Ethics Committee/Institutional Review Board (IEC/IRB) Review

The protocol and required study related documents will be reviewed by the sites respective IEC/IRB. The study will not start until the IEC/IRB has approved the protocol, written informed consent, any written information to be provided to the Subject or any modification thereof, plus any other study related documents required for review. The IEC/IRB shall be constituted and shall operate in accordance with International GCP, the ethical principles that have their origin in the Declaration of Helsinki.

The Investigator will maintain an accurate and complete record of all submissions made to the IRB/IEC. The records should be filed in the Investigator's Study File, and copies will be sent to the Sponsor. The Investigator may delegate IRB/IEC communication responsibilities to another party/vendor (e.g. CRO). This delegation should be clearly documented in writing and filed with the study documents at the site.

11.3 Regulatory Authorities

The Regulatory Authorities will receive the protocol, amendments, reports on SAEs, and the Integrated Clinical Trial Report according to national regulations. As required by local legislation, written approval will be obtained from the Regulatory Authorities prior to commencement of the trial and implementation of e.g. amendments as applicable.

11.4 Informed Consent

Written informed consent will be obtained from all Subjects (or legally acceptable representative) before any trial-related procedures (including any screening or pre-treatment procedures) are performed. Investigators may discuss the availability of the trial and the opportunity for entry with a potential Subject without first obtaining consent. However, informed consent must be obtained and documented prior to initiation of any procedures that are performed solely for the purpose of determining eligibility for research, including withdrawal from current medication(s). When this is done in anticipation of, or in preparation for, the research, it is considered to be part of the research.

The Investigators have both ethical and legal responsibility to ensure that each Subject being considered for inclusion in this trial is given a full explanation of the protocol. This shall be documented on a written informed consent form that shall be approved by the same IEC/IRB responsible for approval of this protocol. Each informed consent form shall include the elements required by the international GCP and must adhere to the ethical principles that have their origin in the Declaration of Helsinki.

Once the appropriate essential information has been provided to the Subject and fully explained by the Investigators (or qualified designees) and it is felt that the Subject understands the implications of participating, the IEC/IRB approved written informed consent form will be signed and dated by both the Subject and the person obtaining consent (Investigators or designees), and by any other parties required by the IEC/IRB.

The original signed informed consent form will be kept with the trial records and a copy of signed informed consent form will be provided to the Subject. Another copy of the signed informed consent form and a source document identifying the trial and recording the dates of participation will be placed in the Subject's medical record.

The monitor will inspect the original completed consent form(s) for all Subjects.

11.5 Confidentiality

All site staff, the Sponsor, and any Sponsor representatives will preserve the confidentiality of all Subjects taking part in the study, in accordance with International GCP, applicable local legislation/regulations. Subject to the requirement for source data verification by the study personnel by reference to the Subject's notes, confidentiality of all Subject identities will be maintained. Only Subject study number and initials will be used on the CRF and in all study correspondence, as permitted. No material bearing a Subject's name will be kept on file by the Sponsor. The written informed consent will contain a clause granting permission for review of the Subjects' source data.

12 PUBLICATION POLICY

The definition of publication for this purpose is any public presentation of the data emerging from this study.

All unpublished information given to the Investigator by the Sponsor shall not be published or disclosed to a third party, other than to the responsible IEC/IRB, within the understanding of the confidentiality of their nature, without the prior written consent of the Sponsor.

Results of this research will be submitted for publication as soon as feasible upon completion of the study in the form of a joint publication(s) between Sponsor and Investigator(s), including site clinical and laboratory Investigators, as appropriate.

13 PROTOCOL AMENDMENT POLICY

Any change to the protocol will be effected by means of a protocol amendment. Any changes which affect Subject safety or welfare will be submitted to the IEC/IRB and Regulatory Authorities prior to implementation. The Investigator, IEC/IRB, and Sponsor must agree on all amendments. No amendment will be implemented until approved by the relevant Authorities and/or IEC/IRB and signed by all required parties. Exceptions to this are when the Investigator considers that the Subject's safety is compromised.

Protocol amendments detailing minor administrative changes should be submitted by the Investigator to the IEC/IRB and Regulatory Authorities, either for notification purposes or approval as appropriate.

14 FINANCIAL ASPECTS, INSURANCE AND INDEMNITY

The study Sponsor and funder is the Global Alliance for TB Drug Development (TB Alliance). The TB Alliance is a not for profit, product development partnership accelerating the discovery and development of new TB drugs that will shorten treatment, be effective against susceptible and resistant strains, be compatible with antiretroviral therapies for those HIV-TB Subjects currently on such therapies, and improve treatment of latent infection.

The TB Alliance works with public and private partners worldwide. It is committed to ensuring that approved new regimens are affordable, adopted and available to those who need them.

The Subjects will not receive any incentives for their involvement in the study. The Sponsor has made provision to reimburse the Subjects for out-of-pocket expenses such as travelling to and from the study site and other miscellaneous costs as a result of their study participation.

The Sponsor certifies that it has liability insurance coverage for itself and will provide an associated certificate upon request. The insurance does not relieve the Investigators of the obligation to maintain their own liability insurance as required by applicable law. The Sponsor does not assume any obligation for the medical treatment of other injuries and illnesses.

15 REFERENCES

Dietze R, Teixeira L, Rocha LMC, et al. "Safety and bactericidal activity of rifalazil in patients with pulmor tuberculosis" Antimicrob Agents Chemother 2001; 47 (7): 1972-6.

Donald PR, Sirgel FA, Venter A, et al. "Early bactericidal activity of antituberculosis agents" Expert Rev A infect Ther 2003; 1 (1): 141-55.

Package Insert, Sirturo™, Janssen Therapeutics, September 2014

TMC207 (Bedaquiline) Investigator's Brochure, Janssen Research and Development, Edition 9, 09 Octo 2015.

Tasneen R, Betoudji F, Tyagi S, et al. Contributions of oxazolidinones to the efficacy of novel regim containing bedaquiline and pretomanid in a mouse model of tuberculosis. Antimicrob. Agents Chemot Posted Online 26 October 2015. DoI:10.1128/AAC.01691-15..

Koul A, Dendouga N, Vergauwen K, et al. "Diarylquinolines target subunit c of mycobacterial ATP syntha Nat Chem Biol 2007; 3: 323-4.

Andries K, Verhasselt P, Guillemont J, et al. "A diarylquinoline drug active on the ATP synthase Mycobacterium turberculosis" Science 2005; 307: 223-7.

Huitric E, Verhasselt P, Andries K, Hoffner SE. "In vitro antimycobacterial spectrum of a diarylquinoline synthase inhibitor" Antimicrob Agents Chemother 2007; 51: 4202-4.

Koul A, Vranckx L, Dendouga N, et al. "Diarylquinolines are bactericidal for dormant mycobacteria as a re of disturbed homeostasis" J Biol Chem 2008; 283: 25273-80.

Mdluli K, Nuermberger, E. "Pre-Clinical Drug Combination Studies to Identify Novel Regimens for TB". Recent Advances in TB Drug Development symposium, 40th Union World Conference, International Ur Against TB and Lung Disease, Cancun, Mexico, 2009.

Mdluli K, Nuermberger E, Taylor K, Williams N, Koen A, Khisimuzi E, Rokeya T, Si-Yang L, Peloquin C, Dines "Sterilizing activity of novel TMC207- and PA-824-containing regimens in a murine model of tuberculo Antimicrobial Agents and Chemotherapy 2011; 55: 5485-5492.

Rustomjee R, Diacon AH, Allen J, et al. "Early bactericidal activity and pharmacokinetics of the diarylquino TMC207 in treatment of pulmonary tuberculosis" Antimicrob Agents Chemother 2008 Aug; 52(8): 283 Epub 2008 May 27.

Diacon AH, Dawson R, Von Groote-Bidlingmaier F, Symons G, Venter A, Donald PR, Conradie A, Erondu Ginsberg AM, Egizi E, Winter H, Becker P, Mendel CM. "Randomized dose-ranging study of the 14-day e bactericidal activity of bedaquiline (TMC207) in patients with sputum microscopy smear-positive pulmor tuberculosis" Antimicrob Agents Chemother. 2013 May; 57(5):2199-203. doi: 10.1128/AAC.02243-12.

Diacon AH, Dawson R, von Groote-Bidlingmaier F, Symons G, Venter A, Donald PR, van Niekerk C, Everit Winter H, Becker P, Mendel CM, Spigelman MK. "14-day bactericidal activity of PA-824, bedaquil pyrazinamide, and moxifloxacin combinations: a randomised trial". Lancet. 2012 Sep 15; 380(9846):986- doi: 10.1016/S0140-6736(12)61080-0. Epub 2012 Jul 23

Investigator's Brochure, Global Alliance for TB Drug Development, PA-824, Version 16, April 2014.

Stover, C. K., P. Warrener, D. R. VanDevanter, et al. 2000. "A small-molecule nitroimidazopyran drug candic for the treatment of tuberculosis" Nature 405:962–966.

Lenaerts AJ, Gruppo V, Marietta KS, Johnson CM, Driscoll DK, Tompkins NM, Rose JD, Reynolds RC, and O IM. "Preclinical Testing of the Nitroimidazopyran PA-824 for Activity against Mycobacterium tuberculosis Series of In Vitro and In Vivo Models" Antimicrob Agents Chemother. 2005 June; 49(6): 2294–2301.

Package Insert, ZYVOX™, Pfizer Pharmaceuticals, January 2014.

Oxford Textbook of Palliative Medicine, Oxford University Press. 1993; 109.

"Multidrug-resistant tuberculosis (MDR-TB) Fact Sheet October 2013 Update" © WHO October 2013 (number c7). Available: http://www.who.int/tb/challenges/mdr/mdr_tb_factsheet.pdf

Gandhi NR, et al. "Extensively drug-resistant tuberculosis as a cause of death in patients co-infected v tuberculosis and HIV in a rural area of South Africa" Lancet 2006 Nov 4; 368(9547):1575-80.

Centers for Disease Control and Prevention (2006) "Emergence of Mycobacterium tuberculosis with exten resistance to second-line drugs worldwide, 2000–2004" MMWR Morb Mortal Wkly Rep 55: 301–3 Available: http://www.cdc.gov/mmwr/preview/mmwrhtml/mm5511a2.htm

Pietersen E, et al. "Long-term outcomes of patients with extensively drug-resistant tuberculosis in South Afr a cohort study" Lancet, 2014 Jan 16. pii: S0140-6736(13)62675-6.

Lee, et al. "Linezolid for treatment of chronic extensively drug-resistant tuberculosis" N Engl J Med. 2012 18; 367(16):1508-18.

Leach KL, Brickner SJ, Noe MC, Miller PF. "Linezolid, the first oxazolidinone antibacterial agent" Ann NY A Sci. 2011 Mar; 1222:49-54. 2011. Lee, et al. "Prevalence of and risk factors for resistance to second-line dr in people with multi-drug resistant tuberculosis in eight countries: a prospective cohort study" Lancet. 2 Oct 20; 380(9851):1406-17.

Tato M, et al. "In vitro activity of linezolid against Mycobacterium tuberculosis complex, including multidr resistant Mycobacterium bovis isolates" Int. J. Antimicrob Agents 2006 Jul;28(1)75-8.

WHO Treatment of Tuberculosis Guidelines, 4th Edition. 2010.

Schecter GF, et al. "Linezolid Treatment for MDR-TB" Clinical Infectious Diseases 2010; 50:49-55.

Park IN, et al. "Efficacy and tolerability of daily-half dose linezolid in patients with intractable multidr resistant tuberculosis." B Antimicrobial Chemotherapy 2006; 58:701-704.;

Singla R. "Linezolid: an Effective, Safe and Cheap Drug for Patients Failing Multidrug-resistant Tubercul Treatment in India" Eur Respir J. 2012 Apr;39(4):956-62. doi: 10.1183/09031936.00076811. Epub 2011 29.

Approved Package Insert for South Africa, ZYVOXID™ (linezolid), Pfizer Pharmaceuticals, 02 March 2012.

Padayatchi N, Mac Kenzie WR, Hirsch-Moverman Y, Feng PJ, Villarino E, Saukkonen J, Heilig CM, Weiner M Sadr WM. "Lessons from a randomised clinical trial for multidrug-resistant tuberculosis" INT J TUBERC LU DIS 16(12):1582–1587,2012. http://dx.doi.org/10.5588/ijtld.12.0315

Cox H, Ford, N. "Linezolid for the treatment of complicated drug-resistant tuberculosis: a systematic rev and meta-analysis" INT J TUBERC LUNG DIS *16(4):447–454, 2012.*

Package Insert, Lizomac™ 600 (linezolid), MacLeods Pharmaceutical, 2014.

APPENDIX 1 THE IUATLD SCALE

The IUATLD scale proposes five groups for reporting the results of reading smears for acid fast bacilli. They should be recorded as follows:

FINDING	RECORDING
No acid-fast bacilli found in at least 100 fields	negative
1 to 9 acid-fast bacilli per 100 fields	exact figure/100/scanty positive
10 to 99 acid-fast bacilli per 100 fields	+
1 to 10 acid-fast bacilli per field in at least 50 fields	++
More than 10 acid-fast bacilli per field in at least 20 fields	+++

Reference: The Public Health Service National Tuberculosis Reference Laboratory and the National Laboratory Network. Minimum Requirements, Role and Operation in a Low-Income Country. International Union Against Tuberculosis and Lung Disease 1998.

APPENDIX 2 DIVISION OF MICROBIOLOGY AND INFECTIOUS DISEASES (DMID) ADULT TOXICITY TABLE

Source: U.S. National Institute of Allergy and Infectious Diseases, DMID, November 2007 (Draft)

ABBREVIATIONS: Abbreviations utilized in the Table:

ULN = Upper Limit of Normal	LLN = Lower Limit of Normal
R_x = Therapy	Req = Required
Mod = Moderate	IV = Intravenous
ADL = Activities of Daily Living	Dec = Decreased

ESTIMATING SEVERITY GRADE

For abnormalities NOT found elsewhere in the Toxicity Tables use the scale below to estimate grade of severity:

Grade	Severity Rating	Definition
**GRADE 1**	Mild	Transient or mild discomfort (< 48 hours); no medical intervention/therapy required.
**GRADE 2**	Moderate	Mild to moderate limitation in activity - some assistance may be needed; no or minimal medical intervention/therapy required.
**GRADE 3**	Severe	Marked limitation in activity, some assistance usually required; medical intervention/therapy required, hospitalizations possible.
**GRADE 4**	Potentially Life Threatening	Extreme limitation in activity, significant assistance required; significant medical intervention/therapy required, hospitalization or hospice care probable.

SERIOUS OR LIFE-THREATENING AEs

ANY clinical event deemed by the clinician to be serious or life-threatening should be considered a grade 4 event. Clinical events considered to be serious or life-threatening include, but are not limited to: seizures, coma, tetany, diabetic ketoacidosis, disseminated intravascular coagulation, diffuse petechiae, paralysis, acute psychosis, severe depression.

COMMENTS REGARDING THE USE OF THESE TABLES

- Standardized and commonly used toxicity tables (Division of AIDS, NCI's Common Toxicity Criteria (CTC), and World Health Organization (WHO)) have been adapted for use by the Division of Microbiology and Infectious Diseases (DMID) and modified to better meet the needs of patients in DMID trials.
- For parameters not included in the following Toxicity Tables, sites should refer to the "Guide For Estimating Severity Grade" located above.
- Criteria are generally grouped by body system.
- Some protocols may have additional protocol specific grading criteria, which will supersede the use of these tables for specified criteria.

HEMATOLOGY				
	**Grade 1**	**Grade 2**	**Grade 3**	**Grade 4**
**Hemoglobin**	9.5 - 10.5 gm/dL	8.0 - 9.4gm/dL	6.5 - 7.9 gm/dL	< 6.5 gm/dL
**Absolute Neutrophil Count**	1000-1500/mm^3	750-999/mm^3	500-749/mm^3	<500/mm^3
**Platelets**	75,000-99,999/mm^3	50,000-74,999/mm^3	20,000-49,999/mm^3	<20,000/mm^3
**WBCs**	11,000-13,000/ mm^3	13,000-15,000 /mm^3	15,000-30,000/mm^3	>30,000 or <1,000 /mm^3
**% Polymorphonuclear Leucocytes + Band Cells**	> 80%	90 – 95%	>95%	----------
**Abnormal Fibrinogen**	Low:<br>100-200 mg/dL<br>High:<br>400-600 mg/dL	Low:<br><100 mg/dL<br>High:<br>>600 mg/dL	Low:<br>< 50 mg/dL<br><br>----------	Fibrinogen associated with gross bleeding or with disseminated coagulation
**Fibrin Split Product**	20-40 mcg/ml	41-50 mcg/ml	51-60 mcg/ml	> 60 mcg/ml
**Prothrombin Time (PT)**	1.01 - 1.25 x ULN	1.26-1.5 x ULN	1.51 -3.0 x ULN	>3 x ULN
**Activated Partial Thromboplastin (APPT)**	1.01 -1.66 x ULN	1.67 - 2.33 x ULN	2.34 - 3 x ULN	> 3 x ULN
**Methemoglobin**	5.0 - 9.9 %	10.0 - 14.9 %	15.0 - 19.9%	> 20.0 %

**CHEMISTRIES**				
	**Grade 1**	**Grade 2**	**Grade 3**	**Grade 4**
**Hyponatremia**	130-135 mEq/L	123-129 mEq/L	116-122 mEq/L	< 116 mEq/L or abnormal sodium *with* mental status changes or seizures
**Hypernatremia**	146-150 mEq/L	151-157 mEq/L	158-165 mEq/L	> 165 mEq/L or abnormal sodium *with* mental status changes or seizures
**Hypokalemia**	3.0 - 3.4 mEq/L	2.5 - 2.9 mEq/L	2.0 - 2.4 mEq/L or intensive replacement therapy or hospitalization required	< 2.0 mEq/L or abnormal potassium *with* paresis, ileus or life-threatening arrhythmia
**Hyperkalemia**	5.6 - 6.0 mEq/L	6.1 - 6.5 mEq/L	6.6 - 7.0 mEq/l	> 7.0 mEq/L or abnormal potassium *with* life-threatening arrhythmia
**Hypoglycemia**	55-64 mg/dL	40-54 mg/dL	30-39 mg/dL	<30 mg/dL or abnormal glucose *with* mental status changes or coma
**Hyperglycemia (nonfasting and no prior diabetes)**	116 - 160 mg/dL	161- 250 mg/dL	251 - 500 mg/dL	> 500 mg/dL or abnormal glucose *with* ketoacidosis or seizures
**Hypocalcemia (corrected for albumin)**	8.4 - 7.8 mg/dL	7.7 - 7.0 mg/dL	6.9 - 6.1 mg/dL	< 6.1 mg/dL or abnormal calcium *with* life threatening arrhythmia or tetany
**Hypercalcemia (correct for albumin)**	10.6 - 11.5 mg/dL	11.6 - 12.5 mg/dL	12.6 - 13.5 mg/dL	> 13.5 mg/dL or abnormal calcium *with* life threatening arrhythmia
**Hypomagnesemia**	1.4 - 1.2 mEq/L	1.1 - 0.9 mEq/L	0.8 - 0.6 mEq/L	< 0.6 mEq/L or abnormal magnesium *with* life-threatening arrhythmia
**Hypophosphatemia**	2.0 - 2.4 mg/dL	1.5 -1.9 mg/dL or replacement Rx required	1.0 -1.4 mg/dL intensive therapy or hospitalization required	< 1.0 mg/dL or abnormal phosphate *with* life-threatening arrhythmia
**Hyperbilirubinemia (when accompanied by any increase in other liver function test)**	1.1 - <1.25 x ULN	1.25 - <1.5 x ULN	1.5 – 1.75 x ULN	> 1.75 x ULN
**Hyperbilirubinemia (when other liver function are in the normal range)**	1.1 - <1.5 x ULN	1.5 - <2.0 x ULN	2.0 – 3.0 x ULN	> 3.0 x ULN
**BUN**	1.25 - 2.5 x ULN	2.6 - 5 x ULN	5.1 - 10 x ULN	> 10 x ULN
**Hyperuricemia (uric acid)**	7.5 – 10.0 mg/dL	10.1 – 12.0 mg/dL	12.1 – 15.0 mg/dL	>15.0 mg/dL
**Creatinine**	1.1 - 1.5 x ULN	1.6 - 3.0 x ULN	3.1 - 6 x ULN	> 6 x ULN or dialysis required

**ENZYMES**				
	**Grade 1**	**Grade 2**	**Grade 3**	**Grade 4**
**AST (SGOT)**	1.1 - <2.0 x ULN	2.0 – <3.0 x ULN	3.0 – 8.0 x ULN	> 8 x ULN
**ALT (SGPT)**	1.1 - <2.0 x ULN	2.0 – <3.0 x ULN	3.0 – 8.0 x ULN	> 8 x ULN
**GGT**	1.1 - <2.0 x ULN	2.0 – <3.0 x ULN	3.0 – 8.0 x ULN	> 8 x ULN
**Alkaline Phosphatase**	1.1 - <2.0 x ULN	2.0 – <3.0 x ULN	3.0 – 8.0 x ULN	> 8 x ULN
**Amylase**	1.1 - 1.5 x ULN	1.6 - 2.0 x ULN	2.1 - 5.0 x ULN	> 5.1 x ULN
**Lipase**	1.1 - 1.5 x ULN	1.6 - 2.0 x ULN	2.1 - 5.0 x ULN	> 5.1 x ULN

**URINALYSIS**				
	**Grade 1**	**Grade 2**	**Grade 3**	**Grade 4**
**Proteinuria**	1+ or 200 mg - 1 gm loss/day	2-3+ or 1- 2 gm loss/day	4+ or 2-3.5 gm loss/day	nephrotic syndrome or > 3.5 gm loss/day
**Hematuria**	microscopic only <10 rbc/hpf	gross, no clots >10 rbc/hpf	gross, with or without clots, OR red blood cell casts	obstructive or required transfusion

**CARDIOVASCULAR**				
	**Grade 1**	**Grade 2**	**Grade 3**	**Grade 4**
**Cardiac Rhythm**		asymptomatic, transient signs, no Rx required	recurrent/persistent symptomatic Rx required	unstable dysrythmia; hospitalization and treatment required
**Hypertension**	transient increase > 20 mm/Hg; no treatment	recurrent, chronic increase > 20mm/Hg. /treatment required	acute treatment required; outpatient treatment or hospitalization possible	end organ damage or hospitalization required
**Hypotension**	transient orthostatic hypotension with heart rate increased by <20 beat/min or decreased by <10 mm Hg systolic BP, No treatment required	symptoms due to orthostatic hypotension or BP decreased by <20 mm Hg systolic; correctable with oral fluid treatment	requires IV fluids; no hospitalization required	mean arterial pressure <60mm/ Hg or end organ damage or shock; requires hospitalization and vasopressor treatment
**Pericarditis**	minimal effusion	mild/moderate asymptomatic effusion, no treatment	symptomatic effusion; pain; EKG changes	tamponade; pericardiocentesis or surgery required
**Hemorrhage, Blood Loss**	microscopic/occult	mild, no transfusion	gross blood loss; 1-2 units transfused	massive blood loss; > 3 units transfused

**RESPIRATORY**				
	**Grade 1**	**Grade 2**	**Grade 3**	**Grade 4**
**Cough**	Transient - no treatment	persistent cough; treatment responsive	Paroxysmal cough; uncontrolled with treatment	--------------------
**Bronchospasm, Acute**	transient; no treatment; 70% - 80% FEV_1 of peak flow	requires treatment; normalizes with bronchodilator; FEV_1 50% - 70% (of peak flow)	no normalization with bronchodilator;FEV_1 25% - 50% of peak flow; or retractions present	cyanosis: FEV_1 < 25% of peak flow or intubation necessary
**Dyspnea**	dyspnea on exertion	dyspnea with normal activity	dyspnea at rest	dyspnea requiring Oxygen therapy

GASTROINTESTINAL				
	**Grade 1**	**Grade 2**	**Grade 3**	**Grade 4**
**Nausea**	mild or transient; maintains reasonable intake	moderate discomfort; intake decreased significantly; some activity limited	no significant intake; requires IV fluids	hospitalization required;
**Vomiting**	1 episode in 24 hours	2-5 episodes in 24 hours	>6 episodes in 24 hours or needing IV fluids	physiologic consequences requiring hospitalization or requiring parenteral nutrition
**Constipation**	requiring stool softener or dietary modification	requiring laxatives	obstipation requiring manual evacuation or enema	obstruction or toxic megacolon
**Diarrhea**	mild or transient; 3-4 loose stools/day or mild diarrhea last < 1 week	moderate or persistent; 5-7 loose stools/day or diarrhea lasting >1 week	>7 loose stools/day or bloody diarrhea; or orthostatic hypotension or electrolyte imbalance or >2L IV fluids required	hypotensive shock or physiologic consequences requiring hospitalization
**Oral Discomfort/Dysphagia**	mild discomfort; no difficulty swallowing	some limits on eating/drinking	eating/talking very limited; unable to swallow solid foods	unable to drink fluids; requires IV fluids

**NEUROLOGICAL**				
	**Grade 1**	**Grade 2**	**Grade 3**	**Grade 4**
**Neuro-Cerebellar**	slight incoordination dysdiadochokinesis	intention tremor, dysmetria, slurred speech; nystagmus	locomotor ataxia	incapacitated
**Psychiatric**	mild anxiety or depression	moderate anxiety or depression; therapy required; change in normal routine	severe mood changes requiring therapy; or suicidal ideation; or aggressive ideation	acute psychosis requiring hospitalization; or suicidal gesture/attempt or hallucinations
**Muscle Strength**	Subjective weakness no objective symptoms/signs	mild objective signs/symptoms no decrease in function	objective weakness function limited	paralysis
**Paresthesia (burning, tingling, etc.)**	mild discomfort; no treatment required	moderate discomfort; non-narcotic analgesia required	severe discomfort; or narcotic analgesia required with symptomatic improvement	incapacitating; or not responsive to narcotic analgesia
**Neuro-sensory**	mild impairment in sensation (decreased sensation, e.g., vibratory, pinprick, hot/cold in great toes) in focal area or symmetrical distribution; or change in taste, smell, vision and/or hearing	moderate impairment (mod decreased sensation, e.g., vibratory, pinprick, hot/cold to ankles) and/or joint position or mild impairment that is not symmetrical	severe impairment (decreased or loss of sensation to knees or wrists) or loss of sensation of at least mod degree in multiple different body areas (i.e., upper and lower extremities)	sensory loss involves limbs and trunk; paralysis; or seizures

**MUSCULOSKELETAL**				
	**Grade 1**	**Grade 2**	**Grade 3**	**Grade 4**
**Arthralgia (joint pain)**	mild pain not interfering with function	moderate pain, analgesics and/or pain interfering with function but not with activities of daily living	severe pain; pain and/or analgesics interfering with activities of daily living	disabling pain
**Arthritis**	mild pain with inflammation, erythema or joint swelling – but not interfering with function	moderate pain with inflammation, erythema or joint swelling – interfering with function, but not with activities of daily living	severe pain with inflammation, erythema or joint swelling –and interfering with activities of daily living	permanent and/or disabling joint destruction
**Myalgia**	myalgia with no limitation of activity	muscle tenderness (at other than injection site) or with moderate impairment of activity	severe muscle tenderness with marked impairment of activity	frank myonecrosis

**SKIN**				
	**Grade 1**	**Grade 2**	**Grade 3**	**Grade 4**
**Mucocutaneous**	erythema; pruritus	diffuse, maculo papular rash, dry desquamation	vesiculation or moist desquamation or ulceration	exfoliative dermatitis, mucous membrane involvement or erythema, multiforme or suspected Stevens-Johnson or necrosis requiring surgery
**Induration**	< 15mm	15-30 mm	>30mm	
**Erythema**	< 15mm	15-30 mm	>30mm	
**Edema**	< 15mm	15-30 mm	>30mm	
**Rash at Injection Site**	< 15mm	15-30 mm	>30mm	
**Pruritus**	slight itching at injection site	moderate itching at injection extremity	itching over entire body	

**SYSTEMIC**				
	**Grade 1**	**Grade 2**	**Grade 3**	**Grade 4**
Allergic Reaction	pruritus without rash	localized urticaria	generalized urticaria; angioedema	anaphylaxis
Headache	mild, no treatment required	transient, moderate; treatment required	severe; responds to initial narcotic therapy	intractable; requires repeated narcotic therapy
Fever: oral	37.7 - 38.5 C or 100.0 - 101.5 F	38.6 - 39.5 C or 101.6 - 102.9 F	39.6 - 40.5 C or 103 - 105 F	> 40 C or > 105 F
Fatigue	normal activity reduced < 48 hours	normal activity decreased 25- 50% > 48 hours	normal activity decreased > 50% can't work	unable to care for self

APPENDIX 3 VITAL SIGNS

Vital Signs

The following abnormalities will be defined for vital signs:

**Abnormality Code**	**Vital Signs Parameter**			
	**Pulse**	**DBP**	**SBP**	**RR**
**Abnormalities on actual values**				
**“Abnormally low”**	≤ 50 bpm	≤ 50 mmHg	≤ 90 mm Hg	<12<br>Breaths per minute
**“Grade 1 or mild”**	-	> 90 mmHg-<br><100 mmHg	> 140 mmHg-<br><160 mmHg	17-20<br>Breaths per minute
**“Grade 2 or moderate”**	-	≥ 100 mmHg-<br><110 mmHg	≥ 160 mmHg-<br><180 mmHg	21-25<br>Breaths per minute
**“Grade 3 or severe”**	-	≥ 110 mmHg	≥ 180 mmHg	>25<br>Breaths per minute
**“Abnormally high or Grade 4”**	≥ 120 bpm	-	-	Intubation

APPENDIX 4 KARNOFSKY PERFORMANCE STATUS SCALE DEFINITIONS RATING (%) CRITERIA[19]

Description		%
Able to carry on normal activity and to work; no special care needed.	Normal no complaints; no evidence of disease.	100
	Able to carry on normal activity; minor signs or symptoms of disease.	90
	Normal activity with effort; some signs or symptoms of disease.	80
Unable to work; able to live at home and care for most personal needs; varying amount of assistance needed.	Cares for self; unable to carry on normal activity or to do active work.	70
	Requires occasional assistance, but is able to care for most of his personal needs.	60
	Requires considerable assistance and frequent medical care.	50
Unable to care for self; requires equivalent of institutional or hospital care; disease may be progressing rapidly.	Disabled; requires special care and assistance.	40
	Severely disabled; hospital admission is indicated although death not imminent.	30
	Very sick; hospital admission necessary; active supportive treatment necessary.	20
	Moribund; fatal processes progressing rapidly.	10
	Dead	0

Ref: Oxford Textbook of Palliative Medicine, Oxford University Press. 1993; 109.

APPENDIX 5 EQ-5D-5L QUESTIONNAIRE

Under each heading, please tick the ONE box that best describes your health TODAY

MOBILITY

I have no problems in walking about	❑
I have slight problems in walking about	❑
I have moderate problems in walking about	❑
I have severe problems in walking about	❑
I am unable to walk about	❑

SELF-CARE

I have no problems washing or dressing myself	❑
I have slight problems washing or dressing myself	❑
I have moderate problems washing or dressing myself	❑
I have severe problems washing or dressing myself	❑
I am unable to wash or dress myself	❑

USUAL ACTIVITIES *(e.g. work, study, housework, family or leisure activities)*

I have no problems doing my usual activities	❑
I have slight problems doing my usual activities	❑
I have moderate problems doing my usual activities	❑
I have severe problems doing my usual activities	❑
I am unable to do my usual activities	❑

PAIN / DISCOMFORT

I have no pain or discomfort	❑
I have slight pain or discomfort	❑
I have moderate pain or discomfort	❑
I have severe pain or discomfort	❑
I have extreme pain or discomfort	❑

ANXIETY / DEPRESSION

I am not anxious or depressed	❑
I am slightly anxious or depressed	❑
I am moderately anxious or depressed	❑
I am severely anxious or depressed	❑
I am extremely anxious or depressed	❑

SAMPLE

UK (English) v.2 © 2009 EuroQol Group. EQ-5D™ is a trade mark of the EuroQol Group

- We would like to know how good or bad your health is TODAY.
- This scale is numbered from 0 to 100.
- 100 means the <u>best</u> health you can imagine.
  0 means the <u>worst</u> health you can imagine.
- Mark an X on the scale to indicate how your health is TODAY.
- Now, please write the number you marked on the scale in the box below.

YOUR HEALTH TODAY = ☐

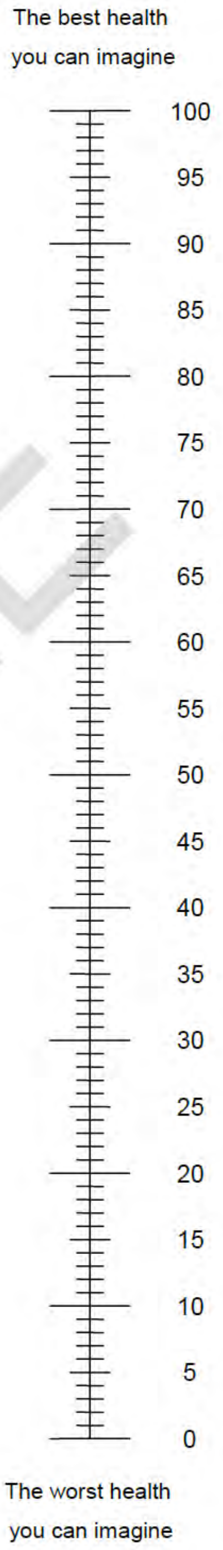

SAMPLE

UK (English) v.2 © 2009 EuroQol Group. EQ-5D™ is a trade mark of the EuroQol Group

APPENDIX 6 Brief Peripheral Neuropathy Screening

BRIEF PERIPHERAL NEUROPATHY SCREEN

Patient Initials				Patient ID									

1. Visit (Circle One)								
	All Subjects	Baseline	Week 4	Week 8	Week 12	Week 16	Week 20	Week 26
		3 Month	6 Month	12 Month	24 Month			
	9 Month Treatment **ONLY**	Week 30	Week 34	Week 39				
	Other	Early Withdrawal	**Unscheduled** For new onset or worsening peripheral neuropathy during treatment					
2. Date of Assessment	D	D	M	M	M	Y	Y	Y	Y

INTERFERENCE WITH WALKING OR SLEEPING

3. In the last two weeks, have pain, aching or burning in your feet interfered with your walking or sleeping? (Check one)	Y	N

3a. *If **YES**, ask the patient to rate the level of interference (1 to 10) to his walking or sleeping caused by this pain, ache or burning (circle one).*

Minimal			Modest				Severe		
01	02	03	04	05	06	07	08	09	10

SUBJECT ELICITED SYMPTOMS

- *Using the faces below, ask the patient to rate the severity of the symptoms for the questions 4, 5, 6 on a scale of 1 (mild) to 10 (severe) for both feet. If the severity is different between the left and right foot, record the severity of the most affected foot.*
- *Enter a score for each symptom.*
- *If a symptom has been present in the past, but not since the last visit, enter '00 – Currently Absent'*
- *If a symptom has never been present, enter '11 – Always Been Normal'*

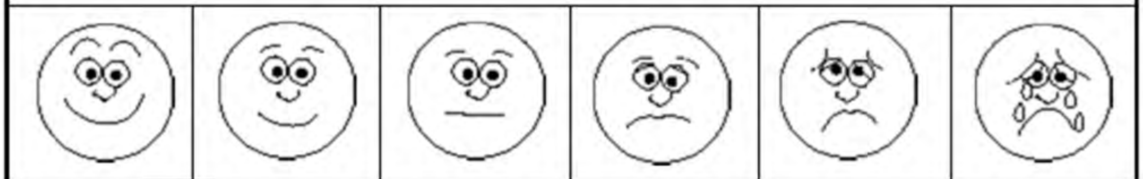

00	02	04	06	08	10
Very Happy, No Symptoms	Just a little bit	A little more	Even more	A whole lot	Worst

During the last 14 days, have you experienced:		Severity	
	4. Pain, aching or burning in feet or legs?		
	5. "Pins and needles" in feet or legs?		
	6. Numbness (lack of feeling) in feet or legs?		

BRIEF PERIPHERAL NEUROPATHY SCREEN

Patient Initials				Patient ID									

PERCEPTION OF VIBRATION

- *Press the ends of a 128 Hz tuning fork together so the sides touch and let go. Place the vibrating tuning fork on the bony prominence on the patient's wrist to be sure that they can recognize the vibration or "buzzing" quality of the tuning fork.*
- *Again, press the ends of the tuning fork hard enough so that the sides touch and let go. Immediately place the vibrating tuning fork gently but firmly on the top of the distal interphalangeal (DIP) joint of the great toe and begin counting the seconds. Instruct the Subject to tell you when they stop feeling the vibration or "buzzing".*
- *Repeat for the great toe on the other foot*

Vibration Perception Grade Scale:
0 – Vibration felt for >10 seconds (normal)
1 – Vibration felt for 6-10 seconds (mild loss)
*2 – Vibration felt for 5 seconds or less (moderate loss)**
*3 – No feeling of vibration (severe loss)**
*9 – Unable to evaluate or did not assess**

	Right	Left
7. Measured vibration grade of great toe DIP joint		

DEEP TENDON REFLEXES

- *The examiner uses one hand to press upward on the ball of the foot, dorsiflexing the Subject's ankle to 90 degrees. Using the reflex hammer (preferably long handled), the examiner strikes the Achilles tendon.*
- *The tendon reflex is felt by the examiner's hand as plantar flexion of the foot, appearing after a slight delay from the time the Achilles tendon was struck.*
- *Repeat for ankle on other leg*

Ankle reflex grade scale:
0 – Absent
1 – Hypoactive
2 – Normal deep tendon reflexes
3 – hyperactive deep tendon reflexes ***(e.g. with prominent spread of toes)***
4 – clonus
9 – unable to evaluate or did not assess

	Right	Left
8. Measured ankle reflex grade		

COMMENTS

Name of Person Completing Form		Name of Clinician (if required)	
Signature of Person Completing Form		Signature of Clinician (if required)	
Date	D D M M M Y Y Y Y	Date	D D M M M Y Y Y Y

APPENDIX 7 TUBERCULOSIS SYMPTOM PROFILE (V3)

TUBERCULOSIS SYMPTOM PROFILE (V3)

This questionnaire asks about symptoms that patients with tuberculosis may or may not experience.

Please read each symptom carefully and think about your experience **during the past 7 days** when you make your response. Then tick (☑) one box for each symptom.

If you **did not** experience the symptom **during the past 7 days**, please tick (☑) "None" for that symptom.

If you **did** experience the symptom **during the past 7 days**, please tick (☑) whether the intensity of the symptom you experienced was "Mild", "Moderate" or "Severe".

**TB Symptom**	**Rate your experience of each symptom over the past 7 days.**			
Feeling feverish	☐ None	☐ Mild	☐ Moderate	☐ Severe
Feeling chills	☐ None	☐ Mild	☐ Moderate	☐ Severe
Excessive sweating	☐ None	☐ Mild	☐ Moderate	☐ Severe
Shortness of breath	☐ None	☐ Mild	☐ Moderate	☐ Severe
Chest pain	☐ None	☐ Mild	☐ Moderate	☐ Severe
Feeling unwell	☐ None	☐ Mild	☐ Moderate	☐ Severe
Tiredness/weakness	☐ None	☐ Mild	☐ Moderate	☐ Severe
Cough	☐ None	☐ Mild	☐ Moderate	☐ Severe
Coughing up mucus	☐ None	☐ Mild	☐ Moderate	☐ Severe
Coughing up blood	☐ None	☐ Mild	☐ Moderate	☐ Severe

During the past 7 days, how would you rate your appetite?

☐ Good ☐ Fair ☐ Poor

Approved, Issued Date 09-Apr-2012

APPENDIX 8 LIVER TOXICITY MANAGEMENT GUIDELINES

Standard anti-TB chemotherapy is known to cause derangement of liver function tests in a substantial number of patients. In many cases, these will be asymptomatic and self-limiting. In some cases, severe hepatitis and even fulminant liver failure and death can occur.

In pre-marketing clinical trials of new drugs and regimens, it is especially important to identify and carefully manage any trial subjects who are at risk of progressing to serious liver injury. The observation of altered liver function to a degree with a high risk of progressing further to liver failure has been referred to informally as *Hy's Law* (Temple 2001; Reuben 2004); this reflects pure hepatocellular injury sufficient to cause hyperbilirubinemia is an ominous indicator of the potential for a drug to cause serious liver injury. Briefly, Hy's Law cases have the following three components:

1. The drug causes hepatocellular injury, generally shown by a higher incidence of 3-fold or greater elevations above the ULN of ALT or AST than the (non-hepatotoxic) control drug or placebo;
2. Among trial subjects showing such aminotransferase (AT) elevations, often with ATs much greater than 3x ULN, one or more also show elevation of serum total bilirubin (TBL) to >2x ULN, without initial findings of cholestasis (elevated serum ALP) ;
3. No other reason can be found to explain the combination of increased AT and TBL, such as viral hepatitis A, B, or C; pre-existing or acute liver disease; or another drug capable of causing the observed injury.

In a clinical trial of new drugs and combinations, it is especially important for Investigators to closely follow any Subjects who have evidence of potential hepatic inflammation or toxicity. During this trial, liver function will be monitored regularly via clinical assessments and blood tests to assist in determining which follow up laboratory measurements will either document resolution of abnormalities or signal the potential for drug-induced liver injury (DILI). The following procedure describes the management of deranged liver function tests.

<u>Procedure</u>

Blood tests for liver function will be taken routinely at Screening (Days -9 to -1), at the specific visits designated in the protocol and at Early Withdrawal. If at any other visit, the Investigator suspects derangement of liver function (e.g. the Subject describes nausea and vomiting, right upper abdominal pain or is jaundiced), blood should be taken for liver function tests and the Subject comprehensively assessed for evidence of hepatitis, hepatic impairment and any potentially contributing cause(s).

The laboratory source (print-out of any results) should be stored alongside or transcribed into the clinical source document. Each abnormal value should be marked as clinically significant (CS) or non-clinically significant (NCS); the assessment of significance is at the discretion of the Investigator. All abnormal results that are clinically significant must be recorded as Adverse Events in the eCRF and graded clinically per the DMID Adult Toxicity Table (Appendix 2).

Assessments and decision making for elevations in aminotransferase values or bilirubin of various levels of concern are detailed below:

<u>Decision to Consider Stopping Drug Regimen Administration</u>

Consideration of stopping drug administration, at least temporarily, to subjects with liver function abnormalities or signs and symptoms of hepatitis should be discussed with the Sponsor Medical Monitor in the following situations:

- ALT or AST >8x ULN;
- ALT or AST >5x ULN for more than 2 weeks;

- ALT or AST >3x ULN with the appearance of fatigue, nausea, vomiting, right upper quadrant pain or tenderness, fever, rash, and/or eosinophilia (>5%).

*If a subject has ALT or AST ≥3x ULN **and** Total Bilirubin >2x ULN, the IMP should be interrupted and the Subject's clinical course discussed with the Sponsor Medical Monitor.*

More detailed assessments and decision making for various levels of elevations in aminotransferase values, alkaline phosphatase or bilirubin are detailed below:

Grade 3 per DMID, ALT, AST, AP ≥ 3x ULN to 8x ULN or if a substantial increase from baseline (such as > 2-fold increase):

- Contact the Subject and recall them as soon as possible. Assess the Subject for other signs and symptoms of more specific hepatic events including hepatic impairment and/or hepatitis. If you are concerned, you should consider arranging for the subject to present to a medical facility (e.g. emergency department) immediately for assessment.
- Assess the clinical significance - if the Subject has jaundice, a coagulation disorder or signs of hepatic encephalopathy, all study medication should be withheld pending assessment/improvement.
- Assess possible contributing factors – This should include (but is not limited to), alcohol, intra-venous and other drug use, travel, unwell contacts, any medications with known hepatotoxic potential, herbal products and dietary supplements, previous or known hepatitis infection and exposure to environmental chemical agents. Although anti-TB chemotherapy is known to cause liver function test derangement, the Subject should always be assessed for other possible cause(s) or contributing factor(s).
- The Subject should also be advised to stop taking any medications/substances, other than the study medications used to treat TB that may be contributing to or causing derangement of liver function tests.
- Make every effort to repeat the testing of ALT, AST, AP and bilirubin within 48 hours to confirm the abnormalities and determine if they are increasing or decreasing. Consider any additional laboratory tests that may help characterize the Subject's clinical condition. Subjects should be tested for viral hepatitis (e.g. hepatitis A and B and any other tests available of viral hepatitis). If tests for viral hepatitis are not available or done, it may still be helpful to collect an additional 10ml sample for serum for freezing (5ml yellow/SST tube x2) which may be tested later. The Subject's consent must be obtained for this.

Elevated liver enzymes considered to be of clinical significance but not accompanied by other signs and symptoms, should be reported as an adverse event and recorded as elevated liver enzymes in the eCRF. If the term "hepatitis" is used, the Safety Data Manager will question the site for additional evidence to support the diagnosis, such as clinical signs, serological or biopsy data. While a liver biopsy is not required to make a diagnosis of hepatitis, the term "hepatitis" should be reserved in most instances for cases where there is supportive evidence beyond a liver enzyme abnormality. However, if the investigator confirms the diagnosis of hepatitis solely on the basis of clinical signs and laboratory values, the diagnosis will be accepted. Should other symptoms or signs be present, these should also be recorded as adverse events in the eCRF.

If ALT, AST, AP are Grade 4 per DMID (> 8x ULN):

- Contact the Subject and recall them as soon as possible. Generally, the trial medication should be stopped, but this should be discussed first with the Sponsor Medical Monitor whenever possible. Assess the subject for other signs and symptoms of more specific hepatic events, including hepatic impairment and/or hepatitis. If you are concerned, you should consider arranging for the subject to present to a medical facility (e.g. emergency department) immediately for assessment.

- Assess the clinical significance – Consider hospitalisation if the ALT is more than 10 times the ULN and/or the Subject has jaundice, a coagulation disorder or signs of hepatic encephalopathy. All study medications should be withheld pending assessment/improvement.
- Assess possible contributing factors – This should include (but is not limited to), alcohol, intra-venous and other drug use, travel, unwell contacts, any medications with known hepatotoxic potential, herbal products and dietary supplements, previous or known hepatitis infection and exposure to environmental chemical agents. Although anti-TB chemotherapy is known to cause liver function test derangement, the subject should always be assessed for other possible cause(s) or contributing factor(s).
- Make every effort to repeat the testing of ALT, AST, AP and bilirubin within 48 hours to confirm the abnormalities and determine if they are increasing or decreasing. Consider any additional laboratory tests that may help characterize the subject's clinical condition. Subjects should be tested for viral hepatitis (e.g. hepatitis A and B and any other tests available of viral hepatitis). If tests for viral hepatitis are not available or done, it may still be helpful to collect an additional 10ml sample for serum for freezing (5ml yellow/SST tube x2) which may be tested later. The Subject's consent must be obtained for this.

Elevated liver enzymes considered to be of clinical significance but not accompanied by other signs and symptoms, should be reported as an adverse event and recorded as elevated liver enzymes in the eCRF. If the term "hepatitis" is used, the Safety Data Manager will question the site for additional evidence to support the diagnosis, such as clinical signs, serological or biopsy data. While a liver biopsy is not required to make a diagnosis of hepatitis, the term "hepatitis" should be reserved in most instances for cases where there is supportive evidence beyond a liver enzyme abnormality. However, if the investigator confirms the diagnosis of hepatitis solely on the basis of clinical signs and laboratory values, the diagnosis will be accepted. Should other symptoms or signs be present, these should also be recorded as adverse events in the eCRF.

General Principles for following Subjects with potential liver toxicity

The Subject should be contacted regularly depending on the Grade of LFT elevations and the magnitude of increase relative to baseline for the Subject. Initially, this should be daily and subsequently depends on clinical course/individual circumstances. Staff must ensure all Subjects know to seek medical attention urgently if they experience any evidence of worsening liver disease. Symptoms may include (but are not limited to), malaise, fever, nausea, vomiting, loss of appetite, dark urine, yellowing of the eyes or skin (jaundice).

Liver function tests should be repeated regularly, such as every 3 days for the first week, then once a week until they return to near baseline values for the Subject. Manage the Subject symptomatically as required using medications that are not potentially hepatotoxic. Infection control issues must be carefully managed whilst TB medications are being withheld, especially if the Subject is still culture positive for acid fast bacilli.

Restarting Medication

If the Investigator (after consultation with the Sponsor Medical Monitor), stops administration of the study medication, consideration may be given to re-starting the study medication. Once the liver function values have decreased substantially and symptoms have significantly improved, a decision must be made about further TB management. This will be dependent on clinical context and the decision must be made in discussion with the Sponsor Medical Monitor. In all cases, treatment should be recommenced under close supervision for any evidence of recurrent liver function abnormalities.

If there is a further significant elevation of hepatic enzymes or bilirubin or symptoms of clinical concern after resumption of study medication, the study medication should be withdrawn permanently. Subjects who permanently discontinue study medication should be managed as clinically indicated according to local National TB Programme guidelines. The Sponsor Medical Monitor can provide advice and examples of suitable treatment regimens to use if required.

Certificate Of Completion

Envelope Id: 16017C17F6A74EE486A81E77E8A19E88

Status: Completed

Subject: Please DocuSign: NiX-TB Protocol Version 5.0 dated16Feb18_CL(FINAL).docx

Source Envelope:

Document Pages: 112

Signatures: 2

Certificate Pages: 5

Initials: 0

AutoNav: Enabled

EnvelopeId Stamping: Disabled

Time Zone: (UTC-05:00) Eastern Time (US & Canada)

Envelope Originator:
Joanna Moreira
40 Wall St FL 24
New York, NY 10005
Joanna.Moreira@tballiance.org
IP Address: 38.105.215.226

Record Tracking

Status: Original<br>4/9/2018 5:23:55 PM	Holder: Joanna Moreira<br>Joanna.Moreira@tballiance.org	Location: DocuSign

Signer Events	Signature	Timestamp
Dan Everitt<br>dan.everitt@tballiance.org<br>VP & Senior Medical Officer<br>Global Alliance for TB Drug Development (Part 11 Compliant)<br>Security Level: Email, Account Authentication (Required)	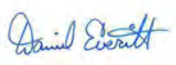<br>Signature ID:<br>32534894-D929-4A59-B14B-10FC37E90452<br>Using IP Address: 73.112.76.48<br><br>With Signing Authentication via DocuSign password<br>With Signing Reasons (on each tab):<br>I approve this document	Sent: 4/9/2018 5:24:16 PM<br>Viewed: 4/9/2018 7:40:08 PM<br>Signed: 4/9/2018 7:40:39 PM
**Electronic Record and Signature Disclosure:**<br>Not Offered via DocuSign		
Francesca Conradie<br>fconradie@witshealth.co.za<br>Security Level: Email, Account Authentication (Required)	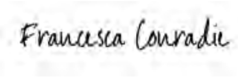<br>Signature ID:<br>8F3C422D-6DE0-4C72-AD39-5BC608A22CC5<br>Using IP Address: 169.1.48.218<br><br>With Signing Authentication via DocuSign password<br>With Signing Reasons (on each tab):<br>I approve this document	Sent: 4/9/2018 7:40:41 PM<br>Viewed: 4/10/2018 4:06:41 AM<br>Signed: 4/10/2018 4:08:02 AM
**Electronic Record and Signature Disclosure:**<br>Accepted: 8/29/2017 9:12:16 AM<br>ID: 0455f695-1e64-4dad-9776-29068566de85		

In Person Signer Events	Signature	Timestamp

Editor Delivery Events	Status	Timestamp

Agent Delivery Events	Status	Timestamp

Intermediary Delivery Events	Status	Timestamp

Certified Delivery Events	Status	Timestamp

Carbon Copy Events	Status	Timestamp

Notary Events	Signature	Timestamp

Envelope Summary Events	Status	Timestamps
Envelope Sent	Hashed/Encrypted	4/9/2018 7:40:41 PM
Certified Delivered	Security Checked	4/10/2018 4:06:41 AM
Signing Complete	Security Checked	4/10/2018 4:08:02 AM
Completed	Security Checked	4/10/2018 4:08:02 AM

Payment Events	Status	Timestamps

Electronic Record and Signature Disclosure

ELECTRONIC RECORD AND SIGNATURE DISCLOSURE

From time to time, Global Alliance for TB Drug Development-Sub Account (we, us or Company) may be required by law to provide to you certain written notices or disclosures. Described below are the terms and conditions for providing to you such notices and disclosures electronically through your DocuSign, Inc. (DocuSign) Express user account. Please read the information below carefully and thoroughly, and if you can access this information electronically to your satisfaction and agree to these terms and conditions, please confirm your agreement by clicking the 'I agree' button at the bottom of this document.

Getting paper copies

At any time, you may request from us a paper copy of any record provided or made available electronically to you by us. For such copies, as long as you are an authorized user of the DocuSign system you will have the ability to download and print any documents we send to you through your DocuSign user account for a limited period of time (usually 30 days) after such documents are first sent to you. After such time, if you wish for us to send you paper copies of any such documents from our office to you, you will be charged a $0.00 per-page fee. You may request delivery of such paper copies from us by following the procedure described below.

Withdrawing your consent

If you decide to receive notices and disclosures from us electronically, you may at any time change your mind and tell us that thereafter you want to receive required notices and disclosures only in paper format. How you must inform us of your decision to receive future notices and disclosure in paper format and withdraw your consent to receive notices and disclosures electronically is described below.

Consequences of changing your mind

If you elect to receive required notices and disclosures only in paper format, it will slow the speed at which we can complete certain steps in transactions with you and delivering services to you because we will need first to send the required notices or disclosures to you in paper format, and then wait until we receive back from you your acknowledgment of your receipt of such paper notices or disclosures. To indicate to us that you are changing your mind, you must withdraw your consent using the DocuSign 'Withdraw Consent' form on the signing page of your DocuSign account. This will indicate to us that you have withdrawn your consent to receive required notices and disclosures electronically from us and you will no longer be able to use your DocuSign Express user account to receive required notices and consents electronically from us or to sign electronically documents from us.

All notices and disclosures will be sent to you electronically

Unless you tell us otherwise in accordance with the procedures described herein, we will provide electronically to you through your DocuSign user account all required notices, disclosures, authorizations, acknowledgements, and other documents that are required to be provided or made available to you during the course of our relationship with you. To reduce the chance of you inadvertently not receiving any notice or disclosure, we prefer to provide all of the required notices and disclosures to you by the same method and to the same address that you have given us. Thus, you can receive all the disclosures and notices electronically or in paper format through the paper mail delivery system. If you do not agree with this process, please let us know as described below. Please also see the paragraph immediately above that describes the consequences of your electing not to receive delivery of the notices and disclosures electronically from us.

How to contact Global Alliance for TB Drug Development-Sub Account:
You may contact us to let us know of your changes as to how we may contact you electronically, to request paper copies of certain information from us, and to withdraw your prior consent to receive notices and disclosures electronically as follows:
To contact us by email send messages to: christi.baine@tballiance.org

To advise Global Alliance for TB Drug Development-Sub Account of your new e-mail address
To let us know of a change in your e-mail address where we should send notices and disclosures electronically to you, you must send an email message to us at christi.baine@tballiance.org and in the body of such request you must state: your previous e-mail address, your new e-mail address. We do not require any other information from you to change your email address..
In addition, you must notify DocuSign, Inc to arrange for your new email address to be reflected in your DocuSign account by following the process for changing e-mail in DocuSign.
To request paper copies from Global Alliance for TB Drug Development-Sub Account
To request delivery from us of paper copies of the notices and disclosures previously provided by us to you electronically, you must send us an e-mail to christi.baine@tballiance.org and in the body of such request you must state your e-mail address, full name, US Postal address, and telephone number. We will bill you for any fees at that time, if any.
To withdraw your consent with Global Alliance for TB Drug Development-Sub Account
To inform us that you no longer want to receive future notices and disclosures in electronic format you may:

i. decline to sign a document from within your DocuSign account, and on the subsequent page, select the check-box indicating you wish to withdraw your consent, or you may;
ii. send us an e-mail to christi.baine@tballiance.org and in the body of such request you must state your e-mail, full name, IS Postal Address, telephone number, and account number. We do not need any other information from you to withdraw consent.. The consequences of your withdrawing consent for online documents will be that transactions may take a longer time to process..

Required hardware and software

Operating Systems:	Windows2000? or WindowsXP?
Browsers (for SENDERS):	Internet Explorer 6.0? or above
Browsers (for SIGNERS):	Internet Explorer 6.0?, Mozilla FireFox 1.0, NetScape 7.2 (or above)
Email:	Access to a valid email account
Screen Resolution:	800 x 600 minimum
Enabled Security Settings:	•Allow per session cookies<br><br>•Users accessing the internet behind a Proxy Server must enable HTTP 1.1 settings via proxy connection

** These minimum requirements are subject to change. If these requirements change, we will provide you with an email message at the email address we have on file for you at that time providing you with the revised hardware and software requirements, at which time you will

have the right to withdraw your consent.

Acknowledging your access and consent to receive materials electronically

To confirm to us that you can access this information electronically, which will be similar to other electronic notices and disclosures that we will provide to you, please verify that you were able to read this electronic disclosure and that you also were able to print on paper or electronically save this page for your future reference and access or that you were able to e-mail this disclosure and consent to an address where you will be able to print on paper or save it for your future reference and access. Further, if you consent to receiving notices and disclosures exclusively in electronic format on the terms and conditions described above, please let us know by clicking the 'I agree' button below.

By checking the 'I Agree' box, I confirm that:

- I can access and read this Electronic CONSENT TO ELECTRONIC RECEIPT OF ELECTRONIC RECORD AND SIGNATURE DISCLOSURES document; and

- I can print on paper the disclosure or save or send the disclosure to a place where I can print it, for future reference and access; and

- Until or unless I notify Global Alliance for TB Drug Development-Sub Account as described above, I consent to receive from exclusively through electronic means all notices, disclosures, authorizations, acknowledgements, and other documents that are required to be provided or made available to me by Global Alliance for TB Drug Development-Sub Account during the course of my relationship with you.

Changes in the Conduct of the Nix-TB Trial Through Amendments

The original protocol dated 21 April 2014 was amended 4 times during the life of the trial. It is to be noted that there were 2 instances in which protocol modifications/clarifications were made via the use of a protocol memorandum and/or clarification letter to our Principal Investigators and trial center staff. These 2 protocol changes, which are detailed below, were later implemented into formal amendments.

The objective of the first memorandum was to strengthen the intent of protocol exclusion criterion number 1 and number 2, that patients should be excluded where participation in the trial would compromise the well-being of the patient or in the judgment of the Investigator, or when the patient was not expected to survive for more than 12 weeks. Due to the 4 early deaths in the trial which all occurred at Study center 02 – Brooklyn Chest Hospital –, an internal analysis was done to investigate any underlying risk factors for death occurring with the patient population that was being screened at Study center 02 versus Study center 01 – Sizwe Hospital. The analysis included a review of the safety data from 37 patients that were enrolled, including the 4 patients who died. Based on the analysis, it was identified that low weight, and particularly low BMI, as well as low albumin levels on screening, were more highly associated with the patients who subsequently died relative to the other patients in the trial. A recommendation letter was sent out to Study center 01 and Study center 02 on 02 May 2016, allowing the Investigators to use their discretion and their judgment when considering protocol exclusion criterion number 1 and number 2, and to consider avoiding enrolling patients with any of the following characteristics: BMI <17, weight <35 kg, or albumin <32 g/L. While the protocol was formally amended to add these exclusion criteria on 16 February 2018, all patients had been enrolled in the trial at that time. Documentation of the memorandum can be found in Appendix 16.1.1.

An additional memorandum and note to file were intended to clarify the original intent of wording in the protocol that was confusing and misinterpreted. There was misinterpretation around the intent of historical documentation of tuberculosis required for inclusion criterion number 6 for culture and DST for patients in the Nix-TB trial. Clarification memorandum number 7 was sent to Investigators on 05 June 2017, to clarify the intent of the wording in the protocol. This clarified an error in punctuation and noted the intent of the wording was that the timing of DST was not time-limited as long as it was for the patient's current episode of TB infection. Subsequently, a note to file was written 03 August 2017, to clarify that the intent of inclusion criterion number 6 was that the timing of both culture and DST also included any test results reported during Screening and did not exclude results of tests during Screening. This allowed for patients not to be considered to have violated the protocol if they had molecular confirmation of TB in their sputum or molecular tests for drug susceptibility done at screening. Both the Clarification memorandum #7 and the note to file were incorporated into a protocol amendment on 16 February 2018, although all patients had been enrolled in the trial at that time. Documentation of the memorandum and note to file can be found in Appendix 16.1.1.

Amendment 1 was instituted before the first patient was enrolled, while Amendment 2, Administrative Change 3, and Amendment 4 were instituted after the patient enrollment. A copy of the most recent protocol is provided in Appendix 16.1.1. Brief summaries of the non-administrative changes are outlined below.

Amendment 1 dated 18 March 2015 implemented the following changes:

- Added an allowance for patients to continue on bedaquiline and pretomanid alone after linezolid toxicity issues if they have completed at least 1 full month of treatment with the specified dose of 600 mg BID and were smear-negative, as well as preclinical data in support of the limited linezolid administration/continuation of bedaquiline and pretomanid post linezolid toxicity;
- Amended frequency of DSMC meetings to at least every 6 months;
- Amended the general mycobacteriology assessments to clarify only single sample is to be used for mycobacteriology characterization, and only the first positive culture at the End of Treatment if the first positive culture is contaminated is to be characterized;
- Clarified exclusion criteria number 5 for CD4/ARV regarding specific CD4 exclusion and expanded on the wording for allowable ARVs;
- Corrected exclusion criteria number 16 (g), total bilirubin ranges from greater than (>) to greater than or equal (≥);
- Corrected exclusion criteria number16 (h), serum creatinine level from less than to greater than 2 × ULN;
- Corrected the temperature range for the storage of the trial treatments from between 15ºC to 30ºC to 15ºC to 25ºC;
- Specified that respiratory rate was part of vital signs assessments and included a vital signs abnormality GRADE appendix;
- Added an option to allow for patients to be re-screened fully after a screen failure;
- Amended the options for the action taken with the trial treatment as a result of an AE to include “dose reduced”;
- As trypsin-like immunoreactivity test was not typically available in the country where the trial was performed, mention of it was removed as any indications of pancreatitis were monitored in the trial by means of lipase, total amylase, and clinical signs/symptoms;
- Removed the Tukey honestly significant difference from the QTc analysis as there was only 1 treatment regimen;
- Corrected numbering on the Brief Peripheral Neuropathy Screening scale in Appendix 6 of the protocol (corrected reference to interviewer questions 4, 5, and 6 instead of 8, 9, and 10 as originally written).

Amendment 2 dated 22 January 2016 implemented the following changes:

- Changed linezolid dosing from 600 mg BID to 1200 mg QD based on preliminary data from a linezolid clinical trial that noted similar bactericidal effect on TB when either dosing scheme was given for 14 days. A single daily dose of the total amount may have been less toxicity as, the time over expected toxicity concentrations was lower when administered QD. Removed 300 mg BID option to simplify, all regimens now QD dosing. Clarified dosing with meal (specified elsewhere in the protocol) within synopsis. Added allowance for continuing trial treatment without linezolid if the patients received at least 4 weeks of the 1200 mg total daily dose;
- Amended the primary endpoint follow-up from 24 months following End of Treatment to 6 months following the End of Treatment to be in line with Sponsor’s other Phase 3 trial and because the majority of relapses were expected within 6 months of completion of treatment;
- Added secondary endpoint for bacteriologic failure or relapse or clinical failure through 24 months following End of Treatment;

- Removed TTP as a secondary endpoint;
- Added secondary endpoint of change from baseline weight;
- Changed the number of PK sub-trial patients from 30 to 20 patients, as 20 patients would give adequate precision to estimate the PK parameters of interest;
- Noted speciation of *M. tuberculosis* isolates was done by the trial laboratory (also clarified definition of trial laboratory) and not by the central laboratory at baseline, and first positive at the End of Treatment or through follow-up and added to be done for any positive culture on or after the Week 16 visit to ensure informed treatment duration decisions. This assured that late positive cultures were truly *M. tuberculosis* and not a new infection with a non-TB mycobacteria;
- Amended interim analysis to be more general as details were to be outlined in the SAP;
- Corrected the visit windows on the trial flow chart for Weeks 12 through 16 (weekly visits) to note ± 3 days instead of ± 7 days. Also added details of assessment for unscheduled visits due to treatment extension and clarified windows for within visit assessments;
- Incorporated the collection of AEs and concomitant medications at follow-up visits held 1 and 2 months after the last trial treatment administration;
- Clarified on an ambiguity regarding the required follow-up visits in relation to number of trial treatment administrations received. Less than 15 administrations did not require the follow-up visits;
- Clarified that all HIV-positive patients were to have a CD4 count performed;
- Clarified timing of and specific assessments at the final treatment visit for patients who extended their treatment to 9 months due to interruptions;
- Amended on the HIV status that if the patient’s HIV status was a confirmed known positive, a repeated HIV test at Screening was not required;
- Amended and clarified on the tests to be performed on the sputum samples and the mycobacteriology characterization;
- Corrected inclusion requirement for resistance testing prior to enrollment, removed time restriction on date of results. Allowing the molecular tests to replace historical positive culture;
- Added exclusion for alcohol/illegal drug abuse;
- Corrected exclusion requirement regarding HIV infected patients and CD4 count. The “less than” sign was missing to note that counts less than 50 were exclusionary. Amended suggestions for ARV treatment based on toxicities;
- Added CYP3A4 inhibitors and inducers to specific treatment exclusion;
- Added exclusion of patients who had received more than 2 weeks of bedaquiline or linezolid;
- In order to ensure the safety of the patients enrolled in the trial more stringent safety laboratory monitoring was added to exclusion criterion number 19;
- Changed time period required between cultures to be in line with other Phase 3 trial;
- In order to ensure the safety of the patients enrolled in the trial, information on concomitant medications known to be hepatotoxic was added as there were cases of elevated ALT and AST in another trial using pretomanid with other medications that were associated with elevated ALT and AST;
- Removed requirement for coagulation laboratory tests at Screening;

- Added definitions for the circumstances under which hospitalization would not to be reported as an SAE and clarified on SAE follow-up reporting;
- The following items were updated to clarify ambiguity:
  - Withdrawal form treatment versus withdrawal from trial. Added treatment non-compliance as a reason for withdrawal. Corrected protocol section reference;
  - Definition of treatment completion;
  - Changed "strain typing" to "genotyping";
  - Culture was done at Screening to be in line with existing wording noting that Screening through Week 4 culture could be considered baseline;
  - Added reference to additional relevant protocol section;
  - Direct Microscopy as used in the protocol was intended to mean direct examination of the smear after the sputum had been decontaminated and concentrated and not that microscopy was to be performed directly from the sputum sample;
  - Recommended linezolid dose interruptions for toxicities;
  - Source of early morning sputum sample. Removed MGIT TTP from efficacy variable;
  - Options for trial treatment returns and destruction.

Protocol Administrative Change Number 3 dated 24 April 2017 implemented the following changes:

- Addressed a typographical error;
- Clarified on the number of patients required for the PK sub-trial.

Amendment 4 dated 16 February 2018 implemented the following changes:

- Removed reference in the protocol of pyrazinamidase encoded in the genes of the mycobacterium species (pncA) sequencing from the mycobacterial characterization, as this will not be done;
- Correction of Month 12 follow-up to Month 6 follow-up;
- Based on the Efficacy SAP, descriptive analysis of the subgroup of populations by HIV status and CD4 count as an exploratory point will not be performed. The protocol language was changed to make it broader and in line with the language in the Efficacy SAP;
- Aligned the protocol and Laboratory Manual with regards to the collection of 2 spot sputum samples onsite when an early morning sputum sample was not available;
- Corrected trial flow chart to show that the collection of AEs, concomitant medication and trial treatment compliance was to be performed at each visit during the treatment period rather than every third visit;
- Updated inclusion criterion number 2 to include body weight of ≥35 kg instead of ≥30 kg;
- Clarified on inclusion criterion number 6, so that the eligibility assessment of XDR-TB per inclusion criterion number 6a, was to have documented culture and drug-sensitivity testing results within 3 months prior to treatment assignment, including any test results reported during Screening;

- Added new exclusion criterion number 5, patients were not to have a BMI of <17 kg/m^2;
- Clarified on exclusion criterion number 20, patients were not to have an albumin level of <32 g/L;
- Clarified on exclusion criterion number 20, total bilirubin was not to be Grade 3 or greater (≥2.0 × ULN), or ≥1.5 up to 2.0 ×ULN when accompanied by any increase in other liver function test instead of only total bilirubin of >1.5 × ULN to be excluded;
- Modified the protocol language regarding treatment extension to go beyond the timepoint of just Month 4 visit and include specific language stating option of treatment extension can be considered if culture results are positive or revert to being culture positive between Month 4 and Month 6 visit with consideration of the patient's clinical conditions. All decisions regarding treatment extensions are to be discussed with the medical monitor prior to any implementation;
- With regards to dose interruptions or modifications, the following stipulation was added: When the total of missed dosing days and/or pauses is greater than 7 days, the same number of missed dosing days should be dispensed/treatment extended to make up for the total missed doses.

<u>Changes to the AE causality reporting and action taken with the trial treatment</u>

Originally the Investigator's assigned AE attribution/causality to the overall treatment regimen. However, according to the Data Handling Report (dated 23 November 2017), from Version 8 of the eCRF, the relationship to each trial drug and action taken with each trial drug was added to the eCRF. As it was only added on 17 February 2017 these fields only had to be completed for AEs with start dates on or after 17 February 2017.

Nix-TB
Efficacy Statistical Analysis Plan

Protocol Title: Protocol Title: A Phase 3 open-label trial assessing the safety and efficacy of bedaquiline plus pretomanid plus linezolid in Subjects with pulmonary infection of either extensively drug-resistant tuberculosis (XDR-TB) or treatment intolerant / non-responsive multi-drug resistant tuberculosis (MDR-TB).

Protocol Number: NiX-TB-(B-L-Pa).

Version: *1.0*

Author name:	Angela Crook
Author position:	Trial Statistician
Author signature:	
Date:	11 July 2017
Approval name:	Dan Everitt
Approval position:	Senior Medical Officer
Approval signature:	
Date:	11 July 2017

Table of Contents

1. Introduction 4

2. Primary Efficacy Endpoint 4

3. Definitions and data handling issues 5

3.1. Definitions 5

3.2. Inability to produce sputum 6

3.3. Isolated positive cultures... 6

3.4. Timing of events 6

4. Analysis populations 6

4.1. Exclusions from ITT analysis (late screening failures) 7

4.2. Additional exclusions from MITT analysis 7

4.3. Additional exclusions from PP analysis 8

4.4. Lost to Follow-up or Early Withdrawal 8

4.5. Definition of adequate treatment 9

4.6. Determining cause of death 9

5. Baseline comparisons of key characteristics 9

6. Classification of primary endpoint status 10

6.1.1. Favourable status (all analyses).. 10

6.1.2. Unfavourable status in ITT population 10

6.1.3. Unfavourable status in MITT population... 10

6.1.4. Unfavourable status in PP population... 10

7. Primary endpoint analysis... 10

8. Sensitivity analyses of primary endpoint analysis 11

9. Secondary efficacy analyses of primary endpoint.. 11

9.1. Time to event unfavorable outcome analysis 11

10. Secondary efficacy endpoints 11

10.1. Incidence of bacteriologic failure or relapse at 24 months after the end of treatment 11

10.2 Time to sputum culture conversion to negative status... 11

10.3 Culture conversion status at 4, 6, 8, 12 and 16 weeks 11

10.4 TB symptoms and weight 11

10.5 Patient reported health status 12

10.6 Weight 12

11 Pharmacokinetics-Pharmacodynamics (PK-PD)analyses 12

12 Sub-group analyses 12

13 Reasons for treatment failure as determined by the local PI 12

14 Further exploratory analyses 12

15 APPENDICES 13

15.1 Appendix 1 Algorithm for Interpretation of Positive MGIT Results 13

15.2 Appendix 2 Interpretation of Relapse and Re-infection using Whole Genome Sequence (WGS) data

Version History:

Version Number/Date	Change
0.1 01May2017	First version drafted
0.2 12 May2017	Incorporated comments from team
0.3 23 May2017	Incorporated comments from team after meeting on 22 May 2017
0.4 21 June 2017	Incorporated comments from team after meeting on 20 June 2017
0.5 03 July 2017	Changes incorporated included possible change to timing of primary endpoint and clarification of derived MGIT results in Appendix 1
0.6 10 July 2017	Pre-final version following call on 10 Jul 2017
1.0 11 July 2017	Final version approved

1. Introduction

This document outlines the efficacy statistical analysis plan (SAP) for the protocol Nix-TB, a phase 3 open-label clinical trial assessing the efficacy, safety, tolerability and pharmacokinetics of bedaquiline plus pretomanid plus linezolid in patients with pulmonary of either extensively drug-resistant tuberculosis (XDR-TB) or treatment intolerant / non-responsive multi-drug resistant tuberculosis (MDR-TB).

Up to 200 patients will be enrolled and all patients will be treated with bedaquiline 400 mg once daily for 2 weeks then 200mg 3 times per week plus pretomanid 200mg once daily plus linezolid, initially at 600 mg bid, then amended on Jan 22, 2016, to 1200mg once daily.

Treatment duration will be 6 months, with the option to extend to 9 months if the patient remains culture positive at 4 months from start of treatment (and is not withdrawn from the study).

Patients who complete treatment will return for follow-up visits 1, 2 and 3 months after end of treatment then every 3 months up to 24 months after end of treatment. Patients who withdraw after ≤14 days of IMP administration will return for an Early Withdrawal visit only. Patients who withdraw after ≥15 days of IMP will return for the Early Withdrawal, and for the 3, 12 and 24 month follow up visits after their last dose of IMP.

The Data Safety Monitoring Committee (DSMC) will review the data at least every 6 months. In addition, interim analyses will be performed cumulatively on every 15 patients who complete treatment (or are withdrawn early). Consideration will be given to stopping the trial early for safety concerns or futility although there are no formal stopping rules. This document covers interim analyses after the first one done on the first 15 participants, and final analyses including the analyses for the New Drug Application (NDA). The NDA will be based on the first 45 patients reaching primary endpoint and additional data collected by that time point will also be summarised.

The primary efficacy analysis will be conducted using culture results from liquid culture (MGIT). This document covers

2. Primary Efficacy Endpoint

The primary efficacy endpoint will be the incidence of bacteriologic failure or relapse or clinical failure at 6 months after the end of therapy. See section 6 for the detailed definition of an "unfavourable response".

There will be three main analyses of the primary efficacy endpoint: An intent to treat (ITT) analysis; a modified intent to treat (MITT) analysis and a per protocol (PP) analysis.

The "unfavourable" rates in any defined 'ITT' population will likely be increased by factors other than bacteriologic or clinical treatment failure and relapse. The MITT analysis will therefore be considered primary for publication purposes. However, we recognize that FDA and other regulatory agencies will consider the ITT analysis primary for the purpose of the NDA filing.

NB: In the event that more than 10% of patients are culture positive at 4 months and have their treatment extended for further 3 months, the primary endpoint analysis will be defined as 15 months from start of therapy for all patients.

3. Definitions and data handling issues

3.1. Definitions

Positive culture refers to the culture being positive for M.tb. False positive or contaminated sputum cultures, without speciation data confirming presence of M.tb, will be treated as missing. Specimens classified as non-tuberculous mycobacteria (NTM) and negative for M.tb will be treated as contaminated. Full details of the bacteriology algorithm for reporting MGIT results can be found in Appendix 1. Two sputum samples per visit are collected at each visit throughout treatment and follow-up. The culture result for a given visit is established using all samples obtained for that visit. A positive culture takes precedence over a negative culture at the same visit.

Culture negative status is achieved when a patient produces at least 2 negative culture results at different visits (at least 7 days apart) without an intervening positive culture result for M.tb. The date of the first negative culture of these two is the date at which culture negative status was obtained. Once obtained, culture negative status continues until there are two positive cultures at different visits (at least 7 days apart), without an intervening negative culture, or until there is a single positive culture not followed by two negative cultures. Culture negative status can be achieved at any time during treatment or follow-up but before any re-treatment. Culture negative status can be re-established.

Patients with two contaminated or missing samples at a given visit will be asked to return to produce two more sputum samples.

Treatment failure is defined as being declared an unfavourable status (as defined in section 6) at or before the end of treatment or failing to attain culture negative status and being declared an unfavourable outcome or patient is withdrawn at or before the end of treatment for clinical (TB) reasons including being re-treated (or changing from protocol treatment) for TB.

Relapse is defined as failing to maintain culture negative status or being declared an unfavourable outcome after the end of treatment in those patients who attained culture negative status by the end of treatment, and had culture conversion to positive status with the **same** *Mycobacterium tuberculosis (M.tb)* strain or after the end of treatment in those patients who attained culture negative status by the end of treatment and were withdrawn for clinical (TB) reasons including being re-treated (or changing from protocol treatment) for TB. Details are given in Appendix 2.

Reinfection is defined as failing to maintain culture negative status or being declared an unfavourable outcome (including being withdrawn for clinical (TB) reasons including being re-treated or changing from protocol treatment for TB) after the end of treatment in those patients who attained culture negative status by the end of treatment and had culture conversion to positive status with a *Mycobacterium tuberculosis (M.tb)* strain that is **different** from the infecting strain at baseline. If reinfection cannot be distinguished from relapse, the patient will be assumed to have relapsed. A single positive sample will be sufficient for strain typing to compare to baseline. Full details are in Appendix 2.

The ***treatment period*** is defined as 6 months (total of 26 weeks) of the BPa therapy (linezolid may be stopped early) plus any days made up for interrupted doses of BPa therapy (or 9 months in those remaining culture positive at month 4 and who are not withdrawn).

The ***follow-up period*** is defined as the period after the last treatment dose to the end of follow-up.

3.2. Inability to produce sputum

In general, inability to produce sputum is treated as being equivalent to having a negative (favourable) culture result. This includes the rare situation where a patient who never achieves culture negative status due to inability to produce sputum, but completes follow-up without clinical or microbiological evidence of relapse. Such a patient will be considered to have a favourable outcome.

3.3. Isolated positive cultures

It is known that occasionally patients produce sputum samples that are "isolated positives", that is a positive culture preceded by a series of negative cultures and followed thereafter by at least 2 negative cultures without an intervening positive result. This phenomenon may be the result of a sealed cavity breaking down or laboratory contamination and does not in itself signify that the patient is relapsing. In the event of a single positive culture result occurring in a patient who has previously been classified as having culture negative status (in the absence of any retreatment), the patient will not be classified as a recurrence unless a second positive culture result is obtained at a separate visit (at least 7 days apart) without an intervening negative culture or unless the patient is lost to follow up or completes the study (and is unable to be brought back) before two negative cultures are obtained. As there is a higher incidence of positives with liquid culture and sometimes even serial "isolated positives" the clinical condition of the patient will also be considered in deciding whether the patient has an unfavourable outcome and re-treatment is indicated.

To expand a bit, most of the experience with isolated positives has been with solid culture. Because liquid culture is more sensitive, it is possible that more than one isolated positive may occasionally occur. Therefore, the clinical condition of the patient will also be considered when deciding whether re-treatment is indicated and in determining the outcome. For example, if a patient after being culture negative has two positive cultures in a row, but is deemed to be doing well clinically, the investigator may choose to leave the patient untreated on clinical grounds. In such a case, so long as two consecutive negative cultures are eventually obtained in the absence of treatment, the patient will not be classified as an unfavourable outcome.

3.4. Timing of events

In all analyses, visit date rather than day or week number will be used to define the timing of events. The 6-month regimen will be taken as a total of 26 weeks, i.e. 182 dosing days, from the start of therapy, after accounting for any treatment interruptions. For those who extend treatment to 9 months this will be 39 weeks (273 days) from start of therapy, again after accounting for any treatment interruptions.

For the end of treatment visit (months 6/9), a ±1-week window will be applied (as per the protocol). For the 3-monthly visits after the end of therapy, a window of ±2 weeks will be applied (as per the protocol). Additional programming will be required for cases where end of treatment date is not clearly recorded.

In the event that more than 10% of patients are culture positive at 4 months and have their treatment extended for a further 3 months, the primary endpoint analysis will be defined as 15 months from start of therapy for all patients. In this case the visit date for the endpoint analysis will be chosen as the one closest to 65 weeks (26+39) from start of therapy (unless patient is declared unfavourable before this date).

4. Analysis populations

Patients who are never culture positive during the baseline period, (screening through week 4) but are eligible based on documented M.tb by culture or molecular test within 3 months prior to screening will be included in all analysis populations.

The analysis populations for efficacy analyses are:

- The **Intent to treat** (**ITT**) population is defined as all patients excluding late screening failures (see 4.1)
- The **Modified intent to treat** (**MITT**) population is defined as the ITT population with extra exclusions (See 4.2)
- The **Per-protocol (PP)** population is defined as the MITT population with extra exclusions (see 4.3)

Exclusions from these populations will be reported as “unassessable” status and are described below.

4.1. Exclusions from ITT analysis (late screening failures)

1. Patients withdrawn from treatment because they were found to be ineligible (late exclusions from the study), based on data collected prior to enrollment, including patients who do not have documented evidence of M.tb within 3 months of screening. Note, reinfections will not be excluded from the ITT population.

4.2. Additional exclusions from MITT analysis

1. Patients who, having completed treatment, are lost to follow-up or withdrawn from the study, their last status being culture negative and their last positive culture result (“isolated positive culture”) followed by at least two negative culture results at different visits (at least 7 days apart, without an intervening positive culture)

2. Women who become pregnant during treatment and stop their allocated treatment

3. Patients who died during treatment from violent or accidental cause (e.g. road traffic accident). N.B.: This does not include death from suicide, which will be considered an unfavourable outcome.

4. Patients who died during follow-up (after the end of treatment) with no evidence of failure or relapse of their TB, their last status being culture negative and their last positive culture result (“isolated positive culture”) followed by at least two negative culture results at different visits (at least 7 days apart), and who have not already been classified as unfavourable.

5. Patients who, after being classified as having culture negative status, are re-infected with a new strain different from that with which they were originally infected. Reinfection will be defined specifically as a patient infected with a strain that is genetically different from the initial strain (see Appendix 2).

6. Patients who are able to produce sputum at their primary endpoint visit, whose sputum samples are all contaminated or missing, who cannot be brought back for repeat cultures, provided they have not already been classified as unfavourable and provided their last positive culture was followed by at least two negative cultures. N.B.: This does not apply to patients who are unable to produce sputum at 6 months after end of treatment, or to patients who are able to be brought back subsequently and produce negative cultures.

Patients in categories 1-6 above who had already been classified as having an unfavourable outcome will not be excluded.

4.3. Additional exclusions from PP analysis

1. Patients lost to follow-up or withdrawn before the end of treatment due to reasons other than treatment failure, unless they have already been classified as having an unfavourable outcome.

2. Patients whose treatment was modified or extended (beyond what is permitted in the protocol) for reasons (e.g. an adverse drug reaction) other than an unfavourable therapeutic response to treatment, unless they have already been classified as having an unfavourable outcome.

3. Patients not meeting the definition of having received an adequate amount of their allocated study regimen (see section 4.5 for definition), provided this is not due to unfavourable outcome.

4. Patients who are classified as "major protocol deviations for analysis" (see below), unless they have already been classified as having an unfavourable outcome on the basis of data obtained prior to the protocol deviation.

A list of all protocol deviations will be compiled throughout the course of the study.

A ***Major Protocol Deviation for Analysis*** is defined as a serious protocol deviation which is likely to affect to a significant degree the scientific value of the trial. These patients will be included in the ITT and MITT analyses, but not in the Per Protocol analysis. A list of all major protocol deviations for analysis will be approved by the study Coordinating Investigator before database lock.

A ***Minor Protocol Deviation*** is defined as a technical deviation which does not result in harm to the trial subjects or significantly affect the scientific value of the reported results of the trial.

4.4. Lost to Follow-up or Early Withdrawal

Lost to Follow-up or Early Withdrawals *before* the end of the treatment (month 6 or 9) are considered as unfavourable outcomes for ITT and MITT. However, these patients will be excluded from the Per Protocol analysis. The MITT and Per Protocol analyses will consider Lost to Follow-up *after* end of treatment as unassessable unless at the time of default from follow-up the patient a) was already classified as having an unfavourable outcome, b) did not have culture negative status, or c) had a positive culture result ("isolated positive culture") not followed by at least two negative culture results at different visits (at least 7 days apart), in which cases the patient will be classified as having an unfavourable outcome. We believe this is the most appropriate approach for the primary analysis because together with the non-tuberculosis deaths, this group is likely to considerably out-number the bacteriological failures and relapses. These patients will be considered as having an unfavourable outcome in the ITT analysis.

There is a clear precedent for this analytic approach in other TB trials, and these trials also provide examples of why the inclusion of the losses to follow-up as unfavourable greatly affects the results.

Data from the Priftin trial which led to accelerated approval of rifapentine and a trial conducted by the International Union Against TB & Lung Disease (IUATLD) in African and Asian sites illustrate the problems associated with classifying all losses to follow-up and deaths as having an unfavourable outcome.

In the Priftin trial bacteriological relapses occurred in 5% of patients on the rifampicin based regimen compared to 11% on the rifapentine based regimen. Approximately one third of patients were lost to follow-up and when this group combined with patients unassessable for other reasons were added to the bacteriological failures, the rates increased to 53% and 57% respectively. The true bacteriological relapses were greatly outnumbered by these other groups. At the time of the licensing submission to the FDA it was recognised that because there were a substantial number of patients likely to be unassessable the main focus should be on the relapse rates. In the final statistical report the results were first reported excluding those unassessable and then assuming all losses had an unfavourable outcome and finally assuming all losses had a favourable outcome.

In the study conducted by the IUATLD the published failure/relapse rates 12 months after stopping treatment based on 1044 assessable patients were 4% for the control regimen and 10% and 14% in each of the experimental arms. If the 311 unassessable patients were considered to have an unfavourable outcome these rates would increase to 24%, 32% and 35% respectively. The 311 unassessable patients were not evenly distributed across the three trial arms. There were 42 deaths, of which 20 occurred in one of the experimental arms (the more efficacious of the two) and 11 in each of the other, a difference which was not considered to be due to the treatment, but due to chance. There were also imbalances among those without a bacteriological assessment (7 in one arm versus 19 and 22 in the other two arms) and in the distribution of losses to follow-up.

4.5. Definition of adequate treatment

The definition of adequate treatment sets a limit for the amount of treatment missed. Patients not taking the adequate amount of treatment by this definition will be excluded from the PP analysis.

Patients treated for 6 months with no treatment extension, to meet the definition of adequate treatment they must have taken at least 146 doses (80%) of their allocated 182 day (26 weeks) treatment regimen within 242 days of starting therapy (i.e. 26 weeks plus an allowable 60 day halt (including a maximum of 35 consecutive days) as per the protocol).

For patients who have their treatment extended to 9 months (39 weeks), to meet the definition of adequate treatment, they must have taken at least 219 doses (80%) within 333 days.

A dose is defined as taking the required daily dose of both pretomanid and bedaquiline.

4.6. Determining cause of death

A list of all ***TB-related*** and ***non-TB-related deaths*** will be generated and approved by a review committee of physicians not associated with the trial before database lock. Similarly, a list of violent or accidental deaths will be generated.

5. Baseline comparisons of key characteristics

The following baseline characteristics of patients will be summarised: age, gender, race, site, weight, height, BMI, smoking status, TB type (XDR /non-XDR), HIV status/CD4 count/on ARV, cavitation, initial bacterial load in sputum as indicated by baseline Time to Positivity (TTP) result from MGIT, drug resistance.

6. Classification of primary endpoint status

Patients will be classified as having a favourable, unfavourable or unassessable status at 6 months after the end of therapy.

6.1.1. Favourable status (all analyses)

Patients with a negative culture status at 6 months from end of therapy who had not already been classified as having an unfavourable outcome, and whose last positive culture result ("isolated positive culture") was followed by at least two negative culture results.

6.1.2. Unfavourable status in ITT population

Patients in the ITT analysis population who do not have a favourable outcome at 6 months from end of therapy will be considered to have an unfavourable response in the ITT analysis.

6.1.3. Unfavourable status in MITT population

1. Patients not classified as having achieved or maintained culture negative status when last seen, or
2. Patients previously classified as having culture negative status who, following the end of treatment, have two positive cultures without an intervening negative culture, (however, see Section 3.3 for an exception), or
3. Patients who had a positive culture not followed by at least two negative cultures when last seen, or
4. Patients dying from any cause during treatment, except from violent or accidental cause (e.g. road traffic accident), not including suicide (i.e., suicide will be considered an unfavourable outcome) or
5. Patients definitely or possibly dying from TB related cause during the follow-up phase or
6. Patients requiring an extension of their treatment beyond that permitted by the protocol, a restart or a change of treatment for any reason except reinfection or pregnancy, or
7. Patients lost to follow up or withdrawn from the study before the end of treatment

6.1.4. Unfavourable status in PP population

1. Patients not classified as having achieved or maintained culture negative status when last seen, or
2. Patients previously classified as having culture negative status who, following the end of treatment, have two positive cultures without an intervening negative culture, (however, see Section 3.3 for an exception), or
3. Patients who had a positive culture not followed by at least two negative cultures when last seen, or
4. Patients dying from any cause during the treatment phase, except from violent or accidental cause (e.g. road traffic accident), not including suicide (i.e.., suicide will be considered an unfavourable outcome), or
5. Patients definitely or possibly dying from TB related cause during the follow-up phase, or
6. Patients requiring a restart or a change of treatment because of an unfavourable outcome with or without bacteriological confirmation, i.e. on bacteriological, radiographic or clinical grounds, unless due to reinfection with a new organism

7. Primary endpoint analysis

The MITT analyses will be considered primary. The proportion of assessable patients with a favourable and unfavourable outcome, with 95% confidence intervals, will be presented. For success, the lower bound of the 95% confidence interval for a favourable outcome should be above 50%. This MITT analysis is consistent with the TB literature over the past 50 years. **However, we recognize that for the purposes of the NDA, FDA and other regulatory agencies will consider the ITT analysis primary, where all patients who are not proven to have a favourable outcome will be classified as having an unfavourable outcome.**

8. Sensitivity analyses of primary endpoint analysis

In addition to analysing the primary endpoint data by ITT, MITT and PP, it is planned to conduct the following sensitivity analyses:

1. An analysis of the ITT, MITT and PP populations including only the XDR patients

2. An analysis of patients in the MITT and PP populations where reinfections are classified as unfavourable outcomes

3. An analysis of the MITT and PP populations treating all deaths as unfavourable

4. An analysis of the ITT, MITT and PP populations excluding patients who were never culture positive during the baseline period (screening through week 4), but were eligible based on documented M.tb by culture or molecular test within 3 months prior to screening.

9. Secondary efficacy analyses of primary endpoint

9.1. Time to event unfavorable outcome analysis

Time to an unfavourable outcome will be analysed with Kaplan Meier plots and Cox's proportional-hazards regressions analysis. These analyses will be performed according to ITT, MITT and PP endpoint classifications. Time to event will be calculated in days from the date of enrolment up to the first date associated with the reason for unfavourable status or (if favourable) the date of the 6 month after end of therapy visit.

10. Secondary efficacy endpoints

10.1. Incidence of bacteriologic failure or relapse at 24 months after the end of treatment

Efficacy analyses as described for the primary endpoint will be repeated for the 24 month after the end of treatment endpoint as a confirmatory analysis.

10.2 Time to sputum culture conversion to negative status

Time to culture negative status (first of two negative cultures without an intervening positive culture) will be analysed using survival analysis techniques, Kaplan Meier plots and Cox proportional hazard regression.

10.3 Culture conversion status at 4, 6, 8, 12 and 16 weeks

Patients will be classified as being culture positive, culture negative, dead or unassessable at 4, 6, 8, 12 and 16 weeks. Every effort will be made to obtain a sputum sample from all patients, but it is recognised that some patients may not have produced any sputum in the preceding week and may be unable to do so when requested. Patients who cannot produce sputum will be classified as being culture negative at that time point. The proportion culture negative will be those classified as being culture negative divided by the total considered culture negative, culture positive or have died. This proportion will be estimated from the Kaplan Meier estimates from the time to culture conversion to negative status analysis.

10.4 TB symptoms

Each TB symptom will be summarised by n (%): none (0), mild (1), moderate (2), severe (3) at each visit collected as per the protocol: baseline, week 8, end of treatment, 6 and 24 months from end of treatment.

In addition baseline and change from baseline score at each time point listed above for each symptom and for total symptom score will be summarised by mean, median, IQR and range.

10.5 Patient reported health status

Patient reported health status is measured by the 5 domains of EQ5D. These will be summarised at baseline, week 8, end of treatment, 6 and 24 months from end of treatment by randomised group and change from baseline at each follow-up assessment by mean, median, IQR and range by randomised group.

10.6 Weight

Baseline weight and change from baseline weight throughout treatment and at 6 and 24 months after the end of therapy will be summarised by mean, median, IQR and range.

11 Pharmacokinetics-Pharmacodynamics (PK-PD)analyses

Details of the PK parameter estimation and analysis are detailed in a separate PK SAP. PK-PD analyses will be described in a separate PK-PD SAP.

12 Sub-group analyses

To assess consistency of results, exploratory sub-group analyses of the primary endpoint on the MITT analysis population will be considered. For example, depending on numbers consideration will be given to subgroup analyses by: age; gender; race; smoking status; TB type (XDR vs not) HIV status/CD4 count; cavitation, initial bacterial load in sputum as indicated by baseline TTP result from MGIT; ARV taken or not during the treatment period.

13 Reasons for treatment failure as determined by the local PI

Reason(s) that led the site investigator to conclude that an individual patient failed treatment or relapsed will be classified as a) bacteriology alone, b) clinical deterioration alone, c) radiological deterioration alone, d) bacteriology plus clinical deterioration, e) bacteriology plus radiological deterioration, f) clinical deterioration plus radiological deterioration, or g) bacteriology plus clinical deterioration plus radiological deterioration. These classifications will be tabulated and compared to outcomes derived from the algorithm described in section 6.

14 Further exploratory analyses

The exploratory efficacy endpoints and analyses are as follows:

- Evaluate whether any of the secondary endpoints predicts relapse-free cure.

- Correlation of time over mitochondrial protein synthesis inhibition (MPS50) with linezolid toxicity (the MPS50 value will be an assumed value from literature).

15 APPENDICES

15.1 Appendix 1 Algorithm for Interpretation of Positive MGIT Results

15.2 Figure A1. Algorithm for reporting MGIT final results

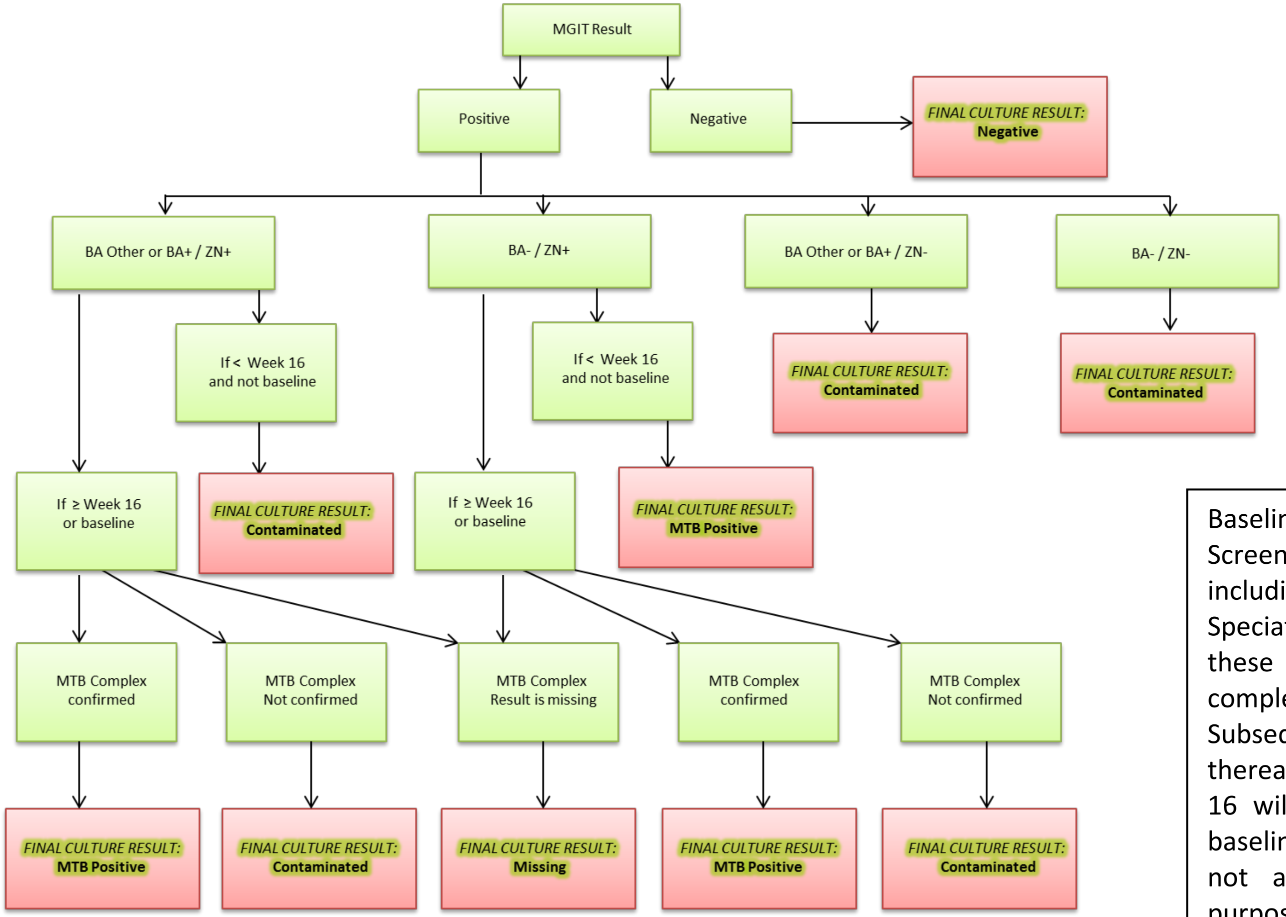

Baseline: Any visit from Screening up to (and including) week 4. Speciation will be done for these samples until a MTB complex is confirmed. Subsequent samples thereafter and before week 16 will be treated as post baseline (i.e. “<week 16 and not a baseline”) for the purpose of this algorithm.

Table A2. Derived MGIT results per visit

**Derived sample Culture Result (Visit X)**	**Derived Sample Culture Result (Visit X)**	**Final Derived Result for Visit X**
Positive	Missing/Negative/Contaminated	Positive
Negative	Missing/Contaminated	Negative
Contaminated	Missing/Contaminated	Contaminated

15.2 Appendix 2 Interpretation of Relapse and Re-infection using Whole Genome Sequence (WGS) data

The purpose of the WGS analysis is to determine if the two M. tuberculosis strains from a given patient (positive culture at baseline and at or after the end of treatment) can be considered the **same** (treatment failure/bacteriologic failure or relapse/bacteriological relapse), or **different** (re-infection/bacteriological re-infection). To do this, WGS two M. tuberculosis strains are compared, the number of SNPs/variants determined, and the criteria outlined below followed. These cut offs have been determined from previously published reports (REMoxTB and RIFAQUIN trials) that show a clear genetic distinction between relapse and re-infection cases of M.tb infection.

- ≤12 SNPs different = Relapse
- ≥100 SNPs different = Reinfection
- >12 and <100 SNPs different = Indeterminate. These results will be reviewed on case by case basis and are likely to be rare. Additional sequence analysis may be performed and/or additional samples may need to be tested. Any additional investigations will be documented on the 'WGS Indeterminate Proforma' which also includes the final conclusion of 'relapse' or re-infection' based on this further review. A patient will be considered a relapse unless there is sufficient evidence to support a classification of re-infection.

SAFETY STATISTICAL ANALYSIS PLAN

FINAL AND INTERIM ANALYSES

NiX-TB-(B-L-Pa)

A PHASE 3 OPEN-LABEL TRIAL ASSESSING THE SAFETY AND EFFICACY OF BEDAQUILINE PLUS PRETOMANID PLUS LINEZOLID IN SUBJECTS WITH PULMONARY INFECTION OF EITHER EXTENSIVELY DRUG-RESISTANT TUBERCULOSIS (XDR-TB) OR TREATMENT INTOLERANT/NON-RESPONSIVE MULTI-DRUG RESISTANT TUBERCULOSIS (MDR-TB)

AUTHOR: Louise van Aswegen

VERSION NUMBER AND DATE: Version 1.0, 06JUL2017

STATISTICAL ANALYSIS PLAN SIGNATURE PAGE

	Name	Signature	Date
**Author:**	Louise van Aswegen		
**Position:**	Senior Biostatistician		
**Company:**	QuintilesIMS, South Africa		

The signatures below indicate review and approval of the proposed final and interim safety analyses and presentation of data as planned for protocol NiX-TB-(B-L-Pa) Version 1.0, dated 21 April 2014, and protocol amendments 01, dated 18 March 2015 (incorporated into the working protocol Version 2.0, dated 18 March 2015) and 02, dated 22 January 2016 (incorporated into the working protocol Version 3.0, dated 22 January 2016).

This version of the SAP was approved by the undersigned.

**Approved By:**	Daniel Everitt		
**Position:**	Vice President and Senior Medical Officer		
**Company:**	Global Alliance for TB Drug Development		
**Approved By:**	Joanna Moreira		
**Position:**	Clinical Manager, Clinical Operations		
**Company:**	Global Alliance for TB Drug Development		

STATISTICAL ANALYSIS PLAN SIGNATURE PAGE

	Name	Signature	Date
**Author:**	Louise van Aswegen		13JUL2017
**Position:**	Senior Biostatistician		
**Company:**	QuintilesIMS, South Africa		

The signatures below indicate review and approval of the proposed final and interim safety analyses and presentation of data as planned for protocol NiX-TB-(B-L-Pa) Version 1.0, dated 21 April 2014, and protocol amendments 01, dated 18 March 2015 (incorporated into the working protocol Version 2.0, dated 18 March 2015) and 02, dated 22 January 2016 (incorporated into the working protocol Version 3.0, dated 22 January 2016).

This version of the SAP was approved by the undersigned.

**Approved By:**	Daniel Everitt		11 July 2017
**Position:**	Vice President and Senior Medical Officer		
**Company:**	Global Alliance for TB Drug Development		
**Approved By:**	Joanna Moreira		12 July 2017
**Position:**	Clinical Manager, Clinical Operations		
**Company:**	Global Alliance for TB Drug Development		

MODIFICATION HISTORY

Unique Identifier for this Version	Date of Document Version	Author	Significant Changes from Previous Authorized Version
1.0	06July2017	Louise van Aswegen	Not applicable. First authorized version.

TABLE OF CONTENTS

Page:

Statistical Analysis Plan Signature Page ... 2
Modification History ... 3
Table of Contents ... 5
1. Introduction ... 8
2. Study Objectives ... 8
3. Study Design ... 9
3.1. General Description ... 9
3.2. Schedule of Events ... 11
3.3. Changes/Clarifications to Analysis from Protocol ... 11
4. Planned Analyses ... 13
4.1. Data Safety and Monitoring Committee Analysis ... 14
4.2. Interim Analyses ... 14
4.3. Final Analysis ... 15
5. Analysis Populations ... 16
5.1. Re-screened Subjects ... 17
5.2. All Enrolled Analysis Population ... 17
5.3. Intent to Treat (ITT) Analysis Population ... 17
5.4. Safety Analysis Population ... 17
6. General Considerations ... 18
6.1. Reference Start Date and Study Day ... 18
6.2. Baseline ... 18
6.3. Treatment-emergent Incidence ... 19
6.4. Unscheduled Visits ... 19
6.5. End of Treatment/End of Study ... 20
6.6. Windowing Conventions (only applicable to the first ia) ... 20
6.7. Statistical Tests ... 22
6.8. Common Calculations ... 24
6.9. Software Version ... 24
7. Statistical Considerations ... 24
7.1. Multicenter Studies ... 24
7.2. Multiple Comparisons/Multiplicity ... 24
7.3. Missing Data ... 25
7.4. Examination of Subgroups ... 25
8. Output Presentations ... 25
9. Disposition and Premature Discontinuation ... 26
9.1. Analysis ... 26

9.2. Derivations 27
10. protocol Deviations 27
11. Demographic Characteristics 28
11.1. Derivations 29
12. Baseline Characteristics 29
13. Disease History 29
13.1. Derivations 30
14. Chest X-Ray at Screening 31
15. Ophthalmology History 31
16. Drug Resistance History 32
17. Baseline Drug Susceptibility 33
18. Concomitant Medications 33
19. Concomitant Procedures 34
20. Medical History 35
21. Study Drug Exposure 35
21.1. Derivations 38
22. Efficacy Analysis (only applicable to the first ia) 39
22.1. Primary Efficacy 39
22.1.1. Derivations 39
22.1.2. Definitions 42
22.1.3. Primary Efficacy Endpoint 43
22.1.4. Analysis Methods 43
22.2. Other Secondary Efficacy 45
22.2.1. Other Secondary Efficacy Endpoints and Definitions 45
22.2.2. Tuberculosis Symptoms 45
22.2.3. Subject Reported Health Status: EQ-5D-5L 46
22.2.4. Weight 49
23. Safety Outcomes 50
23.1. Adverse Events 50
23.1.1. Severity 51
23.1.2. Relationship 51
23.1.3. Action Taken with Study Drug 52
23.1.4. Adverse Events (AEs) Leading to Death 53
23.1.5. Serious Adverse Events (AEs) 53
23.1.6. Adverse Events (AEs) Leading to Discontinuation of Study Drug 53
23.1.7. All Treatment-emergent AEs (TEAEs) 54
23.1.8. Adverse Events (AEs) of Special Interest 54
23.1.1. Liver-Related AEs 56
23.1.2. Derivations 56
23.2. Laboratory Tests 56

23.2.1. Division of Microbiology and Infectious Disease (DMID) Grading for Laboratory Data . 57
23.2.2. Liver-Related Abnormalities and Evaluation if Drug-Induced Serious Hepatotoxicity ... 58
23.2.3. Derivations ... 59
23.3. Vital Signs ... 59
23.3.1. Notable Abnormalities ... 60
23.4. Electrocardiogram Parameters ... 61
23.4.1. Electrocardiogram Prolongation ... 61
23.5. Ophthalmological Examination ... 62
23.6. Slit Lamp Examination ... 63
23.7. Peripheral Neuropathy ... 64
23.7.1. Derivations ... 65
24. List of References ... 67
APPENDIX 1. Programming Conventions for Tables, By-subject Data Listings and Figures ... 68
APPENDIX 2. Partial Adverse Event Date Conventions ... 72
APPENDIX 3. Partial Medications Date Conventions ... 73
APPENDIX 4. Laboratory Tests ... 74
Appendix 5: Division of Microbiology and Infectious Disease (DMID) Laboratory Tests Toxicity ... 76
Appendix 6: Vital Signs Notable Abnormalities ... 80

1. INTRODUCTION

This document describes the rules and conventions that will be used in the planned presentation and analysis of safety and efficacy data performed by QuintilesIMS according to protocol NiX-TB-(B-L-Pa) for the final and the interim analyses (IA) as set out in the relevant versions of the output templates [20161411 Nix-TB-(B-L-Pa) Interim Analysis Output Templates Version 1.0 and 20170630 Nix-TB-(B-L-Pa) Final and Interim Analysis Output Templates Version 0.1]. The pharmacokinetic (PK) analysis of this study will be described in separate statistical analysis plans (SAPs). From the second IA onwards, the fifth data safety monitoring committee (DSMC) onwards and the final analysis, Medical Research Council (MRC) Clinical Trials Unit (CTU) at University College London (UCL) (MRC CTU at UCL) will be responsible for the efficacy, which will be described in a separate SAP as set up by MRC CTU at UCL. It describes the data that will be summarized and analyzed, including specifics of the statistical analyses that will be performed.

QuintilesIMS will be responsible for the DSMC analyses including safety and efficacy data for the DSMC meetings 1, 2, 3 and 4. For all subsequent DSMC analyses, QuintilesIMS will only be responsible for the safety data and MRC CTU at UCL will be responsible for the efficacy data. For the first IA, QuintilesIMS will be responsible for the safety and efficacy data. For the subsequent IAs and the final analysis, QuintilesIMS will only be responsible for the safety data and MRC CTU at UCL will be responsible for the efficacy data.

This SAP is based on protocol Version 1.0, dated 21 April 2014, and protocol amendments 01, dated 18 March 2015 (incorporated into the working protocol Version 2.0, dated 18 March 2015) and 02, dated 22 January 2016 (incorporated into the working protocol Version 3.0 dated 22 January 2016).

2. STUDY OBJECTIVES

The objective of this study is to evaluate the efficacy, safety, tolerability and PK of Bedaquiline (B) plus Pretomanid (Pa) plus Linezolid (L) (B-L-Pa) after 6 months of treatment (with an option to treat for 9 months in subjects who are still culture positive at Month 4) in subjects with either pulmonary extensive drug-resistant (XDR) tuberculosis (TB), treatment intolerant or non-responsive multi-drug resistant (MDR) TB (MDR-TB).

The primary endpoint of this study is:

- Incidence of bacteriologic failure or relapse or clinical failure through follow up until 6 months after the End of Treatment.

The secondary endpoints of this study are:

- Incidence of bacteriologic failure or relapse or clinical failure through follow up until 24 months after the End of Treatment as a confirmatory analysis.
- Time to sputum culture conversion to negative status through the treatment period.
- Proportion of subjects with sputum culture conversion to negative status at 4, 6, 8, 12, 16 and 26 or 39 weeks.
- Linezolid dosing (actual) and efficacy will be explored.
- Change from Baseline TB symptoms.
- Change from Baseline in Patient Reported Health Status.
- Change from Baseline weight.

The exploratory endpoints and analyses of this study are:

- Evaluate whether any of the secondary endpoints predicts relapse free cure.
- Sub-analysis of populations by Human Immunodeficiency Virus (HIV) status and CD4 count.
- Correlation of Time over mitochondrial protein synthesis inhibition (MPS50) with Linezolid toxicity (the MPS50 value will be an assumed value from literature).

3. STUDY DESIGN

3.1. GENERAL DESCRIPTION

Up to 200 male and female subjects aged 14 years and older, with confirmed sputum positive for *Mycobacterium tuberculosis* (M.tb.) in culture pulmonary XDR-TB, or with pulmonary MDR-TB with a documented intolerability or non-response to the best treatment available for 6 months or more will be enrolled.

- After a maximum of 9 days for Screening, all subjects will receive 6 months of treatment. Subjects will have follow-up visits 1 and 2 months after treatment completion, and thereafter every 3 months, up to Month 24. If a subject is still culture positive by Month 4, they may have treatment extended to 9 months (with 24 months of follow-up) or be withdrawn from the study.
- Subjects who withdraw after ≤ 14 days of treatment should attend an early withdrawal visit. Subjects who withdraw after ≥ 15 days of treatment should return for an early withdrawal visit and follow-up visits at 3, 12 and 24 months after their last dose of treatment (to confirm survival status, occurrence of serious adverse event [SAEs] and resolution of TB symptoms).

A schematic of the study design is presented below.

Figure 1: Study Schematic

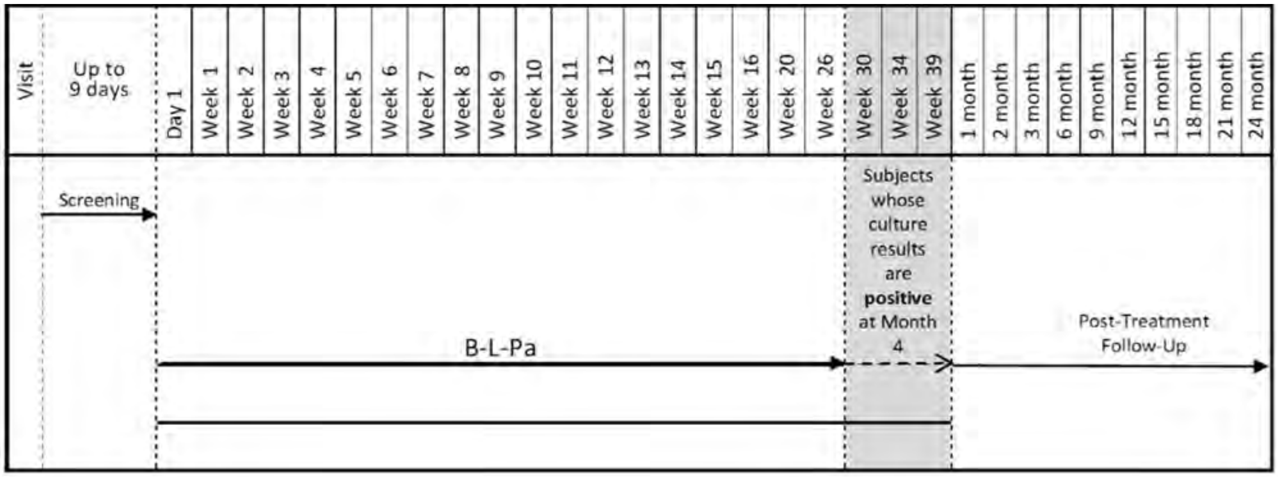

All subjects will receive B-L-Pa for the duration of treatment as follows:

Table 1: Study Drug Administration

Study Drug	Dose to be Administered Orally
Bedaquiline	400 mg once daily for Day 1 to Day 14 then<br>200 mg three times per week for remainder of treatment
Pretomanid	200 mg once daily
Linezolid	600 mg twice a day (BID) (Protocol Version 1 and 2)<br>1200 mg once a day (QD) (Protocol Version 3)<br>If adverse events develop, the Investigator can interrupt or reduce the dose to either 600 mg QD or 300 mg QD<br>If subjects have toxicity issues with Linezolid that would prohibit further treatment with that drug, they can remain on Bedaquiline and Pretomanid if they received the initial 1200 mg total daily dose of Linezolid for at least 4 consecutive weeks of treatment and they are smear negative or with trace/scanty results and judged to be clinically improving by the Investigator

3.2. Schedule of Events

The schedule of events can be found in Section 1.2: Trial Flow Chart of the protocol.

3.3. Changes/Clarifications to Analysis from Protocol

The definition of treatment-emergent adverse events (TEAEs) changed from:

- AEs which started or worsened on or after the first study drug administration until the last scheduled visit/assessment/measurement will be regarded as treatment-emergent.

to

- AEs which started or worsened on or after the first study drug administration up to 14 days after the last study drug administration.

For analysis of culture data (except for stable negative conversion) for DSMC 1 to 4 and IA 1, the derived result at Baseline will be considered positive for all subjects regardless of culture results derived per sample. This is due to the fact that the inclusion criteria stipulate that subjects with one of the following pulmonary TB conditions:

- XDR-TB with:
  - Documented culture positive (for M.tb.) results within 3 months prior to Screening or M.tb. confirmed in sputum based on molecular test within 3 months prior to or at Screening.
  - Historical documented resistance to Isoniazid, Rifamycins, a Fluoroquinolone and an injectable at any time.
- MDR-TB documented by culture positive results (M.tb.) within 3 months prior to Screening with documented non-response to treatment with the best available regimen for 6 months or more prior to enrollment who in the opinion of the Investigator have been adherent to treatment and will be adherent to study regimen.
- MDR-TB documented by culture positive (for M.tb.) results within 3 months prior to Screening who are unable to continue second line drug regimen due to a documented intolerance to:
  - PAS, Ethionamide, Aminoglycosides or Fluoroquinolones;
  - Current treatment not listed above that renders subject eligible for the study in the Investigator’s opinion.

The study protocol stipulates that the first IA will be conducted when the first 15 subjects reach Month 6 after completion of treatment. The first IA will however be conducted after the 15th subject has completed Month 6 visit OR prematurely discontinued study drug. The list of the 15 subjects will be provided by TB Alliance.

The following IAs will be cumulative including the next 15 subjects or more whom completed the Month 6 visit or prematurely discontinued the study drug as identified by TB Alliance.

4. Planned Analyses

The following formal analyses will be performed for this study:

**Analysis**	**Company Responsible**	**Details/Comments**	**Domains**
Data Safety and Monitoring Committee Analyses (DSMC)			
DSMC 1 to 4	QuintilesIMS	Safety and Efficacy	All
DSMC 5 onwards	QuintilesIMS	Safety	All applicable domains excluding efficacy
DSMC 5 onwards	MRC CTU at UCL	Efficacy	Culture Data<br>Tuberculosis Symptoms<br>Subject Reported Health Status: EQ-5D-5L<br>Weight
Interim Analyses (IA)			
IA 1	QuintilesIMS	Safety and Efficacy	
IA 2 onwards	QuintilesIMS	Safety	All applicable domains excluding efficacy
IA 2 onwards	MRC CTU at UCL	Efficacy	Culture Data<br>Tuberculosis Symptoms<br>Subject Reported Health Status: EQ-5D-5L<br>Weight
Final Analysis	QuintilesIMS	Safety	All applicable domains excluding efficacy
Final Analysis	MRC CTU at UCL	Efficacy	Culture Data<br>Tuberculosis Symptoms<br>Subject Reported Health Status: EQ-5D-5L<br>Weight

4.1. DATA SAFETY AND MONITORING COMMITTEE ANALYSIS

Interim safety and efficacy data will be reviewed by the DSMC as follows:

- At least every 6 months after the first subject is enrolled.
- *Ad hoc* meetings can be requested by TB Alliance or the DSMC based on rates of SAEs or if safety concerns arise during the study.

The DSMC analyses and output templates are described in a separate document [Nix-TB-(B-L-Pa) DSMC Output Template].

4.2. INTERIM ANALYSES

Each interim analysis will be performed for every 15 subjects completing/prematurely discontinuing study drug. The timing of the first interim analysis will be after the 15th subject has completed Month 6 visit. The list of the 15 subjects or more, will be provided by TB Alliance for each IA. Data will be presented cumulatively.

The first IA was planned following:

- QuintilesIMS Biostatistics (QIMS BIOS) review and acceptance of the Study Data Tabulation Model (SDTM) datasets received from eClinical Solutions (eCS).
- TB Alliance authorization of the IA output templates. TB Alliance confirmed via email that they were in agreement with the output templates to be used for the first IA.
- TB Alliance review of TB medications and AEs of special interest.
- TB Alliance review and authorization of the analysis populations.
- QuintilesIMS BIOS review of the database and subsequently providing data issues to Data Management via the Data Issues Log.

The second IA onwards is planned following:

- QuintilesIMS Biostatistics (QIMS BIOS) and MRC CTU at UCL review and acceptance of the Study Data Tabulation Model (SDTM) datasets received from eClinical Solutions (eCS).
- TB Alliance authorization of the final and interim analyses version of the SAP and output templates.
- TB Alliance review of TB medications and AEs of special interest.
- TB Alliance review and authorization of the analysis populations.
- QuintilesIMS BIOS review of the database and subsequently providing data issues to Data Management via the Data Issues Log.

The IA will be performed on a clean database:

- All outstanding data issues and queries resolved for the identified subjects and visits.
- All unresolvable data issues documented in the DHR from Data Management.
- All coding of medications, medical conditions, physical examination abnormalities and AEs completed.
- Reconciliation of the following data with electronic case report form (eCRF) data completed successfully:
    - Safety laboratory data.
    - Serious adverse events (SAEs).

4.3. Final Analysis

The final analysis will be performed after all subjects have completed Month 24 visit after the End of Treatment.

The final analysis is planned following:

- QuintilesIMS Biostatistics (QIMS BIOS) and MRC CTU at UCL review and acceptance of the SDTM datasets received eCS.
- TB Alliance authorization of the final and interim analyses version of the SAP and output templates.
- TB Alliance review of TB medications and AEs of special interest.
- TB Alliance review and authorization of the analysis populations.
- QuintilesIMS BIOS review of the database and subsequently data issues provided to Data Management via the Data Issues Log.
- QuintilesIMS BIOS review and authorization of DHR.

The final analysis will be performed on a clean database:

- All outstanding data issues and queries resolved.
- All unresolvable data issues documented in the DHR from Data Management.
- All coding of medications, medical conditions, physical examination abnormalities and AEs completed.
- Reconciliation of the following data with case report form (eCRF) data completed successfully:
    - Safety laboratory data.
    - Serious adverse events (SAEs).

5. Analysis Populations

Analysis populations established by QuintilesIMS for the IA and final analysis will be determined programmatically based on the definitions listed below.

The Intent to treat (ITT), modified ITT (MITT) and per-protocol (PP) analysis populations will be determined by MRC CTU at UCL for IA 2 onwards and the final analysis, and they will be providing the assignment on to QuintilesIMS. Refer to the efficacy SAP for these analysis population definitions.

5.1. Re-screened Subjects

Re-screened subjects will be identified by means of the re-screened flag in the (SDTM.SC) dataset (SDTM.SC.SCORRES = 'Y' when SDTM.SC.SCTESTCD = 'RESCREEN'). Subjects will only be accounted for once and therefore the records related to the original subject number for re-screened subjects will be excluded from all analysis.

5.2. All Enrolled Analysis Population

This analysis population will include all subjects who have provided informed consent.

5.3. Intent to Treat (ITT) Analysis Population

For the first IA, the Intent to Treat (ITT) analysis population comprised all subjects who were included in the All Enrolled analysis population that were assigned to study drug.

For subsequent IAs and the final analysis, refer to the efficacy SAP for details.

5.4. Safety Analysis Population

For the first IA the Safety analysis population was defined as all subjects included in the ITT analysis population and received at least one administration of study drug.

For all subsequent IAs and the final analysis, the Safety analysis population will include all subjects who receive at least one administration of study drug.

Therefore a subject will be programmatically included in the Safety analysis population if the subject has at least one date of study drug administration available in the SDTM data (SDTM.EX.EXSTDTC).

The Safety analysis population will be used as primary analysis set for all safety analysis.

The ITT and the Safety analysis populations were used for all analysis (safety and efficacy) of the first IA.

6. General Considerations

6.1. Reference Start Date and Study Day

Study day will be calculated relative to the reference start date which will be used to present relative start/stop days of assessments/events. The reference start date (Day 1) is defined as the earliest (minimum) date of first study drug administration of the three study drugs (B, Pa, L). The reference end date is defined as the latest (maximum) date of last study drug administration of the three drugs.

- If the date of assessment/event is on or after the reference start date then:
  - o Study day = (Date of assessment/event – Reference start date) + 1.
- If the date of assessment/event is prior to the reference start date then:
  - o Study day = (Date of assessment/event – Reference start date).

In the case where the assessment/event date is partial or missing, for which no imputation rules apply, study day, and any corresponding durations will be presented as missing in the by-subject data listings.

6.2. Baseline

Unless stated otherwise, Baseline is defined as the last available non-missing assessment (scheduled or unscheduled) prior to or on Day 1. In the case where the last available non-missing assessment and the reference start date coincide, the assessment will be considered pre-dose. For example, if vital signs or laboratory assessments fall on the date of first study drug administration and the time of the assessment is not available, the applicable assessment will be considered as Baseline. However, medications commencing on the date of first study drug administration will be considered post-baseline, i.e. concomitant medications. For AEs, the answer to the question 'Did the adverse event occur prior to first dose of study medication' will also be taken into consideration when determining if the event can be regarded as treatment-emergent or not.

For the culture data for IA 1, Baseline is defined as the last available non-missing assessment (scheduled or unscheduled) prior to or on Day 1 or any positive culture result between Screening and Week 4. For all analysis purposes (except for stable culture negative conversion), the derived

result at Baseline will be considered positive for all subjects regardless of culture results derived per sample.

For the Baseline drug susceptibility data, Baseline is defined as the worst available non-missing assessment (scheduled or unscheduled) prior to or on Day 1 or any resistant result between Screening and Week 4, according to the following hierarchies, per method and drug:

- Resistant, sensitive, susceptible, contaminated, indeterminate, missing

For analysis purposes, a separate Baseline visit will be presented for each subject, where applicable, in by-subject data listings.

Post-baseline is defined as any assessment (scheduled or unscheduled) after Baseline (per data domain as described above).

6.3. TREATMENT-EMERGENT INCIDENCE

A treatment-emergent incidence is defined as any event (TEAEs, liver-related abnormalities, QT interval prolongation) (scheduled or unscheduled) which started after the first study drug administration up to 14 days (gap period) after the last study drug administration.

A post-treatment incidence is defined as any event (AEs, liver-related abnormalities, QT interval prolongation) (scheduled or unscheduled) which started after 14 days (gap period) after the last study drug administration.

6.4. UNSCHEDULED VISITS

For all safety analyses, data recorded at the scheduled visit will be presented in by-visit summaries. Unscheduled assessments will not be included in by-visit summaries, but will be included in by-subject data listings. Unscheduled visits will also be considered for Baseline and End of Treatment/End of Study values as well as for determining treatment-emergent incidence. Unscheduled visits will be sorted in a chronological order within subject in the by-subject data listings.

6.5. END OF TREATMENT/END OF STUDY

End of Treatment is defined as the non-missing post-baseline assessment (scheduled or unscheduled) closest to the last study drug administration target date/day within the period following the first study drug administration up to and including 14 days after the last study drug administration. Where an assessment prior to the last study drug administration date and an assessment after the last study drug administration date are equidistant from the target date/day, the result prior to the last study drug administration date will be regarded as End of Treatment.

End of Study is defined as the last non-missing post-baseline assessment (scheduled or unscheduled) assigned in the study and will only be presented when applicable.

6.6. WINDOWING CONVENTIONS (ONLY APPLICABLE TO THE FIRST IA)

The derived culture result per visit is based on an early morning and a spot sputum sample taken on the applicable scheduled visit. If one of the two results is missing at the scheduled visit the following steps should be followed:

- Check whether there is an unscheduled assessment available before or after the applicable scheduled visit. If there is no unscheduled assessment then the single sputum sample will be used to derive the culture result per visit.
- If an unscheduled assessment prior to the applicable scheduled visit is available; check if the previous scheduled visit had both sputum samples (spot and early morning) available. If the unscheduled assessment occurred after the applicable scheduled visit; check if the next scheduled visit had both sputum samples available. If the previous/next (as applicable) scheduled visit had both sputum samples available and the unscheduled visit has the applicable missing sputum sample result available, the result should be used together with the sputum sample result to derive the culture result per visit at the scheduled visit by mapping the unscheduled visit to the applicable scheduled visit.
- If the previous/next (as applicable) scheduled visit does not have both sputum samples available, then the unscheduled assessment will be assigned to the applicable scheduled visit based on the target days in Table 2.
- Once these steps have been followed for all scheduled visits with missing sputum samples, it will be determined if a subject has all scheduled visits up to his/her study completion or

premature study discontinuation, where sputum sampling was to be performed per protocol.

- If a subject does not have all of the applicable scheduled visits, it will be checked if there are any unscheduled assessments that fall within the relevant target days for the missing scheduled visit. If this is the case, the unscheduled assessments will be mapped to the missing scheduled visit based on the target days in Table 2.

Table 2: Visit Target Days for Protocol-specified Sputum Sampling Visits

Visit Name	Target Visit Day	Start Day of Visit Window	Midpoint	End Day of Visit Window
Screening	Day -9 to 0	-9	Not applicable	0
Day 1	Day 1	1	1	1
Week 1	Day 7	2	7	11
Week 2	Day 14	12	14	21
Week 4	Day 28	22	28	35
Week 6	Day 42	36	42	49
Week 8	Day 56	50	56	70
Week 12	Day 84	71	84	98
Week 16	Day 112	99	112	126
Week 20	Day 140	127	140	161
Week 26 (subjects who completed 6 months of treatment)	Day 182	162	182	Up to 14 days after the last study drug administration
Week 26 (subjects who completed 9 months of treatment)	Day 182	162	182	196
Week 30 (subjects who completed 9 months of treatment)	Day 210	197	210	224
Week 34 (subjects who completed 9 months of treatment)	Day 238	225	238	256
Week 39 (subjects who completed 9 months of treatment)	Day 273	257	273	Up to 14 days after the last study drug administration
End of Treatment	Non-missing post-baseline assessment (scheduled or unscheduled) closest to the last study drug administration target date/day within the period following the first study drug			

Visit Name	Target Visit Day	Start Day of Visit Window	Midpoint	End Day of Visit Window
	administration up to and including 14 days after the last study drug administration.			
Month 1	Day 30	1	30	46
Month 2	Day 61	47	61	76
Month 3	Day 91	77	91	137
Month 6	Day 183	138	183	229
Month 9	Day 274	230	274	320
Month 12	Day 365	321	364	411
Month 15	Day 457	412	457	503
Month 18	Day 548	504	548	594
Month 21	Day 639	595	639	685
Month 24	Day 731	685	731	∞
End of Study	Last non-missing post-baseline assessment (scheduled or unscheduled) assigned to the study.			

For scheduled visits, multiple records may exist for a particular sputum sample (early morning or spot). A worst-case approach will be used to determine the culture result per sample, based on the following hierarchy:

- Positive.
- Negative.
- Contaminated.

6.7. STATISTICAL TESTS

Unless otherwise specified:

- The default summary statistics for quantitative variables will be as follows:
  - Number of subjects in each category (n).
  - Mean.
  - Standard deviation (SD).
  - Median.
  - Minimum.
  - Maximum.

- The default summary statistics for qualitative variables will be as follows:
  - Number of subjects in each category (n).
  - The percentages of subjects in each category (%) can be presented relative to either one of the following:
    - The total number of subjects in the relevant analysis population.
    - The total number of subjects in the relevant analysis population, with assessments available (observed cases).
  - In the event of missing assessments, a 'Missing' category showing the number of subjects with missing assessments will be presented. Percentage of subjects with missing data will not be presented.

Univariate statistics:

- Statistics should be presented in the same order across tables (i.e. n, mean, SD, minimum, median and maximum).
- If the original data has N decimal places, then the summary statistics should have the following decimal places:
  - Minimum, maximum: N.
  - Mean and median: N + 1.
  - SD: N + 2.

Frequencies and percentages (n and %):

- Percent values should be reported inside parentheses, with one space between the count and the left parenthesis of the percentage. Parentheses should be justified to accept a maximum of 100.0 as a value and padded with blank space if the percent is less than 100.0. An example is given below:
  - 77 (100.0)
  - 50 (64.9)

Confidence intervals (CIs):

- CIs should be presented with one additional decimal place as that of the raw data, and SDs and SEs with two additional decimal places as that of the raw data.

- CIs should be justified so that parentheses displayed on consecutive lines of a table 'line up'.

P-values:

- P-values should be reported to four decimal places.

Ratios:

- Ratios should be reported with one additional decimal place as that of the raw data.

6.8. Common Calculations

For quantitative assessments, the change from Baseline will be calculated as:

- Change from Baseline at Week x/Month y= (Result at Week x/Month y– Baseline).

6.9. Software Version

All analyses will be conducted using SAS® Version 9.4.

7. Statistical Considerations

7.1. Multicenter Studies

This study will be conducted by multiple Investigators at multiple study centres. All centres will be pooled for analysis purposes.

7.2. Multiple Comparisons/Multiplicity

Not applicable.

7.3. MISSING DATA

For the handling of partial dates for AEs refer to Partial Adverse Event Date Conventions of this SAP.

For the handling of partial dates for medications refer to APPENDIX 3Partial Adverse Event Date Conventions of this SAP.

7.4. EXAMINATION OF SUBGROUPS

All outputs will be presented by current TB diagnosis at Baseline.

- Current TB diagnosis at Baseline:
  - XDR.
  - MDR (this includes MDR-TB treatment non-responsive and MDR-TB treatment intolerant).
  - Total (XDR and MDR).

8. OUTPUT PRESENTATIONS

Appendix 1: Programming Conventions for Outputs contains conventions for presentation of data in TLFs.

The output templates provided together with this SAP describe the presentations for the final and IA analyses and therefore the format and content of the TLFs to be provided by QuintilesIMS Biostatistics.

Note that verbatim terms, specifications (for example the reason a specific assessment was not done) and all variables in the TLFs containing the suffix (eCRF) contain verbatim text that may include spelling mistakes. Verbatim text will be presented in the by-subject data listings 'as is' and no manual 'hard-coding' correction of such data will be made.

The following concatenated variable will be presented in all by-subject data listings:

- Subject number, Baseline HIV status and TB type, separated out with a '/'. For example: 02-9003-003/+/XDR.

9. Disposition and Premature Discontinuation

9.1. Analysis

All subjects who provided informed consent will be accounted for in the study. Data as recorded on the Subject Disposition eCRF will be presented in a table (not applicable for the first IA analysis) and a by-subject data listing for the All Enrolled (final analysis)/ITT analysis population (IA analysis). Accounting for the key study milestones the following will be presented in the by-subject data listing:

- Date of informed consent relative to overall reference start date.
- Reason for screening failure (only applicable for the final analysis).
- Date of first study drug administration (overall reference start date) and last study drug administration (overall reference stop date).
- Treatment completion/discontinuation and the relevant primary reason for treatment discontinuation.
- Month 6 completion/discontinuation and the relevant primary reason for discontinuation.
- Month 24 completion/discontinuation and the relevant primary reason for discontinuation.
- Date of death relative to overall reference start date as well as the following information related to the death: Reason for death, relationship to TB and if the death was violent or accidental.

Subject disposition will be presented as a flowchart detailing:

- Subjects enrolled (who signed informed consent).
- Screening failures (not applicable for the IA analysis).
- Subjects in the ITT analysis population.
- Subjects who received study drug (Safety analysis population).
- Subjects identified for the IA (not applicable for the final analysis).
- Subjects in the MITT analysis population (not applicable for the first IA analysis).
- Subjects in the PP analysis population (not applicable for the first IA analysis).
- Subjects who discontinued treatment.
- Subjects who completed treatment.

- Subjects who discontinued prior to and including the Month 6 follow-up visit.
- Subjects who completed Month 6 follow-up.
- Subjects who discontinued prior to and including the Month 24 follow-up visit.
- Subjects who completed Month 24 follow-up.

A subject's treatment, Month 6 and Month 24 status (completion/discontinuation) and screening failures will be based on the Subject Disposition eCRF as recorded by the Investigator. Subjects who prematurely discontinue study participation prior or at the End of Treatment will not be regarded for the completion status at the Month 6 follow-up visit. Subjects who prematurely discontinue study participation prior or at the Month 6 follow-up visit will not be regarded for the completion status at the Month 24 follow-up visit.

Whether a subject is included in the All Enrolled analysis population and/or the Safety analysis population will be determined programmatically (refer to Section 5 Analysis Populations). The number of subjects identified for the ITT, mITT and PP analysis populations will be obtained from MRC CTU at UCL.

9.2. DERIVATIONS

Difference between first and last study drug administration (months):

- Difference (months) = ([Date of last study drug administration – date of first stuy drug administration] + 1)/(365.25/12).

10. PROTOCOL DEVIATIONS

All major protocol deviations data as recorded on the external EXCEL sheet provided by TB Alliance will be presented in a summary table for the ITT analysis population and in a by-subject data listing for the All Enrolled analysis population (final analysis)/ITT analysis population (IA analysis).

The following information will be presented in the by-subject data listing:

- Date and study day deviation noted.
- Deviation type.
    - o Informed consent.

  - Eligibility and entry criteria.
  - Withdrawal.
  - IMP administration.
  - Concomitant treatment.
  - Procedural (physical examinations/vital signs/PK/laboratory/cardiology/ophthalmology etc.).
  - Visit schedule.
  - Adverse event/specific toxicity/serious adverse event.
  - Source documentation.
  - Regulatory/ethics.
  - Administration criteria (outside of data issues).
  - Other.
- Deviation specified.

11. DEMOGRAPHIC CHARACTERISTICS

Demographic characteristics as recorded on the Demography eCRF will be presented.

With reference to Section 6 General Considerations and Section 7 Statistical Considerations of this SAP, the following data will be summarized:

- Age (years): Calculated relative to Screening.
- Gender (including childbearing potential for female subjects). Percentage (%) of childbearing potential status will be calculated relative to the total number of female subjects with data available.
  - Male.
  - Female.
- Race:
  - Asian.
  - Black or African American.
  - Native Hawaiian or other Pacific Islander.
  - White.
  - Mixed race.
  - Other.

The following will also be presented in the by-subject data listing:

- Country.

11.1. Derivations

Age (years): Calculated relative to Screening using the following SAS® code:

- Age (years) = int((intck('month', <date of birth>, <date of Screening>) - (day(<date of Screening>) < day(<date of birth>)))/12). Date of Screening to be obtained from SDTM.SV.SVSTDTC using the relevant record where SDTM.SV.VISIT = 'SCREENING'. Date of birth to be obtained from SDTM.DM.BRTHDTC.

12. Baseline Characteristics

Baseline characteristics as recorded on the Vital Signs eCRF, the Safety Laboratory Sample Collection eCRF, the Karnofsky Performance Status eCRF and the Chest X-Ray eCRF will be presented.

With reference to Section 6 General Considerations and Section 7 Statistical Considerations of this SAP, the following data will be summarized:

- Baseline height (cm).
- Baseline weight (kg).
- Baseline body mass index (BMI) (kg/m^2).
- Baseline CD4 count (cells/uL).
- Karnofsky score (%), where a score of 100 indicates normal, no complaints and 0 indicates death.

13. Disease History

Disease history as recorded on the TB and Human Immunodeficiency Virus (HIV) History eCRF will be presented.

With reference to Section 6 General Considerations and Section 7 Statistical Considerations of this SAP, the following data will be summarized:

- HIV status.
    - Negative.
    - Positive.
- Duration since HIV diagnosis (years): Calculated relative to the first study drug administration.
- Original TB diagnosis:
    - Drug-sensitive (DS).
    - Multi drug-resistant (MDR).
    - Extensively drug-resistant (XDR).
- Duration since original TB diagnosis (months): Calculated relative to the date of first study drug administration.
- Current TB diagnosis:
    - Extensively drug-resistant TB (XDR-TB).
    - Multi drug-resistant TB (MDR-TB) non-responsive.
    - Multi drug-resistant TB (MDR-TB) intolerant.
- Duration since current TB diagnosis (months): Calculated relative to the date of first study drug administration.
- Duration since most recent positive culture (days): Calculated relative to the date of first study drug administration.

13.1. DERIVATIONS

Duration since HIV (years):

- Duration (years) = Absolute value ([date of HIV diagnosis – date of first study drug administration]/]365.25]).

Duration since original/current TB diagnosis (months):

- Duration (months) = Absolute value ([date of diagnosis – date of first study drug adminisation]/]365.25/12]).

Duration since most recent positive culture (days):

- Duration (days) = Absolute value (date of most recent positive culture – date of first study drug administration).

14. CHEST X-RAY AT SCREENING

Chest X-Ray results at Screening as recorded on the Chest X-ray eCRF will be presented.

With reference to Section 6 General Considerations and Section 7 Statistical Considerations of this SAP, the following data will be summarized:

- Result.
  - Normal.
  - Abnormal.
- Compatible with TB:
  - Yes.
  - No.
- Cavities:
  - No cavities.
  - Unilateral cavities.
  - Bilateral cavities.

15. OPHTHALMOLOGY HISTORY

Ophthalmology history as recorded on the Ophthalmology Medical History eCRF will be presented.

With reference to Section 6 General Considerations and Section 7 Statistical Considerations of this SAP, the following data will be summarized:

- Personal history of vision or eye disorders:
    - Yes.
    - No.
- Immediate family history of cataracts:
    - Yes. If yes, description, type and severity of cataracts.
    - No.
- Personal history or prior refractive eye surgery:
    - Yes.
    - No.
- History of eye trauma
    - Right eye:
        - Yes.
        - No.
    - Left eye:
        - Yes.
        - No.

16. DRUG RESISTANCE HISTORY

Drug resistance history as recorded on the TB and HIV History eCRF will be presented.

With reference to Section 6 General Considerations and Section 7 Statistical Considerations of this SAP, the following data will be summarized:

- Result per applicable drug:
    - Sensitive.
    - Resistant.
    - Indeterminate.
    - Missing.

17. BASELINE DRUG SUSCEPTIBILITY

Baseline drug susceptibility as recorded on the Mycobacteriology Laboratory: Mycobacteriology Characterization eCRF.

With reference to Section 6 General Considerations and Section 7 Statistical Considerations of this SAP, the following data will be summarized:

- Result per applicable drug and per applicable method:
    - Resistant.
    - Sensitive/susceptible.
    - Contaminated/indeterminate/missing.

18. CONCOMITANT MEDICATIONS

Medications as recorded on the Concomitant Medications eCRF will be coded using World Health Organization–Drug Dictionary (WHO-DD). The version of the coding dictionary will be updated at an ongoing basis, to the latest version, during the course of the study.

Medications will be classified as:

- Prior medications (P): Medications starting and ending prior to the first study drug administration.
- Concomitant medications (C): Medications taken on or after the first study drug administration up to and including 14 days after the last study drug administration or are ongoing.
- Post-treatment medications (F): Medications taken after the last study drug administration date + 14 days or are ongoing.

Concomitant medications and post-treatment medications are not mutually exclusive and a medication can therefore be classified as both C/F.

Medications of interest include:

- Anti-retroviral medications: All medications where the ATC level 1 code starts with J05AF, J05AG or J05AR.
- TB medications: All medications where the indication for the medication is recorded as XDR and/or MDR as confirmed by TB Alliance.

19. CONCOMITANT PROCEDURES

Concomitant procedures as recorded on the Concomitant Procedures eCRF will be presented.

With reference to Section 6 General Considerations and Section 7 Statistical Considerations of this SAP, the data will be summarized by System Organ Class (SOC), and Preferred Term (PT). If a concomitant procedure occurs more than once for a subject per level of summarization, the subject will only be counted once per Medical Dictionary for Regulatory Activities (MedDRA) SOC or PT. System Organ Classes will be sorted by total descending frequency. Preferred Terms (PTs) will be sorted by total descending frequency within each SOC. If SOCs or PTs have the same total frequency they will be sorted alphabetically

Partial dates will not be imputed and as such study day will be presented as missing. Uncoded concomitant procedures will be presented as applicable using the Investigator's Verbatim Term (eCRF) presented as PT within the text 'UNCODED' as SOC (only applicable for the IA).

Concomitant procedures are coded using MedDRA. The version of the coding dictionary will be updated at an ongoing basis, to the latest version, during the course of the study.

20. MEDICAL HISTORY

Medical history as recorded on the Medical/Treatment History eCRF will be presented.

With reference to Section 6 General Considerations and Section 7 Statistical Considerations of this SAP, the following data will be summarized by SOC and PT. If a medical history condition occurs more than once for a subject per level of summarization, the subject will only be counted once per Medical Dictionary for Regulatory Activities (MedDRA) SOC or PT. System Organ Classes will be sorted by total descending frequency. Preferred Terms (PTs) will be sorted by total descending frequency within each SOC. If SOCs or PTs have the same total frequency they will be sorted alphabetically.

Partial dates will not be imputed and as such study day will be presented as missing. Uncoded medical history will be presented as applicable using the Investigator's Verbatim Term (eCRF) presented as PT within the text 'UNCODED' as SOC (only applicable for the IA).

Medical history is coded using MedDRA Version. The version of the coding dictionary will be updated at an ongoing basis, to the latest version, during the course of the study.

21. STUDY DRUG EXPOSURE

Study drug administration data as recorded on the Study Drug Dosing eCRF, missed dose data as recorded on the Missed Doses eCRF and premature study discontinuations due to an AE as recorded on the Subject Disposition eCRF will be presented.

With reference to Section 6 General Considerations and Section 7 Statistical Considerations of this SAP, the following data will be summarized:

- Actions (study drug administration, missed doses, interruptions) that occurred simultaneously for all three drugs and separately for Linezolid, irrespective of whether it occurred in the other two drugs:
  - Number of subjects with premature study drug administration discontinuation due to an adverse event.
  - Number of subjects with at least one dose interruption. Interruptions less than 2 days will not be regarded as an interruption.
  - Primary reason for dose interruption:

        - Adverse event.
        - Other.
    - Maximum duration of dose interruption due to an adverse event (days).
    - Total duration of dose interruption due to an adverse event (days).

- The following will be presented for Linezolid only, irrespective of whether it occurred in the other two drugs:
    - Number of subjects with at least one Linezolid reduction.
    - Reason for Linezolid dose reduction:
        - Adverse event.
        - Other.
    - Number of subjects with at least one Linezolid dose interruption and/or dose reduction due to an adverse event. Subjects are only counted once per category, regardless of the number of events.
- Linezolid administration will be summarized in a table, including the following (not applicable for the first IA analysis):
    - Planned cumulative dose (mg).
    - Actual cumulative dose (mg).
    - Relative dose (%).

The following will be presented as a by-subject figure for subjects who had a dose interruption and/or dose reduction and/or premature discontinuation of Linezolid due to an AE:

- Linezolid administration timeline according to the study day (x-axis) and by total daily dose (y-axis). Missed doses will be indicated by means of a green circle annotated on the figure for the duration of the missed doses and interruptions by means of a red line annotated on the figure for the duration of each interruption.
- A separate timeline for each of the other two study drugs will also be presented, similar to that of Linezolid.
- Adverse events related to relevant actions for each study drug will be displayed on the bottom section of the figure according to the relative study day (x-axis). Each AE will be displayed on a separate line and labeled as AE1 to AEx where x is the total number of AEs related to the applicable actions taken. The Preferred Term of each corresponding number and relevant severity (grade) will be listed below the contents of the figure.

A Kaplan-Meier plot will be presented for:

- Time (days) to first dose interruption and/or dose reduction of Linezolid due to AE.
- The figure will be repeated for the following subsets of subjects:
  - Subjects where the AE associated with the interruption and/or dose reduction is myelosuppression. Myelosuppresion will be identified by means of the AE System Organ Class (SOC) = BLOOD AND LYMPHATIC SYSTEM DISORDERS.
  - Subjects where the AE associated with the interruption and/or dose reduction is neuropathy. Neuropathy will be identified by means of the AE High Level Group Term (HLGT) = PERIPHERAL NEUROPATHIES.

The LIFETEST procedure in SAS® is a non-parametric procedure for analyzing survival data to compute the Kaplan-Meier plot, which is a non-parametric maximum likelihood estimate of the survival functions.

The following SAS® code will be utilized:

```
proc lifetest data=dd outsurv=dd1 method=KM alpha=0.05 alphaqt=0.05 conftype=linear
plots=(survival(strata=individual));
                    time <survival time> * <censoring indicator>;
                    by treatment;
run;
```

With the following indicators for the censoring of subjects:

- 1: If the subject had the event meeting the criteria for the specific event.
- 0: If the subject did not have the event meeting the criteria. Such a subject will be censored.

Subjects who do not have a dose interruption and/or dose reduction in Linezolid due to an AE will be censored at the time of treatment discontinuation or treatment completion.

21.1. DERIVATIONS

Based on the aforementioned, the following variables, as presented in the planned output shells will be derived:

- Duration of study drug administration/interruption/missed dose:
  - Duration of study drug administration/interruption/missed dose (days) = (End date of applicable event – start date of applicable event) + 1.
- Total duration on treatment (months):
  - Total duration on treatment= (Total duration of all individual study drug administrations – total duration of all missed doses)/(365.25/12).
- Reduction in Linezolid:
  - Records where the total daily dose decreases. An example would be 600 mg BID to 600 mg QD or 600 BID to 300 BID.
- Maximum duration of interruption (days):
  - For subjects who had more than one interruption due to an AE, only the dose interruption with the longest duration will be considered.
- Total duration of interruption (days):
  - For subjects with more than one dose interruption due to an AE, the duration of all dose interruptions due to an AE will be summed.
- Premature study drug administration discontinuation:
  - The B-L-Pa combination will include subjects who prematurely discontinue study drug as recorded on the Subject Disposition eCRF and the reason for premature discontinuation is adverse event or death. For Linezolid this is where the main reason of the last action taken per subject for Linezolid on the Study Drug Dosing eCRF is indicated as adverse event.
- Time (days) to first dose interruption and/or dose reduction of Linezolid due to an AE:
  - Time (days) = (Date of interruption or reduction – date of first study drug administration) + 1.

- Planned cumulative dose (mg) for Linezolid:
  - Planned cumulative dose (mg) = (Planned Dose x total number of days on Linezolid) where planned Dose = 1200 mg total daily dose in accordance with the study protocol.
- Actual cumulative dose (mg) for Linezolid:
  - Actual cumulative dose (mg) =$\sum_{x=1}^{n} \text{Actual Total Daily Dose} \times \text{total number of days on specific dose}$ where n is equal to the number of times a subject is on different doses (for example 600 BID versus 600 QD) and x is the specific dose. If a subject remains on one total daily dose (for example 1200 mg) for the entire treatment period, then n will be equal to 1.
- Relative dose (%) for Linezolid:
  - Relative Dose (%) = (Actual cumulative dose/planned cumulative dose) x 100.

22. Efficacy Analysis (only applicable to the first IA)

The efficacy analysis of the primary and key secondary efficacy endpoints will be performed using the ITT analysis population (first IA analysis).

22.1. Primary Efficacy

22.1.1. Derivations

At each visit two culture results will be available, a spot sample and an early morning sample. The derived culture result per sample will be derived as indicated in the table below. Subjects who are not able to produce sputum will be regarded as negative for that applicable sample.

Table 3: Derived Culture Result per Sample Algorithm

MGIT	ZNS	Blood Agar	Speciation*	Derived Culture Result per Sample	TTP Valid (Y/N)
Positive	Positive	Negative	Not done	Positive	Y
Positive	Positive	Negative	Positive	Positive	Y
Positive	Positive	Negative	Negative	Contaminated	N

MGIT	ZNS	Blood Agar	Speciation*	Derived Culture Result per Sample	TTP Valid (Y/N)
Positive	Positive	Positive or Other	Not done	Contaminated	N
Positive	Positive	Positive or Other	Positive	Contaminated	N
Positive	Positive	Positive or Other	Negative	Contaminated	N
Positive	Negative	Positive or Other	Not done	Contaminated	N
Positive	Negative	Negative	Not done	Contaminated	N
Negative	Not done	Not done	Not done	Negative	N
Negative	Positive	Negative	Positive	Negative	N
MGIT: Mycobacterial growth indicator tube. TTP: Time to sputum culture positivity. ZNS: Ziehl-Neelsen smear. *Only applicable for results at Baseline and from Week 16 onwards.					

A final single result per visit will be derived as indicated in the table below. For all analysis purposes (except for stable culture negative conversion), the derived result at Baseline will be considered positive for all subjects, regardless of the actual culture result derived per sample.

Table 4: Derived Result per Visit Algorithm

Spot Sample Derived Culture Result	Early Morning Derived Culture Result	Final Derived Result per Visit
Positive	Missing/Negative/Contaminated	Positive
Missing/Negative/Contaminated	Positive	Positive
Negative	Missing/Contaminated	Negative
Missing/Contaminated	Negative	Negative
Contaminated	Missing/Contaminated	Contaminated
Missing/Contaminated	Contaminated	Contaminated

Figure 2: Algorithm for Interpretation of Positive MGIT Results

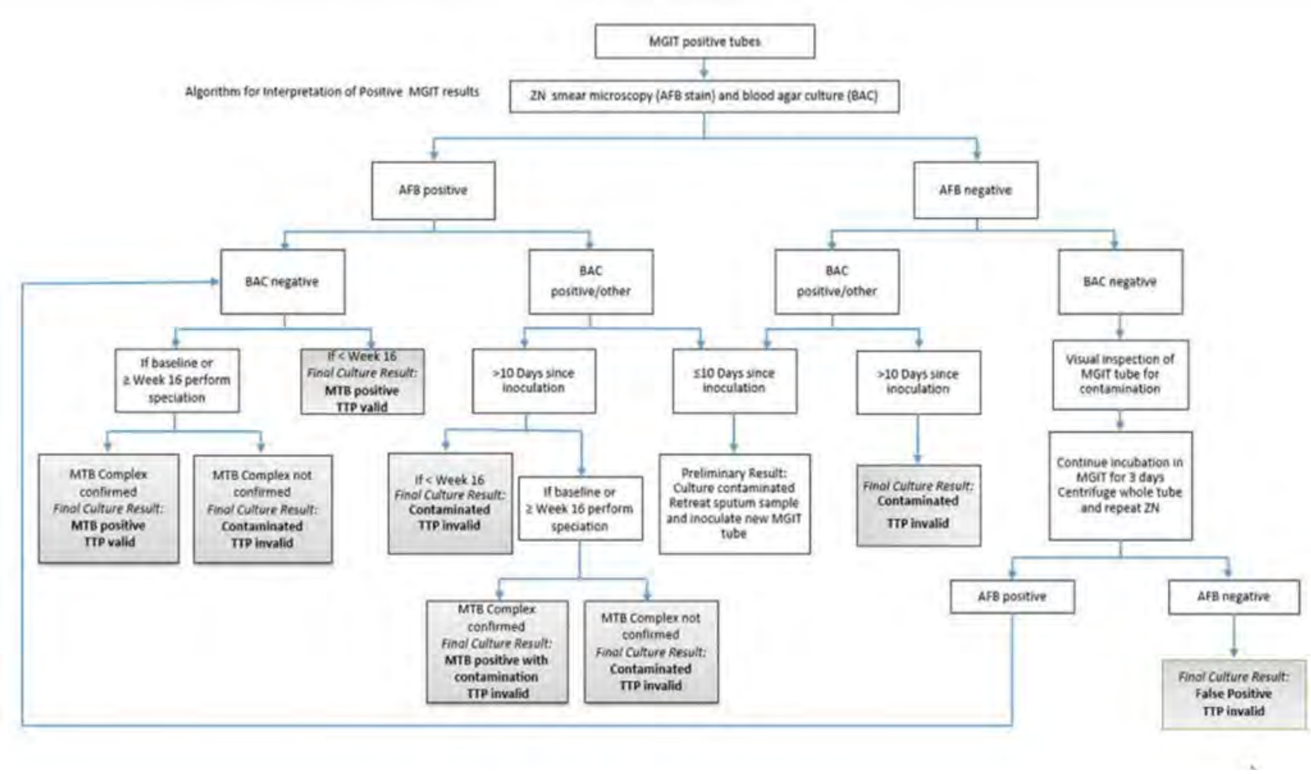

AFB: ZNS result. BAC: Blood agar result.

For the derived clinical failure status, medications recorded on the Concomitant Medications eCRF where the indication is recorded as XDR or MDR will be considered to check if a subject received TB treatment after the first study drug administration. If the medication starts after the first study drug administration but before the End of Treatment, this will be considered as a medication used due to treatment failure. If the medication starts after the End of Treatment this will be considered a medication used due to relapse. In addition, TB-related death will be identified by means of the Cause of death's relationship to subject's TB question on the Subject Disposition eCRF.

For clinical failure (Investigator), the bacteriology, clinical and radiological deterioration as recorded on the Investigator Assessment eCRF will be considered for a failure. For a success, the TB treatment success result will be considered.

22.1.2. DEFINITIONS

The following definitions are applicable:

- Bacteriological failure is defined as failing to attain confirmed culture negative status by the End of Treatment.
- Relapse (reinfection and reoccurrence) is defined as failing to maintain derived culture negative status after the End of Treatment. Therefore, this will be all subjects who achieve stable culture negative conversion at the End of Treatment and who have two consecutive culture positive (based on derived result per visit) samples.
- Reinfection is defined as failing to main derived culture negative status after the End of Treatment due to reinfection with a genetically distinguishable Mycobacterium tuberculosis strain. This will be relapse with a genetically distinguishable Mycobacterium tuberculosis strain (if the result from 'Are the two strains indistinguishable?' is 'No') as recorded on the Mycobacteriology Laboratory: Mycobacteriology Characterization eCRF.
- Reoccurrence is defined as failing to maintain derived culture negative status after the End of Treatment with a genetically distinguishable identical Mycobacterium tuberculosis strain. This will be relapse with a genetically identical Mycobacterium tuberculosis strain (if the result from 'Are the two strains indistinguishable?' is 'Yes') as recorded on the Mycobacteriology Laboratory: Mycobacteriology Characterization eCRF.
- Clinical failure (derived) is defined as change from the protocol-specified TB treatment due to treatment failure, retreatment for TB during follow-up period or TB-related death.
- Clinical failure (Investigator) as recorded on the Investigator Assessment eCRF.
- An unfavorable outcome is defined as subjects with bacteriological failure and/or relapse and/or derived clinical failure.
- Favorable outcome is defined as subjects who are not regarded as unfavorable.
- Stable culture negative conversion: At least 2 consecutive culture negative (based on derived result per visit) samples at least 7 days apart.

22.1.3. PRIMARY EFFICACY ENDPOINT

The primary efficacy endpoint if the study is:

- Proportion of treatment failure (unfavorable outcome), defined as bacteriologic failure or relapse or clinical failure (derived) through follow-up until 6 months after the End of Treatment.

They key secondary endpoints of this study are:

- Time to sputum culture conversion to negative status through the treatment period.
- Proportion of subjects with sputum culture conversion to negative status at 4, 6, 8, 12, 16 and 26 or 39 weeks.

22.1.4. ANALYSIS METHODS

A 95% CI based on the Clopper-Pearson 95% CI for a single binomial proportion will be calculated for the:

- Proportion of subjects with:
  - o An unfavorable outcome as defined above. Percentage (%) of subjects relative to the total number of subjects in the relevant analysis population.

The following SAS® code will be utilized:

```
proc freq data=dd ;
        tables variable / binomial (exact) alpha = .05;
        by treatment;
run;
```

Given that the ITT analysis population is defined as all subjects assigned to treatment, subjects with no evidence of study drug administration or insufficient data to determine unfavorable/favorable outcome will be regarded as favorable.

The following key secondary endpoint will be analyzed in a similar manner as described above:

- Time to sputum culture conversion to negative status through the treatment period.

- Proportion of subjects with sputum culture conversion to negative status at 4, 6, 8, 12, 16 and 26 or 39 weeks.

A 95% CI based on the Clopper-Pearson 95% for a single binomial proportion will be calculated for the:

- Subjects with a stable culture negative conversion at each post-baseline visit.
- Proportion of subjects with:
  - Favorable outcome. Percentage (%) of subjects relative to the total number of subjects in the relevant analysis population.
  - Bacteriological failure. Percentage (%) of subjects relative to the total number of subjects in the relevant analysis population.
  - Relapse. Percentage (%) of subjects relative to the total number of subjects in the relevant analysis population with a derived culture negative status at the End of Treatment.
  - Relapse due to a reinfection. Percentage (%) of subjects relative to the total number of subjects in the relevant analysis population with a derived culture negative status at the End of Treatment.
  - Relapse due to reoccurrence. Percentage (%) of subjects relative to the total number of subjects in the relevant analysis population with a derived culture negative status at the End of Treatment.
  - Clinical failure treatment failure. Percentage (%) of subjects relative to the total number of subjects in the relevant analysis population.
  - Clinical failure during following-up period. Percentage (%) of subjects relative to the total number of subjects in the relevant analysis population who completed treatment and entered the follow-up period.
  - Clinical failure (Investigator's assessment) at Month 6. Percentage (%) of subjects relative to the total number of subjects in the relevant analysis population who completed treatment and entered the follow-up period.
  - Clinical failure (Investigator's assessment) at Month 24. Percentage (%) of subjects relative to the total number of subjects in the relevant analysis population who completed treatment and entered the follow-up period.

In addition, Kaplan-Meier analysis of the median time (days) to event will be performed (refer to 21.1: Derivations for relevant information regarding the Kaplan-Meier analysis) for the following:

- Time to first stable culture negative conversion (days) = (Date of first stable culture negative conversion – date of first study drug administration) + 1. The first of the two negative cultures without an intervening positive culture will be considered. Subjects who do not have a stable culture negative conversion will be censored at the time of treatment discontinuation or treatment completion. Only subjects with a positive culture status at Baseline will be considered.
- Time to relapse/reinfection/reoccurrence (days) = (Date of relapse/reinfection/reoccurrence – date of last study drug administration) + 1. Subjects who do not have a relapse/reinfection/reoccurrence will be censored at the time of premature study discontinuation or study completion.

22.2. OTHER SECONDARY EFFICACY

22.2.1. OTHER SECONDARY EFFICACY ENDPOINTS AND DEFINITIONS

The other secondary efficacy endpoints of this study are:

- Change from Baseline in TB symptoms (refer to Section 22.2.2: Tuberculosis Symptoms).
- Change from Baseline in Patient Reported Health Status (refer to Section 22.2.3: Subject Reported Healthy Status: EQ-5D-5L).
- Change from Baseline in weight (kg) (refer to Section 22.2.4: Weight).

The analysis will be performed using the Safety analysis population defined as all subjects who received at least one administration of study drug.

22.2.2. TUBERCULOSIS SYMPTOMS

Tuberculosis symptom data as recorded on the Tuberculosis Symptom Profile eCRF will be presented.

With reference to Section 6 General Considerations and Section 7 Statistical Considerations of this SAP, the following data will be summarized:

- TB symptoms:
  - Feeling feverish.
  - Feeling chills.
  - Excessive sweating.
  - Shortness of breath.
  - Chest pain.
  - Feeling unwell.
  - Tiredness/weakness.
  - Cough.
  - Coughing up mucus.
  - Coughing up blood.
- And the result of each TB symptom at each scheduled visit:
  - None.
  - Mild.
  - Moderate.
  - Severe.
- A shift table for change from Baseline at the End of Treatment/End of Study (when applicable) will be presented. Percentage (%) of subjects in each category will be calculated relative to the total number of subjects in the relevant analysis population, with assessments available at Baseline and End of Treatment/End of Study (when applicable) visit.
- The results for each TB symptom at each visit will also be presented in a stacked bar graph displaying the number of subjects with each result. All mild, moderate or severe results will be displayed in a by-subject data listing.

22.2.3. Subject Reported Health Status: EQ-5D-5L

Subject reported health status: European quality of life – 5 dimensions (EQ-5D-5L) data as recorded on the EQ-5D-5L eCRF will be presented.

With reference to Section 6 General Considerations and Section 7 Statistical Considerations of this SAP, the following data will be summarized:

- Subjects reported health status: EQ-5D-5L qualitative items and applicable results:
    - Mobility.
    - Self-care.
    - Usual activities.
    - Pain/discomfort.
    - Anxiety/depression.
- And the result of each item at each scheduled visit:
    - None.
    - Slight.
    - Moderate.
    - Severe.
    - Extreme/unable.
- Subject reported health status: EQ-5D-5DL quantitative item:
    - Visual analog scale (VAS) Score. A score of 100 means the best health one can imagine and 0 means the worst health one can imagine. Please see below:

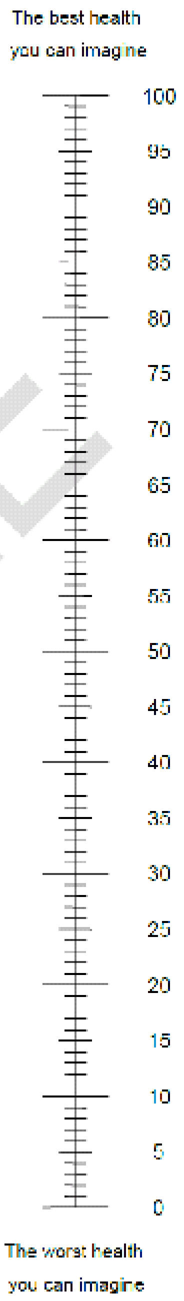

- o Observed result at each visit.

  - Change from Baseline and Baseline value at each post-baseline visit and the End of Treatment/End of Study (when applicable). Change from Baseline will only be derived if both the Baseline and the relevant post-baseline/End of Treatment/End of Study (when applicable) assessment are available.

- A shift table for change from Baseline at the End of Treatment/End of Study (when applicable) will be presented. Percentage (%) of subjects in each category will be calculated relative to the total number of subjects in the relevant analysis population, with assessments available at Baseline and End of Treatment/End of Study (when applicable) visit.
- The result for each item at each visit will also be presented in a stacked bar graph displaying the number of subjects with each result.
- A scatterplot for VAS score by visit will be provided.

22.2.4. WEIGHT

Weight (kg) as recorded on the Vital Signs eCRF will be presented.

With reference to Section 6 General Considerations and Section 7 Statistical Considerations of this SAP, the following data will be summarized:

- Observed result at each visit.
- Change from Baseline and Baseline value at each post-baseline visit and the End of Treatment/End of Study (when applicable). Change from Baseline will only be derived if both the Baseline and the relevant post-baseline/End of Treatment/End of Study (when applicable) assessment are available.
- Percentage (%) change from Baseline at each visit. This is only applicable to the figure presentation of mean (+/-) SD at each relevant post-baseline visit.

All assessments (scheduled or unscheduled) will be presented in the by-subject data listings.

23. SAFETY OUTCOMES

The Safety analysis population will be used as primary analysis population for all safety analyses.

23.1. ADVERSE EVENTS

Adverse events (AEs) will be presented as recorded on the Adverse Events eCRF.

Adverse events will be categorized as follows:

- Prior AEs: Defined as AEs which started prior to the first study drug administration.
- TEAE: Defined as AEs which started or worsened on or after the first study drug administration up to and including 14 days after the last study drug administration.
- Post-treatment AE: Defined as AEs which started or worsened after 14 days after the last study drug administration.

Adverse events (AEs) will be coded using Medical Dictionary for Regulatory Activities (MedDRA) Version 19.0.

With reference to Section 6 General Considerations and Section 7 Statistical Considerations of this SAP, the incidence of TEAEs will be presented by SOC and PT or SOC, high level group term (HLGT) and PT (AEs of special interest only). Uncoded AEs (i.e., AEs with no dictionary coding information available) are only to be presented if applicable using the Investigator's Verbatim Term (eCRF) as PT within the text 'UNCODED' as SOC (only applicable for IA). System Organ Classes (SOCs) will be sorted by total decreasing frequency. High level group terms (HLGT) (if applicable) will be sorted by total decreasing frequency with each SOC. Preferred Terms (PTs) will be sorted by total decreasing frequency within each SOC or HLGT (if applicable). If SOCs, HLGTs (if applicable) or PTs have the same total frequency will be sorted alphabetically.

For presentation and derivation purposes, the following statistics are defined:

- n: Defined as the number of subjects with at least one TEAE in each category. Subjects with multiple TEAEs in each category are counted only once in each category.
- E: Defined as the number of mentions (events) in each category, i.e., the actual unique number of events.

- N: Defined as the total number of subjects in the relevant analysis population.
- %: Defined as the percentage of subjects with at least one TEAE in each category calculated relative to the total number of subjects in the relevant analysis population.

23.1.1. SEVERITY

The severity (Division of Microbiology and Infectious Disease [DMID] grading) of each AE, as indicated by the Investigator, is classified as:

- Grade 1 (mild).
- Grade 2 (moderate).
- Grade 3 (severe).
- Grade 4 (potentially life threatening).

23.1.2. RELATIONSHIP

The relationship to the study drug regimen, as indicated by the Investigator, is classified as:

- Not related.
- Unlikely.
- Possible.
- Probable.
- Certain.

If the relationship to the study drug regimen is indicated as 'Possible', 'Probable', 'Certain' by the Investigator, the relationship to Investigational medicinal product (MP) will be assessed for:

- Bedaquiline:
    - Not related.
    - Unlikely.
    - Possible.
    - Probable.
    - Certainly.

- Pretomanid (same categories as for Bedaquiline).
- Linezolid (same categories as for Bedaquiline).

The relationship to each IMP was only added for data entered from 01 January 2017 onwards and will therefore only be presented in by-subject data listings.

23.1.3. ACTION TAKEN WITH STUDY DRUG

The action taken with study drug (overall), as indicated by the Investigator, is classified as:

- Investigational medicinal product (IMP) unchanged.
- Investigational medicinal product (IMP) interrupted.
- Investigational medicinal product (IMP) stopped.
- Not applicable.
- Investigational medicinal product (IMP) dose reduced.

The action taken with Linezolid IMP, as indicated by the Investigator, is classified as:

- Investigational medicinal product (IMP) unchanged.
- Investigational medicinal product (IMP) interrupted.
- Investigational medicinal product (IMP) stopped.
- Not applicable.
- Investigational medicinal product (IMP) dose reduced.

The action taken with Bedaquiline/Pretomanid IMP, as indicated by the Investigator, is classified as:

- Investigational medicinal product (IMP) unchanged.
- Investigational medicinal product (IMP) interrupted.
- Investigational medicinal product (IMP) stopped.
- Not applicable.
- Investigational medicinal product (IMP) dose reduced.

The action taken with each IMP was only added for data entered from 01 January 2017 onwards and will therefore only be presented in by-subject data listings.

Action taken (overall) classified as IMP stopped will be used to identify and select all AEs leading to discontinuation of study drug. Action taken classified as IMP interrupted will be used to identify and select all AEs leading to interruption of study drug.

Drug associated with the action taken with study drug will be obtained from the Study Drug Dosing eCRF by means of the action taken with Linezolid IMP and/or action taken with Bedaquiline/Pretomanid IMP.

23.1.4. Adverse Events (AEs) Leading to Death

Adverse events (AEs) leading to death are selected as all AEs with an outcome recorded as 'Fatal'.

23.1.5. Serious Adverse Events (AEs)

Serious AEs are events judged as 'Serious' by the Investigator. Serious criteria include the following events:

- Death.
- Life-threatening.
- Initial or prolonged hospitalization.
- Persistent or significant disability/incapacity.
- Congenital anomaly or birth defect.
- Other medically important event (not covered by other 'serious' criteria).

23.1.6. Adverse Events (AEs) Leading to Discontinuation of Study Drug

Adverse events (AEs) leading to permanent discontinuation of study drug will be identified and selected based on response of 'Yes' to the question 'Did the adverse event cause the subject to be discontinued from the study?

23.1.7. All Treatment-emergent AEs (TEAEs)

Based on the aforementioned definition, an overview summary will be provided as follows:

Variable
SUBJECTS WITH AT LEAST ONE
TEAE
TEAES LEADING TO DEATH
SERIOUS TEAE (INCLUDING DEATH)
TEAE LEADING TO EARLY WITHDRAWAL
TEAE LEADING TO DISCONTINUATION OF STUDY DRUG
TEAE LEADING TO INTERRUPTION OF STUDY DRUG
GRADE III AND/OR IV TEAE
DRUG-RELATED TEAE
TEAE OF SPECIAL INTEREST

23.1.8. Adverse Events (AEs) of Special Interest

Adverse events (AEs) of special interest will be selected based on an identified list of PT, High Level Term (HLT) and High Level Group Terms (HLGT) and confirmed by TB Alliance.

The number (n) and percentage (%) of subjects with at least on TEAE of special interest will be presented as a histogram with HLGT terms on the x-axis and the number of subjects on the y-axis.

The summary table will be presented by SOC, HLGT and PT.

Preferred Term
ALANINE AMINOTRANSFERASE INCREASED
ASPARTATE AMINOTRANSFERASE INCREASED
BLOOD ALKALINE PHOSPHATASE INCREASED
AMYLASE INCREASED
LIPASE INCREASED
MYALGIA
MUSCLE NECROSIS
BLOOD CREATINE PHOSPHOKINASE MB INCREASED
ELECTROCARDIOGRAM QT PROLONGED
BUNDLE BRANCH BLOCK LEFT

**Preferred Term**
ATRIOVENTRICULAR BLOCK SECOND DEGREE
ATRIOVENTRICULAR BLOCK COMPLETE
HAEMOGLOBIN DECREASED
NEUROPATHY PERIPHERAL
VISUAL ACUITY REDUCED
CHROMATOPSIA
LACTICT ACIDOSIS
BLOOD BICARBONATE DECREASED
ANAEMIA
BURNING SENSATION
GENERALISED TONIC-CLONIC SEIZURE
HYPERAMYLASAEMIA
NEUTROPENIA
DISSEMINATED TUBERCULOSIS
PAPILLOEDEMA
EYE SWELLING
PARAESTHESIA
BONE MARROW FAILURE
LIPASE URINE INCREASED
**High Level Term**
NEUTROPENIAS
PERIPHERAL NEUROPATHIES NEC
THROMBOCYTOPENIAS
**High Level Group Term**
HEPATIC AND BILIARY NEOPLASMS BENIGN
HEPATIC AND HEPATOBILIARY DISORDERS
HEPATOBILIARY DISORDERS CONGENITAL
HEPATOBILIARY NEOPLASMS MALIGNANT AND UNSPECIFIED
HEPATOBILIARY INVESTIGATIONS
HEPATOBILIARY THERAPEUTIC PROCEDURES
CARDIAC ARRHYTHMIAS
NEUROPENIAS
THROMBOCYTOPENIAS

23.1.1. LIVER-RELATED AEs

Liver-related AEs will be selected based on an identified list of High Level Group Terms (HLGT) and confirmed by TB Alliance.

The summary table will be presented by SOC and PT.

High Level Group Term
HEPATIC AND BILIARY NEOPLASMS BENIGN
HEPATIC AND HEPATOBILIARY DISORDERS
HEPATOBILIARY DISORDERS CONGENITAL
HEPATOBILIARY NEOPLASMS MALIGNANT AND UNSPECIFIED
HEPATOBILIARY INVESTIGATIONS
HEPATOBILIARY THERAPEUTIC PROCEDURES

23.1.2. DERIVATIONS

Adverse event duration (days) is calculated as follows:

- Adverse event duration (days) = (Stop date - start date) + 1.

23.2. LABORATORY TESTS

Safety laboratory data as captured on the Safety Laboratory Sample Collection and Safety Laboratory Test Results eCRFs will be presented. All relevant laboratory category/variable and unit are presented in Laboratory Tests of this SAP.

With reference to Section 6 General Considerations and Section 7 Statistical Considerations of this SAP, the following data will be summarized:

- Observed result at each visit.
- Baseline and change from Baseline at each post-baseline visit and the End of Treatment. Change from Baseline will only be derived if both the Baseline and the relevant post-baseline/End of Treatment assessment are available.

23.2.1. Division of Microbiology and Infectious Disease (DMID) Grading for Laboratory Data

The laboratory results will be graded programmatically using the DMID, Version November 2007. For details regarding each of the following levels, refer to Appendix 5: Division of Microbiology and Infectious Disease (DMID) Laboratory Tests Toxicity of this SAP:

- Grade 1.
- Grade 2.
- Grade 3.
- Grade 4.

Subjects with a result not complying with the DMID grades for a given laboratory assessment will be assigned a Grade 0 severity.

With reference to Section 6 General Considerations and Section 7 Statistical Considerations of this SAP, the following data will be summarized:

- Division of Microbiology and Infectious Disease (DMID) grade for each relevant laboratory test at each post-baseline visit.
    - Percentage of subjects in each category relative to the number of subjects in the relevant population with data available for the applicable test at the applicable visit
- A change in each laboratory variable based on the shift from Baseline DMID grade to the End of Treatment.
    - Percentage of subjects in each category relative to the number of subjects in the relevant population with data available for the applicable test at both Baseline and the End of Treatment visit.
- A figure of the mean +/- SD per visit will be presented for those laboratory categories and tests with at least one ≥ grade 3 values.
- Laboratory profile plots will be created per subject, for subjects with a ≥ grade 3 toxicity value for the following categories and tests:

- o Hematology: Hemoglobin, platelets, neutrophils and/or white blood cells. All tests will be plotted on one figure, by visit (x-axis) and upper limit of normal (ULN) normalized value (y-axis).
- o Chemistry: Amylase and lipase in a similar format as described for the hemoglobin, platelets, neutrophils and/or white blood cells figure.

23.2.2. LIVER-RELATED ABNORMALITIES AND EVALUATION IF DRUG-INDUCED SERIOUS HEPATOTOXICITY

With reference to Section 6 General Considerations and Section 7 Statistical Considerations of this SAP, the following data will be summarized:

- The classification of liver-related abnormalities: Alanine aminotransferase (ALT), aspartate aminotransferase (AST), alkaline phosphatase (ALP) and total bilirubin at each visit, End of Treatment and treatment-emergent incidence as follows:
  - o > 3 x upper limit of normal (ULN).
  - o > 3 x ULN.
  - o > 8 x ULN.
  - o Classification categories are not mutually exclusive.
  - o Treatment-emergent incidence includes all liver-related abnormalities (per classification) after the study drug administration up to and including 14 days after the last study drug administration.
  - o Percentage of subjects in each category relative to the total number of subjects in the relevant analysis population with data available for each specific test at each applicable visit.
- The evaluation of drug-induced serious hepatotoxicity based on the following classifications at each visit:
  - o Total bilirubin ≥ 2 x ULN and ALT ≥ 3 x ULN (possible Hy's law).
  - o Total bilirubin > 2 x ULN and ALT > 3 x ULN.
  - o Total bilirubin > 2 x ULN and ALT > 5 x ULN.
  - o Total bilirubin > 2 x ULN and ALT > 8 x ULN.
  - o Classification categories are not mutually exclusive.
  - o Treatment-emergent incidence includes all liver-related abnormalities (per classification) identified after the first study drug administration up to and including 14 days after the last study drug administration.

- o Percentage of subjects in each category relative to the number of subjects in the relevant population with data available for both total bilirubin and ALT.
- o Similar classifications for total bilirubin and AST will be performed.

All assessments (scheduled or unscheduled) will be presented in by-subject data listings.

The following figures will be presented:

- An eDISH plot of treatment-emergent incidence of total bilirubin versus ALT. A similar plot of total bilirubin versus AST will be produced.
- By-subject profile plots for subjects with treatment-emergent eDISH abnormalities including the following laboratory tests: ALT, AST, ALP and total bilirubin.

23.2.3. Derivations

Numerical laboratory assessments reported as ‘< X’, i.e. below limit of quantification (BLQ), or ‘> X’, i.e. above the upper limit of quantification (ULQ), will be converted to X for the purpose of numerical summaries, but will be presented as recorded, i.e. as ‘< X’ or ‘> X’ in the by-subject data listings.

Quantitative laboratory assessments will be categorized in accordance with the relevant laboratory reference ranges as follows:

- Low: Below the lower limit of the laboratory reference range.
- Normal: Within the laboratory reference range (upper and lower limit included).
- High: Above the upper limit of the laboratory reference range.

23.3. Vital Signs

Vital signs data (BMI, systolic blood pressure, diastolic blood pressure, heart rate, axillary body temperature and respiratory rate) as captured on the Vital Signs eCRF will be presented.

With reference to Section 6 General Considerations and Section 7 Statistical Considerations of this SAP, the following data will be summarized:

- Observed result at each visit.

- Baseline and change from Baseline results at each post-baseline visit and the End of Treatment/End of Study (when applicable). Change from Baseline will only be derived if both the Baseline and the relevant post-baseline/End of Treatment/End of Study (when applicable) assessment is available.

All assessments (scheduled or unscheduled) will be presented in by-subject data listings.

23.3.1. NOTABLE ABNORMALITIES

The following vital signs assessments will be classified according to the criteria below, as specified in Appendix 3: Vital Signs of the study protocol:

- Diastolic blood pressure.
- Systolic blood pressure.

The categories are as follows:

- Abnormally low.
- Grade 1 or mild.
- Grade 2 or moderate.
- Grade 3 or severe.

Subjects with a result not complying with the above categories for a given vital signs assessment will be assigned Grade 0.

With reference to Section 6 General Considerations and Section 7 Statistical Considerations of this SAP, the following data will be summarized:

- Notable abnormality for each relevant vital signs test at each post-baseline visit.
    - o Percentage of subjects in each category relative to the number of subjects in the relevant population with data available for the applicable test at the applicable visit.
- A change in each vital signs test based on the shift from Baseline notable abnormality to the End of Treatment/End of Study (when applicable).

- o Percentage of subjects in each category relative to the number of subjects in the relevant population with data available for the applicable test at both Baseline and the End of Treatment/End of Study (when applicable) visit.

23.4. Electrocardiogram Parameters

Electrocardiogram (ECG) data (heart rate, PR, time required for depolarization and repolarization of ventricles [QT], QT interval corrected using Bazett's method [QTcB], QT interval corrected using Fridericia's method [QTcF]) as captured on the 12-Lead ECG Results eCRF will be presented.

The overall interpretation as judged by the Investigator will be classified and displayed in by-subject data listings only as:

- Normal.
- Abnormal, not clinically significant.
- Abnormal, clinically significant.

With reference to Section 6 General Considerations and Section 7 Statistical Considerations of this SAP, the following data will be summarized:

- Observed result at each visit.
- Baseline and change from Baseline results at each post-baseline visit and at the End of Treatment visits. Change from Baseline will only be derived if both the Baseline and the relevant post-baseline/End of Treatment assessment is available.

All assessments (scheduled or unscheduled) will be presented in by-subject data listings.

23.4.1. Electrocardiogram Prolongation

QT, QTcB and QTcF will be classified as.

- > 450 msec.
- > 480 msec.
- ≥ 500 msec.

The aforementioned categories are not mutually exclusive and a result can therefore be included in one or more categories.

- At post-baseline visits and the End of Treatment visit, the change from Baseline will be categorized as:
  - o > 30 msec increase from Baseline.
  - o > 60 msec increase from Baseline.
  - o A change will only be derived if both the Baseline and the relevant post-baseline or End of Treatment assessment is available. Percentages (%) will be based on the number of subjects with data available at both Baseline and the relevant post-baseline or End of Treatment assessments.

The following profile plot figures will be presented:

- Mean QT, QTcB, QTcF and heart rate intervals over time.
- Mean change from Baseline in QT, QTcB and QTcF intervals over time.

The following by-subject data listing will be presented:

- Electrocardiogram results.

23.5. OPHTHALMOLOGICAL EXAMINATION

Visual acuity and color vision data as captured on the Ophthalmology Examination (Visual acuity color vision) eCRF will be presented.

- The Ophthalmologist/delegate will test distance, near and visual acuity for both the right and the left eye. The best corrected visual acuity will be measured. Metric designations will be used.
  - o For the distance visual acuity the Snellen chart and notation will be used and the evaluation will be performed at 6 meters.
  - o For the near visual acuity the Jaeger chart and notation will be used. The evaluation will be performed at 40 cm.
- For color vision, each eye should be tested individually (covering the opposite eye), using the 24 plate Ishihara series, held out at a distance of 75 cm at a right angle to the subject.

- o All 24 plates in the book should be presented to the subject, one at a time. A total score out of 21 for each eye will be recorded on the color vision assessment form.

With reference to Section 6 General Considerations and Section 7 Statistical Considerations of this SAP, data will be summarized for visual acuity (near), visual acuity (distance) and color vision:

- Change from Baseline at each post-baseline visit and the End of Treatment/End of Study (when applicable) per eye, based on the following categories:
  - o > 2 categories increase.
  - o 2 category increase.
  - o 1 category increase.
  - o No change.
  - o > 2 categories decease.
  - o 2 category decease,
  - o 1 category decrease.
  - o Percentage (%) subjects in each category relative to the total number of subjects in the relevant analysis populations with data available at Baseline and the relevant post-baseline/End of Treatment/End of Study (when applicable) visit.

A worsening will be regarded as:

- Near: When the numeric value next to 'J' increases by one or more.
- Distance: When the numeric value next to '6/' increases by one or more.
- Color vision: When the numeric value decreases by one or more.

A by-subject figure will be presented for subjects who experience any worsening from Baseline in any of the aforementioned.

All scheduled and unscheduled assessments will be presented in a by-subject data listing.

23.6. Slit Lamp Examination

Age-related eye disease study 2 (AREDS2) lens grading data as captured on the Slit Lamp Examination eCRF will be presented.

The slit lamp examination results will be documented using the AREDS2 clinical lens opacity classification and grading system. With this system, the lens will be evaluated and graded by comparison to a series of standard photographs for each possible type of lens opacity, the location and degree of lens opacity being determined. The possible type of lens opacity and its grading will be recorded. The possible types are nuclear (9 possible grades), cortical (9 possible grades) and posterior sub-capsular (PSC) (9 possible grades).

The lens will be examined and compared to the corresponding series of three standard photographs for each of the three types of lens opacity. The lens will be graded according to these standard photographs for severity or extent of each type of lens opacity in the eye under consideration. The grades range from 0.0 (for clear), to 4.0 (for completely opacified), with the standards defining whole steps between these two extremes. The grading of 8.0 should be used if the lens cannot be evaluated.

Results according to the AREDS2 grading system:

- 0.0 = No lens opacity.
- > 0.0 to <= 4.0 = Lens opacity present.
- 8.0 = Cannot evaluate.

With reference to Section 6 General Considerations and Section 7 Statistical Considerations of this SAP, the following data will be summarized at each visit, and per eye:

- Proportion of subjects with opacity for the following categories:
    - Nuclear.
    - Cortical.
    - Posterior subcapsular.
    - Percentage (%) of subjects in each category relative to the total number of subjects in the relevant analysis population with data available for each eye and applicable visit.

All scheduled and unscheduled assessments will be presented in a by-subject data listing.

23.7. PERIPHERAL NEUROPATHY

Peripheral neuropathy data as captured on the Peripheral Neuropathy eCRF will be presented.

With reference to Section 6 General Considerations and Section 7 Statistical Considerations of this SAP, the following data will be summarized at each visit:

- Individual item and total neuropathy numeric score. Items include:
    - Rate of interference with walking or sleeping.
    - Subject elicited symptoms:
        - Pain, aching or burning in feet or legs.
        - 'Pins and needles' in feet and legs.
        - Numbness (lack of feeling) in feet or legs.
- A change in each item based on the shift from Baseline to the End of Treatment/End of Study (when applicable) based on the eCRF categories (response category) for each item. A change will only be derived if both the Baseline and the relevant End of Treatment/End of Study (when applicable) assessment is available. Percentages (%) will be based on the number of subjects with data available at both Baseline and the relevant End of Treatment/End of Study (when applicable) assessments.

23.7.1. Derivations

Total neuropathy symptom score is derived as the sum of the numeric scores of the following individual questions:

- Interference with walking or sleeping:
    - 01 (minimal).
    - 02 (minimal).
    - 03 (minimal).
    - 04 (modest).
    - 05 (modest).
    - 06 (modest).
    - 07 (modest).
    - 08 (severe).
    - 09 (severe).
    - 10 (severe).
- Pain, aching or burning in feet/legs.

- 00- Very happy, no symptoms.
- 02 – Just a little bit.
- 04 – A little more.
- 06 – Even more.
- 08 – A whole lot.
- 10 – Worst.

- Pins and needles in feet/legs (same categories as pain, aching or burning in feet/legs).
- Numbness (lack of feeling) in feet/legs (same categories as pain, aching or burning in feet/legs).

24. LIST OF REFERENCES

[1] NiX-TB Protocol Version 1 21APR2014.

[2] NiX-TB Protocol Version 2 final working protocol 18MAR2015.

[3] NiX-TB Protocol Version 3 final working protocol 22JAN2016.

[4] NiX-TB Annotated CRF Version 5.0 dated 11May2016.

APPENDIX 1. PROGRAMMING CONVENTIONS FOR TABLES, BY-SUBJECT DATA LISTINGS AND FIGURES

Paper Size, Orientation and Margins

The margin, page size and line size specifications as stipulated below will be used for the presentation of all TLFs:

	Landscape	Portrait
Margins (Inches):<br>Top<br>Bottom<br>Left<br>Right	 1.25<br>1<br>1<br>1	 1<br>1<br>1.25<br>1
Header (Inches)	0.5	0.5
Footer (Inches)	0.5	0.5
SAS® specifications:<br>PAGESIZE<br>LINE SIZE	 46<br>134	 67<br>93

Fonts

The font type ‘Courier New’ should be used as default for tables and by-subject data listings, with a font size of 8. The font color should be black. No bolding, underlining and italics are permitted.

1

TB Alliance	B-L-Pa
NiX-TB-(B-L-Pa)	Page xx of xx

Table 14.1.4
Demographics
Safety Analysis Population

By-group<xxxx> 2

Variable 3	Statistic	B-L-Pa XDR (N=15)	B-L-Pa MDR (N=0)	B-L-Pa Total (N=15)
AGE (YEARS) 4	n	15		15
	MEAN	33.1		33.1
	SD	10.87		10.87
	MINIMUM	20		20
	MEDIAN	31.0		31.0
	MAXIMUM	52		52
GENDER 5				
FEMALE	n (%)	5 (33.3) 6		5 (33.3)
CHILDBEARING POTENTIAL*				
YES	n (%)	5 (100.0)		5 (100.0)
NO	n (%)	0		0
NOT APPLICABLE	n (%)	0		0
MALE	n (%)	10 (66.6)		10 (66.6)

[illegible]atment group: B = Bedaquiline. L = Linezolid. Pa = Pretomanid. MDR: Multi drug-resistant. SD: Standard deviation. XDR: Extensively drug-resistant. n = Number of subjects in each category. 7 [illegible] Total number of subjects in the relevant analysis population. % = Percentage of subjects in each category relative to the total number of subjects in the relevant analysis population with data available. *: % for [illegible]dbearing potential = Percentage of subjects in each category relative to the total number of female subjects in the relevant analysis population with data available. Age (years): Calculated relative to Screening.

Program: x:\xxxxxx\xxxxxx\xxxxxx\xxxxxx.sas (ddmmmyyyy hh:mm) 8	File: xxxxxx.rtf

1. Header information. **2.** By-group information. **3.** Column heading information. **4.** General information. **5.** Denominator information. **6.** Alignment information. **7.** Footnote information. **8.** Filename information.

Footnotes are to be ordered as follows:

- Non-standard abbreviations, separated by a full stop.
- n = Number of subjects… N = Total number of subjects… % = Percentage of subjects…
- Definitions.
- Footnotes pertaining to statistical methodology.
- If coding is presented, the version of the coding dictionary used.
- Study-specific footnotes to clarify data points within the specific presentation.

Figure Output Conventions

Figures should be provided in RTF files using the SAS® Output Delivery System (ODS).

Dates and Times

Depending on data available, dates and times will take the form ddMMMyyyy and hh:mm.

Spelling Format

The spelling format to be used is English US.

Presentation of Visits

For outputs, visits will be represented in the following order:

Visit Name
Screening
Baseline (derived)
Day 1
Week 1
Week 2
Week 3
Week 4
Week 5
Week 6
Week 7
Week 8
Week 9
Week 10
Week 11
Week 12
Week 13
Week 14
Week 15
Week 16
Week 20
Week 26
End of Treatment (derived)
Month 1
Month 2
Month 3
Month 6
Month 9
Month 12

Visit Name
Month 15
Month 18
Month 21
Month 25
End of Study (derived)
Unscheduled (chronologically)
Early Withdrawal

APPENDIX 2. PARTIAL ADVERSE EVENT DATE CONVENTIONS

Date imputations for partial AEs are only used for classification of TEAEs and partial dates will be displayed in the applicable by-subject data listings.

Algorithm for Treatment-emergent Adverse Events

Start Date	Action
Known	If AE start date < Reference start or AE start date > Reference end date + 14 days, then not treatment-emergent adverse event (TEAE).<br>If Reference start date ≤ AE start date ≤ Reference end date + 14 days, then TEAE.<br>If AE start date = Study drug start date and the variable '...prior to first dose of study medication' is equal to 'no', then TEAE.<br>If AE start date = Study drug start date and the variable '...prior to first dose of study medication' is equal to 'yes', then not TEAE.
Partial, but known components show that event cannot have started during treatment period up to and including 14 days after last study drug administration	Not TEAE according to definition.
Partial, but known components show that event could have started during treatment period up to and including 14 days after the last study drug administration (or 'Did the adverse event occur prior to first dose of study medication' is indicated as 'yes')	If AE start date < Reference start date or AE start date > Reference end date + 14 days, then not TEAE.<br>If Reference start date ≤ AE start date ≤ Reference end date + 14 days, then TEAE.<br>Else assumed TEAE.

APPENDIX 3. PARTIAL MEDICATIONS DATE CONVENTIONS

Date imputations for partial medications are only used for classification of concomitant medications and partial dates will be displayed in the applicable by-subject data listings.

Algorithm for Concomitant Medications (CM)

Start Date	Stop Date	Action
Known	Known or ongoing or partial	If CM start date < Reference start date and CM end date >= Reference start date or ongoing then CM.<br>If CM start date is >= Reference start date and CM start date <= Reference end date + 14 days then CM.<br>Else not CM.
Partial, but known components show that medication could have started during treatment period up to and including 14 days after the last study drug administration	Known, ongoing or partial	Assumed CM.
Partial, but known components show that medication could not have started during treatment period up to and including 14 days after the last study drug administration	Known or ongoing or partial	If CM start date < Reference start date and CM end date (or CM end date is partial and known components show that medicaiton could have ended during the treatment period up to and including 14 days ater the last study drug administration) >= Reference start date or ongoing then CM.<br>Else not CM.

APPENDIX 4. LABORATORY TESTS

Laboratory Category	Laboratory Test	Standard Unit
Chemistry	Alanine Aminotransferase	U/L
Chemistry	Albumin	g/L
Chemistry	Alkaline Phosphatase	IU/L
Chemistry	Aspartate Aminotransferase	U/L
Chemistry	Bicarbonate	mmol/L
Chemistry	Calcium Corrected for Albumin	mmol/L
Chemistry	Chloride	mmol/L
Chemistry	Creatinine	umol/L
Chemistry	Creatinine Kinase MB	ug/L
Chemistry	Creatinine Phosphikinase	IU/L
Chemistry	Direct Bilirubin	umol/L
Chemistry	Gamma Glutamyl Transferase	U/L
Chemistry	Glucose	mmol/L
Chemistry	Indirect Bilirubin	umol/L
Chemistry	Lactate Dehydrogenase	U/L
Chemistry	Lipase	U/L
Chemistry	Magnesium	mmol/L
Chemistry	Phosphate	mmol/L
Chemistry	Potassium	mmol/L
Chemistry	Serum Urea	mmol/L
Chemistry	Sodium	mmol/L
Chemistry	Total Amylase	U/L
Chemistry	Total Bilirubin	umol/L
Chemistry	Total Protein	g/L
Chemistry	Uric Acid	umol/L
Hematology	Basophils	%
Hematology	Basophils Absolute Count	10^9/L
Hematology	Eosinophils	%
Hematology	Eosinophils Absolute Count	10^9/L
Hematology	Hematocrit	%
Hematology	Hemoglobin	gm/dL
Hematology	Lymphocytes	%

Laboratory Category	Laboratory Test	Standard Unit
Hematology	Lymphocytes Absolute Count	10^9/L
Hematology	Monocytes	%
Hematology	Monocytes Abolsute Count	10^9/L
Hematology	Neutrophils	%
Hematology	Neutrophils Absolute Count	10^9/L
Hematology	Platelets	10^9L
Hematology	Red Blood Cells	10^9L
Hematology	White Blood Cell Count	10^9L
Urinalysis	Bilirubin	Not Applicable
Urinalysis	Creatinine	umol/L
Urinalysis	Glucose	Not Applicable
Urinalysis	Ketones	Not Applicable
Urinalysis	Leukocytes	/HPF
Urinalysis	Microalbumin	g/L
Urinalysis	Nitrite	Not Applicable
Urinalysis	pH	Not Applicable
Urinalysis	Protein	Not Applicable
Urinalysis	Sodium	mmol/L
Urinalysis	Specific Gravity	Not Applicable
Urinalysis	Urobilinogen	Not Applicable

APPENDIX 5: DIVISION OF MICROBIOLOGY AND INFECTIOUS DISEASE (DMID) LABORATORY TESTS TOXICITY

Chemistry				
	**Grade 1**	**Grade 2**	**Grade 3**	**Grade 4**
Hyponatremia (Sodium)<br>mEq/L<br>mmol/L	 130 – 135<br>130 – 135	 123 – 129<br>123 – 129	 116 - 122<br>116 – 122	 < 116<br>< 116<br>Or abnormal sodium with mental status changes or seizures
Hypernatremia (Sodium)<br>mEq/L<br>mmol/L	 146 – 150<br>146 – 150	 151 – 157<br>151 – 157	 158 – 165<br>158 – 165	 > 165<br>> 165<br>Or abnormal sodium with mental status changes or seizures
Hypokalemia (Potassium)<br>mEq/L<br>mmol/L	 3.0 – 3.4<br>3.0 – 3.4	 2.5 – 2.9<br>2.5 – 2.9	 2.0 – 2.4<br>2.0 – 2.4<br>Or intensive replacement therapy or hospitalization required	 < 2.0<br>< 2.0<br>Or abnormal potassium with paresis, ileus or life-threatening arrhythmia
Hyperkalemia (Potassium)<br>mEq/L<br>mmol/L	 5.6 – 6.0<br>5.6 – 6.0	 6.1 – 6.5<br>6.1 – 6.5	 6.6 – 7.0<br>6.6 – 7.0	 > 7.0<br>> 7.0<br>Or abnormal potassium with life-threatetning arrhythmia
Hypoglycemia (Glucose)<br>mg/dL<br>mmol/L	 55- 64<br>3.0525 – 3.552	 40 – 54<br>2.22 – 2.997	 30 -39<br>1.665 – 2.1645	 < 30<br>< 1.665<br>Or abnormal glucose with mental status changes or coma
Hyperglycemia (Glucose)<br>mg/dL<br>mmol/L	 116 – 160<br>6.438 – 8.88	 161 – 250<br>8.9355 – 13.875	 251 – 500<br>13.9305 – 27.75	 > 500<br>> 27.75

Chemistry				
	Grade 1	Grade 2	Grade 3	Grade 4
				Or abnormal glucose with ketoacidosis or seizures
Hypocalcemia (Calcium corrected for Albumin)<br>mg/dL<br>mmol/L	7.8– 8.4<br>1.95 – 2.1	7.0 – 7.7<br>1.75 – 1.925	6.1 – 6.9<br>1.525 – 1.725	< 6.1<br>< 1.525<br>Or abnormal calcium with life threatetning arrythmia or tetany
Hypercalcemia (Calcium corrected for Albumin)<br>mg/dL<br>mmol/L	10.6 – 11.5<br>2.65 – 2.875	11.6 – 12.5<br>2.9 – 3.125	12.6 – 13.5<br>3.15 – 3.375	> 13.5<br>> 3.375<br>Or abnormal calcium with life threatening arrythmia
Hypomagnesemia (Magnesium)<br>mEq/L<br>mmol/L	1.2 – 1.4<br>0.6 – 0.7	0.9 – 1.1<br>0.45 – 0.55	0.6 – 0.8<br>0.3 – 0.4	< 0.6<br>< 0.3<br>Or abnormal magnesium with life-threatening arrythmia
Hypophosphatemia (Phosphate)<br>mg/dL<br>mmol/L	2.0 – 2.4<br>0.646 – 0.7752	1.5 – 1.9<br>0.4845 – 0.6137<br>Or replacement Rx required	1.0 – 1.4<br>0.323 - 0.4522<br>Intensive therapy or hospitalization required	< 1.0<br>< 0.323<br>Or abnormal phsophate with life-threatening arrythmia
Hyperbilirubinemia (when accompanied by any increase in other liver function test)	1.1 - < 1.25 x ULN	1.25 - <1.5 x ULN	1.5 – 1.75 x ULN	> 1.75 x ULN
Hyperbilirubinemia (when other liver function are in the normal range)	1.1 - < 1.5 x ULN	1.5 - <2.0 x ULN	2.0 – 3.0 x ULN	> 3.0 x ULN

Chemistry				
	**Grade 1**	**Grade 2**	**Grade 3**	**Grade 4**
BUN	1.25 - 2.5 x ULN	2.6 - 5 x ULN	5.1 - 10 x ULN	> 10 x ULN
Hyperuricemia (Uric Acid)<br>mg/dL<br>umol/L	 7.5 – 10.0<br>446.1 – 594.8	 10.1 – 12.0<br>600.75 – 713.76	 12.1 – 15.0<br>719.71 – 892.2	 > 15.0<br>> 892.2
Creatinine	1.1 - 1.5 x ULN	1.6 - 3.0 x ULN	3.1 - 6 x ULN	> 6 x ULN or dialysis required
AST (SGOT)	1.1 - < 2.0 x ULN	2.0 - <3.0 x ULN	3.0 – 8.0 x ULN	> 8.0 x ULN
ALT (SGPT)	1.1 - < 2.0 x ULN	2.0 - <3.0 x ULN	3.0 – 8.0 x ULN	> 8.0 x ULN
GGT	1.1 - < 2.0 x ULN	2.0 - <3.0 x ULN	3.0 – 8.0 x ULN	> 8.0 x ULN
Alkaline Phosphatase	1.1 - < 2.0 x ULN	2.0 - <3.0 x ULN	3.0 – 8.0 x ULN	> 8.0 x ULN
Amylase	1.1 - 1.5 x ULN	1.6 - 2.0 x ULN	2.1 – 5.0 x ULN	> 5.1 x ULN
Lipase	1.1 - 1.5 x ULN	1.6 - 2.0 x ULN	2.1 – 5.0 x ULN	> 5.1 x ULN

Hematology				
	**Grade 1**	**Grade 2**	**Grade 3**	**Grade 4**
Hemoglobin (gm/dL)	9.5 – 10.5	8.0 – 9.4	6.5 – 7.9	< 6.5
Neutrophils Absolute Count<br>/mm^3<br>10^9/L	 1000 – 1500<br>1.0 – 1.5	 750 – 999<br>0.75 – 0.99	 500 – 749<br>0.5 – 0.749	 < 500<br>< 0.5
Platelets<br>/mm^3<br>10^9/L	 75000 – 99999<br>75 – 99.99	 50000 – 74999<br>50 – 74.99	 20000 – 49999<br>20 – 49.99	 < 20000<br>< 20
White Blood Cell Count<br>/mm^3<br>10^9/L	 11000 – 13000<br>11.0 – 13.0	 13000 – 15000<br>13.0 – 15.0	 15000 – 30000<br>15.0 – 30.0	 > 30000 or < 1000<br>> 30 or < 10
% Polymorphonuclear Leucocytes + Band Cells	> 80%	90 – 95%	> 95%	Not Applicable
Abnormal Fibrinogen	Low:<br>100-200 mg/dL<br>High:<br>400-600 mg/dL	Low:<br><100 mg/dL<br>High:<br>>600 mg/dL	Low:<br>< 50 mg/dL	Fibrinogen associated with gross bleeding or with disseminated

Hematology				
	Grade 1	Grade 2	Grade 3	Grade 4
				coagulation
Fibrin Split Product	20-40 mcg/ml	41-50 mcg/ml	51-60 mcg/ml	> 60 mcg/ml
Prothrombin Time (PT)	1.01 - 1.25 x ULN	1.26-1.5 x ULN	1.51 -3.0 x ULN	>3 x ULN
Activated Partial Thromboplastin (APPT)	1.01 -1.66 x ULN	1.67 - 2.33 x ULN	2.34 - 3 x ULN	> 3 x ULN
Methemoglobin	5.0 - 9.9 %	10.0 - 14.9 %	15.0 - 19.9%	> 20.0 %

Urinalysis				
	Grade 1	Grade 2	Grade 3	Grade 4
Proteinuria (Protein)	1+ or 200 mg - 1 mg loss/day	2 - 3+ or 1 - 2 mg loss/day	4+ or 2 - 3.5 mg loss/day	Nephroticsyndrome or > 3.5 mg loss/day
Hematuria	microscopic only <10 rbc/hpf	gross, no clots >10 rbc/hpf	gross, with or without clots, OR red blood cell casts	obstructive or required transfusion

APPENDIX 6: VITAL SIGNS NOTABLE ABNORMALITIES

Vital Signs					
	**Abnormally Low**	**Grade 1/ Mild**	**Grade 2/ Moderate**	**Grade 3/ Severe**	**Grade 4/ Abnormally High**
Diastolic Blood Pressure	≤ 50 mmHg	> 90 mmHg- <100 mmHg	≥ 100 mmHg- <110 mmHg	≥ 110 mmHg	Not Applicable
Systolic Blood Pressure	≤ 90 mm Hg	> 140 mmHg- <160 mmHg	≥ 160 mmHg- <180 mmHg	≥ 180 mmHg	Not Applicable

SAFETY STATISTICAL ANALYSIS PLAN

FINAL AND INTERIM ANALYSES

NiX-TB-(B-L-Pa)

A PHASE 3 OPEN-LABEL TRIAL ASSESSING THE SAFETY AND EFFICACY OF BEDAQUILINE PLUS PRETOMANID PLUS LINEZOLID IN SUBJECTS WITH PULMONARY INFECTION OF EITHER EXTENSIVELY DRUG-RESISTANT TUBERCULOSIS (XDR-TB) OR TREATMENT INTOLERANT/NON-RESPONSIVE MULTI-DRUG RESISTANT TUBERCULOSIS (MDR-TB)

AUTHOR: Louise van Aswegen

VERSION NUMBER AND DATE: Version 2.0, 14NOV2017

STATISTICAL ANALYSIS PLAN SIGNATURE PAGE

	Name	Signature	Date
**Author:**	Louise van Aswegen	[signature]	15NOV2017
**Position:**	Senior Biostatistician		
**Company:**	QuintilesIMS, South Africa		

The signatures below indicate review and approval of the proposed final and interim safety analyses and presentation of data as planned for protocol NiX-TB-(B-L-Pa) Version 1.0, dated 21 April 2014, and protocol amendments 01, dated 18 March 2015 (incorporated into the working protocol Version 2.0, dated 18 March 2015), 02, dated 22 January 2016 (incorporated into the working protocol Version 3.0, dated 22 January 2016) and 03, dated 24 April 2017 (incorporated into the working protocol Version 4.0, dated 24 April 2017).

This version of the SAP was approved by the undersigned.

**Approved By:**	Daniel Everitt	DocuSigned by: [signature] Signer Name: Dan Everitt Signing Reason: I approve this document Signing Time: 11/15/2017 12:59:02 PM EST 32534894D9294A59B14B10FC37E90452	November 15, 2017 | 12:59 P
**Position:**	Vice President and Senior Medical Officer		
**Company:**	Global Alliance for TB Drug Development		
**Approved By:**	Joanna Moreira	DocuSigned by: [signature] Signer Name: Joanna Moreira Signing Reason: I have reviewed this document Signing Time: 11/15/2017 2:26:32 PM EST D7909ECEE1CF40F696C102E04042B149	November 15, 2017 | 2:26 PM
**Position:**	Clinical Manager, Clinical Operations		
**Company:**	Global Alliance for TB Drug Development		

MODIFICATION HISTORY

Unique Identifier for this Version	Date of Document Version	Author	Significant Changes from Previous Authorized Version
1.0	06July2017	Louise van Aswegen	Not applicable. First authorized version.
2.0	14Nov2017	Louise van Aswegen	Made updates supporting the updated made to the output templates Version 2.0

TABLE OF CONTENTS

Page:

Statistical Analysis Plan Signature Page 2
Modification History 3
Table of Contents 4
1. Introduction 7
2. Study Objectives 7
3. Study Design 8
3.1. General Description 8
3.2. Schedule of Events 10
3.3. Changes/Clarifications to Analysis from Protocol 10
4. Planned Analyses 12
4.1. Data Safety and Monitoring Committee Analysis 13
4.2. Interim Analyses 13
4.3. Final Analysis 14
5. Analysis Populations 15
5.1. Re-screened Subjects 16
5.2. All Enrolled Analysis Population 16
5.3. Intent to Treat (ITT) Analysis Population 16
5.4. Safety Analysis Population 16
6. General Considerations 17
6.1. Reference Start Date and Study Day 17
6.2. Baseline 17
6.3. Treatment-emergent Incidence 18
6.4. Unscheduled Visits 18
6.5. End of Treatment/End of Study 19
6.6. Windowing Conventions (only applicable to the first ia) 19
6.7. Statistical Tests 21
6.8. Common Calculations 23
6.9. Software Version 23
7. Statistical Considerations 23
7.1. Multicenter Studies 23
7.2. Multiple Comparisons/Multiplicity 23
7.3. Missing Data 24
7.4. Examination of Subgroups 24
8. Output Presentations 24
9. Disposition and Premature Discontinuation 25
9.1. Analysis 25

9.2. Derivations 26
10. protocol Deviations 26
11. Demographic Characteristics 27
11.1. Derivations 28
12. Baseline Characteristics 28
13. Disease History 28
13.1. Derivations 29
14. Chest X-Ray at Screening 30
15. Ophthalmology History 30
16. Drug Resistance History 31
17. Baseline Drug Susceptibility 32
18. Concomitant Medications 32
19. Concomitant Procedures 33
20. Medical History 34
21. Study Drug Exposure 34
21.1. Derivations 37
22. Efficacy Analysis (only applicable to the first ia) 38
22.1. Primary Efficacy 38
22.1.1. Derivations 38
22.1.2. Definitions 41
22.1.3. Primary Efficacy Endpoint 42
22.1.4. Analysis Methods 42
22.2. Other Secondary Efficacy 44
22.2.1. Other Secondary Efficacy Endpoints and Definitions 44
22.2.2. Tuberculosis Symptoms 44
22.2.3. Subject Reported Health Status: EQ-5D-5L 45
22.2.4. Weight 48
23. Safety Outcomes 49
23.1. Adverse Events 49
23.1.1. Severity 50
23.1.2. Relationship 50
23.1.3. Action Taken with Study Drug 51
23.1.4. Adverse Events (AEs) Leading to Death 52
23.1.5. Serious Adverse Events (AEs) 52
23.1.6. Adverse Events (AEs) Leading to Discontinuation of Study Drug 52
23.1.7. All Treatment-emergent AEs (TEAEs) 53
23.1.8. Adverse Events (AEs) of Special Interest 53
23.1.1. Liver-Related AEs 55
23.1.2. Derivations .... Error! Bookmark not defined.
23.2. Laboratory Tests 55

23.2.1. Division of Microbiology and Infectious Disease (DMID) Grading for Laboratory Data .55
23.2.2. Liver-Related Abnormalities and Evaluation if Drug-Induced Serious Hepatotoxicity ...57
23.2.3. Derivations....58
23.3. Vital Signs....58
23.3.1. Notable Abnormalities 59
23.4. Electrocardiogram Parameters 60
23.4.1. Electrocardiogram Prolongation....60
23.5. Ophthalmological Examination....61
23.6. Slit Lamp Examination....62
23.7. Peripheral Neuropathy....63
23.7.1. Derivations....64
24. List of References....66
APPENDIX 1. Programming Conventions for Tables, By-subject Data Listings and Figures.....67
APPENDIX 2. Partial Adverse Event Date Conventions 71
APPENDIX 3. Partial Medications Date Conventions 72
APPENDIX 4. Laboratory Tests....73
Appendix 5: Division of Microbiology and Infectious Disease (DMID) Laboratory Tests Toxicity.......75
Appendix 6: Vital Signs Notable Abnormalities 79

1. INTRODUCTION

This document describes the rules and conventions that will be used in the planned presentation and analysis of safety and efficacy data performed by QuintilesIMS according to protocol NiX-TB-(B-L-Pa) for the final and the interim analyses (IA) as set out in the relevant versions of the output templates [Interim Analysis Output Templates Version 1.0 and Nix-TB-(B-L-Pa) Final and Interim Analysis Output Templates Version 1.0 and 2.0]. The pharmacokinetic (PK) analysis of this study will be described in separate statistical analysis plans (SAPs). From the second IA onwards, the fifth data safety monitoring committee (DSMC) onwards and the final analysis, Medical Research Council (MRC) Clinical Trials Unit (CTU) at University College London (UCL) (MRC CTU at UCL) will be responsible for the efficacy, which will be described in a separate SAP as set up by MRC CTU at UCL. It describes the data that will be summarized and analyzed, including specifics of the statistical analyses that will be performed.

QuintilesIMS will be responsible for the DSMC analyses including safety and efficacy data for the DSMC meetings 1, 2, 3 and 4. For all subsequent DSMC analyses, QuintilesIMS will only be responsible for the safety data and MRC CTU at UCL will be responsible for the efficacy data. For the first IA, QuintilesIMS will be responsible for the safety and efficacy data. For the subsequent IAs and the final analysis, QuintilesIMS will only be responsible for the safety data and MRC CTU at UCL will be responsible for the efficacy data.

This SAP is based on protocol Version 1.0, dated 21 April 2014, and protocol amendments 01, dated 18 March 2015 (incorporated into the working protocol Version 2.0, dated 18 March 2015) , 02, dated 22 January 2016 (incorporated into the working protocol Version 3.0 dated 22 January 2016) and 03, dated 24 April 2017 (incorporated into the working protocol Version 4.0 dated 24 April 2017).

2. STUDY OBJECTIVES

The objective of this study is to evaluate the efficacy, safety, tolerability and PK of Bedaquiline (B) plus Pretomanid (Pa) plus Linezolid (L) (B-L-Pa) after 6 months of treatment (with an option to treat for 9 months in subjects who are still culture positive at Month 4) in subjects with either pulmonary extensive drug-resistant (XDR) tuberculosis (TB), treatment intolerant or non-responsive multi-drug resistant (MDR) TB (MDR-TB).

The primary endpoint of this study is:

- Incidence of bacteriologic failure or relapse or clinical failure through follow up until 6 months after the End of Treatment.

The secondary endpoints of this study are:

- Incidence of bacteriologic failure or relapse or clinical failure through follow up until 24 months after the End of Treatment as a confirmatory analysis.
- Time to sputum culture conversion to negative status through the treatment period.
- Proportion of subjects with sputum culture conversion to negative status at 4, 6, 8, 12, 16 and 26 or 39 weeks.
- Linezolid dosing (actual) and efficacy will be explored.
- Change from Baseline TB symptoms.
- Change from Baseline in Patient Reported Health Status.
- Change from Baseline weight.

The exploratory endpoints and analyses of this study are:

- Evaluate whether any of the secondary endpoints predicts relapse free cure.
- Sub-analysis of populations by Human Immunodeficiency Virus (HIV) status and CD4 count.
- Correlation of Time over mitochondrial protein synthesis inhibition (MPS50) with Linezolid toxicity (the MPS50 value will be an assumed value from literature).

3. STUDY DESIGN

3.1. GENERAL DESCRIPTION

Up to 200 male and female subjects aged 14 years and older, with confirmed sputum positive for *Mycobacterium tuberculosis* (M.tb.) in culture pulmonary XDR-TB, or with pulmonary MDR-TB with a documented intolerability or non-response to the best treatment available for 6 months or more will be enrolled.

- After a maximum of 9 days for Screening, all subjects will receive 6 months of treatment. Subjects will have follow-up visits 1 and 2 months after treatment completion, and thereafter every 3 months, up to Month 24. If a subject is still culture positive by Month 4, they may have treatment extended to 9 months (with 24 months of follow-up) or be withdrawn from the study.
- Subjects who withdraw after ≤ 14 days of treatment should attend an early withdrawal visit. Subjects who withdraw after ≥ 15 days of treatment should return for an early withdrawal visit and follow-up visits at 3, 12 and 24 months after their last dose of treatment (to confirm survival status, occurrence of serious adverse event [SAEs] and resolution of TB symptoms).

A schematic of the study design is presented below.

Figure 1: Study Schematic

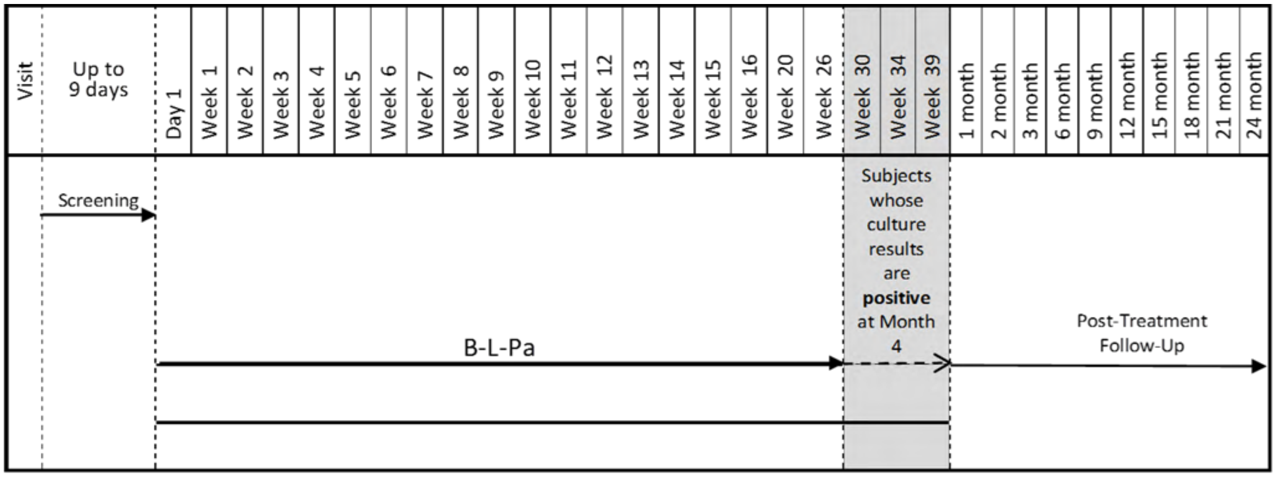

All subjects will receive B-L-Pa for the duration of treatment as follows:

Table 1: Study Drug Administration

Study Drug	Dose to be Administered Orally
Bedaquiline	400 mg once daily for Day 1 to Day 14 then<br>200 mg three times per week for remainder of treatment
Pretomanid	200 mg once daily
Linezolid	600 mg twice a day (BID) (Protocol Version 1 and 2)<br>1200 mg once a day (QD) (Protocol Version 3)<br>If adverse events develop, the Investigator can interrupt or reduce the dose to either 600 mg QD or 300 mg QD<br>If subjects have toxicity issues with Linezolid that would prohibit further treatment with that drug, they can remain on Bedaquiline and Pretomanid if they received the initial 1200 mg total daily dose of Linezolid for at least 4 consecutive weeks of treatment and they are smear negative or with trace/scanty results and judged to be clinically improving by the Investigator

3.2. Schedule of Events

The schedule of events can be found in Section 1.2: Trial Flow Chart of the protocol.

3.3. Changes/Clarifications to Analysis from Protocol

The definition of treatment-emergent adverse events (TEAEs) changed from:

- AEs which started or worsened on or after the first study drug administration until the last scheduled visit/assessment/measurement will be regarded as treatment-emergent.

to

- AEs which started or worsened on or after the first study drug administration up to 14 days after the last study drug administration.

For analysis of culture data (except for stable negative conversion) for DSMC 1 to 4 and IA 1, the derived result at Baseline will be considered positive for all subjects regardless of culture results derived per sample. This is due to the fact that the inclusion criteria stipulate that subjects with one of the following pulmonary TB conditions:

- XDR-TB with:
    - Documented culture positive (for M.tb.) results within 3 months prior to Screening or M.tb. confirmed in sputum based on molecular test within 3 months prior to or at Screening.
    - Historical documented resistance to Isoniazid, Rifamycins, a Fluoroquinolone and an injectable at any time.
- MDR-TB documented by culture positive results (M.tb.) within 3 months prior to Screening with documented non-response to treatment with the best available regimen for 6 months or more prior to enrollment who in the opinion of the Investigator have been adherent to treatment and will be adherent to study regimen.
- MDR-TB documented by culture positive (for M.tb.) results within 3 months prior to Screening who are unable to continue second line drug regimen due to a documented intolerance to:
    - PAS, Ethionamide, Aminoglycosides or Fluoroquinolones;
    - Current treatment not listed above that renders subject eligible for the study in the Investigator's opinion.

The study protocol stipulates that the first IA will be conducted when the first 15 subjects reach Month 6 after completion of treatment. The first IA will however be conducted after the 15th subject has completed Month 6 visit OR prematurely discontinued study drug. The list of the 15 subjects will be provided by TB Alliance.

The following IAs will be cumulative including the next 15 subjects or more whom completed the Month 6 visit or prematurely discontinued the study drug as identified by TB Alliance.

4. Planned Analyses

The following formal analyses will be performed for this study:

Analysis	Company Responsible	Details/Comments	Domains
Data Safety and Monitoring Committee Analyses (DSMC)			
DSMC 1 to 4	QuintilesIMS	Safety and Efficacy	All
DSMC 5 onwards	QuintilesIMS	Safety	All applicable domains excluding efficacy
DSMC 5 onwards	MRC CTU at UCL	Efficacy	Culture Data<br>Tuberculosis Symptoms<br>Subject Reported Health Status: EQ-5D-5L<br>Weight
Interim Analyses (IA)			
IA 1	QuintilesIMS	Safety and Efficacy	
IA 2 onwards	QuintilesIMS	Safety	All applicable domains excluding efficacy
IA 2 onwards	MRC CTU at UCL	Efficacy	Culture Data<br>Tuberculosis Symptoms<br>Subject Reported Health Status: EQ-5D-5L<br>Weight
Final Analysis	QuintilesIMS	Safety	All applicable domains excluding efficacy
Final Analysis	MRC CTU at UCL	Efficacy	Culture Data<br>Tuberculosis Symptoms<br>Subject Reported Health Status: EQ-5D-5L<br>Weight

4.1. Data Safety and Monitoring Committee Analysis

Interim safety and efficacy data will be reviewed by the DSMC as follows:

- At least every 6 months after the first subject is enrolled.
- *Ad hoc* meetings can be requested by TB Alliance or the DSMC based on rates of SAEs or if safety concerns arise during the study.

The DSMC analyses and output templates are described in a separate document [Nix-TB-(B-L-Pa) DSMC Output Template].

4.2. Interim Analyses

Each interim analysis will be performed for every 15 subjects completing/prematurely discontinuing study drug. The timing of the first interim analysis will be after the 15th subject has completed Month 6 visit. The list of the 15 subjects or more, will be provided by TB Alliance for each IA. Data will be presented cumulatively.

The first IA was planned following:

- QuintilesIMS Biostatistics (QIMS BIOS) review and acceptance of the Study Data Tabulation Model (SDTM) datasets received from eClinical Solutions (eCS).
- TB Alliance authorization of the IA output templates. TB Alliance confirmed via email that they were in agreement with the output templates to be used for the first IA.
- TB Alliance review of TB medications and AEs of special interest.
- TB Alliance review and authorization of the analysis populations.
- QuintilesIMS BIOS review of the database and subsequently providing data issues to Data Management via the Data Issues Log.

The second IA onwards is planned following:

- QuintilesIMS Biostatistics (QIMS BIOS) and MRC CTU at UCL review and acceptance of the Study Data Tabulation Model (SDTM) datasets received from eClinical Solutions (eCS).
- TB Alliance authorization of the final and interim analyses version of the SAP and output templates.
- TB Alliance review of TB medications and AEs of special interest.
- TB Alliance review and authorization of the analysis populations.
- QuintilesIMS BIOS review of the database and subsequently providing data issues to Data Management via the Data Issues Log.

The IA will be performed on a clean database:

- All outstanding data issues and queries resolved for the identified subjects and visits.
- All unresolvable data issues documented in the DHR from Data Management.
- All coding of medications, medical conditions, physical examination abnormalities and AEs completed.
- Reconciliation of the following data with electronic case report form (eCRF) data completed successfully:
    - Safety laboratory data.
    - Serious adverse events (SAEs).

4.3. Final Analysis

The final analysis will be performed after all subjects have completed Month 24 visit after the End of Treatment.

The final analysis is planned following:

- QuintilesIMS Biostatistics (QIMS BIOS) and MRC CTU at UCL review and acceptance of the SDTM datasets received eCS.
- TB Alliance authorization of the final and interim analyses version of the SAP and output templates.
- TB Alliance review of TB medications and AEs of special interest.
- TB Alliance review and authorization of the analysis populations.
- QuintilesIMS BIOS review of the database and subsequently data issues provided to Data Management via the Data Issues Log.
- QuintilesIMS BIOS review and authorization of DHR.

The final analysis will be performed on a clean database:

- All outstanding data issues and queries resolved.
- All unresolvable data issues documented in the DHR from Data Management.
- All coding of medications, medical conditions, physical examination abnormalities and AEs completed.
- Reconciliation of the following data with case report form (eCRF) data completed successfully:
    - Safety laboratory data.
    - Serious adverse events (SAEs).

5. Analysis Populations

Analysis populations established by QuintilesIMS for the IA and final analysis will be determined programmatically based on the definitions listed below.

The Intent to treat (ITT), modified ITT (MITT) and per-protocol (PP) analysis populations will be determined by MRC CTU at UCL for IA 2 onwards and the final analysis, and they will be providing the assignment on to QuintilesIMS. Refer to the efficacy SAP for these analysis population definitions.

5.1. Re-screened Subjects

Re-screened subjects will be identified by means of the re-screened flag in the (SDTM.SC) dataset (SDTM.SC.SCORRES = 'Y' when SDTM.SC.SCTESTCD = 'RESCREEN'). Subjects will only be accounted for once and therefore the records related to the original subject number for re-screened subjects will be excluded from all analysis.

5.2. All Enrolled Analysis Population

This analysis population will include all subjects who have provided informed consent.

5.3. Intent to Treat (ITT) Analysis Population

For the first IA, the Intent to Treat (ITT) analysis population comprised all subjects who were included in the All Enrolled analysis population that were assigned to study drug.

For subsequent IAs and the final analysis, refer to the efficacy SAP for details.

5.4. Safety Analysis Population

For the first IA the Safety analysis population was defined as all subjects included in the ITT analysis population and received at least one administration of study drug.

For all subsequent IAs and the final analysis, the Safety analysis population will include all subjects who receive at least one administration of study drug.

Therefore a subject will be programmatically included in the Safety analysis population if the subject has at least one date of study drug administration available in the SDTM data (SDTM.EX.EXSTDTC).

The Safety analysis population will be used as primary analysis set for all safety analysis.

The ITT and the Safety analysis populations were used for all analysis (safety and efficacy) of the first IA.

6. General Considerations

6.1. Reference Start Date and Study Day

Study day will be calculated relative to the reference start date which will be used to present relative start/stop days of assessments/events. The reference start date (Day 1) is defined as the earliest (minimum) date of first study drug administration of the three study drugs (B, Pa, L). The reference end date is defined as the latest (maximum) date of last study drug administration of the three drugs.

- If the date of assessment/event is on or after the reference start date then:
  - Study day = (Date of assessment/event – Reference start date) + 1.
- If the date of assessment/event is prior to the reference start date then:
  - Study day = (Date of assessment/event – Reference start date).

In the case where the assessment/event date is partial or missing, for which no imputation rules apply, study day, and any corresponding durations will be presented as missing in the by-subject data listings.

6.2. Baseline

Unless stated otherwise, Baseline is defined as the last available non-missing assessment (scheduled or unscheduled) prior to or on Day 1. In the case where the last available non-missing assessment and the reference start date coincide, the assessment will be considered pre-dose. For example, if vital signs or laboratory assessments fall on the date of first study drug administration and the time of the assessment is not available, the applicable assessment will be considered as Baseline. However, medications commencing on the date of first study drug administration will be considered post-baseline, i.e. concomitant medications. For AEs, the answer to the question 'Did the adverse event occur prior to first dose of study medication' will also be taken into consideration when determining if the event can be regarded as treatment-emergent or not.

For the culture data for IA 1, Baseline is defined as the last available non-missing assessment (scheduled or unscheduled) prior to or on Day 1 or any positive culture result between Screening and Week 4. For all analysis purposes (except for stable culture negative conversion), the derived

result at Baseline will be considered positive for all subjects regardless of culture results derived per sample.

For the Baseline drug susceptibility data, Baseline is defined as the worst available non-missing assessment (scheduled or unscheduled) prior to or on Day 1 or any resistant result between Screening and Week 4, according to the following hierarchies, per method and drug:

- Resistant, sensitive, susceptible, contaminated, indeterminate, missing

For analysis purposes, a separate Baseline visit will be presented for each subject, where applicable, in by-subject data listings.

Post-baseline is defined as any assessment (scheduled or unscheduled) after Baseline (per data domain as described above).

6.3. Treatment-emergent Incidence

A treatment-emergent incidence is defined as any event (TEAEs, liver-related abnormalities, QT interval prolongation) (scheduled or unscheduled) which started after the first study drug administration up to 14 days (gap period) after the last study drug administration.

A post-treatment incidence is defined as any event (AEs, liver-related abnormalities, QT interval prolongation) (scheduled or unscheduled) which started after 14 days (gap period) after the last study drug administration.

6.4. Unscheduled Visits

For all safety analyses, data recorded at the scheduled visit will be presented in by-visit summaries. Unscheduled assessments will not be included in by-visit summaries, but will be included in by-subject data listings. Unscheduled visits will also be considered for Baseline and End of Treatment/End of Study values as well as for determining treatment-emergent incidence. Unscheduled visits will be sorted in a chronological order within subject in the by-subject data listings.

6.5. END OF TREATMENT/END OF STUDY

End of Treatment is defined as the non-missing post-baseline assessment (scheduled or unscheduled) closest to the last study drug administration target date/day within the period following the first study drug administration up to and including 14 days after the last study drug administration. Where an assessment prior to the last study drug administration date and an assessment after the last study drug administration date are equidistant from the target date/day, the result prior to the last study drug administration date will be regarded as End of Treatment.

End of Study is defined as the last non-missing post-baseline assessment (scheduled or unscheduled) assigned in the study and will only be presented when applicable.

6.6. WINDOWING CONVENTIONS (ONLY APPLICABLE TO THE FIRST IA)

The derived culture result per visit is based on an early morning and a spot sputum sample taken on the applicable scheduled visit. If one of the two results is missing at the scheduled visit the following steps should be followed:

- Check whether there is an unscheduled assessment available before or after the applicable scheduled visit. If there is no unscheduled assessment then the single sputum sample will be used to derive the culture result per visit.
- If an unscheduled assessment prior to the applicable scheduled visit is available; check if the previous scheduled visit had both sputum samples (spot and early morning) available. If the unscheduled assessment occurred after the applicable scheduled visit; check if the next scheduled visit had both sputum samples available. If the previous/next (as applicable) scheduled visit had both sputum samples available and the unscheduled visit has the applicable missing sputum sample result available, the result should be used together with the sputum sample result to derive the culture result per visit at the scheduled visit by mapping the unscheduled visit to the applicable scheduled visit.
- If the previous/next (as applicable) scheduled visit does not have both sputum samples available, then the unscheduled assessment will be assigned to the applicable scheduled visit based on the target days in Table 2.
- Once these steps have been followed for all scheduled visits with missing sputum samples, it will be determined if a subject has all scheduled visits up to his/her study completion or

premature study discontinuation, where sputum sampling was to be performed per protocol.

- If a subject does not have all of the applicable scheduled visits, it will be checked if there are any unscheduled assessments that fall within the relevant target days for the missing scheduled visit. If this is the case, the unscheduled assessments will be mapped to the missing scheduled visit based on the target days in Table 2.

Table 2: Visit Target Days for Protocol-specified Sputum Sampling Visits

Visit Name	Target Visit Day	Start Day of Visit Window	Midpoint	End Day of Visit Window
Screening	Day -9 to 0	-9	Not applicable	0
Day 1	Day 1	1	1	1
Week 1	Day 7	2	7	11
Week 2	Day 14	12	14	21
Week 4	Day 28	22	28	35
Week 6	Day 42	36	42	49
Week 8	Day 56	50	56	70
Week 12	Day 84	71	84	98
Week 16	Day 112	99	112	126
Week 20	Day 140	127	140	161
Week 26 (subjects who completed 6 months of treatment)	Day 182	162	182	Up to 14 days after the last study drug administration
Week 26 (subjects who completed 9 months of treatment)	Day 182	162	182	196
Week 30 (subjects who completed 9 months of treatment)	Day 210	197	210	224
Week 34 (subjects who completed 9 months of treatment)	Day 238	225	238	256
Week 39 (subjects who completed 9 months of treatment)	Day 273	257	273	Up to 14 days after the last study drug administration
End of Treatment	Non-missing post-baseline assessment (scheduled or unscheduled) closest to the last study drug administration target date/day within the period following the first study drug			

Visit Name	Target Visit Day	Start Day of Visit Window	Midpoint	End Day of Visit Window
	administration up to and including 14 days after the last study drug administration.			
Month 1	Day 30	1	30	46
Month 2	Day 61	47	61	76
Month 3	Day 91	77	91	137
Month 6	Day 183	138	183	229
Month 9	Day 274	230	274	320
Month 12	Day 365	321	364	411
Month 15	Day 457	412	457	503
Month 18	Day 548	504	548	594
Month 21	Day 639	595	639	685
Month 24	Day 731	685	731	∞
End of Study	Last non-missing post-baseline assessment (scheduled or unscheduled) assigned to the study.			

For scheduled visits, multiple records may exist for a particular sputum sample (early morning or spot). A worst-case approach will be used to determine the culture result per sample, based on the following hierarchy:

- Positive.
- Negative.
- Contaminated.

6.7. STATISTICAL TESTS

Unless otherwise specified:

- The default summary statistics for quantitative variables will be as follows:
    - Number of subjects in each category (n).
    - Mean.
    - Standard deviation (SD).
    - Median.
    - Minimum.
    - Maximum.

- The default summary statistics for qualitative variables will be as follows:
  - Number of subjects in each category (n).
  - The percentages of subjects in each category (%) can be presented relative to either one of the following:
    - The total number of subjects in the relevant analysis population.
    - The total number of subjects in the relevant analysis population, with assessments available (observed cases).
  - In the event of missing assessments, a 'Missing' category showing the number of subjects with missing assessments will be presented. Percentage of subjects with missing data will not be presented.

Univariate statistics:

- Statistics should be presented in the same order across tables (i.e. n, mean, SD, minimum, median and maximum).
- If the original data has N decimal places, then the summary statistics should have the following decimal places:
  - Minimum, maximum: N.
  - Mean and median: N + 1.
  - SD: N + 2.

Frequencies and percentages (n and %):

- Percent values should be reported inside parentheses, with one space between the count and the left parenthesis of the percentage. Parentheses should be justified to accept a maximum of 100.0 as a value and padded with blank space if the percent is less than 100.0. An example is given below:
  - 77 (100.0)
  - 50 (64.9)

Confidence intervals (CIs):

- CIs should be presented with one additional decimal place as that of the raw data, and SDs and SEs with two additional decimal places as that of the raw data.

- CIs should be justified so that parentheses displayed on consecutive lines of a table 'line up'.

P-values:

- P-values should be reported to four decimal places.

Ratios:

- Ratios should be reported with one additional decimal place as that of the raw data.

6.8. Common Calculations

For quantitative assessments, the change from Baseline will be calculated as:

- Change from Baseline at Week x/Month y= (Result at Week x/Month y– Baseline).

6.9. Software Version

All analyses will be conducted using SAS® Version 9.4.

7. Statistical Considerations

7.1. Multicenter Studies

This study will be conducted by multiple Investigators at multiple study centres. All centres will be pooled for analysis purposes.

7.2. Multiple Comparisons/Multiplicity

Not applicable.

7.3. MISSING DATA

For the handling of partial dates for AEs refer to Partial Adverse Event Date Conventions of this SAP.

For the handling of partial dates for medications, refer to APPENDIX 3Partial Adverse Event Date Conventions of this SAP.

7.4. EXAMINATION OF SUBGROUPS

All outputs will be presented by current TB diagnosis at Baseline.

- Current TB diagnosis at Baseline:
    - XDR.
    - MDR (this includes MDR-TB treatment non-responsive and MDR-TB treatment intolerant).
    - Total (XDR and MDR).

8. OUTPUT PRESENTATIONS

Appendix 1: Programming Conventions for Outputs contains conventions for presentation of data in TLFs.

The output templates provided together with this SAP describe the presentations for the final and IA analyses and therefore the format and content of the TLFs to be provided by QuintilesIMS Biostatistics.

Note that verbatim terms, specifications (for example the reason a specific assessment was not done) and all variables in the TLFs containing the suffix (eCRF) contain verbatim text that may include spelling mistakes. Verbatim text will be presented in the by-subject data listings 'as is' and no manual 'hard-coding' correction of such data will be made.

The following concatenated variable will be presented in all by-subject data listings:

- Subject number, Baseline HIV status and TB type, separated out with a '/'. For example: 02-9003-003/+/XDR.

9. Disposition and Premature Discontinuation

9.1. Analysis

All subjects who provided informed consent will be accounted for in the study. Data as recorded on the Subject Disposition eCRF will be presented in a table (not applicable for the first IA analysis) and a by-subject data listing for the All Enrolled (final analysis)/ITT analysis population (IA analysis). Accounting for the key study milestones the following will be presented in the by-subject data listing:

- Date of informed consent relative to overall reference start date.
- Reason for screening failure (only applicable for the final analysis).
- Date of first study drug administration (overall reference start date) and last study drug administration (overall reference stop date).
- Treatment completion/discontinuation and the relevant primary reason for treatment discontinuation.
- Month 6 completion/discontinuation and the relevant primary reason for discontinuation.
- Month 24 completion/discontinuation and the relevant primary reason for discontinuation.
- Date of death relative to overall reference start date as well as the following information related to the death: Reason for death, relationship to TB and if the death was violent or accidental.

Subject disposition will be presented as a flowchart detailing:

- Subjects enrolled (who signed informed consent).
- Screening failures (not applicable for the IA analysis).
- Subjects in the ITT analysis population.
- Subjects who received study drug (Safety analysis population).
- Subjects identified for the IA (not applicable for the final analysis).
- Subjects in the MITT analysis population (not applicable for the first IA analysis).
- Subjects in the PP analysis population (not applicable for the first IA analysis).
- Subjects who discontinued treatment.
- Subjects who completed treatment.

- Subjects who discontinued prior to and including the Month 6 follow-up visit.
- Subjects who completed Month 6 follow-up.
- Subjects who discontinued prior to and including the Month 24 follow-up visit.
- Subjects who completed Month 24 follow-up.

A subject's treatment, Month 6 and Month 24 status (completion/discontinuation) and screening failures will be based on the Subject Disposition eCRF as recorded by the Investigator. Subjects who prematurely discontinue study participation prior or at the End of Treatment will not be regarded for the completion status at the Month 6 follow-up visit. Subjects who prematurely discontinue study participation prior or at the Month 6 follow-up visit will not be regarded for the completion status at the Month 24 follow-up visit.

Whether a subject is included in the All Enrolled analysis population and/or the Safety analysis population will be determined programmatically (refer to Section 5 Analysis Populations). The number of subjects identified for the ITT, mITT and PP analysis populations will be obtained from MRC CTU at UCL.

9.2. DERIVATIONS

Difference between first and last study drug administration (months):

- Difference (months) = ([Date of last study drug administration – date of first stuy drug administration] + 1)/(365.25/12).

10. PROTOCOL DEVIATIONS

All major protocol deviations data as recorded on the external EXCEL sheet provided by TB Alliance will be presented in a summary table for the ITT analysis population and in a by-subject data listing for the All Enrolled analysis population (final analysis)/ITT analysis population (IA analysis).

The following information will be presented in the by-subject data listing:

- Date and study day deviation noted.
- Deviation type.
    - Informed consent.

- Eligibility and entry criteria.
- Withdrawal.
- IMP administration.
- Concomitant treatment.
- Procedural (physical examinations/vital signs/PK/laboratory/cardiology/ophthalmology etc.).
- Visit schedule.
- Adverse event/specific toxicity/serious adverse event.
- Source documentation.
- Regulatory/ethics.
- Administration criteria (outside of data issues).
- Other.

- Deviation specified.

11. DEMOGRAPHIC CHARACTERISTICS

Demographic characteristics as recorded on the Demography eCRF will be presented.

With reference to Section 6 General Considerations and Section 7 Statistical Considerations of this SAP, the following data will be summarized:

- Age (years): Calculated relative to Screening.
- Gender (including childbearing potential for female subjects). Percentage (%) of childbearing potential status will be calculated relative to the total number of female subjects with data available.
    - Male.
    - Female.
- Race:
    - Asian.
    - Black or African American.
    - Native Hawaiian or other Pacific Islander.
    - White.
    - Mixed race.
    - Other.

The following will also be presented in the by-subject data listing:

- Country.

11.1. Derivations

Age (years): Calculated relative to Screening using the following SAS® code:

- Age (years) = int((intck('month', <date of birth>, <date of Screening>) - (day(<date of Screening>) < day(<date of birth>)))/12). Date of Screening to be obtained from SDTM.SV.SVSTDTC using the relevant record where SDTM.SV.VISIT = 'SCREENING'. Date of birth to be obtained from SDTM.DM.BRTHDTC.

12. Baseline Characteristics

Baseline characteristics as recorded on the Vital Signs eCRF, the Safety Laboratory Sample Collection eCRF, the Karnofsky Performance Status eCRF and the Chest X-Ray eCRF will be presented.

With reference to Section 6 General Considerations and Section 7 Statistical Considerations of this SAP, the following data will be summarized:

- Baseline height (cm).
- Baseline weight (kg).
- Baseline body mass index (BMI) (kg/m^2).
- Baseline CD4 count (cells/uL).
- Karnofsky score (%), where a score of 100 indicates normal, no complaints and 0 indicates death.

13. Disease History

Disease history as recorded on the TB and Human Immunodeficiency Virus (HIV) History eCRF will be presented.

With reference to Section 6 General Considerations and Section 7 Statistical Considerations of this SAP, the following data will be summarized:

- HIV status.
    - Negative.
    - Positive.
- Duration since HIV diagnosis (years): Calculated relative to the first study drug administration.
- Original TB diagnosis:
    - Drug-sensitive (DS).
    - Multi drug-resistant (MDR).
    - Extensively drug-resistant (XDR).
- Duration since original TB diagnosis (months): Calculated relative to the date of first study drug administration.
- Current TB diagnosis:
    - Extensively drug-resistant TB (XDR-TB).
    - Multi drug-resistant TB (MDR-TB) non-responsive.
    - Multi drug-resistant TB (MDR-TB) intolerant.
- Duration since current TB diagnosis (months): Calculated relative to the date of first study drug administration.
- Duration since most recent positive culture (days): Calculated relative to the date of first study drug administration.

13.1. DERIVATIONS

Duration since HIV (years):

- Duration (years) = Absolute value ([date of HIV diagnosis – date of first study drug administration]/]365.25]).

Duration since original/current TB diagnosis (months):

- Duration (months) = Absolute value ([date of diagnosis – date of first study drug adminisation]/]365.25/12]).

Duration since most recent positive culture (days):

- Duration (days) = Absolute value (date of most recent positive culture – date of first study drug administration).

14. Chest X-Ray at Screening

Chest X-Ray results at Screening as recorded on the Chest X-ray eCRF will be presented.

With reference to Section 6 General Considerations and Section 7 Statistical Considerations of this SAP, the following data will be summarized:

- Result.
    - Normal.
    - Abnormal.
- Compatible with TB:
    - Yes.
    - No.
- Cavities:
    - No cavities.
    - Unilateral cavities.
    - Bilateral cavities.

15. Ophthalmology History

Ophthalmology history as recorded on the Ophthalmology Medical History eCRF will be presented.

With reference to Section 6 General Considerations and Section 7 Statistical Considerations of this SAP, the following data will be summarized:

- Personal history of vision or eye disorders:
    - Yes.
    - No.
- Immediate family history of cataracts:
    - Yes. If yes, description, type and severity of cataracts.
    - No.
- Personal history or prior refractive eye surgery:
    - Yes.
    - No.
- History of eye trauma
    - Right eye:
        - Yes.
        - No.
    - Left eye:
        - Yes.
        - No.

16. Drug Resistance History

Drug resistance history as recorded on the TB and HIV History eCRF will be presented.

With reference to Section 6 General Considerations and Section 7 Statistical Considerations of this SAP, the following data will be summarized:

- Result per applicable drug:
    - Sensitive.
    - Resistant.
    - Indeterminate.
    - Missing.

17. Baseline Drug Susceptibility

Baseline drug susceptibility as recorded on the Mycobacteriology Laboratory: Mycobacteriology Characterization eCRF.

With reference to Section 6 General Considerations and Section 7 Statistical Considerations of this SAP, the following data will be summarized:

- Result per applicable drug and per applicable method:
  - Resistant.
  - Sensitive/susceptible.
  - Contaminated/indeterminate/missing.

18. Concomitant Medications

Medications as recorded on the Concomitant Medications eCRF will be coded using World Health Organization–Drug Dictionary (WHO-DD). The version of the coding dictionary will be updated at an ongoing basis, to the latest version, during the course of the study.

Medications will be classified as:

- Prior medications (P): Medications starting and ending prior to the first study drug administration.
- Concomitant medications (C): Medications taken on or after the first study drug administration up to and including 14 days after the last study drug administration or are ongoing.
- Post-treatment medications (F): Medications taken after the last study drug administration date + 14 days or are ongoing.

Concomitant medications and post-treatment medications are not mutually exclusive and a medication can therefore be classified as both C/F.

Medications of interest include:

- Anti-retroviral medications: All medications where the ATC level 1 code starts with J05AF, J05AG or J05AR.
- TB medications: All medications where the indication for the medication is recorded as XDR and/or MDR as confirmed by TB Alliance.

19. CONCOMITANT PROCEDURES

Concomitant procedures as recorded on the Concomitant Procedures eCRF will be presented.

With reference to Section 6 General Considerations and Section 7 Statistical Considerations of this SAP, the data will be summarized by System Organ Class (SOC), and Preferred Term (PT). If a concomitant procedure occurs more than once for a subject per level of summarization, the subject will only be counted once per Medical Dictionary for Regulatory Activities (MedDRA) SOC or PT. System Organ Classes will be sorted by total descending frequency. Preferred Terms (PTs) will be sorted by total descending frequency within each SOC. If SOCs or PTs have the same total frequency they will be sorted alphabetically

Partial dates will not be imputed and as such study day will be presented as missing. Uncoded concomitant procedures will be presented as applicable using the Investigator's Verbatim Term (eCRF) presented as PT within the text 'UNCODED' as SOC (only applicable for the IA).

Concomitant procedures are coded using MedDRA. The version of the coding dictionary will be updated at an ongoing basis, to the latest version, during the course of the study.

20. Medical History

Medical history as recorded on the Medical/Treatment History eCRF will be presented.

With reference to Section 6 General Considerations and Section 7 Statistical Considerations of this SAP, the following data will be summarized by SOC and PT. If a medical history condition occurs more than once for a subject per level of summarization, the subject will only be counted once per Medical Dictionary for Regulatory Activities (MedDRA) SOC or PT. System Organ Classes will be sorted by total descending frequency. Preferred Terms (PTs) will be sorted by total descending frequency within each SOC. If SOCs or PTs have the same total frequency they will be sorted alphabetically.

Partial dates will not be imputed and as such study day will be presented as missing. Uncoded medical history will be presented as applicable using the Investigator’s Verbatim Term (eCRF) presented as PT within the text ‘UNCODED’ as SOC (only applicable for the IA).

Medical history is coded using MedDRA Version. The version of the coding dictionary will be updated at an ongoing basis, to the latest version, during the course of the study.

21. Study Drug Exposure

Study drug administration data as recorded on the Study Drug Dosing eCRF, missed dose data as recorded on the Missed Doses eCRF and premature study discontinuations due to an AE as recorded on the Subject Disposition eCRF will be presented.

With reference to Section 6 General Considerations and Section 7 Statistical Considerations of this SAP, the following data will be summarized:

- Actions (study drug administration, missed doses, interruptions) that occurred simultaneously for all three drugs and separately for Linezolid, irrespective of whether it occurred in the other two drugs:
  - o Number of subjects with premature study drug administration discontinuation due to an adverse event.
  - o Number of subjects with at least one dose interruption. Interruptions less than 2 days will not be regarded as an interruption.
  - o Primary reason for dose interruption:

        - Adverse event.
        - Other.
    - Maximum duration of dose interruption due to an adverse event (days).
    - Total duration of dose interruption due to an adverse event (days).

- The following will be presented for Linezolid only, irrespective of whether it occurred in the other two drugs:
    - Number of subjects with at least one Linezolid reduction.
    - Reason for Linezolid dose reduction:
        - Adverse event.
        - Other.
    - Number of subjects with at least one Linezolid dose interruption and/or dose reduction due to an adverse event. Subjects are only counted once per category, regardless of the number of events.

- Linezolid administration will be summarized in a table, including the following (not applicable for the first IA analysis):
    - Planned cumulative dose (mg).
    - Actual cumulative dose (mg).
    - Relative dose (%).

The following will be presented as a by-subject figure for subjects who had a dose interruption and/or dose reduction and/or premature discontinuation of Linezolid due to an AE:

- Linezolid administration timeline according to the study day (x-axis) and by total daily dose (y-axis). Missed doses will be indicated by means of a green circle annotated on the figure for the duration of the missed doses and interruptions by means of a red line annotated on the figure for the duration of each interruption.
- A separate timeline for each of the other two study drugs will also be presented, similar to that of Linezolid.
- Adverse events related to relevant actions for each study drug will be displayed on the bottom section of the figure according to the relative study day (x-axis). Each AE will be displayed on a separate line and labeled as AE1 to AEx where x is the total number of AEs related to the applicable actions taken. The Preferred Term of each corresponding number and relevant severity (grade) will be listed below the contents of the figure.

A Kaplan-Meier plot will be presented for:

- Time (days) to first dose interruption and/or dose reduction of Linezolid due to AE.
- The figure will be repeated for the following subsets of subjects:
  - Subjects where the AE associated with the interruption and/or dose reduction is myelosuppression. Myelosuppresion will be identified by means of the AE System Organ Class (SOC) = BLOOD AND LYMPHATIC SYSTEM DISORDERS.
  - Subjects where the AE associated with the interruption and/or dose reduction is neuropathy. Neuropathy will be identified by means of the AE High Level Group Term (HLGT) = PERIPHERAL NEUROPATHIES.

The LIFETEST procedure in SAS® is a non-parametric procedure for analyzing survival data to compute the Kaplan-Meier plot, which is a non-parametric maximum likelihood estimate of the survival functions.

The following SAS® code will be utilized:

```
proc lifetest data=dd outsurv=dd1 method=KM alpha=0.05 alphaqt=0.05 conftype=linear
plots=(survival(strata=individual));
                    time <survival time> * <censoring indicator>;
                    by treatment;
run;
```

With the following indicators for the censoring of subjects:

- 1: If the subject had the event meeting the criteria for the specific event.
- 0: If the subject did not have the event meeting the criteria. Such a subject will be censored.

Subjects who do not have a dose interruption and/or dose reduction in Linezolid due to an AE will be censored at the time of treatment discontinuation or treatment completion.

21.1. DERIVATIONS

Based on the aforementioned, the following variables, as presented in the planned output shells will be derived:

- Duration of study drug administration/interruption/missed dose:
  - Duration of study drug administration/interruption/missed dose (days) = (End date of applicable event – start date of applicable event) + 1.
- Total duration on treatment (months):
  - Total duration on treatment= (Total duration of all individual study drug administrations – total duration of all missed doses)/(365.25/12).
- Reduction in Linezolid:
  - Records where the total daily dose decreases. An example would be 600 mg BID to 600 mg QD or 600 BID to 300 BID.
- Maximum duration of interruption (days):
  - For subjects who had more than one interruption due to an AE, only the dose interruption with the longest duration will be considered.
- Total duration of interruption (days):
  - For subjects with more than one dose interruption due to an AE, the duration of all dose interruptions due to an AE will be summed.
- Premature study drug administration discontinuation:
  - The B-L-Pa combination will include subjects who prematurely discontinue study drug as recorded on the Subject Disposition eCRF and the reason for premature discontinuation is adverse event or death. For Linezolid this is where the main reason of the last action taken per subject for Linezolid on the Study Drug Dosing eCRF is indicated as adverse event.
- Time (days) to first dose interruption and/or dose reduction of Linezolid due to an AE:
  - Time (days) = (Date of interruption or reduction – date of first study drug administration) + 1.

- Planned cumulative dose (mg) for Linezolid:
  - Planned cumulative dose (mg) = (Planned Dose x total number of days on Linezolid) where planned Dose = 1200 mg total daily dose in accordance with the study protocol.
- Actual cumulative dose (mg) for Linezolid:
  - Actual cumulative dose (mg) $=\sum_{x=1}^{n} \text{Actual Total Daily Dose} \times \text{total number of days on specific dose}$ where n is equal to the number of times a subject is on different doses (for example 600 BID versus 600 QD) and x is the specific dose. If a subject remains on one total daily dose (for example 1200 mg) for the entire treatment period, then n will be equal to 1.
- Relative dose (%) for Linezolid:
  - Relative Dose (%) = (Actual cumulative dose/planned cumulative dose) x 100.

22. EFFICACY ANALYSIS (ONLY APPLICABLE TO THE FIRST IA)

The efficacy analysis of the primary and key secondary efficacy endpoints will be performed using the ITT analysis population (first IA analysis).

22.1. PRIMARY EFFICACY

22.1.1. DERIVATIONS

At each visit two culture results will be available, a spot sample and an early morning sample. The derived culture result per sample will be derived as indicated in the table below. Subjects who are not able to produce sputum will be regarded as negative for that applicable sample.

Table 3: Derived Culture Result per Sample Algorithm

MGIT	ZNS	Blood Agar	Speciation*	Derived Culture Result per Sample	TTP Valid (Y/N)
Positive	Positive	Negative	Not done	Positive	Y
Positive	Positive	Negative	Positive	Positive	Y
Positive	Positive	Negative	Negative	Contaminated	N

MGIT	ZNS	Blood Agar	Speciation*	Derived Culture Result per Sample	TTP Valid (Y/N)
Positive	Positive	Positive or Other	Not done	Contaminated	N
Positive	Positive	Positive or Other	Positive	Contaminated	N
Positive	Positive	Positive or Other	Negative	Contaminated	N
Positive	Negative	Positive or Other	Not done	Contaminated	N
Positive	Negative	Negative	Not done	Contaminated	N
Negative	Not done	Not done	Not done	Negative	N
Negative	Positive	Negative	Positive	Negative	N

MGIT: Mycobacterial growth indicator tube. TTP: Time to sputum culture positivity. ZNS: Ziehl-Neelsen smear.
*Only applicable for results at Baseline and from Week 16 onwards.

A final single result per visit will be derived as indicated in the table below. For all analysis purposes (except for stable culture negative conversion), the derived result at Baseline will be considered positive for all subjects, regardless of the actual culture result derived per sample.

Table 4: Derived Result per Visit Algorithm

Spot Sample Derived Culture Result	Early Morning Derived Culture Result	Final Derived Result per Visit
Positive	Missing/Negative/Contaminated	Positive
Missing/Negative/Contaminated	Positive	Positive
Negative	Missing/Contaminated	Negative
Missing/Contaminated	Negative	Negative
Contaminated	Missing/Contaminated	Contaminated
Missing/Contaminated	Contaminated	Contaminated

Figure 2: Algorithm for Interpretation of Positive MGIT Results

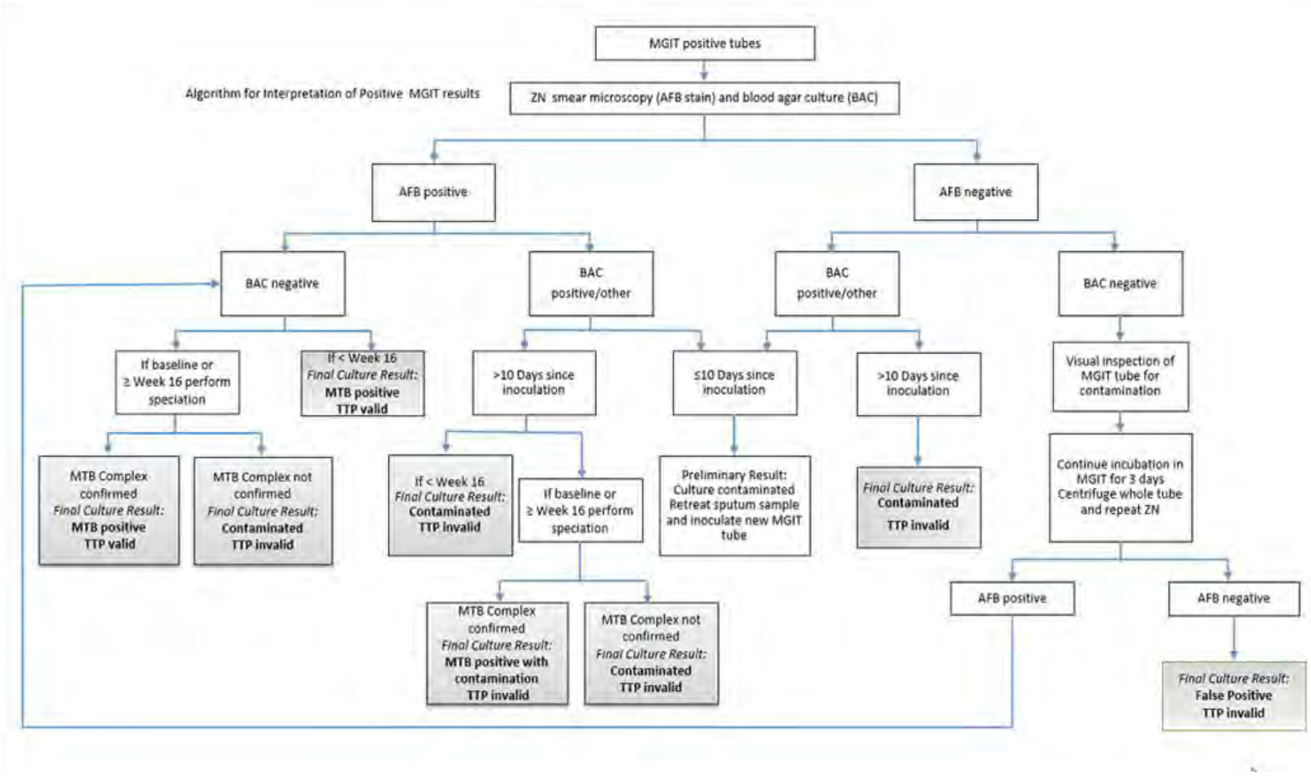

AFB: ZNS result. BAC: Blood agar result.

For the derived clinical failure status, medications recorded on the Concomitant Medications eCRF where the indication is recorded as XDR or MDR will be considered to check if a subject received TB treatment after the first study drug administration. If the medication starts after the first study drug administration but before the End of Treatment, this will be considered as a medication used due to treatment failure. If the medication starts after the End of Treatment this will be considered a medication used due to relapse. In addition, TB-related death will be identified by means of the Cause of death’s relationship to subject’s TB question on the Subject Disposition eCRF.

For clinical failure (Investigator), the bacteriology, clinical and radiological deterioration as recorded on the Investigator Assessment eCRF will be considered for a failure. For a success, the TB treatment success result will be considered.

22.1.2. DEFINITIONS

The following definitions are applicable:

- Bacteriological failure is defined as failing to attain confirmed culture negative status by the End of Treatment.
- Relapse (reinfection and reoccurrence) is defined as failing to maintain derived culture negative status after the End of Treatment. Therefore, this will be all subjects who achieve stable culture negative conversion at the End of Treatment and who have two consecutive culture positive (based on derived result per visit) samples.
- Reinfection is defined as failing to main derived culture negative status after the End of Treatment due to reinfection with a genetically distinguishable Mycobacterium tuberculosis strain. This will be relapse with a genetically distinguishable Mycobacterium tuberculosis strain (if the result from 'Are the two strains indistinguishable?' is 'No') as recorded on the Mycobacteriology Laboratory: Mycobacteriology Characterization eCRF.
- Reoccurrence is defined as failing to maintain derived culture negative status after the End of Treatment with a genetically distinguishable identical Mycobacterium tuberculosis strain. This will be relapse with a genetically identical Mycobacterium tuberculosis strain (if the result from 'Are the two strains indistinguishable?' is 'Yes') as recorded on the Mycobacteriology Laboratory: Mycobacteriology Characterization eCRF.
- Clinical failure (derived) is defined as change from the protocol-specified TB treatment due to treatment failure, retreatment for TB during follow-up period or TB-related death.
- Clinical failure (Investigator) as recorded on the Investigator Assessment eCRF.
- An unfavorable outcome is defined as subjects with bacteriological failure and/or relapse and/or derived clinical failure.
- Favorable outcome is defined as subjects who are not regarded as unfavorable.
- Stable culture negative conversion: At least 2 consecutive culture negative (based on derived result per visit) samples at least 7 days apart.

22.1.3. PRIMARY EFFICACY ENDPOINT

The primary efficacy endpoint if the study is:

- Proportion of treatment failure (unfavorable outcome), defined as bacteriologic failure or relapse or clinical failure (derived) through follow-up until 6 months after the End of Treatment.

They key secondary endpoints of this study are:

- Time to sputum culture conversion to negative status through the treatment period.
- Proportion of subjects with sputum culture conversion to negative status at 4, 6, 8, 12, 16 and 26 or 39 weeks.

22.1.4. ANALYSIS METHODS

A 95% CI based on the Clopper-Pearson 95% CI for a single binomial proportion will be calculated for the:

- Proportion of subjects with:
  - o An unfavorable outcome as defined above. Percentage (%) of subjects relative to the total number of subjects in the relevant analysis population.

The following SAS® code will be utilized:

```
proc freq data=dd ;
        tables variable / binomial (exact) alpha = .05;
        by treatment;
run;
```

Given that the ITT analysis population is defined as all subjects assigned to treatment, subjects with no evidence of study drug administration or insufficient data to determine unfavorable/favorable outcome will be regarded as favorable.

The following key secondary endpoint will be analyzed in a similar manner as described above:

- Time to sputum culture conversion to negative status through the treatment period.

- Proportion of subjects with sputum culture conversion to negative status at 4, 6, 8, 12, 16 and 26 or 39 weeks.

A 95% CI based on the Clopper-Pearson 95% for a single binomial proportion will be calculated for the:

- Subjects with a stable culture negative conversion at each post-baseline visit.
- Proportion of subjects with:
    - Favorable outcome. Percentage (%) of subjects relative to the total number of subjects in the relevant analysis population.
    - Bacteriological failure. Percentage (%) of subjects relative to the total number of subjects in the relevant analysis population.
    - Relapse. Percentage (%) of subjects relative to the total number of subjects in the relevant analysis population with a derived culture negative status at the End of Treatment.
    - Relapse due to a reinfection. Percentage (%) of subjects relative to the total number of subjects in the relevant analysis population with a derived culture negative status at the End of Treatment.
    - Relapse due to reoccurrence. Percentage (%) of subjects relative to the total number of subjects in the relevant analysis population with a derived culture negative status at the End of Treatment.
    - Clinical failure treatment failure. Percentage (%) of subjects relative to the total number of subjects in the relevant analysis population.
    - Clinical failure during following-up period. Percentage (%) of subjects relative to the total number of subjects in the relevant analysis population who completed treatment and entered the follow-up period.
    - Clinical failure (Investigator's assessment) at Month 6. Percentage (%) of subjects relative to the total number of subjects in the relevant analysis population who completed treatment and entered the follow-up period.
    - Clinical failure (Investigator's assessment) at Month 24. Percentage (%) of subjects relative to the total number of subjects in the relevant analysis population who completed treatment and entered the follow-up period.

In addition, Kaplan-Meier analysis of the median time (days) to event will be performed (refer to 21.1: Derivations for relevant information regarding the Kaplan-Meier analysis) for the following:

- Time to first stable culture negative conversion (days) = (Date of first stable culture negative conversion – date of first study drug administration) + 1. The first of the two negative cultures without an intervening positive culture will be considered. Subjects who do not have a stable culture negative conversion will be censored at the time of treatment discontinuation or treatment completion. Only subjects with a positive culture status at Baseline will be considered.
- Time to relapse/reinfection/reoccurrence (days) = (Date of relapse/reinfection/reoccurrence – date of last study drug administration) + 1. Subjects who do not have a relapse/reinfection/reoccurrence will be censored at the time of premature study discontinuation or study completion.

22.2. Other Secondary Efficacy

22.2.1. Other Secondary Efficacy Endpoints and Definitions

The other secondary efficacy endpoints of this study are:

- Change from Baseline in TB symptoms (refer to Section 22.2.2: Tuberculosis Symptoms).
- Change from Baseline in Patient Reported Health Status (refer to Section 22.2.3: Subject Reported Healthy Status: EQ-5D-5L).
- Change from Baseline in weight (kg) (refer to Section 22.2.4: Weight).

The analysis will be performed using the Safety analysis population defined as all subjects who received at least one administration of study drug.

22.2.2. Tuberculosis Symptoms

Tuberculosis symptom data as recorded on the Tuberculosis Symptom Profile eCRF will be presented.

With reference to Section 6 General Considerations and Section 7 Statistical Considerations of this SAP, the following data will be summarized:

- TB symptoms:
    - Feeling feverish.
    - Feeling chills.
    - Excessive sweating.
    - Shortness of breath.
    - Chest pain.
    - Feeling unwell.
    - Tiredness/weakness.
    - Cough.
    - Coughing up mucus.
    - Coughing up blood.
- And the result of each TB symptom at each scheduled visit:
    - None.
    - Mild.
    - Moderate.
    - Severe.
- A shift table for change from Baseline at the End of Treatment/End of Study (when applicable) will be presented. Percentage (%) of subjects in each category will be calculated relative to the total number of subjects in the relevant analysis population, with assessments available at Baseline and End of Treatment/End of Study (when applicable) visit.
- The results for each TB symptom at each visit will also be presented in a stacked bar graph displaying the number of subjects with each result. All mild, moderate or severe results will be displayed in a by-subject data listing.

22.2.3. SUBJECT REPORTED HEALTH STATUS: EQ-5D-5L

Subject reported health status: European quality of life – 5 dimensions (EQ-5D-5L) data as recorded on the EQ-5D-5L eCRF will be presented.

With reference to Section 6 General Considerations and Section 7 Statistical Considerations of this SAP, the following data will be summarized:

- Subjects reported health status: EQ-5D-5L qualitative items and applicable results:
    - Mobility.
    - Self-care.
    - Usual activities.
    - Pain/discomfort.
    - Anxiety/depression.
- And the result of each item at each scheduled visit:
    - None.
    - Slight.
    - Moderate.
    - Severe.
    - Extreme/unable.
- Subject reported health status: EQ-5D-5DL quantitative item:
    - Visual analog scale (VAS) Score. A score of 100 means the best health one can imagine and 0 means the worst health one can imagine. Please see below:

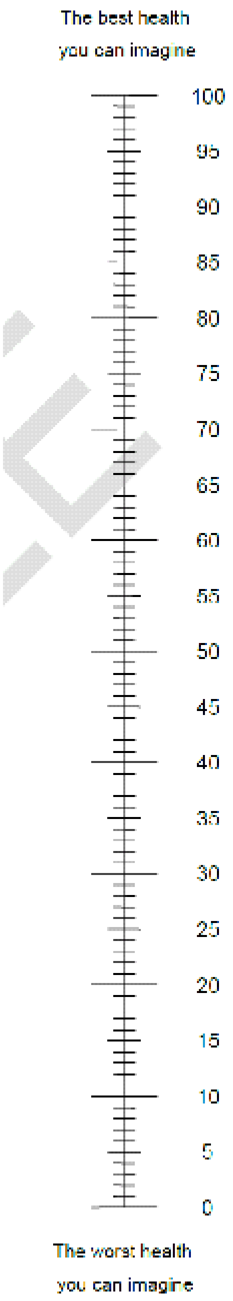

- Observed result at each visit.

  - Change from Baseline and Baseline value at each post-baseline visit and the End of Treatment/End of Study (when applicable). Change from Baseline will only be derived if both the Baseline and the relevant post-baseline/End of Treatment/End of Study (when applicable) assessment are available.
- A shift table for change from Baseline at the End of Treatment/End of Study (when applicable) will be presented. Percentage (%) of subjects in each category will be calculated relative to the total number of subjects in the relevant analysis population, with assessments available at Baseline and End of Treatment/End of Study (when applicable) visit.
- The result for each item at each visit will also be presented in a stacked bar graph displaying the number of subjects with each result.
- A scatterplot for VAS score by visit will be provided.

22.2.4. WEIGHT

Weight (kg) as recorded on the Vital Signs eCRF will be presented.

With reference to Section 6 General Considerations and Section 7 Statistical Considerations of this SAP, the following data will be summarized:

- Observed result at each visit.
- Change from Baseline and Baseline value at each post-baseline visit and the End of Treatment/End of Study (when applicable). Change from Baseline will only be derived if both the Baseline and the relevant post-baseline/End of Treatment/End of Study (when applicable) assessment are available.
- Percentage (%) change from Baseline at each visit. This is only applicable to the figure presentation of mean (+/-) SD at each relevant post-baseline visit.

All assessments (scheduled or unscheduled) will be presented in the by-subject data listings.

23. SAFETY OUTCOMES

The Safety analysis population will be used as primary analysis population for all safety analyses.

23.1. ADVERSE EVENTS

Adverse events (AEs) will be presented as recorded on the Adverse Events eCRF.

Adverse events will be categorized as follows:

- Prior AEs: Defined as AEs which started prior to the first study drug administration.
- TEAE: Defined as AEs which started or worsened on or after the first study drug administration up to and including 14 days after the last study drug administration.
- Post-treatment AE: Defined as AEs which started or worsened after 14 days after the last study drug administration.

Adverse events (AEs) will be coded using Medical Dictionary for Regulatory Activities (MedDRA) Version 19.0.

With reference to Section 6 General Considerations and Section 7 Statistical Considerations of this SAP, the incidence of TEAEs will be presented by SOC and PT or SOC, high level group term (HLGT) and PT (AEs of special interest only). Uncoded AEs (i.e., AEs with no dictionary coding information available) are only to be presented if applicable using the Investigator's Verbatim Term (eCRF) as PT within the text 'UNCODED' as SOC (only applicable for IA). System Organ Classes (SOCs) will be sorted by total decreasing frequency. High level group terms (HLGT) (if applicable) will be sorted by total decreasing frequency with each SOC. Preferred Terms (PTs) will be sorted by total decreasing frequency within each SOC or HLGT (if applicable). If SOCs, HLGTs (if applicable) or PTs have the same total frequency will be sorted alphabetically.

For presentation and derivation purposes, the following statistics are defined:

- n: Defined as the number of subjects with at least one TEAE in each category. Subjects with multiple TEAEs in each category are counted only once in each category.
- E: Defined as the number of mentions (events) in each category, i.e., the actual unique number of events.

- N: Defined as the total number of subjects in the relevant analysis population.
- %: Defined as the percentage of subjects with at least one TEAE in each category calculated relative to the total number of subjects in the relevant analysis population.

23.1.1. Severity

The severity (Division of Microbiology and Infectious Disease [DMID] grading) of each AE, as indicated by the Investigator, is classified as:

- Grade 1 (mild).
- Grade 2 (moderate).
- Grade 3 (severe).
- Grade 4 (potentially life threatening).

23.1.2. Relationship

The relationship to the study drug regimen, as indicated by the Investigator, is classified as:

- Not related.
- Unlikely.
- Possible.
- Probable.
- Certain.

If the relationship to the study drug regimen is indicated as 'Possible', 'Probable', 'Certain' by the Investigator, the relationship to Investigational medicinal product (MP) will be assessed for:

- Bedaquiline:
    - Not related.
    - Unlikely.
    - Possible.
    - Probable.
    - Certainly.

- Pretomanid (same categories as for Bedaquiline).
- Linezolid (same categories as for Bedaquiline).

The relationship to each IMP was only added for data entered from 01 January 2017 onwards and will therefore only be presented in by-subject data listings.

23.1.3. ACTION TAKEN WITH STUDY DRUG

The action taken with study drug (overall), as indicated by the Investigator, is classified as:

- Investigational medicinal product (IMP) unchanged.
- Investigational medicinal product (IMP) interrupted.
- Investigational medicinal product (IMP) stopped.
- Not applicable.
- Investigational medicinal product (IMP) dose reduced.

The action taken with Linezolid IMP, as indicated by the Investigator, is classified as:

- Investigational medicinal product (IMP) unchanged.
- Investigational medicinal product (IMP) interrupted.
- Investigational medicinal product (IMP) stopped.
- Not applicable.
- Investigational medicinal product (IMP) dose reduced.

The action taken with Bedaquiline/Pretomanid IMP, as indicated by the Investigator, is classified as:

- Investigational medicinal product (IMP) unchanged.
- Investigational medicinal product (IMP) interrupted.
- Investigational medicinal product (IMP) stopped.
- Not applicable.
- Investigational medicinal product (IMP) dose reduced.

The action taken with each IMP was only added for data entered from 01 January 2017 onwards and will therefore only be presented in by-subject data listings.

Action taken (overall) classified as IMP stopped will be used to identify and select all AEs leading to discontinuation of study drug. Action taken classified as IMP interrupted will be used to identify and select all AEs leading to interruption of study drug.

23.1.4. ADVERSE EVENTS (AES) LEADING TO DEATH

Adverse events (AEs) leading to death are selected as all AEs with an outcome recorded as 'Fatal'.

23.1.5. SERIOUS ADVERSE EVENTS (AES)

Serious AEs are events judged as 'Serious' by the Investigator. Serious criteria include the following events:

- Death.
- Life-threatening.
- Initial or prolonged hospitalization.
- Persistent or significant disability/incapacity.
- Congenital anomaly or birth defect.
- Other medically important event (not covered by other 'serious' criteria).

23.1.6. ADVERSE EVENTS (AES) LEADING TO DISCONTINUATION OF STUDY DRUG

Adverse events (AEs) leading to permanent discontinuation of study drug will be identified and selected based on response of 'Yes' to the question 'Did the adverse event cause the subject to be discontinued from the study?

23.1.7. ALL TREATMENT-EMERGENT AEs (TEAEs)

Based on the aforementioned definition, an overview summary will be provided as follows:

Variable
SUBJECTS WITH AT LEAST ONE
TEAE
TEAES LEADING TO DEATH
SERIOUS TEAE (INCLUDING DEATH)
TEAE LEADING TO EARLY WITHDRAWAL
TEAE LEADING TO DISCONTINUATION OF STUDY DRUG
TEAE LEADING TO INTERRUPTION OF STUDY DRUG
GRADE III AND/OR IV TEAE
DRUG-RELATED TEAE
TEAE OF SPECIAL INTEREST

23.1.8. ADVERSE EVENTS (AEs) OF SPECIAL INTEREST

Adverse events (AEs) of special interest will be selected based on an identified list of PT, High Level Term (HLT) and High Level Group Terms (HLGT) and confirmed by TB Alliance.

The number (n) and percentage (%) of subjects with at least on TEAE of special interest will be presented as a histogram with HLGT terms on the x-axis and the number of subjects on the y-axis.

The summary table will be presented by SOC, HLGT and PT.

Preferred Term
ALANINE AMINOTRANSFERASE INCREASED
ASPARTATE AMINOTRANSFERASE INCREASED
BLOOD ALKALINE PHOSPHATASE INCREASED
AMYLASE INCREASED
LIPASE INCREASED
MYALGIA
MUSCLE NECROSIS
BLOOD CREATINE PHOSPHOKINASE MB INCREASED
ELECTROCARDIOGRAM QT PROLONGED
BUNDLE BRANCH BLOCK LEFT

Preferred Term
ATRIOVENTRICULAR BLOCK SECOND DEGREE
ATRIOVENTRICULAR BLOCK COMPLETE
HAEMOGLOBIN DECREASED
NEUROPATHY PERIPHERAL
VISUAL ACUITY REDUCED
CHROMATOPSIA
LACTICT ACIDOSIS
BLOOD BICARBONATE DECREASED
ANAEMIA
BURNING SENSATION
GENERALISED TONIC-CLONIC SEIZURE
HYPERAMYLASAEMIA
NEUTROPENIA
DISSEMINATED TUBERCULOSIS
PAPILLOEDEMA
EYE SWELLING
PARAESTHESIA
BONE MARROW FAILURE
LIPASE URINE INCREASED
**High Level Term**
NEUTROPENIAS
PERIPHERAL NEUROPATHIES NEC
THROMBOCYTOPENIAS
**High Level Group Term**
HEPATIC AND BILIARY NEOPLASMS BENIGN
HEPATIC AND HEPATOBILIARY DISORDERS
HEPATOBILIARY DISORDERS CONGENITAL
HEPATOBILIARY NEOPLASMS MALIGNANT AND UNSPECIFIED
HEPATOBILIARY INVESTIGATIONS
HEPATOBILIARY THERAPEUTIC PROCEDURES
CARDIAC ARRHYTHMIAS
NEUROPENIAS
THROMBOCYTOPENIAS

23.1.1. LIVER-RELATED AEs

Liver-related AEs will be selected based on an identified list of High Level Group Terms (HLGT) and confirmed by TB Alliance.

The summary table will be presented by SOC and PT.

High Level Group Term
HEPATIC AND BILIARY NEOPLASMS BENIGN
HEPATIC AND HEPATOBILIARY DISORDERS
HEPATOBILIARY DISORDERS CONGENITAL
HEPATOBILIARY NEOPLASMS MALIGNANT AND UNSPECIFIED
HEPATOBILIARY INVESTIGATIONS
HEPATOBILIARY THERAPEUTIC PROCEDURES

23.2. LABORATORY TESTS

Safety laboratory data as captured on the Safety Laboratory Sample Collection and Safety Laboratory Test Results eCRFs will be presented. All relevant laboratory category/variable and unit are presented in Laboratory Tests of this SAP.

With reference to Section 6 General Considerations and Section 7 Statistical Considerations of this SAP, the following data will be summarized:

- Observed result at each visit.
- Baseline and change from Baseline at each post-baseline visit and the End of Treatment. Change from Baseline will only be derived if both the Baseline and the relevant post-baseline/End of Treatment assessment are available.

23.2.1. DIVISION OF MICROBIOLOGY AND INFECTIOUS DISEASE (DMID) GRADING FOR LABORATORY DATA

The laboratory results will be graded programmatically using the DMID, Version November 2007. For details regarding each of the following levels, refer to Appendix 5: Division of Microbiology and Infectious Disease (DMID) Laboratory Tests Toxicity of this SAP:

- Grade 1.
- Grade 2.
- Grade 3.
- Grade 4.

Subjects with a result not complying with the DMID grades for a given laboratory assessment will be assigned a Grade 0 severity.

With reference to Section 6 General Considerations and Section 7 Statistical Considerations of this SAP, the following data will be summarized:

- Division of Microbiology and Infectious Disease (DMID) grade for each relevant laboratory test at each post-baseline visit.
  - o Percentage of subjects in each category relative to the number of subjects in the relevant population with data available for the applicable test at the applicable visit
- A change in each laboratory variable based on the shift from Baseline DMID grade to the End of Treatment.
  - o Percentage of subjects in each category relative to the number of subjects in the relevant population with data available for the applicable test at both Baseline and the End of Treatment visit.
- A figure of the mean +/- SD per visit will be presented for those laboratory categories and tests with at least one ≥ grade 3 values.
- Laboratory profile plots will be created per subject, for subjects with a ≥ grade 3 toxicity value for the following categories and tests:
  - o Hematology: Hemoglobin, platelets, neutrophils and/or white blood cells. All tests will be plotted on one figure, by visit (x-axis) and upper limit of normal (ULN) normalized value (y-axis).
  - o Chemistry: Amylase and lipase in a similar format as described for the hemoglobin, platelets, neutrophils and/or white blood cells figure.

23.2.2. LIVER-RELATED ABNORMALITIES AND EVALUATION IF DRUG-INDUCED SERIOUS HEPATOTOXICITY

With reference to Section 6 General Considerations and Section 7 Statistical Considerations of this SAP, the following data will be summarized:

- The classification of liver-related abnormalities: Alanine aminotransferase (ALT), aspartate aminotransferase (AST), alkaline phosphatase (ALP) and total bilirubin at each visit, End of Treatment and treatment-emergent incidence as follows:
    - > 3 x upper limit of normal (ULN).
    - > 3 x ULN.
    - > 8 x ULN.
    - Classification categories are not mutually exclusive.
    - Treatment-emergent incidence includes all liver-related abnormalities (per classification) after the study drug administration up to and including 14 days after the last study drug administration.
    - Percentage of subjects in each category relative to the total number of subjects in the relevant analysis population with data available for each specific test at each applicable visit.
- The evaluation of drug-induced serious hepatotoxicity based on the following classifications at each visit:
    - Total bilirubin ≥ 2 x ULN and ALT ≥ 3 x ULN (possible Hy's law).
    - Total bilirubin > 2 x ULN and ALT > 3 x ULN.
    - Total bilirubin > 2 x ULN and ALT > 5 x ULN.
    - Total bilirubin > 2 x ULN and ALT > 8 x ULN.
    - Classification categories are not mutually exclusive.
    - Treatment-emergent incidence includes all liver-related abnormalities (per classification) identified after the first study drug administration up to and including 14 days after the last study drug administration.
    - Percentage of subjects in each category relative to the number of subjects in the relevant population with data available for both total bilirubin and ALT.
    - Similar classifications for total bilirubin and AST will be performed.

All abnormal assessments (scheduled or unscheduled) will be presented in by-subject data listings.

The following figures will be presented:

- An eDISH plot of treatment-emergent incidence of total bilirubin versus ALT. A similar plot of total bilirubin versus AST will be produced.
- By-subject profile plots for subjects with treatment-emergent eDISH abnormalities including the following laboratory tests: ALT, AST, ALP and total bilirubin.

23.2.3. DERIVATIONS

Numerical laboratory assessments reported as '< X', i.e. below limit of quantification (BLQ), or '> X', i.e. above the upper limit of quantification (ULQ), will be converted to X for the purpose of numerical summaries, but will be presented as recorded, i.e. as '< X' or '> X' in the by-subject data listings.

Quantitative laboratory assessments will be categorized in accordance with the relevant laboratory reference ranges as follows:

- Low: Below the lower limit of the laboratory reference range.
- Normal: Within the laboratory reference range (upper and lower limit included).
- High: Above the upper limit of the laboratory reference range.

23.3. VITAL SIGNS

Vital signs data (BMI, systolic blood pressure, diastolic blood pressure, heart rate, axillary body temperature and respiratory rate) as captured on the Vital Signs eCRF will be presented.

With reference to Section 6 General Considerations and Section 7 Statistical Considerations of this SAP, the following data will be summarized:

- Observed result at each visit.
- Baseline and change from Baseline results at each post-baseline visit and the End of Treatment/End of Study (when applicable). Change from Baseline will only be derived if both the Baseline and the relevant post-baseline/End of Treatment/End of Study (when applicable) assessment is available.

23.3.1. NOTABLE ABNORMALITIES

The following vital signs assessments will be classified according to the criteria below, as specified in Appendix 3: Vital Signs of the study protocol:

- Diastolic blood pressure.
- Systolic blood pressure.

The categories are as follows:

- Abnormally low.
- Grade 1 or mild.
- Grade 2 or moderate.
- Grade 3 or severe.

Subjects with a result not complying with the above categories for a given vital signs assessment will be assigned Grade 0.

With reference to Section 6 General Considerations and Section 7 Statistical Considerations of this SAP, the following data will be summarized:

- Notable abnormality for each relevant vital signs test at each post-baseline visit.
    - o Percentage of subjects in each category relative to the number of subjects in the relevant population with data available for the applicable test at the applicable visit.
- A change in each vital signs test based on the shift from Baseline notable abnormality to the End of Treatment/End of Study (when applicable).
    - o Percentage of subjects in each category relative to the number of subjects in the relevant population with data available for the applicable test at both Baseline and the End of Treatment/End of Study (when applicable) visit.

All abnormal assessments (scheduled or unscheduled) will be presented in by-subject data listings.

23.4. Electrocardiogram Parameters

Electrocardiogram (ECG) data (heart rate, PR, time required for depolarization and repolarization of ventricles [QT], QT interval corrected using Bazett's method [QTcB], QT interval corrected using Fridericia's method [QTcF]) as captured on the 12-Lead ECG Results eCRF will be presented.

The overall interpretation as judged by the Investigator will be classified and displayed in by-subject data listings only as:

- Normal.
- Abnormal, not clinically significant.
- Abnormal, clinically significant.

With reference to Section 6 General Considerations and Section 7 Statistical Considerations of this SAP, the following data will be summarized:

- Observed result at each visit.
- Baseline and change from Baseline results at each post-baseline visit and at the End of Treatment visits. Change from Baseline will only be derived if both the Baseline and the relevant post-baseline/End of Treatment assessment is available.

23.4.1. Electrocardiogram Prolongation

QT, QTcB and QTcF will be classified as.

- > 450 msec.
- > 480 msec.
- ≥ 500 msec.

The aforementioned categories are not mutually exclusive and a result can therefore be included in one or more categories.

- At post-baseline visits and the End of Treatment visit, the change from Baseline will be categorized as:
    - > 30 msec increase from Baseline.

- > 60 msec increase from Baseline.
- A change will only be derived if both the Baseline and the relevant post-baseline or End of Treatment assessment is available. Percentages (%) will be based on the number of subjects with data available at both Baseline and the relevant post-baseline or End of Treatment assessments.

The following profile plot figures will be presented:

- Mean QT, QTcB, QTcF and heart rate intervals over time.
- Mean change from Baseline in QT, QTcB and QTcF intervals over time.

All abnormal assessments (scheduled or unscheduled) will be presented in by-subject data listings.

23.5. OPHTHALMOLOGICAL EXAMINATION

Visual acuity and color vision data as captured on the Ophthalmology Examination (Visual acuity color vision) eCRF will be presented.

- The Ophthalmologist/delegate will test distance, near and visual acuity for both the right and the left eye. The best corrected visual acuity will be measured. Metric designations will be used.
  - For the distance visual acuity the Snellen chart and notation will be used and the evaluation will be performed at 6 meters.
  - For the near visual acuity the Jaeger chart and notation will be used. The evaluation will be performed at 40 cm.
- For color vision, each eye should be tested individually (covering the opposite eye), using the 24 plate Ishihara series, held out at a distance of 75 cm at a right angle to the subject.
  - All 24 plates in the book should be presented to the subject, one at a time. A total score out of 21 for each eye will be recorded on the color vision assessment form.

With reference to Section 6 General Considerations and Section 7 Statistical Considerations of this SAP, data will be summarized for visual acuity (near), visual acuity (distance) and color vision:

- Change from Baseline at each post-baseline visit and the End of Treatment/End of Study (when applicable) per eye, based on the following categories:

- > 2 categories increase.
- 2 category increase.
- 1 category increase.
- No change.
- > 2 categories decease.
- 2 category decease,
- 1 category decrease.
- Percentage (%) subjects in each category relative to the total number of subjects in the relevant analysis populations with data available at Baseline and the relevant post-baseline/End of Treatment/End of Study (when applicable) visit.

A worsening will be regarded as:

- Near: When the numeric value next to 'J' increases by one or more.
- Distance: When the numeric value next to '6/' increases by one or more.
- Color vision: When the numeric value decreases by one or more.

A by-subject figure will be presented for subjects who experience any worsening from Baseline in any of the aforementioned.

All scheduled and unscheduled assessments will be presented in a by-subject data listing.

23.6. Slit Lamp Examination

Age-related eye disease study 2 (AREDS2) lens grading data as captured on the Slit Lamp Examination eCRF will be presented.

The slit lamp examination results will be documented using the AREDS2 clinical lens opacity classification and grading system. With this system, the lens will be evaluated and graded by comparison to a series of standard photographs for each possible type of lens opacity, the location and degree of lens opacity being determined. The possible type of lens opacity and its grading will be recorded. The possible types are nuclear (9 possible grades), cortical (9 possible grades) and posterior sub-capsular (PSC) (9 possible grades).

The lens will be examined and compared to the corresponding series of three standard photographs for each of the three types of lens opacity. The lens will be graded according to these

standard photographs for severity or extent of each type of lens opacity in the eye under consideration. The grades range from 0.0 (for clear), to 4.0 (for completely opacified), with the standards defining whole steps between these two extremes. The grading of 8.0 should be used if the lens cannot be evaluated.

Results according to the AREDS2 grading system:

- 0.0 = No lens opacity.
- > 0.0 to <= 4.0 = Lens opacity present.
- 8.0 = Cannot evaluate.

With reference to Section 6 General Considerations and Section 7 Statistical Considerations of this SAP, the following data will be summarized at each visit, and per eye:

- Proportion of subjects with opacity for the following categories:
  - Nuclear.
  - Cortical.
  - Posterior subcapsular.
  - Percentage (%) of subjects in each category relative to the total number of subjects in the relevant analysis population with data available for each eye and applicable visit.

All scheduled and unscheduled assessments will be presented in a by-subject data listing.

23.7. PERIPHERAL NEUROPATHY

Peripheral neuropathy data as captured on the Peripheral Neuropathy eCRF will be presented.

With reference to Section 6 General Considerations and Section 7 Statistical Considerations of this SAP, the following data will be summarized at each visit:

- Individual item and total neuropathy numeric score. Items include:
  - Rate of interference with walking or sleeping.
  - Subject elicited symptoms:
    - Pain, aching or burning in feet or legs.
    - ‘Pins and needles’ in feet and legs.

    - Numbness (lack of feeling) in feet or legs.
- A change in each item based on the shift from Baseline to the End of Treatment/End of Study (when applicable) based on the eCRF categories (response category) for each item. A change will only be derived if both the Baseline and the relevant End of Treatment/End of Study (when applicable) assessment is available. Percentages (%) will be based on the number of subjects with data available at both Baseline and the relevant End of Treatment/End of Study (when applicable) assessments.

23.7.1. DERIVATIONS

Total neuropathy symptom score is derived as the sum of the numeric scores of the following individual questions:

- Interference with walking or sleeping:
    - 01 (minimal).
    - 02 (minimal).
    - 03 (minimal).
    - 04 (modest).
    - 05 (modest).
    - 06 (modest).
    - 07 (modest).
    - 08 (severe).
    - 09 (severe).
    - 10 (severe).
- Pain, aching or burning in feet/legs.
    - 00- Very happy, no symptoms.
    - 02 – Just a little bit.
    - 04 – A little more.
    - 06 – Even more.
    - 08 – A whole lot.
    - 10 – Worst.
- Pins and needles in feet/legs (same categories as pain, aching or burning in feet/legs).

- Numbness (lack of feeling) in feet/legs (same categories as pain, aching or burning in feet/legs).

24. LIST OF REFERENCES

[1] NiX-TB Protocol Version 1 21APR2014.

[2] NiX-TB Protocol Version 2 final working protocol 18MAR2015.

[3] NiX-TB Protocol Version 3 final working protocol 22JAN2016.

[4[NiX-TB Protocol Version 4 final working protocol 24APR2017.

[4] NiX-TB Annotated CRF Version 8.0 dated 17February2017.

APPENDIX 1. PROGRAMMING CONVENTIONS FOR TABLES, BY-SUBJECT DATA LISTINGS AND FIGURES

Paper Size, Orientation and Margins

The margin, page size and line size specifications as stipulated below will be used for the presentation of all TLFs:

	Landscape	Portrait
Margins (Inches):		
Top	1.25	1
Bottom	1	1
Left	1	1.25
Right	1	1
Header (Inches)	0.5	0.5
Footer (Inches)	0.5	0.5
SAS® specifications:		
PAGESIZE	46	67
LINE SIZE	134	93

Fonts

The font type 'Courier New' should be used as default for tables and by-subject data listings, with a font size of 8. The font color should be black. No bolding, underlining and italics are permitted.

1

TB Alliance	B-L-Pa
NiX-TB-(B-L-Pa)	Page xx of xx

Table 14.1.4
Demographics
Safety Analysis Population

By-group<xxxx> 2

		B-L-Pa		
Variable 3	Statistic	XDR (N=15)	MDR (N=0)	Total (N=15)
AGE (YEARS) 4	n	15		15
	MEAN	33.1		33.1
	SD	10.87		10.87
	MINIMUM	20		20
	MEDIAN	31.0		31.0
	MAXIMUM	52		52
GENDER 5				
FEMALE	n (%)	5 (33.3) 6		5 (33.3)
CHILDBEARING POTENTIAL*				
YES	n (%)	5 (100.0)		5 (100.0)
NO	n (%)	0		0
NOT APPLICABLE	n (%)	0		0
MALE	n (%)	10 (66.6)		10 (66.6)

[illegible]atment group: B = Bedaquiline. L = Linezolid. Pa = Pretomanid. MDR: Multi drug-resistant. SD: Standard deviation. XDR: Extensively drug-resistant. n = Number of subjects in each category. 7 [illegible] Total number of subjects in the relevant analysis population. % = Percentage of subjects in each category relative to the total number of subjects in the relevant analysis population with data available. *: % for [illegible]dbearing potential = Percentage of subjects in each category relative to the total number of female subjects in the relevant analysis population with data available. Age (years): Calculated relative to Screening.

Program: x:\xxxxxx\xxxxxx\xxxxxx\xxxxxx.sas (ddmmmyyyy hh:mm) 8	File: xxxxxx.rtf

1. Header information. 2. By-group information. 3. Column heading information. 4. General information. 5. Denominator information. 6. Alignment information. 7. Footnote information. 8. Filename information.

Footnotes are to be ordered as follows:

- Non-standard abbreviations, separated by a full stop.
- n = Number of subjects… N = Total number of subjects… % = Percentage of subjects…
- Definitions.
- Footnotes pertaining to statistical methodology.
- If coding is presented, the version of the coding dictionary used.
- Study-specific footnotes to clarify data points within the specific presentation.

Figure Output Conventions

Figures should be provided in RTF files using the SAS® Output Delivery System (ODS).

Dates and Times

Depending on data available, dates and times will take the form ddMMMyyyy and hh:mm.

Spelling Format

The spelling format to be used is English US.

Presentation of Visits

For outputs, visits will be represented in the following order:

Visit Name
Screening
Baseline (derived)
Day 1
Week 1
Week 2
Week 3
Week 4
Week 5
Week 6
Week 7
Week 8
Week 9
Week 10
Week 11
Week 12
Week 13
Week 14
Week 15
Week 16
Week 20
Week 26
End of Treatment (derived)
Month 1
Month 2
Month 3
Month 6
Month 9
Month 12

Visit Name
Month 15
Month 18
Month 21
Month 25
End of Study (derived)
Unscheduled (chronologically)
Early Withdrawal

APPENDIX 2. PARTIAL ADVERSE EVENT DATE CONVENTIONS

Date imputations for partial AEs are only used for classification of TEAEs and partial dates will be displayed in the applicable by-subject data listings.

Algorithm for Treatment-emergent Adverse Events

Start Date	Action
Known	If AE start date < Reference start or AE start date > Reference end date + 14 days, then not treatment-emergent adverse event (TEAE).<br>If Reference start date ≤ AE start date ≤ Reference end date + 14 days, then TEAE.<br>If AE start date = Study drug start date and the variable '...prior to first dose of study medication' is equal to 'no', then TEAE.<br>If AE start date = Study drug start date and the variable '...prior to first dose of study medication' is equal to 'yes', then not TEAE.
Partial, but known components show that event cannot have started during treatment period up to and including 14 days after last study drug administration	Not TEAE according to definition.
Partial, but known components show that event could have started during treatment period up to and including 14 days after the last study drug administration (or 'Did the adverse event occur prior to first dose of study medication' is indicated as 'yes')	If AE start date < Reference start date or AE start date > Reference end date + 14 days, then not TEAE.<br>If Reference start date ≤ AE start date ≤ Reference end date + 14 days, then TEAE.<br>Else assumed TEAE.

APPENDIX 3. PARTIAL MEDICATIONS DATE CONVENTIONS

Date imputations for partial medications are only used for classification of concomitant medications and partial dates will be displayed in the applicable by-subject data listings.

Algorithm for Concomitant Medications (CM)

Start Date	Stop Date	Action
Known	Known or ongoing or partial	If CM start date < Reference start date and CM end date >= Reference start date or ongoing then CM.<br>If CM start date is >= Reference start date and CM start date <= Reference end date + 14 days then CM.<br>Else not CM.
Partial, but known components show that medication could have started during treatment period up to and including 14 days after the last study drug administration	Known, ongoing or partial	Assumed CM.
Partial, but known components show that medication could not have started during treatment period up to and including 14 days after the last study drug administration	Known or ongoing or partial	If CM start date < Reference start date and CM end date (or CM end date is partial and known components show that medicaiton could have ended during the treatment period up to and including 14 days ater the last study drug administration) >= Reference start date or ongoing then CM.<br>Else not CM.

APPENDIX 4. LABORATORY TESTS

Laboratory Category	Laboratory Test	Standard Unit
Chemistry	Alanine Aminotransferase	U/L
Chemistry	Albumin	g/L
Chemistry	Alkaline Phosphatase	IU/L
Chemistry	Aspartate Aminotransferase	U/L
Chemistry	Bicarbonate	mmol/L
Chemistry	Calcium Corrected for Albumin	mmol/L
Chemistry	Chloride	mmol/L
Chemistry	Creatinine	umol/L
Chemistry	Creatinine Kinase MB	ug/L
Chemistry	Creatinine Phosphikinase	IU/L
Chemistry	Direct Bilirubin	umol/L
Chemistry	Gamma Glutamyl Transferase	U/L
Chemistry	Glucose	mmol/L
Chemistry	Indirect Bilirubin	umol/L
Chemistry	Lactate Dehydrogenase	U/L
Chemistry	Lipase	U/L
Chemistry	Magnesium	mmol/L
Chemistry	Phosphate	mmol/L
Chemistry	Potassium	mmol/L
Chemistry	Serum Urea	mmol/L
Chemistry	Sodium	mmol/L
Chemistry	Total Amylase	U/L
Chemistry	Total Bilirubin	umol/L
Chemistry	Total Protein	g/L
Chemistry	Uric Acid	umol/L
Hematology	Basophils	%
Hematology	Basophils Absolute Count	10^9/L
Hematology	Eosinophils	%
Hematology	Eosinophils Absolute Count	10^9/L
Hematology	Hematocrit	%
Hematology	Hemoglobin	gm/dL
Hematology	Lymphocytes	%

Laboratory Category	Laboratory Test	Standard Unit
Hematology	Lymphocytes Absolute Count	10^9/L
Hematology	Monocytes	%
Hematology	Monocytes Abolsute Count	10^9/L
Hematology	Neutrophils	%
Hematology	Neutrophils Absolute Count	10^9/L
Hematology	Platelets	10^9L
Hematology	Red Blood Cells	10^9L
Hematology	White Blood Cell Count	10^9L
Urinalysis	Bilirubin	Not Applicable
Urinalysis	Creatinine	umol/L
Urinalysis	Glucose	Not Applicable
Urinalysis	Ketones	Not Applicable
Urinalysis	Leukocytes	/HPF
Urinalysis	Microalbumin	g/L
Urinalysis	Nitrite	Not Applicable
Urinalysis	pH	Not Applicable
Urinalysis	Protein	Not Applicable
Urinalysis	Sodium	mmol/L
Urinalysis	Specific Gravity	Not Applicable
Urinalysis	Urobilinogen	Not Applicable

APPENDIX 5: DIVISION OF MICROBIOLOGY AND INFECTIOUS DISEASE (DMID) LABORATORY TESTS TOXICITY

Chemistry				
	**Grade 1**	**Grade 2**	**Grade 3**	**Grade 4**
Hyponatremia (Sodium)<br>mEq/L<br>mmol/L	 130 – 135<br>130 – 135	 123 – 129<br>123 – 129	 116 - 122<br>116 – 122	 < 116<br>< 116<br>Or abnormal sodium with mental status changes or seizures
Hypernatremia (Sodium)<br>mEq/L<br>mmol/L	 146 – 150<br>146 – 150	 151 – 157<br>151 – 157	 158 – 165<br>158 – 165	 > 165<br>> 165<br>Or abnormal sodium with mental status changes or seizures
Hypokalemia (Potassium)<br>mEq/L<br>mmol/L	 3.0 – 3.4<br>3.0 – 3.4	 2.5 – 2.9<br>2.5 – 2.9	 2.0 – 2.4<br>2.0 – 2.4<br>Or intensive replacement therapy or hospitalization required	 < 2.0<br>< 2.0<br>Or abnormal potassium with paresis, ileus or life-threatening arrhythmia
Hyperkalemia (Potassium)<br>mEq/L<br>mmol/L	 5.6 – 6.0<br>5.6 – 6.0	 6.1 – 6.5<br>6.1 – 6.5	 6.6 – 7.0<br>6.6 – 7.0	 > 7.0<br>> 7.0<br>Or abnormal potassium with life-threatetning arrhythmia
Hypoglycemia (Glucose)<br>mg/dL<br>mmol/L	 55- 64<br>3.0525 – 3.552	 40 – 54<br>2.22 – 2.997	 30 -39<br>1.665 – 2.1645	 < 30<br>< 1.665<br>Or abnormal glucose with mental status changes or coma
Hyperglycemia (Glucose)<br>mg/dL<br>mmol/L	 116 – 160<br>6.438 – 8.88	 161 – 250<br>8.9355 – 13.875	 251 – 500<br>13.9305 – 27.75	 > 500<br>> 27.75

Chemistry				
	Grade 1	Grade 2	Grade 3	Grade 4
				Or abnormal glucose with ketoacidosis or seizures
Hypocalcemia (Calcium corrected for Albumin)<br>mg/dL<br>mmol/L	7.8– 8.4<br>1.95 – 2.1	7.0 – 7.7<br>1.75 – 1.925	6.1 – 6.9<br>1.525 – 1.725	< 6.1<br>< 1.525<br>Or abnormal calcium with life threatetning arrythmia or tetany
Hypercalcemia (Calcium corrected for Albumin)<br>mg/dL<br>mmol/L	10.6 – 11.5<br>2.65 – 2.875	11.6 – 12.5<br>2.9 – 3.125	12.6 – 13.5<br>3.15 – 3.375	> 13.5<br>> 3.375<br>Or abnormal calcium with life threatening arrythmia
Hypomagnesemia (Magnesium)<br>mEq/L<br>mmol/L	1.2 – 1.4<br>0.6 – 0.7	0.9 – 1.1<br>0.45 – 0.55	0.6 – 0.8<br>0.3 – 0.4	< 0.6<br>< 0.3<br>Or abnormal magnesium with life-threatening arrythmia
Hypophosphatemia (Phosphate)<br>mg/dL<br>mmol/L	2.0 – 2.4<br>0.646 – 0.7752	1.5 – 1.9<br>0.4845 – 0.6137<br>Or replacement Rx required	1.0 – 1.4<br>0.323 - 0.4522<br>Intensive therapy or hospitalization required	< 1.0<br>< 0.323<br>Or abnormal phsophate with life-threatening arrythmia
Hyperbilirubinemia (when accompanied by any increase in other liver function test)	1.1 - < 1.25 x ULN	1.25 - <1.5 x ULN	1.5 – 1.75 x ULN	> 1.75 x ULN
Hyperbilirubinemia (when other liver function are in the normal range)	1.1 - < 1.5 x ULN	1.5 - <2.0 x ULN	2.0 – 3.0 x ULN	> 3.0 x ULN

Chemistry				
	**Grade 1**	**Grade 2**	**Grade 3**	**Grade 4**
BUN	1.25 - 2.5 x ULN	2.6 - 5 x ULN	5.1 - 10 x ULN	> 10 x ULN
Hyperuricemia (Uric Acid)<br>mg/dL<br>umol/L	 7.5 – 10.0<br>446.1 – 594.8	 10.1 – 12.0<br>600.75 – 713.76	 12.1 – 15.0<br>719.71 – 892.2	 > 15.0<br>> 892.2
Creatinine	1.1 - 1.5 x ULN	1.6 - 3.0 x ULN	3.1 - 6 x ULN	> 6 x ULN or dialysis required
AST (SGOT)	1.1 - < 2.0 x ULN	2.0 - <3.0 x ULN	3.0 – 8.0 x ULN	> 8.0 x ULN
ALT (SGPT)	1.1 - < 2.0 x ULN	2.0 - <3.0 x ULN	3.0 – 8.0 x ULN	> 8.0 x ULN
GGT	1.1 - < 2.0 x ULN	2.0 - <3.0 x ULN	3.0 – 8.0 x ULN	> 8.0 x ULN
Alkaline Phosphatase	1.1 - < 2.0 x ULN	2.0 - <3.0 x ULN	3.0 – 8.0 x ULN	> 8.0 x ULN
Amylase	1.1 - 1.5 x ULN	1.6 - 2.0 x ULN	2.1 – 5.0 x ULN	> 5.1 x ULN
Lipase	1.1 - 1.5 x ULN	1.6 - 2.0 x ULN	2.1 – 5.0 x ULN	> 5.1 x ULN

Hematology				
	**Grade 1**	**Grade 2**	**Grade 3**	**Grade 4**
Hemoglobin (gm/dL)	9.5 – 10.5	8.0 – 9.4	6.5 – 7.9	< 6.5
Neutrophils Absolute Count<br>/mm^3<br>10^9/L	 1000 – 1500<br>1.0 – 1.5	 750 – 999<br>0.75 – 0.99	 500 – 749<br>0.5 – 0.749	 < 500<br>< 0.5
Platelets<br>/mm^3<br>10^9/L	 75000 – 99999<br>75 – 99.99	 50000 – 74999<br>50 – 74.99	 20000 – 49999<br>20 – 49.99	 < 20000<br>< 20
White Blood Cell Count<br>/mm^3<br>10^9/L	 11000 – 13000<br>11.0 – 13.0	 13000 – 15000<br>13.0 – 15.0	 15000 – 30000<br>15.0 – 30.0	 > 30000 or < 1000<br>> 30 or < 10
% Polymorphonuclear Leucocytes + Band Cells	> 80%	90 – 95%	> 95%	Not Applicable
Abnormal Fibrinogen	Low:<br>100-200 mg/dL<br>High:<br>400-600 mg/dL	Low:<br><100 mg/dL<br>High:<br>>600 mg/dL	Low:<br>< 50 mg/dL	Fibrinogen associated with gross bleeding or with disseminated

Hematology				
	Grade 1	Grade 2	Grade 3	Grade 4
				coagulation
Fibrin Split Product	20-40 mcg/ml	41-50 mcg/ml	51-60 mcg/ml	> 60 mcg/ml
Prothrombin Time (PT)	1.01 - 1.25 x ULN	1.26-1.5 x ULN	1.51 -3.0 x ULN	>3 x ULN
Activated Partial Thromboplastin (APPT)	1.01 -1.66 x ULN	1.67 - 2.33 x ULN	2.34 - 3 x ULN	> 3 x ULN
Methemoglobin	5.0 - 9.9 %	10.0 - 14.9 %	15.0 - 19.9%	> 20.0 %

Urinalysis				
	Grade 1	Grade 2	Grade 3	Grade 4
Proteinuria (Protein)	1+ or 200 mg - 1 mg loss/day	2 - 3+ or 1 - 2 mg loss/day	4+ or 2 - 3.5 mg loss/day	Nephroticsyndrome or > 3.5 mg loss/day
Hematuria	microscopic only <10 rbc/hpf	gross, no clots >10 rbc/hpf	gross, with or without clots, OR red blood cell casts	obstructive or required transfusion

APPENDIX 6: VITAL SIGNS NOTABLE ABNORMALITIES

Vital Signs					
	Abnormally Low	Grade 1/ Mild	Grade 2/ Moderate	Grade 3/ Severe	Grade 4/ Abnormally High
Diastolic Blood Pressure	≤ 50 mmHg	> 90 mmHg- <100 mmHg	≥ 100 mmHg- <110 mmHg	≥ 110 mmHg	Not Applicable
Systolic Blood Pressure	≤ 90 mm Hg	> 140 mmHg- <160 mmHg	≥ 160 mmHg- <180 mmHg	≥ 180 mmHg	Not Applicable

Certificate Of Completion

Envelope Id: 40005813F2144667AAD50CC640116398		Status: Completed
Subject: Please DocuSign: 20171114 NiX-TB-(B-L-Pa) Final and Interim Safety Analyses SAP V2.0.docx		
Source Envelope:		
Document Pages: 79	Signatures: 2	Envelope Originator:
Certificate Pages: 2	Initials: 0	Joanna Moreira
AutoNav: Enabled		40 Wall St FL 24
EnvelopeId Stamping: Disabled		New York, NY 10005
Time Zone: (UTC-05:00) Eastern Time (US & Canada)		Joanna.Moreira@tballiance.org
		IP Address: 38.105.215.233

Record Tracking

Status: Original<br>11/15/2017 12:53:09 PM	Holder: Joanna Moreira<br>Joanna.Moreira@tballiance.org	Location: DocuSign

Signer Events	Signature	Timestamp
Dan Everitt<br>dan.everitt@tballiance.org<br>VP & Senior Medical Officer<br>Global Alliance for TB Drug Development (Part 11 Compliant)<br>Security Level: Email, Account Authentication (Required)	Signature ID:<br>32534894-D929-4A59-B14B-10FC37E90452<br>Using IP Address: 38.105.215.226<br><br>With Signing Authentication via DocuSign password<br>With Signing Reasons (on each tab):<br>I approve this document	Sent: 11/15/2017 12:53:22 PM<br>Viewed: 11/15/2017 12:58:41 PM<br>Signed: 11/15/2017 12:59:05 PM
**Electronic Record and Signature Disclosure:**<br>Not Offered via DocuSign		
Joanna Moreira<br>joanna.moreira@tballiance.org<br>Clinical Project Manager<br>Global Alliance for TB Drug Development (Part 11 Compliant)<br>Security Level: Email, Account Authentication (Required)	Signature ID:<br>D7909ECE-E1CF-40F6-96C1-02E04042B149<br>Using IP Address: 38.105.215.226<br><br>With Signing Authentication via DocuSign password<br>With Signing Reasons (on each tab):<br>I have reviewed this document	Sent: 11/15/2017 12:59:06 PM<br>Viewed: 11/15/2017 2:26:21 PM<br>Signed: 11/15/2017 2:26:59 PM
**Electronic Record and Signature Disclosure:**<br>Not Offered via DocuSign		

In Person Signer Events	Signature	Timestamp

Editor Delivery Events	Status	Timestamp

Agent Delivery Events	Status	Timestamp

Intermediary Delivery Events	Status	Timestamp

Certified Delivery Events	Status	Timestamp

Carbon Copy Events	Status	Timestamp

Notary Events	Signature	Timestamp

Envelope Summary Events	Status	Timestamps
Envelope Sent	Hashed/Encrypted	11/15/2017 12:59:06 PM
Certified Delivered	Security Checked	11/15/2017 2:26:21 PM
Signing Complete	Security Checked	11/15/2017 2:26:59 PM
Completed	Security Checked	11/15/2017 2:26:59 PM

Payment Events	Status	Timestamps

Changes to the Nix-TB Safety Statistical Analysis Plan

From: Safety Statistical Analysis Plan, Final and Interim Analyses, V1, 06Jul2017

To: Safety Statistical Analysis Plan, Final and Interim Analyses, V2, 14Nov2017

1) The following text in section 23.1.3 (Action taken with study drug) was removed: "Drug associated with the action taken with study drug will be obtained from the Study Drug Dosing eCRF by means of the action taken with Linezolid IMP and/or action taken with Bedaquiline/Pretomanid IMP".
2) The derivation of AE duration in days was deleted.
3) In section 23.2.2 (Liver-related abnormalities and evaluation of drug-induced serious hepatotoxicity) it was indicated that all abnormal assessments will be presented (as opposed to all assessment – normal or abnormal).
4) A reference to protocol version 4 was added.
5) The date of the latest CRF version was added.

LABORATORY MANUAL

MYCOBACTERIOLOGY LABORATORIES

Protocol Title:
A Phase 3 open-label trial assessing the safety and efficacy of bedaquiline plus pretomanid plus linezolid in Subjects with pulmonary infection of either extensively drug-resistant tuberculosis (XDR-TB) or treatment intolerant / non-responsive multi-drug resistant tuberculosis (MDR-TB)

Protocol Number: NiX-TB (B-L-Pa)
Version: 5.0; 15 MAY 2018

Supersedes: Version 1.0; 11 MAR 2015
Version 2.0; 08 JUL 2015
Version 3.0; 10 NOV 2016
Version 4.0; 15 AUG 2017

Signatures:

Juliano Timm, PhD MPH<br>Microbiology Consultant, TB Alliance	Date
Daniel E. Everitt, MD<br>Vice President and Senior Medical Officer, TB Alliance	Date

TABLE OF CONTENTS

2. ABBREVIATIONS 5

3. CONTACT DETAILS 6

4. INTRODUCTION 7

5. MYCOBACTERIOLOGY LABORATORY TESTING 8

6. STANDARD OPERATING PROCEDURES (SOP) 12

6.1. SOP 1: Sputum Handling 13

6.2. SOP 2: Preparation of Samples for Storage and/or Shipment 16

6.3. SOP 3: Sputum Decontamination 19

6.4. SOP 4: Acid-fast Bacilli (AFB) Microscopy *24*

6.5. SOP 5: Hain Line Probe Assays (LPA) 30

6.6. SOP 6: GeneXpert MTB/RIF (or Xpert MTB /RIF) 36

6.7. SOP 7: Liquid Culture by Mycobacteria Growth Indicator Tube (MGIT) 41

6.8. SOP 8: MPT64 Antigen Test 48

6.9. SOP 9: DNA Extraction 51

6.10. SOP 10: Drug Susceptibility Testing (DST) by Mycobacteria Growth Indicator Tube (MGIT) 56

6.11. SOP 11: Minimum Inhibitory Concentration (MIC) 63

6.12. SOP 12: Whole Genome Sequencing (WGS) 76

Figures

Figure 1: Schematic Microbiology Testing for NiX-TB 8
Figure 2: Flowchart for Processing Screening Sputum Samples 10
Figure 3: Flowchart for Processing of Sputum Samples During Treatment and Follow-up Phases (Day 1 - Month 24) 11
Figure 4: Scanning Scheme for Smear Examination 27
Figure 5: Diagram Showing Work Flow in a PCR Laboratory 35
Figure 6: Example of GeneXpert View 38
Figure 7: Algorithm for Interpretation of Positive MGIT Cultures 44
Figure 8: Example of printed barcodes 67
Figure 9: Example of TBeXiST worklist 71
Figure 10: Example of a TBeXiST plot, annotated to show the 'resistant' and 'Susceptible' growth curves relative to the Growth Control 72
Figure 11: Example of TBeXiST Interpretation Report 73

Tables

Table 1: Schedule of Visits with Microbiological Assessments 9
Table 2: SOPs Applicable to Each Laboratory Type 12
Table 3: Sputum Samples Collected During the Trial 13
Table 4: Storage of Microbiological Samples 16
Table 5: WHO/IUTLD Grading Scale for 200x (Auramine Staining) 27
Table 6: WHO/IUATLD Grading System for ZN Smears 28
Table 7: Uses of LPAs in NiX study 30
Table 8: Interpretation of the MPT64 Antigen Assays 49
Table 9: Preparation of DST Drug Stock Solutions 57
Table 10: Working concentrations of DST Drugs 59
Table 11: Manufacturer and storage instructions for the study drugs 64
Table 12: Drug concentrations used for MGIT MIC testing 64
Table 13: Drug concentrations used for MGIT Drug Sensitivity Testing 65
Table 14: MIC cut-off values for each drug, for a sample to be considered resistant and therefore require further testing 74
Table 15: Acceptable MIC range for the MTB H37Rv reference strain quality control 74
Table 16: Contact Details for Shipment of DNA from UCL to PHE 77
Table 17: PHE SOP list 79
Table 18: Data files generated during the sequence analysis 82

Version History:

Master Version Number/Date	Change
1.0/11 March 2015	Initial version
2.0/08 Jul 2015	Re-organized sections and procedures/content to align with NC-006 lab manual where appropriate
3.0/10 Nov 2016	• Updated central laboratories' contact details.<br>• Added suggested Laboratory Reporting Forms (LRF), as well as new sample transport forms.<br>• Reorganized summary figures.<br>• Changed storage/shipment of positive isolates, including where they are shipped and which assessments are performed on isolates in each location.<br>• Added additional smear and speciation assessments to ensure proper clinical care and follow up of subjects.<br>• Consolidated all SOPs related to AFB microscopy into one.<br>• Consolidated all SOPs related to Hain LPA into one.<br>• Updated SOP on MGIT culture (SOP 8).<br>• Introduced a new SOP (SOP 9) on MPT64 antigen testing.<br>• Updated SOP on DST by MGIT (SOP 11).
4.0/15Aug2017	• Updated UCL Central Laboratory contact details and deleted information on TASK central laboratory.<br>• Deleted all references to *pncA* sequence analysis, including corresponding SOP and forms.<br>• Added SOPs for Minimum Inhibitory Concentration (MIC) and Whole Genome Sequencing (WGS).<br>• Updated Laboratory Reporting Forms (LRF) and sample transport form.<br>• Updated Drug Susceptibility Testing (DST) SOP.<br>• Deleted any reference to St. Andrews Central Laboratory

This Version Number/Date	Change
5.0/15May2018	Updated Whole Genome Sequencing (WGS) SOP and related forms

2. ABBREVIATIONS

AFB	Acid Fast Bacilli
ATCC	American Type Culture Collection
B	Bedaquiline
BA	Blood Agar
BSC	Biological Safety Cabinet
CL3/BSL3	Containment Level 3
CQIF	Continuous Quality Improvement Form
CRF	Case Report Form
DMSO	Dimethyl Sulfoxide
DST	Drug Susceptibility Testing
E	Ethambutol
EMS	Early Morning Sputum
FQ	Fluoroquinolones
GC	Growth Control
I	Isoniazid
HYB	Hybridization Buffer
IQC	Internal Quality Control
K	Kanamycin
L	Linezolid
LIMS	Laboratory Information Management System
LM	Laboratory manual
LRF	Laboratory Report Form
MGIT	Mycobacteria Growth Indicator Tube
MIC	Minimum Inhibitory Concentration
MIN	Minute(s)
M	Moxifloxacin
MOTT	Mycobacteria other than *M. tuberculosis*
MTB	*Mycobacterium tuberculosis*
MTBC	Mycobacterium tuberculosis complex
NALC	N-Acetyl L-Cysteine
NaOH	Sodium Hydroxide
OADC	Oleic Acid Albumin Dextrose Complex
OD	Optical Density
Pa	Pretomanid
PANTA	Polymyxin B, Amphotericin B, Nalaxidic acid, Trimethoprim, Azlocillin
PBS	Phosphate Buffered Saline
PCR	Polymerase Chain Reaction
PPE	Personal protective equipment
PCR	Polymerase Chain reaction
QC	Quality Control
R	Rifampicin
S	Streptomycin
SIRE	Streptomycin, Isoniazid, Rifampicin, Ethambutol
SOP	Standard Operating Procedure
STR	Stringent Wash Solution
T	Temperature
TTP	Time to Positivity
UCL	University College London
Z	Pyrazinamide
ZN	Ziehl-Neelsen

3. CONTACT DETAILS

<table>
<tr><th colspan="6">Isolates from baseline and at/after end of treatment (if applicable),
Local Laboratory to send to:</th></tr>
<tr><th colspan="2"></th><th>Contact Person</th><th colspan="3">Contact Details</th></tr>
<tr><td rowspan="5">UCL Central Laboratory</td><td rowspan="2">To discuss routine shipment receipt and courier requests</td><td>Anna Bateson</td><td>E-mail</td><td>a.bateson@ucl.ac.uk</td><td rowspan="5">DEPARTMENT OF CLINICAL MICROBIOLOGY
ROYAL FREE HOSPITAL CAMPUS
ROWLAND HILL STREET
LONDON NW3 2PF UNITED KINGDOM</td></tr>
<tr><td>Julio Ortiz Canseco</td><td>E-mail</td><td>julio.canseco@ucl.ac.uk</td></tr>
<tr><td rowspan="3">As permit consignee for shipments</td><td rowspan="3">Prof Timothy McHugh</td><td>Tel</td><td>+44 20 7472 6402</td></tr>
<tr><td>Fax</td><td>+44 207 794 0433</td></tr>
<tr><td>E-mail</td><td>t.mchugh@ucl.ac.uk</td></tr>
</table>

4. INTRODUCTION

The NiX-TB clinical trial is a phase 3 open-label trial assessing the safety and efficacy of bedaquiline (B) plus pretomanid (Pa) plus linezolid (L) in subjects with pulmonary infection of either extensively drug-resistant tuberculosis (XDR-TB) or treatment intolerant / non-responsive multi-drug resistant tuberculosis (MDR-TB).

Throughout the study, basic microbiological assays will be conducted at a number of study laboratories receiving samples directly from the sites, whereas characterization assays will be conducted at the central laboratory, University College London (UCL) Central Mycobacteriology Laboratory – as follows:

- Study Laboratories:
  - On screening sputum samples: perform acid-fast bacilli (AFB) smear microscopy, a molecular test – GeneXpert MTB/RIF or HAIN MTBDR*plus* (Version 2) – and MGIT culture to detect *Mycobacterium tuberculosis* (MTB) and obtain time-to-positivity (TTP).
  - On all sputum samples collected post-screening: perform MGIT culture to detect MTB and obtain TTP. In addition, at one baseline visit [preferably the early morning sputum (EMS) at Day 1 or Screening to Week 4 if Day 1 cultures are contaminated or negative) and all visits Week 16 and beyond, speciate the AFB positive MGIT cultures to confirm presence of MTB complex (MTBC) bacteria.
  - Store all non-contaminated, AFB positive MGIT cultures until the end of the trial.
  - Ship isolates obtained at baseline and at/after end of treatment (only first positive visit) to UCL for further characterisation.

- UCL Laboratory:
  - Performs drug susceptibility testing (DST) to multiple antibiotics in liquid culture, and minimum inhibitory concentration (MIC) determinations for the study drugs, on isolates obtained at baseline and at/after end of treatment.
  - Extracts DNA from these isolates and co-ordinates whole genome sequencing (WGS) – shipment of the DNA to a designated reference laboratory, data analysis and reporting of results generated
  - Stores the isolates and DNA for a maximum of 5 years after the trial closure.

The diagrams and tables that follow before the start of the SOPs give an overall orientation to the individual assays, when and where they are performed.

This manual is used in combination with the following documents:

- Appendix A – Sputum Specimen Transport Form
- Appendix B – LRF1: Screening Samples
- Appendix C – LRF2: Treatment and Follow-up Samples
- Appendix D – LRF3: Speciation
- Appendix E – Isolate Shipment to UCL Laboratory
  (Study Lab - complete one per shipment to UCL)
- Appendix F – LRF4: Drug Susceptibility Testing (used by central lab)
- Appendix G – LRF5: Minimum Inhibitory Concentration (used by central lab)
- Appendix H – LRF6: Paired Whole Genome Sequencing (used by central lab)
- Appendix I – DNA Quantification Worksheet (used by central lab)
- NiX-TB (B-L-Pa) Protocol
- NiX-TB Laboratory Quality Control Manual

5. MYCOBACTERIOLOGY LABORATORY TESTING

The Mycobacteriology laboratory testing that occurs at each laboratory (study and central laboratories) is summarized in Figure 1

Figure 1: Schematic Microbiology Testing for NiX-TB

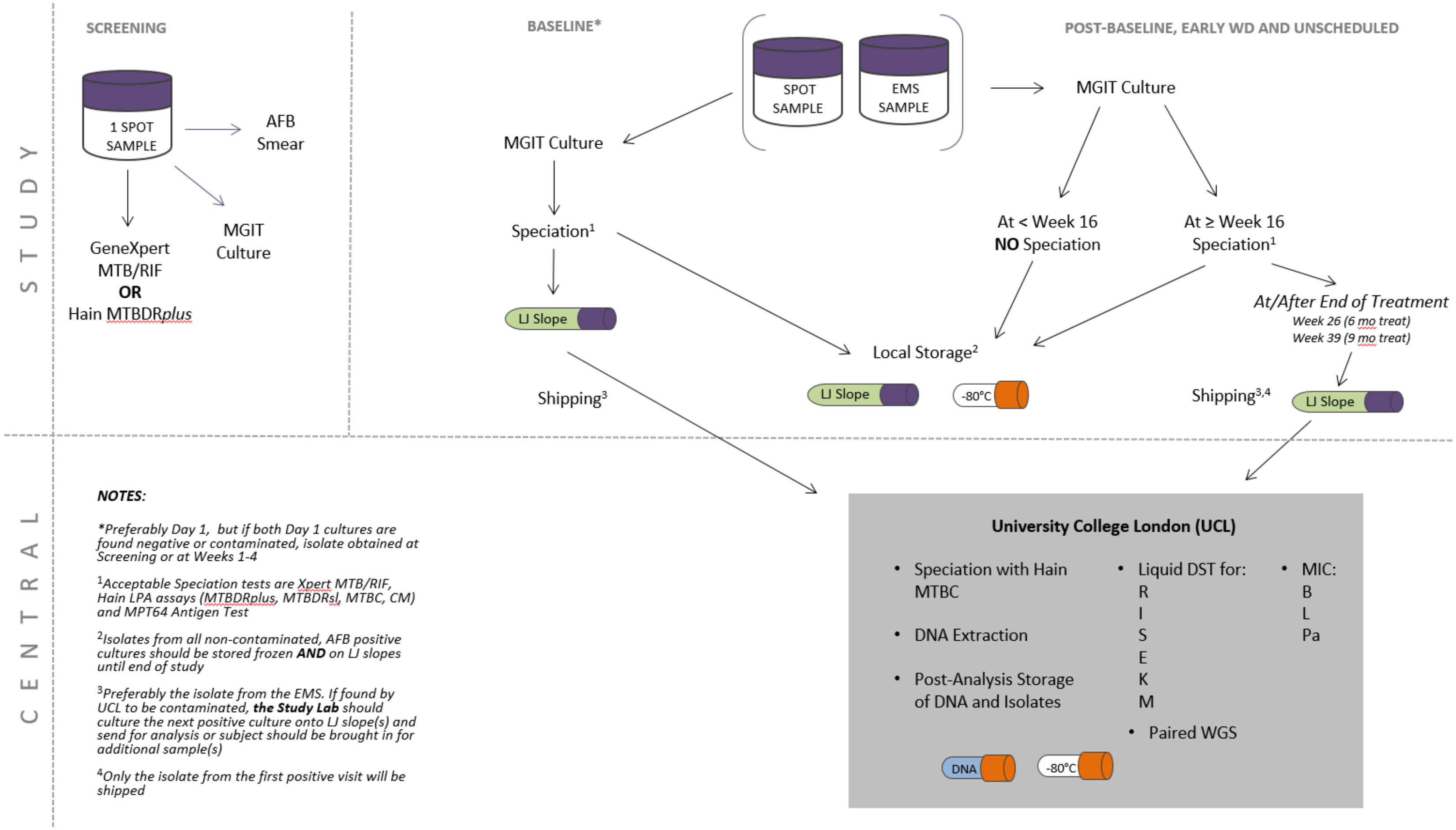

Table 1: Schedule of Visits with Microbiological Assessments

Screening	Treatment	Follow Up Post End of Treatment
Day -9 to Day -1	Day 1	1 month
	Week 1	2 month
	Week 2	3 month
	Week 4	6 month
	Week 6	9 month
	Week 8	12 month
	Week 12	15 month
	Week 16	18 month
	Week 20	21 month
	Week 26	24 month
	Week 30 (9 months treatment)	
	Week 34 (9 months treatment)	
	Week 39 (9 months treatment)	
	**Unscheduled**	
	**Early Withdrawal**	

Figure 2: Flowchart for Processing Screening Sputum Samples

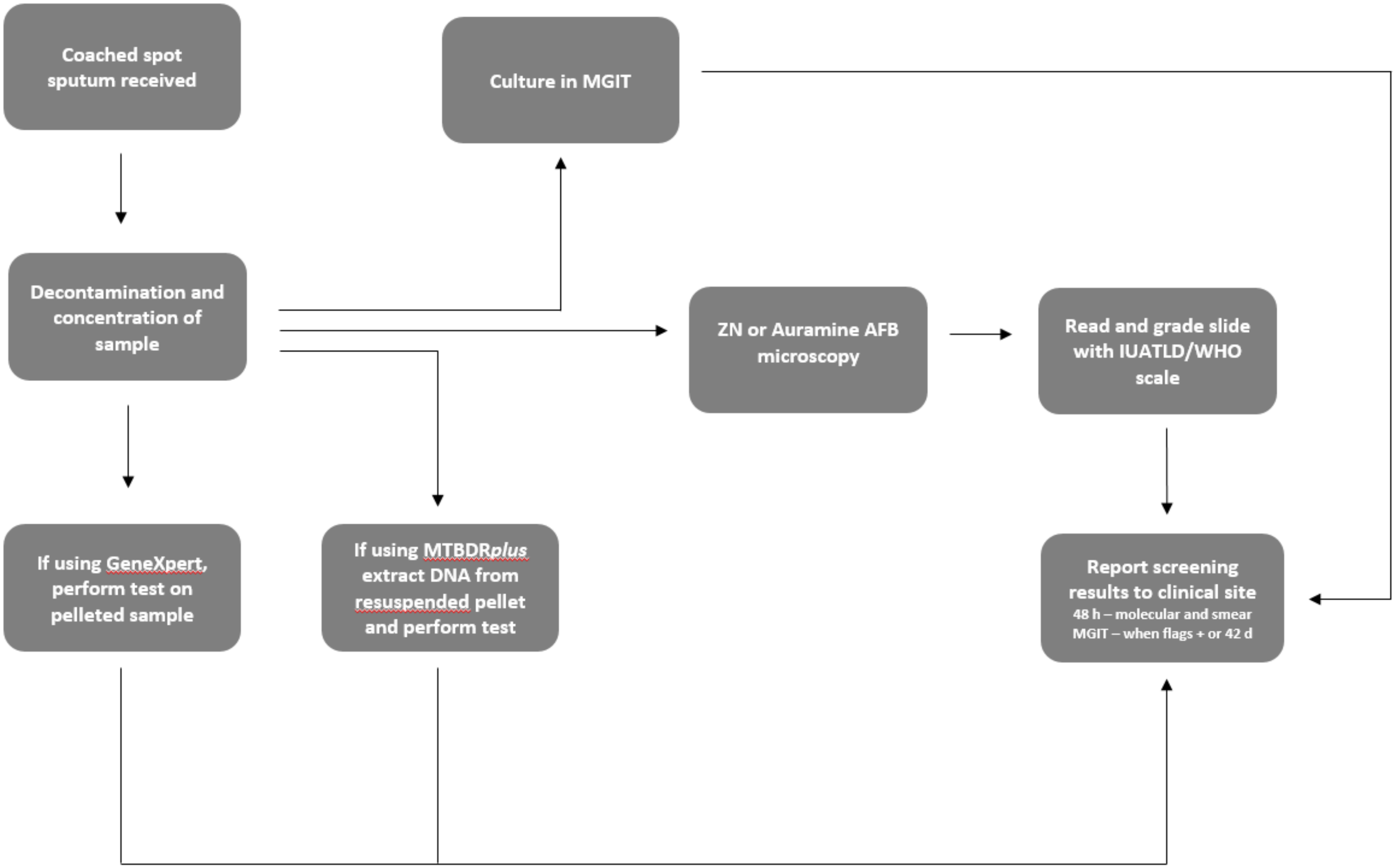

Figure 3: Flowchart for Processing of Sputum Samples During Treatment and Follow-up Phases (Day 1 - Month 24)

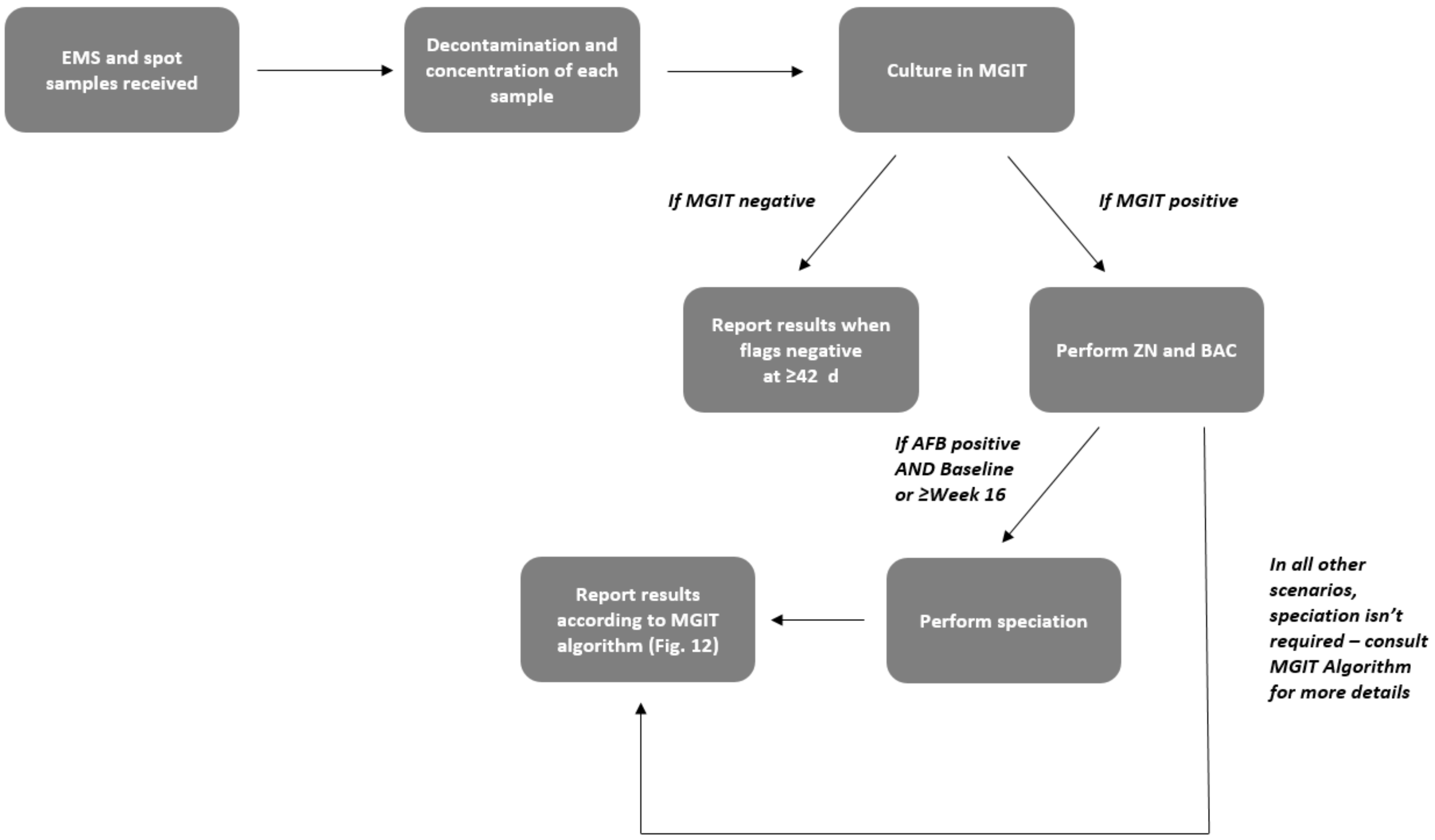

6. STANDARD OPERATING PROCEDURES (SOP)

The SOPs which apply to each mycobacteriology laboratory type are described below.

Table 2: SOPs Applicable to Each Laboratory Type

Laboratory	Role	SOPs
Study Laboratories	Mycobacteriology assessments during Screening, Treatment and Post-Treatment phases:	
	• AFB smear microscopy to confirm presence of AFB in sputum samples or cultures	1, 3, 4
	• Molecular test to detect MTB in Screening sputum samples	1, 3, 5, 6
	• MGIT culture to detect MTB and obtain TTP	1, 3, 7
	• Speciation for confirmation of presence of MTBC bacteria	5, 6, 8
	• Storage and shipping of MTB isolates	2
UCL Central Mycobacteriology Laboratory	Mycobacteriology Characterisation:	
	• Speciation for confirmation of presence of MTBC bacteria	5
	• DST in the MGIT System	10
	• MIC determination	11
	• Storage of MTB isolates	2
	• Whole Genome Sequencing	9, 12

6.1. SOP 1: Sputum Handling

6.1.1. Purpose

Proper collection and transport of sputum specimens is required to ensure quality laboratory results. Upon receipt of the specimens, proper labelling must be verified before processing the specimens. Prior to and during shipping of samples correct handling procedures must also be followed.

6.1.2. Principle

Sputum specimens in this study are collected in the early morning at home or while in the hospital ("early morning" samples), and as spot samples at the research site. These are delivered to the study microbiology laboratory where they are received, checked and logged-in prior to any tests being performed.

6.1.3. Procedure

6.1.3.1. Receipt of specimen at the study laboratory

The samples described in Table 3 will be received at the study laboratory.

Table 3: Sputum Samples Collected During the Trial

Timing	Specimens Collected
Days -9 to -1 (Screening)	One spot sputum collected at the research site under the coaching and observation of the trial staff.
• All visits from Day 1 up to and including Month 24 follow-up visit.<br>• If both sputum samples given at Weeks 16, 26 (if 6 mo treatment), 39 (if 9 mo treatment) or Month 24 (follow up) are contaminated, subject should be brought back in to give additional samples.<br>• Unscheduled visits: subject should be brought back for an unscheduled visit to collect sputum to ensure that the following conditions are met at end of treatment/end of follow-up or at early withdrawal:<br>o Two sequential negative sputum results.<br>o Two sequential positive sputum results.<br>o Unable to produce sputum after documentation of two negative sputum cultures with no intervening positive and are clinically asymptomatic<br>• Early Withdrawal Visit	Two sputum samples:<br>• One early morning sputum (EMS) collected and brought by subject from home or collected in hospital.<br>• One spot sputum collected at the site under the coaching and observation of the trial staff.<br>• **Alternatively,** two spot sputa collected at the site, in case the patient did not bring an EMS sample.

6.1.3.2. Logging-in of sputum specimen at the study laboratory

1. On receipt of samples at the laboratory:
   - Measure the temperature of the transport box and ensure the temperature is not below 2° C or above 8° C.
   - Assign a unique laboratory accession number to the specimen. The laboratory accession number is used to label tubes for all subsequent downstream processing of this specimen (e.g. cryotubes, MGIT tubes, agar plates, microscope slides, etc.) and for the reporting of data on the approved laboratory source documentation, case report form (CRF) and results.

2. Complete the Study Lab Specific Specimen Transfer Form (the form used by lab that receives samples directly from the site; Appendix A or equivalent)
   - Place a laboratory accession number label on the form for each specimen;
   - Complete the date and time the samples are received; the temperature from the maximum/minimum thermometer inside the transport container; and whether the specimen has been received within 24 hours of collection.
   - Perform a visual check of the specimens to confirm they are in good condition (i.e. the sample does not contain only saliva, or excessive blood quantity and is of appropriate volume) and complete this information onto the form.

3. Check if the specimen label details match those on the Specimen Transport Form.
4. If the specimens are not processed within 30 min of receipt at the laboratory, place in the designated sputum refrigerator (2-8 °C) and record the time and fridge identifier on the Specimen Transfer Form.
5. The specimen register should be completed to link the specimen details with the accession number.
6. The Specimen Transfer Form must be stored at the laboratory in the study laboratory file. A copy of the Specimen Transfer Form may be sent back to the clinic and/or data office as appropriate

6.1.3.3. Timing of specimen receipt

1. The laboratory must process all specimens as soon as possible **and no later than 48 hours after the specimen is collected.**
2. Logistical issues related to the personnel shift or appropriate arrangements for specimens shipping must be pre-arranged to meet this timeframe.
3. If laboratories are closed on weekends or for public holidays, short-term storage for no more than 48 hours is acceptable provided that:
   - appropriate refrigerated conditions (2-8 °C) are maintained,
   - the sputum is in good condition (i.e. the sample does not contain only saliva, or excessive blood quantity and is of appropriate volume),
   - the Specimen Transfer Form is completed as described above,

If the sample is processed out of the window period (more than 48 hours after collection), contact TB Alliance in order to assess the validity of the data and the specimens.

6.1.3.4. Handling of specimen receipt issues

If the delegated laboratory staff finds leaking specimen containers, mis-labelling, incomplete labelling, incomplete or mismatching specimen labels and accompanying forms, follow the procedures listed below.

Leaking container

Carefully scrutinize the specimen containers to determine any sample leakage. Liquid seen could be due to condensation inside the pathoseal bag only. Do not open the pathoseal bag if the specimen container leaked. Instead, after marking the

specimen as "LEAKED" in the Specimen Transfer Form, seal the leaked specimens in a biohazard autoclave bag and discard following the biowaste lab procedures.

Immediately notify the clinical site, who will decide if another specimen(s) could be collected.

Mis-labelled specimens

The Specimen Transfer Form and specimen study specific labels must be fully completed and match. If they do not match, the laboratory must contact the clinical site to obtain any outstanding/missing information before the specimen is processed. The contact with the clinical site must be documented in writing and signed and dated.

Specimens without the matching forms are **not** to be processed until a fully completed Specimen Transfer Form or clarification is received. If attempts to correct the forms cannot ensure proper identification of the specimen, the collection should be repeated.

Unlabelled specimens

Unlabelled specimens will **not** be processed unless the shipment contains multiple specimens from a single visit for a subject. In this case, the clinical site will be contacted and asked to resolve the discrepancies and complete the labelling process. Pending this correction, the specimen will be stored in the laboratory refrigerator.

If there are more than one subject's samples in a shipment (or the information on samples from a single subject's visit cannot be obtained within the appropriate time period), the specimen(s) will be discarded and new sample(s) will be requested.

Specimens arriving outside the designated temperature range

Every effort should be made to maintain the sputum samples within the specified temperature range (2-8°C) during all the steps prior to sample processing (i.e. soon after the collection of the sample at the clinical site and during transportation to the laboratory). This is essential to minimize the growth of any contaminating bacteria that may be present in the sputum sample. If a sputum sample arrives at the laboratory outside of the temperature range, a repeat sample should be requested as soon as possible. If it is not possible to obtain another sample (i.e. the patient has left the clinic), the original sample must be processed to avoid losing the time-point.

Small volume samples

Every effort should be made by the site clinical team to ensure a good quality sputum sample of sufficient volume (>2 ml) is collected. Sputum samples cannot be pooled to increase volume. If the sputum sample is less than 2 ml and a good quality specimen, it should still be processed. Although sputum processing is less accurate when the specimen volume is less than 2 ml (because the sputum pellet is re-suspended in 1.5 – 2.0 ml of PBS after centrifugation), it is still valuable to determine whether acid fast bacilli can be detected. The volume must be noted as less than 2.0 ml in the approved laboratory source documentation so these samples can be excluded from the quantitative culture analyses [e.g. Mycobacteria Growth Indicator Tube (MGIT) and Time-To-Positivity (TTP)].

6.1.4. Documentation

Suggested Specimen Requisition Form and Laboratory Report Form (LRF) 1 (if the specimen is from Screening) or LRF2 (for all other visits) are found in Appendices A to C. Study Laboratories may use their own forms, or a Laboratory Information Management System (LIMS), as long as they capture all the required information related to sputum collection, transport and receipt for completion of the electronic CRF (highlighted fields in the LRFs). The use of these alternate forms will have to be approved by TB Alliance.

6.2. SOP 2: Preparation of Samples for Storage and/or Shipment

6.2.1. Purpose

The samples described in Table 4 are stored and/or shipped in this trial.

Table 4: Storage of Microbiological Samples

Sample	Prepared and Stored by	Stored Samples	Storage Period	Shipped Samples
MTB isolates from all AFB+/BAC- cultures	Study Laboratory	For each isolate:<br>• 1 LJ culture stored at room temp.<br>• 1 culture at -70-80°C in 50% glycerol stored at study lab	Until trial closure	If from baseline* or the first positive* at/after end of treatment (after confirmed positive for MTBC):<br>• 1 LJ to UCL
MTB DNA and isolates after mycobacteriological characterisation	UCL Laboratory	• Isolates at -70-80°C in 50% glycerol<br>• DNA at -20°C	A maximum of 5 years after trial closure	DNAs shipped to WGS laboratory<br>• from all baseline isolates<br>• from MTB isolated at the first positive sample at/after end of treatment, for which there is a matching baseline counterpart

*See notes below

NOTES:

- **Only one baseline isolate for each patient** will be shipped to UCL Central Laboratory. In order of preference: 1) the isolate obtained from the EMS (or spot sputum) collected at Day 1; or 2) the first positive culture from Screening up to Week 4, in this order of predilection: Screening > Week 1 > Week 2 > Week 4. For all visits, whenever both EMS and spot cultures are positive, the isolate from the former is to be shipped.
- **Only isolates confirmed to be MTBC** by any speciation test (SOPs 5, 6 or 9) will be shipped to UCL.

The purpose of this SOP is to describe the methodologies for preparation and required storage conditions of these samples.

6.2.2. Procedures

<u>**Storage of positive MGIT cultures**</u>

The **Study Laboratory** will store all MGIT positive cultures found to contain AFB (ZN positive) and no contamination (BAC negative). Two samples should be stored for each isolate, one on an LJ slope and the other in 50% glycerol at -70°C to -80°C, for the duration of the trial. A logbook must be kept of all isolates in storage.

The **UCL Central Laboratory** will store all isolates after mycobacteriology characterisation testing is complete, in 50% glycerol at -70°C to -80°C, as well as their DNAs at -20°C, for a maximum of 5 years after trial closure

All procedures are to be carried out inside a biosafety cabinet (BSC), using the appropriate biosafety procedures and personal protective equipment (PPE)

Equipment and Reagents
- Biological Safety Cabinet (BSC)
- Discard bucket containing appropriate mycobacteriocidal disinfectant (specified in local Health and Safety documentation)
- Cotton wool or paper towels soaked in mycobacteriocidal disinfectant
- Cryovial (with rubber o-ring seal), and appropriate storage box
- Sterile micropipettes and aerosol resistant tips (ART), or disposal pipettes
- Sterile PBS/Middlebrook 7H9 medium
- Sterile glycerol 50% (in PBS or 7H9 medium)
- LJ slope

Storage on LJ slope
To inoculate a LJ slope, vortex well the MGIT tube, open the lid, and using a micropipette or disposal pipette, take 100 - 200µl of the culture and pipette onto the slope. Securely fasten and label with both the patient number, the lab accession number, and the date. Once growth is obtained these positive slopes will be stored in a rack in a cool dark place. To maintain the isolates, LJ slopes should be sub-cultured every 6 months (unless required earlier because the slope is disintegrating).

Storage at -70-80°C
Spin down the MGIT culture and resuspend the deposit with 1 ml of 50% glycerol (in PBS or 7H9 medium) and transfer into a cryovial (with rubber o-ring seal in lid). Securely fasten and label with both patient number, the lab accession number (also handwrite this number in permanent marker in case sticker is removed during freezing) and date. Place in an appropriate storage box and freeze at –70°C to -80°C.

If more than one positive culture is generated from a visit (e.g, early morning and spot); the resulting cryovials should be stored in separate freezers (if possible).

LJ slopes for shipment to UCL Mycobacteriology Laboratory
Study Laboratory will also inoculate a LJ slope for shipment to UCL Central Laboratory, at baseline (it is acceptable to use Screening to Week 4 isolates if both Day 1 cultures are contaminated or negative) **and** at or after the end of the treatment period. **Only one isolate**, preferably the one obtained from the EMS, will be shipped in either case.

The LJ slopes will be shipped to the UCL on a 6-weekly basis. LJ slopes should be sealed with parafilm, wrapped in absorbent tissue in bubble wrap, placed securely inside sealable plastic hazard container, inside sealed cardboard hazard box. Packaging should be conforming to IATA ICAO P1620 or as required by qualified courier company. This box should be labelled with appropriate hazard handling labels for IATA class 6.2, category A UN2814 Infectious Substance.

Complete the isolate shipment log (Attachment Q) and send a copy with the shipment and keep the original for records.

The UCL Central Laboratory should be contacted (see Contacts Table on page 7 for full details) to agree on a suitable shipment date, and be informed when the package is collected (providing the airway bill number). The Study lab is expected to make its own arrangements with the courier.

6.2.3. Documentation

The suggested LRF2 (Appendix C) captures the information for completing the eCRF fields (highlighted) related to isolate storage and shipment. Study Laboratories may use their own forms or LIMS as long as these pieces of information are included, and they are approved by the Sponsor.

In addition, when shipping samples to UCL Central Laboratory, Local Laboratories must complete Appendix E: Isolate Shipment to UCL (when shipping isolate for DST, MIC and/or WGS analysis), send the signed original along the shipment, and keep a copy in the Study File.

6.3. SOP 3: Sputum Decontamination

6.3.1. Purpose

Sputum processing has two major functions: liquefy the specimen and decontaminate of bacteria other than mycobacteria. Although there are several techniques available, none are ideal, i.e., none of them will selectively kill only contaminating flora and achieve complete liquefaction of the specimen. A reasonable compromise is to kill as much of the contaminating bacteria as possible while harming as few mycobacteria as possible.

At screening, only one spot sample is collected, decontaminated and used for the assays detailed in Figure 3. At all other visits, an EMS and a spot sputum are collected, decontaminated and cultured in the MGIT System.

6.3.2. Principle

The decontamination process is carried out using N-Acetyl-L-Cysteine – Sodium Hydroxide (NALC-NaOH), (Equivalent commercially available reagents, e.g. Mycoprep or Alpha Tec NAC-PAC, can also be used as approved by TB Alliance). NALC, a mucolytic agent, is used for rapid digestion, enabling NaOH, the decontaminating agent, to be used at a lower final concentration than that required in the absence of NALC – in this study, the range 1% - 1.5% is acceptable. Sodium citrate is also included in the decontamination solution to chelate heavy metals ions, which if present in the specimen may inactivate the NALC. Phosphate buffered saline is used to neutralise the NaOH and dilute the homogenate to lessen the viscosity and specific gravity prior to centrifugation.

NOTE: If the specimen has a significant quantity of blood mixed with it (not just blood tinged), do not use NaOH-NALC method because NALC does not work in the presence of blood. Use NaOH method (4% NaOH only; 1:1 (v/v) with sputum sample).

6.3.3. Procedure

Decontamination of sputum is to be carried out inside a BSC, using the appropriate biosafety procedures and PPE

Equipment and Reagents

- 2.9% sodium citrate
- 4% NaOH
- NALC powder
- Sterile, break-resistant glass bottle
- 50 ml conical, graduated polypropylene centrifuge tubes with tight screw cap and appropriate rack
- Biohazard bags
- Biological safety cabinet (BSC)
- Benchguard (or alternative work surface protection)
- Appropriate PPE
- Ethanol 70%
- Pipette and aerosol resistant tips (ART)
- 3 ml Pasteur pipette
- New microscope slides, frosted one side and one end, clean and dry
- Paper towel soaked in mycobacteriocidal disinfectant, in case of spills
- Pencil or grease pen for labelling slides
- Permanent marker
- Phosphate buffered saline (pH 6.8)
- Plastic box (if available)
- Refrigerated centrifuge with sealed buckets and inserts suitable for 50 mL tubes

- Sterile (6 ml) graduated pipette
- Test tube rack for 50 ml centrifuge tubes
- Timer
- Vortex mixer
- Waste containers (including splash proof receptacle for liquids containing mycobacteriocidal disinfectant)

Specimen Registration

1. Sputum samples should be processed as soon as possible and **no longer than 48 hours** after the sample is produced to reduce the risk of contamination and maximize the recovery of viable mycobacteria. Logistics for specimen transportation should be pre-arranged so samples are processed within the specific timeframe. **Samples should be refrigerated if they are not processed within 30 min of receipt in the laboratory.**
2. Double check patient data and laboratory accession numbers. Laboratory accession labels should have been attached to the specimen container and the accompanying laboratory source documentation on receipt of the sample.
    - In addition, attach laboratory accession labels to:
        - 50 ml centrifuge tube for NaOH/NALC decontamination process
        - Plastic box for storage of decontaminated specimen
        - MGIT tube used to culture the specimen.
    - The patient screening number or patient number (patient identifier - post enrolment) are also written in permanent marker on all tubes and containers that will subsequently contain the patient specimen.
    - For screening samples only, a microscope slide is labelled with the laboratory accession number and the patient screening number using a pencil or grease pen.

The specimens and all of the labelled bottles and slides are then ready to be processed.

3. The patient details and laboratory accession number are entered into specimen log book or study register. The study visit for which the specimen has been collected (e.g. Screening, Baseline, Week 1 etc.) is also recorded.

Preparation of BSC and Good Microbiology Practices

1. If not used previously that day, the BSC should be allowed to purge for 20 minutes prior to working inside it, and a smoke test done or anemometer used to ensure the functionality thereof.
2. Using absorbent cotton wool or paper towels, decontaminate the BSC interior with the appropriate mycobactericidal agent, followed by 70% ethanol, and dry with a paper towel.
3. Place the necessary equipment, consumables and waste containers inside the BSC. Decontaminate these as well with the appropriate mycobactericidal agent, followed by 70% ethanol.
4. Place a clean Benchguard/ absorbent paper sheet (or alternative work surface protection) inside the BSC.
5. Place a discard bucket with a biohazard bag containing mycobactericidal agent inside BSC for disposal of contaminated materials.
6. Do not work with more specimens than can be placed in the centrifuge at one time. When processing multiple batches on the same day, clean the BSC with disinfectant, turn on UV lights (if available) for 20 minutes, and allow air to circulate in the BSC for 20 minutes between batches, prior to repeating steps 1-5.
7. Place specimen tubes at least one space apart in rack to aid in preventing contamination.
8. Work methodically with the tubes on one side and discard buckets close to the specimens, to avoid spillage/confusion of samples.
9. To reduce the risk of cross-contamination, ensure that reagent containers do not come in contact with the edge of the specimen container.
10. Remove only one cap at a time from the tubes (specimen collection tubes, culture tubes) to avoid cross-contamination and misplacing the caps on the wrong tubes.
11. Ensure that tubes, bottles, racks, pipette aid, etc. that are removed from the safety cabinet are free from any droplets/potential contaminants. If necessary, wipe the rack or tube with a paper towel soaked in mycobactericidal agent prior to removal from the BSC. In addition, if any suspicion of droplets from a specimen is seen on gloves, wipe gloves with a disinfectant-soaked towel and change gloves prior to processing additional specimens.

12. Upon completion of work, place paper towels in discard bucket. Wipe all BSC surfaces with mycobactericidal disinfectant, followed by 70% ethanol, let stand 3 minutes and wipe dry. If BSC is turned off in the evening, be sure to leave on for 1 hour before turning off. If available, turn on UV light inside BSC for at least 1 hour.

Preparation of decontamination mixture (NaOH/NALC/sodium citrate)

1. Add 500 ml 4% NaOH to 10 g NALC and mix gently to dissolve (do NOT shake vigorously).
2. Pour into a sterile, break-resistant glass bottle.
3. Add 500 ml 2.9% Sodium Citrate to the 500 ml of 4% NaOH/NALC solution. Mix gently. This is the working solution of the decontamination mixture (2% NaOH; 1% NALC; 1.45% sodium citrate) and is stable for 24 hours if stored at 2-8°C.
4. If a smaller volume is required, adjust accordingly e.g. add 200 ml 4% NaOH to 4 g NALC, mix gently and pour into an appropriately sized sterile, break-resistant glass bottle. Add 200 mL sodium citrate to the NaOH/NALC mix to give 400 mL working solution.
5. Transfer some of the working solution into a sterile tube and use this to add to the specimens. This avoids contaminating the stock bottle.

If an equivalent commercially available option has been approved by TB Alliance (e.g. Mycoprep or NAC-PAC) refer to the manufacturer's instructions. Mycoprep contains a concentration of NALC of 0.5% whereas for this study 1% is required – as for the in-house preparation of NaOH-NALC. Therefore, additional NALC powder should be added to the MycoPrep to obtain a similar concentration (i.e. to obtain 1%, 0.75 g of NALC powder must be added to 150 ml MycoPrep).

NOTE: TB Alliance must agree **before** any change is made to the concentration of the decontamination solution

Process of decontamination using NALC/NaOH/sodium citrate

1. Before processing specimens, prepare a waste container with disinfectant at the appropriate concentration and place a paper towel soaked in disinfectant (according to the Local Health and Safety Guidelines) on the work surface inside the BSC.
2. Ensure refrigerated specimens and reagents have been brought to room temperature before processing.
3. Switch on the centrifuge so that it can start to cool to 4-12°C.
4. A batch should **not** consist of more than 7 patient specimens in total.
5. Include a negative control with each batch of specimens (maximum total of 8 tubes per batch, see 'Quality Control' section below).
6. Transfer specimen into a 50 ml centrifuge tube with a screw cap. Make a note of the volume on the approved laboratory source documentation. For samples more than 10ml, see instructions at the end of this section. DNA stored at -80°C
7. Immediately add the NaOH-NALC sodium citrate solution in a volume equal to the quantity of specimen. Tighten the cap.
8. Start the timer.
9. Vortex for 15-30 seconds. Invert the tube so all contents are exposed to NaOH-NALC solution
10. Repeat steps 7, 8 and 10 for the subsequent specimens at 30 seconds or 1 min intervals.
11. It is important to mix well during the decontamination period to expose all the sputum to the digestion solution. It is preferable to use a shaker platform to improve homogenization. However, if a shaker is unavailable, vortex the tubes gently (to mimic the shacking action) 2-3 more times during the incubation period.
12. Make sure the specimen is completely liquefied. If still mucoid, add a further small quantity of NaOH-NALC sodium citrate solution. Mix well with the vortex again.
13. After 20 minutes, add phosphate buffered saline (PBS, pH 6.8) up to 50 ml. **Addition of sterile water is not a suitable alternative for phosphate buffer.** Mix well (lightly vortex or invert a few times). Continue to add the PBS to all specimens at 30 seconds or 1 min intervals (as above), so each specimen is **only** exposed to decontamination solution for 20 minutes; mycobacteria will be killed off if exposed to NaOH for longer than the stipulated time.
    NOTE: if using the Alpha Tec NAC-PAC Red Kit, add buffer until the red colour disappears. It might not be up to the 50 ml mark.
14. Transfer tubes in a 50 ml tube rack to the centrifuge.

15. Place the tubes in the centrifuge bucket, ensuring that they are equally balanced, and that the biosafety covers have been put in place for each centrifuge bucket. The centrifuge should be pre-cooled.
16. Centrifuge the specimen at a speed of 3,000 g (**NOT** 3,000 rpm, the centrifuge must be calibrated) for 15 min at 4°C (see Notes).
17. After centrifugation, remove centrifuge buckets and place in the BSC before opening. Do not open the buckets on the open bench in case there has been a spillage or breakage during centrifugation.
18. Carefully decant as much of the supernatant as possible into a suitable splash proof container (discard container) containing a mycobactericidal disinfectant (according to the Local Health and Safety Guidelines). **Make sure the sediment is not lost during decanting of the supernatant fluid**. The discard container must contain an appropriate starting concentration of disinfectant such that the final concentration of the disinfectant after addition of all the supernatants is still sufficient to kill *M. tuberculosis*.
19. Add 2 ml of phosphate buffered saline (pH 6.8) to the sediment using a sterile pipette/3mL Pasteur pipette and re-suspend it using a pipette or vortex mixer if required. Use the re-suspended pellet to prepare smears for acid-fast bacteria (AFB) microscopy (screening samples only; SOP 4) and for inoculation of MGIT tube (SOP 8).
20. Store any leftover sediment in the bijou container (or, if not available, in the original 50 ml tube) at 4°C, for 10 days until it is confirmed the inoculated media are not contaminated.
21. If contamination is detected in the MGIT culture within 10 days, the decontamination procedure should be repeated with this remaining sediment following exactly the same procedure and new culture inoculated.

NOTE: For large volume samples (greater than 10 ml) there is not sufficient space in the 50 ml tube to complete the process. For these samples, note the total volume on the approved laboratory source document, then split the sample into two approximately equal volumes. Process the samples separately, and combine back into a single tube when adding PBS (add 1 ml to each sample) to re-suspend the sediment (step 19). Use the combined sample for microscopy and Hain MTBDR*plus*/GeneXpert (where applicable) and to inoculate a MGIT tube.

6.3.4. Quality Control

A negative control tube is added in the middle of each batch of specimens processed in order to ensure that there is not contamination present in stock solutions and no carry-over of MTB from one specimen to another. The negative control must be treated the same way as the patient samples. The negative control is included in microscopy, Hain MTBDR*plus* (where applicable) and MGIT culture. If there is only one specimen in the batch a negative control is not required.

If a positive signal – presence of AFB or other microorganism on the slide, or MTB confirmed by MTBDR*plus*, or growth on the MGIT culture is detected from the negative control, **notify the TBA Microbiology Consultant immediately.** In addition,

- Observe the technician who processed these specimens for issues known to contribute to contamination; e.g., poor organization of tubes in the BSC, splashing when adding reagents, opening caps too soon after vortexing etc.
- Visually check all reagents used, and subculture to blood agar (BA) if contamination is suspected.
- Monitor closely the patient samples processed in that batch closely for expected results.

NOTES:

- The NaOH-NALC reagent contains strong alkali and causes severe burns. NaOH is irritating to the eyes and skin. Gloves and eye/face protection must be worn when working with NaOH. In the event of eye or skin contact, rinse immediately with an eye wash system or tap water for at least 15 min and seek medical advice. If ingested, seek medical advice.
- All sample processing related to sputum culture must be done in a class I BSC in a CL3/BSL3 Laboratory unless BSC class I is not available. A class 2A BSC laboratory is acceptable if it is under negative pressure.
-
- NALC loses activity rapidly in solution, so it **must** be made fresh daily.
- The final pH of the specimen concentrate greatly affects the recovery and time-to-detection of mycobacteria.
- High pH will lower the positivity rate and increases the time-to-detection of positive culture and may also cause

transient false fluorescence.

- With NaOH-NALC digestion, do not agitate the tube vigorously. Extensive aeration causes oxidation of NALC and makes it ineffective.
- Always use a refrigerated and calibrated centrifuge. Temperature build up during centrifugation increases the killing effect on mycobacteria and adversely affect the positivity rate and time-to-positivity in cultures.

6.3.5. Documentation

Study Laboratories must report the date and time of specimen processing on LRF1 or LRF2, or sponsor-approved lab-specific forms or LIMS. In addition, record the order samples were processed and decontamination times using Quality Manual Attachment F.

6.4. SOP 4: Acid-fast Bacilli (AFB) Microscopy

6.4.1. Purpose

To detect acid-fast bacilli (AFB) by microscopic examination of clinical specimens and cultures. Both viable and non-viable bacilli will stain. A quantitative grading system (WHO/IUATLD scaling system) is used to report the number of AFB observed in stained smears from direct or concentrated sputum specimens. Results of sputum smears must be reported to the study staff within 48 hours of receipt of the sputum specimen.

6.4.2. Principle

In the NiX-TB study, fluorescent microscopy is the preferred method for examining screening samples. Like Ziehl-Neelsen (ZN), fluorescence staining includes a stain, decolouriser and counter stain. With auramine-O stain, organisms fluoresce bright yellow, non-specific debris stains pale yellow, and the background is almost black. With auramine-rhodamine stain organisms fluoresce yellow-red in an almost black background.

Brightfield microscopy, ZN staining, is used for confirming the presence of AFB in positive MGIT cultures and on LJ slopes if the colonies do not resemble MTB. The ZN method uses a carbol fuchsin stain, acid alcohol decolouriser and methylene blue counter stain. Acid-fast organisms stain purple and the background and debris stains blue. ZN can also be used for examining screening samples.

Kinyoun is a variant of the ZN method where cold carbol fuchsin stain is used.

6.4.3. Procedure

- Auramine-O smear microscopy is the preferred method for screening sputum specimens.

- ZN smear microscopy is performed:
  - As part of the MGIT Culture work-up process (SOP 8) to confirm the presence of AFB in the MGIT positive cultures.
  - To confirm the absence of AFB in decontamination negative controls.
  - On LJ slopes prepared for storage or to ship to UCL if the colonies do not resemble MTB.
  - As requested by the investigator when considering permanently withholding linezolid for the remainder of treatment.

- Kinyoun is used at **UCL Mycobacteriology Laboratory** to confirm the presence of AFB in cultures found resistance to any drug by the MGIT DST assay, or on MGIT/ LJ subcultures set up to prepare these assays.

Prior to the preparation of the smear, all sputum specimens are decontaminated and concentrated as described in SOP 3. Smears must be prepared, air dried and heat-fixed on the same day they are decontaminated.

Smear results should be reported as soon as possible, **no later than 48 hours of sample receipt**. Each time a batch of patient smears is prepared, a positive QC smear using MTB (control strains H37Rv/H37Ra) must be stained alongside the samples to ensure the quality staining of the slides.

Equipment and Reagents

- Aerosol Resistant Tips (ART)
- Biological Safety Cabinet (category CL3/BSL3 laboratory before heat-fixation of the slide)
- Mycobactericidal disinfectant (specified in the local health and safety guidelines)

- Distilled water (chlorine free)
- Hot plate (or slide warmer)
- Microscope slides, frosted at one end, new and unscratched
- Fixative (optional)
- Immersion oil
- Positive control slide used with each batch (containing *M. tuberculosis* H37Rv)
- Paper towel soaked in appropriate disinfectant
- Pasteur pipette (Pastette)
- Pencil for labelling slide
- Slide drying rack
- Staining rack
- Staining sink
- Slide storage box
- Wash bottle with distilled water
- Waste containers

<u>For auramine-O Stain or auramine-rhodamine stain:</u>
- Auramine-O or auramine-rhodamine staining kit
    - Or auramine/auramine-rhodamine staining solution, 0.5-1% acid alcohol, 0.5% potassium permanganate prepared in-house (see laboratory's procedure for preparation)
- Fluorescence or LED microscope
- Dark room

<u>For Ziehl-Neelsen stain:</u>
- Ziehl-Neelsen staining kit
    - Or carbol fuschin solution, 3% acid alcohol, methylene blue solution (see laboratory's procedure for preparation)
- Bunsen burner or electric heating block
- Light microscope
- Immersion oil
- Lens paper & lens cleaning solution

<u>For Kinyoun stain:</u>
- Carbol fuschin KF, 3% acid alcohol, brilliant green or methylene blue (see laboratory's procedure for preparation)

<u>Process</u>

<u>Step One: Preparation of smears</u>
Decontamination, culture inoculation and smear preparation must be performed before the screening sputum sample is removed from the BSC in the Containment Level 3 (CL3/BSL3) Laboratory. Prior to heat-fixation, the slides must remain in the BSC inside the CL3/BSL3 Laboratory.

1. Label the frosted end of a clean, dry, new and unscratched slide with the patient screening number, lab accession number and date using a pencil.
2. Vortex the decontaminated deposit to resuspend and mix thoroughly.
3. Transfer 50 µl of well-mixed resuspended pellet from the decontaminated sputum specimen onto the slide, using a pipette with sterile ART (or an appropriate loop or Pastette).
4. Spread sample, covering a circle approximately 2 cm in diameter. Allow the slides to air dry before heat fixing.
5. Place the slides for at least 15 minutes on a hotplate set between 65°C to 75°C to heat-fix the samples (or as specified in the local Health and Safety guidelines).
    - Once heat-fixed, smears can be stained outside the BSC in the CL3/BSL3 laboratory and can be examined

by microscopy in either the Containment Level 2 (CL2/BSL2) or CL3/BSL3 Laboratory once it is dry. Heat-fixing does not kill mycobacteria, so be careful when handling smears.

NOTES: if preparing smears from positive cultures, only step 2 will differ, as follows:

- From positive MGIT cultures, vortex MGIT tube well, unscrew cap and sample, using an ART or Pastette, an aliquot (approx. 50 µl or 2 drops) of broth onto the slide. Dispose of ART or pipette into the appropriate waste container.
- When examining colonies on solid medium, dispense approx. 50 µl of distilled water on the glass slide with an ART or Pastette. Using a sterile loop or disposable applicator stick, transfer 2-3 colonies to the water drop and gently mix to make a smooth, thin suspension. Dispose of loop or applicator stick into the appropriate waste container.

Step Two: Procedure for fluorescent staining (Auramine-O or Auramine-rhodamine)

1. Place slides on a staining rack so that they are at least 1 cm apart and flood with auramine / Auramine-rhodamine stain and let sit for 15 minutes.
2. Rinse the auramine / auramine-rhodamine away with distilled water and drain the slides.
3. Decolourise with 0.5% acid alcohol for 2 minutes.
4. Rinse again with distilled water and drain.
5. Flood slides with 0.5% potassium permanganate for 2 min.
6. Rinse potassium permanganate away with distilled water. Do not allow potassium permanganate to act longer than 2 min., or it might quench the fluorescence of AFB.
7. Air-dry the slides. **Do not blot!**
8. Protect smears from light by placing in a storage box and examine as soon as possible since the fluorescence diminishes quickly. **Examine smear within 24 hours of staining.**

NOTE: Include an AFB positive and AFB negative slide in each staining batch

Alternative Step Two: Procedure for ZN staining

1. Place the slides on the staining rack so that they are at least 1cm apart and flood with carbol fuchsin.
2. Heat the slide to steaming with the flame from a Bunsen burner. An electric heating block may also be used. Apply only enough additional heat to keep the slide steaming for 5 minutes. Do not let the stain boil or dry.
3. Re-flood the slide with fresh carbol fuchsin and heat again until steaming, then let stand for 5 minutes.
4. Wash away the carbol fuchsin with distilled water.
5. Flood slides with 3% acid alcohol.
6. Let stand for 2-3 minutes (more acid alcohol should be used if the liquid becomes heavily stained).
7. Wash away the acid alcohol with distilled water and drain the slides.
8. Flood the slides with malachite green (or methylene blue) and leave to stand for 1-2 min.
9. Wash away the malachite green (or methylene blue) with distilled water and tilt the slides to drain.
10. Allow slides to air dry in the slide rack. **Do not blot!**
11. Once air dry and immediately prior to examination of the slide, apply a drop of immersion oil.

Alternative Step Two: Procedure for Kinyoun staining

1. Place the slides on the staining rack and flood with TB Carbol fuchsin KF for 4 minutes. Do not heat.
2. Wash gently with distilled water.
3. Decolorize with TB decolorizer (3% acid alcohol) for 3-5 seconds.
4. Wash gently with distilled water.
5. Counterstain with either TB Brilliant Green K or TB Methylene Blue for 30 seconds.
6. Wash gently with distilled water.
7. Air dry. If using TB Methylene Blue, dry over gentle heat.
8. Apply a drop of immersion oil and a cover slip if needed to keep slides stored.

Step Three: Microscopic examination and reading of fluorescent stained smear

Examine the auramine-stained smears with a fluorescent source microscope.

1. Scan the entire smear with the 20x or 15x objective, with 10x eye piece (this is a 200X or 250X magnification).
2. Use a scanning pattern of rows either up and down the slide or across and back. Using 200 or 250X magnification, one 2 mm length is equivalent to 30 fields, which is sufficient to report a negative result.
3. Occasionally use the 40x or 45x objective for closer examination of the bacterial morphology.
4. Confirming morphology at higher magnification avoids false positive results due to fluorescing debris.
5. Examine the control slides first. If expected results are not observed, do not proceed to examine the slides from the screening spot sample.
6. The mycobacteria can appear as rod, coccoid, and filamentous shapes. In sputum smears, individual rods of MTB may be aggregated side-by-side or end-to-end to form "cords". Count bacilli in the number of fields appropriate for degree of positivity, e.g., the higher the smear positivity, the fewer fields need to be counted. Average the count for the number of fields and record on the lab's microscopy worksheet according to the WHO/IUTLD grading scale below.

Table 5: WHO/IUTLD Grading Scale for 200x (Auramine Staining)

**No of AFB per length/field (200x)**	**WHO/IUTLD Grading 200x Auramine**
No AFB per one length	No AFB seen/negative
1-4 AFB/one length	Confirmation required*
5-49 AFB/one length	Scanty
3-24 AFB/one field	1+
25-250 AFB/one field	2+
>250 AFB/one field	3+

Ref: http://www.stoptb.org/wg/gli/documents.asp?xpand=2

7. All slides must be kept for the duration of the study.

*Have another microscopist reexamine the smear and if still scanty, prepare another smear from the same specimen and examine before reporting results. If 1-4 AFB/one length are seen on repeat, report this as "Scanty".

Alternative Step Three: Microscopic examination and reading of ZN- or Kinyoun-stained smear

Examine the ZN-stained smears with a light (bright field) microscope and the 100x oil objective.

1. Examine the smears 100 fields using a regular pattern, such as the one shown in Figure 4. Start with the positive QC slide. If the QC slide is negative, do not report smear results obtained from that batch. Report and resolve the problem.
2. If smear is being done at investigator's request to possibly remove subject from linezolid treatment, record both the average number of AFBs and assign the corresponding grading as shown in Table 6. To confirm positive MGIT or LJ subcultures, only record whether smear was positive or negative for the presence of AFB.

Figure 4: Scanning Scheme for Smear Examination

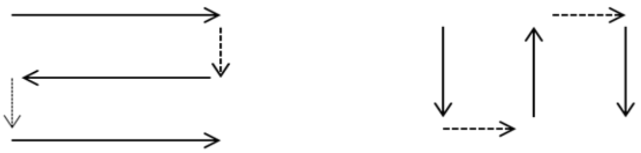

Table 6: WHO/IUATLD Grading System for ZN Smears

No. of AFBs (average over 100 fields)	WHO/IUATLD Reporting
None	No AFB seen (NS)
1-9 per 100 fields	Scanty
1-9 per 10 fields	+
1-9 per field	++
>9 per field	+++

Possible false results:
When atypical rods are seen, they may be other mycobacteria (pathogenic or non-pathogenic) or other partially acid-fast organisms.

The morphology should be broken down and analysed using the following categories to confirm and distinguish mycobacteria from any possible artefacts:
- Size (length and width)
- Colour (shade and intensity of stain)
- Shape (curved, straight, etc.)
- Pattern (beaded, banded, etc.)
- Distribution on smear (e.g. cording)
- Uniformity of appearance

Acid-fast artefacts may be present in the smear, therefore it is essential to view cell morphology carefully. Most artefacts show considerable variation while mycobacteria are uniform in size, arrangement, and staining patterns within a slide.

A few examples of the causes of artefacts (and possible solutions) are:
- Contamination of slides by tap water with saprophytic mycobacteria – **always use distilled water**.
- Spots of stain deposit (when slide is not properly decolorized) can be mistaken for AFB – **review the control slide to ensure slides were decolorized appropriately.**
- Waxes and oils in dirty specimen containers may appear as acid-fast particles or react with non-acid-fast bacteria and make them appear acid-fast.
- Heavy metal ions in staining solutions or high chlorine content in water interfere with the fluorescent staining and may disrupt the fluorescent adhesion to the mycobacteria.
- If the smear is too thick, debris may cover AFB and make it hard to visualize.
- If the smear is too thin, there may not be enough material to see, showing a low number of (or possibly no) AFB.

NOTE: Sputum smears from screening samples and investigator queries should be kept for the duration of the study in case any smear needs to be rechecked. Gently wipe the slide clean with a tissue and store in a labelled box in the BSL2 or BSL3 laboratory. For smears prepared from positive MGIT tubes, these can be discarded once the respective MGIT results have been reported. Discard these slides into a covered sharps bin inside the BSC in the BSL3 laboratory

6.4.4. Quality Control

The following QC is required:
- Each new shipment or lot number of staining reagents (for ZN/Kinyoun: carbol fuschin, malachite green/methylene blue or 3% acid alcohol; for fluorescent: auramine / auramine-rhodamine, 0.5% acid alcohol, 0.5% potassium permanganate) must be QC tested using a positive QC smear containing MTB (H37Rv/H37Ra) strain and a negative QC smear containing *E. coli*. Both the positive and negative controls must pass for the reagents to be used for staining samples. If the QC fails, repeat the test with new controls. **If the repeat test fails,**

do not use the reagents and contact the supplier.
Results of this QC are to be reported using NiX-TB Mycobacteriology Quality Manual Attachment Ei.

- Each time a batch of patient sputum smears is carried out, include a negative control (decontamination mixture only) and a positive QC smear containing MTB (H37Rv/H37Ra strain) to check that each stage of the procedure is working correctly. The positive control slide must pass for the microscopy results to be reported. If the positive QC fails, new smears from all samples in the batch must be prepared and re-stained.
  Results of this QC are to be reported using Quality Manual Attachment F and H.
- Each batch of MGIT samples stained should also include a positive control slide containing MTB (H37Rv/H37Ra), to check the staining procedure has been performed correctly. If the positive control fails, then the results from the cultures cannot be relied on and should be repeated with a new positive QC slide.
  Results of this QC are to be reported using Quality Manual Attachment H.

For every ten screening slides examined:

1. A review by a second person (i.e. Delegated Laboratory Staff, DLS) is required and the results recorded independently using the **NiX-TB Mycobacteriology Quality Manual Attachment I.**
2. The results for the WHO/IUATLD scaling system should be the same for both counts. If the results do not match:
   - Inform the Laboratory Manager.
   - A third person will count and confirm results. The two equivalent accounts from the three are the final result.
   - Re-train staff member who got the results wrong.

6.4.5. Documentation

<u>For screening specimens:</u>
Report the date slide was read and the result of reading, following the WHO/IUATLD scaling system, in LRF1, or sponsor-approved lab-specific form or LIMS.

<u>For AFB presence confirmation in cultures:</u>
Report the date slide was read and the result of reading in LRF2, or sponsor-approved lab-specific form or LIMS.

6.5. SOP 5: Hain Line Probe Assays (LPA)

6.5.1. Purpose

Used by the **Study Laboratories** as a rapid test to confirm the presence of MTB in screening sputum, as well as in AFB positive MGIT cultures from Baseline and at/after Week 16.

It will also be used by **UCL Mycobacteriology Laboratory** to confirm the speciation result obtained by the Study Laboratory prior to any additional characterization work.

Table 7 below details which specific LPAs can be used on each situation.

Table 7: Uses of LPAs in NiX study

Sample / Laboratory	LPA (GenoType) Test	Comment
Screening sputum at Study Laboratories	MTBDR*plus* **(Version 2 only)**	**Only** if GeneXpert was not used
AFB pos. MGIT culture at baseline and at/after Week 16, at Study Laboratories	MTBC, Mycobacterium CM, MTBDR*plus*, MTBDR*sl*	**Only** if MTP64 Ag test was not used, or was used but the test result was “invalid” or was “negative” and presence of MTBC is strongly suspected
Baseline and first MTBC pos. MGIT culture at or after end of treatment, at UCL Laboratory	MTBC	

6.5.2. Principle

Line probe assay technology involves the following steps: First, DNA is extracted from a culture of mycobacteria or directly from decontaminated and concentrated clinical specimens. Next, polymerase chain reaction (PCR) amplification of the resistance-determining region of the gene under question and/or a genomic region allowing for speciation is performed using biotinylated primers. Following amplification, labeled PCR products are hybridized with specific oligonucleotide probes immobilized on a strip. Captured labeled hybrids are detected by colorimetric development, enabling detection of the presence of MTBC or the species within the complex, as well as the presence of wild-type and mutation probes for resistance. If a mutation is present in one of the target regions, the amplicon will not hybridize with the relevant probe. Mutations are therefore detected by lack of binding to wild-type probes, as well as by binding to specific probes for the most commonly occurring mutations. The post-hybridization reaction leads to the development of coloured bands on the strip at the site of probe binding and is observed by eye, or using a specialized scanner (GenoScan).

In this study, DNA will be isolated at the study labs from sputum samples or cultures using the GenoLyse method, which is described below (see Appendix 1 for more details). UCL central lab will extract DNA by boiling/sonicating method, which is also described below. For a description of the LPA method, consult the instructions provided by Hain Lifesciences for the selected LPA – **taking into consideration the available test version.**

6.5.3. Procedures

<u>Equipment and reagents required for DNA extraction</u>

- Absorbent paper
- Calibrated thermometer
- Microfuge with fixed rotor angle
- Graduated cylinders
- Biological safety cabinet (BSC)

- 95°C water bath
- Micropipettors, 20-200 µL, 100-1000 µL
- Aerosol resistant tips (ART)
- 3 ml Pasteur Pipettes (Pastettes)
- 1.5 ml screw-cap Eppendorf tubes
- Appropriate Disinfectant
- Genolyse Lysis Buffer (A-LYS)
- Genolyse Neutralisation Buffer (A-NB)

Equipment and reagents not provided in the kits required for Amplification
- PCR tubes (DNase and RNase free)
- Dedicated Laboratory Gowns + hooks to hang the gowns on
- Nitrile disposable gloves (Small, Medium, Large)
- Cover Shoes
- Spray bottle for 1% freshly prepared sodium hypochlorite
- Spray bottle for 70% alcohol
- PCR Hood with UV-lamp
- Bacterial DNA extracts
- Aliquoted master mix
- Thermal Cycler (heating rate: 3°C/sec, cooling rate: 2°C/sec, precision: +/- 0.2°C)
- Micropipettes (P20, P200)
- Pipette stand
- Aerosol-resistant tips (ART)
- Discard container with plastic bag and 1% freshly prepared for sodium hypochlorite
- Medical waste box for contaminated material
- Marker Pen

Equipment and reagents not provided in the kits required for Detection
- Shaking water bath or TwinCubator
- Sterile water (molecular biology grade)
- Graduate cylinder
- Timer
- Tweezers
- Vortex
- Waste receptacles (including splash proof receptacle for liquids containing appropriate liquid disinfectant)
- Water bath or heating block (set to 95°C)

Process

Preparation of BSC

1. The BSC should be allowed to purge for 20 minutes prior to beginning work. Whilst the BSC is purging, the heating block / water bath (filled with tap water) should be switched on and have reached the desired temperature of 95°C before commencing the DNA extraction procedure. The temperature must be verified by placing a suitable thermometer (up to 110°C), into the appropriate hole in the water bath.
2. Disinfect the BSC with the appropriate mycobactericidal disinfectant, followed by 70% ethanol.
3. Clean all surfaces, pipettes and tip boxes, with 1% sodium hypochlorite, followed by 70% ethanol.

Step One: DNA isolation from decontaminated sputum and from positive cultures

DNA isolation is to be carried out inside a BSC, using the appropriate biosafety procedures and PPE

If using bacteria from sputum or grown in liquid media:

1. For each sample, label the sides of two 1.5 ml screw-capped Eppendorf tube(s) with the laboratory no. The first tube will be used for the DNA extraction process and the second will receive the isolated DNA.
2. Using a 1-3 mL sterile, disposable Pasteur pipette, transfer 500µl of the decontaminated sputum into a labelled 1.5mL screw cap tube; when using bacteria grown in liquid media, transfer 1mL.
3. Centrifuge for 10 minutes at 10,000 x g in a standard table top centrifuge with aerosol tight rotor.
4. Using a Pasteur pipette, carefully remove the supernatant. If the pellet has been aspirated, place the contents back into the same tube and centrifuge again.
5. Discard supernatant into a 50 mL tube filled with 10 mL of 3.5-5% sodium hypochlorite.
6. Resuspend pellet in 100 µl Lysis Buffer (A-LYS) by vortexing. No clumps should be visible.
7. Incubate for 5 minutes at 95°C in a water bath. Briefly spin down.
8. Add 100µl Neutralisation Buffer (A-NB) and vortex sample for 5 seconds.
9. Spin down for 5 minutes at full speed in a standard table top centrifuge with an aerosol tight rotor.
10. Using a P200 micropipette and ART, immediately pipette 100 µl of the supernatant (which now contains the DNA) slowly and carefully into the second labelled Eppendorf tube. Directly use 5µl of the supernatant for PCR. The remainder of the sample should be stored at - 20°C. Switch off the heating block / water bath. If a water bath was used, empty the contents thereof. Clean all surfaces, pipettes, tip boxes, storage boxes, etc. with 1% sodium hypochlorite followed by 70% ethanol. Disinfect the BSC with the appropriate mycobactericidal disinfectant, followed by 70% ethanol.

If using bacteria grown on solid media:

1. Collect bacteria with an inoculation loop and suspend in 100 µl Lysis Buffer (A-LYS), vortex, and continue to steps 7-10 above.

<u>Alternative Step One: DNA isolation from positive cultures for speciation by GenoType MTBC **(UCL Only)**</u>

1. When using bacteria grown on solid medium (LJ slopes), collect bacteria with an inoculation loop and suspend in approximately 300 µl of water (molecular biology grade)
2. When using bacteria grown in liquid media, directly apply 1 ml into a suitable tube and spin down for 15 minutes in a standard centrifuge tube with an aerosol tight rotor at approx 10000 x g. Discard supernatant and re-suspend the bacteria in 100-300 µl of water (molecular biology grade) by vortexing.
3. Microfuge briefly after vortexing to ensure all liquid is in the bottom of the tube before heat killing (step 4).
4. Incubate bacteria from 1 or 2 for 30 min at 95°C (water bath).
5. Incubate for 15 minutes in an ultrasonic bath
6. Spin down for 5 minutes at full speed and directly use 5 µl of the supernatant for PCR. In case DNA solution is to be stored for an extended time period, transfer supernatant to a new tube.

<u>Step Two: Amplification</u>

Observe the usual precautions for amplification set-up (see 'Good laboratory practice when performing molecular amplification assays' at the end of this SOP). It is essential that all reagents and materials used in the set-up for DNA isolation and amplifications are free from DNAses.

From this point on strictly follow the instructions provided by Hain Lifesciences for the selected LPA (PDF files of the Instructions for Use provided by Hain with each kit are available as Appendices 2-6.

6.5.4. Quality Control

Each time the test is performed a positive and negative control must be run alongside samples.

- Positive control: A suspension (0.5 McFarland) of a MTB control strain (H37Rv/H37Ra) subjected to the 3 steps mentioned above – DNA isolation (GenoLyse or boiling/sonication), PCR amplification, and hybridization.

- Negative control: molecular grade water subjected to the same 3 steps mentioned above.

Results of this QC are to be reported using NiX-TB Mycobacteriology Quality Manual Attachments L.

6.5.5. Documentation

Report test results either using LRF1, if it is a Screening specimen, or LRF3, if the visit required speciation (baseline or Week 16 or beyond). Alternatively, lab-specific forms or LIMS may be used as long as the required fields (highlighted in LRF1 and LRF3) are present and approved by the Sponsor.

A photocopy of Evaluation Sheet provided with the kit or local equivalent, with the developed strips (covered with a clear adhesive tape) should also be kept along the result report form.

6.5.6. Good Laboratory Practice When Performing Molecular Amplification Assays

6.5.6.1. Introduction

This SOP describes key elements of how to organise facilities for polymerase chain reaction (PCR) testing including workflow, reagents, consumables and staff within a molecular diagnostic laboratory.

The ability of PCR to produce large numbers of copies of a target sequence from minute quantities -sometimes single copies - of DNA has provided the exquisite sensitivity that makes PCR a powerful diagnostic tool. However, this ability also necessitates that extreme care be taken to avoid the generation of false-positive results.

False-positive results can result from sample-to-sample contamination and, perhaps more commonly, from the carry-over of DNA from a previous amplification of the same target.

Careful consideration should be given to facility design and operation within clinical laboratories in which nucleic acid amplification-based assays are performed. This document describes procedures that will help to minimise the carry-over of amplified DNA.

6.5.6.2. General Considerations

<u>Organisation of Work</u>

Practise good housekeeping policy at all times. Do not keep tubes or reagents any longer than necessary. All reagents, reaction tubes etc. should be clearly labelled. Records of batch numbers of all reagent batches used in individual assays should be kept.

Avoid entering pre-amplification rooms immediately after working in rooms where products, cloned materials and cultures are handled. If working with these materials is inconvenient or unavoidable, use of clean lab coats, gloves and hand washing is necessary. Change gloves frequently.

Ensure that all equipment, including paper, pens and lab coats are dedicated for use only in that particular laboratory (i.e. laboratory coat) for each of the PCR rooms. Workbooks and sheets that have been in contaminated areas shall not be taken into clean PCR areas.

PCR reagents should be aliquoted to avoid excessive freeze-thawing and to protect stock reagents if contamination occurs.

Pulse centrifuge tubes before opening the reagents. Uncap and close tubes carefully to prevent aerosols.
Bench areas in PCR laboratories should be wiped daily with hypochlorite solution or 70% ethanol following use.
All new members of staff, visitors and students must be trained in use of the PCR facilities.

6.5.6.3. Specimen Processing

Avoid molecular contamination problems of PCR through care (Good Laboratory Practice), being tidy and following the unidirectional workflow (see below).

Physical Separation of Pre-PCR and Post-PCR Assay Stages

To prevent carry-over of amplified DNA sequences, PCR reactions should be set up in a separate room or containment area from that used for post-PCR manipulations.

A complete separate set of necessary laboratory equipment, consumables, and laboratory coats should be dedicated for the specific use of pre- or post-PCR manipulations according to the area designation. Care must be taken to ensure that amplified DNA, virus cultures or DNA clones other than low copy number control material do not enter the 'Pre-PCR area'.

Reagents and supplies should be taken directly from storage into the pre-PCR area and should never be taken or shared with areas in which post-PCR analyses are being performed. Similarly, equipment such as pipettors should never be taken into the containment area after use with amplified material.

The Unidirectional Workflow

Workflow between these rooms/areas must be unidirectional i.e. from clean areas to contaminated areas, but not from contaminated areas to clean labs. Dedicated laboratory coats should be supplied for each area and gloves shall be changed between areas.

Reagent Preparation Clean Room (DNA –Free Room)

It is very important to keep this room/area free of any biological material (this includes DNA/RNA extracts, samples, cloned materials and PCR products).

Procedures carried out in this area include preparation and aliquoting of reagent stocks and preparation of reaction mixes prior to the addition of the clinical nucleic acid. Aliquoting of primers and other reagents is recommended to minimise any consequence of contamination and reduce assay downtime.

The Nucleic Acid Extraction Room

Extraction of nucleic acid from clinical samples must be performed in areas where PCR products and stocks of cloned materials have not been handled. A second clean area is thus required for this purpose. The second area is where the samples are processed, where the reverse transcriptase step of RT-PCRs is performed and where the extracted DNA or cDNA and positive control is added to the PCR reaction mixes (previously prepared in the reagent preparation room).

Specimens for PCR should come directly from the clean specimen receipt room into the extraction laboratory; the samples should never enter rooms where PCR products and cloned DNA are present.

The Amplification Room

The amplification room is the area in which the PCR machines are housed. It may also contain a containment area in which, for nested PCRs, the second round reaction mixes are inoculated with the primary reaction product. Cloned DNAs should not be brought into this area.

Where PCR machines are shared, a clear booking system is recommended to provide a cohesive system for the assays. Individual users' PCR programs in the thermocyclers should not be edited by other users (even temporarily) without notification to the program owner.

The Product Analysis Room

This is the room in which post-PCR manipulations are performed (e.g. - agarose gel electrophoresis of products, PCR-ELISA detection systems). This is a contaminated area and therefore no reagents, equipment, laboratory coats etc. from this room should be used in any of the other PCR areas.

Figure 5: Diagram Showing Work Flow in a PCR Laboratory

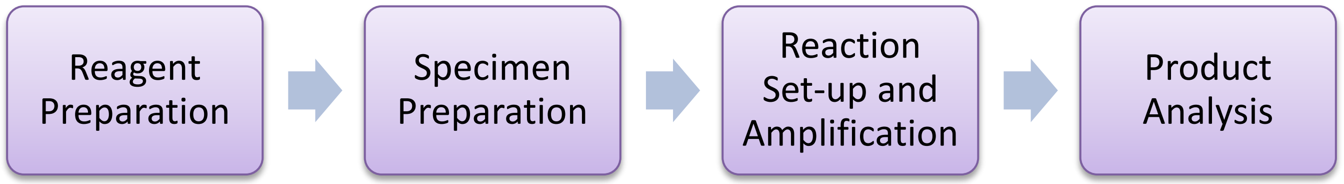

NOTE: Although four rooms are ideal, many laboratories only have two rooms available. Pre-PCR and extraction can therefore be carried out within defined areas of a larger laboratory and amplification and product analysis are in a second laboratory

6.5.6.4. References

Health Protection Agency National Standard Method 'Good Laboratory Practice when Performing Molecular Amplification Assays' Issue no. 3 Issue Date 02.08.06

6.6. SOP 6: GeneXpert MTB/RIF (or Xpert MTB /RIF)

6.6.1. Purpose

Used by the **Study Laboratories** as a rapid test for:

- Detecting MTBC bacteria on sputum samples at screening,
- Checking for rifampicin (R or RIF) resistance
- Confirmation of presence of MTBC bacteria on positive MGIT cultures at screening, Baseline and at/after Week 16.

NOTES:

- If Hain MTBDR*plus* is used (SOP 5) on screening specimens, do not perform GeneXpert.
- Only use GeneXpert for speciation of cultures if no other acceptable method (MPT64 Ag test or Hain LPA) is available.
- Ensure the correct software version is used for the cartridge.

6.6.2. Principle

Xpert MTB/RIF is an automated test that uses semi-quantitative, nested real-time PCR for the simultaneous detection of MTBC DNA an RIF resistance associated mutations in the *rpoB* gene in sputum samples (or cultures). The test requires no other instrumentation other than the GeneXpert System and provide results within 2 hours.

Basic step by step instructions required to perform the test are detailed below. For a full description of the system, protocol (e.g. preparing the cartridge) and QC procedures, as well as how to visualize figures and software settings see the Xpert MTB/RIF System Operator Manual – Appendix 2 of this manual.

6.6.3. Procedure

<u>**Equipment and Reagents**</u>

- Autoclave bags
- Barcode labels for cartridge identification
- GeneXpert Dx System equipped with $GX_{2.1}$ software (catalogue number varies by configuration):
    - GeneXpert instrument, computer, barcode wand reader, and Operator Manual
- Lockable container with appropriate disinfectant
- Micro tube 1.5 ml
- Rack able to hold 15 ml Falcon tubes
- Sterile Falcon tubes (16.5x120 mm, 15 ml)
- Sterile transfer pipettes (3.5 ml)
- Timer
- Vortex Mixer
- Xpert MTB /RIF kit (CGXMTB /RIF-10) [contains sufficient reagents to process 10 patient or quality-control specimens]

<u>**Process**</u>

<u>Step One: Preparing the sputum sediments</u>

1. Using a sterile transfer pipette, transfer 0.5 ml of re-suspended pellet (decontaminated sputum from SOP 2, not untreated sputum sample) to a conical, screw-capped tube.
2. Add 1.5 ml of Xpert MTB/RIF Sample Reagent (SR) to the 0.5 ml of re-suspended sediment using a sterile transfer pipette and shake vigorously 10 to 20 times (a single shake is one back-and-forth movement).
3. Incubate the specimen for 15 minutes at room temperature. Between 5 and 10 minutes of incubation, shake the

specimen vigorously again 10 to 20 times. Samples should be liquefied with no visible clumps of sputum. Particulate matter may exist that is not part of the sample.

Step Two: Preparing the cartridge

1. Label each Xpert MTB/RIF cartridge with the laboratory accession number by writing on the sides of the cartridge or attach ID label. Note: do not put the label on the lid or obstruct the existing 2D barcode on the cartridge.
2. Using the sterile transfer pipette provided with the kit, aspirate the liquefied sample into the transfer pipette until the meniscus is above the minimum mark. **Do not process the sample further if there is insufficient volume.**
3. Open the cartridge lid and transfer sample into the open port of the Xpert MTB/RIF cartridge. Dispense sample slowly to minimize risk of aerosol formation.
4. Close the cartridge lid and make sure the lid snaps firmly into place. Remaining liquefied sample may be kept for up to 12 hours at 2-8°C should repeat testing be required.
5. Load the cartridge into the GeneXpert Dx instrument and start the test within 30 minutes of preparing the cartridge.

Step Three: Starting the test

1. Before you start the test, ensure the system is equipped with the $GX_{2.1}$ software AND the Xpert MTB/RIF assay is imported into the software.
2. Turn on the computer, followed by the GeneXpert Dx instrument (if not already on).
3. On the Windows™ desktop, double-click the GeneXpert Dx shortcut icon.
4. Log on to the GeneXpert Dx System software using your user name and password.
5. In the GeneXpert Dx System window, click **Create Test**. The Scan Cartridge Barcode dialog box appears.
6. Scan the 2D barcode located on the Xpert MTB/RIF cartridge. The Create Test window appears. The software will automatically fill the boxes for the following fields: Select Assay, Reagent Lot ID, Cartridge SN, and Expiration Date based on the barcode information.
7. In the **Sample ID** box, scan or type the sample lab accession number. Cross-check to ensure it is typed or scanned correctly. The sample ID/lab accession number is associated with the test results in the "**View Results**" window and all generated reports.
8. Click **Start Test**. In the dialog box that appears, type your password.
9. Open the instrument module door with the flashing green light and load the cartridge.
10. Close the door. The green light will stop flashing and become steady once the test starts. When the test is finished, the green light will turn off and the system will release the door lock.
11. Once the system releases the door lock at the end of the run, open the module door and remove the cartridge.
12. Used cartridges are considered capable of transmitting infectious agents. Dispose the used cartridges according to your institution's and country's safety guidelines.

6.6.4. Quality Control

Each test includes a Sample Processing Control (SPC) and Probe Check Control(PCC):

- Sample Processing Control (SPC):
  Ensures the sample was correctly processed. The SPC contains non-infectious spores in the form of a dry spore cake that is included in each cartridge to verify adequate processing of MTB. The SPC verifies that lysis of MTB has occurred if the organisms are present and verifies that specimen processing is adequate. Additionally, this control detects specimen associated inhibition of the real-time PCR assay. The SPC should be positive in a negative sample and can be negative or positive in a positive sample. The SPC passes if it meets the validated acceptance criteria. The test result will be "Invalid" if the SPC is not detected in a negative test.

- Probe Check Control (PCC):
  Before the start of the PCR reaction, the GeneXpert Dx System measures the fluorescence signal from the probes to monitor bead rehydration, reaction-tube filling, probe integrity and dye stability. Probe Check passes if it meets the assigned acceptance criteria.

Incoming batches of Xpert MTB/RIF cartridges should be tested using a sample of cartridges and at least one known positive and one known negative specimen, to ensure expected performance. If this QC fails, contact Cepheid Technical Support.

6.6.5. Interpretation of Results

The results are interpreted by the GeneXpert DX System from measured fluorescent signals and embedded calculation algorithms and will be displayed in the "**View Results**" window of the GeneXpert machine. An example of this is shown in Figure 6 below.

Figure 6: Example of GeneXpert View

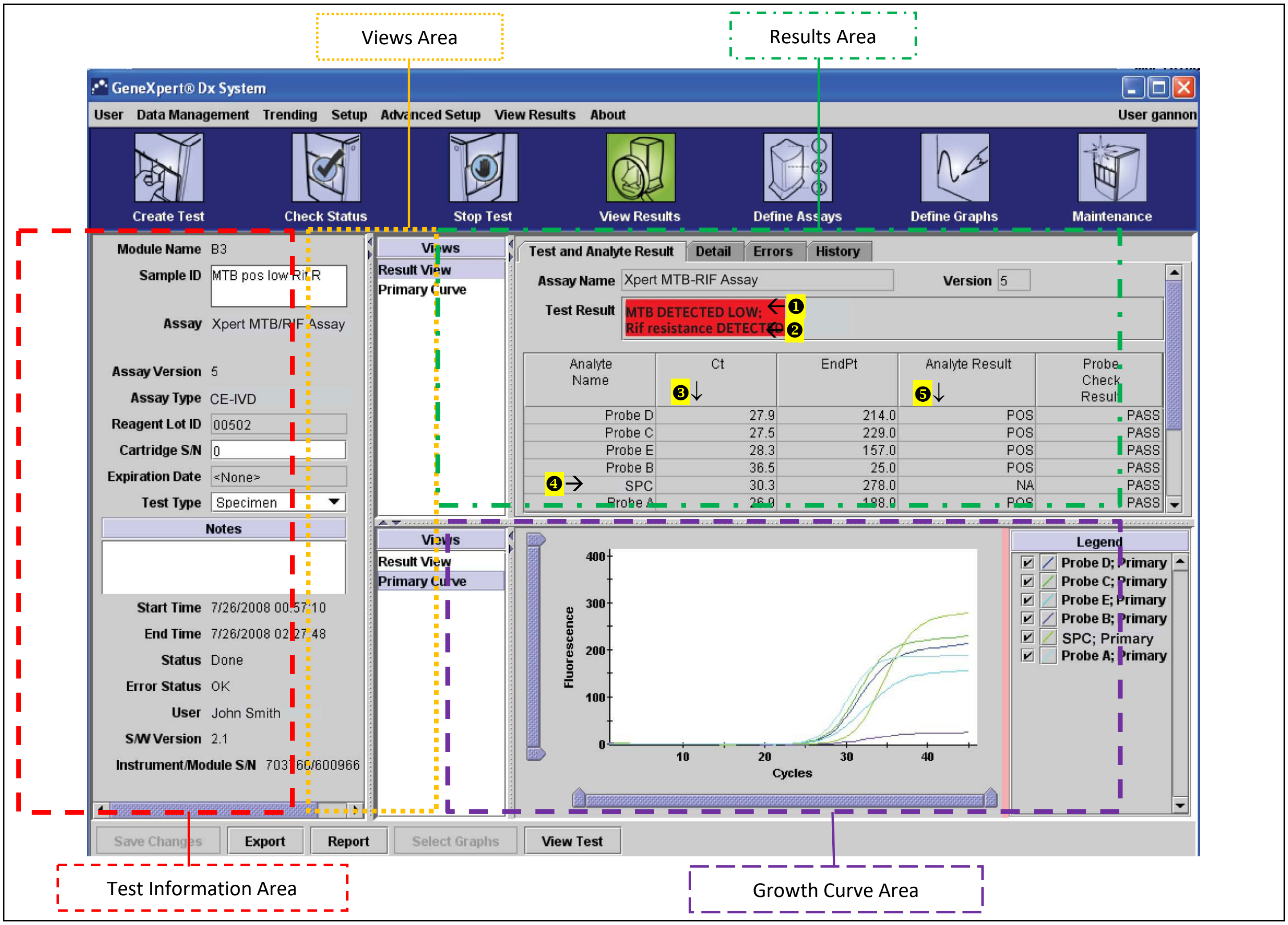

The results are read from the Results Area as follows:

In the "Test Result" field two results (if applicable) will be noted:

- The first result line (❶ in Figure 6) will relate to whether MTB is detected or not.
- The second result line (❷ in Figure 6) will relate to the Rifampicin resistance result will be recorded, if MTB has been detected.

In the "Ct column" (❸ in Figure 6) the starting concentration of the DNA template (sample) is displayed:

- Lower Ct values = a higher starting concentration;
- Higher Ct values = a lower starting concentration.

Quality Control is read:

- Sample Processing Control (SPC) Result (❹ in Figure 6) read in the SPC row.

- Probe Check Control (PCC) Column (❺ in Figure 6) read via the Analyte Result Column.

MTB Detected
MTB target DNA is detected.

- MTBDetected - The MTB result will be displayed as High, Medium, Low or Very Low depending on the Ct value of the MTB target present in the sample.
- Rif Resistance DETECTED, Rif Resistance NOT DETECTED, or Rif Resistance INDETERMINATE will be displayed only in MTBDETECTED results and will be on a separate line from the MTBDETECTED result.
- Rif Resistance DETECTED; a mutation in the *rpoB* gene has been detected that falls within the valid delta Ct setting.
- Rif Resistance INDETERMINATE; the MTB concentration was very low and resistance could not be determined.
- Rif Resistance NOT DETECTED; no mutation in the *rpoB* gene has been detected.
- SPC - NA (not applicable); SPC signal is not required since MTB amplification may compete with this control.
- Probe Check - PASS; all probe check results pass.

MTB Not Detected
MTB target DNA is not detected, SPC meets acceptance criteria.
- MTB NOT DETECTED - MTB target DNA is not detected.
- SPC - Pass; SPC has a Ct valid range and endpoint above the endpoint minimum setting.
- Probe Check - PASS; all probe check results pass.

RIF Not Detected
RIF target DNA is not detected, SPC meets acceptance criteria.
- RIF NOT DETECTED - RIF target DNA is not detected
- SPC - Pass; SPC has a Ct valid range and endpoint above the endpoint minimum setting.
- Probe Check - PASS; all probe check results pass.

INVALID
Presence or absence of MTB cannot be determined, repeat test with extra specimen. SPC does not meet acceptance criteria, the sample was not properly processed, or PCR is inhibited.
- MTBINVALID - Presence or absence of MTB DNA cannot be determined.
- SPC - FAIL; MTB target result is negative and the SPC Ct is not within valid range.
- Probe Check - PASS; all probe check results pass.

ERROR
- MTB - NO RESULT
- SPC - NO RESULT
- Probe Check - FAIL*; one or more of the probe check results fail.

*If the probe check passed, the error is caused by a system component failure.

NO RESULT
- MTB - NO RESULT
- SPC - NO RESULT
- Probe Check - NA (not applicable)

Reasons to Repeat the Assay

Repeat the test using a new cartridge or initiate alternate procedures if one of the following test results occurs:
- An INVALID result indicates that the SPC failed. The sample was not properly processed or PCR was inhibited.
- An ERROR result indicates that the Probe Check control failed and the assay was aborted possibly due to the reaction tube being filled improperly, a reagent probe integrity problem was detected, or because the maximum pressure limits were exceeded or there was a GeneXpert module failure.

- A NO RESULT indicates that insufficient data were collected. For example, the operator stopped a test that was in progress.
- Rifampicin resistance is indeterminate.

6.6.6. Documentation

Report test results either using LRF1, if it is a Screening specimen, or LRF3, if the visit required speciation (baseline or at/after Week 16). Alternatively, lab-specific forms or LIMS may be used as long as the required fields (highlighted in LRF 1 and LRF3) are present and they are approved by the Sponsor.

6.7. SOP 7: Liquid Culture by Mycobacteria Growth Indicator Tube (MGIT)

6.7.1. Purpose

Used by the **Study Laboratories** for culture of sputum samples for:

- Definitive diagnosis of MTB (presence or absence in the sputum sample).
- Assessment of the bacterial load in the original sample by determining the time taken for culture tube to signal positive (time-to-positivity, TTP; or time-to-detection, TTD) in a BACTEC MGIT instrument.

6.7.2. Principle

MGIT tubes contain a fluorescent compound embedded in silicone on the base of the tube. This complex is sensitive to the presence of oxygen dissolved in the liquid medium during continuous incubation at 37°C. The instrument monitors the tubes every hour for increasing fluorescence. The presence of fluorescence beyond a threshold identifies a tube as positive. An instrument positive tube contains approximately 10^5 to 10^6 CFU/ml.

The usual 'MGIT protocol', and the one adopted in this study, lasts 42 days. A MGIT culture remaining negative for 42 days and showing no other visible sign of growth is considered negative. And any culture flagged positive, must undergo confirmatory ZN stain microscopy, blood agar culture (BAC) (and speciation if applicable).

6.7.3. Procedure

Refer to the manufacturer's instructions for the overview of the MGIT instrument as well as detailed procedures (e.g. MGIT tubes preparation, incubation conditions, uploading and unloading of the tubes in the instrument).

NOTES:

- Blood samples are not suitable for use in the MGIT system.
- MGIT tubes can be prepared in a BSC outside of the Containment Level 3 (CL3/BSL3) laboratory. Inoculation of the MGIT tubes with sputum sediment and confirmatory testing of positive tubes must be carried out in the BSC in a CL3/BSL3 lab.
- Prior to use, examine all tubes and vials for evidence of contamination or damage - in particular, dropped tubes must be examined carefully for damage. Unsuitable or damaged tubes MUST be discarded.
- In the unlikely event of a broken tube in the machine – close the drawer and turn off the machine, evacuate the room. Local Health and Safety Guidelines should be followed for actions following a spill.

<u>**Equipment and Reagents**</u>

- BSC
- Benchguard (or alternative work surface protection)
- Appropriate PPE
- Mycobactericidal disinfectant (specified in local Health and Safety documentation)
- Ethanol 70%
- BACTEC MGIT 960 7 ml MGIT tubes
- MGIT PANTA
- MGIT Growth Supplement
- Graduated Plastic Pasteur Pipettes
- Discard bucket containing appropriate liquid mycobactericidal disinfectant
- Biohazard bags
- 1000 µL pipette and aerosol resistant tips
- Sterile, disposable 10 ml pipettes
- Markers

<u>Step One: Preparation of BSC and MGIT components</u>

1. Using absorbent cotton wool or paper towels, decontaminate the BSC with an appropriate mycobactericidal agent, followed by 70% ethanol.
2. Place the necessary equipment, consumables and waste containers inside the BSC. Then, decontaminate these as well, starting with the mycobactericidal agent, followed by 70% ethanol.
3. Place a clean Benchguard/ absorbent paper sheet (or alternative work surface protectant) inside the BSC.
4. Always ensure that the daily QC and maintenance of the instrument (see below) has been done and that the incubation protocol is set for 42 days prior to placing the inoculated MGIT tubes into the BACTEC MGIT 960 instrument.
5. Reconstitute MGIT PANTA (Polymyxin B, Amphotericin B, Nalaxidic acid, Trimethoprim, Azlocillin) with 15 m of MGIT OADC (Oleic Acid Albumin Dextrose Complex) Growth Supplement. Mix completely until dissolved. This supplement mixture is stable for 5 days if stored at 2-8°C (write the date of reconstitution on the bottle to document this).

<u>Step Two: Inoculation and incubation of the MGIT tubes</u>

1. For each specimen, label a MGIT tube with the patient number (or screening number if pre-enrolment) and the laboratory accession number label. Record the MGIT tube number in the approved laboratory source documentation. If working with a LIMS system, add an accession barcode label to each tube.
2. Add 0.8 ml of the supplements mixed above to each MGIT tube using a sterile pipette. Be careful to avoid contamination. The mixed supplements should be added to the MGIT medium prior to inoculation of specimen in MGIT tube. Do not add the mixed supplements after the inoculation of specimen.
3. Add 0.5 ml of a well-mixed, decontaminated and concentrated sputum specimen (SOP 3) to the appropriately labelled MGIT tube. Immediately recap the MGIT tube tightly and mix well by inversion several times. Wipe the MGIT tube with a paper towel soaked in disinfectant before removing it from the BSC. Use a separate graduated Pasteur pipette or micropipette for each specimen. Dispose of waste pipette tips into the discard bucket containing mycobactericidal disinfectant.
4. Store the remaining sputum sediment at 4°C for 10 days, until confirmed the MGIT tube is not contaminated.
5. The negative control from each batch of processed sputum samples should also be inoculated into a MGIT tube (labelled with 'negative', the date and batch number), and details completed on the local source document.
6. Enter the tubes in the machine following procedures provided by the manufacturer (always scan the MGIT barcode first and assign station through the <Tube entry> function).

NOTES:

- Do not place tubes without the instrument assigning a station.
- Do not remove tubes unless they are positive or out-of-protocol negatives (negative at 42 days)
- Do not re-assign tubes to a new station

<u>Step Three: Removal and work-up of positive MGIT tubes</u>

NOTE: Only discard positive MGIT tubes after autoclavation

<u>Equipment and Reagents</u>

- BSC
- Discard bucket containing mycobactericidal disinfectant (specified in local Health and Safety documentation)
- Blood agar (BA) plate
- 10 µl loop
- Plastic tube for centrifugation of sample
- Glass microscope slide
- Sterile Pasteur pipette and aerosol resistant tips.
- LJ slope
- 37°C incubator
- Centrifuge/microfuge

1. Remove the MGIT tubes from the machine according to manufacturer's instructions.
   NOTE: A positive tube can be re-entered in the machine for further incubation, but within 5 hours of removal. If the tube is returned via the 'tube entry' operation (see manufacturer's instructions), positive routines are reset, the start of incubation is retained, and monitoring of the tube resumes.
2. For positive MGIT tubes, print out the 'Unloaded Positives Report' which records the date the tube flagged positive and the number of days and hours taken to reach positivity (TTP).
3. Record the laboratory accession numbers of all unloaded tubes next to their corresponding results on the print outs. The print outs must be **signed and dated** by the staff member unloading the tubes. Result sheets must be kept in an unloaded positives folder or with the corresponding patient's worksheets/LRFs.
4. For positive tubes, the following must be performed:
   4.1. Blood Agar Culture (BAC) on all positive MGIT tubes:
      a. Label one BA plate with the laboratory accession number and the patient number.
      b. Vortex the MGIT tube well.
      c. Inoculate the BA plate with liquid from the MGIT tube using a 10 µL disposable loop, and incubate in a 37°C incubator for 48 hours.
      d. In the meantime, the MGIT tube must also be re-incubated. Return the tube to the MGIT machine or place it in an incubator at 37°C.
      e. Check for growth or contamination on the BA plate daily.
   4.2. Perform ZN stain microscopy (SOP 4) on all positive MGIT tubes even if confirmed to be contaminated.
      a. Using a Pasteur pipette or micropipette and ART, remove 50 µL of the MGIT culture and place onto a slide.
      b. Heat fix, stain and examine the slide for the presence of AFB per SOP 4.
      c. Although it is not necessary to semi-quantify the number of AFBs at this stage, describe the AFB identified in the smear (i.e. typical or atypical morphology and whether cording is seen).
5. Once the results of the ZN and BAC become available, refer to the algorithm shown on Figure 7.

Figure 7: Algorithm for Interpretation of Positive MGIT Cultures

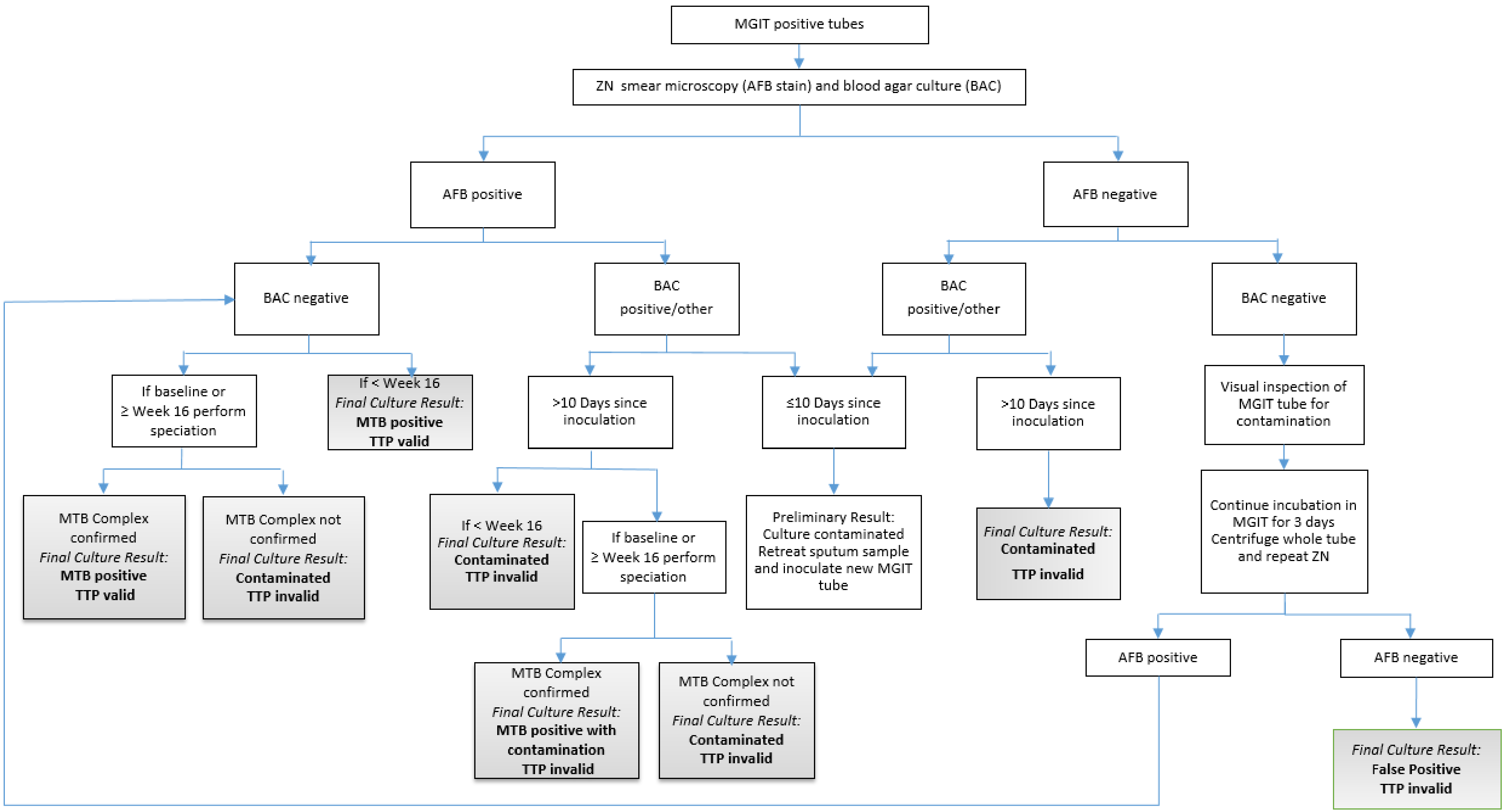

Step Four: Interpretation of results

BAC		AFB (ZN smear)		Result		MGIT TTP
Negative	and	Present (positive)	this is	True Positive	and means	Valid

1. If the sample is from baseline or from Week 16 or beyond, perform speciation – depending upon the selected test – see SOPs 5, 6 or 9.
2. If the MPT64 Ag Test is going to be performed, wait for 48-72 hours (from the time it signalled positive) before removing the MGIT tube. This additional incubation time is recommended to increase the test sensitivity.

BAC		AFB (ZN smear)		Result		MGIT TTP
Positive or Other*	and	Present or Absent	this is	Contaminated	and means	Invalid

* Situations where BAC conclusion should be marked as Other:

a. BAC not done (e.g. test not set up, discarded before reading, result missing).
b. BAC is negative, but MGIT tube visibly contaminated with fungal or other microbial growth.
c. BAC is negative, but evidence of fungal hyphae seen on ZN smear.

1. If the sample is confirmed as **contaminated within the first 10 days of inoculation**, the remaining sputum sediment (from the original decontamination) stored at 4°C is **to be retreated** per SOP 3 and a new MGIT culture to be inoculated.
2. If the contamination is confirmed after 10 days, the original sample will not be retreated and the overall result will be recorded as contaminated.
3. If the culture is contaminated (BAC positive or other) but contains AFB (ZN positive) AND corresponds to a sample from baseline or from week 16 or beyond, perform speciation.

BAC		AFB (ZN smear)		Result		MGIT TTP
Negative	and	Absent	this is	Possible False Positive	and means	Confirm it is a true false positive culture by checking if there are no AFBs present.

Confirm it is a true false positive by performing the following:

1. Examine the MGIT tube – record if is it turbid (in particular, if it shows a 'typical turbidity', i.e. breadcrumbs at the bottom of the tube) or showing any evidence of microbial growth (MTB or any contaminant).
    - A clear tube may be a true false positive resulting from altered reagent pH.
2. Repeat the ZN smear a second time to double check for AFBs.
3. If AFBs are not detected in the second smear, re-incubate the MGIT tube for at least 3 days (in MGIT machine or incubator) to allow further growth of any MTB present. After 3 days of additional incubation, repeat the ZN smear again.
4. This time, prior to performing the ZN smear, centrifuge the MGIT tube as follows:
    - If the centrifuge cannot accommodate MGIT tubes:
        - Inside the BSC, remove 1 ml of well mixed fluid from MGIT tube with a sterile Pasteur pipette into an Eppendorf tube.
        - Spin the sample using a microfuge.
        - Remove most of the supernatant and re-suspend pellet in remaining fluid (about 250 μL).
        - Label a slide and prepare a smear with the resuspended pellet.
        - OR
    - If the centrifuge can accommodate MGIT tubes:
        - Spin MGIT tubes without decanting at 3,000 xg for 15 min.
        - Tip off all but 2 mL of the supernatant into a discard bucket containing appropriate disinfectant.
        - Resuspend the pellet in the remaining supernatant.
        - Label a slide and prepare a smear with the re-suspended pellet.

Step Five: Storage/shipment for further investigations

Two samples must be stored for each isolate, one in 50% glycerol at -70°C to -80°C, and the other on a LJ slope. For the preparation of cultures for storage/shipment, follow SOP 2. A record must be kept of all isolates in storage.

In addition, for select visits, additional LJ subcultures are to be made for shipping to the Central Laboratories – see SOP 2 for details.

Step Six: Removal and work-up of negative MGIT tubes

The threshold for tubes to be declared as negative in BACTEC MGIT 960 is 42 days. Any tube that has not flagged positive prior to or at day 42 should be reported as a negative result. If a sample flags positive after 42 days, this must also be reported as negative.

1. Remove the MGIT negative tubes from the machine according to manufacturer's instructions.
2. Visually inspect all negative tubes. Any tube that appears to have growth must be processed as follows:
   2.1. Perform ZN and BAC as described above for positive tubes.
   2.2. Contact TB Alliance for instructions on how to report the results, if either AFB or another organism is detected.
3. Print the 'Unloaded Negatives Report', record the accession numbers of all unloaded tubes next to the results on the print outs. The print outs must be **signed and dated** by the staff member unloading the tubes and must be kept either in an unloaded negatives folder or with the patient's worksheets.
4. Autoclave negative tubes prior to discarding.

6.7.4. Quality Control

New media and supplements

A MTB reference strain (H37Rv/H37Ra) is tested to ensure that the medium supports growth of mycobacteria

Procedure
1. Prepare a fresh culture of MTB (H37Rv/H37Ra)
2. Adjust the turbidity to 0.5 McFarland.
3. Dilute the 0.5 McFarland suspension as follows to obtain a 1:500 dilution:
   - Add 1 ml of the suspension to 4 mL of sterile saline – Dilution 1 (1:5).
   - Add 1 ml of Dilution 1 to 4 mL of sterile saline – Dilution 2 (1:50).
   - Add 1 ml of Dilution 2 to 4 mL of sterile saline – Dilution 3 (1:500).
4. Prepare the MGIT tube following the normal MGIT culture procedure.
5. Label each tube with the date, expiry date and mark as 'Control'.
6. Inoculate one MGIT tube from each new batch number with 0.5 mL Dilution 3.
7. The control tube should become positive within 6-10 days. If the QC tubes do not give the expected results do not use the remaining tubes of the batch. Repeat the QC test and if fails, contact the manufacturer for troubleshooting.

Results of this QC are to be reported using NiX-TB Mycobacteriology Quality Manual Attachment Eiii.

MGIT maintenance must be performed daily and monthly, preferably before unloading or loading of tubes, and **recorded on Quality Manual Attachment J.**

MGIT failure/breakdown lasting more than 24 hours:

Follow manufacturer's instructions. Briefly, if power is lost for more than 24 hours remove all tubes and place in a 37°C incubator. Read manually using an ultraviolet (UV) trans-illuminator (365 nm) or a Wood's lamp with a long-wave bulb or black-light (wear eye protection). Once the tubes are removed they must be read off-line daily throughout the 6 week protocol. Tubes must not be returned to the MGIT instrument. Prepare smears and stain any positive tubes for confirmation of AFBs. Before disposing of any negative tubes check for turbidity and perform Z-N microscopy to ensure tubes are negative.

If there is no access to a UV light, take a small sample using aseptic technique from the MGIT tube daily, make a smear and perform Z-N staining. Calculate the TTP from the date the tubes were inoculated to the date the tubes were confirmed positive/negative manually.

6.7.5. Documentation

The suggested LRF1 (for Screening samples) and LRF2 (Treatment and Follow-up samples) capture the information for completing the eCRF fields (highlighted) related to MGIT culture and follow-up tests. Study Laboratories may use their own forms or LIMS as long as these pieces of information are included and they are approved by the Sponsor.

NOTE: TTD/TTP should only be reported for cultures flagged positive, which are not found to be contaminated (BAC negative), and AFB positive (ZN positive). In addition, if the visit requires speciation (SOP 9) wait for the result of this test – in this case, only report the TTD/TTP the culture is found positive for MTBC.

6.8. SOP 8: MPT64 Antigen Test

6.8.1. Purpose

Used by the **Study Laboratories** to rapidly identify the *M. tuberculosis* complex (MTBC) in acid-fast bacilli (AFB) positive MGIT cultures.

6.8.2. Principle

The CE marked BD TBc ID, SD Bioline, or Capilia TB-Neo are all commercial variations of the same test, MPT64 antigen assay (or MPT64Ag test), and all have been shown to be highly sensitive and specific in a number of studies conducted in clinical settings. The MPT64Ag test is a lateral flow, immunochromatographic assay that detects MPT64 antigen, a mycobacterial protein that is specifically secreted from MTBC while growing in culture. When a bacterial suspension is added to the test device, the MPT64 antigen binds to anti-MPT64 antibodies conjugated to colloidal gold particles present on the test strip, forming an antigen-antibody complex. This antigen-antibody complex then migrates across the test strip to the reaction area, where it is captured by a second specific MPT64 antibody fixed to the membrane. If MPT64 antigen is present in the sample, a colour reaction is produced by the labelled colloidal gold particles and is visualized as a pink (or purple) to red line. An internal positive control is included to validate proper test performance. The test will detect the following species of MTBC: *M. tuberculosis*, *M. bovis*, *M. africanum*, and *M. microti*.

In this study, the MPT64 Ag test will be used to differentiate MTBC from mycobacteria other than *M. tuberculosis* (MOTT) in AFB positive MGIT cultures **from Baseline (Day 1 or screening to week 4 if Day 1 is contaminated or negative) or at/after Week 16 only**. Also note that **only one baseline sample requires speciation**.

6.8.3. Procedure

To be performed inside a BSC using the appropriate biosafety procedures and PPE.

<u>**Equipment and Reagents**</u>

- BSC
- Appropriate PPE
- Mycobactericidal disinfectant
- Benchguard (or alternative work surface protection)
- Cotton wool or paper towels
- Biohazard bags
- Screw top bottle for waste
- Extraction buffer (commercially available or in-house prepared) – (KH_2PO_4, NaCl, Tween 80)
- MPT64Ag test device
- 200 μL micropipette and sterile ARTs
- 10 μL sterile disposable loops
- Sterile 2 mL cryovials

<u>**Process**</u>

Ideally, test AFB smear-positive MGIT tubes within 2-3 days of instrument positivity.

1. If devices are refrigerated, bring to room temperature in the foil pouch prior to testing.
2. Remove the test device from the foil pouch and place on a dry, flat surface.
3. Label the device with the patient or screening number and laboratory accession number.
4. Vortex the tightly capped MGIT tube for 30 seconds to ensure the suspension is well- mixed.
5. Add 100 μl of liquid culture to the sample well.

6. Start timer for 15 minutes. As the test develops, a purple colour will migrate across the result window in the centre of the test device.
7. Interpret the test result 15 minutes after sample application. **Do not read the test after 60 minutes.**

Result Interpretation

The tables below show the proper interpretation of each type of kit. Record the result on the local lab worksheet.

Table 8: Interpretation of the MPT64 Antigen Assays

**SD Bioline TB Ag MPT64 (Configuration of cartridge is similar in the Tauns Capilia TB-Neo kit.)**	
1. **Negative**: only the Control Band ‘C’ appears (purple to red line) in the result window.	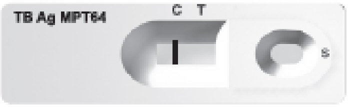
2. **Positive:** appearance of two colour bands (‘T’ band and ‘C’ band) in the result window, no matter which band appears first.	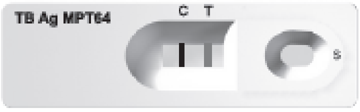
3. **Invalid result**: if the control band is not visible in the result window after performing the test, the result is considered invalid.	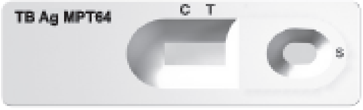 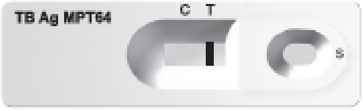
**BD TBcID**	
1. **Negative**: A pink/purple to red line forms only on the reading area labelled Control ‘**C**’.	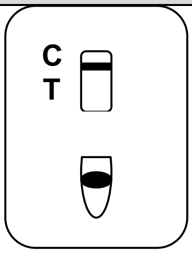
2. **Positive**: Pink/purple to red lines form on the reading areas labelled Test ‘**T**’ and ‘**C**’ of the device.	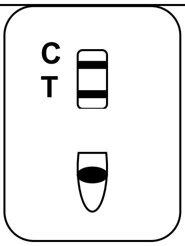
3. **Invalid result**: If no line is observed on the reading area labelled [**C**], technical errors or product damage has occurred. In this case, the test should be considered invalid and repeated using a new device.	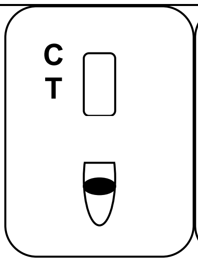 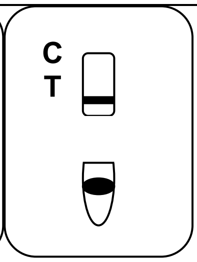

NOTES:

- If the MPT64Ag test is negative, but MTB is highly suspected – AFB microscopic morphology and isolate growth pattern in MGIT are consistent with MTB – use a PCR-based test (GeneXpert; or Hain MTBDR*plus*, MTBDR*sl,* MTBC or Mycobacterium CM) to detect bacteria that may have a mutation in the MPT64 gene.
- If the rapid ID test is invalid: investigate causes for the invalid result and check the expiry date of kit, decontaminating a heavily contaminated culture, etc.

6.8.4. Quality Control

Frequency:

- Each new lot or shipment of kits.
- Weekly, or with each batch of patient tests, if testing is performed less frequently.

Controls:

- Internal reagent control in device.
- Positive control: Culture of MTB reference strain (H37Rv or H37Ra) in MGIT broth.
- Negative control: Culture of a MOTT strain (e.g. a well characterized strain of *M. avium* complex) in MGIT broth or broth from an uninoculated MGIT tube.

Acceptable results: Correct results as expected for all controls

- Internal control line is visible.
- MTB must result in a positive test.
- MOTT strain or uninoculated broth must result in a negative test.

Corrective actions: If any control result is unacceptable, do not report patient tests.

- Repeat test with new controls; if acceptable, repeat patient tests.
- If repeat results still unacceptable, notify supervisor immediately and investigate potential causes for failure.
- After investigation is complete and QC is acceptable, repeat patient tests and report results.

Results of this QC are to be recorded on the Nix-TB Quality Manual Attachment Evii.

6.8.5. Documentation

The suggested LRF3 captures the information for completing the eCRF fields (highlighted) related to speciation. Study Laboratories may use their own forms or LIMS as long as these pieces of information are included and they are approved by the Sponsor.

6.9. SOP 9: DNA Extraction

6.9.1. Purpose

Used by the **UCL Mycobacteriology Laboratory** to extract DNA from the isolates from the baseline and first positive culture at or after the end of treatment that is indicative of treatment failure or relapse/reinfection [confirmed positive cultures at or after week 26 (6 month treatment arm)/week 36 (9 month treatment arm)].

The method described here is designed to yield microgram quantities of high molecular weight DNA suitable for genotyping.

6.9.2. Principle

Although DNA can be extracted from *M. tuberculosis* (MTB) bacilli by a variety of methods, with a range of complexity, the method described here is designed to yield high quality large fragment DNA from a colony pick. Using a combination of enzymatic digestion and organic partition, colonies picked from the LJ slope yield nanogram to microgram quantities of DNA. Following heat killing of the colonies, bacteria are digested first with lysozyme to breakdown the cell wall then with proteinase K, which has further action on the cell wall but, importantly, digests any enzymes released by the lysed bacterium, including DNases. MTB is lipid rich and so two rounds of detergent are used, first SDS and then CTAB. These detergents have action on molecules with different charges thus affecting different cell wall components. EDTA is used to chelate Mg^{+2} and Ca^{+2} ions, inhibiting DNase activity, similarly high salt concentrations inhibit DNA-enzyme binding. Finally, organic solvents are used to partition the DNA to an aqueous phase, leaving lipids and proteins in the organic phase. The aqueous phase is then concentrated using isopropanol, this concentrates the DNA and removes excess salt. Isopropanol is used in preference to ethanol as a lower volume for precipitation can be used (1:1 rather than 2:1).

6.9.3. Procedure

6.9.3.1. Isolation of genomic DNA from *M. tuberculosis*

<u>Equipment/Reagents</u>

- Biological Safety Cabinet
- Waterbath or heating blocks (80-95°C, 60-65°C and 37°C)
- 10 µl loops
- 1.5 ml screw-capped Eppendorf tubes with rubber ‘O’ ring seal
- 1000 µl pipette and aerosol resistant tips
- 200 µl pipette and aerosol resistant tips
- Tris-EDTA (TE) buffer
- Microfuge
- 10 mg/ml lysozyme
- 10% Sodium dodecyl sulphate (SDS)
- 10 mg/ml Proteinase K
- 5M NaCl
- Cetyl trimethylammonium bromide (CTAB)
- Chloroform
- Isoamylalcohol
- Sterile DNAase-free 1.5 ml eppendorf tubes
- 70% ethanol
- Isopropanol
- -20°C freezer
- 4°C refrigerator

Preparation of extraction reagents

Lysozyme solution: 10 mg/ml.
Store in small aliquots at -20°C. Use a new aliquot each time, do not re-freeze. Any remaining reagent should be discarded.

10% SDS
Add distilled water to 10 g of SDS to make up 100 ml of total solution. . Dissolve by heating at 65 °C for 20 min. Do not autoclave. Store at room temperature for no longer than 1 month

Proteinase K: 10 mg/ml.
Store in small aliquots at -20 °C. Use a new aliquot each time, do not re-freeze. Any remaining reagent should be discarded. Unless stated otherwise on manufacturing guidelines.

5M NaCl
29.2 g NaCl/100 ml distilled water. Heat to 65°C and mix until dissolved (this may take hours). Autoclave at 121°C for 20 minutes. Store at room temperature for no longer than 1 year.

CTAB/NaCl (10% CTAB in 0.7 M NaCl)
Dissolve 4.1 g NaCl in 80 ml distilled water. While stirring, add 10 g CTAB. If necessary, heat solution to 65 °C Adjust the volume to 100 ml with distilled water and autoclave at 121°C for 20 minutes. Store at room temperature for no longer than 6 months.

Chloroform/isoamylalcohol (24:1)
Mix 1 part of isoamylalcohol with 24 parts of chloroform. Mix thoroughly by shaking vigorously for 5 seconds. Store in cool, dark, ventilated place, use within 6 months or by expiry date indicated.

70% Ethanol
70 ml 100% ethanol in 30 ml distilled water, store at -20°C.

Process

Vortexing is not recommended at any stage of the extraction as this causes DNA shearing. All steps, until after the heat killing of MTB culture (step 6) and briefly centrifuging the tubes (step 7) must be carried out in an appropriate BSC inside a BSL/CL-3 Laboratory.

1. Fill the waterbath with tap water and set for 80°C. If a waterbath is unavailable use 95°C heating block
2. Label sufficient 1.5mL screw capped tubes containing ‘O’ rings with patient number and laboratory accession number.
3. Aliquot 400µL volumes of 1x Tris-EDTA (TE) buffer into the tubes using aerosol resistant tips.
4. From LJ slopes with good growth, take all organisms using a 10 µl loop and emulsify them in the appropriate tubes containing the TE buffer taking care not to create splashes or aerosols.
5. Pulse down the tubes in the microfuge using the aerosol-containing rotor for 5 seconds to ensure that all organisms are at the bottom of the tube, and unload the rotor in the BSC.
6. Place the tubes in a suitable rack and heat-kill in the waterbath/heating block at 80-95°C for 30 minutes or as specified by the local health and safety guidelines.
7. Pulse down the tubes, as above. Tubes can now be removed from the Containment Level 3 Laboratory, unless otherwise stated in local Health and Safety documentation
8. Add 50µL 10mg/mL lysozyme and mix gently with the pipette. Incubate at 37°C in the waterbath, incubator or heating block overnight (if overnight is not possible at least one hour is required).
9. Set the waterbath to 65°C or switch on 60°C heating block. Pre-warm the CTAB/NaCL to 60-65°C.
10. Add 70µL 10% SDS and 5µL 10mg/ml proteinase K. Mix gently with the pipette and incubate at 60-65°C for 10 minutes.
11. Add 100µL 5M NaCl to each tube.
12. Add 100µL CTAB/NaCl (pre-warmed to 60-65°C). Mix gently with the pipette and incubate at 60-65°C for 10 minutes.

13. Switch off and empty the waterbath.
14. Add 750µL chloroform/isoamylalcohol (24:1 v/v). Mix by inversion.
15. Place into correctly labelled 1.5ml universal tubes. Microfuge at 10,000 g for 5 minutes. Ensure the opening of the lids face inwards to develop a clear pellet.
16. Label sterile DNAase free 1.5mL Eppendorf tubes and aliquot 450µL volumes of ice-cold isopropanol to each.
17. Transfer the aqueous supernatants into the Eppendorf tubes containing isopropanol. Take care not to disturb the interface. Mix by gentle inversion.
18. Place at -20°C for 30 minutes. Also place a glass container of 70% ethanol at –20°C.
19. After at least 30 minutes, microfuge at 10 000 g for 15 minutes at room temperature.
20. Remove the supernatants and wash the pellets with 1mL ice-cold 70% ethanol. Invert gently.
21. Microfuge at 10 000 g for 5 minutes at room temperature. Remove and discard as much of the ethanol as possible. Lay or tilt the tubes with open lids to allow the pellets to air-dry (at least 15 min).
22. Rehydrate the pellets in approx. 100µL (depending on pellet size) molecular grade water (or TE buffer with a maximum concentration of 0.1mM EDTA) overnight at 4°C (or 1 hour at 65 °C).

6.9.3.2. Estimation of DNA concentration

DNA concentration in each of the samples will be estimated by both of the methods detailed below to ensure the quantity and quality is sufficient for WGS analysis.

Estimation of DNA Concentration Using Nanodrop

Equipment/Reagents

- NanoDrop Spectrophotometer
- BM compatible PC (see NanoDrop user's manual for computer requirements, software installation and set-up)
- 2 µl Pippette with appropriate tips
- Soft laboratory wipe/tissue
- De-ionised water
- Tris-EDTA (TE)

Process

1. Install software onto your computer and attach USB cable between the NanoDrop and PC (as described in the User's Manual).
2. To measure nucleic acid concentration and quality select the 'Nucleic Acid' application module.
3. Follow instructions by loading 1 µl distilled or PCR grade water sample to initialize the instrument. Wipe pedestals clean (using lint-free tissue).
4. Select sample type 'DNA-50' for double stranded DNA (default). Enter sample ID if appropriate.
5. Always perform a blank run before testing DNA samples (this will ensure the instrument is working properly and the pedestal is clean).
6. With the sampling arm open, pipette 1 µl molecular grade water (or TE buffer with a maximum concentration of 0.1mM EDTA if this was used to rehydrate the DNA) onto the lower measurement pedestal.
7. Close the sampling arm and click on the 'Blank' button.
8. When the measurement is complete, wipe the blanking buffer from both pedestals using a laboratory wipe (lint-free tissue).
9. Analyse an aliquot of the blanking solution as though it were a sample. This is done by using the 'Measure' button (F1). The result should be a spectrum with relatively flat baseline. Wipe the blank from both the upper and lower pedestal surfaces and repeat the process until the spectrum is flat.
10. Clean the pedestals by wiping with a laboratory cloth.
11. Pipette 1 µl of sample DNA onto the lower measurement pedestal (if you are unsure about your sample or your pipettor accuracy, a 1.5 – 2 µl sample is recommended to ensure the liquid sample column is formed and the light path is completely covered by sample).
12. Make sure the sample type is DNA-50 and enter any sample ID details.

13. Click 'Measure'.
14. Repeat for any other samples, wiping the pedestals in between samples.
15. The results should save automatically and at the end of the set of samples click 'Show Report'. This can then be printed and saved. Alternatively, record the DNA concentration and the 260/280 ratio in the table provided.
16. Clean after use by wiping with 70% ethanol followed by distilled water.

Estimation of DNA Concentration Using the Qubit Fluorometer

The Qubit dsDNA Assay Kits are designed for accurate DNA quantification and are highly selective for double-stranded DNA (dsDNA) over RNA.

Equipment/Reagents

- Qubit® dsDNA Assay BR Kit. This is the broad range (BR) kit with quantitation range of 2-1000ng. Note: *The high sensitivity (HR) kit (quantitation range of 0.2-100ng) can also be used if required.*
    - Qubit® dsDNA BR Reagent (Component A)
    - Qubit® dsDNA BR Buffer (Component B)
    - Qubit® dsDNA BR Standard #1 (Component C)
    - Qubit® dsDNA BR Standard #2 (Component D)
- Disposable plastic container for mixing the Qubit® working solution
- Thin-wall, clear, 0.5ml PCR tubes. Acceptable tubes include Qubit® assay tubes (Life Technologies Cat No. Q32856) or Axygen® PCR-05-C tubes (VWR Cat No. 10011-830).
- Qubit Fluorometer (2.0)

Storage of reagents: The Qubit® dsDNA BR Reagent and Buffer must be stored at room temperature (22-28°C) and the Qubit® DNA standards must be stored at 4°C. The Qubit Reagent must be protected from light. When stored as directed kits are stable for 6 months.

Process

1. Set up the required number of 0.5ml tubes for the samples and standards (2 tubes) and label the lids with the sample Laboratory Accession Number (LAN).
2. Prepare Qubit® working solution by diluting the Qubit® dsDNA BR Reagent 1:200 in the Qubit® dsDNA BR Buffer in a clean plastic tube. The final volume in each tube is 200µl l, so prepare enough working solution for all the samples and the two standards. For example, for 8 samples and 2 standards make 2ml of working solution by diluting 10 µl l of reagent into 1990µl of buffer.
3. Add 190 µl of Qubit® working solution to each of the standard tubes, then add 10ul of each Qubit® standard to the appropriate tube and mix by vortexing for 2-3 seconds. Be careful not to create bubbles.
4. Add Qubit® working solution to individual sample tubes so that the final volume including the sample is 200 µl. The volume of the sample can be between 1-20 µl, so the volume of working solution can be adjusted accordingly.
5. Add each sample to the appropriate assay tubes and mix by vortexing for 2-3 seconds.
6. Allow all tubes to incubate at room temperature for 2 minutes - the samples are now ready to be read on the fluorometer.
7. On the home screen of the Qubit® Fluorometer, press **DNA** and then select **dsDNA Broad Range** as the assay type. The standards screen is displayed.
8. Press **Yes** to read the standards. Insert the tube containing Standard #1 into the sample chamber, close the lid and press **Read**. When the reading is complete (~3 seconds) remove the standard. Insert the tube containing Standard #2 into the sample chamber, close the lid and press **Read**. When the reading is complete remove the standard. When the calibration is complete the instrument displays the Sample screen.
9. Insert a sample tube into the sample chamber, close the lid and press Read. When the reading is complete (approx. 3 seconds) remove the tube. The instrument displays the results on the screen. The first value displayed is the concentration in the assay tube.
10. To find out the concentration in the original sample, press Calculate Stock Conc. The **Dilution Calculator Screen** is displayed. Select the volume of the sample that you added to the assay tube, once selected the Qubit® Fluorometer

calculates the original sample concentration using the volume and the measured assay concentration. Record this concentration in the table provided in the NiX-TB Quality Manual Attachment M.

11. Press Read Next Sample, and repeat steps 9 and 10 for all remaining samples.

See SOP 13 for further details on sample shipment and WGS analysis.

6.10. SOP 10: Drug Susceptibility Testing (DST) by Mycobacteria Growth Indicator Tube (MGIT)

6.10.1. Purpose

Used by the **UCL Mycobacteriology Laboratory** for Drug Susceptibility Testing (DST) for streptomycin (S), isoniazid (I), rifampicin (R), ethambutol (E), moxifloxacin (M), kanamycin (K) and pyrazinamide (Z).

DST to S, I, R, E, M and K is performed using the Bactec MGIT 960 SIRE kit. Whereas DST to Z is performed using the Bactec MGIT 960 PZA kit. Moxifloxacin will be tested at two concentrations, 2µg/ml and 0.5µg/ml, although only the result from the testing at 0.5µg/ml will be entered into the eCRF.

Susceptibility testing will be performed on pre-treatment isolates from the baseline (Day 1 or Screening to week 4, if Day 1 cultures are negative or contaminated) and the isolates from patients suspected of failure or relapse after treatment (a positive culture at or after week 24 [6 month treatment arm)/week 36 (9 month treatment arm) and any new positive culture thereafter] in order to identify the presence of resistance to any of the study drugs.

NOTE: Prior to any drug susceptibility testing (DST), a HAIN MTBC assay (SOP 5) is performed at the UCL Central Mycobacteriology Laboratory to confirm the presence of MTB complex (MTC). If the HAIN test does not confirm MTC, the local laboratory will be contacted and a new culture will be sent.

6.10.2. Principle

Susceptibility testing in the MGIT 960 system is based on the same principles as isolation from sputum (detection of growth). DST is performed using an AST (antibiotic susceptibility testing) set, which consists of a Growth Control tube and one tube for each drug, as well as a bar-coded tube carrier that holds the set. A known concentration of drug is added to a MGIT tube, along with the specimen, and growth is compared with a drug-free control of the same specimen. If the drug is active against the mycobacterial isolate (isolate susceptible), growth will be inhibited and fluorescence will be suppressed in the drug-containing tube; meanwhile, the drug-free control will grow and show increasing fluorescence. If the isolate is resistant, growth and its corresponding increase in fluorescence will be evident in both the drug-containing and the drug-free tube. The growth rate of the test isolate is compared in the presence and in the absence of antibiotics. An isolate is determined resistant if 1% or more of the test population grows in the presence of the critical concentration of the drug.

6.10.3. Procedure

Equipment/Reagents

- Biological Safety Cabinet
- Discard bucket containing appropriate liquid disinfectant (specified in local Health and Safety documentation
- 7ml MGIT tubes
- BD SIRE MGIT kit reagents
- BD Moxifloxacin HCl lyophilised powder
- BD Kanamycin sulphate lyophilised powder
- BD BACTEC MGIT supplement (for SIRE and PZA drug kits)
- McFarland standards
- p1000 and p200 pipettes and aerosol resistant tips
- Sterile saline
- Blood agar plates
- Glass slide

For DST from LJ slopes:

- Middlebrook 7H9 broth

- Capped sterile tube containing glass beads
- Vortex

6.10.3.1. Preparation of drug stocks for susceptibility testing

Drug stocks and preparation of MGIT tubes can be carried out outside of the CL3/BSL3 laboratory.

Table 9: Preparation of DST Drug Stock Solutions

TASK	INSTRUCTIONS
NOTE **– the following may be reconstituted with different volumes. Failure to use the appropriate volume of sterile distilled/deionised water for reconstitution of the drugs will invalidate these tests**	
Prepare BACTEC^{TM} MGIT^{TM} 960 SIRE Drug Kit	• Reconstitute each BACTEC^{TM} MGIT^{TM} 960 SIRE Kit Streptomycin lyophilised drug vial with **4 ml** of sterile distilled/deionised water to make a stock solution of 83µg/ml.<br>• Reconstitute each BACTEC^{TM} MGIT^{TM} 960 SIRE Kit Isoniazid lyophilised drug vial with **4 ml** of sterile distilled/deionised water to make a stock solution of 8.3µg/ml.<br>• Reconstitute each BACTEC^{TM} MGIT^{TM} 960 SIRE Kit Rifampicin lyophilised drug vial with **4 ml** of sterile distilled/deionised water to make a stock solution of 83µg/ml.<br>• Reconstitute each BACTEC^{TM} MGIT^{TM} 960 SIRE Kit Ethambutol lyophilised drug vial with **4 ml** of sterile distilled/deionised water to make a stock solution of 415µg/ml.
Prepare BACTEC MGIT 960 Pyrazinamide Kit	• Reconstitute each BACTEC^{TM} MGIT^{TM} 960 PZA drug vial with **2.5 ml** of sterile distilled/deionised water to make a stock solution of 83µg/ml.
Prepare BD Moxifloxacin HCl	• Reconstitute each BD Moxifloxacin Hydrochloride drug vial with **3 ml** of sterile distilled/deionised water to make a stock solution of 166µg/ml. This will be used for the Moxi-2µg/ml set, for the Moxi-0.5 µg/ml this will be diluted 1:4 in sterile distilled/deionised water.
Prepare BD Kanamycin Sulphate	• Reconstitute the BACTEC^{TM} MGIT^{TM} 960 Kanamycin lyophilised drug vial with **4 ml** of sterile distilled/deionised water to make a stock solution of 207.5µg/ml.
**NOTE:** On receipt of SIRE and PZA kit reagents and moxifloxacin and kanamycin powder, store the lyophilised drug vials at 2 - 8°C. Once reconstituted, the antibiotic solutions should be aliquoted out and may subsequently be frozen and stored at -20°C or colder for up to six months, but must not exceed the original expiry date of the kit/vial. Once thawed, use immediately. Discard any unused portions.	

6.10.3.2. Preparation of MGIT tubes for DST testing

ADD Epi centre info here – for SIRE, K, M.
For the MGIT DST, the drugs can be set up as 4 individual sets each with a growth control (SIRE, M, K, and Z) using the 5- and 2- tube carrier sets as outlined below. MGIT DSTs may also be performed via the EpiCenter software (a user interface for the MGIT instrument) including the TB eXiST (TB eXtended Susceptibility Testing) module, as is done for the MIC testing (see SOP 11). Using TBeXiST it is possible to extend susceptibility testing to all MTB isolates against primary drugs. For example, some MDR-TB and XDR strains are slow growing and therefore may not reach the completed threshold within the 13 days required for the growth control (GU>400) for automatic DST interpretation in the MGIT using the carrier sets, giving an x200 readout. TBeXiST can be used for SIRE, K, M-2 and M-0.5 as one set with a single growth control, and if required can be combined with the MIC testing so that these DSTs and MICs are performed in a single experiment with one growth control (up to 50 drug tubes can be set up with a single growth control). For pyrazinamide, this requires different tubes and supplement, so needs to be set up as an independent experiment with a separate growth control. This should be done routinely using the carrier sets, but it is acceptable to use the TBeXiST if the standard test fails due to slow growth outside the acceptable window (>21 days). For information about labelling and loading MGIT tubes into MGIT using the EpiCenter and TBeXiST software, refer to SOP11, and associated user guides – 'Registering TBeXiST MGIT

tube for MIC-DST Users Guide' and 'Interpretation and Reporting of MIC-DST in TBeXiST Users Guide'. The drug concentrations selected must be those detailed in Table 10.

Preparation of the drug sets using AST carrier sets:

For preparation of SIRE set:

1. Label five 7 mL MGIT tubes for each test isolate with the appropriate laboratory accession label and the patient study number. In addition, label tubes with one of each of the following: GC (Growth Control), STR (streptomycin), INH (isoniazid), RIF (rifampicin), EMB (ethambutol).
2. Place the tubes in the correct sequence in the 5 tube AST set carrier (see BACTEC MGIT 960 User's Manual, AST Instructions).
3. Aseptically add 0.8 ml of BACTEC MGIT SIRE Supplement to each SIRE tube. It is important to use the supplement supplied with the kit.
4. Aseptically pipette 100 µl of 83 µg/mL MGIT STR solution to the appropriately labelled MGIT tube.
5. Aseptically pipette 100 µl of 8.3 µg/mL MGIT INH solution to the appropriately MGIT tube.
6. Aseptically pipette 100 µl of 83 µg/mL MGIT RIF solution to the appropriately MGIT tube.
7. Aseptically pipette 100 µl of 415 µg/mL MGIT EMB solution to the appropriately labelled MGIT tube.
8. It is important to add the correct drug to the corresponding tube. No antibiotics should be added to the MGIT GC tube.

For preparation of the moxifloxacin 2µg/ml set:

1. Label two 7mL MGIT tubes for each test isolate with the appropriate laboratory accession label and the patient study number. In addition, label tubes with one of each of the following: GC (Growth Control) and MOX-2
2. Place tubes in the correct sequence for the 2 tube AST set carrier (see BACTEC MGIT 960 User's manual, AST instructions)
3. For moxifloxacin set, the tubes and supplement from the BD SIRE set can be used. Aseptically add 0.8ml of BACTEC MGIT SIRE supplement to each tube.
4. Aseptically pipette 100µl of 166µg/ml MGIT MOX solution to the appropriately labelled MGIT tube.
5. No antibiotics should be added to the MGIT GC tube.

For preparation of the moxifloxacin 0.5µg/ml set:

1. Label two 7mL MGIT tubes for each test isolate with the appropriate laboratory accession label and the patient study number. In addition, label tubes with one of each of the following: GC (Growth Control) and MOX-0.5
2. Place tubes in the correct sequence for the 2 tube AST set carrier (see BACTEC MGIT 960 User's manual, AST instructions)
3. For moxifloxacin set, the tubes and supplement from the BD SIRE set can be used. Aseptically add 0.8ml of BACTEC MGIT SIRE supplement to each tube.
4. Dilute the 166µg/ml MGIT MOX solution 1:4 in sterile distilled/deionised water (e.g. 100µl drug stock solution and 300µl of water).
5. Aseptically pipette 100µl of the new drug solution to the appropriately labelled MGIT tube.
6. No antibiotics should be added to the MGIT GC tube.

For preparation of the kanamycin set:

1. Label two 7mL MGIT tubes for each test isolate with the appropriate laboratory accession label and the patient study number. In addition, label tubes with one of each of the following: GC (Growth Control) and K (kanamycin)
2. Place tubes in the correct sequence for the 2 tube AST set carrier (see BACTEC MGIT 960 User's manual, AST instructions)
3. For kanamycin set, the tubes and supplement from the BD SIRE set can be used. Aseptically add 0.8ml of BACTEC MGIT SIRE supplement to each tube.
4. Aseptically pipette 100µl of 207.5ug/ml MGIT K solution to the appropriately labelled MGIT tube.
5. No antibiotics should be added to the MGIT GC tube.

For preparation of the pyrazinamide set:

1. Label two 7mL PZA MGIT tubes for each test isolate with the appropriate laboratory accession label and the patient

study number. In addition, label tubes with one of each of the following: GC (Growth Control) and PZA (pyrazinamide)
2. Place tubes in the correct sequence for the 2 tube AST set carrier (see BACTEC MGIT 960 User's manual, AST instructions)
3. Aseptically add 0.8mL of BACTEC MGIT PZA supplement to each PZA tube. It is important to use PZA tubes and supplement as the pH of the medium is lower (pH 5.9)
4. Aseptically pipette 100uL of 8000µg/ml MGIT PZA solution to the appropriately labelled MGIT tube.
5. No antibiotics should be added to the MGIT GC tube.

Table 10: Working concentrations of DST Drugs

Drug	Concentration of drug after reconstitution	Volume added to MGIT tubes for test	Final concentration in MGIT tubes
MGIT STR	83µg/ml	100µl	**1.0µg/ml**
MGIT INH	8.3µg/ml	100µl	**0.1µg/ml**
MGIT RIF	83µg/ml	100µl	**1.0µg/ml**
MGIT EMB	415µg/ml	100µl	**5.0µg/ml**
MOX -2	166µg/ml	100µl	**2.0µg/ml**
MOX -0.5	41.5 µg/ml*	100µl	**0.5 µg/ml**
KAN	207.5µg/ml	100µl	**2.5µg/ml**
PZA	8000µg/ml	100µl	**100µg/ml**

*This concentration is achieved by a 1:4 dilution of the stock solution prepared for MOX-2

6.10.3.3. Using inoculum from positive MGIT – carried out in BSC in CL3/BSL3 laboratory

Once a MGIT tube has become positive it must be used for DSTs within the appropriate timeframe (1-5 days). The concentration of the inoculum is critical to the correct performance of susceptibility testing and the following instructions must be adhered to strictly.
On the day the MGIT flags positive (day 0), the culture should be identified as a PURE growth of *M. tuberculosis* and tube should be re-incubated for a minimum of one day (day 1). This can be in the MGIT machine or in a separate 37C incubator.

Day 1 and Day 2 – the growth in the tube can be used directly. Glass beads should be used to break up the clumps in the MGIT culture and obtain a uniform bacterial suspension. Pre-prepared sterile glass beads (minimum 4 beads, 5 mm diameter) in saline (3 ml) are used. Remove the saline by pipetting and pour all the beads into the MGIT tube. Mix well by vortexing to break up clumps as much as possible (between 2 and 10 minutes) and let any remaining clumps settle out for 30 minutes. Use the supernatant undiluted.

Days 3, 4 and 5 – the growth in the tube should be diluted before use. Vortex the MGIT culture with beads as described above and leave for 30 minutes for any remaining clumps to settle out. Dilute 1 ml of supernatant in 4 mL of sterile saline (1:5 dilution). Use this diluted culture for the DST drug tubes.

>5 Days – subculture into a new MGIT tube and wait for this to flag positive. Treat as above and use within 5 days to set up the DST.

NOTE: Cultures grown in liquid or solid media can be used to prepare a seed MGIT tube. When positive, the seed MGIT can then be used to prepare the inoculum as described above. From a liquid culture, a 1:100 dilution should be made of the broth, and 500µl added to the seed MGIT tube. For solid media, a loop of growth scraped from the slope or plate should be added to the seed MGIT tube. After incubation, the TTP of the seed MGIT tube must be 4 days or more for use as a DST inoculum. If the seed tube becomes positive in 4 days or less, a new seed tube should be prepared.

6.10.3.4. Using an inoculum from LJ slope – carried out in BSC in CL3/BSL3 laboratory

1. All preparations must be made from the pure cultures of MTB. The isolate must be confirmed, by appropriate identification techniques.
2. Add 4 mL of Middlebrook 7H9 Broth (or BBL MGIT broth) to a 16.5 x 128 mm sterile tube with cap containing 8 – 10 glass beads.
3. Scrape with a sterile loop as many colonies as possible from growth no more than 14 days old, trying not to remove any solid medium. Suspend the colonies in the Middlebrook 7H9 Broth.
4. Vortex the suspension for 2 – 3 min to break up the larger clumps. The suspension should exceed a 1.0 McFarland standard in turbidity.
5. Let the suspension sit for 20 min without disturbing.
6. Transfer the supernatant fluid to another 16.5 x 128 mm sterile tube with cap (avoid transferring any of the sediment) and let sit for another 15 min.
7. Transfer the supernatant fluid (it should be smooth, free of any clumps) to a third 16.5 x 128 mm sterile tube. NOTE: The organism suspension should be greater than a 0.5 McFarland standard at this step.
8. Adjust suspension to a 0.5 McFarland standard by a visual comparison with a 0.5 McFarland turbidity standard. Do not adjust below a 0.5 McFarland Standard.
9. Dilute 1 mL of the adjusted suspension in 4 mL of sterile saline (1:5 dilutions).

6.10.3.5. Growth Control tube preparation and inoculation – carried out in a BSC in CL3/BSL3 laboratory

For SIRE, MOX and KAN Growth Control Tubes:

1. Aseptically pipette 0.1 ml of the organism suspension (used to inoculate drug tubes) into a total of 10mL of sterile saline to prepare the 1:100 GC suspension (1% growth control).
2. Mix the GC suspension thoroughly.
3. Inoculate 0.5mL of the 1:100 GC suspension into the MGIT tubes labelled "GC", using a micropipettor and aerosol resistant tips. Dispose of pipette into discard pot of liquid disinfectant

For PZA Growth Control Tubes:

1. Aseptically pipette 0.5 ml of the organism suspension (used to inoculate drug tubes) into a total of 4.5 mL of sterile saline to prepare the 1:10 GC suspension (1% growth control).
2. Mix the GC suspension thoroughly.
3. Inoculate 0.5 ml of the 1:10 GC suspension into the MGIT tubes labelled "GC", using a micropipettor and aerosol resistant tips. Dispose of pipette into discard pot of liquid disinfectant
4. Spread 0.1mL of the organism suspension to a BAP.
5. Enclose the blood agar plate in a plastic bag.
6. Incubate at 35 - 37°C.
7. Check the blood agar plate at 48 hours for bacterial contamination. If the blood agar plate shows no growth, then allow AST testing to proceed. If the blood agar plate shows growth, discard the AST set (refer to the BACTEC MGIT 960 User's Manual, AST Instructions) and repeat testing with pure culture.

6.10.3.6. Inoculation of tubes containing test drugs – carried out in BSC in CL3/BSL3 laboratory

1. Aseptically pipette 0.5 ml of the organism suspension into each of the seven remaining drug tubes (STR, INH, RIF, EMB, PZA, MOX and KAN), using a micropipettor and aerosol resistant tips. Dispose of pipette into discard pot of liquid disinfectant
2. Tightly recap the tubes.
3. Mix tubes thoroughly by gentle inversion 3 to 4 times.
4. For entry into the MGIT instrument, **without** the TBeXiST software, use the carrier sets and enter AST set into the BACTEC MGIT 960 using the AST set entry feature (refer to the BACTEC MGIT 960 User's Manual, AST Instructions). Ensure that the order of the tubes in the AST set carrier conforms to the set carrier definitions selected when performing the AST set entry feature (from left to right)
    - SIRE – 5 tube carrier set (GC, S, I, R, E)

- PZA – 2 tube carrier set (GC, Z)
- MOX (2 and 0.5) – 2 tube carrier set – load as 'undefined drug' (GC, M)
- KAN - 2 tube carrier set – load as 'undefined drug' (GC, K)

5. After inoculation of MGIT tubes, spread 0.1mL of the organism suspension to a Blood Agar plate.
6. Enclose the blood agar plate in a plastic bag.
7. Incubate at 35-37°C.
8. Check the blood agar plate at 48 hours for bacterial contamination. If the blood agar plate shows no growth, then allow AST testing to proceed. If the blood agar plate shows growth, discard the AST set (refer to the BACTEC MGIT 960 User's Manual, AST Instructions) and repeat testing with pure culture.

6.10.3.7. How to interpret DST results

The BACTEC MGIT 960 instrument continually monitors all tubes for increased fluorescence. Analysis of fluorescence in the drug-containing tubes compared to the fluorescence in the Growth Control tube is used to determine susceptibility results.

Using the carrier sets, the BACTEC MGIT 960 automatically interprets these results and reports a susceptible (S) or resistant (R) result for the SIRE and PZA tests on the AST print outs

For moxifloxacin and kanamycin, because the AST has been loaded as 'undefined drug' the results need to be interpreted manually. The growth unit of the GC tube should be 400 GU, for the drug tube if the **growth units are more than 100 the isolate is resistant (R)**, whereas if the **growth units are less than 100 the isolate is sensitive (S).** It is also important to check the time in protocol (TIP) is within the acceptable timeframe of 4-13 days. If outside this range the test should be considered invalid because the growth control failed to reach 400 GU in the required time (see Error messages below). This result [Susceptible (S)/Resistant (R)/Invalid (X)] and the drug (moxifloxacin or kanamycin) should be documented on the AST print out.

All AST print outs should be labelled with the laboratory accession numbers of the samples and signed off by the member of staff unloading the tubes.

Error messages – If the AST print out shows an 'X' (X200 and X400)– this means the run has failed because the growth control tube reached 400 GU outside of the acceptable time frame:
SIRE, moxifloxacin and kanamycin- 4 to 13 days, PZA- 4-21 days.

In this case the result is invalid and no interpretation (S/R) will be shown. This could be caused by contamination with rapid growing microorganisms (including NTMs), or as a result of the inoculum being prepared incorrectly (adding too many or too few mycobacteria). These samples will need to be repeated.

6.10.3.8. Confirming resistant isolates

All resistant isolates should be verified by preparing a blood agar culture (BAC) to check for growth of contaminating bacteria (SOP 8). All resistant tubes must also be visually inspected to check for typical 'breadcrumb' morphology of MTB seen in MGIT tubes. This visual inspection and the blood agar result should be documented on the reverse of LRF 4. If there is anything unusual about the growth seen in the MGIT tube (turbidity suggestive of contamination, or atypical growth), or the resistance profile is not as expected (i.e inconsistent with previous results), a smear should be performed to confirm the presence of AFB. Staining of smears for AFB from resistant cultures will be performed using the Kinyoun or ZN stain (SOP 4). The smear result will also be recorded on the reverse of LRF4. Together these additional tests will confirm the culture was pure and the resistant result not caused by growth of contaminating bacteria.

- If the BAC shows no growth, the colony morphology is typical **and** the smear shows no concomitant flora (if performed), you can accept the resistant result.
- If the BAC shows growth and / or the smear shows concomitant flora, you cannot use the resistant result, repeat the susceptibility testing with a pure MTC culture.

As mentioned above, AFB smear is not performed routinely on the resistant cultures, but only if there is any ambiguity or sign of contamination not seen on BA. This is because:

- Nix-TB isolates are expected to be resistant to most drugs.
- The culture used to prepare the inoculum/or the subculture for the inoculum has a Hain/Xpert which confirms the presence of MTB before DSTs are set up.
- All resistant results are routinely repeated.
- BA confirms lack of contamination which could be causing false resistance.

Resistant results for SIRE, K, M and Z must be repeated for confirmation. Similarly, if the results for one of these drugs is inconsistent with previous results for the same patient – e.g., baseline result was resistant and follow-up result was susceptible – review the QC and repeat the test. If the results of the confirmatory test match the initial DST, then the results can be accepted. If the repeat testing shows a susceptible result, the data is discrepant and the DST must be repeated a third time to confirm which result is correct. It is not acceptable to automatically assume the susceptible result is the valid result. DSTs may also be required to be repeated to resolve laboratory discrepancies between DST data collected at site laboratories and the UCL Mycobacteriology Laboratory.

In addition, when sub-culturing isolates for repeat testing it is important, as far as possible, to go back to the original positive culture rather than performing multiple subcultures. This will help to minimise the risk of cross contamination or modification of drug resistance profile, through selection.

6.10.4. Quality Control

It is extremely important to perform quality control on the drug sensitivity testing procedure. This must be carried out for each new batch of reagents (drug kits and tubes), using the pan-susceptible MTB strain H37Rv (ATCC 27294), which is susceptible to all of the test drugs. If the QC fails, all results for the batch should be reviewed, new reagents purchased and testing of clinical samples repeated.

These QC results should be recorded in the NiX-TB Quality Manual Attachment Ex.

6.11. SOP 11: Minimum Inhibitory Concentration (MIC)

6.11.1. Purpose

This SOP is for use by the UCL Central Mycobacteriology Laboratory to determine the minimum concentration of each of the study drugs – pretomanid (Pa), linezolid (L) and bedaquiline (B) - that inhibits the growth of *Mycobacterium tuberculosis* (MTB) in liquid medium. Minimum Inhibitory Concentration (MIC) testing will be performed, using the BACTEC MGIT 960 instrument, on:

- Pre-treatment isolates from a baseline visit (Day 1, or Screening to Week 4 if Day 1 cultures are negative or contaminated)
- Isolates from patients suspected of treatment failure or relapse or reinfection after treatment (a positive culture at or after week 26 [6 month treatment arm)/week 36 (9 month treatment arm) and any new positive culture thereafter] in order to identify a shift in susceptibility to any of the study drugs.

NOTE: Prior to any MIC testing, a Hain MTBC assay (SOP 5) is performed at the UCL Central Mycobacteriology Laboratory to confirm the presence of MTB complex (MTBC). If the Hain test does not confirm MTBC, the local laboratory will be contacted and a new culture will be sent.

6.11.2. Principle

MIC testing is based on the same principle as the MGIT DST (SOP 11). For MIC, a range of concentrations of the test drugs (pretomanid, bedaquiline and linezolid) are added to a panel of MGIT tubes and inoculated with the test culture. A growth control (GC) tube with 1/100 the inoculum of the test isolate is included with each test and serves as a comparison for growth. If the test drug concentration is active against the mycobacterial isolate (susceptible), growth will be inhibited and fluorescence will be suppressed in the drug-containing tube (and hence growth units (GU) suppressed). The growth control (GC) and any drug concentrations at which the mycobacterial isolate is resistant will grow and will have increasing fluorescence and GU detected by the MGIT instrument.

To interpret this growth, when the GC tube reaches 400 GU the test is considered finished and the growth units are checked for every drug concentration. In the drug containing tubes, results above 100 GU are considered as resistant to that particular concentration, while anything lower than 100 GU is considered susceptible. The MIC is defined as the lowest concentration where the GU are less than 100.

For standard MGIT-based breakpoint DSTs (see SOP 11), the interpretation can be performed by the instrument and included on the antimicrobial susceptibility testing (AST) reports. For MIC tests and breakpoint DSTs (outside of the standard BD sets), the EpiCentre software (a user interface for the MGIT instrument) including the TB eXiST (TB eXtended Susceptibility Testing) module must be used. With this eXtended Susceptibility Testing module, it is possible to:

- Extend susceptibility testing to all TB isolates against primary drugs. For example, some MDR-TB and XDR strains are dysgenic and therefore may not reach a completed signal within the 13 days required for the growth control (GU>400) for automatic DST interpretation of SIRE results in MGIT instruments.
- Freely test a drug belonging to the currently described 2nd line classes, and potential new drugs.
- MIC testing of any chosen drugs at a range of freely chosen concentrations. This is the scope of using the EpiCentre and TBeXiST covered in this SOP.

6.11.3. Procedure

This MIC protocol will be carried out with all MTB isolates received at the UCL Central Mycobacteriology Laboratory eligible for DST testing as described above. MIC testing will be performed using pretomanid (Pa), linezolid (L) and bedaquiline (B), see Table 1 for manufacturer and storage details. The concentrations tested for these drugs are 2-fold serial dilutions across the following ranges: Pa - 2 to 0.063 µg/ml, L - 8 to 0.25 µg/ml and B - 2 to 0.063 µg/ml (see Table 2).

6.11.3.1. Equipment/Reagents

- Biological Safety Cabinet (BSC)
- Discard bucket containing appropriate liquid disinfectant (specified in local Health and Safety documentation)
- Vortex mixer
- Micropipettes (P-1000, P-200 and P-10)
- Filtered pipette tips (100-1000 µl tips, 10-200µl tips and 1-20 µl tips)
- Appropriate racks to contain all the necessary tubes
- 7ml MGIT tubes
- BD BACTEC MGIT supplement (OADC)
- Sterile saline
- Sterile 5mm glass beads
- Blood agar plates (BAP)
- Drug stocks – see below for preparation details
- Sterile DMSO for drug stock serial dilutions
- 1.5ml Eppendorf tubes

6.11.3.2. Preparation of drug stocks and working solutions

Table 11: Manufacturer and storage instructions for the study drugs

Drug	Code	Solubility	Storage (Powder)	Manufacturer
**Pretomanid**	Pa	DMSO	4°C	Metrics Inc
**Linezolid**	L	DMSO	-20°C	Generon
**Bedaquiline**	B	DMSO	RT	Janssen

Table 12: Drug concentrations used for MGIT MIC testing

Drug	Drug concentration (µg/ml)					
**Pa**	2	1	0.5	0.25	0.125	0.063
**L**	8	4	2	1	0.5	0.25
**B**	2	1	0.5	0.25	0.125	0.063

Drug manufacturer details and storage conditions for each of the study drugs are detailed in Table 11.

For preparation of the **stock solutions** see directions below (and Table 13):

Pretomanid (Pa): Dissolve 0.04g (40mg) of Pa in 10ml of sterile DMSO (4mg/ml). Aliquot in sterile cryotube vials volumes of 50µl or as required.

Linezolid (L): Dissolve 0.025g (25mg) of L in 1.780ml of sterile DMSO (to obtain a 14mg/ml stock solution). Aliquot in sterile cryotube vials volumes of 50µl or as required.

Bedaquiline (B): Dissolve 0.04g (40mg) of B in 10ml of sterile DMSO (4mg/ml stock solution). Aliquot in sterile cryotube vials volumes of 50µl or as required.

Once reconstituted, the aliquoted antibiotic solutions should be stored frozen at -20°C or colder for up to six months, or up to the original expiry date of the drug powder vial, whichever comes first. Once thawed, use immediately. Discard any unused portions.

Table 13: Drug concentrations used for MGIT Drug Sensitivity Testing

Drug	Dry Weight (mg)	Reconstitution volume (DMSO, ml)	Stock Concentration (mg/ml)	Dilution Factor (DF) and volume (in DMSO)	Volume added to MGIT	Final Concentration (in MGIT tube of 8.4ml total)
Pretomanid (Pa)	40	10	4	**DF 1:23.8** 42 µl to 958 µl	100 µl	2 µg/ml
Linezolid (L)	25	1.780	14	**DF 1:20.8** 48 µl to 952 µl	100 µl	8 µg/ml
Bedaquiline (B)	40	10	4	**DF 1:23.8** 42 µl to 958 µl	100 µl	2 µg/ml

The concentration described in this table corresponds to the highest of the range of Table 12. Starting from this concentration serial 2-fold dilutions in DMSO will be repeated five time to achieve the lowest concentration to be tested. The exact volumes used to prepare the 2-fold dilutions will depend on the number of samples being tested in a batch (See Step 2: Prepare and Add Drugs).

6.11.3.3. TBeXiST Workflow Description Overview

An overview of the TBeXiST workflow is as follows:

1. Request and register the MIC tests in TBeXisT, print barcodes and assign to MGIT tubes
2. Add MGIT supplement and diluted drugs to the MGIT tubes (This can be done outside the CL3 laboratory)
3. Prepare test inocula and add to the MGIT tubes (This should be carried out inside a BSC in the CL3/BSL3 laboratory)
4. Load tubes into the MGIT instrument
5. Once test complete, interpret and finalise the test
6. Remove completed tubes from the MGIT instrument
7. Print the Interpretation Report and complete LRF5 with MIC values

6.11.3.4. Step 1: Specimen Registration, order TB eXiST tests, print barcodes

For standard MIC testing in the NiX-TB study all 3 drugs will be tested together at the 6 concentrations outlined in Table 12 and compared against a single growth control. As required, individual drugs can be repeated in isolation or the concentration range adjusted if the MIC value for a given sample is out of range. For simplicity, the standard 3 drug MIC will be used as the example included in the methods.

One BACTEC MGIT 7ml tube is required per drug concentration tested and one per growth control per test group (i.e. for each test run with a given MTB isolate/Lab accession number). Hence 19 tubes are required for each sample:

- 1 tube - Growth Control
- 6 tubes - Pretomanid concentrations
- 6 tubes - Linezolid concentrations
- 6 tubes - Bedaquiline concentrations

Prior to use, examine all MGIT tubes for evidence of damage. Do not use any tube that is cracked or has other defects. Do not use a tube if the medium is discoloured, cloudy or appears to be contaminated.

Create Patient/Specimen - for further stepwise instructions on the using the EpiCenter software for specimen registration including annotated screen shots, refer to the Registering TBeXiST MGIT tube for MIC-DST Users Guide, at the page numbers shown for each step where appropriate

1. Logon to the EpiCenter by clicking the Logon icon (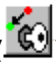), and enter the assigned user name and password. Each operator has a unique username and password which allows an audit trail to see which user performed different activities/tests.
2. Click the specimen registration icon (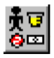) and go to the Rapid Login tab. In the Rapid Login tab, fill in the Patient ID and the Patient Name fields with the NiX-TB study patient identifier (format 00-0000-000). Assign an accession number – this will be the NiX-TB laboratory accession number and the date in the following format, e.g. P1234567_20Nov2016.

   NOTE: It is essential that the accession number field is unique to every test run (each set of tests samples and the corresponding growth control), so the date must be included to allow for repetition of testing on a single accession number. If multiple independent tests are set up on a given sample on the same day (unlikely), then run numbers will also need to be added to the accession number field e.g. P1234567_20Nov2016_run1. See Users Guide pages 1-3.

3. Order the Growth Control by selecting the "TBeX Growth Control" Test Group from the Available Tests frame. In the pop up window, the 'Isolate Number' must be completed as '1' and the 'organism ID' as '*Mycobacterium tuberculosis*'. Scan the first MGIT tube using the barcode reader – the test and the MGIT tube number will appear in the 'Ordered Tests' frame.
4. Order the Antimicrobial test groups. These are pre-programmed test groups for each drug at the concentrations specified in Table 12. Select 'TBeX-Pretomanid' to add the 6 concentrations for pretomanid to the 'Ordered Tests' frame. As above complete Isolate number as '1' and scan each the MGIT tube barcodes in order. Repeat for linezolid and bedaquiline by selecting 'TBeX-Linezolid' and 'TBeX-Bedaquiline' respectively. There will now be 18 test drug concentrations and a growth control listed in the 'Ordered tests' frame, each with a MGIT tube number listed (in sequence # column). Press save.
5. Once saved, all the new barcode labels will be printed automatically. These should then be affixed to each corresponding MGIT tube at the same height as the pre-existing MGIT tube barcode (sequence #) and without obscuring it. Cross-reference the sequence # on the MGIT tube with that on the 'Ordered Tests' frame to ensure each label is attached to the correct tube.

Figure 8: Example of printed barcodes

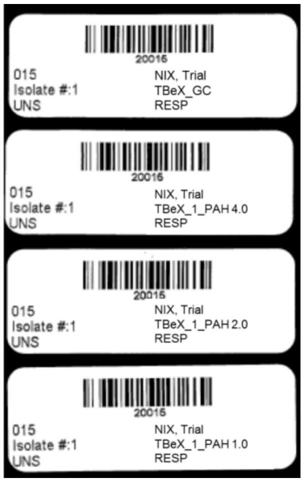

6.11.3.5. Step 2: Prepare and add drug dilutions to MGIT tubes

For preparation of the drug sets:

Aseptically add 0.8 mL of BACTEC MGIT Supplement in all tubes. BBL MGIT OADC 4 ml (cat# 245116), BBL Middlebrook OADC Enrichment (cat# 212240) or BACTEC MGIT SIRE supplement can be used.

Pretomanid and Bedaquiline

Prior to use, thaw one vial of stock solution for each drug, ensure the drug is fully dissolved and follow the steps below:

1. Take one vial of stock solution (4mg/ml) and prepare the working solution (2µg/ml final concentration in the MGIT), as per the details in Table 13, for each drug. This 1ml working solution (WS1) is the highest drug concentration and the volume is sufficient for 4 test isolates.
2. Add 500µl of DMSO to 5 screwcap Eppendorf tubes (labelled W2-W6) for the serial dilutions for each drug.
3. For each drug, take 500µl of WS1 and add to the DMSO in the WS2 tube, mix by pipetting. This will create a new, two-fold diluted working solution (1µg/ml final concentration in the MGIT).
4. Take 500µl of WS2 and add to the DMSO in the WS3 tube, mix by pipetting. This will create a new, two-fold diluted working solution (0.5µg/ml final concentration in the MGIT)
5. Repeat for all five serial dilutions for both drugs.
6. Add 100µl of each WS to the corresponding MGIT tubes. The drug and drug concentration are clearly marked on the TBeXiST barcode label.

Linezolid

Prior to use, thaw one vial of stock solution, ensure the drug is fully dissolved and follow the steps below:

1. Take one vial of stock solution (14mg/ml) and prepare the working solution (8ug/ml final concentration in the MGIT) as per the details in Table 13. This 1ml working solution (WS1) is the highest drug concentration and the volume is sufficient for 4 test isolates.
2. Add 500µl of DMSO to 5 screwcap Eppendorf tubes (labelled W2-W6) for the serial dilutions.
3. Take 500µl of WS1 and add to the DMSO in the WS2 tube, mix by pipetting. This will create a new, two-fold diluted working solution (4µg/ml final concentration in the MGIT)

4. Take 500µl of WS2 and add to the DMSO in the WS3 tube, mix by pipetting. This will create a new, two-fold diluted working solution (2µg/ml final concentration in the MGIT).
5. Repeat for all five serial dilutions.
6. Add 100µl of each WS to the corresponding MGIT tubes.

6.11.3.6. Step 3: Preparation of test inoculum and inoculation of MGIT tubes - carried out in BSC in CL3/BSL3 laboratory

Preparing inoculum from positive MGIT
Once a MGIT tube has become positive it must be used for MIC testing within 1-5 days. The concentration of the inoculum is critical to the correct performance of susceptibility testing and the following instructions must be adhered to strictly. On the day the MGIT flags positive (day 0), the culture should be re-incubated for a minimum of one extra day (day 1). This can be in the MGIT machine or in a separate 37°C incubator.

Day 1 and Day 2 – the growth in the tube can be used directly. Glass beads should be used to break up the clumps in the MGIT culture and obtain a uniform bacterial suspension. Pre-prepared sterile glass beads (minimum 4 beads, 5 mm diameter) in saline (3 ml) are used. Remove the saline by pipetting and pour all the beads into the MGIT tube. Mix well by vortexing to break up clumps as much as possible (between 2 and 10 minutes) and let any remaining clumps settle out for 30 minutes. Use the supernatant undiluted.

Days 3, 4 and 5 – the growth in the tube should be diluted before use. Vortex the MGIT culture with beads as described above and leave for 30 minutes for any remaining clumps to settle out. Dilute 2 ml of supernatant in 8 mL of sterile saline (1:5 dilution). Use this diluted culture for the MIC drug tubes.

NOTE: to be able to run a complete set of all three drugs at once, the MGIT culture must be incubated until days 3-5 and diluted as described above in order to have sufficient inocula for the 19 MGIT tubes per sample.

>5 Days – subculture into a new MGIT tube and wait for this to flag positive. Treat as above and use within 5 days to set up the MIC test.

NOTE: Cultures grown in liquid or solid media can be used to prepare a seed MGIT tube. When positive, the seed MGIT can then be used to prepare the inoculum as described above. From a liquid culture, a 1:100 dilution should be made of the broth, and 500µl added to the seed MGIT tube. For solid media, a loop of growth scraped from the slope or plate should be added to the seed MGIT tube. After incubation, the TTP of the seed MGIT tube must be 4 days or more for use as a MIC test inoculum. If the seed tube becomes positive in less than 4 days, a new seed tube should be prepared.

Preparing an inoculum from a LJ slope
1. Add 4 mL of Middlebrook 7H9 Broth (or BBL MGIT broth) to a 16.5 x 128 mm sterile tube with cap containing 8 – 10 glass beads.
2. Scrape with a sterile loop as many colonies as possible from growth no more than 14 days old, trying not to remove any solid medium. Suspend the colonies in the Middlebrook 7H9 Broth.
3. Vortex the suspension for 2 – 3 min to break up the larger clumps. The suspension should exceed a 1.0 McFarland standard in turbidity.
4. Let the suspension sit for 20 min without disturbing.
5. Transfer the supernatant fluid to another 16.5 x 128mm sterile tube with cap (avoid transferring any of the sediment) and let sit for another 15 min.
6. Transfer the supernatant fluid (it should be smooth, free of any clumps) to a third 16.5 x 128mm sterile tube. NOTE: The organism suspension should be greater than a 0.5 McFarland standard at this step.
7. Adjust suspension to a 0.5 McFarland standard by a visual comparison with a 0.5 McFarland turbidity standard. Do not adjust below a 0.5 McFarland Standard.
8. Dilute 2 mL of the adjusted suspension in 8mL of sterile saline (1:5 dilutions).

Growth Control tube preparation and inoculation

1. Aseptically pipette 0.1 ml of the organism suspension (prepared as described above from the MGIT or LJ subculture) into a total of 10mL of sterile saline to prepare the 1:100 GC suspension (1% growth control).
2. Mix the GC suspension thoroughly.
3. Inoculate 0.5mL of the 1:100 GC suspension into the labelled Growth Control (GC) MGIT tube, using a micropipettor and aerosol resistant tips. The Lab Accession Number and Patient Number will be clearly marked on TBeXiST barcode label.
4. Mix tubes thoroughly by gentle inversion 3 to 4 times.

Inoculation of tubes containing test drugs

1. Aseptically pipette 0.5 ml of the organism suspension into each of the 18 remaining drug tubes (6 concentrations for each of the 3 test drugs), using a micropipettor and aerosol resistant tips.
2. Tightly recap the tubes.
3. Mix tubes thoroughly by gentle inversion 3 to 4 times.

Purity check

1. To ensure the inoculum that was used to set up the MIC test is not contaminated, spread 0.1mL of the organism suspension to a blood agar plate (BAP).
2. Seal the BAP in a plastic zip-lock bag.
3. Incubate at 35 - 37°C.
4. Check the BAP at 48 hours for bacterial contamination. If it shows no growth, then allow MIC test to proceed. If the BAP shows growth, discard the MIC tubes and repeat testing with pure culture.

6.11.3.7. Step 4 - Tube Loading

In the BD BACTEC™ MGIT™ software, the option "Accession Barcoding" must be enabled. This will allow the MGIT Instrument to scan both the MGIT tube barcode (sequence #) and the Accession barcode (on the TBeXiST generated barcode label)

NOTE: As all tubes belong to the same TB eXiST set (drug containing tubes and growth control for a given sample), they must have the same start-of-test date and time. **THEY MUST ALL BE LOADED SIMULTANEOUSLY IN THE SAME DRAWER** of the BD BACTEC MGIT instrument by scanning their sequence barcode number and accession barcode number. DO NOT CLOSE THE DRAWER UNTIL ALL TUBES HAVE BEEN ADDED. It is also strongly recommended not to open any drawer during the reading window [hour - 2 minutes (:58) till hour + 5 minutes (:05)] so no tube readings are missed.

1. Open a drawer and press the "Tube Entry" Soft Key

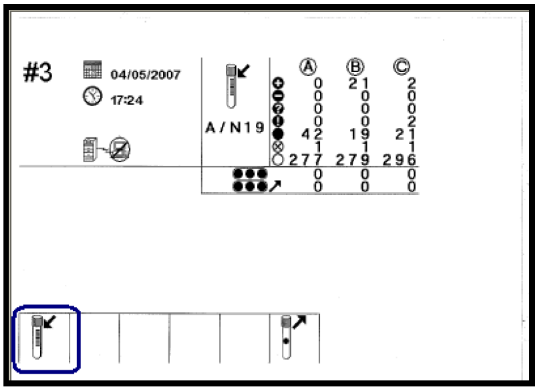

2. Scan the Tube Sequence #

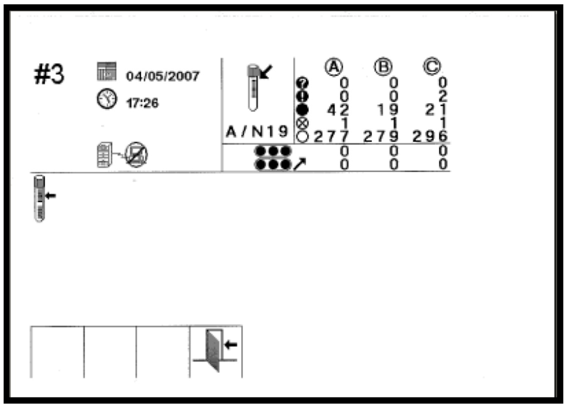

3. Scan the Accession # (TBeXiST barcode)

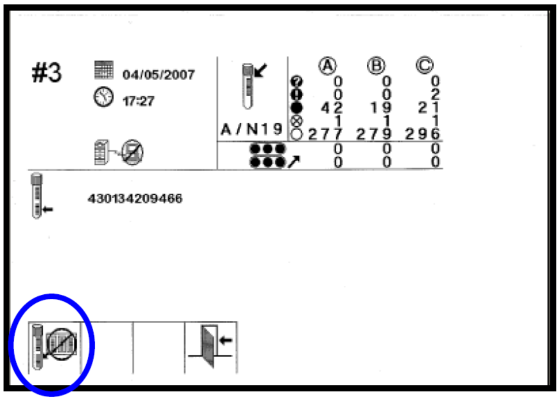

4. Load the tube into the instrument - Insert the tube in the station with the green indicator.
5. Repeat steps 2-4 for all remaining tubes and close the drawer

An Accession # associated to TB eXiST tests MUST NOT BE MODIFIED AT ANY TIME. Once inserted into the instrument, a tube MUST NOT BE MOVED to another position in the same drawer or another drawer.

6.11.3.8. Step 5: Interpreting and Finalising the results of completed test runs

For further stepwise instructions on using the EpiCenter software for interpreting and finalising results including annotated screen shots, refer to the 'Interpretation and Reporting of MIC-DST in TBeXiST Users Guide'.

1. Login to the EpiCenter as described above. Click on the Reports icon, and in the 'Filter Reports' tab, select the TBeXiST Worklist. Click on 'Print Preview', select 'Enter the minimum GU value for the GC' from the table, enter 400 in the space provided, and click 'Run'. This will bring up a listing of ALL MIC samples in the MGIT instrument for which the Growth Control has reached 400GU and therefore the test is complete and ready to be interpreted and finalised.
2. Print the TBeXiST Worklist, staple together the pages and sign and date. This worklist shows the growth units in all the drug containing tubes at the point the growth control reached 400GU (the first MGIT instrument reading where the GC ≥ 400 GU). It also shows the GU at the time of generation of the worklist (if different). It is the column at the timepoint that the GC≥400GU that must be used for the interpretation (see Figure 9 for an example TBeXiST worklist).
   NOTE: This signed worklist must be kept in the NiX-TB lab file as it is the source data for the GU in each tube at the time of interpretation.

Figure 9: Example of TBeXiST worklist

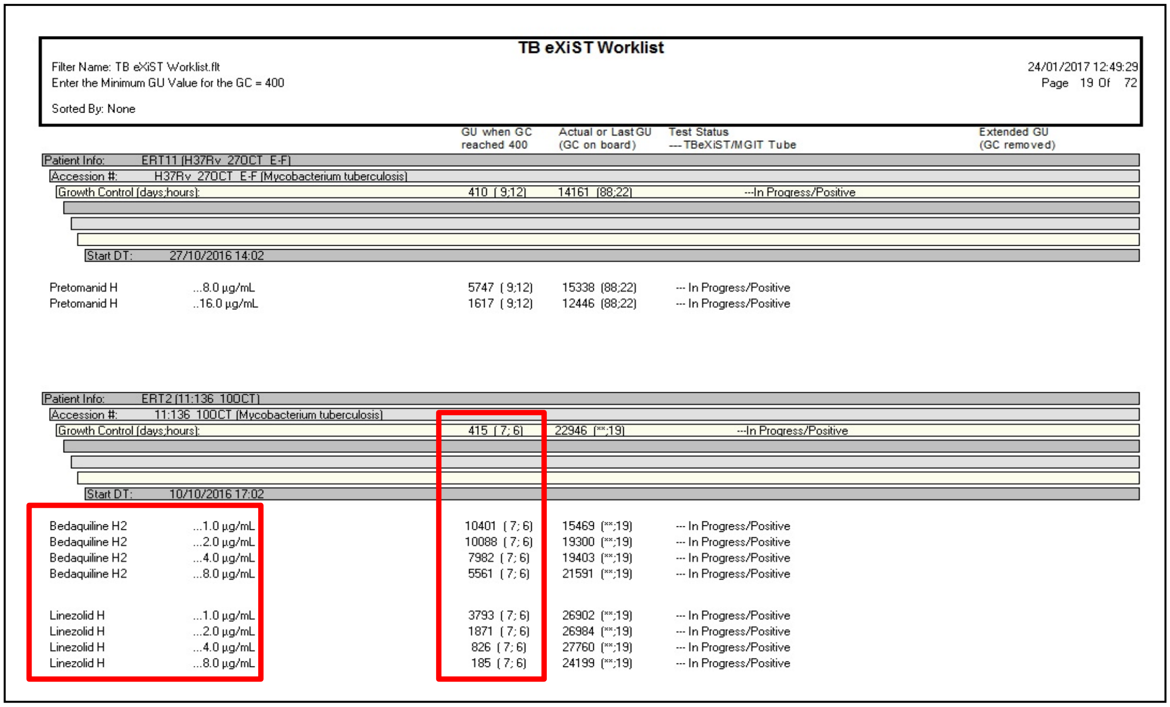

3. Generate TBeXiST plots for all of the test drugs – this is a graphical representation of the GU seen in the worklist, with a growth curve shown for each drug concentration. See Figure 10 for an annotated example. To do this, click on the 'Reports' icon, and in the 'Filter Reports' tab, select 'BACTEC MGIT TBeXiST plot'. Click on 'Print Preview', select 'Enter Accession Number' from the table and enter the accession number of interest, then select 'Drug' and enter the drug name. Click 'Run'. A separate plot has to be created for each drug. To print these plots in colour, they need to be saved to file and transferred out of the laboratory using a memory stick. To do this, select 'Export', select a JPEG file type, and save in the NiX-TB eXiST Plots folder using the filename format of Patient No_Lab Accession No_Date_Drug. Next carry out the interpretation and finalising of result. Click on the 'Data View' icon, and select 'TBeXiST Specimens in Progress'. From the sample listing table select the first Lab Accession Number on the TBeXiST worklist. Right click and select 'Specimen Registration' option. This is the window where each drug concentration is assigned a susceptible or resistant interpretation based on the GC noted on the worklist.
4. Select the Growth Control tube from the 'Ordered Tests' frame, choose 'Threshold 400 Reached' from the 'Test Status' window and tick the 'Finalised' box.
5. Working through each drug containing tube – select the tube from the 'Ordered Tests' frame and refer to the GU for the selected drug on the printed worklist. If the GU are ≥100, choose 'Complete-R' from the 'Test Status' window,

‘R’ from the Interpretation drop down menu, and tick the ‘Finalised’ box. If the GU are <100, choose ‘Complete-S’ from the ‘Test Status’ window, ‘S’ from the Interpretation drop down menu, and tick the ‘Finalised’ box. See Users guide pages 11-16.

6. When this is complete for all 18 drug containing tubes, click ‘Save’

Figure 10: Example of a TBeXiST plot, annotated to show the ‘resistant’ and ‘Susceptible’ growth curves relative to the Growth Control

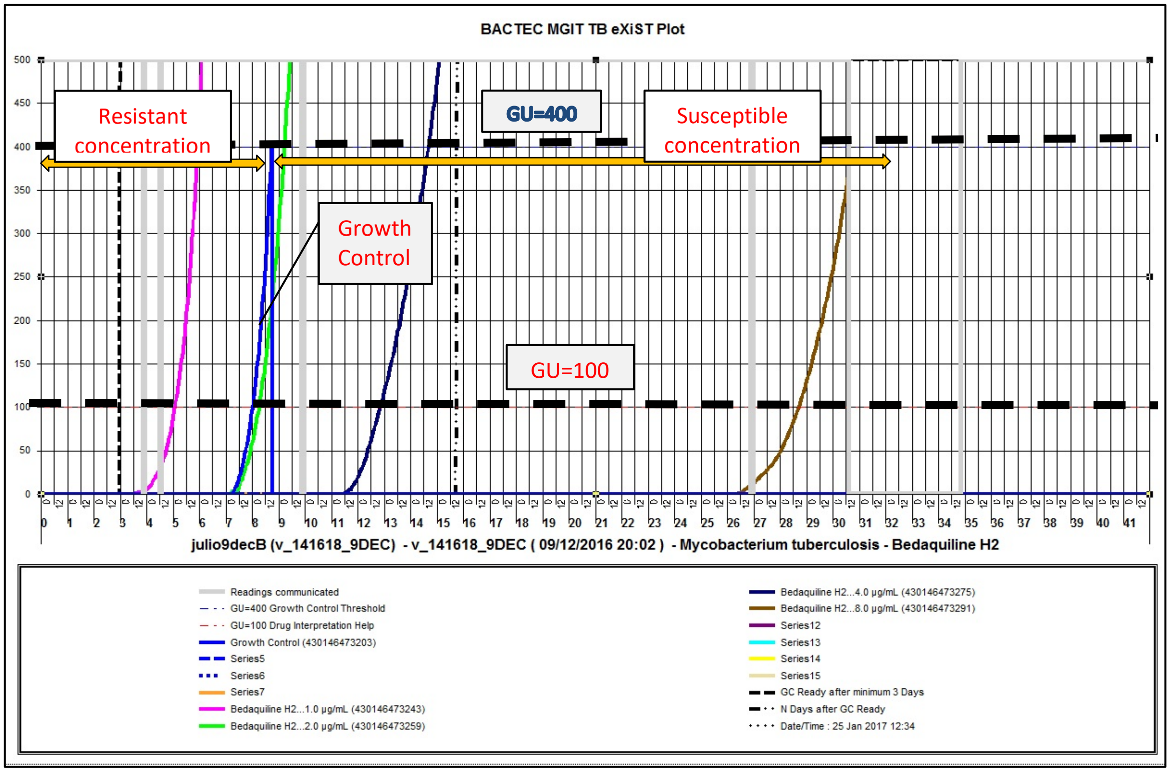

To print these plots in colour, they need to be saved to file and transferred out of the laboratory using a memory stick. To do this, select ‘Export’, select a JPEG file type, and save in the NiX-TB eXiST Plots folder using the filename format of Patient No_Lab Accession No_Date_Drug.

6.11.3.9. Step 6: Generate Interpretation Report and complete LRF5 with MIC values

1. To generate an Interpretation Report of the finalised results, click on the Reports icon, and in the ‘Filter Reports’ tab, select ‘TBeXiST Interpretation Report’. Click on ‘Print Preview’, select ‘Enter Accession Number’ from the table, enter the Accession Number of report to be generated, and click ‘Run’.
2. Print the TBeXiST Interpretation Report for each sample. One report will include all the drugs and concentrations tested. An example report is shown in Figure 11.
3. Annotate the printed report with the MIC concentration for each drug. **The MIC is the lowest concentration of a given drug that has been assigned a ‘Susceptible’ status (and therefore has growth units <100).** As the assigning of ‘Susceptible’ and ‘Resistant’ status is done manually, the Interpretation report should be cross checked with the corresponding Worklist print out (showing the GU) to double check the MIC is correct. The Interpretation report should then be signed and dated.
4. MIC values for each drug are then entered from the Interpretation report into LRF5. If the MIC is out of the range of concentrations tested, it should be reported as >2µg/ml or ≤0.06µg/ml for Pa, >8 µg/ml or ≤0.025µg/ml for L and >2 µg/ml or ≤0.06µg/ml for B. If required, a further round of testing can be performed at a higher/lower set of concentrations as appropriate. This should be noted in the comments section of the LRF if required.
5. Checks for transcription accuracy are performed as part of the verification of the LRF, comparing the LRF to the Worklist print out (showing the GU), and the LRF is signed off by the Laboratory Manager or delegate.

6.11.3.10. Step 7: Remove completed MGIT tubes from the instrument

1. The next step is to remove the completed tubes (those that have been finalised in the above process). Click on the 'Data View' icon, and select '3. Remove and Finalise'; '1. TBeXiST Completed tubes'.
2. This shows the list of all tubes that have now been assigned a S/R interpretation and finalised. The table shows drawer and tube position. Print this list and take print out into the CL3/BSL3 laboratory to identify and unload all the required tubes through the normal process of 'Unloaded Ongoing', 'Unloaded Positives' and 'Unloaded Negatives'. After unloading, click 'Refresh' and all samples in this list will disappear. Keep the unloaded tubes in case further processing is required – i.e. performing blood agar culture or ZN (SOP4) in the case of suspected contamination or a putative 'resistant' result (see section 1.1.4 Confirming resistant results).
3. Next click on the 'Data View' icon, and select '3. Remove and Finalise'; '3. TBeXiST Removed Positives'. This is the list of all positive tubes that have now been assigned a S/R interpretation, finalised and removed from the MGIT machine. Select all records, right click and select 'Assign Test Status' from the pop up menu. Select 'TB-eXiST- Complete' and click 'OK'.
4. Next click on the 'Data View' icon, and select '3. Remove and Finalise'; '2. TB-eXiST – Removed Ongoings' and repeat step 3 to assign them a completed test status.
5. To Finalise tubes with a Negative Status that have been removed from the MGIT instrument (this will be infrequent as it will only occur if the MGIT tubes are left in the instrument for the standard 42 day protocol required for growth and detection), click on the 'Data View' icon, and select 'TBeXiST Specimens in Progress'. From the sample listing table select the Lab Accession Number of the sample to be finalised. Select the tube(s) with negative status, and choose TB-eXiST complete in the 'Test Status' window, and 'Save'.

Figure 11: Example of TBeXiST Interpretation Report

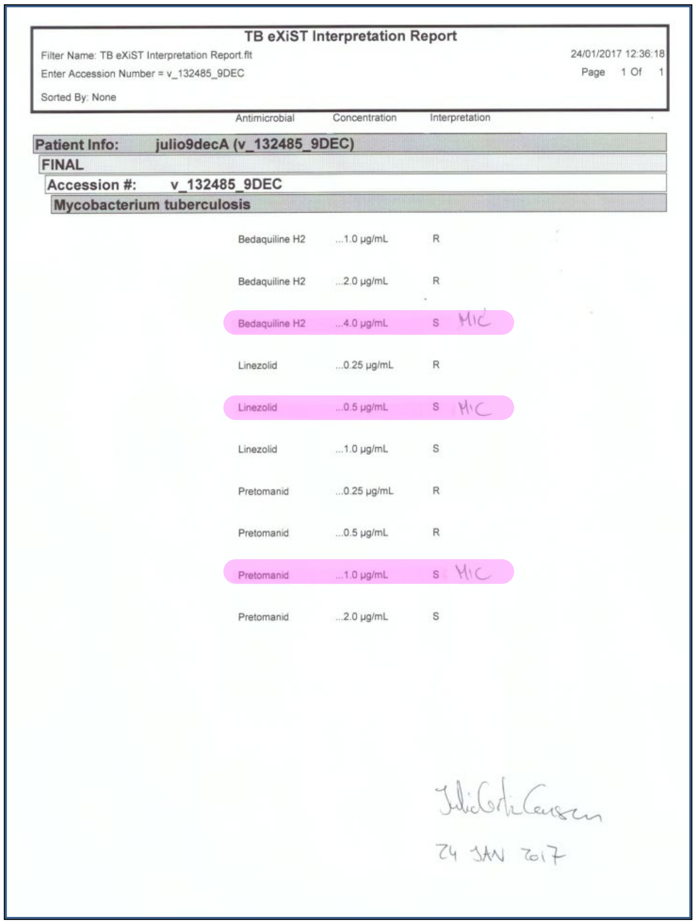

TB eXiST Interpretation Report

Filter Name: TB eXiST Interpretation Report.flt — 24/01/2017 12:36:18
Enter Accession Number = v_132485_9DEC — Page 1 Of 1
Sorted By: None

Patient Info: julio9decA (v_132485_9DEC)
FINAL
Accession #: v_132485_9DEC
Mycobacterium tuberculosis

Antimicrobial	Concentration	Interpretation	
Bedaquiline H2	...1.0 µg/mL	R	
Bedaquiline H2	...2.0 µg/mL	R	
Bedaquiline H2	...4.0 µg/mL	S	MIC
Linezolid	...0.25 µg/mL	R	
Linezolid	...0.5 µg/mL	S	MIC
Linezolid	...1.0 µg/mL	S	
Pretomanid	...0.25 µg/mL	R	
Pretomanid	...0.5 µg/mL	R	
Pretomanid	...1.0 µg/mL	S	MIC
Pretomanid	...2.0 µg/mL	S	

24 JAN 2017

6.11.4. Confirming resistant results

1. For any samples where the MIC is greater than the suggested 'resistant' threshold concentrations outlined in Table 14, a BAP and AFB smear should be prepared to confirm presence of AFB and rule out contamination. This should be done from the highest tube where growth was observed (the one before the MIC), and if necessary the growth control. The blood agar and ZN results should be recorded in the table provided on the reverse of LRF5. If the MIC result is valid (AFB positive and no contamination present), the MIC test should be repeated to confirm the MIC value in a second test. Variability of one dilution either side of the first MIC value is acceptable for the repeat to be valid.

Table 14: MIC cut-off values for each drug, for a sample to be considered resistant and therefore require further testing

**Drug**	Pa	L	B
**MIC (µg/ml) 'Resistant' Threshold Concentration**	≥1 µg/ml	≥2 µg/ml	≥1 µg/ml

2. If contamination is suspected in any samples, visually inspect the tubes, plate onto blood agar and prepare a smear for AFB staining (SOP 4). The blood agar and ZN results should be recorded on the reverse of LRF5.
3. For all samples where contamination is detected, the H37Rv QC from the run is out of the acceptable range (see Quality Control section below), or the results are not clear for another reason (clearly state the reason in the comments section of LRF5), the test must be repeated.

NOTE: If MTB isolated at/after end of treatment is found to have MIC above cut-off (R), whereas the baseline match for the same patient has MIC below cut-off (S), the repeat, confirmatory test should be done with both isolates in parallel.

6.11.5. Quality Control

It is important to perform quality control on the MIC testing procedure. This must be carried out for each new batch of drugs (Pa, L and B), MGIT tube and supplement lots, using the MTB reference strain H37Rv (ATCC 27294) which is susceptible to all of the test drugs. This should be recorded on NiX-TB Quality Manual Attachment Eiii and Exi. In addition, the H37Rv reference strain should be included on each run to check for MIC performance and control for any variability. The results of a given test run should only be accepted if the H37Rv passes the QC giving an acceptable result (see Table 15). The results of this QC are recorded on the Interpretation Report for the H37Rv sample (signed and dated by the laboratory staff member to confirm the result was acceptable). If the QC fails, all results for the batch should be reviewed and, if necessary, new reagents purchased and prepared, and testing of clinical isolates repeated.

Table 15: Acceptable MIC range for the *MTB* H37Rv reference strain quality control

**Drug**	Pa	L	B
**MIC (µg/ml)**	0.12 and 0.25	0.5 and 1	0.125, 0.25 and 0.5

6.11.6. Documentation

LRF5 captures the information for completing the eCRF fields (highlighted) related to MIC testing

6.12. SOP 12: Whole Genome Sequencing (WGS)

6.12.1. Purpose

Used by the **UCL Central Mycobacteriology Laboratory (UCL),** and Genomic Services and Development Unit, Public Heath England (PHE) for molecular typing by Whole Genome Sequencing (WGS) of paired DNA extracts from the isolates at baseline (Day 1, or Screening to Week 4 if Day 1 cultures are negative or contaminated) and the first positive culture at or after the end of treatment [week 26 (6 month treatment arm)/week 39 (9 month treatment arm)]. This data will be used to determine if the paired isolates are the same strain (relapse) or different strains (re-infection), the outcome of which is important for assigning study endpoints.

6.12.2. Principle

DNA will be extracted and quantified as per SOP 9: DNA Extraction at UCL and sent to PHE. DNA will be sent in batches for the baseline samples and for cases of suspected re-infection or relapse (as defined above), these will be prioritized with the corresponding baseline (if not already processed). At PHE, WGS is performed using the Illumina Nextera XT DNA Library Preparation kit and sequencing performed on Illumina sequencers. Data will be analyzed using the assembly and mapping pipeline outlined in detail below. The WGS data from the paired isolates will be compared and number of single nucleotide polymorphisms (SNPs) different determined. WGS data will also be used to identify mutations in known genes associated with resistance to anti-TB drugs. Isolates may also be sent for WGS to resolve discrepancies in laboratory data.

6.12.3. Procedure

6.12.3.1. DNA requirements for WGS analysis

DNA must be extracted and quantified as per SOP 9. Details of the quantification (using Qubit and Nanodrop) must be recorded onto the DNA Quantification Worksheet (Appendix I). To be acceptable to be sent for WGS analysis the extracted DNA from each sample should meet the following criteria:

- 260/280 ratio between 1.8 and 2.0
- Qubit® dsDNA concentration between 6 – 100 ng/μl
- Minimum volume of 60 μl

If this concentration or volume is not achieved, samples with lower concentrations may be able to be processed but this needs to be agreed with PHE beforehand. A copy DNA quantification worksheet (Appendix I) should be emailed to the address stated in Table 16 before the sample shipment, flagging any samples that are below the submission range stated above, and PHE can advise accordingly which samples can be accepted and any additional information required.

If required, DNA should be diluted in molecular grade water to within the optimal concentration range described above, and dilution details documented on the DNA quantification worksheet (Appendix I). If TE buffer is used for dilution the concentration of EDTA should be below 0.1 mM. If there is sufficient DNA, two aliquots will be prepared – one aliquot will be sent to the PHE, and the other will be stored at UCL as a back-up. If not sufficient DNA, a back-up will not be kept at UCL. DNA should be stored at -20°C at UCL until shipment is required.

If the above DNA requirements criteria are not met for a sample, the DNA extraction must be repeated from a fresh culture stored at UCL. If multiple samples in a batch of DNA extractions do not meet those criteria then the extraction reagents

should be reviewed (expiry dates, storage conditions) and new lots prepared as required and a CQIF (Quality Manual Attachment M) completed.

All DNA samples should be provided to PHE in clear 4titude PCR full skirted plates with unique PHE barcode on the left plate edge A1-H1 side. These must be purchased from 4titude, UK (catalogue number SP-0238). Up to 94 samples can be submitted per plate and wells G12 and H12 should be left empty for controls. There is no minimum sample number. The layout of the samples in the plate must match the online order submission. The online sample sheet must be completed and must include a unique sample ID and the sample names cannot include the following characters: spaces \ / : * ? " < >). For the NiX-TB study this should be the patient identifier and the lab accession number, e.g. 01-9031-022_P1136542. This number together with the plate barcode will be used to track samples at all times during processing. Full details of the online submission process are outlined in guidance document 'BW0303 Instructions on sample submission and receiving results' (Appendix 7).

Sample information is submitted using the NGS LIMS (Laboratory Information Management System) also known as Genesifter, and results are accessible using an FTP client (e.g. FileZilla software). Users are required to request a NGS LIMS account through the Infectious Disease Informatics helpdesk and will be required to provide a charge code or purchase order number. When submitting the order details through LIMS the order name for submission on Genesifter should be the same as the submission plate barcode. When the online submission is complete, the UCL laboratory manager will receive email confirmation, and will be notified if there are any issues that need to be addressed before the samples are sent. This email will be printed and kept in Nix-TB UCL file.

6.12.3.2. Shipment of DNA from the UCL to PHE.

DNA should be transported under cold conditions (4°C). A polystyrene box with enough ice packs to maintain ~4°C temperature for duration of shipment, including delays, is acceptable. This shipment is non-hazardous and does not require temperature monitoring.

Shipments should be arranged through the courier City Sprint – courier, sender and recipient details are included in the Table 16. City Sprint will provide the required packaging. If necessary, shipments may be delivered by member of the UCL in person.

Table 16: Contact Details for Shipment of DNA from UCL to PHE

**Courier Details Arranging delivery of packaging materials and collection of DNA**	**City Sprint**<br>Contact details:<br>General - LondonEastHealthcareCT@citysprint.co.uk<br>Rebecca Allen - RAllen@citysprint.co.uk
**Shipper Details**	Centre for Clinical Microbiology<br>University College London,<br>2nd Floor Royal Free Hospital,<br>Rowland Hill St.<br>NW3 2PF, London<br><br>Prof. Tim McHugh (Centre Director): t.mchugh@ucl.ac.uk<br>Tel: +44 (0)207 4726402<br><br>Dr. Julio Ortiz-Canseco: julio.canseco@ucl.ac.uk

	Dr Anna Bateson: a.bateson@ucl.ac.uk<br>Tel: +44 (0)207 794 0500 Ext 31148, 331146
**Recipient Details**	GSDU/Central Stores<br>National Infection Service<br>Public Health England<br>61 Colindale Avenue<br>London, NW9 5EQ<br>Contact telephone numbers: 020 83277898<br><br>**NGS.service@phe.gov.uk**

UCL will liaise with PHE to arrange a suitable day for shipment of samples. It is necessary to notify at least one day in advance. Delivery should be between 9:00 and 16:00 Monday - Thursday.

6.12.3.3. Receipt of DNA at PHE

On arrival, samples will be stored at +2-8°c until ready for sample receipt. During sample receipt the online order and plate will be checked to confirm sample numbers match and the plate will be checked for sample volume. The plate will then be set to "received" through Genesifter and the customer (the UCL laboratory manager) submitting the order will receive an email. If there is a discrepancy the customer will be contacted by email to agree the way forward. This communication is logged through Genesifter. The email correspondence related to discrepancies/sample rejection will be printed and kept in Nix-TB UCL file.

Samples will be rejected for the following reasons:

- Sample concentration is not within the specified range
- Sample volume is different from specified volume
- Samples submitted are not in the designated 96-well plate
- Online submission form has not been completed
- Plate layout does not match the online submission form

At PHE, customer submission plates will be stored in designated storage boxes at +2-8°C for a maximum of 12 weeks from the initial reception date as indicated on Genesifter. After this time the plates will be discarded and the disposal logged onto the disposal log. External customers will have a period of 12 weeks from the initial reception date to arrange appropriate collection of the plate if required. All returned plates will be logged on Genesifter with the date of return. Customer sequencing data is stored on the individual instrument hard drive for a maximum of two months after which it is deleted. Customer data can be retrieved from the computing infrastructure managed by the PHE Bioinformatics Unit and will be retained for a maximum of 6 months.

6.12.3.4. *M. tuberculosis* genotyping by Whole Genome Sequencing

DNA Processing and Sequencing

Libraries will be prepared from the MTB DNA extracts using the Illumina Nextera XT DNA sample preparation kit.

Sequencing of the prepared libraries will be performed using Illumina sequencers and PHE aims to provide a yield per sample of approximately 150 Megabases (Mb) or higher of high quality (Q30 and above), measured by the yield of the control DNA included in each run (this is approximately equivalent to 30-fold coverage for a 5 Mbp size genome).

All reagent batch numbers, instruments used and other processing details are recorded on PHE paperwork. On receipt of the samples and the online order, PHE quantifies the submitted DNA using the Quant-iT ds BR assay kit (Life Technologies, UK). If the DNA submitted is not in the required or pre-arranged concentration it may be rejected.

All PHE SOPs used for processing and sequencing MTB DNA extracts are outlined in Table 17 below.

Table 17: PHE SOP list

SOP no.	Title	Process used for
B13132	NGS Service Sample Receipt of Customer Submissions	Main Processing
B13141	NGS Service Quantification of Customer Submissions	Main Processing
B13142	NGS Service Consolidation of Customer Submissions	Main Processing
B13133	NGS Tracking and Automation of Sample Preparation	Main Processing
B13134	NGS Service Nextera XT Library Preparation	Main Processing
B13135	NGS Service Fragment Sizing of Nextera XT Library Preparation	Main Processing
B13102	NGS PAL creation and Real-Time PCR Quantification	Main Processing
B13136	NGS Service loading of cBot and HiSeq	Main Processing
B13143	NGS Service loading of MiSeq	Main Processing
B13122	Quantification of dsDNA using Quant-iT HS kit on a microplate fluorometer	Quantification of dsDNA
B13140	Quantification of dsDNA using Quant-iT Assay Kit on Qubit Fluorometer	Quantification of dsDNA: Qubit
B13124	Illumina Nextera XT DNA Library Preparation	Library Prep
B13123	Preparation & Dilution of Samples for NGS	Sample Prep
B13138	Use & Maintenance of Perkin Elmer Robots	Perkin Elmer Robot
B13139	Use & Maintenance of BeckMan BioMek NXP Robotics	Biomek Robot
B13125	Preparation and use of HT DNA High Sensitivity LabChip Kit and LabChip GX Instrument	LabChip
B13126	qPCR for Illumina Sequencing Platforms using the KAPA library Quantification Kit	qPCR/KAPA
B13127	Preparing DNA Libraries for Loading onto Illumina Sequencing Platforms	Library Prep for loading
B13130	Use & Maintenance of HiSeq	HiSeq

B13129	Use & Maintenance of cBot	cBot
B13128	Use & Maintenance of MiSeq	MiSeq
B13131	Final NGS Work Flow (Phase 1)	NGS Workflow

On completion of a sequencing run GSDU check the quality of the run by assessing the negative control and the positive *E. coli* K12 control. A minimum of 150 Mb of Q30 and above should be obtained for the positive control which is equivalent to 30-fold coverage for a 5 Mbp size genome.

Receipt of Sequence data at UCL**.**

UCL will receive an automated email through GeneSifter to notify when the run is completed and the results are available. This email will be printed and kept in Nix-TB UCL file. The original data output is in the format of a compressed FASTQ file (fastq.gz -two files per sample) which are used for all downstream analysis. FASTQ files can be downloaded from the FTP server as described in document BW0303 (Appendix 7). It is recommended that the FTP server is accessed using FileZilla software which can be downloaded online. All FASTQ files and the Quality Report for a given run should be downloaded and saved on the UCL 'S:drive' in the 'Nix Sequence data' folder. This is a UCL shared folder with restricted access to staff delegated to be working on the study, and is backed up in accordance with Central UCL IT policy.

Data analysis by the designated study bioinformatician/s

Upon receipt of the data, UCL will contact by email the bioinformatician responsible for analysing the sequence data, who will also access the files as required through the FTP site as described above. UCL will send a table to the bioinformatician listing the Patient ID and laboratory accession numbers for the samples that require analysis, including if they are for a paired isolate analysis or baseline only analysis. The email correspondence with the bioinformatician will be printed and kept in Nix-TB UCL file. Analysis will be performed as described in Witney et al (2017) and outlined in full below:

1. Sequence Quality Control
   - Count reads
   - Align genome with reference strain H37Rv (RefSeq accession: NC_000962.3) using bwa mem (Li, 2013). This generates the BAM file (see Table 18)
   - Sort alignments, and remove duplicates with SAMtools (Li et al, 2009).
   - Reject if Coverage <30x for the aligned sequence or there is significant sequence contamination and do not proceed with further analysis. If the read count is high and the coverage is low, Kraken, a sequence classification tool, will be used to speciates the reads.

   NOTE: If coverage < 30x and sequence is confirmed MTBC, the bioinformatician will inform UCL and UCL will discuss with PHE possible reasons for the low coverage and agree if this can be resolved by re-doing the WGS run (from existing DNA), or if DNA extraction should be repeated and WGS run again from the new sample. If sequence is confirmed non-MTBC (or significantly contaminated), the DNA extraction must be repeated from a confirmed clean MTB isolate at UCL, or if this is not possible, request a new alternative isolate from the local/regional laboratory. These email correspondences will be printed and kept in Nix-TB UCL file.

2. Sequence analysis pipeline
   - Call all genome site positions (this generates a Variant Call Format file (VCF) for all sites – see Table 18). Site statistics are generated using SAMtools mpileup
   - For phylogenetic analysis, first filter sites on the following criteria:

  - mapping quality (MQ) above 30
  - site quality score (QUAL) above 30
  - at least four reads covering each site with at least two reads mapping to each strand (DP4)
  - at least 75% of reads supporting site (DP4) and an allelic frequency of 1 (AF).

  Sites that failed these criteria in any isolate are removed from the analysis.
- INDELS are identified using SAMtools mpileup as above, but setting the minimum fraction of gapped reads for candidates to 0.05. INDELS are filtered out from phylogenetics analysis (including paired isolate comparison - relapse/reinfection)
- Call all variants from H37Rv (this generates a variant call only VCF – see Table 18). Gene annotation is generated for all variant calls using snpEff software.
- Identify SNP variants between the baseline and follow up samples from the same patient to determine relapse/reinfection using above criteria. For all paired isolate analysis, the following will be reported in the 'Annotated SNP list for Paired Analysis' (see Table 18):
  - number of SNPs different
  - the genome position (nucleotide position and codon position (where applicable)
  - the gene (if applicable) – otherwise that it is intergenic and if so any further information generated by snpEff – i.e. proximity to upstream or downstream genes that might indicate SNPs in promoter or other regulatory regions)
  - the variant call (e.g. A->G)
  - variant type (e.g. synonymous/mis-sense)
  - the amino acid change (if applicable)
- All sequences will be used to reconstruct a study-wide phylogenetic tree. Phylogenetic reconstruction will be performed using RAxML (Stamatakis, 2014), with a General Time Reversible (GTR) model of nucleotide substitution and a Gamma model of rate heterogeneity; branch support values are determined using 1000 bootstrap replicates. This will be updated with each new round of WGS data available for the study.
- Assess for the presence of SNPs in the following resistance genes:
  - the 6 genes currently known to be associated with resistance to Pa-824 resistance (*fbiA*, *fbiB*, *fbiC*, *ddn*, *fgd1* and *cofC*). For all resistance genes the following will be reported in the 'Annotated SNP list for PA-824 resistance' (see Table 18):
    - the genome position (nucleotide position and codon position (where applicable)
    - the variant call (e.g. A->G)
    - variant type (e.g. synonymous/mis-sense)
    - the amino acid change (if applicable)
  - Key resistance determining genes for first and second line drugs – this will be done using a publicly available analysis platform.
- Lineage – the lineage/sub-lineage will be assigned according to the classification described in Coll et at (2014), this will be reported alongside the Pa-824 resistance data output (see Table 18).

NOTE: All analysis will be performed using the current versions (updates to be made annually if applicable) of the software packages listed above. As part of the data output (see Table 18), there is a report which details the version numbers used for each batch of samples analysed.

Table 18: Data files generated during the sequence analysis

File Type*	Details
FASTQ.gz (compressed FASTQ)<br>*2 files per sample*	Raw sequence data file/s generated by PHE
BAM<br>*1 file per sample*	Sequence data after alignment with H37Rv (reference strain NC_000962.3)
VCF (all sites)<br>*1 file per sample*	All site calls
VCF (variant calls only, including additional gene annotation generated using snpEff software)<br>*1 file per sample*	A subset of the above which includes only the variant calls (SNPs compared to reference strain)
Annotated SNP list– all paired analysis (relapse and reinfection). Excel file<br>*1 file per pair*	Study specific proforma, generated from comparison of the two VCF files (baseline and follow up) to show annotation of all variants identified between the two strains.
Annotated SNP list (PA-824 resistant genes and lineage) – all samples. Excel file<br>*1 file per sample*	Study specific proforma, generated from the VCF file to show details of all variants in the Pa-824 resistant genes and the strain lineage
Phylogenetic Tree - initial output as a newick file, and also as png image file.<br>*1 file per study*	Cumulative phylogenetic tree showing the relatedness of all samples from the study as WGS data is available
Analysis software version<br>*1 file per batch of samples analysed*	Documents the version of all software used for the sample analysis

***All files will be named to include the sample ID which includes the patient identifier and the lab accession number**

NOTE: The VCF files (all sites and variant calls) will be available for all samples allowing the annotated SNPs to be interrogated further at a later date, should this be required.

Transfer of Analysis Data to UCL

UCL will be informed by email when data is ready and all data files listed in Table 18 (excluding the FASTQ which is transferred as described above) will be made available to UCL using a password protected web portal https://bugs3.sgul.ac.uk/data/bateson/. These will be downloaded and stored on the UCL S: Drive as described above.

6.12.4 Reporting

The required paired analysis data (No. of SNPs different between the baseline and follow up samples) will be entered on to LRF 6 (Appendix H) from the appropriate annotated SNP list file (see Table 18 above). For paired isolates where number of SNPs different is <20, the appropriate annotated SNP list must also be printed and attached to the LRF for ease of reference. For pairs with >20 SNPs different, the full annotated list will be available as electronic copy only as indicated in Table 18.

6.12.5 References

1. Witney AA, Bateson AL, Jindani A, Phillips PP, Coleman D, Stoker NG, Butcher PD, McHugh TD; RIFAQUIN Study Team. Use of whole-genome sequencing to distinguish relapse from reinfection in a completed tuberculosis clinical trial. BMC Med. 2017 Mar 29;15(1):71.
2. Li H. Aligning sequence reads, clone sequences and assembly contigs with BWA-MEM. ArXiv13033997 Q-Bio. 2013. http://arxiv.org/abs/1303.3997.
3. Li H, Handsaker B, Wysoker A, Fennell T, Ruan J, Homer N, et al. The Sequence Alignment/Map format and SAMtools. Bioinformatics. 2009;25(16):2078–9.
4. Stamatakis A. RAxML version 8: a tool for phylogenetic analysis and post-analysis of large phylogenies. Bioinformatics. 2014;30(9):1312–3.
5. Coll F, McNerney R, Guerra-Assunção JA, Glynn JR, Perdigão J, Viveiros M, et al. A robust SNP barcode for typing Mycobacterium tuberculosis complex strains. Nat Commun. 2014;5:4812.

APPENDICES

APPENDIX A - NIX-TB SPUTUM SPECIMEN TRANSPORT FORM

This form should accompany each sputum specimen generated from a Nix-TB patient at the clinical site to the laboratory. Use a separate Form for each sample submitted. Once the form is completed, a copy is made and sent to the site, whereas the original should remain in the lab files.

**SITE SECTION** *(This section should be completed by the Principal Investigator or delegate)*			
Screening or Patient number	__ __ - __ __ __ __ - __ __ __		
Initials	__ __ __ *(use a "–" if there is no middle initial)*	Date* of birth	__ __/__ __ __/__ __ __ __
Type of Sputum Sample		☐ Early Morning ☐ Spot	
Visit specification	Visit date*	__ __/__ __ __/__ __ __ __	
	Visit in the NiX schedule	☐ Screening ☐ Day 1<br>☐ Treatment, Week __ __ ☐ Unscheduled<br>☐ Post-treatment, Month __ __ ☐ Early Withdraw	
Date* of sputum collection	__ __/__ __ __/__ __ __ __	Time▫ of collection	__ __ :__ __
Physician/nurse attending (print name)			
Physician/nurse attending (signature)			

**TRANSPORT SECTION** *(This section should be completed by* driver, courier or person accompanying sample*)*			
Date* of sample dispatch	__ __/__ __ __/__ __ __ __	Time▫ of dispatch	__ __ :__ __
Temperature of transport container (°C)			
Driver/courier/person accompanying sample (print name)			
Driver/courier/person accompanying sample (signature)			

**LABORATORY SECTION** *(This section should be completed by the laboratory technician receiving the samples)*			
Date* sample received	__ __/__ __ __/__ __ __ __	Time▫ received	__ __ :__ __
Temperature of transport container on receipt (°C)			
Sample in good condition (yes/no)			
If no *please give details (detail problems, is this sample going to be processed? has another sample been requested?)*			
Sample processed within 30 minutes (yes/no)			
If no, time sample transferred to fridge (hh:mm, and give fridge ID)			
Laboratory technician (print name)			
Laboratory technician (signature)			
Laboratory Accession number	ATTACH LABEL		

*Date format dd/mmm/yyyy ▫24h clock

APPENDIX B (PAGE 1)

Screening Number	☐☐-☐☐☐☐
Lab Accession Number	ATTACH LABEL

APPENDIX B: NIX-TB LRF 1 – SCREENING SAMPLES			
Version No			
Date			
Initial			

Patient initials	☐☐☐ *(use a "–" if there is no middle initial)*	
Collection date and time	☐☐ ☐☐☐ ☐☐☐☐ day month year	☐☐:☐☐ Time (24 hour)
Sputum quality	☐Saliva ☐ Mucoid ☐ Mucopurulent ☐ Purulent ☐ Bloody ☐ Mostly Blood	
Estimated specimen volume	☐☐.☐ ml	Tech initials ______________
Specimen processing date and time	☐☐ ☐☐☐ ☐☐☐☐ day month year	☐☐:☐☐ Time (24 hour)
**RESULT SECTION – AURAMINE STAIN OR ZIEHL-NEELSEN**	**☐ Not Done (add comment)**	
Date read	☐☐ ☐☐☐ ☐☐☐☐ day month year	Tech initials ______________
Smear result	☐ No AFB seen ☐ Scanty ☐ 1+ ☐ 2+ ☐ 3+ ☐ Missing (add comment)	
Comments (Include the average number of AFBs per field)		
Lab Supervisor * ______________ *Date & Signature*	Lab Manager ______________ *Date & Signature*	
Date reported	☐☐ ☐☐☐ ☐☐☐☐ day month year	______________ *Signature*

* Verified that the results are transcribed to the LRF correctly

APPENDIX B (PAGE 2)

Screening Number	☐☐-☐☐☐☐
Lab Accession Number	ATTACH LABEL

APPENDIX B: NIX-TB LRF 1 – SCREENING SAMPLES			
Version No			
Date			
Initial			

RESULT SECTION – HAIN MTBDR*plus*	☐ Not Done (add comment)	
Date of DNA extraction	☐☐ ☐☐☐ ☐☐☐☐<br>day month year	Tech initials ______
Date of DNA hybridisation (Result date)	☐☐ ☐☐☐ ☐☐☐☐<br>day month year	Tech initials ______
MTB complex confirmed	yes ☐ no ☐	
Rifampicin banding pattern	☐ rpoB<br>☐ rpoB WT1<br>☐ rpoB WT2<br>☐ rpoB WT3<br>☐ rpoB WT4<br>☐ rpoB WT5<br>☐ rpoB WT6<br>☐ rpoB WT7<br>☐ rpoB WT8<br>☐ rpoB MUT1<br>☐ rpoB MUT2A<br>☐ rpoB MUT2B<br>☐ rpoB MUT3	
Rifampicin resistance interpretation	Sensitive ☐ Resistant ☐ Missing (add comment) ☐	
Isoniazid banding pattern	☐ katG<br>☐ katG WT<br>☐ katG MUT1<br>☐ katG MUT2<br>☐ inhA<br>☐ inhA WT1<br>☐ inhA WT2<br>☐ inhA MUT1<br>☐ inhA MUT2<br>☐ inhA MUT3A<br>☐ inhA MUT3B	
Isoniazid resistance interpretation	Sensitive ☐ Resistant ☐ Missing (add comment) ☐	
Comments		

RESULT SECTION – GENEXPERT MTB/RIF	☐ Not Done (add comment)			
Date of test	☐☐ ☐☐☐ ☐☐☐☐<br>day month year	Is the test valid (probe SPC pass)	yes ☐ no ☐	Tech initials ______
MTB complex confirmed	yes ☐ no ☐	If MTB detected: minimum Cycle threshold (the lowest Ct among probes A-E)	☐☐.☐	
IF MTB detected, note semi-quantitative result	High ☐ low ☐ medium ☐ very low ☐			
Rifampicin resistance interpretation	Sensitive ☐ Resistant ☐ Missing (add comment) ☐			
Comments				

Lab Supervisor * ______________________ Lab Manager ______________________
Date & Signature Date & Signature

Date molecular test result reported	☐☐ ☐☐☐ ☐☐☐☐<br>day month year	______________<br>Signature

* Verified that the results are transcribed to the LRF correctly

APPENDIX B (PAGE 3)

Screening Number	☐☐-☐☐☐☐
Lab Accession Number	ATTACH LABEL

APPENDIX B: NIX-TB LRF 1 – SCREENING SAMPLES			
Version No			
Date			
Initial			

RESULT SECTION – MGIT CULTURE ☐ Not Done (add comment)

Date / time protocol started	☐☐ ☐☐☐ ☐☐☐☐ (day, month, year)	☐☐:☐☐ Time	Tech initials ________
MGIT tube No.	☐☐ ☐☐ ☐☐☐☐☐☐☐☐		
MGIT result	☐ pos. ☐ neg.	☐☐ ☐☐☐ ☐☐☐☐ (day, month, year) ☐☐:☐☐ Time	Time to detection (TTD/TTP): ☐☐.☐☐ (Days, Hours) Tech initials ____________

If instrument positive: **Blood Agar Plate (BAP) (after 48 h):**

Date Read	☐☐ ☐☐☐ ☐☐☐☐ (day, month, year)	Result	☐ pos. ☐ neg. ☐ other (add comment)
Comment: Blood Agar Result Other	☐ BAP not done<br>☐ BAP is negative, MGIT tube visibly contaminated<br>☐ BAP is negative, evidence of fungal hyphae seen on ZN smear		Tech initials ____________

If instrument positive: **Ziehl-Neelsen stain:**

Date read	☐☐ ☐☐☐ ☐☐☐☐ (day, month, year)	Result	☐ pos. ☐ neg. ☐ missing (add comment)	☐ cording ☐ typical ☐ atypical
Comments			Tech initials ____________	

Lab Supervisor * ____________________ Lab Manager ____________________
Date & Signature *Date & Signature*

Date MGIT results reported	☐☐ ☐☐☐ ☐☐☐☐ (day, month, year)	____________ *Signature*

RESULT SECTION – ISOLATE STORAGE AND SHIPMENT

Was the subculture prepared for storage and shipment?	yes ☐ no ☐ not applicable ☐
Was a LJ slope shipped to the Central Lab for characterization?	yes ☐ no ☐ not applicable ☐
Comments	

Lab Supervisor * ____________________
Date & Signature

* Verified that the results are transcribed to the LRF correctly

APPENDIX C (PAGE 1)

Subject Number	☐☐-☐☐☐☐-☐☐☐
Lab Accession Number	ATTACH LABEL

APPENDIX C: **NIX-TB LRF 2 – TREATMENT AND FOLLOW-UP**			
Version No			
Date			
Initial			

Patient initials	☐☐☐ (use ' – ' if there is no middle initial)		
Specimen Type	☐ Early Morning ☐ Spot		
Collection date and time	☐☐ day ☐☐☐ month ☐☐☐☐ year	☐☐:☐☐ Time (24 hour)	
Visit specification	☐ Day 1 ☐ Treatment, ☐☐ Week ☐ Early withdraw<br>☐ Post-treatment, ☐☐ Month ☐ Unscheduled		
Sputum quality	☐ Saliva ☐ Mucoid ☐ Mucopurulent ☐ Purulent ☐ Bloody ☐ Mostly Blood		
Estimated specimen volume	☐☐.☐ ml	Tech initials ___________	
Processed date and time	☐☐ day ☐☐☐ month ☐☐☐☐ year	☐☐:☐☐ Time (24 hour)	
**RESULT SECTION – MGIT CULTURE**	**☐ Not Done (add comment)**		
Date / time protocol started	☐☐ day ☐☐☐ month ☐☐☐☐ year	☐☐:☐☐ Time	Tech initials ___________
MGIT tube No.	☐☐ ☐☐ ☐☐☐☐☐☐☐☐		
MGIT result	☐ pos.<br>☐ neg.	☐☐ day ☐☐☐ month ☐☐☐☐ year<br>☐☐:☐☐ Time	Time to detection (TTD/TTP): ☐☐ Days . ☐☐ Hours<br>Tech initials ___________
If instrument positive: **Blood Agar Plate (BAP) (after 48 h):**			
Date Read	☐☐ day ☐☐☐ month ☐☐☐☐ year	Result	☐ pos. ☐ neg. ☐ other (add comment)
Comment: Blood Agar Result Other	☐ BAP not done<br>☐ BAP is negative, MGIT tube visibly contaminated<br>☐ BAP is negative, evidence of fungal hyphae seen on ZN smear	Tech initials ___________	

APPENDIX C (PAGE 2)

Subject Number	☐☐-☐☐☐☐-☐☐☐
Lab Accession Number	ATTACH LABEL

APPENDIX C: **NIX-TB LRF 2 – TREATMENT AND FOLLOW-UP**			
Version No			
Date			
Initial			

If instrument positive: **Ziehl-Neelsen stain:**

Date read	☐☐ ☐☐☐ ☐☐☐☐<br>day month year	Result	☐ pos. ☐ neg.<br>☐ missing (add comment)	☐ cording ☐ typical<br>☐ atypical
Comments		Tech initials ______________		

Lab Supervisor * ________________________ Lab Manager ________________________
Date & Signature *Date & Signature*

Date MGIT results reported	☐☐ ☐☐☐ ☐☐☐☐<br>day month year	________________________<br>*Signature*

RESULT SECTION - ISOLATE STORAGE AND SHIPMENT

Was the sulture prepapared for storage and shipment?	yes ☐ no ☐ not applicable ☐
Was a LJ slope shipped to the Central Lab for characterization?	yes ☐ no ☐ not applicable ☐
Comments	

Lab Supervisor * ________________________
Date & Signature

*Verified that the results are transcribed to the LRF correctly

APPENDIX D

Screening or Subject Number	☐☐-☐☐☐☐-☐☐☐
Lab Accession Number	ATTACH LABEL

APPENDIX D: NIX-TB LRF 3 – SPECIATION			
Version No			
Date			
Initial			

Patient initials	☐☐☐ (use ' – ' if there is no middle initial)	
Specimen Type	☐ Early Morning ☐ Spot	
Collection date and time	☐☐ ☐☐☐ ☐☐☐☐ day month year	☐☐:☐☐ Time (24 hour)
Visit specification	☐ Screening ☐ Day 1 ☐ Treatment, ☐☐ Week<br>☐ Post-treatment, ☐☐ Month ☐ Unscheduled ☐ Early withdraw	
**RESULT SECTION – SPECIATION** ☐ **Not Done (add comment)**		
Date of test	☐☐ ☐☐☐ ☐☐☐☐ day month year	Tech initials ______
Test type	**MPT64 Antigen test** ☐ Hain **Mycobacterium CM** ☐<br>Hain **MTBC** ☐ Hain MTBDR***plus*** ☐<br>Hain MTBDR***sl*** ☐ GeneXpert ☐	
MTB complex confirmed	yes ☐ no ☐ Missing ☐ (add comment)	
Comments		
Result date	☐☐ ☐☐☐ ☐☐☐☐ day month year	Tech initials ______
Lab Supervisor * ______ *Date & Signature*	Lab Manager ______ *Date & Signature*	
Date Reported	☐☐ ☐☐☐ ☐☐☐☐ day month year	______ *Signature*

* Verified that the results are transcribed to the LRF correctly

APPENDIX E: Isolate Shipment to UCL

This form should be completed each time isolates are to be shipped to UCL. A copy of this form should be sent with the LJ slopes.

<table>
<tr><td colspan="4">Laboratory Name:
Shipment Date:
Total number of Slopes:</td><td>Name of requestor:

Signature of requestor:</td></tr>
<tr><th>Patient Number</th><th>Laboratory Accession Number</th><th>Visit Schedule</th><th>Specimen Type</th><th>Comment</th></tr>
<tr><td></td><td></td><td></td><td></td><td></td></tr>
<tr><td></td><td></td><td></td><td></td><td></td></tr>
<tr><td></td><td></td><td></td><td></td><td></td></tr>
<tr><td></td><td></td><td></td><td></td><td></td></tr>
<tr><td></td><td></td><td></td><td></td><td></td></tr>
<tr><td></td><td></td><td></td><td></td><td></td></tr>
<tr><td></td><td></td><td></td><td></td><td></td></tr>
<tr><td></td><td></td><td></td><td></td><td></td></tr>
<tr><td></td><td></td><td></td><td></td><td></td></tr>
<tr><td></td><td></td><td></td><td></td><td></td></tr>
<tr><td></td><td></td><td></td><td></td><td></td></tr>
<tr><td></td><td></td><td></td><td></td><td></td></tr>
<tr><td></td><td></td><td></td><td></td><td></td></tr>
<tr><td colspan="4">To be completed upon reconciliation

Date received:
UCL Staff Name:
UCL Staff Signature:</td><td>Additional comments:</td></tr>
</table>

APPENDIX F (PAGE 1)

APPENDIX F
NIX-TB LRF 4 – Drug Susceptibility Testing

Version No			
Date			
Initial			

Laboratory Accession Number			
Patient number	☐☐-☐☐☐☐-☐☐☐		
Visit specification	☐ Day 1 ☐ Treatment, ☐☐ Week ☐ Post-treatment, ☐☐ Month ☐ Screening ☐ Unscheduled		
**RESULT SECTION**			
**Blood Agar Plate** Date of evaluation	☐☐ day ☐☐☐ month ☐☐☐☐ year	Result ☐ pos. ☐ neg Initial ______	
**SIRE** Date of test / started	☐☐ day ☐☐☐ month ☐☐☐☐ year	Initial _____	Used culture ☐ MGIT ☐ LJ
Streptomycin	☐ sensitive ☐ resistant ☐ missing (add comment)		
Isoniazid	☐ sensitive ☐ resistant ☐ missing (add comment)		
Rifampicin	☐ sensitive ☐ resistant ☐ missing (add comment)		
Ethambutol	☐ sensitive ☐ resistant ☐ missing (add comment)		
Date SIRE removed, or EpiCenter Report generated	day ☐☐ month ☐☐☐ year ☐☐☐☐	☐ Epicenter used	
**KAN** Date of test / started	☐☐ day ☐☐☐ month ☐☐☐☐ year	Initial _____	Used culture ☐ MGIT ☐ LJ
Kanamycin	☐ sensitive ☐ resistant ☐ missing (add comment)		
Date KAN removed, or EpiCenter Report generated	day ☐☐ month ☐☐☐ year ☐☐☐☐	☐ Epicenter used	
**PZA** Date of test / started	☐☐ day ☐☐☐ month ☐☐☐☐ year	Initial _____	Used culture ☐ MGIT ☐ LJ
Pyrazinamide	☐ sensitive ☐ resistant ☐ missing (add comment)		
Date PZA removed, or EpiCenter Report generated	day ☐☐ month ☐☐☐ year ☐☐☐☐	☐ Epicenter used	
**MOX** Date of test / started	☐☐ day ☐☐☐ month ☐☐☐☐ year	Initial _____	Used culture ☐ MGIT ☐ LJ
Moxifloxacin concentration	☐ 0.5µg/ml ☐ 2µg/ml ☐ Other (please specify)		
Moxifloxacin	☐ sensitive ☐ resistant ☐ missing (add comment)		
Date Mox removed, or EpiCenter Report generated	day ☐☐ month ☐☐☐ year ☐☐☐☐	☐ Epicenter used	
Comments			
Lab Supervisor * ______ Date & Signature	Lab Manager ______ Date & Signature		
Date Reported	☐☐ day ☐☐☐ month ☐☐☐☐ year	______ Signature	

* Verified that the results are transcribed to the LRF correctly

APPENDIX F (PAGE 2)

APPENDIX F
NIX-TB LRF 4 – Drug Susceptibility Testing

Version No			
Date			
Initial			

Fill out in case of resistant result:

Drug: ____ Start Date: ____ Initials: ____	Blood agar plate sterile	☐ yes ☐ contaminated	____ Initials/Date Read
	Visible inspection of MGIT tube – Normal TB Morphology seen	☐ yes ☐ no	____ Initials/Date Read
	Confirmation by ZN-stain (if applicable)	☐ AFB+ ☐ contamination ☐ No AFB	____ Initials/Date Read
Drug: ____ Start Date: ____ Initials: ____	Blood agar plate sterile	☐ yes ☐ contaminated	____ Initials/Date Read
	Visible inspection of MGIT tube – Normal TB Morphology seen	☐ yes ☐ no	____ Initials/Date Read
	Confirmation by ZN-stain (if applicable)	☐ AFB+ ☐ contamination ☐ No AFB	____ Initials/Date Read
Drug: ____ Start Date: ____ Initials: ____	Blood agar plate sterile	☐ yes ☐ contaminated	____ Initials/Date Read
	Visible inspection of MGIT tube – Normal TB Morphology seen	☐ yes ☐ no	____ Initials/Date Read
	Confirmation by ZN-stain (if applicable)	☐ AFB+ ☐ contamination ☐ No AFB	____ Initials/Date Read
Drug: ____ Start Date: ____ Initials: ____	Blood agar plate sterile	☐ yes ☐ contaminated	____ Initials/Date Read
	Visible inspection of MGIT tube – Normal TB Morphology seen	☐ yes ☐ no	____ Initials/Date Read
	Confirmation by ZN-stain (if applicable)	☐ AFB+ ☐ contamination ☐ No AFB	____ Initials/Date Read
Drug: ____ Start Date: ____ Initials: ____	Blood agar plate sterile	☐ yes ☐ contaminated	____ Initials/Date Read
	Visible inspection of MGIT tube – Normal TB Morphology seen	☐ yes ☐ no	____ Initials/Date Read
	Confirmation by ZN-stain (if applicable)	☐ AFB+ ☐ contamination ☐ No AFB	____ Initials/Date Read
Lab. Manager (Date and Signature):			

APPENDIX G (PAGE 1)

APPENDIX G

NIX-TB LRF 5 – Minimal Inhibitory Concentration

Version No			
Date			
Initial			

Laboratory Accession Number			
Patient number	☐☐-☐☐☐☐-☐☐☐		
Visit specification (Tick and complete as appropriate)	☐ Day 1 ☐ Treatment, ☐☐ Week ☐ Post-treatment, ☐☐ Month ☐ Screening ☐ Unscheduled		
**RESULT SECTION**			
**Blood agar plate** Date of evaluation	☐☐ day ☐☐☐ month ☐☐☐☐ year	**Result:** ☐ pos. ☐ neg Initials ________	
**BDQ** Test Start Date	☐☐ day ☐☐☐ month ☐☐☐☐ year	Initials ___________	
**BDQ** MIC value	______ µg/ml or Missing ☐ Provide reason…………………………………		
**BDQ** Result Date	☐☐ day ☐☐☐ month ☐☐☐☐ year	Initials ___________	
**LZD** Test Start Date	☐☐ day ☐☐☐ month ☐☐☐☐ year	Initials ___________	
**LZD** MIC value	______ µg/ml or Missing ☐ Provide reason…………………………………		
**LZD** Result Date	☐☐ day ☐☐☐ month ☐☐☐☐ year	Initials ___________	
**PRE** Test Start Date	☐☐ day ☐☐☐ month ☐☐☐☐ year	Initials ___________	
**PRE** MIC value	______ µg/ml or Missing ☐ Provide reason…………………………………		
**PRE** Result Date	☐☐ day ☐☐☐ month ☐☐☐☐ year	Initials ___________	
Comments			

Lab Supervisor * ______________________ *Date & Signature* Lab Manager ______________________ *Date & Signature*

Date Reported	☐☐ day ☐☐☐ month ☐☐☐☐ year	______________ Signature

* Verified that the results are transcribed to the LRF correctly

APPENDIX G (PAGE 2)

APPENDIX G

Version No			
Date			
Initial			

NIX-TB LRF 5 – Minimal Inhibitory Concentration

Fill out in case of resistant result:

Drug: ____<br>Conc (µg/ml): ____<br>Start Date: ____<br>Initials: ____	Blood agar plate sterile	☐ yes ☐ contaminated	____ Initials/Date Read
	Confirmation by ZN-stain	☐ AFB+ ☐ contaminated<br>☐ No AFB	____ Initials/Date Read
Drug: ____<br>Conc (µg/ml): ____<br>Start Date: ____<br>Initials: ____	Blood agar plate sterile	☐ yes ☐ contaminated	____ Initials/Date Read
	Confirmation by ZN-stain	☐ AFB+ ☐ contaminated<br>☐ No AFB	____ Initials/Date Read
Drug: ____<br>Conc (µg/ml): ____<br>Start Date: ____<br>Initials: ____	Blood agar plate sterile	☐ yes ☐ contaminated	____ Initials/Date Read
	Confirmation by ZN-stain	☐ AFB+ ☐ contaminated<br>☐ No AFB	____ Initials/Date Read
Drug: ____<br>Conc (µg/ml): ____<br>Start Date: ____<br>Initials: ____	Blood agar plate sterile	☐ yes ☐ contaminated	____ Initials/Date Read
	Confirmation by ZN-stain	☐ AFB+ ☐ contaminated<br>☐ No AFB	____ Initials/Date Read
Drug: ____<br>Conc (µg/ml): ____<br>Start Date: ____<br>Initials: ____	Blood agar plate sterile	☐ yes ☐ contaminated	____ Initials/Date Read
	Confirmation by ZN-stain	☐ AFB+ ☐ contaminated<br>☐ No AFB	____ Initials/Date Read
Drug: ____<br>Conc (µg/ml): ____<br>Start Date: ____<br>Initials: ____	Blood agar plate sterile	☐ yes ☐ contaminated	____ Initials/Date Read
	Confirmation by ZN-stain	☐ AFB+ ☐ contaminated<br>☐ No AFB	____ Initials/Date Read
Lab. Manager (Date & Signature):____			

APPENDIX H

APPENDIX H	Version No			
	Date			
**NIX-TB LRF 6** – Paired WGS	Initial			

Patient number	☐☐-☐☐☐☐-☐☐☐	
**Isolate 1**	Visit specification (tick and complete as appropriate)	☐ Screening ☐ Day 1 ☐ Treatment, ☐☐ Week
	Laboratory Accession Number	
	Collection date	☐☐ ☐☐☐ ☐☐☐☐<br>day month year
**Isolate 2**	Visit specification (tick and complete as appropriate)	☐ Treatment, ☐☐ Week ☐ Post-Treatment, ☐☐ Month<br>☐ Unscheduled
	Laboratory Accession Number	
	Collection date	☐☐ ☐☐☐ ☐☐☐☐<br>day month year

PAIRED WHOLE GENOME SEQUENCING	
Was paired whole genome sequencing performed?	☐ Yes ☐ No (add comment)
Sequence Run date*	**Isolate 1** ☐☐ ☐☐☐ ☐☐☐☐<br>day month year<br>**Isolate 2** ☐☐ ☐☐☐ ☐☐☐☐<br>day month year
Number of Single Nucleotide Polymorphisms (SNP) differences between isolate 1 and isolate 2*<br>Note: For pairs with <20 SNPs different, please print the associated annotated SNP table and attach to this LRF	
Comments	
Result date<br>(Note: this is the date of the Annotated SNP List Report comparing the two isolates)	☐☐ ☐☐☐ ☐☐☐☐<br>day month year

Lab Supervisor * ______________________ Lab Manager ______________________
Date & Signature *Date & Signature*

* Verified that the results are transcribed to the LRF correctly

APPENDIX I (PAGE 1)

APPENDIX I

DNA QUANTIFICATION WORKSHEET

Lab accession number	Patient ID	Visit	Date of DNA Extraction (if UCL or add 'site')	Read Date	Initials	Nanodrop		Read Date	Initials	Qubit®			Acceptable (y/n)
						260/280 Ratio	Concentration (ng/µl)			Sample Volume (1-20µl)	dsDNA conc in Qubit tube Add UNITS: ____________	Final dsDNA conc (µg/ml) in DNA sample	

Lab Manager (Sign and Date): ____

APPENDIX I (PAGE 2)

APPENDIX I

DNA QUANTIFICATION WORKSHEET

If DNA concentration is acceptable and DNA requires dilution prior to sending for WGS, dilution steps should be documented below.

NOTE: Note this table is only required to be completed if DNA is sent for WGS

Lab accession number	Patient ID	QUBIT Final dsDNA conc (µg/ml) in DNA sample	Final DNA concentration (µg/ml) required for WGS	Dilution Factor (add details)	Final volume of sample sent for WGS (µl)

Lab Manager (Sign and Date): ______________________

Appendix 1 -- Hain Life Sciences, GenoLyse ®, Version 1.0, Instructions for Use, 10/2012

GenoLyse_1012_5161
0-09-02.pdf

Appendix 2 – Xpert *M.tb*/RIF System Operator Manual

XpertMTB_Broch_R9_
EU.pdf

Appendix 3 – Hain GenoType MTBDR*plus*, Version 2.0, Instructions for Use, 10/2011

MTBDRplusV2_1011_
304A-01-02.pdf

Appendix 4 – Hain GenoType MTBDR*sl*, Version 2.0, Instructions for Use, 06/2015

MTBDRslV2_0615_317
A-02-02.pdf

Appendix 5 – Hain GenoType MTBC, Version 1.0, Instructions for Use, 06/2015

GenoType MTBC
(IFU-301-10).pdf

Appendix 6 – Hain GenoType Mycobacterium CM, Version 1.0, Instructions for Use, 08/2016

MYCCMV2_0816_299
A-01-02.pdf

Appendix 7 – BW0303: Instructions on sample submission and receiving results

Adobe Acrobat
Document
