# Supplementary material for: An Exposure-Response Perspective on the Clinical Dose of Pretomanid
Source: Antimicrob Agents Chemother. 2020 Dec 16;65(1):e01121-20. doi: 10.1128/AAC.01121-20 (PMC7927811; doi:10.1128/AAC.01121-20)
Supplement: Supplemental file 1 [file AAC.01121-20-s0001.pdf]

The Clinical Dose of Pretomanid: An Exposure-Response Perspective Supplementary Material

Authors:
Jerry R. Nedelman – TB Alliance, New York, NY

David H. Salinger - Certara Inc., Princeton, NJ; under contract with the Bill and Melinda Gates Foundation

Vishak Subramoney - Certara Strategic Consulting, Canada; under contract with the Bill and Melinda Gates Foundation

Rob Woolson – Rho, Raleigh, NC

Karen Wade – Rho, Raleigh, NC

Mengchun Li – TB Alliance, New York, NY

Daniel Everitt – TB Alliance, New York, NY

Carl M. Mendel – TB Alliance, New York, NY

Mel Spigelman – TB Alliance, New York, NY

S0. Data summaries

Patients by regimen and study

```
##
##          NC-002 NC-005 Nix-TB STAND
##    BPaL        0      0    109     0
##    BPaMZ       0     55      0     0
##    BPaZ        0    119      0     0
##    PaMZ      147      0      0   214
```

Summary statistics of covariates by regimen

```
## $BPaL
##   Cavg (ug/mL)        Age (yr)     Baseline CFU (log10)  BMI (kg/m^2)   Baseline TTP (hr)
##  Min.   : 0.8205   Min.   :17.00   Min.   : NA          Min.   :12.40   Min.   : 57.0
##  1st Qu.: 1.7430   1st Qu.:28.00   1st Qu.: NA          1st Qu.:17.40   1st Qu.:184.0
##  Median : 2.5570   Median :35.00   Median : NA          Median :19.70   Median :278.5
##  Mean   : 2.7955   Mean   :35.61   Mean   :NaN          Mean   :20.64   Mean   :329.8
##  3rd Qu.: 3.4400   3rd Qu.:43.00   3rd Qu.: NA          3rd Qu.:23.10   3rd Qu.:458.4
##  Max.   :10.2300   Max.   :60.00   Max.   : NA          Max.   :41.10   Max.   :869.0
##                                    NA's   :109                          NA's   :21
##
## $BPaMZ
##   Cavg (ug/mL)      Age (yr)     Baseline CFU (log10)  BMI (kg/m^2)   Baseline TTP (hr)
##  Min.   :0.893   Min.   :18.00   Min.   :1.000        Min.   :12.22   Min.   : 65.45
##  1st Qu.:1.820   1st Qu.:23.00   1st Qu.:4.973        1st Qu.:16.33   1st Qu.: 87.07
##  Median :2.475   Median :34.00   Median :5.951        Median :18.44   Median :114.25
##  Mean   :2.402   Mean   :34.38   Mean   :5.549        Mean   :18.43   Mean   :122.37
##  3rd Qu.:2.892   3rd Qu.:43.50   3rd Qu.:6.500        3rd Qu.:19.97   3rd Qu.:148.02
##  Max.   :4.573   Max.   :69.00   Max.   :7.769        Max.   :27.05   Max.   :246.90
##
## $BPaZ
##   Cavg (ug/mL)       Age (yr)     Baseline CFU (log10)  BMI (kg/m^2)   Baseline TTP (hr)
##  Min.   :0.9329   Min.   :18.00   Min.   :1.220        Min.   :15.15   Min.   : 48.64
##  1st Qu.:1.8810   1st Qu.:25.00   1st Qu.:5.137        1st Qu.:17.56   1st Qu.: 81.74
##  Median :2.3720   Median :32.00   Median :5.911        Median :19.27   Median : 92.90
##  Mean   :2.4776   Mean   :34.51   Mean   :5.691        Mean   :19.72   Mean   :103.14
##  3rd Qu.:2.8390   3rd Qu.:41.50   3rd Qu.:6.467        3rd Qu.:20.54   3rd Qu.:109.90
##  Max.   :5.1550   Max.   :69.00   Max.   :7.602        Max.   :32.55   Max.   :323.78
##                                   NA's   :1                            NA's   :1
##
```

```
## $PaMZ
##   Cavg (ug/mL)      Age (yr)     Baseline CFU (log10)  BMI (kg/m^2)   Baseline TTP (hr)
##  Min.   :0.436   Min.   :18.00   Min.   :1.000        Min.   :13.35   Min.   : 40.00
##  1st Qu.:1.738   1st Qu.:25.00   1st Qu.:4.779        1st Qu.:17.48   1st Qu.: 90.96
##  Median :2.422   Median :32.00   Median :5.669        Median :19.05   Median :113.50
##  Mean   :2.776   Mean   :33.87   Mean   :5.502        Mean   :19.91   Mean   :132.60
##  3rd Qu.:3.637   3rd Qu.:41.00   3rd Qu.:6.506        3rd Qu.:21.30   3rd Qu.:153.00
##  Max.   :8.697   Max.   :77.00   Max.   :7.689        Max.   :43.25   Max.   :794.50
##                                  NA's   :218                          NA's   :5

##       Regimen
## Type   BPaL BPaMZ BPaZ PaMZ
##   1XDR   69     0    0    0
##   2MDR   40    55    0   38
##   3DS     0     0  119  323

##       Regimen
## Female BPaL BPaMZ BPaZ PaMZ
##      0   57    39   93  242
##      1   52    16   26  119

##    Regimen
## HIV BPaL BPaMZ BPaZ PaMZ
##   0   53    31  101  277
##   1   56    24   18   84

##           Regimen
## Cavitation BPaL BPaMZ BPaZ PaMZ
##       0      17     4   24   48
##       1      92    51   95  307
##       <NA>    0     0    0    6
```

S1. Time to Sputum Culture Conversion

S1.1 Events by study and regimen

```
## # A tibble: 6 x 4
## # Groups:   Study [5]
##   Study       Regimen Events Censored
##   <chr>       <chr>    <int>    <int>
## 1 NC-002      "PaMZ"      64       81
## 2 NC-005      "BPaMZ"     45        9
## 3 NC-005      "BPaZ"      73       43
## 4 Nix-TB      "BPaL"      86        4
## 5 STAND       "PaMZ"     186       28
## 6 .....Total ""          454      165
```

S1.2 Step 1 Initial screen for evidence of an E-R relationship

Kaplan-Meier plots by Cavg quantile

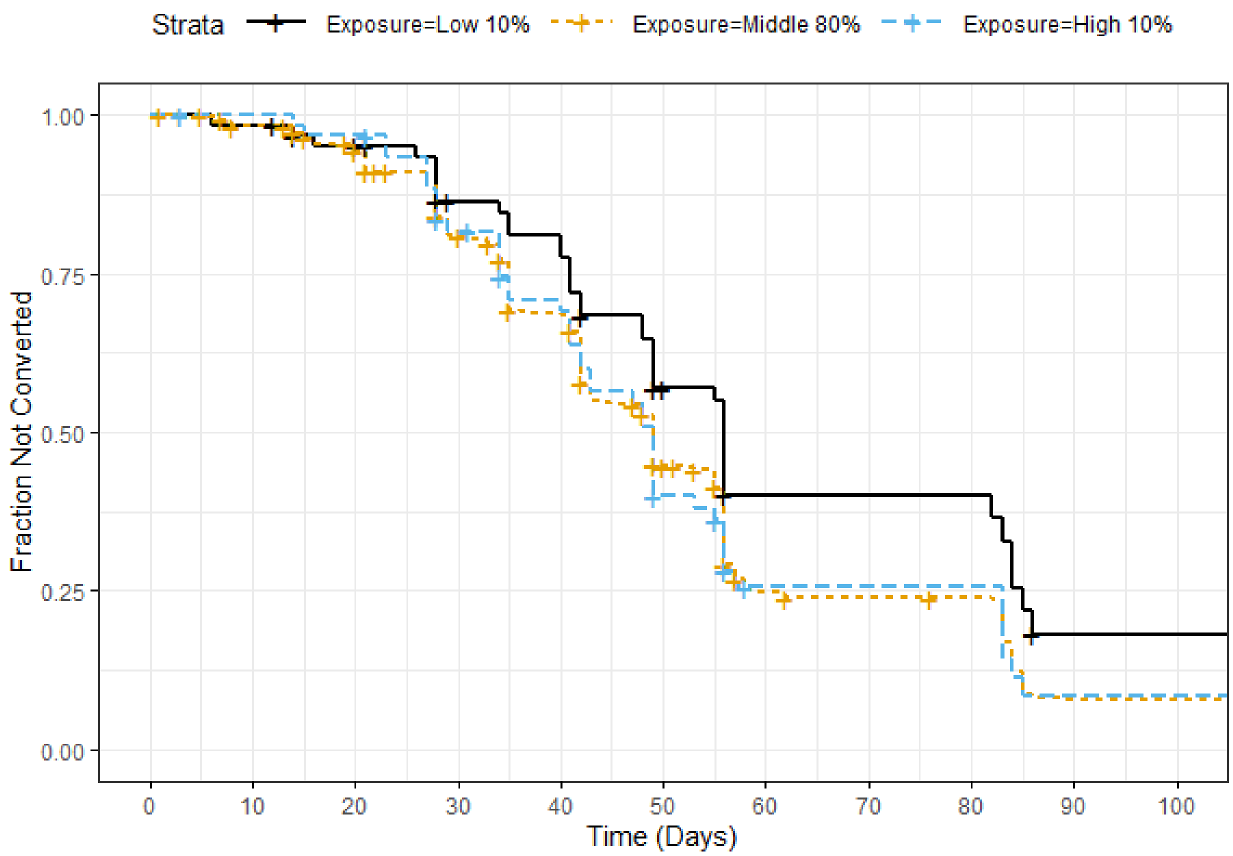

Cox model with regimen and log-transfored Cavg

```
## Call:
## coxph(formula = Surv(time, status) ~ Regimen + dLCavg, data = data2)
##
##                    coef exp(coef) se(coef)      z        p
## RegimenBPaMZ  0.13707   1.14691  0.18703  0.733 0.463627
## RegimenBPaZ  -0.26429   0.76775  0.16224 -1.629 0.103321
## RegimenPaMZ  -0.41688   0.65910  0.12585 -3.313 0.000924
## dLCavg        0.27669   1.31875  0.09912  2.791 0.005247
##
## Likelihood ratio test=24.35  on 4 df, p=6.804e-05
## n= 619, number of events= 454
```

Cavg versus conversion by 8 weeks:

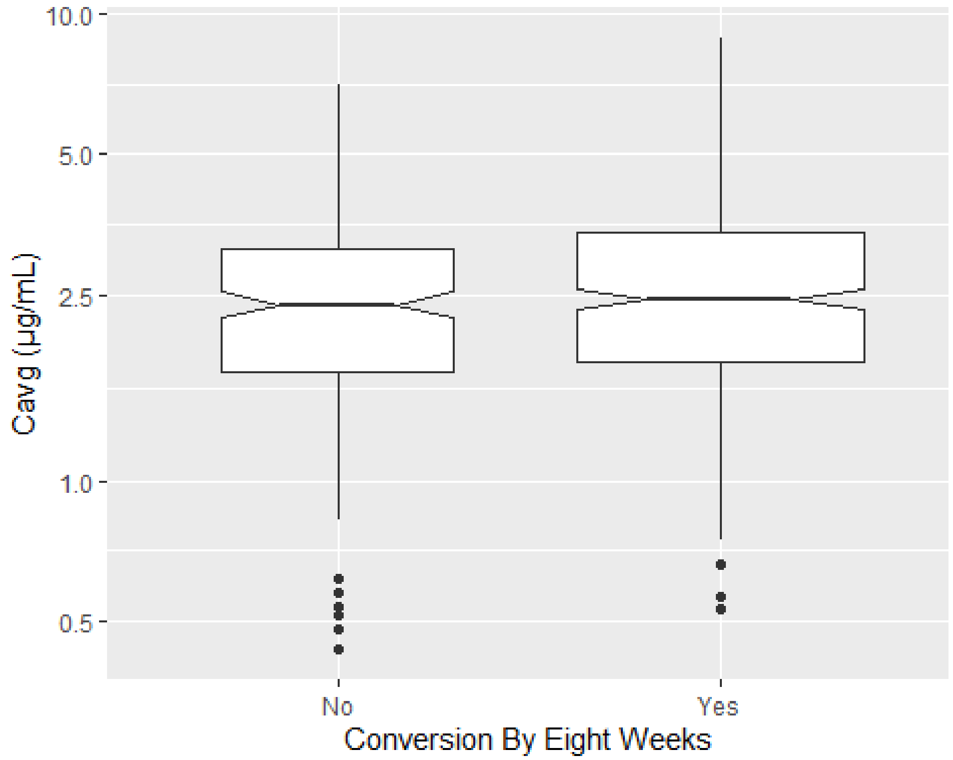

Log-transformed Cavg vs conversion by 8 weeks and Regimen:

```
##
## Call:
## lm(formula = dLCavg ~ Conversion + Regimen, data = ForBox)
##
## Residuals:
##      Min       1Q   Median       3Q      Max
## -1.68128 -0.31118  0.01766  0.32635  1.27033
##
## Coefficients:
##                Estimate Std. Error t value Pr(>|t|)
## (Intercept)   -0.101486   0.057234  -1.773   0.0767 .
```

```
## ConversionYes  0.057417   0.039968   1.437   0.1514
## RegimenBPaMZ  -0.077290   0.082565  -0.936   0.3496
## RegimenBPaZ   -0.039242   0.067214  -0.584   0.5595
## RegimenPaMZ   -0.002862   0.056629  -0.051   0.9597
## ---
## Signif. codes:  0 '***' 0.001 '**' 0.01 '*' 0.05 '.' 0.1 ' ' 1
##
## Residual standard error: 0.4783 on 614 degrees of freedom
## Multiple R-squared:  0.005125,   Adjusted R-squared:  -0.001357
## F-statistic: 0.7907 on 4 and 614 DF,  p-value: 0.5315
```

The Kaplan-Meier plots and the Cox model show some evidence of a relationship between exposure and outcome, so subsequent steps are undertaken.

S1.3 Step 2 Covariate selection for covariates with no missing values

Cavitation and baseline TTP have missing values, which will necessitate reducing the dataset. First examine age, BMI, gender, HIV status, and TB type, which have no missing values.

Regimen and a 4-df spline of centered, log-transformed Cavg (dLCavg) are included in all models.

Age and BMI are also centered and log-transformed to dLAge and dLBMI.

Model 1: dLAge, dLBMI, FEMALE, HIV, Type: No missing values

```
## Call:
## coxph(formula = Surv(time, status) ~ Regimen + dLAge + dLBMI +
##     FEMALE + HIV + Type + X1 + X2 + X3 + X4, data = data1)
##
##                   coef exp(coef) se(coef)      z       p
## RegimenBPaMZ  0.17465   1.19083  0.24255  0.720 0.47150
## RegimenBPaZ   0.03819   1.03893  0.33029  0.116 0.90794
## RegimenPaMZ  -0.16106   0.85124  0.29439 -0.547 0.58430
## dLAge        -0.44746   0.63925  0.15446 -2.897 0.00377
## dLBMI         0.28696   1.33237  0.27853  1.030 0.30289
## FEMALE        0.13734   1.14721  0.11144  1.232 0.21783
## HIV          -0.09253   0.91162  0.11745 -0.788 0.43081
## Type2MDR     -0.01232   0.98776  0.22833 -0.054 0.95698
## Type3DS      -0.27504   0.75954  0.33321 -0.825 0.40912
## X1            1.29337   3.64505  0.54166  2.388 0.01695
## X2            0.64622   1.90832  0.39455  1.638 0.10145
## X3            3.05822  21.28965  1.18518  2.580 0.00987
## X4            1.24825   3.48423  0.49012  2.547 0.01087
##
## Likelihood ratio test=45.56  on 13 df, p=1.689e-05
## n= 619, number of events= 454
```

BMI, FEMALE, HIV, and Type are not significant; Age is significant.

Model 2: dLAge, then ANOVA vs Model 1

```
## Call:
## coxph(formula = Surv(time, status) ~ Regimen + dLAge + X1 + X2 +
##     X3 + X4, data = data1)
##
##                   coef exp(coef) se(coef)      z        p
## RegimenBPaMZ  0.1141    1.1209   0.1885  0.605 0.544876
## RegimenBPaZ  -0.2622    0.7694   0.1638 -1.601 0.109453
## RegimenPaMZ  -0.4223    0.6556   0.1279 -3.301 0.000964
## dLAge        -0.4907    0.6122   0.1479 -3.318 0.000906
## X1            1.3245    3.7603   0.5367  2.468 0.013589
## X2            0.6638    1.9421   0.3867  1.716 0.086079
## X3            3.1187   22.6170   1.1838  2.635 0.008425
## X4            1.3060    3.6913   0.4822  2.709 0.006756
##
## Likelihood ratio test=40.72  on 8 df, p=2.347e-06
## n= 619, number of events= 454
```

```
## Analysis of Deviance Table
##  Cox model: response is  Surv(time, status)
##  Model 1: ~ Regimen + dLAge + dLBMI + FEMALE + HIV + Type + X1 + X2 + X3 +
X4
##  Model 2: ~ Regimen + dLAge + X1 + X2 + X3 + X4
##    loglik  Chisq Df P(>|Chi|)
## 1 -2460.9
## 2 -2463.3 4.8322  5    0.4367
```

This confirms the non-significance of BMI, FEMALE, and HIV.

Test proportional hazards in Model 2

```
##             chisq df     p
## Regimen  6.58900  3 0.086
## dLAge    3.30444  1 0.069
## X1       0.00861  1 0.926
## X2       4.38178  1 0.036
## X3       2.36217  1 0.124
## X4       3.89534  1 0.048
## GLOBAL  13.66176  8 0.091
```

There is some evidence of violation for a couple spline terms, but the global test has p>0.05. Defer remediation on the exposure terms unless and until the problem persists in later steps.

S1.4 Step 3 Covariate interactions

Before moving on to consider the covariates with missing values, assess possible interactions among age, regimen, and exposure, using the full dataset.

Model 3: Test interaction of regimen and exposure

```
## Call:
## coxph(formula = Surv(time, status) ~ Regimen + dLAge + X1 + X2 +
##     X3 + X4 + Regimen:X1 + Regimen:X2 + Regimen:X3 + Regimen:X4,
##     data = data1)
##
##                      coef  exp(coef)   se(coef)      z        p
## RegimenBPaMZ      3.108141  22.379410   4.417828  0.704 0.481716
## RegimenBPaZ      -2.164704   0.114784   5.506398 -0.393 0.694227
## RegimenPaMZ       0.594339   1.811833   2.427124  0.245 0.806554
## dLAge            -0.503403   0.604470   0.150713 -3.340 0.000837
## X1                2.557303  12.900972   2.272185  1.125 0.260385
## X2                0.612641   1.845299   1.464167  0.418 0.675638
## X3                5.851696 347.823745   4.872802  1.201 0.229794
## X4                4.250098  70.112265   1.396569  3.043 0.002340
## RegimenBPaMZ:X1  -3.243871   0.039013   4.200463 -0.772 0.439957
## RegimenBPaZ:X1    1.775265   5.901845   5.321400  0.334 0.738675
## RegimenPaMZ:X1   -1.394858   0.247868   2.340264 -0.596 0.551158
## RegimenBPaMZ:X2  -1.150608   0.316444   3.035454 -0.379 0.704646
## RegimenBPaZ:X2    1.507904   4.517254   3.507202  0.430 0.667236
## RegimenPaMZ:X2    0.179011   1.196034   1.523931  0.117 0.906490
## RegimenBPaMZ:X3  -6.055567   0.002345   9.140599 -0.662 0.507656
## RegimenBPaZ:X3    2.849726  17.283042  11.029305  0.258 0.796115
## RegimenPaMZ:X3   -2.774690   0.062369   5.034159 -0.551 0.581515
## RegimenBPaMZ:X4  -2.438283   0.087311   5.718092 -0.426 0.669805
## RegimenBPaZ:X4   -1.208474   0.298653   4.249222 -0.284 0.776105
## RegimenPaMZ:X4   -3.347031   0.035189   1.497017 -2.236 0.025365
##
## Likelihood ratio test=46.91  on 20 df, p=0.0006031
## n= 619, number of events= 454

## Analysis of Deviance Table
##  Cox model: response is  Surv(time, status)
##  Model 1: ~ Regimen + dLAge + X1 + X2 + X3 + X4
##  Model 2: ~ Regimen + dLAge + X1 + X2 + X3 + X4 + Regimen:X1 + Regimen:X2
+ Regimen:X3 + Regimen:X4
##    loglik  Chisq Df P(>|Chi|)
## 1 -2463.3
## 2 -2460.2 6.1889 12    0.9063
```

Model 4: Test interaction of regimen and age

```
## Call:
## coxph(formula = Surv(time, status) ~ Regimen + dLAge + X1 + X2 +
##     X3 + X4 + Regimen:dLAge, data = data1)
##
##                        coef exp(coef) se(coef)      z        p
## RegimenBPaMZ        0.09694   1.10180  0.19708  0.492 0.622804
## RegimenBPaZ        -0.29577   0.74396  0.16926 -1.747 0.080571
## RegimenPaMZ        -0.43964   0.64427  0.12943 -3.397 0.000682
## dLAge              -0.14980   0.86088  0.37648 -0.398 0.690706
```

```
## X1                    1.31937   3.74108  0.53629  2.460 0.013886
## X2                    0.66807   1.95046  0.38655  1.728 0.083937
## X3                    3.06747  21.48738  1.18463  2.589 0.009615
## X4                    1.29838   3.66337  0.48274  2.690 0.007153
## RegimenBPaMZ:dLAge -0.34809   0.70603  0.55565 -0.626 0.531012
## RegimenBPaZ:dLAge  -0.52234   0.59313  0.53922 -0.969 0.332699
## RegimenPaMZ:dLAge  -0.38161   0.68276  0.41963 -0.909 0.363142
##
## Likelihood ratio test=41.83  on 11 df, p=1.732e-05
## n= 619, number of events= 454

## Analysis of Deviance Table
##  Cox model: response is  Surv(time, status)
##  Model 1: ~ Regimen + dLAge + X1 + X2 + X3 + X4
##  Model 2: ~ Regimen + dLAge + X1 + X2 + X3 + X4 + Regimen:dLAge
##    loglik  Chisq Df P(>|Chi|)
## 1 -2463.3
## 2 -2462.8 1.1076  3    0.7752
```

Model 5: Test interaction of age and exposure

```
## Call:
## coxph(formula = Surv(time, status) ~ Regimen + dLAge + X1 + X2 +
##     X3 + X4 + X1:dLAge + X2:dLAge + X3:dLAge + X4:dLAge, data = data1)
##
##                   coef exp(coef)  se(coef)      z        p
## RegimenBPaMZ  0.090039  1.094217  0.188597  0.477 0.633068
## RegimenBPaZ  -0.286043  0.751231  0.163793 -1.746 0.080747
## RegimenPaMZ  -0.470965  0.624399  0.129272 -3.643 0.000269
## dLAge         1.655951  5.238057  1.807794  0.916 0.359664
## X1            1.286604  3.620471  0.554344  2.321 0.020290
## X2            0.775057  2.170715  0.404077  1.918 0.055099
## X3            3.081549 21.792132  1.229866  2.506 0.012225
## X4            1.339349  3.816556  0.507133  2.641 0.008266
## dLAge:X1     -1.956992  0.141283  1.751688 -1.117 0.263907
## dLAge:X2     -0.031450  0.969039  1.193517 -0.026 0.978977
## dLAge:X3     -5.529468  0.003968  3.872112 -1.428 0.153285
## dLAge:X4      0.204891  1.227391  1.274863  0.161 0.872317
##
## Likelihood ratio test=51.95  on 12 df, p=6.327e-07
## n= 619, number of events= 454

## Analysis of Deviance Table
##  Cox model: response is  Surv(time, status)
##  Model 1: ~ Regimen + dLAge + X1 + X2 + X3 + X4
##  Model 2: ~ Regimen + dLAge + X1 + X2 + X3 + X4 + X1:dLAge + X2:dLAge +
X3:dLAge + X4:dLAge
##    loglik  Chisq Df P(>|Chi|)
## 1 -2463.3
## 2 -2457.7 11.226  4   0.02414 *
```

```
## ---
## Signif. codes:  0 '***' 0.001 '**' 0.01 '*' 0.05 '.' 0.1 ' ' 1
```

Interactions of regimen and exposure and of regimen with age are not significant, but there is an interaction with age and exposure, model5. Test proportional hazards:

```
##             chisq df     p
## Regimen    6.2837  3 0.099
## dLAge      3.8690  1 0.049
## X1         0.0596  1 0.807
## X2         2.6753  1 0.102
## X3         1.0851  1 0.298
## X4         2.0271  1 0.155
## dLAge:X1   3.2975  1 0.069
## dLAge:X2   0.4614  1 0.497
## dLAge:X3   3.3956  1 0.065
## dLAge:X4   0.0245  1 0.876
## GLOBAL    14.5785 12 0.265
```

The proportional hazards assumption looks reasonable.

S1.5 Step 4 Assess covariates with missing values

Model 6: Cavitation

```
## Call:
## coxph(formula = Surv(time, status) ~ Regimen + CAV + dLAge +
##     X1 + X2 + X3 + X4 + X1:dLAge + X2:dLAge + X3:dLAge + X4:dLAge,
##     data = dcav)
##
##                    coef exp(coef)  se(coef)      z       p
## RegimenBPaMZ  0.074939  1.077819  0.189378  0.396 0.69232
## RegimenBPaZ  -0.291083  0.747454  0.163729 -1.778 0.07543
## RegimenPaMZ  -0.486038  0.615058  0.129759 -3.746 0.00018
## CAV           0.082255  1.085732  0.142578  0.577 0.56400
## dLAge         1.588281  4.895327  1.828697  0.869 0.38510
## X1            1.308502  3.700625  0.560940  2.333 0.01966
## X2            0.802610  2.231357  0.408171  1.966 0.04926
## X3            3.067077 21.479030  1.244253  2.465 0.01370
## X4            1.328947  3.777066  0.518750  2.562 0.01041
## dLAge:X1     -1.887562  0.151441  1.771641 -1.065 0.28668
## dLAge:X2      0.068323  1.070711  1.206019  0.057 0.95482
## dLAge:X3     -5.454817  0.004276  3.916668 -1.393 0.16370
## dLAge:X4      0.260430  1.297488  1.288989  0.202 0.83988
##
## Likelihood ratio test=53.67  on 13 df, p=6.919e-07
## n= 613, number of events= 449
```

Cavitation is not significant.

Model BTTP1: Baseline TTP

```
## Call:
## coxph(formula = Surv(time, status) ~ Regimen + dLBTTP + dLAge +
##     X1 + X2 + X3 + X4 + X1:dLAge + X2:dLAge + X3:dLAge + X4:dLAge,
##     data = datBTTP)
##
##                   coef exp(coef) se(coef)      z       p
## RegimenBPaMZ  0.71139   2.03681  0.20617  3.450 0.00056
## RegimenBPaZ   0.52447   1.68956  0.19591  2.677 0.00743
## RegimenPaMZ   0.04200   1.04289  0.14557  0.289 0.77296
## dLBTTP        0.89404   2.44500  0.10585  8.446 < 2e-16
## dLAge         1.03246   2.80797  1.78163  0.580 0.56225
## X1            1.12329   3.07496  0.55940  2.008 0.04464
## X2            0.67065   1.95552  0.40411  1.660 0.09700
## X3            2.95552  19.21164  1.24460  2.375 0.01756
## X4            1.43613   4.20439  0.49986  2.873 0.00407
## dLAge:X1     -1.40253   0.24597  1.72377 -0.814 0.41585
## dLAge:X2      0.26874   1.30831  1.17352  0.229 0.81887
## dLAge:X3     -4.59272   0.01013  3.81361 -1.204 0.22848
## dLAge:X4     -0.13465   0.87402  1.26010 -0.107 0.91490
##
## Likelihood ratio test=117.7  on 13 df, p=< 2.2e-16
## n= 611, number of events= 447
```

There is strong evidence that baseline TTP is related to outcome. Test the assumption of proportional hazards:

```
##              chisq df       p
## Regimen  3.64e+00  3   0.304
## dLBTTP   4.10e+01  1 1.5e-10
## dLAge    3.83e+00  1   0.050
## X1       2.12e-02  1   0.884
## X2       8.28e-01  1   0.363
## X3       5.72e-01  1   0.449
## X4       1.73e+00  1   0.188
## dLAge:X1 2.71e+00  1   0.100
## dLAge:X2 6.57e-01  1   0.418
## dLAge:X3 3.79e+00  1   0.052
## dLAge:X4 8.36e-04  1   0.977
## GLOBAL   5.63e+01 13 2.4e-07
```

There is strong evidence that baseline TTP violates the assumption of proportional hazards. Examine Shcoenfeld residuals to explore the nature of the dependence of the outcome on baseline TTP.

```
## `geom_smooth()` using method = 'loess' and formula 'y ~ x'
```

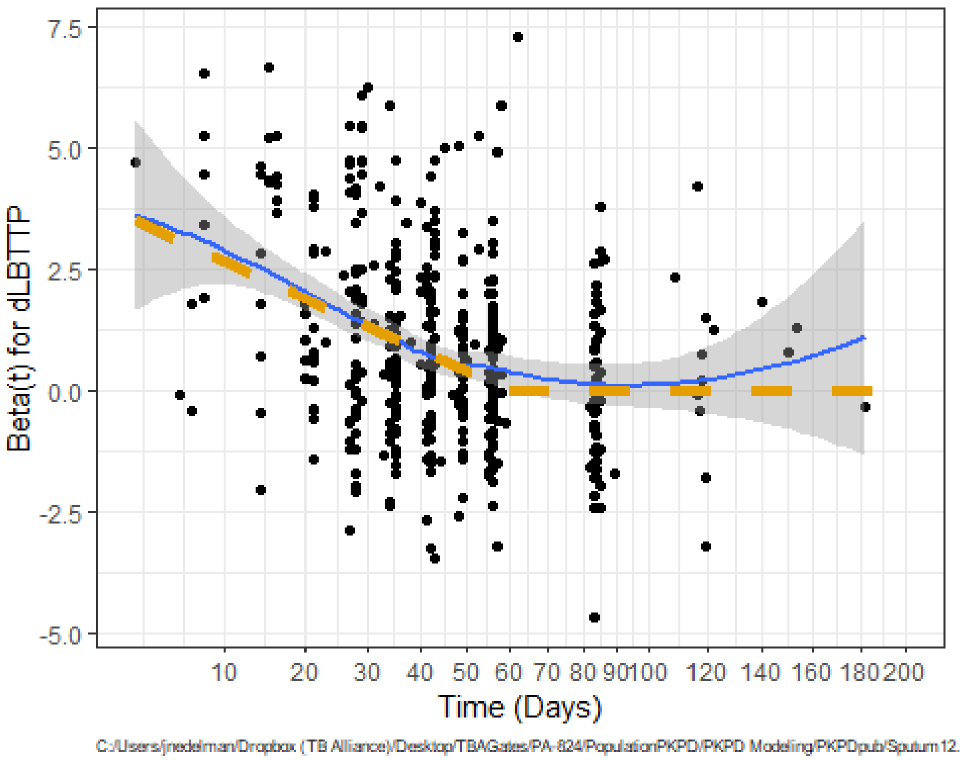

Schoenfeld residuals show that the impact of BTTP decreases with time. The residuals appear to decrease approximately linearly in sqrt(time) until ~ 60 days, then they are flat. Fit a model with a time-dependent coefficient of this form on dLBTTP.

Model BTTP2: Time-dependent coefficient on baseline TTP

```
## Call:
## coxph(formula = Surv(time, status) ~ Regimen + dLBTTP + tt(dLBTTP) +
##     dLAge + X1 + X2 + X3 + X4 + X1:dLAge + X2:dLAge + X3:dLAge +
##     X4:dLAge, data = datBTTP, tt = function(x, t, ...) x * sqrt(pmin(t,
##     60)))
##
##                   coef exp(coef) se(coef)      z        p
## RegimenBPaMZ   0.87076   2.38872  0.21140  4.119 3.80e-05
## RegimenBPaZ    0.65803   1.93099  0.20168  3.263   0.0011
## RegimenPaMZ    0.15447   1.16704  0.15106  1.023   0.3065
## dLBTTP         4.06702  58.38283  0.46169  8.809  < 2e-16
## tt(dLBTTP)    -0.50552   0.60319  0.07236 -6.986 2.83e-12
## dLAge          1.01547   2.76065  1.72112  0.590   0.5552
## X1             1.01168   2.75022  0.53843  1.879   0.0603
## X2             0.69992   2.01360  0.39172  1.787   0.0740
## X3             2.72708  15.28820  1.19451  2.283   0.0224
## X4             1.23937   3.45345  0.49555  2.501   0.0124
## dLAge:X1      -1.46041   0.23214  1.66786 -0.876   0.3812
## dLAge:X2       0.42242   1.52565  1.14525  0.369   0.7122
## dLAge:X3      -4.56607   0.01040  3.68843 -1.238   0.2157
## dLAge:X4      -0.37918   0.68442  1.26662 -0.299   0.7647
```

```
##
## Likelihood ratio test=169.5  on 14 df, p=< 2.2e-16
## n= 611, number of events= 447
```

Now assess interactions with baseline TTP.

Model BTTP3: Test interaction of baseline TTP with regimen

```
## Call:
## coxph(formula = Surv(time, status) ~ Regimen + dLBTTP + tt(dLBTTP) +
##     dLAge + X1 + X2 + X3 + X4 + X1:dLAge + X2:dLAge + X3:dLAge +
##     X4:dLAge + Regimen:dLBTTP + Regimen:tt(dLBTTP), data = datBTTP,
##     tt = function(x, t, ...) x * sqrt(pmin(t, 60)))
##
##                                coef  exp(coef)  se(coef)      z        p
## RegimenBPaMZ              1.090e+00  2.974e+00  4.418e-01  2.467   0.0136
## RegimenBPaZ               1.815e+00  6.141e+00  4.022e-01  4.512 6.42e-06
## RegimenPaMZ               1.243e-01  1.132e+00  1.856e-01  0.670   0.5031
## dLBTTP                    5.244e+00  1.893e+02  1.025e+00  5.115 3.13e-07
## tt(dLBTTP)               -6.842e-01  5.045e-01  1.645e-01 -4.160 3.19e-05
## dLAge                     9.976e-01  2.712e+00  1.720e+00  0.580   0.5619
## X1                        1.007e+00  2.738e+00  5.360e-01  1.879   0.0603
## X2                        7.354e-01  2.086e+00  3.902e-01  1.885   0.0595
## X3                        2.654e+00  1.421e+01  1.189e+00  2.232   0.0256
## X4                        1.173e+00  3.231e+00  4.963e-01  2.363   0.0181
## dLAge:X1                 -1.375e+00  2.528e-01  1.669e+00 -0.824   0.4101
## dLAge:X2                  3.383e-01  1.403e+00  1.147e+00  0.295   0.7680
## dLAge:X3                 -4.432e+00  1.189e-02  3.683e+00 -1.203   0.2288
## dLAge:X4                 -2.571e-01  7.733e-01  1.274e+00 -0.202   0.8400
## RegimenBPaMZ:dLBTTP       1.374e-01  1.147e+00  1.914e+00  0.072   0.9428
## RegimenBPaZ:dLBTTP       -8.722e-02  9.165e-01  1.485e+00 -0.059   0.9532
## RegimenPaMZ:dLBTTP       -2.027e+00  1.318e-01  1.237e+00 -1.638   0.1015
## RegimenBPaMZ:tt(dLBTTP) -2.566e-04  9.997e-01  2.768e-01 -0.001   0.9993
## RegimenBPaZ:tt(dLBTTP)    1.711e-01  1.187e+00  2.231e-01  0.767   0.4430
## RegimenPaMZ:tt(dLBTTP)    2.872e-01  1.333e+00  1.905e-01  1.507   0.1317
##
## Likelihood ratio test=181.8  on 20 df, p=< 2.2e-16
## n= 611, number of events= 447
```

```
## Analysis of Deviance Table
##  Cox model: response is  Surv(time, status)
##  Model 1: ~ Regimen + dLBTTP + tt(dLBTTP) + dLAge + X1 + X2 + X3 + X4 +
X1:dLAge + X2:dLAge + X3:dLAge + X4:dLAge
##  Model 2: ~ Regimen + dLBTTP + tt(dLBTTP) + dLAge + X1 + X2 + X3 + X4 +
X1:dLAge + X2:dLAge + X3:dLAge + X4:dLAge + Regimen:dLBTTP +
Regimen:tt(dLBTTP)
##    loglik  Chisq Df P(>|Chi|)
## 1 -2355.9
## 2 -2349.7 12.258  6   0.05646 .
## ---
## Signif. codes:  0 '***' 0.001 '**' 0.01 '*' 0.05 '.' 0.1 ' ' 1
```

Model BTTP4: Test interaction of baseline TTP with age

```
## Call:
## coxph(formula = Surv(time, status) ~ Regimen + dLBTTP + tt(dLBTTP) +
##     dLAge + X1 + X2 + X3 + X4 + X1:dLAge + X2:dLAge + X3:dLAge +
##     X4:dLAge + dLAge:dLBTTP + dLAge:tt(dLBTTP), data = datBTTP,
##     tt = function(x, t, ...) x * sqrt(pmin(t, 60)))
##
##                      coef exp(coef) se(coef)      z        p
## RegimenBPaMZ      0.87515   2.39922  0.21187  4.131 3.62e-05
## RegimenBPaZ       0.66429   1.94312  0.20209  3.287  0.00101
## RegimenPaMZ       0.16055   1.17416  0.15138  1.061  0.28888
## dLBTTP            4.02418  55.93425  0.46719  8.614  < 2e-16
## tt(dLBTTP)       -0.49974   0.60669  0.07312 -6.835 8.22e-12
## dLAge             1.09137   2.97835  1.74358  0.626  0.53136
## X1                1.02474   2.78638  0.53555  1.913  0.05569
## X2                0.68901   1.99173  0.39058  1.764  0.07773
## X3                2.76051  15.80798  1.18816  2.323  0.02016
## X4                1.25702   3.51493  0.49727  2.528  0.01148
## dLAge:X1         -1.47064   0.22978  1.66976 -0.881  0.37845
## dLAge:X2          0.41384   1.51261  1.14528  0.361  0.71784
## dLAge:X3         -4.55599   0.01050  3.69159 -1.234  0.21715
## dLAge:X4         -0.33896   0.71251  1.26998 -0.267  0.78954
## dLBTTP:dLAge     -1.44458   0.23584  1.00633 -1.435  0.15114
## tt(dLBTTP):dLAge  0.22817   1.25630  0.15551  1.467  0.14232
##
## Likelihood ratio test=171.7  on 16 df, p=< 2.2e-16
## n= 611, number of events= 447

## Analysis of Deviance Table
##  Cox model: response is  Surv(time, status)
##  Model 1: ~ Regimen + dLBTTP + tt(dLBTTP) + dLAge + X1 + X2 + X3 + X4 +
X1:dLAge + X2:dLAge + X3:dLAge + X4:dLAge
##  Model 2: ~ Regimen + dLBTTP + tt(dLBTTP) + dLAge + X1 + X2 + X3 + X4 +
X1:dLAge + X2:dLAge + X3:dLAge + X4:dLAge + dLAge:dLBTTP + dLAge:tt(dLBTTP)
##    loglik  Chisq Df P(>|Chi|)
## 1 -2355.9
## 2 -2354.8 2.1595  2    0.3397
```

Model BTTP5: Test interaction of baseline TTP with exposure

```
## Call:
## coxph(formula = Surv(time, status) ~ Regimen + dLBTTP + tt(dLBTTP) +
##     dLAge + X1 + X2 + X3 + X4 + X1:dLAge + X2:dLAge + X3:dLAge +
##     X4:dLAge + X1:dLBTTP + X2:dLBTTP + X3:dLBTTP + X4:dLBTTP +
##     X1:tt(dLBTTP) + X2:tt(dLBTTP) + X3:tt(dLBTTP) + X4:tt(dLBTTP),
##     data = datBTTP, tt = function(x, t, ...) x * sqrt(pmin(t,
##         60)))
##
##                     coef  exp(coef)   se(coef)      z       p
## RegimenBPaMZ   8.537e-01  2.348e+00  2.115e-01  4.037 5.4e-05
## RegimenBPaZ    6.406e-01  1.898e+00  2.020e-01  3.171 0.00152
```

```
## RegimenPaMZ    1.480e-01  1.160e+00  1.522e-01  0.972 0.33089
## dLBTTP         7.241e+00  1.396e+03  5.729e+00  1.264 0.20622
## tt(dLBTTP)    -9.437e-01  3.892e-01  7.669e-01 -1.230 0.21853
## dLAge          1.016e+00  2.762e+00  1.926e+00  0.528 0.59771
## X1             1.026e+00  2.789e+00  1.353e+00  0.758 0.44843
## X2             4.506e-01  1.569e+00  8.882e-01  0.507 0.61190
## X3             2.909e+00  1.834e+01  2.903e+00  1.002 0.31624
## X4             1.759e+00  5.808e+00  9.015e-01  1.951 0.05101
## dLAge:X1      -1.579e+00  2.061e-01  1.864e+00 -0.847 0.39676
## dLAge:X2       6.047e-01  1.831e+00  1.271e+00  0.476 0.63438
## dLAge:X3      -4.682e+00  9.260e-03  4.091e+00 -1.145 0.25239
## dLAge:X4      -1.184e+00  3.059e-01  1.466e+00 -0.808 0.41901
## dLBTTP:X1     -2.713e+00  6.633e-02  5.498e+00 -0.493 0.62169
## dLBTTP:X2     -1.389e+00  2.494e-01  3.631e+00 -0.382 0.70211
## dLBTTP:X3     -7.193e+00  7.518e-04  1.192e+01 -0.604 0.54606
## dLBTTP:X4      3.433e-01  1.410e+00  3.418e+00  0.100 0.92000
## tt(dLBTTP):X1  3.836e-01  1.468e+00  7.364e-01  0.521 0.60244
## tt(dLBTTP):X2  1.249e-01  1.133e+00  5.026e-01  0.249 0.80367
## tt(dLBTTP):X3  1.058e+00  2.882e+00  1.602e+00  0.661 0.50885
## tt(dLBTTP):X4  8.049e-02  1.084e+00  5.348e-01  0.151 0.88036
##
## Likelihood ratio test=172.8  on 22 df, p=< 2.2e-16
## n= 611, number of events= 447
```

```
## Analysis of Deviance Table
##  Cox model: response is  Surv(time, status)
##  Model 1: ~ Regimen + dLBTTP + tt(dLBTTP) + dLAge + X1 + X2 + X3 + X4 +
X1:dLAge + X2:dLAge + X3:dLAge + X4:dLAge
##  Model 2: ~ Regimen + dLBTTP + tt(dLBTTP) + dLAge + X1 + X2 + X3 + X4 +
X1:dLAge + X2:dLAge + X3:dLAge + X4:dLAge + X1:dLBTTP + X2:dLBTTP + X3:dLBTTP
+ X4:dLBTTP + X1:tt(dLBTTP) + X2:tt(dLBTTP) + X3:tt(dLBTTP) + X4:tt(dLBTTP)
##    loglik  Chisq Df P(>|Chi|)
## 1 -2355.9
## 2 -2354.2 3.2399  8    0.9184
```

None of the interactions with baseline TTP are significant.

S1.6 Step 5: Optimize functional form of Cavg

The best model so far, BTTP2, represents exposure as a 4-df spline of dLCavg. Try models with 1-, 2-, and 3-df splines, and examine AICs.

```
##          df      AIC
## df1model  8 4740.079
## df2model 10 4744.049
## df3model 12 4736.777
## BTTP2    14 4739.721
```

The final model is the one with the 3-df spline for dLCavg. The three spline variables, X1, X2, and X3, are obtained as `ns(dLCavg, df=3)`. Then the model fit is:

```
## Call:
## coxph(formula = Surv(time, status) ~ Regimen + dLBTTP + tt(dLBTTP) +
##     dLAge + X1 + X2 + X3 + X1:dLAge + X2:dLAge + X3:dLAge, data = spl3,
##     tt = function(x, t, ...) x * sqrt(pmin(t, 60)))
##
##                   coef exp(coef) se(coef)      z        p
## RegimenBPaMZ   0.86427   2.37326  0.21077  4.100 4.12e-05
## RegimenBPaZ    0.64777   1.91128  0.20029  3.234  0.00122
## RegimenPaMZ    0.15566   1.16843  0.15009  1.037  0.29968
## dLBTTP         4.06177  58.07683  0.46086  8.814  < 2e-16
## tt(dLBTTP)    -0.50416   0.60401  0.07223 -6.980 2.94e-12
## dLAge          0.49693   1.64367  1.51450  0.328  0.74282
## X1             0.38988   1.47680  0.28997  1.345  0.17877
## X2             2.31300  10.10465  1.03074  2.244  0.02483
## X3             1.27462   3.57732  0.40339  3.160  0.00158
## dLAge:X1       1.03033   2.80198  0.91264  1.129  0.25892
## dLAge:X2      -3.33582   0.03559  3.18279 -1.048  0.29460
## dLAge:X3      -0.35552   0.70081  1.05132 -0.338  0.73524
##
## Likelihood ratio test=168.5  on 12 df, p=< 2.2e-16
## n= 611, number of events= 447
```

S1.7 Hazard ratios from the final model

Plot hazard ratios as a function of Cavg, where the condition for the reference hazard (i.e., the denominator of the ratios) is Cavg=2.6 µg/mL. The same ratio applies within any regimen; the regimen contribution will cancel across the numerator and denominator. However, it will not apply across regimens. The same ratio applies for any value of dLBTTP at any particular time, or for dLBTTP=0 at all times. Such contributions will also cancel across the numerator and denominator. Here is further explanation: We can write the hazard function for the final model as:

```
h(t; Regimen, dLBTTP, dLAge, dLCavg) =
   h0(t) x
   exp(beta1.Reg x (Reg=="BPaZ") + beta2.Reg x (Reg=="BPaMZ") +
         beta3.Reg x (Reg=="PaMZ")) x
     exp(dLBTTP x [beta0.dLBTTP + {beta1.dLBPTTP x f(t)}]) x
     exp(dLAge x beta.dLAge) x
     exp(spline(dLCavg) x beta.spline) x
     exp(dLAge:spline(dLCavg) x beta.interaction)
```

where:

- h0(t) is the “baseline hazard”, which describes 35-year-old subjects (i.e., dLAge == 0) on BPaL with Cavg = 2.6 ug/mL (i.e., dLCavg == 0) and BTTP = 270 (i.e., dLBTTP == 0).
- beta1.Reg, beta2.Reg, and beta3.Reg are contributions for BPaZ, BPaMZ, and PaMZ, respectively.
- dLBTTP is the value of centered baseline log-transformed TTP, and beta0.dLBTTP is the corresponding coefficient.

- f(t) = sqrt(pmin(t,60)), and beta1.dLBTTP is the corresponding coefficient.
- dLAge is the value of centered baseline log-transformed Age, and beta.dLAge is the corresponding coefficient.
- spline(dLCavg) is the vector of spline terms determined by the value dLCAVG, and beta.spline is the corresponding vector of coefficients.
- dLAge:spline(dLCavg) is the vector of spline terms for dLCavg multipled by the value of dLAge, and beta.interaction is the corresponding vector of coefficients.

Then the hazard ratios we are interested in are h(t; Regimen, dLBTTP, dLAge, dLCavg)/h(t; Regimen, dLBTTP, dLAge, 2.6) =
exp([spline(dLCAVG) - spline(0)] x beta.spline) x
exp([dLAge:spline(dLCavg) - dLAge:spline(0)] x beta.interaction)

If we restrict attention to dLAge = 0, i.e., Age = 35 years, then the final term disappears; this is what we will do.

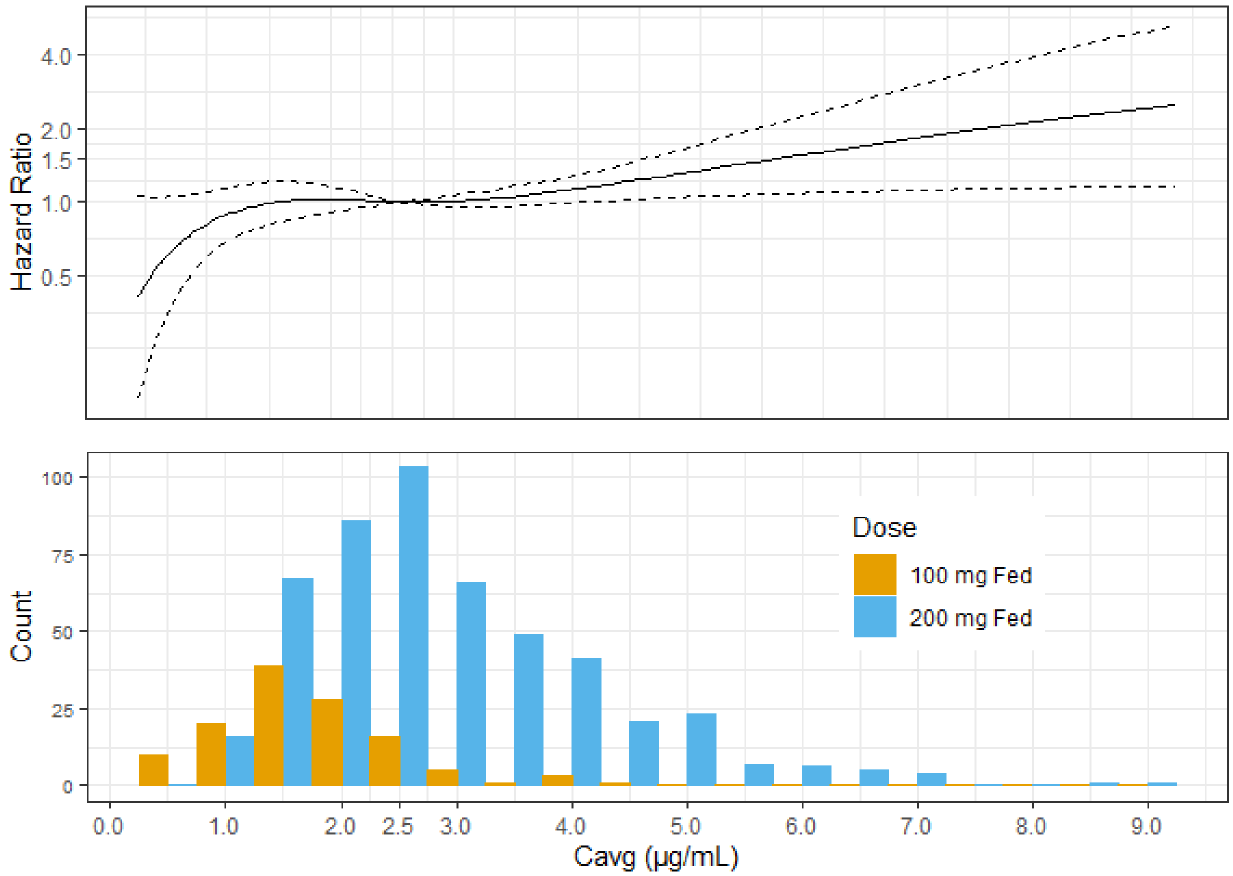

S1.8 Probabilities of failure to sputum convert at 8 weeks

For Cavg, use the 10th and 50th quantiles from Nix-TB (1.5 and 2.6) and half these values, which would represent expected quantiles for 100 mg fed, or 200 mg fasted, in a Nix-TB-like population.

For Age, use the following values for age (quartiles of age in Nix-TB): 28, 35, and 43 years.

```
##    Age    Exposure 95% CI Lower  Estimate 95% CI Upper
## 1   35 100 mg 10%    0.2297536 0.3767186    0.6176919
## 2   35 100 mg 50%    0.1655940 0.2498904    0.3770981
## 3   35 200 mg 10%    0.1517899 0.2302126    0.3491526
## 4   35 200 mg 50%    0.1537186 0.2245317    0.3279660

##    Age    Exposure 95% CI Lower  Estimate 95% CI Upper
## 1   28 100 mg 10%   0.16433630 0.3219128    0.6305839
## 2   28 100 mg 50%   0.08778393 0.1568008    0.2800797
## 3   28 200 mg 10%   0.07879627 0.1403927    0.2501400
## 4   28 200 mg 50%   0.12188077 0.1874417    0.2882685

##    Age    Exposure 95% CI Lower  Estimate 95% CI Upper
## 1   43 100 mg 10%    0.2577124 0.4271452    0.7079716
## 2   43 100 mg 50%    0.2422070 0.3459426    0.4941076
## 3   43 200 mg 10%    0.2271239 0.3250545    0.4652104
## 4   43 200 mg 50%    0.1805394 0.2606713    0.3763695
```

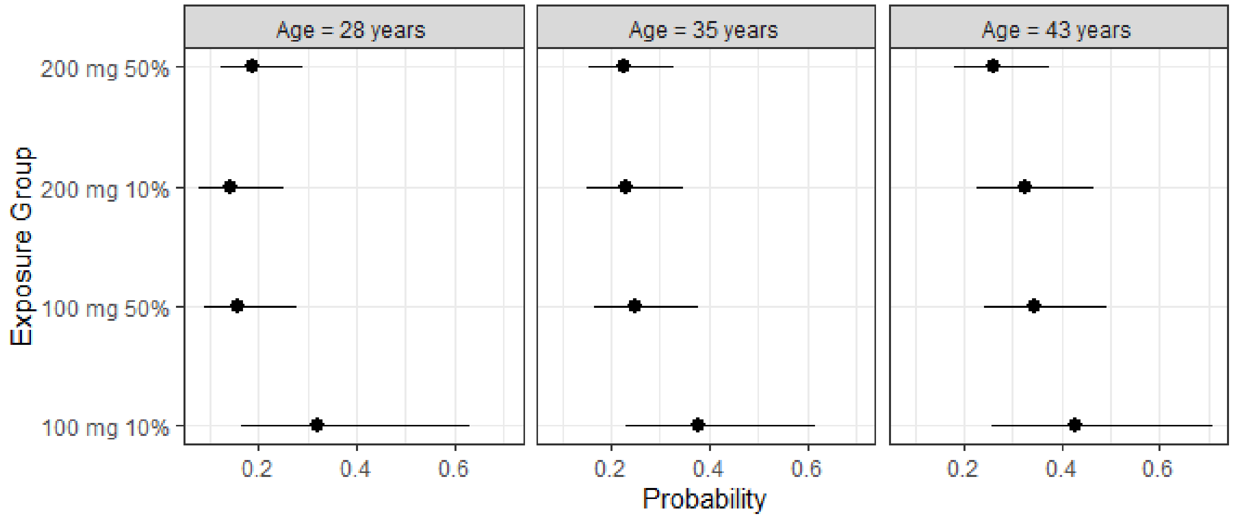

S2. Predicting clinical outcome from F8W

S2.1 The method of Wallis et al (2015)

Wallis et al (2015) modeled the risk of TB recurrence in terms of the proportion positive at two months, which we will interpret as the probabilit of F8W, and the duration (in months) of treatment. Their model is represented by the following equation:

$$logit(P(recurrence)) = 2.5289 + 0.4399 \times logit(P(F8W)) - 2.5018 \times log(Duration)$$

That model is applied here for P(F8W) = 0.23 or 0.38, and Duration = 6 months, yielding probabilities of recurrence 0.077 and 0.103, respectively, or a 34% increase at the lower or fasted dose.

S2.2 Bayesian categorical data analysis based on Nix-TB results

First we find the distribution of patients from the MITT population in Nix-TB by baseline status, week 8 status, and clinical outcome.

```
## # A tibble: 4 x 4
##   BaselineStatus Week8Status    Favorable Unfavorable
##   <fct>          <fct>              <int>       <int>
## 1 Positive       Converted             70           2
## 2 Positive       Still Positive        12           3
## 3 Positive       Died                   0           4
## 4 Negative       N/A                   16           0
```

Now, consider the seven entries in the above table excluding Died/Favorable, which is impossible, to represent a sample from a multinomial distribution with probabilities, in order from left to right and top to bottom:

$$p_{PCF}, p_{PCU}, p_{PSF}, p_{PSU}, p_{PDU}, p_{N-F}, p_{N-U}$$

Find the joint posterior assuming a uniform Dirichlet prior. Then what we're interested in is the posterior distribution of

$$P(Unfavorable\ Outcome) = p_{PCU} + p_{PSU} + p_{PDU} + p_{N-U}$$

conditional on

$$P(F8W) = (p_{PSF} + p_{PSU})/(p_{PCF} + p_{PCU} + p_{PSF} + p_{PSU})$$

with P(F8W) equal to 0.23 or 0.38, corresponding to 200 mg fed and 100 mg fed/200 mg fasted.

To find the conditional posterior of interest, an extremely large number (10 million) of samples from the joint posterior of the multinomial probabilities will be generated. From these, subsets of at least 1000 samples will be extracted where P(F8W) is in an interval around 0.23 or 0.38. Because 0.23 is more consistent with the observed data than 0.38, a

narrower interval will be possible for 0.23 than for 0.38. Here are the resulting intervals and subset sizes:

```
##      pF8W interval1 interval2   subsize
##     0.380     0.362     0.398  1001.000

##       pF8W  interval1  interval2    subsize
##      0.230      0.229      0.231 101636.000
```

Now, with these subsets, the conditional probability of Unfavorable Outcome given P(F8W) is evaluated for P(F8W) = 0.23 and 0.38. First for 0.23:

```
##
## Summary of Conditional Posterior

##    Min. 1st Qu.  Median    Mean 3rd Qu.    Max.
## 0.02949 0.09924 0.11863 0.12066 0.14000 0.28887

##
## 95% Highest Density Credible Interval for Conditional Posterior

## [1] 0.06361497 0.17973232
```

And now for 0.38:

```
##
## Summary of Conditional Posterior

##    Min. 1st Qu.  Median    Mean 3rd Qu.    Max.
## 0.05177 0.11483 0.13914 0.14246 0.16916 0.28616

##
## 95% Highest Density Credible Interval for Conditional Posterior

## [1] 0.06615827 0.21215668
```

S3. Screeing adverse events for evidence of relationship with exposure

S3.0 Events to consider

Sets of adverse events were considered for investigation if they were identified as Adverse Drug Reactions (ADRs) in pretomanid's Investigator Brochure or as AEs of special interest in the NDA submission. A set was included if at least 10% of the subjects in the data pool had such treatment emergent events.

ADRs were:

- Mild to moderate (G1 to G2) nausea (preferred term [PT]) and vomiting (PT)
- G1 and G2 rash (grouped terms, including the following PTs: rash; rash papular; rash maculo-papular; rash erythematous; and rash vesicular)
- Transaminases increased (grouped terms, including the following PTs: transaminases abnormal; transaminases increased; ALT abnormal; AST abnormal; ALT elevated; AST elevated; hepatic enzyme increased; hepatic enzyme abnormal; liver function test abnormal; liver function test increased; hepatic function abnormal)

The single PTs of ALT increased and AST increased were also considered.

AEs of special interest were:

- Hepatic toxicity: all PTs identified in SMQ 'Hepatic Disorder'
- Cataract: all PTs identified in SMQ 'Lens disorders'
- Convulsion: all PTs in SMQ 'Convulsions'
- Testicular degeneration: all PTs identified in SMQ 'Fertility disorders'
- Cutaneous reactions: all PTs identified in SMQ: 'Severe cutaneous adverse reactions'
- Headache: all PTs under High Level Group Term 'Headaches'
- Gastrointestinal Disorders: all PTs under High Level Terms 'Nausea and Vomiting Symptoms', 'Diarrhoea (excl ineffective)'
- Skin and Subcutaneous Tissue Disorders: all PTs under High Level Terms 'Dermatitis and Eczema', 'Rashes, Eruptions and Exanthems NEC', 'Acnes'

The single preferred term of Headache was also considered.

The following table shows the percentages of subjects in the data pool with these events.

```
## # A tibble: 15 x 2
##    AE            Percent
##    <chr>           <dbl>
##  1 GI               28.4
##  2 Hepatic          25.5
##  3 Transaminases    19.2
##  4 Nausea           17.6
##  5 Skin             16.6
##  6 Vomiting         14.7
```

```
##   7 ALT                14.3
##   8 AST                14
##   9 HeadachesSMQ       11
## 10 HeadachePT         10.7
## 11 Rash                9
## 12 Cutaneous           3.4
## 13 Cataract            3.2
## 14 Convulsions         0.6
## 15 Fertility           0.2
```

S3.1 Objective and methodology

The objective here is to screen the ten sets of AEs of interest for evidence of a relationship between time-to-occurrence and pretomanid exposure. Those AEs that pass this screen will be analyzed more deeply later.

For each of the ten sets of AEs, the following is done:

- Plot Kaplan-Meier curves stratified by 10-80-10 of Cavg.
- Cox model with Cavg and Regimen as covariates.
- Box plots of Cavg for those ever/never having the AE by 8 weeks.
- Linear model of log(Cavg) versus AE (yes/no) and Regimen

S3.2 Results

S3.2.0 Patients by study in the analysis dataset

```
## STUDYID
## NC-002 NC-005 NC-006 Nix-TB    Sum
##    147    174    214    109    644
```

S3.2.1 Nausea

```
## Distribution of all events by grade:
##   1   2   3 Sum
## 129  17   1 147
## Number of first events
## [1] 115
```

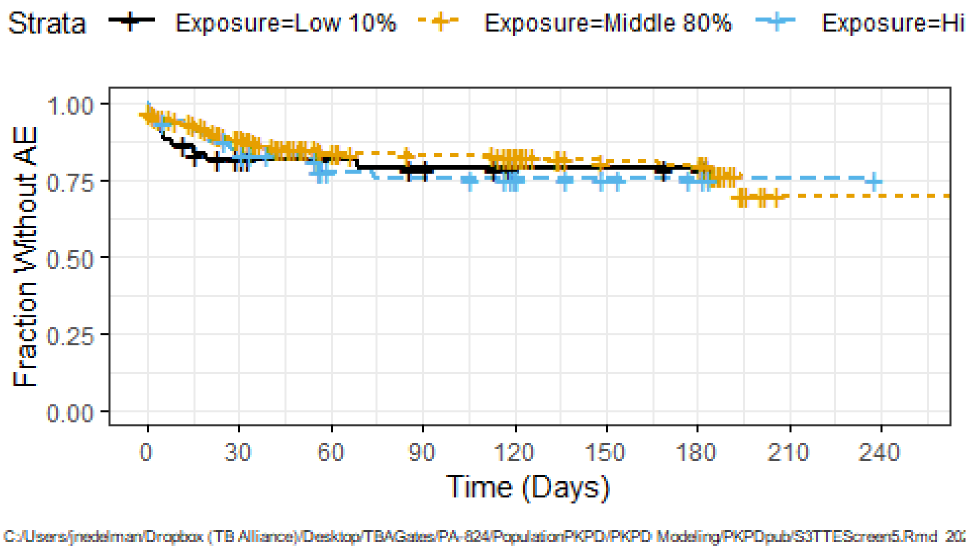


```
## Cox model with regimen and dLCavg, p-value for dLCavg
## [1] 0.6427049
```

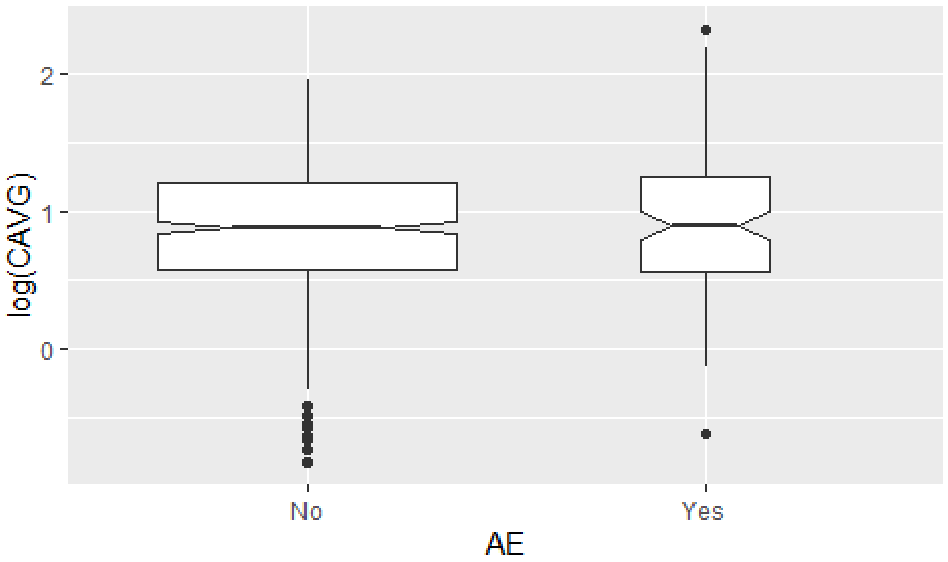


```
## Linear model for log(Cavg) vs AE and Regimen
## [1] 0.8721875
```

No evidence of E-R.

S3.2.2 Vomiting

```
## Distribution of all events by grade:
##   1   2   3 Sum
## 108  20   2 130
## Number of first events
## [1] 96
```

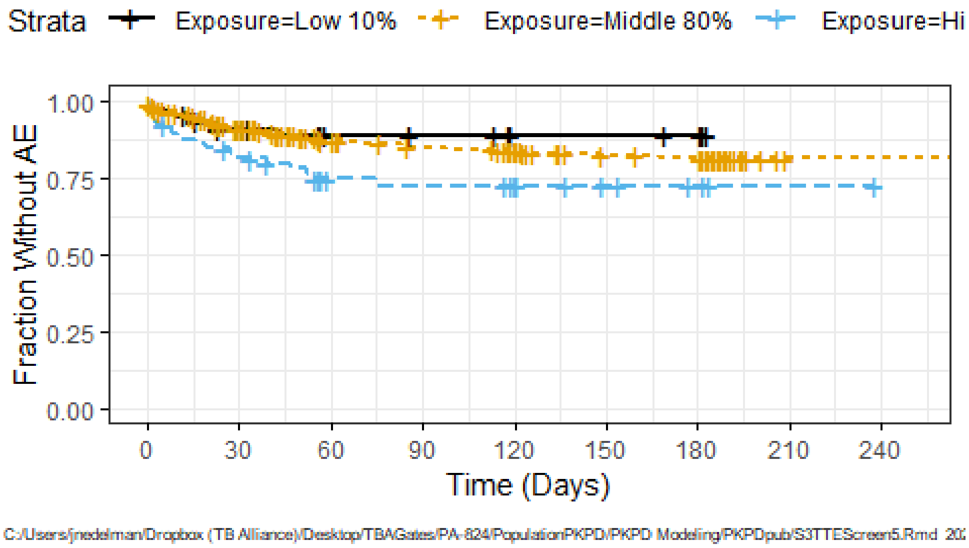


```
## Cox model with regimen and dLCavg, p-value for dLCavg
## [1] 0.002097055
```

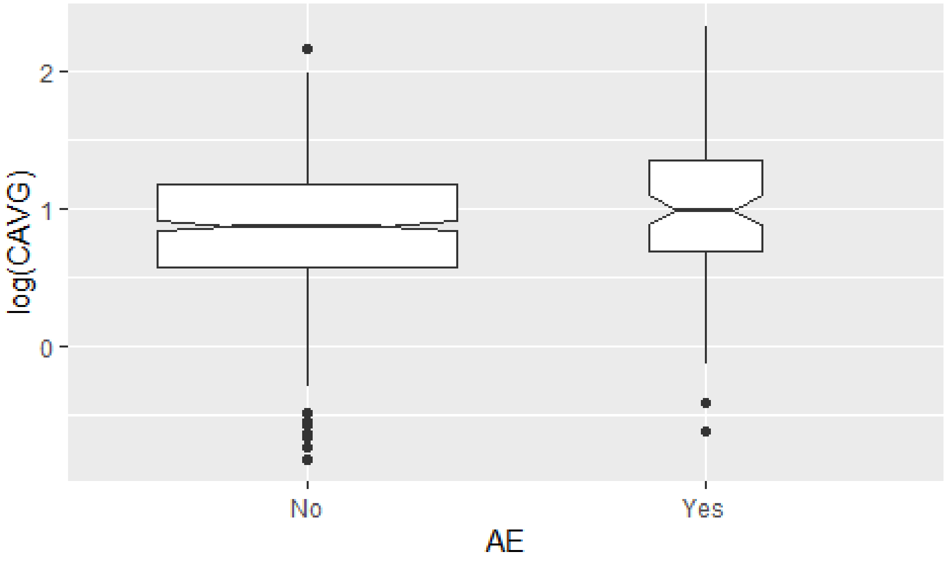


```
## Linear model for log(Cavg) vs AE and Regimen
## [1] 0.02180037
```

Evidence of E-R (all four assessments).

S3.2.3 ALT Increased

```
## Distribution of all events by grade:
##    1    2    3    4 Sum
##   31   21   43   21 116
## Number of first events
## [1] 92
```

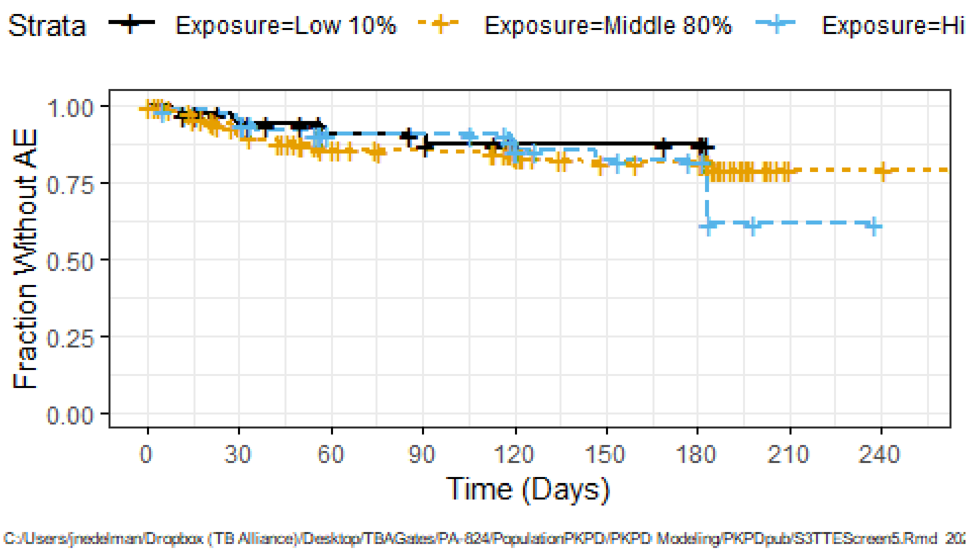


```
## Cox model with regimen and dLCavg, p-value for dLCavg
## [1] 0.5140642
```

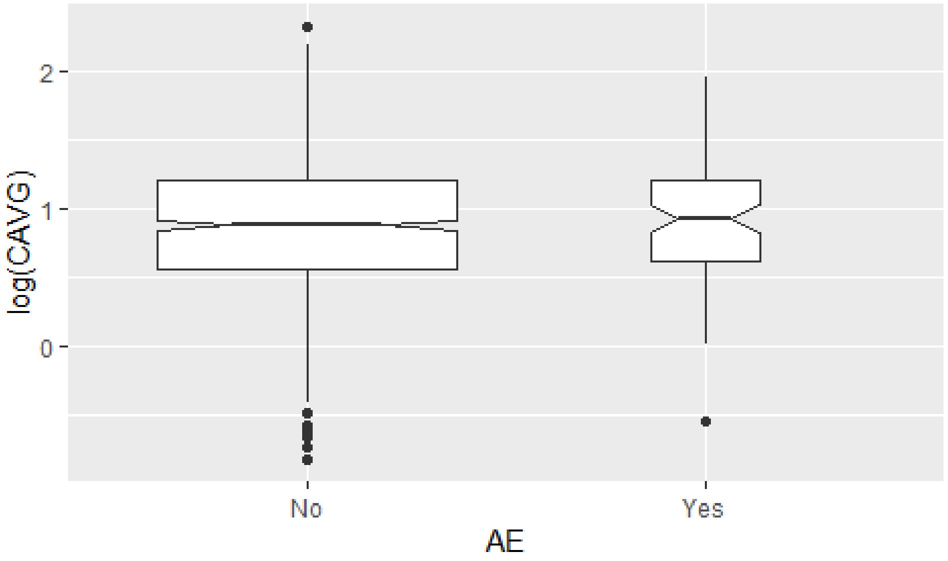


```
## Linear model for log(Cavg) vs AE and Regimen
## [1] 0.3911235
```

No evidence of E-R.

S3.2.4 AST Increased

```
## Distribution of all events by grade:
##   1   2   3   4 Sum
##  28  20  44  21 113
## Number of first events
## [1] 89
```

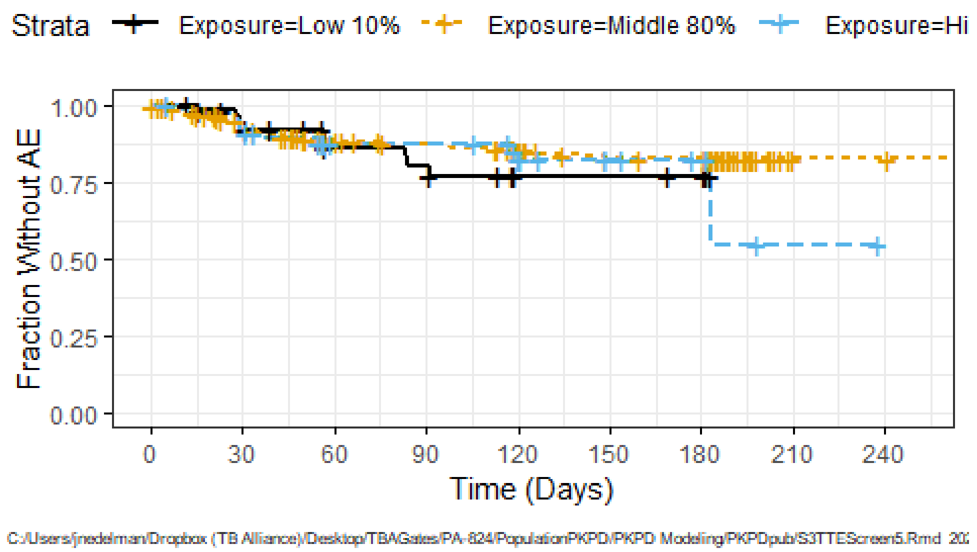


```
## Cox model with regimen and dLCavg, p-value for dLCavg
## [1] 0.593175
```

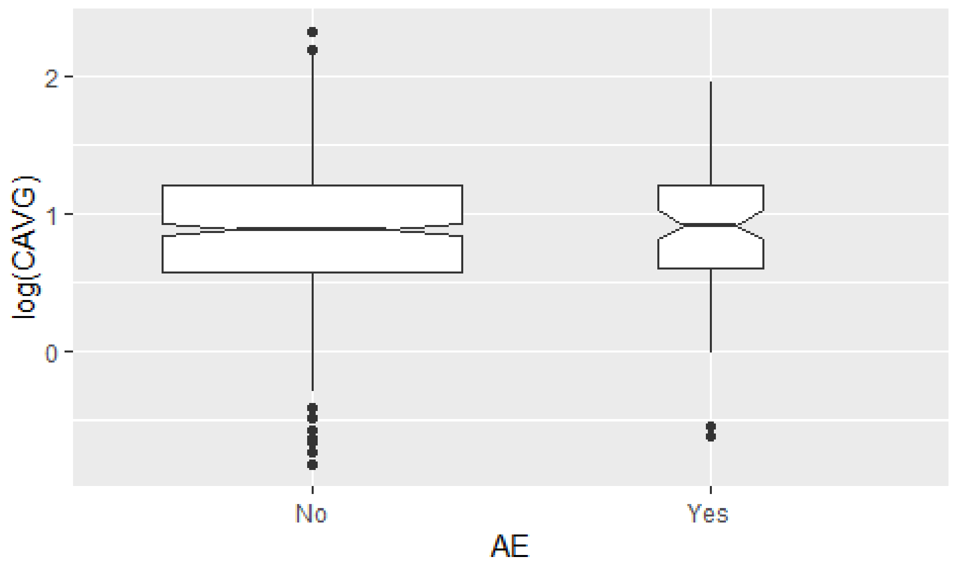


```
## Linear model for log(Cavg) vs AE and Regimen
## [1] 0.5210795
```

No evidence of E-R.

S3.2.5 Headache

```
## Distribution of all events by grade:
##    1   2   3 Sum
##   58  15   2  75
## Number of first events
## [1] 70
```

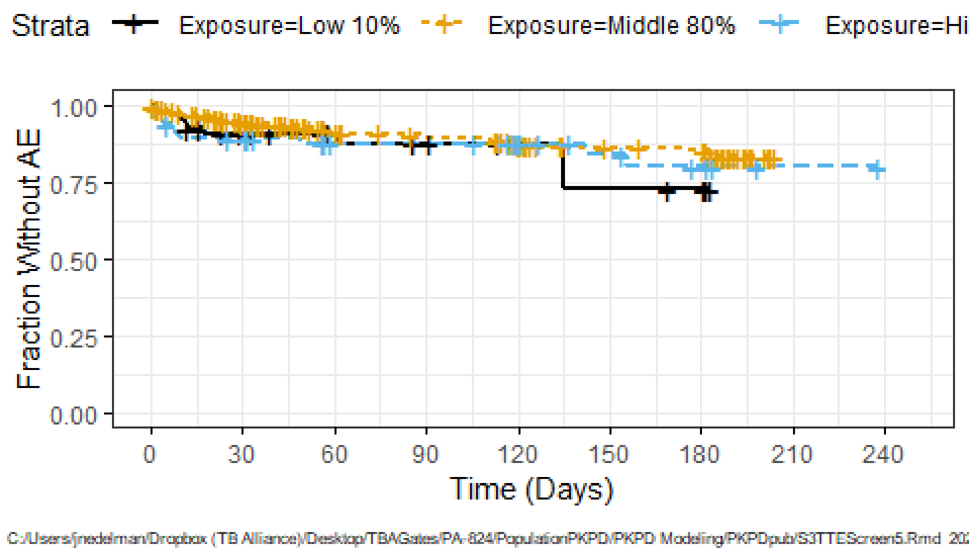


```
## Cox model with regimen and dLCavg, p-value for dLCavg
## [1] 0.5639203
```

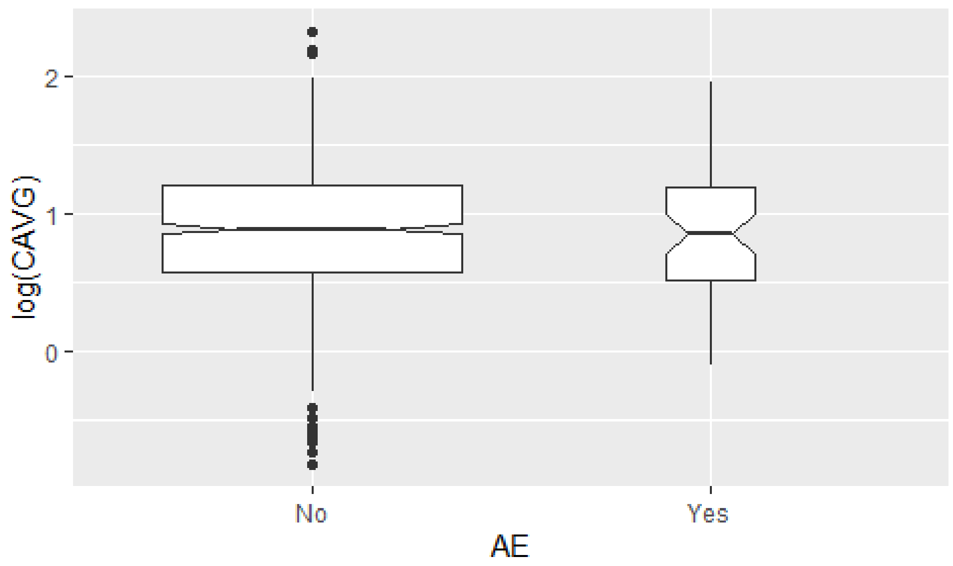


```
## Linear model for log(Cavg) vs AE and Regimen
## [1] 0.8713343
```

No evidence of E-R.

S3.2.6 Gastrointestinal symptoms

```
## Distribution of all events by grade:
##   1   2   3 Sum
## 293  48   3 344
## Number of first events
## [1] 185
```

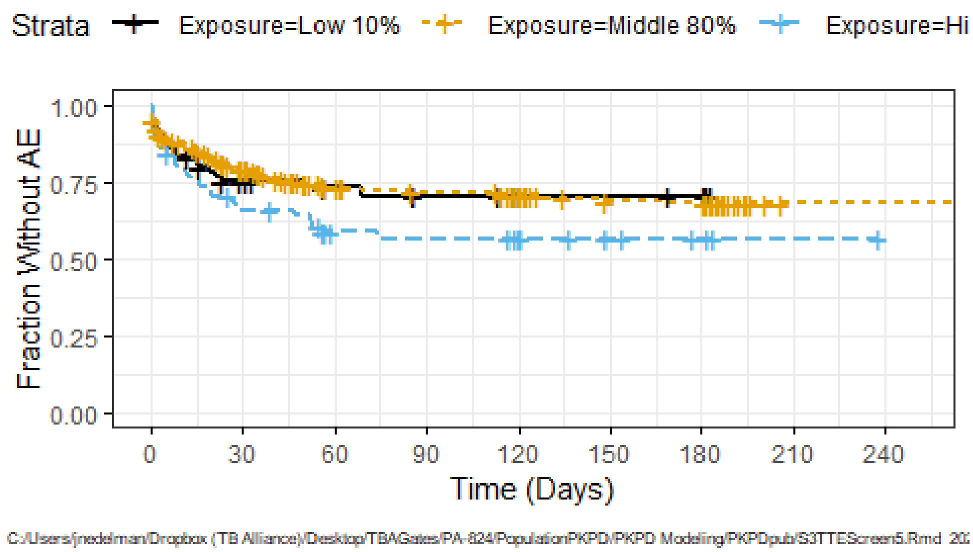


```
## Cox model with regimen and dLCavg, p-value for dLCavg
## [1] 0.1387596
```

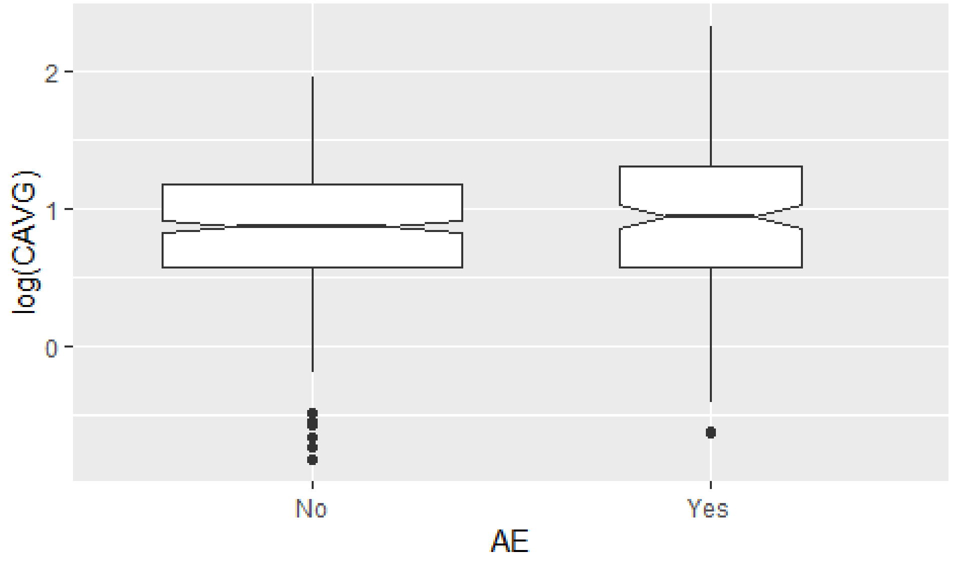


```
## Linear model for log(Cavg) vs AE and Regimen
## [1] 0.1863455
```

Evidence of E-R (K-M plots).

S3.2.7 Hepatic disorders

```
## Distribution of all events by grade:
##   1   2   3   4 Sum
## 117  74 131  64 386
## Number of first events
## [1] 164
```

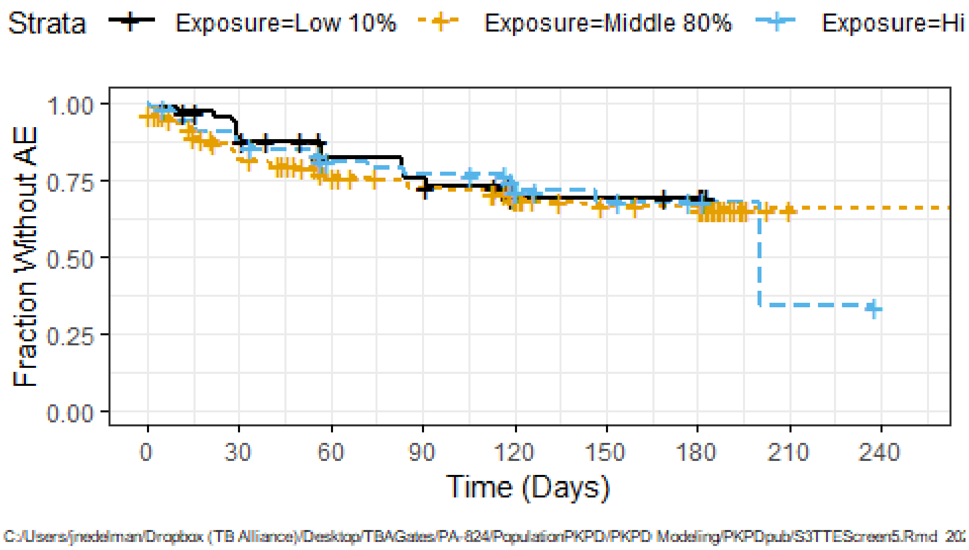


```
## Cox model with regimen and dLCavg, p-value for dLCavg
## [1] 0.6744549
```

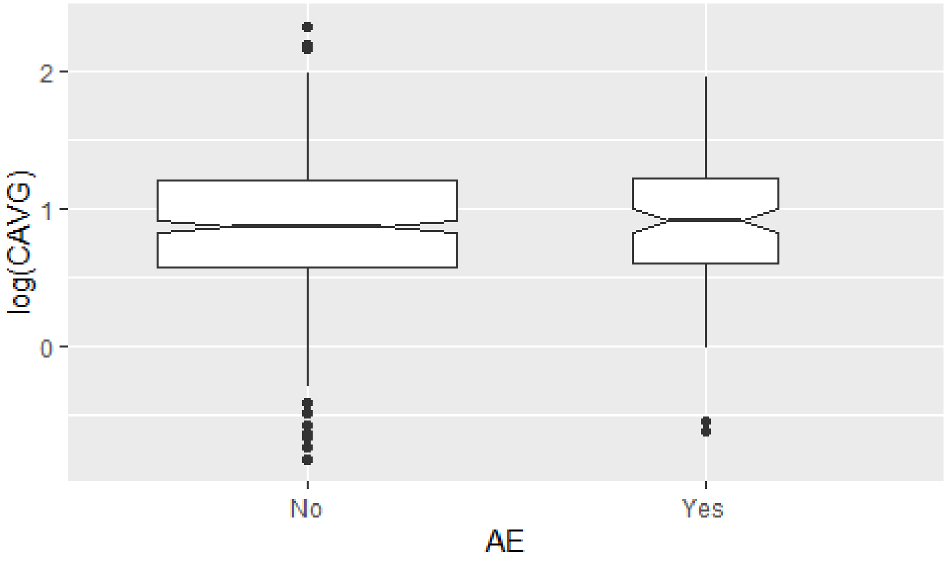


```
## Linear model for log(Cavg) vs AE and Regimen
## [1] 0.5728203
```

No evidence of E-R.

S3.2.8 Transaminases Increased

```
## Distribution of all events by grade:
##   1   2   3   4 Sum
##  63  45  96  43 247
## Number of first events
## [1] 123
```

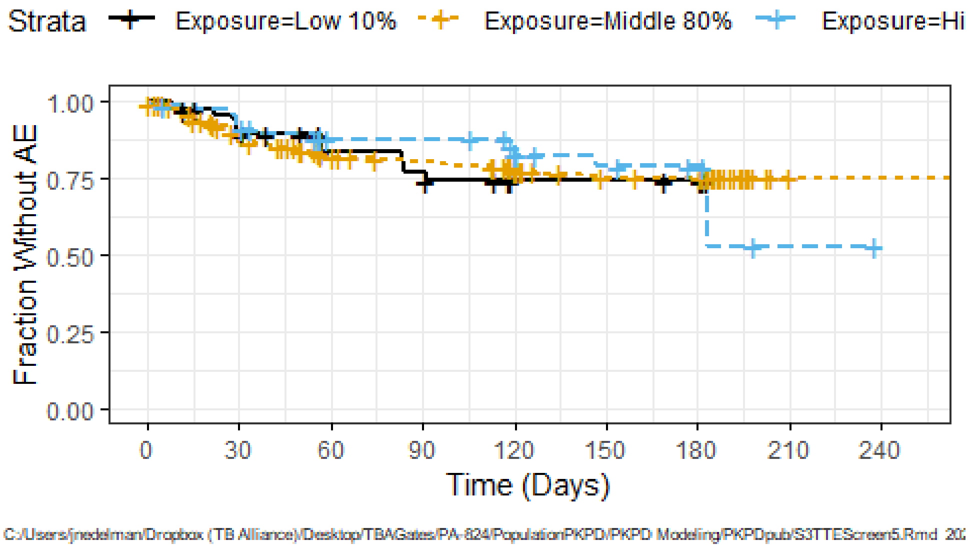


```
## Cox model with regimen and dLCavg, p-value for dLCavg
## [1] 0.4059835
```

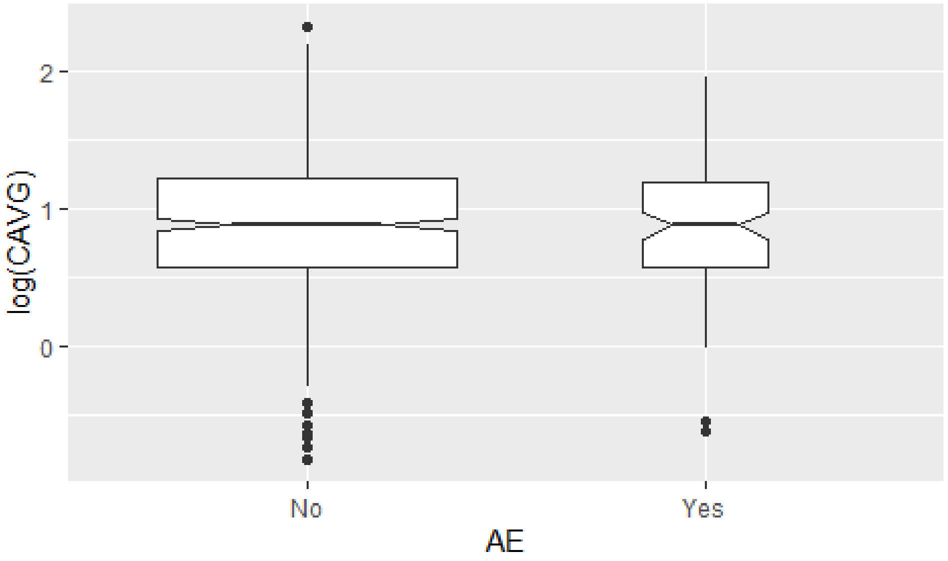


```
## Linear model for log(Cavg) vs AE and Regimen
## [1] 0.7884028
```

No evidence of E-R.

S3.2.9 Skin and subcutaneous tissue disorders

```
## Distribution of all events by grade:
##   1   2 Sum
## 128  11 139
## Number of first events
## [1] 108
```

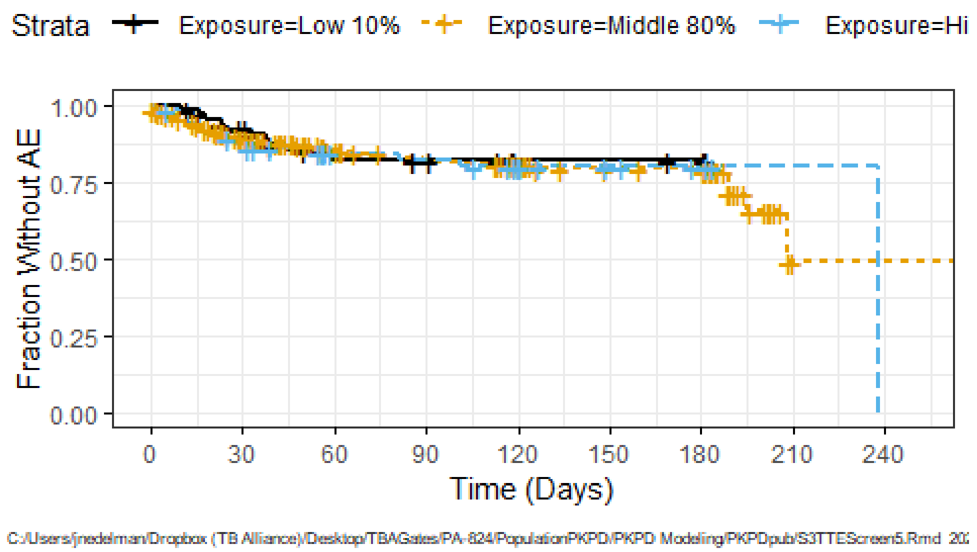


```
## Cox model with regimen and dLCavg, p-value for dLCavg
## [1] 0.9575519
```

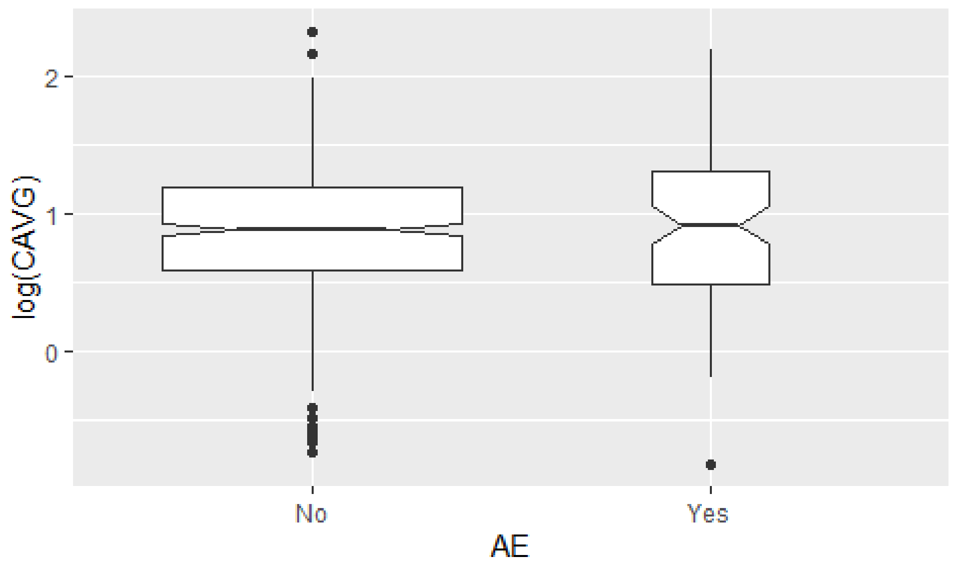


```
## Linear model for log(Cavg) vs AE and Regimen
## [1] 0.9712003
```

No evidence of E-R.

S3.2.10 Headaches

```
## Distribution of all events by grade:
##    1   2   3 Sum
##   60  16   2  78
## Number of first events
## [1] 72
```

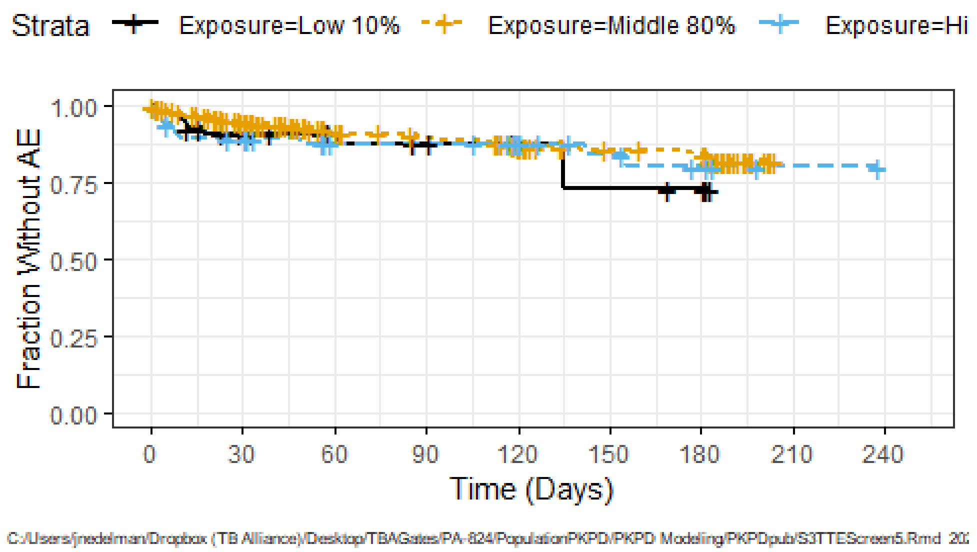


```
## Cox model with regimen and dLCavg, p-value for dLCavg
## [1] 0.6541334
```

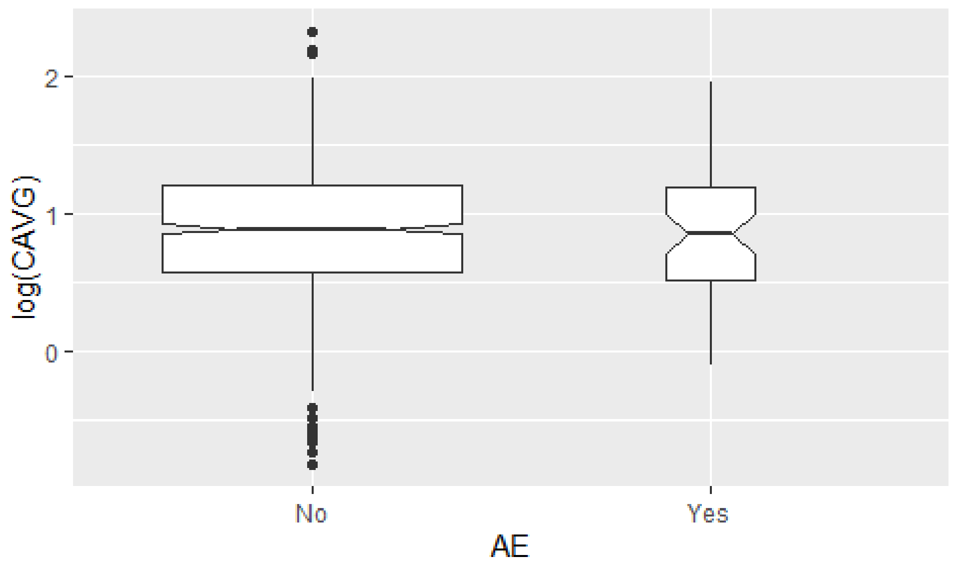


```
## Linear model for log(Cavg) vs AE and Regimen
## [1] 0.8713343
```

No evidence of E-R.

S4. Time to first event of Vomiting

Step 1 of the methodology was conducted when all AE sets were screened, S1. Start here with Step 2.

S4.1 Step 2 Covariate selection

Regimen and a 4-df spline of centered, log-transformed Cavg (dLCavg) are included in all models.

Model 1: dLAge, dLBMI, FEMALE, HIV, Type

```
## Call:
## coxph(formula = Surv(time, status) ~ Regimen + dLAge + dLBMI +
##     FEMALE + HIV + Type + X1 + X2 + X3 + X4, data = spl)
##
##                  coef exp(coef) se(coef)      z      p
## RegimenBPaMZ -0.47663   0.62087  0.49718 -0.959 0.3377
## RegimenBPaZ  -1.09734   0.33376  0.70721 -1.552 0.1207
## RegimenPaMZ  -0.26569   0.76668  0.51433 -0.517 0.6055
## dLAge        -0.38658   0.67937  0.35216 -1.098 0.2723
## dLBMI        -0.40657   0.66593  0.55057 -0.738 0.4602
## FEMALE        0.59998   1.82208  0.23262  2.579 0.0099
## HIV           0.34463   1.41146  0.24005  1.436 0.1511
## Type2MDR     -0.30028   0.74061  0.36411 -0.825 0.4096
## Type3DS      -0.63778   0.52846  0.57623 -1.107 0.2684
## X1           -0.20221   0.81692  1.00939 -0.200 0.8412
## X2            0.03293   1.03348  0.69686  0.047 0.9623
## X3           -0.59381   0.55222  2.20441 -0.269 0.7876
## X4            1.70000   5.47395  0.83849  2.027 0.0426
##
## Likelihood ratio test=55.34  on 13 df, p=3.523e-07
## n= 644, number of events= 96
```

FEMALE is significant, but not Age, BMI, HIV, or Type.

Model 2: FEMALE, then ANOVA vs Model 1

```
## Call:
## coxph(formula = Surv(time, status) ~ Regimen + FEMALE + X1 +
##     X2 + X3 + X4, data = spl)
##
##                 coef exp(coef) se(coef)      z        p
## RegimenBPaMZ -0.6223    0.5367   0.4204 -1.480 0.138795
## RegimenBPaZ  -1.7112    0.1806   0.4851 -3.528 0.000419
## RegimenPaMZ  -0.8299    0.4361   0.2284 -3.633 0.000280
## FEMALE        0.6852    1.9842   0.2157  3.176 0.001493
## X1           -0.3702    0.6906   0.9701 -0.382 0.702730
## X2           -0.1390    0.8702   0.6740 -0.206 0.836601
## X3           -0.8419    0.4309   2.1358 -0.394 0.693454
## X4            1.8330    6.2523   0.8252  2.221 0.026339
##
```

```
## Likelihood ratio test=50.56  on 8 df, p=3.188e-08
## n= 644, number of events= 96
```

```
## Analysis of Deviance Table
##  Cox model: response is  Surv(time, status)
##  Model 1: ~ Regimen + dLAge + dLBMI + FEMALE + HIV + Type + X1 + X2 + X3 +
X4
##  Model 2: ~ Regimen + FEMALE + X1 + X2 + X3 + X4
##    loglik  Chisq Df P(>|Chi|)
## 1 -570.03
## 2 -572.42 4.7778  5    0.4436
```

This confirms the non-significance of Age, BMI, HIV, and Type.

Test proportional hazards in Model 2

```
##              chisq df      p
## Regimen   2.33565  3 0.5057
## FEMALE    8.85442  1 0.0029
## X1        1.75982  1 0.1846
## X2        1.68160  1 0.1947
## X3        3.84490  1 0.0499
## X4        0.00157  1 0.9684
## GLOBAL   19.33026  8 0.0132
```

Proportionality appears to be violated by FEMALE; stratify.

Model 3: Stratification by gender

```
## Call:
## coxph(formula = Surv(time, status) ~ Regimen + strata(FEMALE) +
##     X1 + X2 + X3 + X4, data = spl)
##
##                   coef exp(coef) se(coef)      z        p
## RegimenBPaMZ -0.6076    0.5447   0.4204 -1.445 0.148389
## RegimenBPaZ  -1.6799    0.1864   0.4853 -3.462 0.000537
## RegimenPaMZ  -0.8230    0.4391   0.2279 -3.611 0.000305
## X1           -0.4020    0.6690   0.9671 -0.416 0.677615
## X2           -0.1490    0.8616   0.6703 -0.222 0.824075
## X3           -0.9383    0.3913   2.1289 -0.441 0.659386
## X4            1.8025    6.0647   0.8215  2.194 0.028225
##
## Likelihood ratio test=28.3  on 7 df, p=0.000194
## n= 644, number of events= 96
```

Assess proportional hazards for model 3

```
##            chisq df     p
## Regimen   3.586  3 0.310
## X1        0.861  1 0.353
## X2        4.071  1 0.044
## X3        4.247  1 0.039
## X4        0.517  1 0.472
## GLOBAL   10.863  7 0.145
```

Slight evidence for possible deviation from PH for exposure, but global test is not significant. Keep this model moving forward.

S4.2 Step 3 Covariate interactions

Model 4: Test interaction of regimen and exposure

```
## Call:
## coxph(formula = Surv(time, status) ~ Regimen + strata(FEMALE) +
##     X1 + X2 + X3 + X4 + X1:Regimen + X2:Regimen + X3:Regimen +
##     X4:Regimen, data = spl)
##
##                       coef  exp(coef)   se(coef)      z      p
## RegimenBPaMZ     4.378e+01  1.031e+19  3.809e+01  1.150 0.2503
## RegimenBPaZ      4.337e+01  6.839e+18  3.504e+01  1.238 0.2158
## RegimenPaMZ      4.978e+01  4.176e+21  3.280e+01  1.518 0.1290
## X1               4.970e+01  3.857e+21  3.251e+01  1.529 0.1263
## X2               2.998e+01  1.044e+13  1.931e+01  1.553 0.1205
## X3               9.795e+01  3.445e+42  6.409e+01  1.528 0.1264
## X4               2.567e+01  1.401e+11  1.514e+01  1.696 0.0899
## RegimenBPaMZ:X1 -4.405e+01  7.400e-20  3.752e+01 -1.174 0.2404
## RegimenBPaZ:X1  -4.783e+01  1.694e-21  3.447e+01 -1.388 0.1652
## RegimenPaMZ:X1  -5.001e+01  1.919e-22  3.253e+01 -1.537 0.1242
## RegimenBPaMZ:X2 -2.700e+01  1.880e-12  2.294e+01 -1.177 0.2393
## RegimenBPaZ:X2  -2.345e+01  6.554e-11  2.114e+01 -1.109 0.2674
## RegimenPaMZ:X2  -3.062e+01  5.021e-14  1.932e+01 -1.585 0.1130
## RegimenBPaMZ:X3 -8.322e+01  7.180e-37  7.446e+01 -1.118 0.2637
## RegimenBPaZ:X3  -9.120e+01  2.462e-40  6.897e+01 -1.322 0.1861
## RegimenPaMZ:X3  -9.906e+01  9.547e-44  6.413e+01 -1.545 0.1224
## RegimenBPaMZ:X4 -1.476e+01  3.871e-07  2.079e+01 -0.710 0.4775
## RegimenBPaZ:X4  -4.073e+01  2.052e-18  2.572e+01 -1.584 0.1133
## RegimenPaMZ:X4  -2.424e+01  2.972e-11  1.519e+01 -1.596 0.1105
##
## Likelihood ratio test=42.5  on 19 df, p=0.001514
## n= 644, number of events= 96

## Analysis of Deviance Table
##  Cox model: response is  Surv(time, status)
##  Model 1: ~ Regimen + strata(FEMALE) + X1 + X2 + X3 + X4
##  Model 2: ~ Regimen + strata(FEMALE) + X1 + X2 + X3 + X4 + X1:Regimen +
X2:Regimen + X3:Regimen + X4:Regimen
##    loglik  Chisq Df P(>|Chi|)
## 1 -506.54
## 2 -499.44 14.205 12    0.2878
```

Model 5: Test interaction of regimen and FEMALE

```
## Call:
## coxph(formula = Surv(time, status) ~ Regimen + strata(FEMALE) +
##     X1 + X2 + X3 + X4 + strata(FEMALE):Regimen, data = spl)
##
##                                         coef exp(coef) se(coef)      z       p
## RegimenBPaMZ                        -1.46623   0.23079  0.75954 -1.930 0.05355
## RegimenBPaZ                         -3.00214   0.04968  1.03770 -2.893 0.00382
## RegimenPaMZ                         -0.94075   0.39033  0.33069 -2.845 0.00444
## X1                                  -0.41604   0.65966  0.96573 -0.431 0.66661
## X2                                  -0.13329   0.87521  0.67025 -0.199 0.84236
## X3                                  -0.95984   0.38295  2.12806 -0.451 0.65196
## X4                                   1.88431   6.58180  0.82213  2.292 0.02191
## RegimenBPaMZ:strata(FEMALE)FEMALE=1  1.44536   4.24337  0.91130  1.586 0.11273
## RegimenBPaZ:strata(FEMALE)FEMALE=1   2.16764   8.73762  1.17329  1.847 0.06468
## RegimenPaMZ:strata(FEMALE)FEMALE=1   0.15331   1.16568  0.45118  0.340 0.73402
##
## Likelihood ratio test=34.91  on 10 df, p=0.0001295
## n= 644, number of events= 96

## Analysis of Deviance Table
##  Cox model: response is  Surv(time, status)
##  Model 1: ~ Regimen + strata(FEMALE) + X1 + X2 + X3 + X4
##  Model 2: ~ Regimen + strata(FEMALE) + X1 + X2 + X3 + X4 + strata(FEMALE):Regimen
##    loglik  Chisq Df P(>|Chi|)
## 1 -506.54
## 2 -503.24 6.6068  3   0.08554 .
## ---
## Signif. codes:  0 '***' 0.001 '**' 0.01 '*' 0.05 '.' 0.1 ' ' 1
```

Model 6: Test interaction of exposure and FEMALE

```
## Call:
## coxph(formula = Surv(time, status) ~ Regimen + strata(FEMALE) +
##     X1 + X2 + X3 + X4 + X1:strata(FEMALE) + X2:strata(FEMALE) +
##     X3:strata(FEMALE) + X4:strata(FEMALE), data = spl)
##
##                              coef exp(coef) se(coef)      z        p
## RegimenBPaMZ              -0.6709    0.5112   0.4213 -1.593 0.111254
## RegimenBPaZ               -1.6980    0.1831   0.4857 -3.496 0.000472
## RegimenPaMZ               -0.8944    0.4089   0.2313 -3.867 0.000110
## X1                        -0.8699    0.4190   1.2902 -0.674 0.500147
## X2                         0.3126    1.3670   0.9799  0.319 0.749703
## X3                        -2.1873    0.1122   2.9299 -0.747 0.455339
## X4                         2.4215   11.2628   1.4608  1.658 0.097376
## strata(FEMALE)FEMALE=1:X1  0.8352    2.3053   1.9746  0.423 0.672314
## strata(FEMALE)FEMALE=1:X2 -0.8516    0.4267   1.3956 -0.610 0.541744
## strata(FEMALE)FEMALE=1:X3  2.6216   13.7574   4.4061  0.595 0.551850
## strata(FEMALE)FEMALE=1:X4 -0.9805    0.3751   1.7957 -0.546 0.585023
##
## Likelihood ratio test=34.3  on 11 df, p=0.0003228
## n= 644, number of events= 96

## Analysis of Deviance Table
##  Cox model: response is  Surv(time, status)
##  Model 1: ~ Regimen + strata(FEMALE) + X1 + X2 + X3 + X4
##  Model 2: ~ Regimen + strata(FEMALE) + X1 + X2 + X3 + X4 +
X1:strata(FEMALE) + X2:strata(FEMALE) + X3:strata(FEMALE) + X4:strata(FEMALE)
##    loglik  Chisq Df P(>|Chi|)
## 1 -506.54
## 2 -503.54 6.0018  4     0.199
```

No interactions are retained.

Step 4 of the methodology is skipped, because no covariates had missing values.

S4.3 Step 5: Optimize functional form of Cavg

The best model so far, Model 3, represents exposure as a 4-df spline of dLCavg. Try models with 1-, 2-, and 3-df splines, and examine AICs.

```
##           df      AIC
## df1model   4 1024.169
## df2model   5 1024.515
## df3model   6 1026.493
## model3     7 1027.084
```

The best model is linear in (a 1-df spline for) dLCavg. Assess proportional hazards:

```
##          chisq df    p
## Regimen   3.28  3 0.35
```

```
## X1        2.28  1 0.13
## GLOBAL    6.51  4 0.16
```

Looks OK. As the final model, carry forward an equivalent model with dLCavg as the predictor. Here are the parameter estimates for that model:

```
## Call:
## coxph(formula = Surv(time, status) ~ Regimen + strata(FEMALE) +
##     dLCavg, data = spl)
##
##                  coef exp(coef) se(coef)      z        p
## RegimenBPaMZ -0.6153    0.5405   0.4193 -1.468 0.142224
## RegimenBPaZ  -1.6856    0.1853   0.4838 -3.484 0.000494
## RegimenPaMZ  -0.7859    0.4557   0.2239 -3.510 0.000448
## dLCavg        0.5088    1.6632   0.2261  2.250 0.024443
##
## Likelihood ratio test=25.22  on 4 df, p=4.553e-05
## n= 644, number of events= 96
```

S4.4 Hazard ratios from the final model

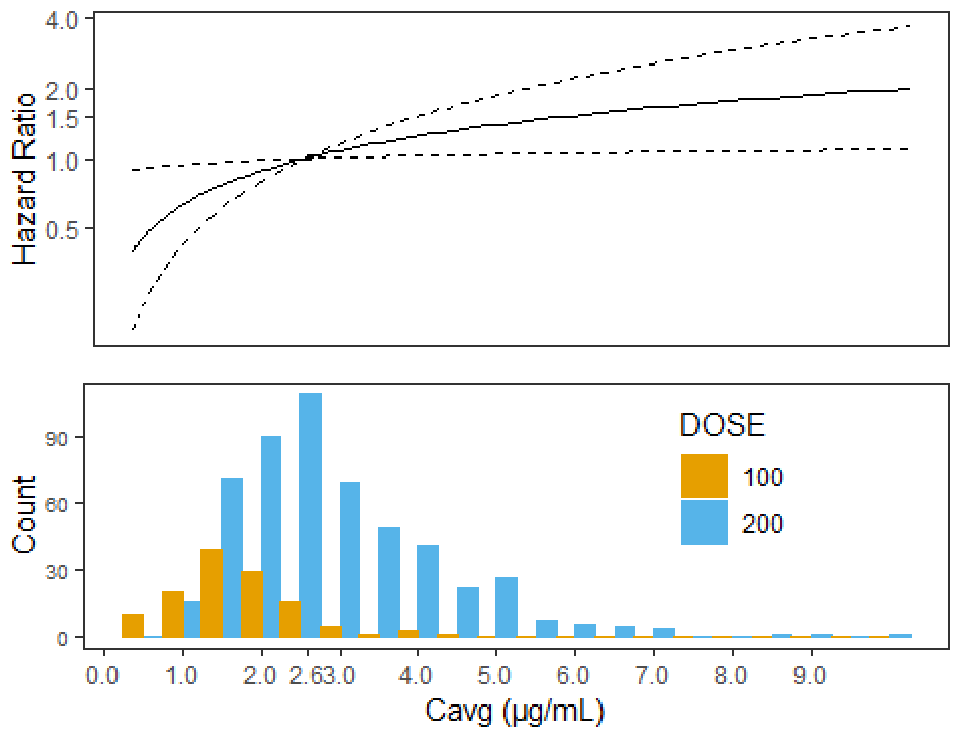

C:/Users/jnedelman/Dropbox (TB Alliance)/Desktop/TBAGates/PA-824/PopulationPKPD/PKPD Modeling/PKPDpub/S4Final.Rmd 2020-07-2

S4.5 Probability of at least one Vomiting event by 6 months

Males

```
##              Gender 95% CI Lower Estimate 95% CI Upper
## 100 mg 10%    Male         0.06     0.15         0.24
```

```
## 100 mg 50%   Male          0.10     0.20         0.28
## 200 mg 10%   Male          0.12     0.21         0.30
## 200 mg 50%   Male          0.17     0.27         0.36
```

Females

```
##            Gender 95% CI Lower Estimate 95% CI Upper
## 100 mg 10% Female         0.08     0.24         0.37
## 100 mg 50% Female         0.16     0.30         0.42
## 200 mg 10% Female         0.19     0.32         0.44
## 200 mg 50% Female         0.28     0.40         0.51
```

S5. Time to first event of GI Symptoms

Step 1 of the methodology was conducted when all AE sets were screened, S1. Start here with Step 2.

S5.1 Step 2 Covariate selection

Regimen and a 4-df spline of centered, log-transformed Cavg (dLCavg) are included in all models.

Model 1: dLAge, dLBMI, FEMALE, HIV, Type

```
## Call:
## coxph(formula = Surv(time, status) ~ Regimen + dLAge + dLBMI +
##     FEMALE + HIV + Type + X1 + X2 + X3 + X4, data = spl)
##
##                    coef exp(coef) se(coef)      z        p
## RegimenBPaMZ -0.68497   0.50410  0.36694 -1.867 0.061940
## RegimenBPaZ  -1.52879   0.21680  0.50206 -3.045 0.002327
## RegimenPaMZ  -0.23776   0.78839  0.37189 -0.639 0.522602
## dLAge        -0.02456   0.97574  0.24380 -0.101 0.919754
## dLBMI        -0.51435   0.59789  0.41393 -1.243 0.214014
## FEMALE        0.60064   1.82329  0.16739  3.588 0.000333
## HIV          -0.06445   0.93759  0.17821 -0.362 0.717618
## Type2MDR      0.10475   1.11043  0.29065  0.360 0.718562
## Type3DS      -0.08588   0.91770  0.42515 -0.202 0.839917
## X1           -0.11282   0.89331  0.68661 -0.164 0.869487
## X2           -0.53255   0.58711  0.50947 -1.045 0.295888
## X3            0.02320   1.02347  1.51865  0.015 0.987813
## X4            1.32161   3.74944  0.67746  1.951 0.051076
##
## Likelihood ratio test=58.8  on 13 df, p=8.605e-08
## n= 644, number of events= 185
```

Female is significant, but not Age, BMI, HIV, or Type.

Model 2: FEMALE, then ANOVA vs Model 1

```
## Call:
## coxph(formula = Surv(time, status) ~ Regimen + FEMALE + X1 +
```

```
##     X2 + X3 + X4, data = spl)
##
##                     coef exp(coef)  se(coef)      z        p
## RegimenBPaMZ -0.565213  0.568239  0.313600 -1.802 0.071492
## RegimenBPaZ  -1.620115  0.197876  0.349957 -4.629 3.67e-06
## RegimenPaMZ  -0.313312  0.731022  0.172744 -1.814 0.069719
## FEMALE        0.542559  1.720404  0.154071  3.521 0.000429
## X1            0.009587  1.009633  0.668071  0.014 0.988550
## X2           -0.418229  0.658212  0.490726 -0.852 0.394066
## X3            0.245165  1.277832  1.494769  0.164 0.869719
## X4            1.443939  4.237352  0.667202  2.164 0.030451
##
## Likelihood ratio test=56.6  on 8 df, p=2.158e-09
## n= 644, number of events= 185
```

```
## Analysis of Deviance Table
##  Cox model: response is  Surv(time, status)
##  Model 1: ~ Regimen + dLAge + dLBMI + FEMALE + HIV + Type + X1 + X2 + X3 +
X4
##  Model 2: ~ Regimen + FEMALE + X1 + X2 + X3 + X4
##    loglik  Chisq Df P(>|Chi|)
## 1 -1120.0
## 2 -1121.2 2.2017  5    0.8206
```

This confirms the non-significance of Age, BMI, HIV, and Type.

Test proportional hazards in Model 2

```
##            chisq df       p
## Regimen 12.87040  3 0.00493
## FEMALE   3.52986  1 0.06027
## X1       1.86285  1 0.17230
## X2       4.61140  1 0.03176
## X3       0.00152  1 0.96887
## X4       0.16335  1 0.68609
## GLOBAL  31.39950  8 0.00012
```

Proportionality appears to be violated by both Regimen, and possibly by FEMALE. Try stratfying on Regimen.

Model 3: Stratify on Regimen, check PH

```
## Call:
## coxph(formula = Surv(time, status) ~ strata(Regimen) + FEMALE +
##     X1 + X2 + X3 + X4, data = spl)
##
##            coef exp(coef) se(coef)      z        p
## FEMALE  0.54668   1.72750  0.15423  3.544 0.000393
## X1      0.05781   1.05951  0.68201  0.085 0.932454
## X2     -0.46197   0.63004  0.50430 -0.916 0.359636
## X3      0.22243   1.24911  1.52414  0.146 0.883968
## X4      1.71183   5.53910  0.69952  2.447 0.014399
```

```
##
## Likelihood ratio test=19.45  on 5 df, p=0.001585
## n= 644, number of events= 185
```

```
##           chisq df      p
## FEMALE  5.11738  1 0.0237
## X1      2.82350  1 0.0929
## X2      6.34954  1 0.0117
## X3      0.00382  1 0.9507
## X4      0.46024  1 0.4975
## GLOBAL 17.37179  5 0.0038
```

PH appears violated for FEMALE and exposure. Try stratifying jointly on Regimen and FEMALE.

Model 4: Stratify jointly on Regimen and FEMALE, check PH

```
## Call:
## coxph(formula = Surv(time, status) ~ strata(Regimen, FEMALE) +
##     X1 + X2 + X3 + X4, data = spl)
##
##        coef exp(coef) se(coef)      z      p
## X1  0.01316   1.01324  0.68189  0.019 0.9846
## X2 -0.43997   0.64406  0.50296 -0.875 0.3817
## X3  0.08260   1.08610  1.52629  0.054 0.9568
## X4  1.56869   4.80034  0.69437  2.259 0.0239
##
## Likelihood ratio test=4.91  on 4 df, p=0.2964
## n= 644, number of events= 185
```

```
##           chisq df     p
## X1     4.32e+00  1 0.038
## X2     8.83e+00  1 0.003
## X3     5.25e-05  1 0.994
## X4     9.97e-01  1 0.318
## GLOBAL 1.25e+01  4 0.014
```

PH appears violated for exposure, but carry this model forward in the hope that interactions and/or reduction of the spline df will ameliorate this.

S5.2 Step 3 Covariate interactions

Model 5:Test interactions of the strata with exposure

```
## Warning in fitter(X, Y, istrat, offset, init, control, weights = weights, : Ran
## out of iterations and did not converge

## Warning in fitter(X, Y, istrat, offset, init, control, weights = weights, : one
## or more coefficients may be infinite

## Call:
## coxph(formula = Surv(time, status) ~ strata(Regimen, FEMALE) +
##     X1 + X2 + X3 + X4 + strata(FEMALE, Regimen):X1 + strata(FEMALE,
##     Regimen):X2 + strata(FEMALE, Regimen):X3 + strata(FEMALE,
##     Regimen):X4, data = spl)
##
##                                                          coef   exp(coef)
## X1                                                  2.249e+01   5.840e+09
## X2                                                  1.384e+01   1.029e+06
## X3                                                  4.667e+01   1.856e+20
## X4                                                  1.156e+01   1.048e+05
## X1:strata(FEMALE, Regimen)FEMALE=0, Regimen=BPaMZ   6.946e+02  4.623e+301
## X1:strata(FEMALE, Regimen)FEMALE=0, Regimen=BPaZ    1.023e+01   2.782e+04
## X1:strata(FEMALE, Regimen)FEMALE=0, Regimen=PaMZ   -2.274e+01   1.326e-10
## X1:strata(FEMALE, Regimen)FEMALE=1, Regimen=BPaL    7.402e+01   1.405e+32
## X1:strata(FEMALE, Regimen)FEMALE=1, Regimen=BPaMZ  -2.897e+00   5.519e-02
## X1:strata(FEMALE, Regimen)FEMALE=1, Regimen=BPaZ   -4.511e+01   2.560e-20
## X1:strata(FEMALE, Regimen)FEMALE=1, Regimen=PaMZ   -2.215e+01   2.412e-10
## X2:strata(FEMALE, Regimen)FEMALE=0, Regimen=BPaMZ   4.476e+02  2.402e+194
## X2:strata(FEMALE, Regimen)FEMALE=0, Regimen=BPaZ    1.495e+01   3.115e+06
## X2:strata(FEMALE, Regimen)FEMALE=0, Regimen=PaMZ   -1.459e+01   4.622e-07
## X2:strata(FEMALE, Regimen)FEMALE=1, Regimen=BPaL    4.370e+01   9.500e+18
## X2:strata(FEMALE, Regimen)FEMALE=1, Regimen=BPaMZ  -5.982e+00   2.523e-03
## X2:strata(FEMALE, Regimen)FEMALE=1, Regimen=BPaZ   -2.666e+01   2.638e-12
## X2:strata(FEMALE, Regimen)FEMALE=1, Regimen=PaMZ   -1.509e+01   2.791e-07
## X3:strata(FEMALE, Regimen)FEMALE=0, Regimen=BPaMZ   1.021e+03         Inf
```

```
## X3:strata(FEMALE, Regimen)FEMALE=0, Regimen=BPaZ    1.803e+01   6.766e+07
## X3:strata(FEMALE, Regimen)FEMALE=0, Regimen=PaMZ   -4.741e+01   2.574e-21
## X3:strata(FEMALE, Regimen)FEMALE=1, Regimen=BPaL    1.436e+02   2.385e+62
## X3:strata(FEMALE, Regimen)FEMALE=1, Regimen=BPaMZ   1.677e+00   5.347e+00
## X3:strata(FEMALE, Regimen)FEMALE=1, Regimen=BPaZ   -8.906e+01   2.094e-39
## X3:strata(FEMALE, Regimen)FEMALE=1, Regimen=PaMZ   -4.612e+01   9.336e-21
## X4:strata(FEMALE, Regimen)FEMALE=0, Regimen=BPaMZ  -1.903e+02   2.232e-83
## X4:strata(FEMALE, Regimen)FEMALE=0, Regimen=BPaZ   -3.038e+01   6.382e-14
## X4:strata(FEMALE, Regimen)FEMALE=0, Regimen=PaMZ   -1.072e+01   2.205e-05
## X4:strata(FEMALE, Regimen)FEMALE=1, Regimen=BPaL    3.510e+01   1.749e+15
## X4:strata(FEMALE, Regimen)FEMALE=1, Regimen=BPaMZ   1.636e+01   1.268e+07
## X4:strata(FEMALE, Regimen)FEMALE=1, Regimen=BPaZ   -2.603e+01   4.968e-12
## X4:strata(FEMALE, Regimen)FEMALE=1, Regimen=PaMZ   -9.217e+00   9.934e-05
##                                                     se(coef)       z        p
## X1                                                 2.845e-01  79.052  < 2e-16
## X2                                                 2.876e-01  48.143  < 2e-16
## X3                                                 6.458e-01  72.268  < 2e-16
## X4                                                 5.284e-01  21.878  < 2e-16
## X1:strata(FEMALE, Regimen)FEMALE=0, Regimen=BPaMZ   5.539e+00 125.406  < 2e-16
## X1:strata(FEMALE, Regimen)FEMALE=0, Regimen=BPaZ    3.007e+00   3.403 0.000666
## X1:strata(FEMALE, Regimen)FEMALE=0, Regimen=PaMZ    4.697e-01 -48.417  < 2e-16
## X1:strata(FEMALE, Regimen)FEMALE=1, Regimen=BPaL    6.860e-01 107.906  < 2e-16
## X1:strata(FEMALE, Regimen)FEMALE=1, Regimen=BPaMZ   1.957e+00  -1.480 0.138770
## X1:strata(FEMALE, Regimen)FEMALE=1, Regimen=BPaZ    2.471e+00 -18.254  < 2e-16
## X1:strata(FEMALE, Regimen)FEMALE=1, Regimen=PaMZ    5.118e-01 -43.271  < 2e-16
## X2:strata(FEMALE, Regimen)FEMALE=0, Regimen=BPaMZ   4.843e+00  92.416  < 2e-16
## X2:strata(FEMALE, Regimen)FEMALE=0, Regimen=BPaZ    1.460e+00  10.244  < 2e-16
## X2:strata(FEMALE, Regimen)FEMALE=0, Regimen=PaMZ    5.053e-01 -28.868  < 2e-16
## X2:strata(FEMALE, Regimen)FEMALE=1, Regimen=BPaL    7.644e-01  57.165  < 2e-16
## X2:strata(FEMALE, Regimen)FEMALE=1, Regimen=BPaMZ   1.711e+00  -3.496 0.000472
## X2:strata(FEMALE, Regimen)FEMALE=1, Regimen=BPaZ    1.855e+00 -14.370  < 2e-16
## X2:strata(FEMALE, Regimen)FEMALE=1, Regimen=PaMZ    5.246e-01 -28.765  < 2e-16
## X3:strata(FEMALE, Regimen)FEMALE=0, Regimen=BPaMZ   2.622e+01  38.944  < 2e-16
## X3:strata(FEMALE, Regimen)FEMALE=0, Regimen=BPaZ    3.496e+00   5.158 2.50e-07
## X3:strata(FEMALE, Regimen)FEMALE=0, Regimen=PaMZ    1.025e+00 -46.260  < 2e-16
## X3:strata(FEMALE, Regimen)FEMALE=1, Regimen=BPaL    2.121e+00  67.723  < 2e-16
```

```
## X3:strata(FEMALE, Regimen)FEMALE=1, Regimen=BPaMZ   3.657e+00   0.458 0.646632
## X3:strata(FEMALE, Regimen)FEMALE=1, Regimen=BPaZ    3.553e+00 -25.069  < 2e-16
## X3:strata(FEMALE, Regimen)FEMALE=1, Regimen=PaMZ    1.238e+00 -37.266  < 2e-16
## X4:strata(FEMALE, Regimen)FEMALE=0, Regimen=BPaMZ   7.717e+01  -2.466 0.013657
## X4:strata(FEMALE, Regimen)FEMALE=0, Regimen=BPaZ    4.900e+00  -6.201 5.61e-10
## X4:strata(FEMALE, Regimen)FEMALE=0, Regimen=PaMZ    1.064e+00 -10.076  < 2e-16
## X4:strata(FEMALE, Regimen)FEMALE=1, Regimen=BPaL    1.273e+00  27.578  < 2e-16
## X4:strata(FEMALE, Regimen)FEMALE=1, Regimen=BPaMZ   4.903e+00   3.336 0.000851
## X4:strata(FEMALE, Regimen)FEMALE=1, Regimen=BPaZ    5.120e+00  -5.084 3.70e-07
## X4:strata(FEMALE, Regimen)FEMALE=1, Regimen=PaMZ    8.488e-01 -10.858  < 2e-16
##
## Likelihood ratio test=50.16  on 32 df, p=0.02149
## n= 644, number of events= 185
```

Interactions of the strata with Cavg lead to a model that is over-parameterized: It does not converge, and reported parameter estimates are extremely large in magnitude with many implausible p-values.

Reduce to BPaL, the regimen of most interest, and repeat these initial steps.

S5.3 Step 2 Repeat – covariate selection restricted to BPaL

Regimen and a 4-df spline of centered, log-transformed Cavg (dLCavg) are included in all models.

Model 1b: dLAge, dLBMI, FEMALE, HIV, Type

```
## Call:
## coxph(formula = Surv(time, status) ~ dLAge + dLBMI + FEMALE +
##     HIV + Type + X1 + X2 + X3 + X4, data = splBPaL)
##
##                coef  exp(coef)   se(coef)      z      p
## dLAge     3.040e-03  1.003e+00  5.010e-01  0.006 0.9952
## dLBMI    -1.006e+00  3.658e-01  6.759e-01 -1.488 0.1368
## FEMALE    1.961e-01  1.217e+00  3.078e-01  0.637 0.5240
## HIV      -1.182e-01  8.885e-01  3.113e-01 -0.380 0.7041
## Type2MDR -1.501e-02  9.851e-01  3.017e-01 -0.050 0.9603
## X1        2.422e+01  3.296e+10  1.541e+01  1.571 0.1161
## X2        1.464e+01  2.284e+06  9.150e+00  1.600 0.1096
## X3        4.935e+01  2.715e+21  3.094e+01  1.595 0.1107
## X4        1.264e+01  3.072e+05  7.012e+00  1.802 0.0716
##
## Likelihood ratio test=16.27  on 9 df, p=0.06143
## n= 109, number of events= 53
```

No covariates are significant.

Model 2b: No covariates, then ANOVA vs Model 1

```
## Call:
## coxph(formula = Surv(time, status) ~ X1 + X2 + X3 + X4, data = splBPaL)
##
##         coef exp(coef)  se(coef)     z      p
## X1 2.496e+01 6.916e+10 1.510e+01 1.653 0.0983
## X2 1.518e+01 3.919e+06 8.935e+00 1.699 0.0893
## X3 5.080e+01 1.156e+22 3.028e+01 1.678 0.0934
## X4 1.318e+01 5.309e+05 6.877e+00 1.917 0.0552
##
## Likelihood ratio test=13.25  on 4 df, p=0.0101
## n= 109, number of events= 53

## Analysis of Deviance Table
##  Cox model: response is  Surv(time, status)
##  Model 1: ~ dLAge + dLBMI + FEMALE + HIV + Type + X1 + X2 + X3 + X4
##  Model 2: ~ X1 + X2 + X3 + X4
##    loglik  Chisq Df P(>|Chi|)
## 1 -225.08
## 2 -226.59 3.0183  5    0.6972
```

This confirms the non-significance of the covariates. Assess PH.

Test proportional hazards in Model 2b

```
##         chisq df     p
## X1      3.036  1 0.081
## X2      0.350  1 0.554
## X3      1.761  1 0.185
## X4      0.353  1 0.552
## GLOBAL 8.162  4 0.086
```

Satisfactory to carry forward.

Step 4 of the methodology is skipped, because no covariates had missing values.

S5.4 Step 5: Optimize functional form of Cavg

The best model so far, Model 2b, represents exposure as a 4-df spline of dLCavg. Try models with 1-, 2-, and 3-df splines, and examine AICs.

```
##           df      AIC
## df1model  1 459.5935
## df2model  2 461.5934
## df3model  3 461.3724
## model2b   4 461.1815
```

The best model is linear in (a 1-df spline for) dLCavg. Assess PH:

```
##         chisq df    p
## dLCavg   0.3  1 0.58
## GLOBAL   0.3  1 0.58
```

Looks OK. As the final model, carry forward an equivalent model with dLCavg as the predictor. Here are the parameter estimates for that model:

```
## Call:
## coxph(formula = Surv(time, status) ~ dLCavg, data = splBPaL)
##
##           coef exp(coef) se(coef)     z       p
## dLCavg 0.9376    2.5539   0.3134 2.991 0.00278
##
## Likelihood ratio test=8.84  on 1 df, p=0.002945
## n= 109, number of events= 53
```

S5.5 Hazard ratios from the final model

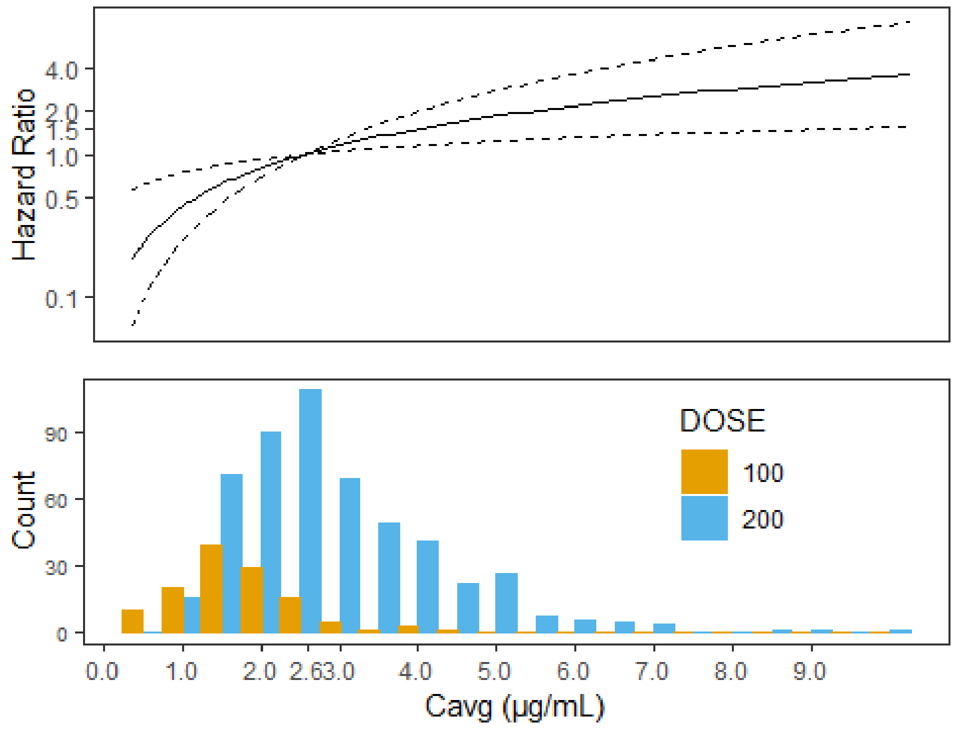

C:/Users/jnedelman/Dropbox (TB Alliance)/Desktop/TBAGates/PA-824/PopulationPKPD/PKPD Modeling/PKPDpub/S5GISymptoms.Rmd 20

S5.6 Probability of at least one Vomiting event by 6 months

```
##               95% CI Lower Estimate 95% CI Upper
## 100 mg 10%          0.03     0.19         0.32
## 100 mg 50%          0.14     0.29         0.42
## 200 mg 10%          0.19     0.33         0.44
## 200 mg 50%          0.38     0.49         0.57
```

References

Wallis RS, Peppard T, Hermann D. 2015. Month 2 culture status and treatment duration as predictors of recurrence in pulmonary tuberculosis: model validation and update. PLOS ONE 10:e0125403
